# Do experimental projection methods outcompete retention time prediction models in non-target screening? A case study on LC/HRMS interlaboratory comparison data†

**DOI:** 10.1039/d5an00323g

**Published:** 2025-07-08

**Authors:** Louise Malm, Anneli Kruve

**Affiliations:** a Department of Materials and Environmental Chemistry, Stockholm University 11418 Stockholm Sweden anneli.kruve@su.se; b Department of Environmental Science, Stockholm University 11418 Stockholm Sweden

## Abstract

Retention time (RT) is essential in evaluating the likelihood of candidate structures in nontarget screening (NTS) with liquid chromatography high resolution mass spectrometry (LC/HRMS). Approaches for estimating the RTs of candidate structures can broadly be divided into projection and prediction methods. The first approach takes advantage of public databases of RTs measured on similar chromatographic systems (CS_source_) and projects these to the chromatographic system applied in the NTS (CS_NTS_) based on a small set of commonly analyzed chemicals. The second approach leverages machine learning (ML) model(s) trained on publicly available retention time data measured on one or more chromatographic systems (CS_training_). Nevertheless, the CS_source_ and CS_training_ might differ substantially from CS_NTS_. Therefore, it is of interest to evaluate the generalizability of projection models and prediction models in CSs routinely applied in NTS. Here we take advantage of the recent NORMAN interlaboratory comparison where 41 known calibration chemicals and 45 suspects were analyzed to evaluate both the projection and prediction approaches on 37 CSs. The accuracy of both approaches was directly linked to the similarity of the CS, and the pH of the mobile phase and the column chemistry were found to be most impactful. Furthermore, for cases where CS_source_ and CS_NTS_ differ substantially but CS_training_ and CS_NTS_ are similar, prediction models often performed on par with the projection models. These findings highlight the need to account for the mobile phase and column chemistry in ML model training and select the prediction model for RT.

## Abstract 2

Retention time (RT) is essential in evaluating the likelihood of candidate structures in nontarget screening (NTS) with liquid chromatography high resolution mass spectrometry (LC/HRMS).

## Graphical abstract

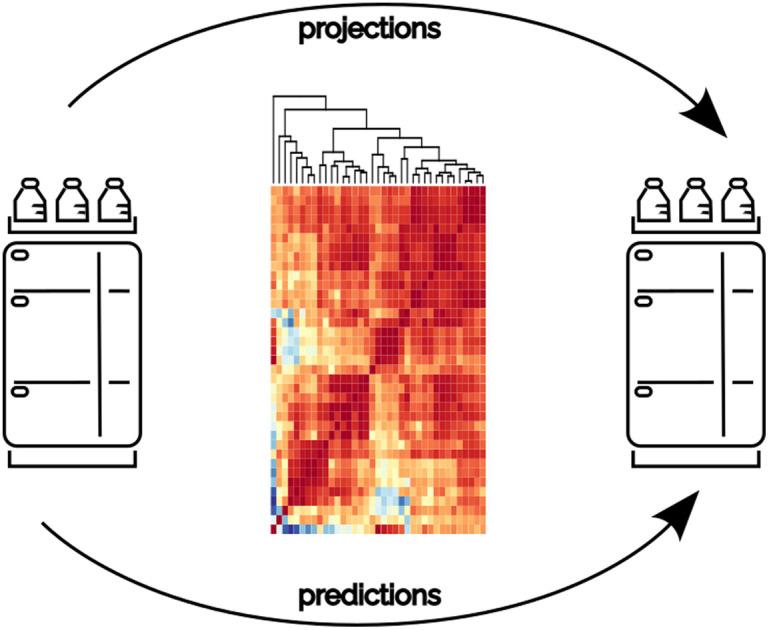

## 1. Introduction

Nontarget screening (NTS) with liquid chromatography high resolution mass spectrometry (LC/HRMS) is widely used for screening of chemicals in various samples. For example, NTS is used to pinpoint chemicals of concern in environmental and food samples and also to identify yet unknown metabolites or pharmaceutically active natural products.^1^ The aim of these methods is to both detect and identify suspected or yet unknown chemicals and the methods used to identify the detected chemicals leverage tandem mass spectra (MS^2^).^2^ Albeit information rich, MS^2^ spectra rarely indicate only one structure, and a combination of different strategies is needed to reduce the list of likely candidate structures.^3,4^ Here empirical analytical information and metadata about the sample can prove useful in prioritizing the correct candidate structure.

Regarding empirical analytical information, retention time (RT),^5–8^ collision cross-section (CCS),^9–11^ ionization mode, and adduct type^12,13^ have proven useful for discriminating candidate structures. Nevertheless, we^14^ and others^4,15^ have previously observed that RT has high potential in distinguishing between the candidate structures in terms of physical separation during analysis as well as evaluating candidate structures with a prediction model. This arises from the orthogonality of the chromatographic separation and HRMS data and indicates the high importance of incorporating RT in candidate structure prioritization workflows in NTS.

Comparing the RT of the detected LC/HRMS feature and the candidate structure can aid in prioritization by indicating less likely candidate structures, *e.g.*, the candidate structures for which the predicted RT differs from the experimentally observed RT more than the uncertainty limits. The RT of the candidate structures can be obtained by (1) projection of experimental reference retention times and (2) prediction of retention times based on the structure with machine learning (ML). The projection methods leverage RT databases^16,17^ of known structures measured on a different chromatographic system (CS_source_)^5^ and is applicable to chemicals already experimentally studied. Nevertheless, the CS_source_ and chromatographic system used in NTS (CS_NTS_) may differ due to equipment (dead volume, flow rate, and column length) as well as separation mechanisms (column chemistry and mobile phase). A small set of ten to 50 chemicals measured on both CSs can be used to fit a generalized additive model (GAM) or similar model between RTs from the source and target CS, which can account for some of these differences. Later, the same model is applied to project the RT of the chemicals measured with the CS_source_ to the CS_NTS_.

In the initialization and validation of a well-known projection approach, PredRet,^5^ it was observed that the number of chemicals commonly analyzed by both CSs and the similarity of the retention mechanism (reversed phase *vs.* HILIC) impact the projection accuracy. Alternatively, retention time indices (RTIs)^6,18,19^ have been suggested to account for the effect of column length, flow rate, and dead volume on RT. Furthermore, retention time order (RTO) is known to be more stable than RT or RTI if the same physical parameters of the CS are changed. Nevertheless, RTO is likely to change for nominally equal CSs for close eluting/co-eluting chemicals due to the variations in the peak shape or with different batches of LC columns^20^ as well as changing mobile phase composition when acids or bases are analysed.^21^ A recent comparison of RTOs of datasets collected into RepoRT^16^ revealed the largest variations for CSs with different columns and mobile phases. In spite of these considerations, an extensive unbiased overview of the applicability of projection methods across different CSs and laboratories is lacking.

The need for experimentally determined RTs can be overcome by ML models^6,8,9,22–32^ predicting RT, RTI, or RTO. Despite obvious advantages, the prediction approach is presumed to underperform projection approaches. Such approaches require a relatively large and representative^33^ dataset for model training and may require a close match of the CS used for collecting training data (CS_training_) and the CS_NTS_ or conversion of RT to RTIs with a predetermined calibration mix.^6^ To overcome this limitation, a complementing prediction approach with projection^5^ was suggested by Bouwmeester *et al.*^34^ and similar approaches have been successfully used thereafter.^24,35^ These approaches project the predicted RT or RTI to CS_NTS_ based on predicted RTs of experimentally measured standards even if commonly measured chemicals are lacking. Nevertheless, a recent comparison by Kwon *et al.*^36^ suggested a large performance variability and generally low performance of the combination of ML and projection methods. Though the exact reason remained unclear, it can be hypothesised that chemical space overlap and CS similarity affect the applicability. Similar to the study by Kwon *et al.*^36^ benchmarking different RT, RTI, or RTO prediction methods on public datasets has been widely discussed;^28^ however, the contribution of variability in chemical space and CSs remains hard to evaluate. In part, this is due to the sparse overlap in the chemical space considered in different studies leading to a set of data where both the chemical space and CSs often vary simultaneously.

Here we leverage the recent NORMAN interlaboratory comparison^37^ to evaluate the performance of the RTI projection and prediction approaches across different CSs commonly used in NTS for environmental analysis, while keeping the chemical space constant. In particular, we are interested in answering the following questions: (1) how different are the CSs in terms of agreement in RT, RTI, and RTO; (2) how do CSs affect the accuracy of the projection methods and how do this confine the best practices in NTS; and (3) how accurate are the prediction models in comparison with projection models. To answer these questions, firstly, we used the calibration chemicals to establish a GAM for the projection of RTIs from the CS_source_ to the CS_NTS_ and evaluate these on the suspected chemicals. Secondly, we trained an ML model to predict the retention time from the structure of the suspected chemicals and evaluated its performance based on the root mean square errors (RMSEs). Lastly, we compared the projection and prediction approaches and contextualized the findings based on the similarity of the CS_source_ and CS_training_ to the CS_NTS_.

## 2. Materials and methods

### 2.1 Data

The retention time data for 38 CSs were available from a recent NTS interlaboratory comparison with the NORMAN network.^37^ Two sets of chemicals, 41 calibrants and 45 suspects were analyzed with each CS and the number of detected chemicals ranged from 12 to 39 and 17 to 40, respectively. The experimental details for all CSs were published previously by Malm *et al.*^37^ In summary, the shortest chromatographic program was 15 min long, leading to RTs of 0.80 min to 9.80 min and the longest program was 54 min, leading to RTs of 11.70 to 36.95 min. All but one CS leveraged reversed phase chromatography (C_18_, C_8_, and C_6_-phenyl and biphenyl) with columns from all major vendors; all CSs used an acidic water phase containing only an acid or an acid with the respective ammonium salt as the mobile phase additive.

### 2.2 Generalized additive model for retention time index projection

To account for the fact that the set of detected calibration chemicals differed from one chromatographic system (CS) to another, the RTIs were calculated for each combination of CSs, so that only the calibration chemicals detected with both CSs were used. In order to allow a straightforward comparison of the performance of the projection and prediction models, independent of the length of the chromatographic program, column dimensions, *etc*., all retention times were converted to retention time indices as described previously by Aalizadeh *et al.*:^6^1
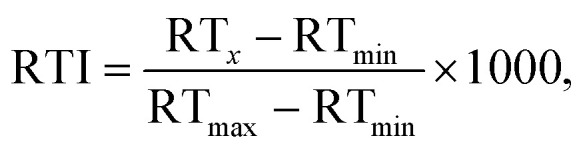
where RT_min_ and RT_max_ are the minimum and maximum retention times observed for the calibrants and RT_*x*_ is the retention time of the chemical of interest. This assures that the RTIs of calibrants for each comparison ranged from 0 to 1000. Scaling RT values between 1 and 1000 has been previously suggested to “have large RTI units between compounds that elute differently and compare the error more realistically” and “define the elution segment for the calibrants”.^6^

RTIs enabled accounting for differences in the flow rate and column length and thereby unifying the range of RTI values; however, the RTIs were sensitive to nonlinear gradient programs, that is, plateaus and changing gradient speeds. To account for such variations in the CSs, the RTI projection with a generalized additive model (GAM) was implemented. That is, the *gam* function from the *mgcv* package in R with smooth function “s” was used and the dimensions *k* for the smooth term was set to 6 for all projections. The GAMs were fitted on the calibration chemicals between the CS_NTS_ and CS_source_, and applied to predict the RTIs in the CS_NTS_ for the suspects based on the “known” RTIs in the CS_source_. Only suspects eluting in the calibration range were considered (RTI range 0 to 1000). Notably, the GAM minimizes the residual in the direction of the *y*-axis (like most models), leading to unsymmetrical projection accuracies and therefore all-to-all CS combinations were considered.

### 2.3 Retention time prediction models

A retention time prediction model was trained leveraging the RT data of environmentally relevant chemicals from Aalizadeh *et al.*^6^ measured with a 15.5 min long chromatographic program using a 5 mM ammonium acetate based buffer as the water phase and methanol as an organic modifier. Prior to modelling, the calibrants and suspects were removed from the dataset to avoid overfitting to these chemicals. For all remaining chemicals in both datasets the 2D and 3D Mordred descriptors were calculated from the mol representation after standardization with RDKit. As part of data cleaning, all descriptors yielding missing values for more than 15 chemicals were discarded, followed by removing all chemicals with any left missing values. Thereafter, the dataset contained retention times for 1795 chemicals and 1328 Mordred descriptors. Prior to modelling, the descriptors with near zero variance and descriptors with high correlation coefficients were removed with *nearZeroVar* (default settings) and *findCorrelated* (cutoff 0.7) functions from the *caret* package in R (version 4.3.2). An *xgbTree* model was trained to predict the RT. The hyperparameters were optimized with five times repeated two-fold cross-validation implemented through the *caret* package in R.

### 2.4 Statistical analysis

The similarity of RTIs across CSs was analyzed with hierarchical clustering combined with a heatmap. The performance of both the projection and prediction models was evaluated based on the RMSEs of the predicted RTI *vs.* experimental RTI. The statistical comparison of the RMSE values was performed using the *F*-test (*p* < 0.05, no correction for multiple comparisons was done). Data, code, and additional visualizations are available in the ESI.†

## 3. Results and discussion

### 3.1 Comparability of the RT, RTI, and RTO across CSs

The same calibration and suspect compound mix was analyzed with all CSs; however, the number of detected calibrants ranged from 12 to 38 and suspects from 17 to 40. The earliest eluting suspect had RT between 0.8 and 7.3 min, depending on the CS. Simultaneously, the RT of the last eluting suspect varied between 8.1 min and 32.4 min. These differences arise from the substantially different lengths of gradient programs used (15 to 54 min), including segments of isocratic elution implemented at different parts of the elution program.

The similarity in the retention time profiles of CSs was evaluated based on the correlation of the RTs with Pearson correlation coefficients and RTOs with Spearman correlation coefficients. Generally, the agreement in RTs for two CSs (Pearson correlation) followed the same order as that based on RTOs (Spearman correlation), with a vast majority of the CS pairs showing *R*^2^ > 0.8. This is expected as both the correlation of RT and RTO values have their advantages and disadvantages. RTOs are less affected by segments of isocratic elution, while RTs are less affected by small differences in the retention time for close eluting or overlapping chemicals. Due to the good agreement of the Pearson and Spearman correlation coefficients (ESI 1†), only the RTO agreement (Spearman correlation) is discussed below.

The Spearman correlation coefficient of RTO values ranged from 0.59 to 0.999 (ESI 1†). Independent of the type of correlation coefficient, the highest similarity was observed for the two pairs of CSs where both CSs were implemented in the same lab, by making use of either a different LC/HRMS instrument (DS_QDF and DS_VQL) or the HRMS mode only (DS_GSB and DS_TDF). The latter is expected to yield different RTs only due to the random variability of the analysis and data processing.

The highest correlation coefficients for CSs implemented in different laboratories (DS_HT and DS_QBD) exceeded a Spearman *ρ* value of 0.999. These CSs used the same LC column (Agilent ZORBAX Eclipse Plus C18) and mobile phase (water and acetonitrile with 0.1% formic acid). Nevertheless, the CSs differed in LC instrumentation, gradient program, column temperature, injection volume, *etc*. This indicates that the retention order may be well preserved, given the same column chemistry and mobile phase, even if other parameters vary.

The highest Spearman ranked correlation coefficient for CSs (DS_BQW and DS_MT) using different columns from different vendors yielded only a marginally lower Spearman *ρ* value of 0.998; however, both CSs made use of a very similar mobile phase containing ammonium formate in both mobile phase components. This indicates a good agreement in RTOs despite having different CSs. However, some pairs of CSs leveraging the same retention mechanism yielded Spearman *ρ* values below 0.9.

Further analysis with hierarchical clustering revealed several clusters of CSs with similar elution profiles that could be associated with the CS parameters. Firstly, one of the CSs showed substantially different RTOs in comparison with all other CSs. A closer investigation revealed that this was the only CS that combined RP and HILIC columns for separation (DS_DX), and the significant differences in the RTI order clearly indicate the orthogonality of this system. Due to the significant differences in the retention mechanism of RP and HILIC, only RP based CSs will be considered further in evaluating the projection or prediction method as both approaches assume the same retention mechanism.

**Fig. 1:**
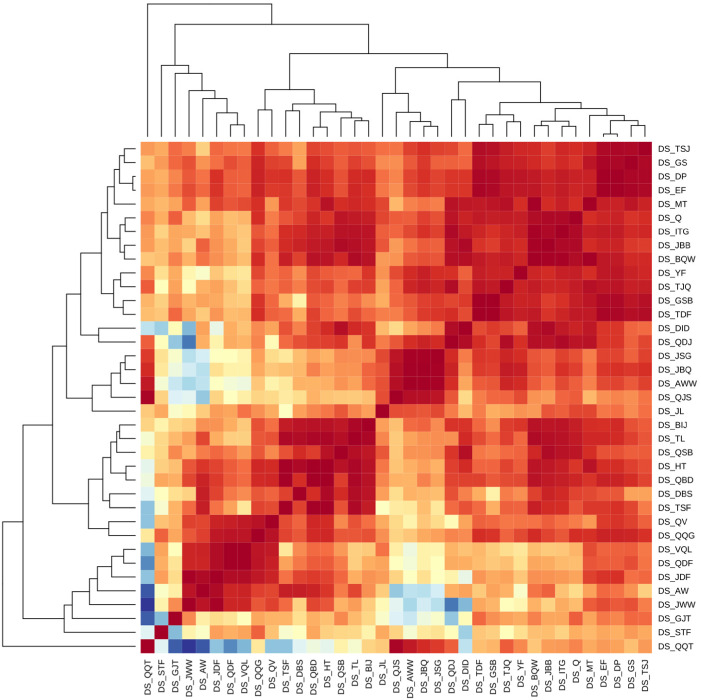
The heatmap of the CSs based on the Spearman correlation coefficient of the RTOs. Red indicates high and blue indicates low Spearman correlation coefficients.

Among the CS using RP-LC, a cluster of five CSs, namely DS_JL, DS_QJS, DS_AWW, DS_JBQ, and DS_JSG, stands out. Furthermore, RTOs from DS_QQT showed a high correlation with the RTOs from the above CSs (Fig. 1). All these CSs made use of ammonium salt as a mobile phase additive either alone or with formic acid. As the majority of the CSs used in this interlaboratory comparison made use of 0.1% formic acid as a water phase additive, the CSs in this cluster use a higher pH of the water phase compared to the majority of the CSs. Comparison of the RTOs from these CSs with RTOs from CSs using formic acid as a mobile phase additive revealed that higher RTOs were exclusively observed for chemicals with basic functional groups (p*K*_a_ values between 3.6 and 8.1) with ammonium formate as a mobile phase additive. Similarly, lower retention time order values were observed for acidic chemicals, suggesting the importance of the acid–base equilibrium of the analytes in the RTO. In addition to the above-mentioned CSs, three additional systems leveraged ammonium salts as mobile phase additives, namely DS_STF, DS_GJT and DS_QSB. The RTOs from DS_STF and DS_GJT showed lower similarity to all other CSs, possibly due to the use of a different stationary phase. DS_STF used a biphenyl column, while DS_GJT used a C_8_ column. On the other hand, DS_QSB clustered together with CSs facilitating formic acid as a water phase additive.

In two of the clusters of CSs (clusters of DS_JWW, DS_AW, DS_JDF, DS_QDF, DS_VQL, DS_QQG, DS_ QV; and cluster of DS_BIJ, DS_TL, DS_QSB, DS_HT, DS_QBD, DS_DBS, and DS_TSF) leveraged exclusively 0.1% formic acid as the water phase additive. Several CSs in the first of these clusters used Agilent Eclipse Plus or Poroshell columns, while in the second Phenomenex Kinetex EVO columns were used in several CSs. This is likely to indicate that in addition to the mobile phase the stationary phase chemistry has an impact on the agreement of the RTOs even if nominally all CSs considered here applied RP separation.

Importantly, the similarity in RTOs is impacted by the chemicals commonly detected by the two CSs. For example, if one of the LC/HRMS methods has not detected some of the acidic or basic compounds that are likely to change RTOs, such an effect will be left undetected here. Nevertheless, the cluster analysis demonstrates that the RTOs largely depend on the similarity of the CSs and the impact on the performance of projection and prediction methods needs close scrutiny.

### 3.2 Peak spacing

Ideal chromatographic systems would allow the separation of all components in the mixture. In NTS, this is virtually impossible due to the high complexity of the environmental samples as well as the limited time available for analyses. For a limited and known number of chemicals, ideal peak spacing would follow a uniform distribution where chemicals elute across the whole elution program and peaks are equally spaced. Under such conditions the peak overlap as well as the resulting matrix effect would be minimized. In the case of NTS, the peak spacing also affects the purity of MS^2^ spectra, especially for data independent acquisition. Last but not least, the distribution can affect RT based prioritization, as candidate structures predicted to elute in regions of high peak density are harder to distinguish from each other.

**Fig. 2:**
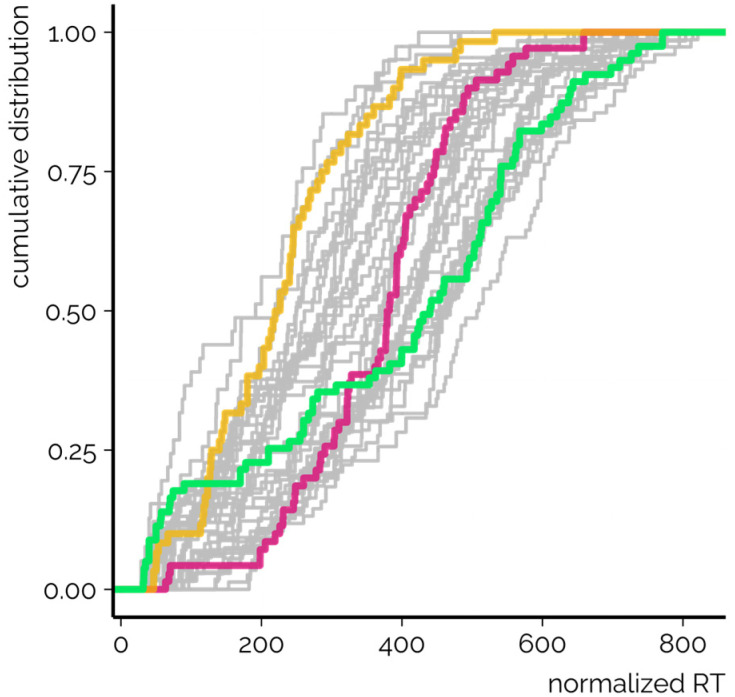
The cumulative distribution of the normalized RTs for all CSs. In green, the CS with the highest standard deviation of the normalized RTs is shown. In contrast, two CSs yielding relatively narrower peak spacing are highlighted in yellow and red. Both calibrants and suspects are considered.

In order to evaluate the peak spacing of CSs used here, RTs were normalized relative to the length of the elution program. The peak spacing was analyzed based on the standard deviation and cumulative distribution of the normalized RTs. It was observed that in some CSs the chemicals eluted almost equally spaced across the whole elution program (Fig. 2, green line). The highest standard deviation of the normalized and non-normalized RTs was observed for CS DS_TSJ, which made use of a relatively long elution program and the duration of the gradient segment exceeded 50% of the total elution program (run time).

Simultaneously, with some CSs a narrow range of normalized RTs was observed. For example, for CS DS_QV (Fig. 2, red line) most of the chemicals yielded RTI values between 200 and 600. This CS has long isocratic elution segments at the beginning and end of the elution program while the gradient segment is relatively short in comparison with the length of the elution program (<40%). The absolute peak spacing based on the distribution of the normalized RTs showed that CS DS_QV yielded one of the lowest RT standard deviations. The distribution of the normalized RTs is therefore also affected by the polarity of the chemicals included in the study. For example, it is fair to believe that incorporating more (very) low and high polarity chemicals would benefit this CS due to the isocratic elution segments. Nevertheless, the chemicals used in this study are chosen as representatives of the chemical contaminants in environmental water samples.

Furthermore, for CS DS_TSF (Fig. 2, yellow line) and many others, the majority of the normalized RTs remained below 400, hinting at a very tight spacing of the peaks. Simultaneously, for CS DS_MT the detailed analysis revealed that the narrow range of normalized RTs is also associated with the low number of detected chemicals. Namely, most of the late eluting chemicals incorporated in this study were undetected with the CS DS_MT, which impacted the analysis of the normalized RTs. The same was true for other CSs yielding the lowest maximum normalized RTs. Nevertheless, no direct association between the standard deviation of the normalized RTs and the number of detected chemicals was observed.

As a result, the analysis of peak spacing, retention time distribution and the peak detectability suggests that longer elution programs with proportionally larger segments of linear gradients provide a more uniform peak distribution. Importantly, this coincides with the insights from Anderson *et al.*,^38^ who found that longer gradient programs increase the number of chemical features detected with LC/HRMS based NTS. It can be simultaneously hypothesized that such elution programs also improve the identification of the detected chemicals based on the RTs due to the expected narrower relative uncertainty.

### 3.3 Projection of RTI

The RMSE of the RTIs of the suspects with the projection approach was 78.1 RTI units over all labs, that is, 7.8% of the total RTI range for the calibrants (Table S1, ESI 2, ESI/results†). The mean absolute deviation (MAD) was 46.5 RTI units and 95% of the absolute deviations were less than 175.2 RTI units. The projection approach facilitating the GAM clearly improved the transferability of the RTIs across CSs. The RMSE of the RTIs of the suspects prior to the GAM was 150.4 RTI units across all combinations of the CSs and in 92.3% of the CS combinations a lower RMSE point estimate was observed after applying the GAM. In 57.7% of the cases the improvement was statistically significant. In 7.7% of the CS combinations (*n* = 102) the RMSE point estimate was higher after projection; however, in only four of the cases the difference in RMSE was statistically significant according to the *F*-test. These findings highlight the need for an RT/RTI projection approach even if commonly analyzed calibrants are used for obtaining the RTIs.

**Fig. 3:**
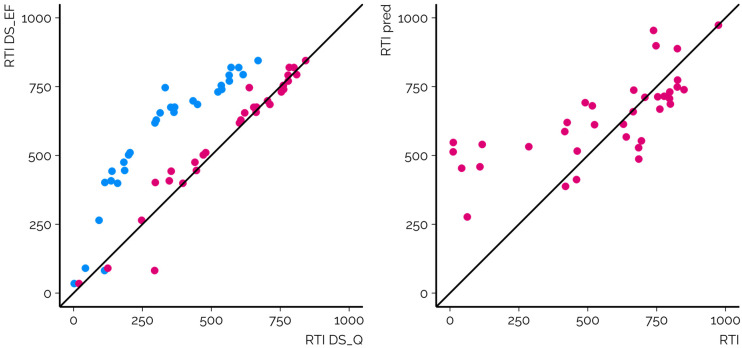
The comparison of (left) the projection of RTI values for suspects analyzed with two different CSs before (blue, RMSE of 317.4 RTI units) and after (red, RMSE of 53.4 RTI units) application of the GAM (DS_Q → DS_EF) and (right) prediction of the RTI values with the ML model for DS_EF (RMSE of 167.7 RTI units).

The necessity for the GAM for RTI projection results from the fact that RTI calculation, as proposed earlier,^6^ makes use of the first and last eluting calibration chemicals and is unaffected by the rest of the calibrants and their RT profiles. Thus, the RTI values are a linear projection of the RTs and overlook any nonlinearity arising from the different gradient profiles, including isocratic segments, used by different CSs. For example, the two CSs exemplified in Fig. 3 used BEH C18 columns and methanol as an organic modifier; however, they differed in the flow rate and organic modifier gradient as well as the position and length of the isocratic elution segment. As a result, the retention order of the chemicals and Spearman correlation of the RTIs from the two CSs are high. Nevertheless, the RTI values are nonlinearly associated, leading to an RMSE of 257.2 RTI units without the GAM. Here the GAM enables an effective projection of the RTIs from one system to another, and the RMSE drops to 55.0 RTI units.

We furthermore observed that the projection of the GAM was very sensitive to the overlap in the calibration chemicals detected by the two CSs. Most of all, the projections were unreliable for suspects outside of the calibration range (lower or higher RTs of suspects than those of calibrants), and all such suspects were omitted in the above analysis. As a result, the projection results are unavailable for 9.8% of the detected suspects across all combinations of CSs. In this sense, it is important to ensure that the calibrants elute across the eluent program.

Lastly, we were also interested in the projection accuracy of the calibrants that can be used to assess the prediction accuracy of the suspects. We therefore compared the RMSE values of the calibrants and suspects for each combination of the CSs. Ideally, the RMSE values would be statistically insignificantly different. Here we observed a weak correlation of the RMSE values of the calibrants and suspects (Pearson's squared correlation coefficient of 0.30). In 63 cases, the RMSE of the suspects was statistically significantly lower than that of the calibrants with a maximum difference of 4.7×. The RMSE was statistically significantly higher for suspects with 535 of the 1332 combinations of CSs and the maximum relative increase in the RMSE was over two orders of magnitude. As a consequence, the RMSE values of the calibrants are insufficient in evaluating the projection accuracy for the suspects for a large fraction of the combinations of CSs. As a result, we suggest evaluating the projection accuracy with a separate set of quality control chemicals that are representative of the chemicals expected to be detected in the samples but are not used for fitting the GAM. This will allow for an independent RMSE evaluation, which can be further used as the uncertainty of the projection approach. For example, given the chemical similarity between the quality controlled chemicals and suspected chemicals, ∼95% of the “true” RTs of the suspected chemicals are expected to be within ±2 RMSE from the RT obtained with the GAM.

### 3.4 Prediction models of RTI

In the case of NTS of complex mixtures, the RTs or RTIs of suspects may be unavailable in public libraries due to, *e.g.*, the lack of analytical standards. This has led to the prediction of the RT(I)s with different ML models.^6,32,39^ The models are often evaluated on chemicals measured on the same CS, which therefore leads to potentially optimistic performance evaluations. The dataset from the interlaboratory comparison provides a great opportunity to evaluate the consistency of the model performance on CSs used in NTS. For this reason, we trained an RT prediction model based on the cleaned and standardized RT data previously published by Aalizadeh *et al.*^6^ The retraining of the model was needed, to provide unbiased evaluation so that the training set did not contain any of the calibration chemicals or suspects. The calibration chemicals were used to project the predicted RTs to the RTI scale of the respective CSs.^6^

**Fig. 4:**
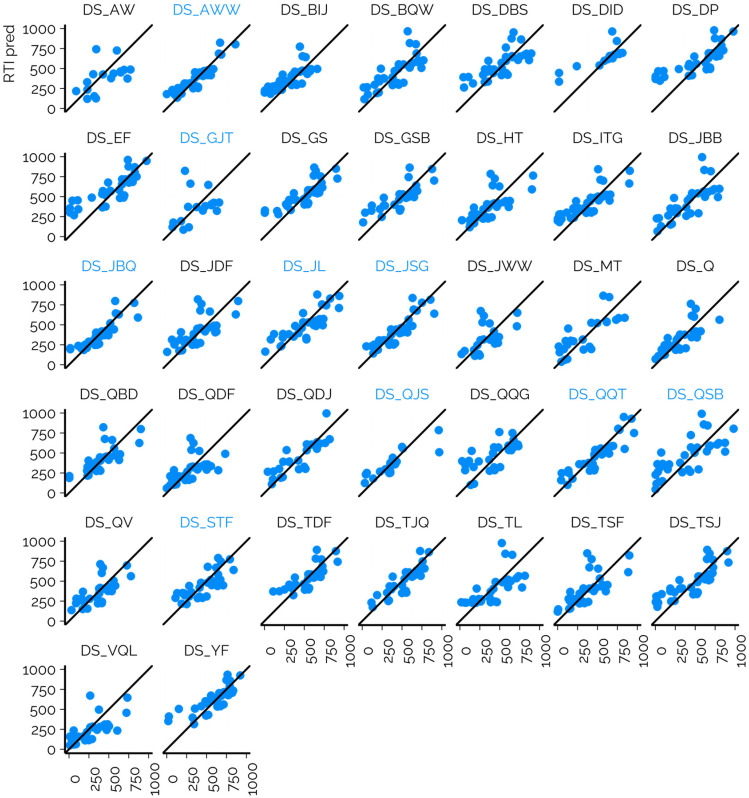
The comparison of the predicted RTI values with ML *vs.* the experimentally observed RTI values of suspects. DS_AWW, DS_GJT, DS_JBQ, DS_JL, DS_JSG, DS_QJS, DS_QQT, DS_QSB, and DS_STF (marked in blue) all leveraged the ammonium-based buffer in the mobile phase, while the other leveraged the mobile phase with formic acid as an additive.

The RMSE of suspects across all CSs was 149.0 RTI units, while the overall MAD was 150.3 RTI units and 95% of the chemicals yielded RTI values below 289.8 units across all CSs (Table S1†). The *R*^2^ values showed the same trends as RMSE values; generally CSs with lower RMSE values showed higher *R*^2^ values of RTI predicted and RTI measured. The lowest *R*^2^ value was 0.12 (DS_GJT) and the highest value was 0.82 (DS_AWW). The performance of the prediction models varied starkly depending on the CS (Fig. 4, ESI 3, ESI/results/NORMAN_RTI_prediction_results_summary.csv†): the RMSE values for the individual CSs ranged from 86.8 to 219.7 RTI units. It must be recognized that the RMSE values of suspects are influenced by (1) the suitability of the training data for the CSs and chemical space in question; (2) the accuracy of the model; and (3) the accuracy of the projection of the predicted RTs to the RTIs of the CS. The RMSE values of the calibrants are primarily impacted by the former two factors and less by the projection accuracy as the experimental RTIs are directly used for fitting the projection model.

We observed that the performance in terms of RMSE was directly correlated with the similarity of the CS_NTS_ (CS used for measuring the suspects) and CS_training_ (used for collecting the training data). All four CS_NTS_ on which the trained models showed the lowest RMSEs for the suspects used an ammonium formate based mobile phase. Similarly, the CS_training_ used a mobile phase containing ammonium formate; however, different columns and mobile phase flow rates were used. Furthermore, for these CSs relatively low RMSEs of the calibrants were observed, also indicating a successful projection of the predicted RTs to experimental RTIs. This is in accordance with the RTO correlations observed above, where a good agreement of the RTOs for ammonium salt containing mobile phases was observed independent of the column. As a result, it is expected that predicted RTIs can be successfully used on CSs using similar mobile phases.

Generally, a weak agreement in RMSE values of the calibrants and suspects was observed, where CSs yielding relatively low values for one set also yielded low values for the other and *vice versa* (Pearson's squared correlation coefficient of 0.47). Nevertheless, in a handful of cases the RMSE values of the calibrants were low, while relatively high RMSE values were observed for suspects. No concrete reasons for the substantially higher RMSEs of suspects could be pinpointed either in terms of chemicals detected or the settings of CSs. Nevertheless, the high RMSEs of the calibrants were usually indicative of high RMSEs of the suspects and could be considered in the interpretation of the results.

Recent findings by Kwon *et al.*^36^ demonstrated that RT models directly trained on a small dataset might outperform ML models combined with projection models; however, the reasons remained unclear. Inspired by these findings, we trained separate RTI prediction models for each CS using calibration chemicals only (ESI 4†). The RMSE values ranged from 131.5 to 351.3 RTI units and the performance of the models clearly correlated with the number of training instances, where the correlation coefficient between the RMSE and number of training datapoints was −0.63. The model trained on the calibration data from the same CS never outperformed the ML model trained on a larger dataset followed by projection. On the other hand, the ML models trained on external large datasets outperformed the models trained on the calibrant data from the same CS in 11 cases (*F*-test, *p* < 0.05). In the remaining cases, the performance of the two models was statistically insignificantly different. This indicates that given sufficient overlap in the chemical space, ML models trained on larger datasets followed by the projection of the predicted RTIs may be preferred to in-house trained models if only limited training data are available.

All in all, the application of ML predicted retention times in the data from 37 CSs indicates that the reliability of the predictions is closely linked to the similarity in the CSs used for collecting the training data and the CS used in NTS. This highlights the need for using CSs in NTS that are similar to community standards if the applicability of ML models for structural elucidation or other tasks is desired.

### 3.5 Comparison of the projection and prediction models

It can be hypothezised that prediction models combined with projection models are less accurate than the projection of experimental RT/RTI values, due to error propagation. Indeed, the RMSE values for the prediction model (larger dataset followed by projection, sections 2.3 and 3.4) are mostly higher than those observed for the projection model (sections 2.2 and 3.3); however, specific combinations of CSs may yield higher RMSE values for the projection method than those for the prediction method (ESI 5†). In total 348 (26.1%) of 1332 combinations of CSs yielded statistically insignificantly different RMSE values with projection and prediction methods, considering only chemicals for which the RTI could be predicted with both. For 32 (2.4%) of the combinations of CSs, the prediction models outperformed projection models, and for the remaining 71.5% of the cases, the projection models yielded statistically significantly lower RMSE values. The comparison is impacted by the accuracy of both the prediction and projection methods.

The prediction models outcompeted the projection models in seven CSs (DS_AWW, DS_DID, DS_JBQ, DS_JSG, DS_QJS, DS_QQT, and DS_YF). In these cases, with the exception of two CSs (DS_DID and DS_YF), the CS_NTS_ mobile phase is similar to the mobile phase of CS_training_ (ammonium salt based buffer solution); therefore, the ML model for RTI prediction has been trained on data with a chromatographically similar mechanism. These five CSs also showed the lowest (top6) RMSE values for the prediction approach.

Furthermore, the comparison of projection and prediction methods is also impacted by the CS_source_ used in the projection approach. Here, most cases where the prediction method outcompeted the projection method are characterized by mismatching the mobile phase of the CS_source_ and CS_NTS_ and therefore high RMSE_projection_ values of the suspects (between 103.7 and 207.7 RTI units). Nevertheless, for the CS_source_ and CS_NTS_ using a similar mobile phase, the projection method outperformed the prediction approach even if the CS_training_ closely matched the CS used in NTS.

This indicates that in the case where the CS used in NTS closely matches the one with experimentally available RTI values, the projection method should be preferred. However, if experimental data with similar CSs are lacking, and the ML model used in the prediction approach is trained on data from a similar CS, the prediction approach could yield similar or higher prediction accuracy than the projection approach from a less similar CS. Moreover, the prediction approach is applicable to all chemicals within the scope of the ML, even the ones that lack previously measured chromatographic data.

## Conclusions

A recent interlaboratory study using the NORMAN network has provided a unique opportunity to evaluate the comparability of RT, RTI, and RTOs across 37 CSs used in laboratories in NTS with RP LC/HRMS on a routine basis. Due to an overlapping set of calibration chemicals and suspects analyzed with all CSs, the impact of CSs could be directly evaluated. Expectedly, the similarity of CSs was found to significantly impact the RTIs and RTOs, where the most influential factors were the additive type and/or pH of the water phase. Additionally, the clustering of RTOs from similar column chemistry was observed.

Furthermore, two commonly applied approaches for evaluating RT(I)s for candidate structures, the projection and prediction approaches, could be compared. As expected from the RTO correlations, the performance of both approaches was affected by the similarity in the mobile phase composition. Thus, projection methods generally outperformed prediction models; however, prediction models were found to occasionally outperform the projection method if the prediction model was trained on similar CSs. Furthermore, the prediction models trained on larger external data should be preferred over small in-house data in spite of mismatching CSs. As a result, we suggest using projection methods when the CS_source_ closely matches with the CS_NTS_ and in other cases, prediction models trained on large representative datasets should be preferred due to their accuracy and application scope.

## Author contributions

L. M.: data curation, investigation, validation, and writing – review & editing. A. K.: conceptualization, formal analysis, funding acquisition, writing – original draft, and writing – review & editing.

## Conflicts of interest

There are no conflicts to declare.

## Supplementary Material

AN-150-D5AN00323G-s001

AN-150-D5AN00323G-s002

## Data Availability

The data supporting this article have been included as part of the ESI.†

## References

[cit1] Hollender J., Schymanski E. L., Ahrens L., Alygizakis N., Béen F., Bijlsma L., Brunner A. M., Celma A., Fildier A., Fu Q., Gago-Ferrero P., Gil-Solsona R., Haglund P., Hansen M., Kaserzon S., Kruve A., Lamoree M., Margoum C., Meijer J., Merel S., Rauert C., Rostkowski P., Samanipour S., Schulze B., Schulze T., Singh R. R., Slobodnik J., Steininger-Mairinger T., Thomaidis N. S., Togola A., Vorkamp K., Vulliet E., Zhu L., Krauss M. (2023). NORMAN Guidance on Suspect and Non-Target Screening in Environmental Monitoring. Environ. Sci. Eur..

[cit2] Schymanski E. L., Jeon J., Gulde R., Fenner K., Ruff M., Singer H. P., Hollender J. (2014). Identifying Small Molecules via High Resolution Mass Spectrometry: Communicating Confidence. Environ. Sci. Technol..

[cit3] Hupatz H., Rahu I., Wang W.-C., Peets P., Palm E. H., Kruve A. (2024). Critical Review on in Silico Methods for Structural Annotation of Chemicals Detected with LC/HRMS Non-Targeted Screening. Anal. Bioanal. Chem..

[cit4] Metz T. O., Chang C. H., Gautam V., Anjum A., Tian S., Wang F., Colby S. M., Nunez J. R., Blumer M. R., Edison A. S., Fiehn O., Jones D. P., Li S., Morgan E. T., Patti G. J., Ross D. H., Shapiro M. R., Williams A. J., Wishart D. S. (2025). Introducing “Identification Probability” for Automated and Transferable Assessment of Metabolite Identification Confidence in Metabolomics and Related Studies. Anal. Chem..

[cit5] Stanstrup J., Neumann S., Vrhovšek U. (2015). PredRet: Prediction of Retention Time by Direct Mapping between Multiple Chromatographic Systems. Anal. Chem..

[cit6] Aalizadeh R., Alygizakis N. A., Schymanski E. L., Krauss M., Schulze T., Ibáñez M., McEachran A. D., Chao A., Williams A. J., Gago-Ferrero P., Covaci A., Moschet C., Young T. M., Hollender J., Slobodnik J., Thomaidis N. S. (2021). Development and Application of Liquid Chromatographic Retention Time Indices in HRMS-Based Suspect and Nontarget Screening. Anal. Chem..

[cit7] Kretschmer F. , Harrieder E.-M., Witting M. and Böcker S., Times Are Changing but Order Matters: Transferable Prediction of Small Molecule Liquid Chromatography Retention Times, December 23, 2024. 10.26434/chemrxiv-2024-wd5j8

[cit8] Domingo-Almenara X., Guijas C., Billings E., Montenegro-Burke J. R., Uritboonthai W., Aisporna A. E., Chen E., Benton H. P., Siuzdak G. (2019). The METLIN Small Molecule Dataset for Machine Learning-Based Retention Time Prediction. Nat. Commun..

[cit9] Celma A., Bade R., Sancho J. V., Hernandez F., Humphries M., Bijlsma L. (2022). Prediction of Retention Time and Collision Cross Section (CCS_H+_, CCS_H–_, and CCS_Na+_) of Emerging Contaminants Using Multiple Adaptive Regression Splines. J. Chem. Inf. Model..

[cit10] Zhou Z., Tu J., Xiong X., Shen X., Zhu Z.-J. (2017). LipidCCS: Prediction of Collision Cross-Section Values for Lipids with High Precision To Support Ion Mobility–Mass Spectrometry-Based Lipidomics. Anal. Chem..

[cit11] Ross D. H., Cho J. H., Xu L. (2020). Breaking Down Structural Diversity for Comprehensive Prediction of Ion-Neutral Collision Cross Sections. Anal. Chem..

[cit12] Broeckling C. D., Ganna A., Layer M., Brown K., Sutton B., Ingelsson E., Peers G., Prenni J. E. (2016). Enabling Efficient and Confident Annotation of LC-MS Metabolomics Data through MS1 Spectrum and Time Prediction. Anal. Chem..

[cit13] Costalunga R., Tshepelevitsh S., Sepman H., Kull M., Kruve A. (2021). Sodium Adduct Formation with Graph-Based Machine Learning Can Aid Structural Elucidation in Non-Targeted LC/ESI/HRMS. Anal. Chim. Acta.

[cit14] Akhlaqi M., Wang W.-C., Möckel C., Kruve A. (2023). Complementary Methods for Structural Assignment of Isomeric Candidate Structures in Non-Target Liquid Chromatography Ion Mobility High-Resolution Mass Spectrometric Analysis. Anal. Bioanal. Chem..

[cit15] Bijlsma L., Berntssen M. H. G., Merel S. (2019). A Refined Nontarget Workflow for the Investigation of Metabolites through the Prioritization by in Silico Prediction Tools. Anal. Chem..

[cit16] Kretschmer F., Harrieder E.-M., Hoffmann M. A., Böcker S., Witting M. (2024). RepoRT: A Comprehensive Repository for Small Molecule Retention Times. Nat. Methods.

[cit17] Zhang Y., Liu F., Li X. Q., Gao Y., Li K. C., Zhang Q. H. (2024). Retention Time Dataset for Heterogeneous Molecules in Reversed–Phase Liquid Chromatography. Sci. Data.

[cit18] Boelrijk J., Van Herwerden D., Ensing B., Forré P., Samanipour S. (2023). Predicting RP-LC Retention Indices of Structurally Unknown Chemicals from Mass Spectrometry Data. J. Cheminf..

[cit19] Stoffel R., Quilliam M. A., Hardt N., Fridstrom A., Witting M. (2022). N-Alkylpyridinium Sulfonates for Retention Time Indexing in Reversed-Phase-Liquid Chromatography-Mass Spectrometry–Based Metabolomics. Anal. Bioanal. Chem..

[cit20] Yang P., McCabe T., Pursch M. (2011). Practical Comparison of LC Columns Packed with Different Superficially Porous Particles for the Separation of Small Molecules and Medium Size Natural Products. J. Sep. Sci..

[cit21] Rosés M. (2004). Determination of the pH of Binary Mobile Phases for Reversed-Phase Liquid Chromatography. J. Chromatogr. A.

[cit22] Ngan H. L. , Turkina V., Van Herwerden D., Yan H., Cai Z. and Samanipour S., Machine Learning for Enhanced Identification in RPLC/HRMS Non-Targeted Workflows, January 29, 2025. 10.26434/chemrxiv-2024-mdl4q-v2 PMC12392258 40791078

[cit23] Mollerup C. B., Mardal M., Dalsgaard P. W., Linnet K., Barron L. P. (2018). Prediction of Collision Cross Section and Retention Time for Broad Scope Screening in Gradient Reversed-Phase Liquid Chromatography-Ion Mobility-High Resolution Accurate Mass Spectrometry. J. Chromatogr. A.

[cit24] Zhang Y., Liu F., Li X. Q., Gao Y., Li K. C., Zhang Q. H. (2024). Generic and Accurate Prediction of Retention Times in Liquid Chromatography by Post–Projection Calibration. Commun. Chem..

[cit25] Kajtazi A., Kajtazi M., Santos Barbetta M. F., Bandini E., Eghbali H., Lynen F. (2025). Prediction of Retention Indices in LC-HRMS for Enhanced Structural Identification of Organic Micropollutants in Water: Selectivity-Based Filtration. Anal. Chem..

[cit26] Wen Y., Talebi M., Amos R. I. J., Szucs R., Dolan J. W., Pohl C. A., Haddad P. R. (2018). Retention Prediction in Reversed Phase High Performance Liquid Chromatography Using Quantitative Structure-Retention Relationships Applied to the Hydrophobic Subtraction Model. J. Chromatogr. A.

[cit27] Rutan S. C., Kempen T., Dahlseid T., Kruger Z., Pirok B., Shackman J. G., Zhou Y., Wang Q., Stoll D. R. (2024). Improved Hydrophobic Subtraction Model of Reversed-Phase Liquid Chromatography Selectivity Based on a Large Dataset with a Focus on Isomer Selectivity. J. Chromatogr. A.

[cit28] Liu Y., Yoshizawa A. C., Ling Y., Okuda S. (2024). Insights into Predicting Small Molecule Retention Times in Liquid Chromatography Using Deep Learning. J. Cheminf..

[cit29] Bach E., Schymanski E. L., Rousu J. (2022). Joint Structural Annotation of Small Molecules Using Liquid Chromatography Retention Order and Tandem Mass Spectrometry Data. Nat. Mach. Intell..

[cit30] Yang Q., Ji H., Fan X., Zhang Z., Lu H. (2021). Retention Time Prediction in Hydrophilic Interaction Liquid Chromatography with Graph Neural Network and Transfer Learning. J. Chromatogr. A.

[cit31] Ju R., Liu X., Zheng F., Lu X., Xu G., Lin X. (2021). Deep Neural Network Pretrained by Weighted Autoencoders and Transfer Learning for Retention Time Prediction of Small Molecules. Anal. Chem..

[cit32] Bonini P., Kind T., Tsugawa H., Barupal D. K., Fiehn O. (2020). Retip: Retention Time Prediction for Compound Annotation in Untargeted Metabolomics. Anal. Chem..

[cit33] Szucs R., Brown R., Brunelli C., Hradski J., Masár M. (2023). Impact of Structural Similarity on the Accuracy of Retention Time Prediction. J. Chromatogr. A.

[cit34] Bouwmeester R., Martens L., Degroeve S. (2020). Generalized Calibration Across Liquid Chromatography Setups for Generic Prediction of Small-Molecule Retention Times. Anal. Chem..

[cit35] Souihi A., Mohai M. P., Palm E., Malm L., Kruve A. (2022). MultiConditionRT: Predicting Liquid Chromatography Retention Time for Emerging Contaminants for a Wide Range of Eluent Compositions and Stationary Phases. J. Chromatogr. A.

[cit36] Kwon Y., Kwon H., Han J., Kang M., Kim J.-Y., Shin D., Choi Y.-S., Kang S. (2023). Retention Time Prediction through Learning from a Small Training Data Set with a Pretrained Graph Neural Network. Anal. Chem..

[cit37] Malm L., Liigand J., Aalizadeh R., Alygizakis N., Ng K., Frøkjær E. E., Nanusha M. Y., Hansen M., Plassmann M., Bieber S., Letzel T., Balest L., Abis P. P., Mazzetti M., Kasprzyk-Hordern B., Ceolotto N., Kumari S., Hann S., Kochmann S., Steininger-Mairinger T., Soulier C., Mascolo G., Murgolo S., Garcia-Vara M., López De Alda M., Hollender J., Arturi K., Coppola G., Peruzzo M., Joerss H., Van Der Neut-Marchand C., Pieke E. N., Gago-Ferrero P., Gil-Solsona R., Licul-Kucera V., Roscioli C., Valsecchi S., Luckute A., Christensen J. H., Tisler S., Vughs D., Meekel N., Talavera Andújar B., Aurich D., Schymanski E. L., Frigerio G., Macherius A., Kunkel U., Bader T., Rostkowski P., Gundersen H., Valdecanas B., Davis W. C., Schulze B., Kaserzon S., Pijnappels M., Esperanza M., Fildier A., Vulliet E., Wiest L., Covaci A., Macan Schönleben A., Belova L., Celma A., Bijlsma L., Caupos E., Mebold E., Le Roux J., Troia E., De Rijke E., Helmus R., Leroy G., Haelewyck N., Chrastina D., Verwoert M., Thomaidis N. S., Kruve A. (2024). Quantification Approaches in Non-Target LC/ESI/HRMS Analysis: An Interlaboratory Comparison. Anal. Chem..

[cit38] Anderson B. G., Raskind A., Habra H., Kennedy R. T., Evans C. R. (2021). Modifying Chromatography Conditions for Improved Unknown Feature Identification in Untargeted Metabolomics. Anal. Chem..

[cit39] Xue J., Wang B., Ji H., Li W. (2024). RT-Transformer: Retention Time Prediction for Metabolite Annotation to Assist in Metabolite Identification. Bioinformatics.

