# Supplementary material for: Do experimental projection methods outcompete retention time prediction models in non-target screening? A case study on LC/HRMS interlaboratory comparison data
Source: Analyst. 2025 Jul 8;150(16):3567–77. doi: 10.1039/d5an00323g (PMC12268317; doi:10.1039/d5an00323g)
Supplement: AN-150-D5AN00323G-s001 [file AN-150-D5AN00323G-s001.zip › SI/SI1_data_visualization.html]

NORMAN interlab RT data visualization


# NORMAN interlab RT data visualization

#### Anneli Kruve

#### 2025-02-14

## Libraries

## Data

Experimental RT data from the interlaboratory comparison.

```
data = read_delim("data/NORMAN_interlab_RT_data.csv")
data_xy_pred = read_delim("results/NORMAN_RTI_projection_results.csv")
gradient_lenght = read_delim("data/gradient_lenght_codes.csv")
```

## Numerical data overview

```
data %>%
  group_by(CS_code, sample_type) %>%
  summarize(count = n()) %>%
  ungroup() %>%
  arrange(count)
```

```
## `summarise()` has grouped output by 'CS_code'. You can override using the
## `.groups` argument.
```

```
## # A tibble: 74 × 3
##    CS_code sample_type count
##    <chr>   <chr>       <int>
##  1 DS_QDJ  cal            12
##  2 DS_MT   cal            15
##  3 DS_DID  sus            17
##  4 DS_AW   cal            18
##  5 DS_QQG  cal            18
##  6 DS_QJS  cal            20
##  7 DS_DID  cal            22
##  8 DS_JWW  cal            22
##  9 DS_GJT  cal            23
## 10 DS_GJT  sus            23
## # ℹ 64 more rows
```

```
data %>%
  group_by(CS_code, sample_type) %>%
  summarize(min = min(RT),
            max = max(RT)) %>%
  ungroup() %>%
  arrange(max)
```

```
## `summarise()` has grouped output by 'CS_code'. You can override using the
## `.groups` argument.
```

```
## # A tibble: 74 × 4
##    CS_code sample_type   min   max
##    <chr>   <chr>       <dbl> <dbl>
##  1 DS_Q    sus         0.946  8.08
##  2 DS_MT   sus         0.8    8.7 
##  3 DS_QDJ  sus         0.884  9.78
##  4 DS_Q    cal         1.03   9.79
##  5 DS_QQG  cal         1.86   9.96
##  6 DS_QQG  sus         1.02  10.1 
##  7 DS_MT   cal         0.870 10.2 
##  8 DS_YF   cal         0.855 11.0 
##  9 DS_YF   sus         1.05  11.1 
## 10 DS_GJT  sus         1.5   11.2 
## # ℹ 64 more rows
```

## Visual data overview

```
data_xy_pred %>%
  group_by(CS_x, CS_y) %>%
  summarize(R2 = cor(RTI_x, RTI_y, method = "pearson")^2) %>%
  ungroup() %>%
  filter(CS_x != CS_y) %>%
  arrange(R2)
```

```
## `summarise()` has grouped output by 'CS_x'. You can override using the
## `.groups` argument.
```

```
## # A tibble: 1,332 × 3
##    CS_x   CS_y      R2
##    <chr>  <chr>  <dbl>
##  1 DS_AW  DS_QJS 0.568
##  2 DS_QJS DS_AW  0.568
##  3 DS_AW  DS_QQT 0.571
##  4 DS_QQT DS_AW  0.571
##  5 DS_MT  DS_QJS 0.618
##  6 DS_QJS DS_MT  0.618
##  7 DS_QJS DS_QSB 0.626
##  8 DS_QSB DS_QJS 0.626
##  9 DS_GJT DS_QJS 0.631
## 10 DS_QJS DS_GJT 0.631
## # ℹ 1,322 more rows
```

```
data_xy_pred %>%
  group_by(CS_x, CS_y) %>%
  summarize(R2 = cor(RTI_x, RTI_y, method = "spearman")^2) %>%
  ungroup() %>%
  filter(CS_x != CS_y) %>%
  arrange(R2)
```

```
## `summarise()` has grouped output by 'CS_x'. You can override using the
## `.groups` argument.
```

```
## # A tibble: 1,332 × 3
##    CS_x   CS_y      R2
##    <chr>  <chr>  <dbl>
##  1 DS_JWW DS_QQT 0.590
##  2 DS_QQT DS_JWW 0.590
##  3 DS_AW  DS_QQT 0.614
##  4 DS_QQT DS_AW  0.614
##  5 DS_GJT DS_QQT 0.617
##  6 DS_QQT DS_GJT 0.617
##  7 DS_JWW DS_QDJ 0.634
##  8 DS_QDJ DS_JWW 0.634
##  9 DS_QDF DS_QQT 0.648
## 10 DS_QQT DS_QDF 0.648
## # ℹ 1,322 more rows
```

Agreement of the Pearson and Spearman correlation coefficients

```
pearson = data_xy_pred %>%
  group_by(CS_x, CS_y) %>%
  summarize(R2 = cor(RTI_x, RTI_y, method = "pearson")^2) %>%
  ungroup() %>%
  filter(CS_x != CS_y) %>%
  unite(combined, CS_x, CS_y) %>%
  rename(pearson = R2)
```

```
## `summarise()` has grouped output by 'CS_x'. You can override using the
## `.groups` argument.
```

```
spearman = data_xy_pred %>%
  group_by(CS_x, CS_y) %>%
  summarize(R2 = cor(RTI_x, RTI_y, method = "spearman")^2) %>%
  ungroup() %>%
  filter(CS_x != CS_y) %>%
  unite(combined, CS_x, CS_y) %>%
  rename(spearman = R2)
```

```
## `summarise()` has grouped output by 'CS_x'. You can override using the
## `.groups` argument.
```

```
pearson_spearman = pearson %>%
  left_join(spearman)
```

```
## Joining with `by = join_by(combined)`
```

```
ggplot(data = pearson_spearman) +
  geom_point(mapping = aes(x = pearson,
                           y = spearman),
             color = highlightercolor3) +
  xlab("Pearson R2") +
  ylab("Spearman R2") +
  my_theme
```

Calculate the similarity of the CSs based on the Spearman R2 of
RTIs.

```
data_xy_heatmap = data_xy_pred %>%
  group_by(CS_x, CS_y) %>%
  summarize(similarity = 1-cor(RTI_x, RTI_y, method = "spearman")^2) %>%
  ungroup()
```

```
## `summarise()` has grouped output by 'CS_x'. You can override using the
## `.groups` argument.
```

```
data_xy_heatmap %>%
  filter(CS_x != CS_y) %>%
  arrange(similarity)
```

```
## # A tibble: 1,332 × 3
##    CS_x   CS_y   similarity
##    <chr>  <chr>       <dbl>
##  1 DS_GSB DS_TDF   0.000384
##  2 DS_TDF DS_GSB   0.000384
##  3 DS_QDF DS_VQL   0.000518
##  4 DS_VQL DS_QDF   0.000518
##  5 DS_HT  DS_QBD   0.000820
##  6 DS_QBD DS_HT    0.000820
##  7 DS_DP  DS_EF    0.00151 
##  8 DS_EF  DS_DP    0.00151 
##  9 DS_QBD DS_TSF   0.00173 
## 10 DS_TSF DS_QBD   0.00173 
## # ℹ 1,322 more rows
```

```
data_xy_heatmap = data_xy_heatmap %>%
  spread(key = CS_y, value = similarity)

data_xy_heatmap = data_xy_heatmap %>%
  mutate_all(~replace_na(., 1))
```

Visualize the correlation as heatmap

```
heatmap(data_xy_heatmap %>%
            column_to_rownames(var = "CS_x") %>%
            as.matrix(),
          scale = "none",
          col = colorRampPalette(brewer.pal(11, "RdYlBu"))(256))
```

```
dev.off()
```

```
## null device 
##           1
```

## Peak Spacing

```
data = data %>%
  left_join(gradient_lenght)
```

```
## Joining with `by = join_by(CS_code)`
```

```
data = data %>%
  mutate(RT_scaled = RT / Lenghts * 1000)
```

Visualizing the RT\_scaled distribution

```
ggplot(data = data %>%
         mutate(CS_code = as.factor(CS_code)), 
       mapping = aes(x = CS_code, 
                     y = RT_scaled)) +
  geom_violin(fill = highlightercolor1) +
  labs(x = "CS", y = "normailized RT") +
  my_theme +
  theme(aspect.ratio = 0.25,
        axis.text.x = element_text(angle = 90))
```

Describe the data based on the RT\_scaled

```
data %>%
  group_by(CS_code) %>%
  summarize(mean = mean(RT_scaled), 
            sd = sd(RT_scaled),
            count = n(),
            max = max(RT_scaled)) %>%
  ungroup() %>%
  arrange(sd)
```

```
## # A tibble: 37 × 5
##    CS_code  mean    sd count   max
##    <chr>   <dbl> <dbl> <int> <dbl>
##  1 DS_QBD   226.  103.    64  483.
##  2 DS_QQG   253.  115.    50  480.
##  3 DS_TSF   228.  115.    60  532.
##  4 DS_MT    182.  116.    41  424.
##  5 DS_QDJ   214.  118.    39  470.
##  6 DS_QV    364.  120.    65  659.
##  7 DS_JWW   379.  121.    55  701.
##  8 DS_HT    301.  123.    67  636.
##  9 DS_GSB   390.  126.    54  644.
## 10 DS_TDF   385.  133.    61  645.
## # ℹ 27 more rows
```

Visualize in culative distribution graph.

```
ggplot(data = data, 
  mapping = aes(x = RT_scaled,
                color = CS_code)) +
  stat_ecdf(geom = "step") +
  scale_colour_manual(values = rep("grey", 41)) + 
  stat_ecdf(mapping = aes(x = RT_scaled),
            data = data %>% 
              filter(CS_code == "DS_QV"), 
            color = highlightercolor4,
            alpha = 0.75,
            size = 1,
            geom = "step") +
    stat_ecdf(mapping = aes(x = RT_scaled),
            data = data %>% 
              filter(CS_code == "DS_TSF"), 
            color = highlightercolor2,
            alpha = 0.75,
            size = 1,
            geom = "step") +
    stat_ecdf(mapping = aes(x = RT_scaled),
            data = data %>% 
              filter(CS_code == "DS_TSJ"), 
            color = highlightercolor1,
            size = 1,
            geom = "step") +
  labs(x = "normalized RT", y = "cumualtive distribution") +
  my_theme +
  theme(legend.position = "none")
```

```
## Warning: Using `size` aesthetic for lines was deprecated in ggplot2 3.4.0.
## ℹ Please use `linewidth` instead.
## This warning is displayed once every 8 hours.
## Call `lifecycle::last_lifecycle_warnings()` to see where this warning was
## generated.
```
