# Supplementary material for: Do experimental projection methods outcompete retention time prediction models in non-target screening? A case study on LC/HRMS interlaboratory comparison data
Source: Analyst. 2025 Jul 8;150(16):3567–77. doi: 10.1039/d5an00323g (PMC12268317; doi:10.1039/d5an00323g)
Supplement: AN-150-D5AN00323G-s001 [file AN-150-D5AN00323G-s001.zip › SI/SI2_projection_GAM.html]

RT projection between CS with GAM


# RT projection between CS with GAM

#### Anneli Kruve

#### 2025-02-14

## Libraries

## Data

Experimental RT data from the interlaboratory comparison.

```
data = read_delim("data/NORMAN_interlab_RT_data.csv")
```

## GAM

Data preprocessing for creating all-by-all combinations of CSs.

```
data_x = data %>%
  select(compound, SMILES, sample_type, CS_x = CS_code, RTx = RT)

data_y = data %>%
  select(compound, SMILES, sample_type, CS_y = CS_code, RTy = RT)

data_xy = data_x %>%
  left_join(data_y)
```

Performing GAM

```
data_xy_pred = tibble()
for(CS_this in levels(factor(data_xy$CS_x))) {
  print(CS_this)
  for(CS_that in levels(factor(data_xy$CS_y))) {
    data_xy_this_that = data_xy %>%
      filter(CS_x == CS_this & CS_y == CS_that) %>%
      select(CS_x, CS_y, compound, RTx, RTy, sample_type) %>%
      unique()
    
    data_xy_this_that = data_xy_this_that %>%
      bind_cols(data_xy_this_that %>%
                  filter(sample_type == "cal") %>%
                  mutate(RTx_min = min(RTx),
                         RTx_max = max(RTx),
                         RTy_min = min(RTy),
                         RTy_max = max(RTy)) %>%
                  select(RTx_min, RTx_max, RTy_min, RTy_max) %>%
                  unique()) %>%
      mutate(RTI_x = (RTx - RTx_min)/(RTx_max-RTx_min)*1000,
             RTI_y = (RTy - RTy_min)/(RTy_max-RTy_min)*1000)
    
    data_xy_this_that = data_xy_this_that %>%
      filter(0 <= RTI_x & RTI_x <= 1000 & 0 <= RTI_y & RTI_y <= 1000)
    
    model_this = gam(RTI_y ~ s(RTI_x, bs = "cr", k = 6),
                     data = data_xy_this_that %>%
                       filter(sample_type == "cal"))
    
    data_xy_this_that = data_xy_this_that %>%
      mutate(RTI_y_pred = predict.gam(model_this,
                                      newdata = data_xy_this_that))
    
    data_xy_pred = data_xy_pred %>%
      bind_rows(data_xy_this_that)
  }
  print(
    ggplot(data = data_xy_pred %>%
             filter(CS_x == CS_this & sample_type == "sus")) +
      geom_point(mapping = aes(x = RTI_x,
                               y = RTI_y),
                 color = highlightercolor6) +
      geom_point(mapping = aes(x = RTI_y_pred,
                               y = RTI_y),
                 color = highlightercolor4) +
      geom_abline(intercept = 0, slope = 1) +
      ylim(-400, 1200) +
      xlim(-400, 1200) +
      labs(x = "projection", y = "original") +
      facet_wrap(~CS_y) +
      my_theme +
      theme(axis.text.x = element_text(angle = 90))
  )
}
```

```
## [1] "DS_AW"
```

```
## [1] "DS_AWW"
```

```
## [1] "DS_BIJ"
```

```
## [1] "DS_BQW"
```

```
## [1] "DS_DBS"
```

```
## [1] "DS_DID"
```

```
## [1] "DS_DP"
```

```
## [1] "DS_EF"
```

```
## [1] "DS_GJT"
```

```
## [1] "DS_GS"
```

```
## [1] "DS_GSB"
```

```
## [1] "DS_HT"
```

```
## [1] "DS_ITG"
```

```
## [1] "DS_JBB"
```

```
## [1] "DS_JBQ"
```

```
## [1] "DS_JDF"
```

```
## [1] "DS_JL"
```

```
## [1] "DS_JSG"
```

```
## [1] "DS_JWW"
```

```
## [1] "DS_MT"
```

```
## [1] "DS_Q"
```

```
## [1] "DS_QBD"
```

```
## [1] "DS_QDF"
```

```
## [1] "DS_QDJ"
```

```
## [1] "DS_QJS"
```

```
## [1] "DS_QQG"
```

```
## [1] "DS_QQT"
```

```
## [1] "DS_QSB"
```

```
## [1] "DS_QV"
```

```
## [1] "DS_STF"
```

```
## [1] "DS_TDF"
```

```
## [1] "DS_TJQ"
```

```
## [1] "DS_TL"
```

```
## [1] "DS_TSF"
```

```
## [1] "DS_TSJ"
```

```
## [1] "DS_VQL"
```

```
## [1] "DS_YF"
```

## Statistical analysis

Calculating the statistics for RTI between source and target CS
without and with GAM.

```
data_xy_pred %>%
  filter(CS_x != CS_y) %>%
  group_by(sample_type) %>%
  summarize(RMSE_original = rmse(RTI_x, RTI_y),
            RMSE_gam = rmse(RTI_y_pred, RTI_y),
            MAD_original = mad(RTI_x, RTI_y),
            MAD_gam = mad(RTI_y_pred, RTI_y),
            Q95_original = quantile(abs(RTI_x - RTI_y), probs = c(0.95)),
            Q95_gam = quantile(abs(RTI_y_pred - RTI_y), probs = c(0.95)),
            R2_original = cor(RTI_x, RTI_y)^2,
            R2_gam = cor(RTI_y_pred, RTI_y)^2) %>%
  ungroup()
```

```
## # A tibble: 2 × 9
##   sample_type RMSE_original RMSE_gam MAD_original MAD_gam Q95_original Q95_gam
##   <chr>               <dbl>    <dbl>        <dbl>   <dbl>        <dbl>   <dbl>
## 1 cal                  134.     62.5         109.    38.6         282.    134.
## 2 sus                  150.     78.1         137.    46.5         304.    175.
## # ℹ 2 more variables: R2_original <dbl>, R2_gam <dbl>
```

```
data_xy_pred %>%
  filter(CS_x != CS_y & sample_type == "sus") %>%
  group_by(CS_x, CS_y) %>%
  summarize(RMSE_gam = rmse(RTI_y_pred, RTI_y),
            MAD_gam = mad(RTI_y_pred, RTI_y),
            Q95_gam = quantile(abs(RTI_y_pred - RTI_y), probs = c(0.95)),
            R2_gam = cor(RTI_y_pred, RTI_y)^2) %>%
  ungroup() %>%
  arrange(RMSE_gam)
```

```
## `summarise()` has grouped output by 'CS_x'. You can override using the
## `.groups` argument.
```

```
## # A tibble: 1,332 × 6
##    CS_x   CS_y   RMSE_gam MAD_gam Q95_gam R2_gam
##    <chr>  <chr>     <dbl>   <dbl>   <dbl>  <dbl>
##  1 DS_DP  DS_EF      6.74    8.67    10.3  1.00 
##  2 DS_EF  DS_DP      6.74    8.68    10.3  1.00 
##  3 DS_VQL DS_QDF     6.85    2.67    14.1  0.999
##  4 DS_QDF DS_VQL     6.93    2.68    14.4  0.999
##  5 DS_QBD DS_TSF     6.97    6.31    12.9  0.999
##  6 DS_QBD DS_HT      7.44    5.06    14.4  0.999
##  7 DS_HT  DS_QBD     8.25    5.85    15.5  0.999
##  8 DS_TSF DS_QBD     8.29    7.23    17.4  0.999
##  9 DS_HT  DS_TSF    10.3     5.42    21.9  0.998
## 10 DS_TSF DS_HT     10.4     6.28    23.3  0.997
## # ℹ 1,322 more rows
```

```
data_xy_pred %>%
  filter(CS_x != CS_y & sample_type == "sus") %>%
  group_by(CS_x, CS_y) %>%
  summarize(RMSE = rmse(RTI_y_pred, RTI_y),
            MAD = mad(RTI_y_pred, RTI_y),
            Q95 = quantile(abs(RTI_y_pred - RTI_y), probs = c(0.95)),
            R2 = cor(RTI_y_pred, RTI_y)^2) %>%
  ungroup() %>%
  group_by(CS_y) %>%
  summarize(min_RMSE = min(RMSE),
            max_RMSE = max(RMSE),
            med_RMSE = median(RMSE),
            min_MAD = min(MAD),
            max_MAD = max(MAD),
            med_MAD = median(MAD),
            min_Q95 = min(Q95),
            max_Q95 = max(Q95),
            med_Q95 = median(Q95),
            min_R2 = min(R2),
            max_R2 = max(R2),
            med_R2 = median(R2)) %>%
  ungroup() %>%
  arrange(min_RMSE)
```

```
## `summarise()` has grouped output by 'CS_x'. You can override using the
## `.groups` argument.
```

```
## # A tibble: 37 × 13
##    CS_y   min_RMSE max_RMSE med_RMSE min_MAD max_MAD med_MAD min_Q95 max_Q95
##    <chr>     <dbl>    <dbl>    <dbl>   <dbl>   <dbl>   <dbl>   <dbl>   <dbl>
##  1 DS_EF      6.74     126.     60.4    8.67   134.     38.5    10.3    229.
##  2 DS_DP      6.74     130.     62.4    8.68   124.     44.7    10.3    236.
##  3 DS_QDF     6.85     157.     88.5    2.67    92.3    44.2    14.1    287.
##  4 DS_VQL     6.93     151.     86.2    2.68   110.     46.1    14.4    288.
##  5 DS_TSF     6.97     134.     53.0    5.42   121.     37.5    12.9    298.
##  6 DS_HT      7.44     146.     56.4    5.06    80.6    39.4    14.4    289.
##  7 DS_QBD     8.25     130.     43.6    5.85   138.     32.8    15.5    275.
##  8 DS_JBQ    11.8      146.     83.1    9.84   141.     56.8    22.8    334.
##  9 DS_JSG    12.7      140.     82.6   10.7    109.     52.2    25.2    329.
## 10 DS_JBB    12.7      129.     57.3   12.5    112.     47.5    18.6    266.
## # ℹ 27 more rows
## # ℹ 4 more variables: med_Q95 <dbl>, min_R2 <dbl>, max_R2 <dbl>, med_R2 <dbl>
```

Statistical significance is tested with F-test.

```
results_var_test = tibble()
for(CS_this in levels(factor(data_xy_pred$CS_x))) {
  for(CS_that in levels(factor(data_xy_pred$CS_y))) {
    data_xy_this_that = data_xy_pred %>%
      filter(CS_x == CS_this & CS_y == CS_that & sample_type == "sus") 
    result = var.test(lm(RTI_y ~ 0 + offset(1*RTI_x), 
                         data = data_xy_this_that), 
                      lm(RTI_y ~ 0 + offset(1*RTI_y_pred), 
                         data = data_xy_this_that))
    results_var_test = results_var_test %>%
      bind_rows(tibble(CS_x = CS_this,
                       CS_y = CS_that,
                       p_value = result$p.value))
  }
}
```

Looking into combinations of CSs from the RMSE improvement point of
view.

```
data_xy_pred %>%
  filter(CS_x != CS_y & sample_type == "sus") %>%
  group_by(sample_type, CS_x, CS_y) %>%
  summarize(RMSE_original = rmse(RTI_x, RTI_y),
            RMSE_gam = rmse(RTI_y_pred, RTI_y),
            improve = case_when(
              RMSE_original > RMSE_gam ~ TRUE,
              TRUE ~ FALSE
            ),
            rate = RMSE_original/RMSE_gam) %>%
  ungroup() %>%
  left_join(results_var_test) %>%
  group_by(improve) %>%
  summarize(count_sign = sum(ifelse(p_value < 0.05, 1, 0)),
            count_insign = sum(ifelse(p_value >= 0.05, 1, 0))) %>%
  ungroup()
```

```
## `summarise()` has grouped output by 'sample_type', 'CS_x'. You can override
## using the `.groups` argument.
## Joining with `by = join_by(CS_x, CS_y)`
```

```
## # A tibble: 2 × 3
##   improve count_sign count_insign
##   <lgl>        <dbl>        <dbl>
## 1 FALSE            4           98
## 2 TRUE           768          462
```

Compare RMSE of calibrants and suspects across all labs

```
ggplot(data = data_xy_pred %>%
         group_by(sample_type, CS_x, CS_y) %>%
         summarize(RMSE_gam = rmse(RTI_y_pred, RTI_y)) %>%
         ungroup() %>%
         select(CS_x, CS_y, sample_type, RMSE_gam) %>%
         spread(key = sample_type, value = RMSE_gam)) +
  geom_point(mapping = aes(x = cal,
                           y = sus),
             color = highlightercolor6) +
  geom_abline(intercept = 0, slope = 1) +
  labs(x = "RMSE cal", y = "RMSE sus") +
  my_theme
```

```
## `summarise()` has grouped output by 'sample_type', 'CS_x'. You can override
## using the `.groups` argument.
```

Comparing the RMSE for calibrants and suspects with F-test.

```
results_var_test_cal_sus = tibble()
for(CS_this in levels(factor(data_xy_pred$CS_x))) {
  for(CS_that in levels(factor(data_xy_pred$CS_y))) {
    data_xy_this_that = data_xy_pred %>%
      filter(CS_x == CS_this & CS_y == CS_that)
    
    result = var.test(lm(RTI_y ~ 0 + offset(1*RTI_y_pred), 
                         data = data_xy_this_that %>%
                           filter(sample_type == "cal")), 
                      lm(RTI_y ~ 0 + offset(1*RTI_y_pred), 
                         data = data_xy_this_that %>%
                           filter(sample_type == "sus")))
    
    results_var_test_cal_sus = results_var_test_cal_sus %>%
      bind_rows(tibble(CS_x = CS_this,
                       CS_y = CS_that,
                       p_value = result$p.value))
  }
}

data_cal_sus = data_xy_pred %>%
  filter(CS_x != CS_y) %>%
  group_by(sample_type, CS_x, CS_y) %>%
  summarize(RMSE_gam = rmse(RTI_y_pred, RTI_y)) %>%
  ungroup() %>%
  select(CS_x, CS_y, sample_type, RMSE_gam) %>%
  spread(key = sample_type, value = RMSE_gam) %>%
  mutate(ratio = cal/sus,
         improve = case_when(
              ratio > 1 ~ TRUE,
              TRUE ~ FALSE
            )) %>%
  left_join(results_var_test_cal_sus)
```

```
## `summarise()` has grouped output by 'sample_type', 'CS_x'. You can override
## using the `.groups` argument.
## Joining with `by = join_by(CS_x, CS_y)`
```

```
data_cal_sus %>%
  arrange(improve, ratio)
```

```
## # A tibble: 1,332 × 7
##    CS_x   CS_y       cal    sus   ratio improve  p_value
##    <chr>  <chr>    <dbl>  <dbl>   <dbl> <lgl>      <dbl>
##  1 DS_DP  DS_EF   0.0482   6.74 0.00716 FALSE   1.68e-53
##  2 DS_EF  DS_DP   0.0482   6.74 0.00716 FALSE   1.68e-53
##  3 DS_QDJ DS_JWW  1.03   133.   0.00774 FALSE   4.00e-14
##  4 DS_JWW DS_QDJ  3.02   157.   0.0192  FALSE   2.33e-11
##  5 DS_QDJ DS_QDF  7.25   104.   0.0696  FALSE   3.81e-11
##  6 DS_GJT DS_QDJ 11.9    150.   0.0794  FALSE   4.83e- 7
##  7 DS_AWW DS_QDJ  8.49    98.1  0.0865  FALSE   3.91e-10
##  8 DS_QDJ DS_QSB  4.74    49.8  0.0952  FALSE   1.08e- 9
##  9 DS_JL  DS_QDJ  9.84    98.0  0.100   FALSE   3.52e- 7
## 10 DS_QDF DS_QDJ  9.94    96.8  0.103   FALSE   2.63e- 9
## # ℹ 1,322 more rows
```

```
data_cal_sus %>%
  group_by(improve) %>%
  summarize(count_sign = sum(ifelse(p_value < 0.05, 1, 0)),
            count_insign = sum(ifelse(p_value >= 0.05, 1, 0))) %>%
  ungroup()
```

```
## # A tibble: 2 × 3
##   improve count_sign count_insign
##   <lgl>        <dbl>        <dbl>
## 1 FALSE          535          480
## 2 TRUE            63          254
```
