# Supplementary material for: Do experimental projection methods outcompete retention time prediction models in non-target screening? A case study on LC/HRMS interlaboratory comparison data
Source: Analyst. 2025 Jul 8;150(16):3567–77. doi: 10.1039/d5an00323g (PMC12268317; doi:10.1039/d5an00323g)
Supplement: AN-150-D5AN00323G-s001 [file AN-150-D5AN00323G-s001.zip › SI/SI3_prediction_RF_GAM.html]

RT prediction model training


# RT prediction model training

#### Anneli Kruve

#### 2025-02-14

## Libraries

## Functions for preprocessing and modelling

```
remove_correlated_nearZero = function(data = tibble(),
                                      cutoff = numeric()) {
  data = data %>%
    mutate(Lipinski = as.numeric(Lipinski),
           GhoseFilter = as.numeric(GhoseFilter))
  
  data = data %>%
    relocate(c(SMILES, RT, mol, standardized_SMILES, standardized_inchikey, MF), .after = last_col())
  
  data = data %>%
    select(-nearZeroVar(data %>%
                          select(-c(SMILES, RT, mol, standardized_SMILES, standardized_inchikey, MF))))
  
  correlation_matrix = cor(data %>%
                             select(-c(SMILES, RT, mol, standardized_SMILES, standardized_inchikey, MF)))
  
  highly_correlated = findCorrelation(correlation_matrix,
                                      cutoff = cutoff)
  
  data = data %>%
    select(-all_of(highly_correlated))
  
  data = data %>%
    select(SMILES, RT, mol, standardized_SMILES, standardized_inchikey, MF, colnames(data))
  
  return(data)
  
}

model_smart_combined = function(training_data = tibble(),
                                test_data = tibble(),
                                cutoff_correlated = 0.7,
                                cross_validation_folds = 5) {
  
  training_data = remove_correlated_nearZero(training_data,
                                             cutoff_correlated)
  
  test_data = test_data %>%
    mutate(Lipinski = as.numeric(Lipinski),
           GhoseFilter = as.numeric(GhoseFilter))
  
  test_data = test_data %>%
    select(colnames(training_data %>% select(-RT))) %>%
    na.omit()
  
  folds = groupKFold(training_data$MF,
                     k = cross_validation_folds)
  
  fitControl = trainControl(method = "repeatedcv",
                            number = 2,
                            repeats = 5)
  
  model_xgbTREE = train(RT ~ ., 
                        data = training_data %>%
                          select(-c(SMILES, mol, standardized_SMILES, standardized_inchikey, MF)), 
                        method = "xgbTree", 
                        trControl = fitControl,
                        verbose = FALSE)
  
  training_data = training_data %>%
    mutate(RT_xgbTREE_pred = predict(model_xgbTREE,
                                     newdata = training_data)) %>%
    select(SMILES, RT, mol, standardized_SMILES, standardized_inchikey, MF, RT_xgbTREE_pred)
  
  test_data = test_data %>%
    mutate(RT_xgbTREE_pred = predict(model_xgbTREE,
                                     newdata = test_data)) %>%
    select(SMILES, mol, standardized_SMILES, standardized_inchikey, MF, RT_xgbTREE_pred)
  
  all = list("training_data" = training_data,
             "test_data" = test_data)
  
  return(all)
}
```

## Reading in data

Cleaned RT data from Aalizadeh et al. with respective Mordred
descriptors.

```
data_training = read_delim("data/training_data_Athens.csv")
```

Mordred descriptors for the calibrants and suspects from NORMAN
interlaboratory comparison.

```
data_norman = read_delim("data/NORMAN_Mordreds_2024-11-02.tsv")
```

Experimental RT data from the interlaboratory comparison.

```
data = read_delim("data/NORMAN_interlab_RT_data.csv")
```

## Modelling retention time based on data from Aalizadeh et al.

```
set.seed(123)
preProcValues = preProcess(data_training %>% 
                             select(-c(SMILES, RT, mol, standardized_SMILES, standardized_inchikey, MF)), 
                           method = c("center", "scale"))

trainTransformed = predict(preProcValues, data_training)
normanTransformed = predict(preProcValues, data_norman)
```

```
## [22:18:53] WARNING: src/c_api/c_api.cc:935: `ntree_limit` is deprecated, use `iteration_range` instead.
## [22:18:53] WARNING: src/c_api/c_api.cc:935: `ntree_limit` is deprecated, use `iteration_range` instead.
## [22:18:54] WARNING: src/c_api/c_api.cc:935: `ntree_limit` is deprecated, use `iteration_range` instead.
## [22:18:54] WARNING: src/c_api/c_api.cc:935: `ntree_limit` is deprecated, use `iteration_range` instead.
## [22:18:54] WARNING: src/c_api/c_api.cc:935: `ntree_limit` is deprecated, use `iteration_range` instead.
## [22:18:54] WARNING: src/c_api/c_api.cc:935: `ntree_limit` is deprecated, use `iteration_range` instead.
## [22:18:54] WARNING: src/c_api/c_api.cc:935: `ntree_limit` is deprecated, use `iteration_range` instead.
## [22:18:54] WARNING: src/c_api/c_api.cc:935: `ntree_limit` is deprecated, use `iteration_range` instead.
## [22:18:54] WARNING: src/c_api/c_api.cc:935: `ntree_limit` is deprecated, use `iteration_range` instead.
## [22:18:54] WARNING: src/c_api/c_api.cc:935: `ntree_limit` is deprecated, use `iteration_range` instead.
## [22:18:54] WARNING: src/c_api/c_api.cc:935: `ntree_limit` is deprecated, use `iteration_range` instead.
## [22:18:54] WARNING: src/c_api/c_api.cc:935: `ntree_limit` is deprecated, use `iteration_range` instead.
## [22:18:55] WARNING: src/c_api/c_api.cc:935: `ntree_limit` is deprecated, use `iteration_range` instead.
## [22:18:55] WARNING: src/c_api/c_api.cc:935: `ntree_limit` is deprecated, use `iteration_range` instead.
## [22:18:55] WARNING: src/c_api/c_api.cc:935: `ntree_limit` is deprecated, use `iteration_range` instead.
## [22:18:55] WARNING: src/c_api/c_api.cc:935: `ntree_limit` is deprecated, use `iteration_range` instead.
## [22:18:55] WARNING: src/c_api/c_api.cc:935: `ntree_limit` is deprecated, use `iteration_range` instead.
## [22:18:55] WARNING: src/c_api/c_api.cc:935: `ntree_limit` is deprecated, use `iteration_range` instead.
## [22:18:56] WARNING: src/c_api/c_api.cc:935: `ntree_limit` is deprecated, use `iteration_range` instead.
## [22:18:56] WARNING: src/c_api/c_api.cc:935: `ntree_limit` is deprecated, use `iteration_range` instead.
## [22:18:56] WARNING: src/c_api/c_api.cc:935: `ntree_limit` is deprecated, use `iteration_range` instead.
## [22:18:56] WARNING: src/c_api/c_api.cc:935: `ntree_limit` is deprecated, use `iteration_range` instead.
## [22:18:56] WARNING: src/c_api/c_api.cc:935: `ntree_limit` is deprecated, use `iteration_range` instead.
## [22:18:56] WARNING: src/c_api/c_api.cc:935: `ntree_limit` is deprecated, use `iteration_range` instead.
## [22:18:57] WARNING: src/c_api/c_api.cc:935: `ntree_limit` is deprecated, use `iteration_range` instead.
## [22:18:57] WARNING: src/c_api/c_api.cc:935: `ntree_limit` is deprecated, use `iteration_range` instead.
## [22:18:57] WARNING: src/c_api/c_api.cc:935: `ntree_limit` is deprecated, use `iteration_range` instead.
## [22:18:57] WARNING: src/c_api/c_api.cc:935: `ntree_limit` is deprecated, use `iteration_range` instead.
## [22:18:58] WARNING: src/c_api/c_api.cc:935: `ntree_limit` is deprecated, use `iteration_range` instead.
## [22:18:58] WARNING: src/c_api/c_api.cc:935: `ntree_limit` is deprecated, use `iteration_range` instead.
## [22:18:58] WARNING: src/c_api/c_api.cc:935: `ntree_limit` is deprecated, use `iteration_range` instead.
## [22:18:58] WARNING: src/c_api/c_api.cc:935: `ntree_limit` is deprecated, use `iteration_range` instead.
## [22:18:59] WARNING: src/c_api/c_api.cc:935: `ntree_limit` is deprecated, use `iteration_range` instead.
## [22:18:59] WARNING: src/c_api/c_api.cc:935: `ntree_limit` is deprecated, use `iteration_range` instead.
## [22:18:59] WARNING: src/c_api/c_api.cc:935: `ntree_limit` is deprecated, use `iteration_range` instead.
## [22:18:59] WARNING: src/c_api/c_api.cc:935: `ntree_limit` is deprecated, use `iteration_range` instead.
## [22:19:00] WARNING: src/c_api/c_api.cc:935: `ntree_limit` is deprecated, use `iteration_range` instead.
## [22:19:00] WARNING: src/c_api/c_api.cc:935: `ntree_limit` is deprecated, use `iteration_range` instead.
## [22:19:00] WARNING: src/c_api/c_api.cc:935: `ntree_limit` is deprecated, use `iteration_range` instead.
## [22:19:00] WARNING: src/c_api/c_api.cc:935: `ntree_limit` is deprecated, use `iteration_range` instead.
## [22:19:00] WARNING: src/c_api/c_api.cc:935: `ntree_limit` is deprecated, use `iteration_range` instead.
## [22:19:00] WARNING: src/c_api/c_api.cc:935: `ntree_limit` is deprecated, use `iteration_range` instead.
## [22:19:00] WARNING: src/c_api/c_api.cc:935: `ntree_limit` is deprecated, use `iteration_range` instead.
## [22:19:00] WARNING: src/c_api/c_api.cc:935: `ntree_limit` is deprecated, use `iteration_range` instead.
## [22:19:00] WARNING: src/c_api/c_api.cc:935: `ntree_limit` is deprecated, use `iteration_range` instead.
## [22:19:00] WARNING: src/c_api/c_api.cc:935: `ntree_limit` is deprecated, use `iteration_range` instead.
## [22:19:01] WARNING: src/c_api/c_api.cc:935: `ntree_limit` is deprecated, use `iteration_range` instead.
## [22:19:01] WARNING: src/c_api/c_api.cc:935: `ntree_limit` is deprecated, use `iteration_range` instead.
## [22:19:01] WARNING: src/c_api/c_api.cc:935: `ntree_limit` is deprecated, use `iteration_range` instead.
## [22:19:01] WARNING: src/c_api/c_api.cc:935: `ntree_limit` is deprecated, use `iteration_range` instead.
## [22:19:01] WARNING: src/c_api/c_api.cc:935: `ntree_limit` is deprecated, use `iteration_range` instead.
## [22:19:01] WARNING: src/c_api/c_api.cc:935: `ntree_limit` is deprecated, use `iteration_range` instead.
## [22:19:02] WARNING: src/c_api/c_api.cc:935: `ntree_limit` is deprecated, use `iteration_range` instead.
## [22:19:02] WARNING: src/c_api/c_api.cc:935: `ntree_limit` is deprecated, use `iteration_range` instead.
## [22:19:02] WARNING: src/c_api/c_api.cc:935: `ntree_limit` is deprecated, use `iteration_range` instead.
## [22:19:02] WARNING: src/c_api/c_api.cc:935: `ntree_limit` is deprecated, use `iteration_range` instead.
## [22:19:02] WARNING: src/c_api/c_api.cc:935: `ntree_limit` is deprecated, use `iteration_range` instead.
## [22:19:02] WARNING: src/c_api/c_api.cc:935: `ntree_limit` is deprecated, use `iteration_range` instead.
## [22:19:03] WARNING: src/c_api/c_api.cc:935: `ntree_limit` is deprecated, use `iteration_range` instead.
## [22:19:03] WARNING: src/c_api/c_api.cc:935: `ntree_limit` is deprecated, use `iteration_range` instead.
## [22:19:03] WARNING: src/c_api/c_api.cc:935: `ntree_limit` is deprecated, use `iteration_range` instead.
## [22:19:03] WARNING: src/c_api/c_api.cc:935: `ntree_limit` is deprecated, use `iteration_range` instead.
## [22:19:04] WARNING: src/c_api/c_api.cc:935: `ntree_limit` is deprecated, use `iteration_range` instead.
## [22:19:04] WARNING: src/c_api/c_api.cc:935: `ntree_limit` is deprecated, use `iteration_range` instead.
## [22:19:04] WARNING: src/c_api/c_api.cc:935: `ntree_limit` is deprecated, use `iteration_range` instead.
## [22:19:04] WARNING: src/c_api/c_api.cc:935: `ntree_limit` is deprecated, use `iteration_range` instead.
## [22:19:05] WARNING: src/c_api/c_api.cc:935: `ntree_limit` is deprecated, use `iteration_range` instead.
## [22:19:05] WARNING: src/c_api/c_api.cc:935: `ntree_limit` is deprecated, use `iteration_range` instead.
## [22:19:05] WARNING: src/c_api/c_api.cc:935: `ntree_limit` is deprecated, use `iteration_range` instead.
## [22:19:05] WARNING: src/c_api/c_api.cc:935: `ntree_limit` is deprecated, use `iteration_range` instead.
## [22:19:06] WARNING: src/c_api/c_api.cc:935: `ntree_limit` is deprecated, use `iteration_range` instead.
## [22:19:06] WARNING: src/c_api/c_api.cc:935: `ntree_limit` is deprecated, use `iteration_range` instead.
## [22:19:06] WARNING: src/c_api/c_api.cc:935: `ntree_limit` is deprecated, use `iteration_range` instead.
## [22:19:06] WARNING: src/c_api/c_api.cc:935: `ntree_limit` is deprecated, use `iteration_range` instead.
## [22:19:06] WARNING: src/c_api/c_api.cc:935: `ntree_limit` is deprecated, use `iteration_range` instead.
## [22:19:06] WARNING: src/c_api/c_api.cc:935: `ntree_limit` is deprecated, use `iteration_range` instead.
## [22:19:06] WARNING: src/c_api/c_api.cc:935: `ntree_limit` is deprecated, use `iteration_range` instead.
## [22:19:06] WARNING: src/c_api/c_api.cc:935: `ntree_limit` is deprecated, use `iteration_range` instead.
## [22:19:07] WARNING: src/c_api/c_api.cc:935: `ntree_limit` is deprecated, use `iteration_range` instead.
## [22:19:07] WARNING: src/c_api/c_api.cc:935: `ntree_limit` is deprecated, use `iteration_range` instead.
## [22:19:07] WARNING: src/c_api/c_api.cc:935: `ntree_limit` is deprecated, use `iteration_range` instead.
## [22:19:07] WARNING: src/c_api/c_api.cc:935: `ntree_limit` is deprecated, use `iteration_range` instead.
## [22:19:07] WARNING: src/c_api/c_api.cc:935: `ntree_limit` is deprecated, use `iteration_range` instead.
## [22:19:07] WARNING: src/c_api/c_api.cc:935: `ntree_limit` is deprecated, use `iteration_range` instead.
## [22:19:07] WARNING: src/c_api/c_api.cc:935: `ntree_limit` is deprecated, use `iteration_range` instead.
## [22:19:07] WARNING: src/c_api/c_api.cc:935: `ntree_limit` is deprecated, use `iteration_range` instead.
## [22:19:08] WARNING: src/c_api/c_api.cc:935: `ntree_limit` is deprecated, use `iteration_range` instead.
## [22:19:08] WARNING: src/c_api/c_api.cc:935: `ntree_limit` is deprecated, use `iteration_range` instead.
## [22:19:08] WARNING: src/c_api/c_api.cc:935: `ntree_limit` is deprecated, use `iteration_range` instead.
## [22:19:08] WARNING: src/c_api/c_api.cc:935: `ntree_limit` is deprecated, use `iteration_range` instead.
## [22:19:08] WARNING: src/c_api/c_api.cc:935: `ntree_limit` is deprecated, use `iteration_range` instead.
## [22:19:08] WARNING: src/c_api/c_api.cc:935: `ntree_limit` is deprecated, use `iteration_range` instead.
## [22:19:09] WARNING: src/c_api/c_api.cc:935: `ntree_limit` is deprecated, use `iteration_range` instead.
## [22:19:09] WARNING: src/c_api/c_api.cc:935: `ntree_limit` is deprecated, use `iteration_range` instead.
## [22:19:09] WARNING: src/c_api/c_api.cc:935: `ntree_limit` is deprecated, use `iteration_range` instead.
## [22:19:09] WARNING: src/c_api/c_api.cc:935: `ntree_limit` is deprecated, use `iteration_range` instead.
## [22:19:10] WARNING: src/c_api/c_api.cc:935: `ntree_limit` is deprecated, use `iteration_range` instead.
## [22:19:10] WARNING: src/c_api/c_api.cc:935: `ntree_limit` is deprecated, use `iteration_range` instead.
## [22:19:10] WARNING: src/c_api/c_api.cc:935: `ntree_limit` is deprecated, use `iteration_range` instead.
## [22:19:10] WARNING: src/c_api/c_api.cc:935: `ntree_limit` is deprecated, use `iteration_range` instead.
## [22:19:11] WARNING: src/c_api/c_api.cc:935: `ntree_limit` is deprecated, use `iteration_range` instead.
## [22:19:11] WARNING: src/c_api/c_api.cc:935: `ntree_limit` is deprecated, use `iteration_range` instead.
## [22:19:11] WARNING: src/c_api/c_api.cc:935: `ntree_limit` is deprecated, use `iteration_range` instead.
## [22:19:11] WARNING: src/c_api/c_api.cc:935: `ntree_limit` is deprecated, use `iteration_range` instead.
## [22:19:12] WARNING: src/c_api/c_api.cc:935: `ntree_limit` is deprecated, use `iteration_range` instead.
## [22:19:12] WARNING: src/c_api/c_api.cc:935: `ntree_limit` is deprecated, use `iteration_range` instead.
## [22:19:12] WARNING: src/c_api/c_api.cc:935: `ntree_limit` is deprecated, use `iteration_range` instead.
## [22:19:12] WARNING: src/c_api/c_api.cc:935: `ntree_limit` is deprecated, use `iteration_range` instead.
## [22:19:12] WARNING: src/c_api/c_api.cc:935: `ntree_limit` is deprecated, use `iteration_range` instead.
## [22:19:12] WARNING: src/c_api/c_api.cc:935: `ntree_limit` is deprecated, use `iteration_range` instead.
## [22:19:13] WARNING: src/c_api/c_api.cc:935: `ntree_limit` is deprecated, use `iteration_range` instead.
## [22:19:13] WARNING: src/c_api/c_api.cc:935: `ntree_limit` is deprecated, use `iteration_range` instead.
## [22:19:13] WARNING: src/c_api/c_api.cc:935: `ntree_limit` is deprecated, use `iteration_range` instead.
## [22:19:13] WARNING: src/c_api/c_api.cc:935: `ntree_limit` is deprecated, use `iteration_range` instead.
## [22:19:13] WARNING: src/c_api/c_api.cc:935: `ntree_limit` is deprecated, use `iteration_range` instead.
## [22:19:13] WARNING: src/c_api/c_api.cc:935: `ntree_limit` is deprecated, use `iteration_range` instead.
## [22:19:13] WARNING: src/c_api/c_api.cc:935: `ntree_limit` is deprecated, use `iteration_range` instead.
## [22:19:13] WARNING: src/c_api/c_api.cc:935: `ntree_limit` is deprecated, use `iteration_range` instead.
## [22:19:14] WARNING: src/c_api/c_api.cc:935: `ntree_limit` is deprecated, use `iteration_range` instead.
## [22:19:14] WARNING: src/c_api/c_api.cc:935: `ntree_limit` is deprecated, use `iteration_range` instead.
## [22:19:14] WARNING: src/c_api/c_api.cc:935: `ntree_limit` is deprecated, use `iteration_range` instead.
## [22:19:14] WARNING: src/c_api/c_api.cc:935: `ntree_limit` is deprecated, use `iteration_range` instead.
## [22:19:14] WARNING: src/c_api/c_api.cc:935: `ntree_limit` is deprecated, use `iteration_range` instead.
## [22:19:14] WARNING: src/c_api/c_api.cc:935: `ntree_limit` is deprecated, use `iteration_range` instead.
## [22:19:15] WARNING: src/c_api/c_api.cc:935: `ntree_limit` is deprecated, use `iteration_range` instead.
## [22:19:15] WARNING: src/c_api/c_api.cc:935: `ntree_limit` is deprecated, use `iteration_range` instead.
## [22:19:15] WARNING: src/c_api/c_api.cc:935: `ntree_limit` is deprecated, use `iteration_range` instead.
## [22:19:15] WARNING: src/c_api/c_api.cc:935: `ntree_limit` is deprecated, use `iteration_range` instead.
## [22:19:15] WARNING: src/c_api/c_api.cc:935: `ntree_limit` is deprecated, use `iteration_range` instead.
## [22:19:15] WARNING: src/c_api/c_api.cc:935: `ntree_limit` is deprecated, use `iteration_range` instead.
## [22:19:16] WARNING: src/c_api/c_api.cc:935: `ntree_limit` is deprecated, use `iteration_range` instead.
## [22:19:16] WARNING: src/c_api/c_api.cc:935: `ntree_limit` is deprecated, use `iteration_range` instead.
## [22:19:16] WARNING: src/c_api/c_api.cc:935: `ntree_limit` is deprecated, use `iteration_range` instead.
## [22:19:16] WARNING: src/c_api/c_api.cc:935: `ntree_limit` is deprecated, use `iteration_range` instead.
## [22:19:17] WARNING: src/c_api/c_api.cc:935: `ntree_limit` is deprecated, use `iteration_range` instead.
## [22:19:17] WARNING: src/c_api/c_api.cc:935: `ntree_limit` is deprecated, use `iteration_range` instead.
## [22:19:17] WARNING: src/c_api/c_api.cc:935: `ntree_limit` is deprecated, use `iteration_range` instead.
## [22:19:17] WARNING: src/c_api/c_api.cc:935: `ntree_limit` is deprecated, use `iteration_range` instead.
## [22:19:18] WARNING: src/c_api/c_api.cc:935: `ntree_limit` is deprecated, use `iteration_range` instead.
## [22:19:18] WARNING: src/c_api/c_api.cc:935: `ntree_limit` is deprecated, use `iteration_range` instead.
## [22:19:18] WARNING: src/c_api/c_api.cc:935: `ntree_limit` is deprecated, use `iteration_range` instead.
## [22:19:18] WARNING: src/c_api/c_api.cc:935: `ntree_limit` is deprecated, use `iteration_range` instead.
## [22:19:19] WARNING: src/c_api/c_api.cc:935: `ntree_limit` is deprecated, use `iteration_range` instead.
## [22:19:19] WARNING: src/c_api/c_api.cc:935: `ntree_limit` is deprecated, use `iteration_range` instead.
## [22:19:19] WARNING: src/c_api/c_api.cc:935: `ntree_limit` is deprecated, use `iteration_range` instead.
## [22:19:19] WARNING: src/c_api/c_api.cc:935: `ntree_limit` is deprecated, use `iteration_range` instead.
## [22:19:19] WARNING: src/c_api/c_api.cc:935: `ntree_limit` is deprecated, use `iteration_range` instead.
## [22:19:19] WARNING: src/c_api/c_api.cc:935: `ntree_limit` is deprecated, use `iteration_range` instead.
## [22:19:20] WARNING: src/c_api/c_api.cc:935: `ntree_limit` is deprecated, use `iteration_range` instead.
## [22:19:20] WARNING: src/c_api/c_api.cc:935: `ntree_limit` is deprecated, use `iteration_range` instead.
## [22:19:20] WARNING: src/c_api/c_api.cc:935: `ntree_limit` is deprecated, use `iteration_range` instead.
## [22:19:20] WARNING: src/c_api/c_api.cc:935: `ntree_limit` is deprecated, use `iteration_range` instead.
## [22:19:20] WARNING: src/c_api/c_api.cc:935: `ntree_limit` is deprecated, use `iteration_range` instead.
## [22:19:20] WARNING: src/c_api/c_api.cc:935: `ntree_limit` is deprecated, use `iteration_range` instead.
## [22:19:20] WARNING: src/c_api/c_api.cc:935: `ntree_limit` is deprecated, use `iteration_range` instead.
## [22:19:20] WARNING: src/c_api/c_api.cc:935: `ntree_limit` is deprecated, use `iteration_range` instead.
## [22:19:21] WARNING: src/c_api/c_api.cc:935: `ntree_limit` is deprecated, use `iteration_range` instead.
## [22:19:21] WARNING: src/c_api/c_api.cc:935: `ntree_limit` is deprecated, use `iteration_range` instead.
## [22:19:21] WARNING: src/c_api/c_api.cc:935: `ntree_limit` is deprecated, use `iteration_range` instead.
## [22:19:21] WARNING: src/c_api/c_api.cc:935: `ntree_limit` is deprecated, use `iteration_range` instead.
## [22:19:21] WARNING: src/c_api/c_api.cc:935: `ntree_limit` is deprecated, use `iteration_range` instead.
## [22:19:21] WARNING: src/c_api/c_api.cc:935: `ntree_limit` is deprecated, use `iteration_range` instead.
## [22:19:22] WARNING: src/c_api/c_api.cc:935: `ntree_limit` is deprecated, use `iteration_range` instead.
## [22:19:22] WARNING: src/c_api/c_api.cc:935: `ntree_limit` is deprecated, use `iteration_range` instead.
## [22:19:22] WARNING: src/c_api/c_api.cc:935: `ntree_limit` is deprecated, use `iteration_range` instead.
## [22:19:22] WARNING: src/c_api/c_api.cc:935: `ntree_limit` is deprecated, use `iteration_range` instead.
## [22:19:22] WARNING: src/c_api/c_api.cc:935: `ntree_limit` is deprecated, use `iteration_range` instead.
## [22:19:22] WARNING: src/c_api/c_api.cc:935: `ntree_limit` is deprecated, use `iteration_range` instead.
## [22:19:23] WARNING: src/c_api/c_api.cc:935: `ntree_limit` is deprecated, use `iteration_range` instead.
## [22:19:23] WARNING: src/c_api/c_api.cc:935: `ntree_limit` is deprecated, use `iteration_range` instead.
## [22:19:23] WARNING: src/c_api/c_api.cc:935: `ntree_limit` is deprecated, use `iteration_range` instead.
## [22:19:23] WARNING: src/c_api/c_api.cc:935: `ntree_limit` is deprecated, use `iteration_range` instead.
## [22:19:24] WARNING: src/c_api/c_api.cc:935: `ntree_limit` is deprecated, use `iteration_range` instead.
## [22:19:24] WARNING: src/c_api/c_api.cc:935: `ntree_limit` is deprecated, use `iteration_range` instead.
## [22:19:24] WARNING: src/c_api/c_api.cc:935: `ntree_limit` is deprecated, use `iteration_range` instead.
## [22:19:24] WARNING: src/c_api/c_api.cc:935: `ntree_limit` is deprecated, use `iteration_range` instead.
## [22:19:25] WARNING: src/c_api/c_api.cc:935: `ntree_limit` is deprecated, use `iteration_range` instead.
## [22:19:25] WARNING: src/c_api/c_api.cc:935: `ntree_limit` is deprecated, use `iteration_range` instead.
## [22:19:26] WARNING: src/c_api/c_api.cc:935: `ntree_limit` is deprecated, use `iteration_range` instead.
## [22:19:26] WARNING: src/c_api/c_api.cc:935: `ntree_limit` is deprecated, use `iteration_range` instead.
## [22:19:26] WARNING: src/c_api/c_api.cc:935: `ntree_limit` is deprecated, use `iteration_range` instead.
## [22:19:26] WARNING: src/c_api/c_api.cc:935: `ntree_limit` is deprecated, use `iteration_range` instead.
## [22:19:26] WARNING: src/c_api/c_api.cc:935: `ntree_limit` is deprecated, use `iteration_range` instead.
## [22:19:26] WARNING: src/c_api/c_api.cc:935: `ntree_limit` is deprecated, use `iteration_range` instead.
## [22:19:26] WARNING: src/c_api/c_api.cc:935: `ntree_limit` is deprecated, use `iteration_range` instead.
## [22:19:26] WARNING: src/c_api/c_api.cc:935: `ntree_limit` is deprecated, use `iteration_range` instead.
## [22:19:27] WARNING: src/c_api/c_api.cc:935: `ntree_limit` is deprecated, use `iteration_range` instead.
## [22:19:27] WARNING: src/c_api/c_api.cc:935: `ntree_limit` is deprecated, use `iteration_range` instead.
## [22:19:27] WARNING: src/c_api/c_api.cc:935: `ntree_limit` is deprecated, use `iteration_range` instead.
## [22:19:27] WARNING: src/c_api/c_api.cc:935: `ntree_limit` is deprecated, use `iteration_range` instead.
## [22:19:27] WARNING: src/c_api/c_api.cc:935: `ntree_limit` is deprecated, use `iteration_range` instead.
## [22:19:27] WARNING: src/c_api/c_api.cc:935: `ntree_limit` is deprecated, use `iteration_range` instead.
## [22:19:27] WARNING: src/c_api/c_api.cc:935: `ntree_limit` is deprecated, use `iteration_range` instead.
## [22:19:27] WARNING: src/c_api/c_api.cc:935: `ntree_limit` is deprecated, use `iteration_range` instead.
## [22:19:28] WARNING: src/c_api/c_api.cc:935: `ntree_limit` is deprecated, use `iteration_range` instead.
## [22:19:28] WARNING: src/c_api/c_api.cc:935: `ntree_limit` is deprecated, use `iteration_range` instead.
## [22:19:28] WARNING: src/c_api/c_api.cc:935: `ntree_limit` is deprecated, use `iteration_range` instead.
## [22:19:28] WARNING: src/c_api/c_api.cc:935: `ntree_limit` is deprecated, use `iteration_range` instead.
## [22:19:28] WARNING: src/c_api/c_api.cc:935: `ntree_limit` is deprecated, use `iteration_range` instead.
## [22:19:28] WARNING: src/c_api/c_api.cc:935: `ntree_limit` is deprecated, use `iteration_range` instead.
## [22:19:29] WARNING: src/c_api/c_api.cc:935: `ntree_limit` is deprecated, use `iteration_range` instead.
## [22:19:29] WARNING: src/c_api/c_api.cc:935: `ntree_limit` is deprecated, use `iteration_range` instead.
## [22:19:29] WARNING: src/c_api/c_api.cc:935: `ntree_limit` is deprecated, use `iteration_range` instead.
## [22:19:29] WARNING: src/c_api/c_api.cc:935: `ntree_limit` is deprecated, use `iteration_range` instead.
## [22:19:30] WARNING: src/c_api/c_api.cc:935: `ntree_limit` is deprecated, use `iteration_range` instead.
## [22:19:30] WARNING: src/c_api/c_api.cc:935: `ntree_limit` is deprecated, use `iteration_range` instead.
## [22:19:30] WARNING: src/c_api/c_api.cc:935: `ntree_limit` is deprecated, use `iteration_range` instead.
## [22:19:30] WARNING: src/c_api/c_api.cc:935: `ntree_limit` is deprecated, use `iteration_range` instead.
## [22:19:31] WARNING: src/c_api/c_api.cc:935: `ntree_limit` is deprecated, use `iteration_range` instead.
## [22:19:31] WARNING: src/c_api/c_api.cc:935: `ntree_limit` is deprecated, use `iteration_range` instead.
## [22:19:31] WARNING: src/c_api/c_api.cc:935: `ntree_limit` is deprecated, use `iteration_range` instead.
## [22:19:31] WARNING: src/c_api/c_api.cc:935: `ntree_limit` is deprecated, use `iteration_range` instead.
## [22:19:32] WARNING: src/c_api/c_api.cc:935: `ntree_limit` is deprecated, use `iteration_range` instead.
## [22:19:32] WARNING: src/c_api/c_api.cc:935: `ntree_limit` is deprecated, use `iteration_range` instead.
## [22:19:32] WARNING: src/c_api/c_api.cc:935: `ntree_limit` is deprecated, use `iteration_range` instead.
## [22:19:32] WARNING: src/c_api/c_api.cc:935: `ntree_limit` is deprecated, use `iteration_range` instead.
## [22:19:33] WARNING: src/c_api/c_api.cc:935: `ntree_limit` is deprecated, use `iteration_range` instead.
## [22:19:33] WARNING: src/c_api/c_api.cc:935: `ntree_limit` is deprecated, use `iteration_range` instead.
## [22:19:33] WARNING: src/c_api/c_api.cc:935: `ntree_limit` is deprecated, use `iteration_range` instead.
## [22:19:33] WARNING: src/c_api/c_api.cc:935: `ntree_limit` is deprecated, use `iteration_range` instead.
## [22:19:33] WARNING: src/c_api/c_api.cc:935: `ntree_limit` is deprecated, use `iteration_range` instead.
## [22:19:33] WARNING: src/c_api/c_api.cc:935: `ntree_limit` is deprecated, use `iteration_range` instead.
## [22:19:33] WARNING: src/c_api/c_api.cc:935: `ntree_limit` is deprecated, use `iteration_range` instead.
## [22:19:33] WARNING: src/c_api/c_api.cc:935: `ntree_limit` is deprecated, use `iteration_range` instead.
## [22:19:34] WARNING: src/c_api/c_api.cc:935: `ntree_limit` is deprecated, use `iteration_range` instead.
## [22:19:34] WARNING: src/c_api/c_api.cc:935: `ntree_limit` is deprecated, use `iteration_range` instead.
## [22:19:34] WARNING: src/c_api/c_api.cc:935: `ntree_limit` is deprecated, use `iteration_range` instead.
## [22:19:34] WARNING: src/c_api/c_api.cc:935: `ntree_limit` is deprecated, use `iteration_range` instead.
## [22:19:34] WARNING: src/c_api/c_api.cc:935: `ntree_limit` is deprecated, use `iteration_range` instead.
## [22:19:34] WARNING: src/c_api/c_api.cc:935: `ntree_limit` is deprecated, use `iteration_range` instead.
## [22:19:34] WARNING: src/c_api/c_api.cc:935: `ntree_limit` is deprecated, use `iteration_range` instead.
## [22:19:34] WARNING: src/c_api/c_api.cc:935: `ntree_limit` is deprecated, use `iteration_range` instead.
## [22:19:35] WARNING: src/c_api/c_api.cc:935: `ntree_limit` is deprecated, use `iteration_range` instead.
## [22:19:35] WARNING: src/c_api/c_api.cc:935: `ntree_limit` is deprecated, use `iteration_range` instead.
## [22:19:35] WARNING: src/c_api/c_api.cc:935: `ntree_limit` is deprecated, use `iteration_range` instead.
## [22:19:35] WARNING: src/c_api/c_api.cc:935: `ntree_limit` is deprecated, use `iteration_range` instead.
## [22:19:36] WARNING: src/c_api/c_api.cc:935: `ntree_limit` is deprecated, use `iteration_range` instead.
## [22:19:36] WARNING: src/c_api/c_api.cc:935: `ntree_limit` is deprecated, use `iteration_range` instead.
## [22:19:36] WARNING: src/c_api/c_api.cc:935: `ntree_limit` is deprecated, use `iteration_range` instead.
## [22:19:36] WARNING: src/c_api/c_api.cc:935: `ntree_limit` is deprecated, use `iteration_range` instead.
## [22:19:36] WARNING: src/c_api/c_api.cc:935: `ntree_limit` is deprecated, use `iteration_range` instead.
## [22:19:36] WARNING: src/c_api/c_api.cc:935: `ntree_limit` is deprecated, use `iteration_range` instead.
## [22:19:37] WARNING: src/c_api/c_api.cc:935: `ntree_limit` is deprecated, use `iteration_range` instead.
## [22:19:37] WARNING: src/c_api/c_api.cc:935: `ntree_limit` is deprecated, use `iteration_range` instead.
## [22:19:37] WARNING: src/c_api/c_api.cc:935: `ntree_limit` is deprecated, use `iteration_range` instead.
## [22:19:37] WARNING: src/c_api/c_api.cc:935: `ntree_limit` is deprecated, use `iteration_range` instead.
## [22:19:38] WARNING: src/c_api/c_api.cc:935: `ntree_limit` is deprecated, use `iteration_range` instead.
## [22:19:38] WARNING: src/c_api/c_api.cc:935: `ntree_limit` is deprecated, use `iteration_range` instead.
## [22:19:39] WARNING: src/c_api/c_api.cc:935: `ntree_limit` is deprecated, use `iteration_range` instead.
## [22:19:39] WARNING: src/c_api/c_api.cc:935: `ntree_limit` is deprecated, use `iteration_range` instead.
## [22:19:39] WARNING: src/c_api/c_api.cc:935: `ntree_limit` is deprecated, use `iteration_range` instead.
## [22:19:39] WARNING: src/c_api/c_api.cc:935: `ntree_limit` is deprecated, use `iteration_range` instead.
## [22:19:39] WARNING: src/c_api/c_api.cc:935: `ntree_limit` is deprecated, use `iteration_range` instead.
## [22:19:39] WARNING: src/c_api/c_api.cc:935: `ntree_limit` is deprecated, use `iteration_range` instead.
## [22:19:40] WARNING: src/c_api/c_api.cc:935: `ntree_limit` is deprecated, use `iteration_range` instead.
## [22:19:40] WARNING: src/c_api/c_api.cc:935: `ntree_limit` is deprecated, use `iteration_range` instead.
## [22:19:40] WARNING: src/c_api/c_api.cc:935: `ntree_limit` is deprecated, use `iteration_range` instead.
## [22:19:40] WARNING: src/c_api/c_api.cc:935: `ntree_limit` is deprecated, use `iteration_range` instead.
## [22:19:40] WARNING: src/c_api/c_api.cc:935: `ntree_limit` is deprecated, use `iteration_range` instead.
## [22:19:40] WARNING: src/c_api/c_api.cc:935: `ntree_limit` is deprecated, use `iteration_range` instead.
## [22:19:40] WARNING: src/c_api/c_api.cc:935: `ntree_limit` is deprecated, use `iteration_range` instead.
## [22:19:40] WARNING: src/c_api/c_api.cc:935: `ntree_limit` is deprecated, use `iteration_range` instead.
## [22:19:41] WARNING: src/c_api/c_api.cc:935: `ntree_limit` is deprecated, use `iteration_range` instead.
## [22:19:41] WARNING: src/c_api/c_api.cc:935: `ntree_limit` is deprecated, use `iteration_range` instead.
## [22:19:41] WARNING: src/c_api/c_api.cc:935: `ntree_limit` is deprecated, use `iteration_range` instead.
## [22:19:41] WARNING: src/c_api/c_api.cc:935: `ntree_limit` is deprecated, use `iteration_range` instead.
## [22:19:41] WARNING: src/c_api/c_api.cc:935: `ntree_limit` is deprecated, use `iteration_range` instead.
## [22:19:41] WARNING: src/c_api/c_api.cc:935: `ntree_limit` is deprecated, use `iteration_range` instead.
## [22:19:42] WARNING: src/c_api/c_api.cc:935: `ntree_limit` is deprecated, use `iteration_range` instead.
## [22:19:42] WARNING: src/c_api/c_api.cc:935: `ntree_limit` is deprecated, use `iteration_range` instead.
## [22:19:42] WARNING: src/c_api/c_api.cc:935: `ntree_limit` is deprecated, use `iteration_range` instead.
## [22:19:42] WARNING: src/c_api/c_api.cc:935: `ntree_limit` is deprecated, use `iteration_range` instead.
## [22:19:43] WARNING: src/c_api/c_api.cc:935: `ntree_limit` is deprecated, use `iteration_range` instead.
## [22:19:43] WARNING: src/c_api/c_api.cc:935: `ntree_limit` is deprecated, use `iteration_range` instead.
## [22:19:43] WARNING: src/c_api/c_api.cc:935: `ntree_limit` is deprecated, use `iteration_range` instead.
## [22:19:43] WARNING: src/c_api/c_api.cc:935: `ntree_limit` is deprecated, use `iteration_range` instead.
## [22:19:44] WARNING: src/c_api/c_api.cc:935: `ntree_limit` is deprecated, use `iteration_range` instead.
## [22:19:44] WARNING: src/c_api/c_api.cc:935: `ntree_limit` is deprecated, use `iteration_range` instead.
## [22:19:44] WARNING: src/c_api/c_api.cc:935: `ntree_limit` is deprecated, use `iteration_range` instead.
## [22:19:44] WARNING: src/c_api/c_api.cc:935: `ntree_limit` is deprecated, use `iteration_range` instead.
## [22:19:45] WARNING: src/c_api/c_api.cc:935: `ntree_limit` is deprecated, use `iteration_range` instead.
## [22:19:45] WARNING: src/c_api/c_api.cc:935: `ntree_limit` is deprecated, use `iteration_range` instead.
## [22:19:45] WARNING: src/c_api/c_api.cc:935: `ntree_limit` is deprecated, use `iteration_range` instead.
## [22:19:45] WARNING: src/c_api/c_api.cc:935: `ntree_limit` is deprecated, use `iteration_range` instead.
## [22:19:46] WARNING: src/c_api/c_api.cc:935: `ntree_limit` is deprecated, use `iteration_range` instead.
## [22:19:46] WARNING: src/c_api/c_api.cc:935: `ntree_limit` is deprecated, use `iteration_range` instead.
## [22:19:46] WARNING: src/c_api/c_api.cc:935: `ntree_limit` is deprecated, use `iteration_range` instead.
## [22:19:46] WARNING: src/c_api/c_api.cc:935: `ntree_limit` is deprecated, use `iteration_range` instead.
## [22:19:47] WARNING: src/c_api/c_api.cc:935: `ntree_limit` is deprecated, use `iteration_range` instead.
## [22:19:47] WARNING: src/c_api/c_api.cc:935: `ntree_limit` is deprecated, use `iteration_range` instead.
## [22:19:47] WARNING: src/c_api/c_api.cc:935: `ntree_limit` is deprecated, use `iteration_range` instead.
## [22:19:47] WARNING: src/c_api/c_api.cc:935: `ntree_limit` is deprecated, use `iteration_range` instead.
## [22:19:47] WARNING: src/c_api/c_api.cc:935: `ntree_limit` is deprecated, use `iteration_range` instead.
## [22:19:47] WARNING: src/c_api/c_api.cc:935: `ntree_limit` is deprecated, use `iteration_range` instead.
## [22:19:47] WARNING: src/c_api/c_api.cc:935: `ntree_limit` is deprecated, use `iteration_range` instead.
## [22:19:47] WARNING: src/c_api/c_api.cc:935: `ntree_limit` is deprecated, use `iteration_range` instead.
## [22:19:48] WARNING: src/c_api/c_api.cc:935: `ntree_limit` is deprecated, use `iteration_range` instead.
## [22:19:48] WARNING: src/c_api/c_api.cc:935: `ntree_limit` is deprecated, use `iteration_range` instead.
## [22:19:48] WARNING: src/c_api/c_api.cc:935: `ntree_limit` is deprecated, use `iteration_range` instead.
## [22:19:48] WARNING: src/c_api/c_api.cc:935: `ntree_limit` is deprecated, use `iteration_range` instead.
## [22:19:48] WARNING: src/c_api/c_api.cc:935: `ntree_limit` is deprecated, use `iteration_range` instead.
## [22:19:48] WARNING: src/c_api/c_api.cc:935: `ntree_limit` is deprecated, use `iteration_range` instead.
## [22:19:49] WARNING: src/c_api/c_api.cc:935: `ntree_limit` is deprecated, use `iteration_range` instead.
## [22:19:49] WARNING: src/c_api/c_api.cc:935: `ntree_limit` is deprecated, use `iteration_range` instead.
## [22:19:49] WARNING: src/c_api/c_api.cc:935: `ntree_limit` is deprecated, use `iteration_range` instead.
## [22:19:49] WARNING: src/c_api/c_api.cc:935: `ntree_limit` is deprecated, use `iteration_range` instead.
## [22:19:49] WARNING: src/c_api/c_api.cc:935: `ntree_limit` is deprecated, use `iteration_range` instead.
## [22:19:49] WARNING: src/c_api/c_api.cc:935: `ntree_limit` is deprecated, use `iteration_range` instead.
## [22:19:50] WARNING: src/c_api/c_api.cc:935: `ntree_limit` is deprecated, use `iteration_range` instead.
## [22:19:50] WARNING: src/c_api/c_api.cc:935: `ntree_limit` is deprecated, use `iteration_range` instead.
## [22:19:50] WARNING: src/c_api/c_api.cc:935: `ntree_limit` is deprecated, use `iteration_range` instead.
## [22:19:50] WARNING: src/c_api/c_api.cc:935: `ntree_limit` is deprecated, use `iteration_range` instead.
## [22:19:51] WARNING: src/c_api/c_api.cc:935: `ntree_limit` is deprecated, use `iteration_range` instead.
## [22:19:51] WARNING: src/c_api/c_api.cc:935: `ntree_limit` is deprecated, use `iteration_range` instead.
## [22:19:51] WARNING: src/c_api/c_api.cc:935: `ntree_limit` is deprecated, use `iteration_range` instead.
## [22:19:51] WARNING: src/c_api/c_api.cc:935: `ntree_limit` is deprecated, use `iteration_range` instead.
## [22:19:52] WARNING: src/c_api/c_api.cc:935: `ntree_limit` is deprecated, use `iteration_range` instead.
## [22:19:52] WARNING: src/c_api/c_api.cc:935: `ntree_limit` is deprecated, use `iteration_range` instead.
## [22:19:52] WARNING: src/c_api/c_api.cc:935: `ntree_limit` is deprecated, use `iteration_range` instead.
## [22:19:52] WARNING: src/c_api/c_api.cc:935: `ntree_limit` is deprecated, use `iteration_range` instead.
## [22:19:53] WARNING: src/c_api/c_api.cc:935: `ntree_limit` is deprecated, use `iteration_range` instead.
## [22:19:53] WARNING: src/c_api/c_api.cc:935: `ntree_limit` is deprecated, use `iteration_range` instead.
## [22:19:54] WARNING: src/c_api/c_api.cc:935: `ntree_limit` is deprecated, use `iteration_range` instead.
## [22:19:54] WARNING: src/c_api/c_api.cc:935: `ntree_limit` is deprecated, use `iteration_range` instead.
## [22:19:54] WARNING: src/c_api/c_api.cc:935: `ntree_limit` is deprecated, use `iteration_range` instead.
## [22:19:54] WARNING: src/c_api/c_api.cc:935: `ntree_limit` is deprecated, use `iteration_range` instead.
## [22:19:54] WARNING: src/c_api/c_api.cc:935: `ntree_limit` is deprecated, use `iteration_range` instead.
## [22:19:54] WARNING: src/c_api/c_api.cc:935: `ntree_limit` is deprecated, use `iteration_range` instead.
## [22:19:54] WARNING: src/c_api/c_api.cc:935: `ntree_limit` is deprecated, use `iteration_range` instead.
## [22:19:54] WARNING: src/c_api/c_api.cc:935: `ntree_limit` is deprecated, use `iteration_range` instead.
## [22:19:55] WARNING: src/c_api/c_api.cc:935: `ntree_limit` is deprecated, use `iteration_range` instead.
## [22:19:55] WARNING: src/c_api/c_api.cc:935: `ntree_limit` is deprecated, use `iteration_range` instead.
## [22:19:55] WARNING: src/c_api/c_api.cc:935: `ntree_limit` is deprecated, use `iteration_range` instead.
## [22:19:55] WARNING: src/c_api/c_api.cc:935: `ntree_limit` is deprecated, use `iteration_range` instead.
## [22:19:55] WARNING: src/c_api/c_api.cc:935: `ntree_limit` is deprecated, use `iteration_range` instead.
## [22:19:55] WARNING: src/c_api/c_api.cc:935: `ntree_limit` is deprecated, use `iteration_range` instead.
## [22:19:55] WARNING: src/c_api/c_api.cc:935: `ntree_limit` is deprecated, use `iteration_range` instead.
## [22:19:55] WARNING: src/c_api/c_api.cc:935: `ntree_limit` is deprecated, use `iteration_range` instead.
## [22:19:56] WARNING: src/c_api/c_api.cc:935: `ntree_limit` is deprecated, use `iteration_range` instead.
## [22:19:56] WARNING: src/c_api/c_api.cc:935: `ntree_limit` is deprecated, use `iteration_range` instead.
## [22:19:56] WARNING: src/c_api/c_api.cc:935: `ntree_limit` is deprecated, use `iteration_range` instead.
## [22:19:56] WARNING: src/c_api/c_api.cc:935: `ntree_limit` is deprecated, use `iteration_range` instead.
## [22:19:57] WARNING: src/c_api/c_api.cc:935: `ntree_limit` is deprecated, use `iteration_range` instead.
## [22:19:57] WARNING: src/c_api/c_api.cc:935: `ntree_limit` is deprecated, use `iteration_range` instead.
## [22:19:57] WARNING: src/c_api/c_api.cc:935: `ntree_limit` is deprecated, use `iteration_range` instead.
## [22:19:57] WARNING: src/c_api/c_api.cc:935: `ntree_limit` is deprecated, use `iteration_range` instead.
## [22:19:57] WARNING: src/c_api/c_api.cc:935: `ntree_limit` is deprecated, use `iteration_range` instead.
## [22:19:57] WARNING: src/c_api/c_api.cc:935: `ntree_limit` is deprecated, use `iteration_range` instead.
## [22:19:58] WARNING: src/c_api/c_api.cc:935: `ntree_limit` is deprecated, use `iteration_range` instead.
## [22:19:58] WARNING: src/c_api/c_api.cc:935: `ntree_limit` is deprecated, use `iteration_range` instead.
## [22:19:59] WARNING: src/c_api/c_api.cc:935: `ntree_limit` is deprecated, use `iteration_range` instead.
## [22:19:59] WARNING: src/c_api/c_api.cc:935: `ntree_limit` is deprecated, use `iteration_range` instead.
## [22:19:59] WARNING: src/c_api/c_api.cc:935: `ntree_limit` is deprecated, use `iteration_range` instead.
## [22:19:59] WARNING: src/c_api/c_api.cc:935: `ntree_limit` is deprecated, use `iteration_range` instead.
## [22:20:00] WARNING: src/c_api/c_api.cc:935: `ntree_limit` is deprecated, use `iteration_range` instead.
## [22:20:00] WARNING: src/c_api/c_api.cc:935: `ntree_limit` is deprecated, use `iteration_range` instead.
## [22:20:00] WARNING: src/c_api/c_api.cc:935: `ntree_limit` is deprecated, use `iteration_range` instead.
## [22:20:00] WARNING: src/c_api/c_api.cc:935: `ntree_limit` is deprecated, use `iteration_range` instead.
## [22:20:01] WARNING: src/c_api/c_api.cc:935: `ntree_limit` is deprecated, use `iteration_range` instead.
## [22:20:01] WARNING: src/c_api/c_api.cc:935: `ntree_limit` is deprecated, use `iteration_range` instead.
## [22:20:01] WARNING: src/c_api/c_api.cc:935: `ntree_limit` is deprecated, use `iteration_range` instead.
## [22:20:01] WARNING: src/c_api/c_api.cc:935: `ntree_limit` is deprecated, use `iteration_range` instead.
## [22:20:01] WARNING: src/c_api/c_api.cc:935: `ntree_limit` is deprecated, use `iteration_range` instead.
## [22:20:01] WARNING: src/c_api/c_api.cc:935: `ntree_limit` is deprecated, use `iteration_range` instead.
## [22:20:02] WARNING: src/c_api/c_api.cc:935: `ntree_limit` is deprecated, use `iteration_range` instead.
## [22:20:02] WARNING: src/c_api/c_api.cc:935: `ntree_limit` is deprecated, use `iteration_range` instead.
## [22:20:02] WARNING: src/c_api/c_api.cc:935: `ntree_limit` is deprecated, use `iteration_range` instead.
## [22:20:02] WARNING: src/c_api/c_api.cc:935: `ntree_limit` is deprecated, use `iteration_range` instead.
## [22:20:02] WARNING: src/c_api/c_api.cc:935: `ntree_limit` is deprecated, use `iteration_range` instead.
## [22:20:02] WARNING: src/c_api/c_api.cc:935: `ntree_limit` is deprecated, use `iteration_range` instead.
## [22:20:02] WARNING: src/c_api/c_api.cc:935: `ntree_limit` is deprecated, use `iteration_range` instead.
## [22:20:02] WARNING: src/c_api/c_api.cc:935: `ntree_limit` is deprecated, use `iteration_range` instead.
## [22:20:03] WARNING: src/c_api/c_api.cc:935: `ntree_limit` is deprecated, use `iteration_range` instead.
## [22:20:03] WARNING: src/c_api/c_api.cc:935: `ntree_limit` is deprecated, use `iteration_range` instead.
## [22:20:03] WARNING: src/c_api/c_api.cc:935: `ntree_limit` is deprecated, use `iteration_range` instead.
## [22:20:03] WARNING: src/c_api/c_api.cc:935: `ntree_limit` is deprecated, use `iteration_range` instead.
## [22:20:04] WARNING: src/c_api/c_api.cc:935: `ntree_limit` is deprecated, use `iteration_range` instead.
## [22:20:04] WARNING: src/c_api/c_api.cc:935: `ntree_limit` is deprecated, use `iteration_range` instead.
## [22:20:04] WARNING: src/c_api/c_api.cc:935: `ntree_limit` is deprecated, use `iteration_range` instead.
## [22:20:04] WARNING: src/c_api/c_api.cc:935: `ntree_limit` is deprecated, use `iteration_range` instead.
## [22:20:04] WARNING: src/c_api/c_api.cc:935: `ntree_limit` is deprecated, use `iteration_range` instead.
## [22:20:04] WARNING: src/c_api/c_api.cc:935: `ntree_limit` is deprecated, use `iteration_range` instead.
## [22:20:05] WARNING: src/c_api/c_api.cc:935: `ntree_limit` is deprecated, use `iteration_range` instead.
## [22:20:05] WARNING: src/c_api/c_api.cc:935: `ntree_limit` is deprecated, use `iteration_range` instead.
## [22:20:05] WARNING: src/c_api/c_api.cc:935: `ntree_limit` is deprecated, use `iteration_range` instead.
## [22:20:05] WARNING: src/c_api/c_api.cc:935: `ntree_limit` is deprecated, use `iteration_range` instead.
## [22:20:06] WARNING: src/c_api/c_api.cc:935: `ntree_limit` is deprecated, use `iteration_range` instead.
## [22:20:06] WARNING: src/c_api/c_api.cc:935: `ntree_limit` is deprecated, use `iteration_range` instead.
## [22:20:07] WARNING: src/c_api/c_api.cc:935: `ntree_limit` is deprecated, use `iteration_range` instead.
## [22:20:07] WARNING: src/c_api/c_api.cc:935: `ntree_limit` is deprecated, use `iteration_range` instead.
## [22:20:07] WARNING: src/c_api/c_api.cc:935: `ntree_limit` is deprecated, use `iteration_range` instead.
## [22:20:07] WARNING: src/c_api/c_api.cc:935: `ntree_limit` is deprecated, use `iteration_range` instead.
## [22:20:08] WARNING: src/c_api/c_api.cc:935: `ntree_limit` is deprecated, use `iteration_range` instead.
## [22:20:08] WARNING: src/c_api/c_api.cc:935: `ntree_limit` is deprecated, use `iteration_range` instead.
## [22:20:08] WARNING: src/c_api/c_api.cc:935: `ntree_limit` is deprecated, use `iteration_range` instead.
## [22:20:08] WARNING: src/c_api/c_api.cc:935: `ntree_limit` is deprecated, use `iteration_range` instead.
## [22:20:09] WARNING: src/c_api/c_api.cc:935: `ntree_limit` is deprecated, use `iteration_range` instead.
## [22:20:09] WARNING: src/c_api/c_api.cc:935: `ntree_limit` is deprecated, use `iteration_range` instead.
## [22:20:09] WARNING: src/c_api/c_api.cc:935: `ntree_limit` is deprecated, use `iteration_range` instead.
## [22:20:09] WARNING: src/c_api/c_api.cc:935: `ntree_limit` is deprecated, use `iteration_range` instead.
## [22:20:09] WARNING: src/c_api/c_api.cc:935: `ntree_limit` is deprecated, use `iteration_range` instead.
## [22:20:09] WARNING: src/c_api/c_api.cc:935: `ntree_limit` is deprecated, use `iteration_range` instead.
## [22:20:09] WARNING: src/c_api/c_api.cc:935: `ntree_limit` is deprecated, use `iteration_range` instead.
## [22:20:09] WARNING: src/c_api/c_api.cc:935: `ntree_limit` is deprecated, use `iteration_range` instead.
## [22:20:10] WARNING: src/c_api/c_api.cc:935: `ntree_limit` is deprecated, use `iteration_range` instead.
## [22:20:10] WARNING: src/c_api/c_api.cc:935: `ntree_limit` is deprecated, use `iteration_range` instead.
## [22:20:10] WARNING: src/c_api/c_api.cc:935: `ntree_limit` is deprecated, use `iteration_range` instead.
## [22:20:10] WARNING: src/c_api/c_api.cc:935: `ntree_limit` is deprecated, use `iteration_range` instead.
## [22:20:10] WARNING: src/c_api/c_api.cc:935: `ntree_limit` is deprecated, use `iteration_range` instead.
## [22:20:10] WARNING: src/c_api/c_api.cc:935: `ntree_limit` is deprecated, use `iteration_range` instead.
## [22:20:11] WARNING: src/c_api/c_api.cc:935: `ntree_limit` is deprecated, use `iteration_range` instead.
## [22:20:11] WARNING: src/c_api/c_api.cc:935: `ntree_limit` is deprecated, use `iteration_range` instead.
## [22:20:11] WARNING: src/c_api/c_api.cc:935: `ntree_limit` is deprecated, use `iteration_range` instead.
## [22:20:11] WARNING: src/c_api/c_api.cc:935: `ntree_limit` is deprecated, use `iteration_range` instead.
## [22:20:11] WARNING: src/c_api/c_api.cc:935: `ntree_limit` is deprecated, use `iteration_range` instead.
## [22:20:11] WARNING: src/c_api/c_api.cc:935: `ntree_limit` is deprecated, use `iteration_range` instead.
## [22:20:12] WARNING: src/c_api/c_api.cc:935: `ntree_limit` is deprecated, use `iteration_range` instead.
## [22:20:12] WARNING: src/c_api/c_api.cc:935: `ntree_limit` is deprecated, use `iteration_range` instead.
## [22:20:12] WARNING: src/c_api/c_api.cc:935: `ntree_limit` is deprecated, use `iteration_range` instead.
## [22:20:12] WARNING: src/c_api/c_api.cc:935: `ntree_limit` is deprecated, use `iteration_range` instead.
## [22:20:13] WARNING: src/c_api/c_api.cc:935: `ntree_limit` is deprecated, use `iteration_range` instead.
## [22:20:13] WARNING: src/c_api/c_api.cc:935: `ntree_limit` is deprecated, use `iteration_range` instead.
## [22:20:13] WARNING: src/c_api/c_api.cc:935: `ntree_limit` is deprecated, use `iteration_range` instead.
## [22:20:13] WARNING: src/c_api/c_api.cc:935: `ntree_limit` is deprecated, use `iteration_range` instead.
## [22:20:14] WARNING: src/c_api/c_api.cc:935: `ntree_limit` is deprecated, use `iteration_range` instead.
## [22:20:14] WARNING: src/c_api/c_api.cc:935: `ntree_limit` is deprecated, use `iteration_range` instead.
## [22:20:15] WARNING: src/c_api/c_api.cc:935: `ntree_limit` is deprecated, use `iteration_range` instead.
## [22:20:15] WARNING: src/c_api/c_api.cc:935: `ntree_limit` is deprecated, use `iteration_range` instead.
## [22:20:15] WARNING: src/c_api/c_api.cc:935: `ntree_limit` is deprecated, use `iteration_range` instead.
## [22:20:15] WARNING: src/c_api/c_api.cc:935: `ntree_limit` is deprecated, use `iteration_range` instead.
## [22:20:16] WARNING: src/c_api/c_api.cc:935: `ntree_limit` is deprecated, use `iteration_range` instead.
## [22:20:16] WARNING: src/c_api/c_api.cc:935: `ntree_limit` is deprecated, use `iteration_range` instead.
## [22:20:16] WARNING: src/c_api/c_api.cc:935: `ntree_limit` is deprecated, use `iteration_range` instead.
## [22:20:16] WARNING: src/c_api/c_api.cc:935: `ntree_limit` is deprecated, use `iteration_range` instead.
## [22:20:16] WARNING: src/c_api/c_api.cc:935: `ntree_limit` is deprecated, use `iteration_range` instead.
## [22:20:16] WARNING: src/c_api/c_api.cc:935: `ntree_limit` is deprecated, use `iteration_range` instead.
## [22:20:16] WARNING: src/c_api/c_api.cc:935: `ntree_limit` is deprecated, use `iteration_range` instead.
## [22:20:16] WARNING: src/c_api/c_api.cc:935: `ntree_limit` is deprecated, use `iteration_range` instead.
## [22:20:17] WARNING: src/c_api/c_api.cc:935: `ntree_limit` is deprecated, use `iteration_range` instead.
## [22:20:17] WARNING: src/c_api/c_api.cc:935: `ntree_limit` is deprecated, use `iteration_range` instead.
## [22:20:17] WARNING: src/c_api/c_api.cc:935: `ntree_limit` is deprecated, use `iteration_range` instead.
## [22:20:17] WARNING: src/c_api/c_api.cc:935: `ntree_limit` is deprecated, use `iteration_range` instead.
## [22:20:17] WARNING: src/c_api/c_api.cc:935: `ntree_limit` is deprecated, use `iteration_range` instead.
## [22:20:17] WARNING: src/c_api/c_api.cc:935: `ntree_limit` is deprecated, use `iteration_range` instead.
## [22:20:18] WARNING: src/c_api/c_api.cc:935: `ntree_limit` is deprecated, use `iteration_range` instead.
## [22:20:18] WARNING: src/c_api/c_api.cc:935: `ntree_limit` is deprecated, use `iteration_range` instead.
## [22:20:18] WARNING: src/c_api/c_api.cc:935: `ntree_limit` is deprecated, use `iteration_range` instead.
## [22:20:18] WARNING: src/c_api/c_api.cc:935: `ntree_limit` is deprecated, use `iteration_range` instead.
## [22:20:18] WARNING: src/c_api/c_api.cc:935: `ntree_limit` is deprecated, use `iteration_range` instead.
## [22:20:18] WARNING: src/c_api/c_api.cc:935: `ntree_limit` is deprecated, use `iteration_range` instead.
## [22:20:19] WARNING: src/c_api/c_api.cc:935: `ntree_limit` is deprecated, use `iteration_range` instead.
## [22:20:19] WARNING: src/c_api/c_api.cc:935: `ntree_limit` is deprecated, use `iteration_range` instead.
## [22:20:19] WARNING: src/c_api/c_api.cc:935: `ntree_limit` is deprecated, use `iteration_range` instead.
## [22:20:19] WARNING: src/c_api/c_api.cc:935: `ntree_limit` is deprecated, use `iteration_range` instead.
## [22:20:20] WARNING: src/c_api/c_api.cc:935: `ntree_limit` is deprecated, use `iteration_range` instead.
## [22:20:20] WARNING: src/c_api/c_api.cc:935: `ntree_limit` is deprecated, use `iteration_range` instead.
## [22:20:20] WARNING: src/c_api/c_api.cc:935: `ntree_limit` is deprecated, use `iteration_range` instead.
## [22:20:20] WARNING: src/c_api/c_api.cc:935: `ntree_limit` is deprecated, use `iteration_range` instead.
## [22:20:21] WARNING: src/c_api/c_api.cc:935: `ntree_limit` is deprecated, use `iteration_range` instead.
## [22:20:21] WARNING: src/c_api/c_api.cc:935: `ntree_limit` is deprecated, use `iteration_range` instead.
## [22:20:22] WARNING: src/c_api/c_api.cc:935: `ntree_limit` is deprecated, use `iteration_range` instead.
## [22:20:22] WARNING: src/c_api/c_api.cc:935: `ntree_limit` is deprecated, use `iteration_range` instead.
## [22:20:22] WARNING: src/c_api/c_api.cc:935: `ntree_limit` is deprecated, use `iteration_range` instead.
## [22:20:22] WARNING: src/c_api/c_api.cc:935: `ntree_limit` is deprecated, use `iteration_range` instead.
## [22:20:23] WARNING: src/c_api/c_api.cc:935: `ntree_limit` is deprecated, use `iteration_range` instead.
## [22:20:23] WARNING: src/c_api/c_api.cc:935: `ntree_limit` is deprecated, use `iteration_range` instead.
## [22:20:23] WARNING: src/c_api/c_api.cc:935: `ntree_limit` is deprecated, use `iteration_range` instead.
## [22:20:23] WARNING: src/c_api/c_api.cc:935: `ntree_limit` is deprecated, use `iteration_range` instead.
## [22:20:24] WARNING: src/c_api/c_api.cc:935: `ntree_limit` is deprecated, use `iteration_range` instead.
## [22:20:24] WARNING: src/c_api/c_api.cc:935: `ntree_limit` is deprecated, use `iteration_range` instead.
## [22:20:24] WARNING: src/c_api/c_api.cc:935: `ntree_limit` is deprecated, use `iteration_range` instead.
## [22:20:24] WARNING: src/c_api/c_api.cc:935: `ntree_limit` is deprecated, use `iteration_range` instead.
## [22:20:24] WARNING: src/c_api/c_api.cc:935: `ntree_limit` is deprecated, use `iteration_range` instead.
## [22:20:24] WARNING: src/c_api/c_api.cc:935: `ntree_limit` is deprecated, use `iteration_range` instead.
## [22:20:24] WARNING: src/c_api/c_api.cc:935: `ntree_limit` is deprecated, use `iteration_range` instead.
## [22:20:24] WARNING: src/c_api/c_api.cc:935: `ntree_limit` is deprecated, use `iteration_range` instead.
## [22:20:25] WARNING: src/c_api/c_api.cc:935: `ntree_limit` is deprecated, use `iteration_range` instead.
## [22:20:25] WARNING: src/c_api/c_api.cc:935: `ntree_limit` is deprecated, use `iteration_range` instead.
## [22:20:25] WARNING: src/c_api/c_api.cc:935: `ntree_limit` is deprecated, use `iteration_range` instead.
## [22:20:25] WARNING: src/c_api/c_api.cc:935: `ntree_limit` is deprecated, use `iteration_range` instead.
## [22:20:25] WARNING: src/c_api/c_api.cc:935: `ntree_limit` is deprecated, use `iteration_range` instead.
## [22:20:25] WARNING: src/c_api/c_api.cc:935: `ntree_limit` is deprecated, use `iteration_range` instead.
## [22:20:26] WARNING: src/c_api/c_api.cc:935: `ntree_limit` is deprecated, use `iteration_range` instead.
## [22:20:26] WARNING: src/c_api/c_api.cc:935: `ntree_limit` is deprecated, use `iteration_range` instead.
## [22:20:26] WARNING: src/c_api/c_api.cc:935: `ntree_limit` is deprecated, use `iteration_range` instead.
## [22:20:26] WARNING: src/c_api/c_api.cc:935: `ntree_limit` is deprecated, use `iteration_range` instead.
## [22:20:27] WARNING: src/c_api/c_api.cc:935: `ntree_limit` is deprecated, use `iteration_range` instead.
## [22:20:27] WARNING: src/c_api/c_api.cc:935: `ntree_limit` is deprecated, use `iteration_range` instead.
## [22:20:27] WARNING: src/c_api/c_api.cc:935: `ntree_limit` is deprecated, use `iteration_range` instead.
## [22:20:27] WARNING: src/c_api/c_api.cc:935: `ntree_limit` is deprecated, use `iteration_range` instead.
## [22:20:27] WARNING: src/c_api/c_api.cc:935: `ntree_limit` is deprecated, use `iteration_range` instead.
## [22:20:27] WARNING: src/c_api/c_api.cc:935: `ntree_limit` is deprecated, use `iteration_range` instead.
## [22:20:28] WARNING: src/c_api/c_api.cc:935: `ntree_limit` is deprecated, use `iteration_range` instead.
## [22:20:28] WARNING: src/c_api/c_api.cc:935: `ntree_limit` is deprecated, use `iteration_range` instead.
## [22:20:29] WARNING: src/c_api/c_api.cc:935: `ntree_limit` is deprecated, use `iteration_range` instead.
## [22:20:29] WARNING: src/c_api/c_api.cc:935: `ntree_limit` is deprecated, use `iteration_range` instead.
## [22:20:29] WARNING: src/c_api/c_api.cc:935: `ntree_limit` is deprecated, use `iteration_range` instead.
## [22:20:29] WARNING: src/c_api/c_api.cc:935: `ntree_limit` is deprecated, use `iteration_range` instead.
## [22:20:30] WARNING: src/c_api/c_api.cc:935: `ntree_limit` is deprecated, use `iteration_range` instead.
## [22:20:30] WARNING: src/c_api/c_api.cc:935: `ntree_limit` is deprecated, use `iteration_range` instead.
## [22:20:30] WARNING: src/c_api/c_api.cc:935: `ntree_limit` is deprecated, use `iteration_range` instead.
## [22:20:30] WARNING: src/c_api/c_api.cc:935: `ntree_limit` is deprecated, use `iteration_range` instead.
## [22:20:31] WARNING: src/c_api/c_api.cc:935: `ntree_limit` is deprecated, use `iteration_range` instead.
## [22:20:31] WARNING: src/c_api/c_api.cc:935: `ntree_limit` is deprecated, use `iteration_range` instead.
## [22:20:31] WARNING: src/c_api/c_api.cc:935: `ntree_limit` is deprecated, use `iteration_range` instead.
## [22:20:31] WARNING: src/c_api/c_api.cc:935: `ntree_limit` is deprecated, use `iteration_range` instead.
## [22:20:32] WARNING: src/c_api/c_api.cc:935: `ntree_limit` is deprecated, use `iteration_range` instead.
## [22:20:32] WARNING: src/c_api/c_api.cc:935: `ntree_limit` is deprecated, use `iteration_range` instead.
## [22:20:32] WARNING: src/c_api/c_api.cc:935: `ntree_limit` is deprecated, use `iteration_range` instead.
## [22:20:32] WARNING: src/c_api/c_api.cc:935: `ntree_limit` is deprecated, use `iteration_range` instead.
## [22:20:32] WARNING: src/c_api/c_api.cc:935: `ntree_limit` is deprecated, use `iteration_range` instead.
## [22:20:32] WARNING: src/c_api/c_api.cc:935: `ntree_limit` is deprecated, use `iteration_range` instead.
## [22:20:32] WARNING: src/c_api/c_api.cc:935: `ntree_limit` is deprecated, use `iteration_range` instead.
## [22:20:32] WARNING: src/c_api/c_api.cc:935: `ntree_limit` is deprecated, use `iteration_range` instead.
## [22:20:33] WARNING: src/c_api/c_api.cc:935: `ntree_limit` is deprecated, use `iteration_range` instead.
## [22:20:33] WARNING: src/c_api/c_api.cc:935: `ntree_limit` is deprecated, use `iteration_range` instead.
## [22:20:33] WARNING: src/c_api/c_api.cc:935: `ntree_limit` is deprecated, use `iteration_range` instead.
## [22:20:33] WARNING: src/c_api/c_api.cc:935: `ntree_limit` is deprecated, use `iteration_range` instead.
## [22:20:33] WARNING: src/c_api/c_api.cc:935: `ntree_limit` is deprecated, use `iteration_range` instead.
## [22:20:33] WARNING: src/c_api/c_api.cc:935: `ntree_limit` is deprecated, use `iteration_range` instead.
## [22:20:34] WARNING: src/c_api/c_api.cc:935: `ntree_limit` is deprecated, use `iteration_range` instead.
## [22:20:34] WARNING: src/c_api/c_api.cc:935: `ntree_limit` is deprecated, use `iteration_range` instead.
## [22:20:34] WARNING: src/c_api/c_api.cc:935: `ntree_limit` is deprecated, use `iteration_range` instead.
## [22:20:34] WARNING: src/c_api/c_api.cc:935: `ntree_limit` is deprecated, use `iteration_range` instead.
## [22:20:35] WARNING: src/c_api/c_api.cc:935: `ntree_limit` is deprecated, use `iteration_range` instead.
## [22:20:35] WARNING: src/c_api/c_api.cc:935: `ntree_limit` is deprecated, use `iteration_range` instead.
## [22:20:35] WARNING: src/c_api/c_api.cc:935: `ntree_limit` is deprecated, use `iteration_range` instead.
## [22:20:35] WARNING: src/c_api/c_api.cc:935: `ntree_limit` is deprecated, use `iteration_range` instead.
## [22:20:36] WARNING: src/c_api/c_api.cc:935: `ntree_limit` is deprecated, use `iteration_range` instead.
## [22:20:36] WARNING: src/c_api/c_api.cc:935: `ntree_limit` is deprecated, use `iteration_range` instead.
## [22:20:36] WARNING: src/c_api/c_api.cc:935: `ntree_limit` is deprecated, use `iteration_range` instead.
## [22:20:36] WARNING: src/c_api/c_api.cc:935: `ntree_limit` is deprecated, use `iteration_range` instead.
## [22:20:37] WARNING: src/c_api/c_api.cc:935: `ntree_limit` is deprecated, use `iteration_range` instead.
## [22:20:37] WARNING: src/c_api/c_api.cc:935: `ntree_limit` is deprecated, use `iteration_range` instead.
## [22:20:37] WARNING: src/c_api/c_api.cc:935: `ntree_limit` is deprecated, use `iteration_range` instead.
## [22:20:37] WARNING: src/c_api/c_api.cc:935: `ntree_limit` is deprecated, use `iteration_range` instead.
## [22:20:38] WARNING: src/c_api/c_api.cc:935: `ntree_limit` is deprecated, use `iteration_range` instead.
## [22:20:38] WARNING: src/c_api/c_api.cc:935: `ntree_limit` is deprecated, use `iteration_range` instead.
## [22:20:39] WARNING: src/c_api/c_api.cc:935: `ntree_limit` is deprecated, use `iteration_range` instead.
## [22:20:39] WARNING: src/c_api/c_api.cc:935: `ntree_limit` is deprecated, use `iteration_range` instead.
## [22:20:39] WARNING: src/c_api/c_api.cc:935: `ntree_limit` is deprecated, use `iteration_range` instead.
## [22:20:39] WARNING: src/c_api/c_api.cc:935: `ntree_limit` is deprecated, use `iteration_range` instead.
## [22:20:39] WARNING: src/c_api/c_api.cc:935: `ntree_limit` is deprecated, use `iteration_range` instead.
## [22:20:39] WARNING: src/c_api/c_api.cc:935: `ntree_limit` is deprecated, use `iteration_range` instead.
## [22:20:39] WARNING: src/c_api/c_api.cc:935: `ntree_limit` is deprecated, use `iteration_range` instead.
## [22:20:39] WARNING: src/c_api/c_api.cc:935: `ntree_limit` is deprecated, use `iteration_range` instead.
## [22:20:40] WARNING: src/c_api/c_api.cc:935: `ntree_limit` is deprecated, use `iteration_range` instead.
## [22:20:40] WARNING: src/c_api/c_api.cc:935: `ntree_limit` is deprecated, use `iteration_range` instead.
## [22:20:40] WARNING: src/c_api/c_api.cc:935: `ntree_limit` is deprecated, use `iteration_range` instead.
## [22:20:40] WARNING: src/c_api/c_api.cc:935: `ntree_limit` is deprecated, use `iteration_range` instead.
## [22:20:40] WARNING: src/c_api/c_api.cc:935: `ntree_limit` is deprecated, use `iteration_range` instead.
## [22:20:40] WARNING: src/c_api/c_api.cc:935: `ntree_limit` is deprecated, use `iteration_range` instead.
## [22:20:41] WARNING: src/c_api/c_api.cc:935: `ntree_limit` is deprecated, use `iteration_range` instead.
## [22:20:41] WARNING: src/c_api/c_api.cc:935: `ntree_limit` is deprecated, use `iteration_range` instead.
## [22:20:41] WARNING: src/c_api/c_api.cc:935: `ntree_limit` is deprecated, use `iteration_range` instead.
## [22:20:41] WARNING: src/c_api/c_api.cc:935: `ntree_limit` is deprecated, use `iteration_range` instead.
## [22:20:41] WARNING: src/c_api/c_api.cc:935: `ntree_limit` is deprecated, use `iteration_range` instead.
## [22:20:41] WARNING: src/c_api/c_api.cc:935: `ntree_limit` is deprecated, use `iteration_range` instead.
## [22:20:42] WARNING: src/c_api/c_api.cc:935: `ntree_limit` is deprecated, use `iteration_range` instead.
## [22:20:42] WARNING: src/c_api/c_api.cc:935: `ntree_limit` is deprecated, use `iteration_range` instead.
## [22:20:42] WARNING: src/c_api/c_api.cc:935: `ntree_limit` is deprecated, use `iteration_range` instead.
## [22:20:42] WARNING: src/c_api/c_api.cc:935: `ntree_limit` is deprecated, use `iteration_range` instead.
## [22:20:43] WARNING: src/c_api/c_api.cc:935: `ntree_limit` is deprecated, use `iteration_range` instead.
## [22:20:43] WARNING: src/c_api/c_api.cc:935: `ntree_limit` is deprecated, use `iteration_range` instead.
## [22:20:43] WARNING: src/c_api/c_api.cc:935: `ntree_limit` is deprecated, use `iteration_range` instead.
## [22:20:43] WARNING: src/c_api/c_api.cc:935: `ntree_limit` is deprecated, use `iteration_range` instead.
## [22:20:44] WARNING: src/c_api/c_api.cc:935: `ntree_limit` is deprecated, use `iteration_range` instead.
## [22:20:44] WARNING: src/c_api/c_api.cc:935: `ntree_limit` is deprecated, use `iteration_range` instead.
## [22:20:44] WARNING: src/c_api/c_api.cc:935: `ntree_limit` is deprecated, use `iteration_range` instead.
## [22:20:44] WARNING: src/c_api/c_api.cc:935: `ntree_limit` is deprecated, use `iteration_range` instead.
## [22:20:45] WARNING: src/c_api/c_api.cc:935: `ntree_limit` is deprecated, use `iteration_range` instead.
## [22:20:45] WARNING: src/c_api/c_api.cc:935: `ntree_limit` is deprecated, use `iteration_range` instead.
## [22:20:46] WARNING: src/c_api/c_api.cc:935: `ntree_limit` is deprecated, use `iteration_range` instead.
## [22:20:46] WARNING: src/c_api/c_api.cc:935: `ntree_limit` is deprecated, use `iteration_range` instead.
## [22:20:46] WARNING: src/c_api/c_api.cc:935: `ntree_limit` is deprecated, use `iteration_range` instead.
## [22:20:46] WARNING: src/c_api/c_api.cc:935: `ntree_limit` is deprecated, use `iteration_range` instead.
## [22:20:47] WARNING: src/c_api/c_api.cc:935: `ntree_limit` is deprecated, use `iteration_range` instead.
## [22:20:47] WARNING: src/c_api/c_api.cc:935: `ntree_limit` is deprecated, use `iteration_range` instead.
## [22:20:47] WARNING: src/c_api/c_api.cc:935: `ntree_limit` is deprecated, use `iteration_range` instead.
## [22:20:47] WARNING: src/c_api/c_api.cc:935: `ntree_limit` is deprecated, use `iteration_range` instead.
## [22:20:47] WARNING: src/c_api/c_api.cc:935: `ntree_limit` is deprecated, use `iteration_range` instead.
## [22:20:47] WARNING: src/c_api/c_api.cc:935: `ntree_limit` is deprecated, use `iteration_range` instead.
## [22:20:47] WARNING: src/c_api/c_api.cc:935: `ntree_limit` is deprecated, use `iteration_range` instead.
## [22:20:47] WARNING: src/c_api/c_api.cc:935: `ntree_limit` is deprecated, use `iteration_range` instead.
## [22:20:48] WARNING: src/c_api/c_api.cc:935: `ntree_limit` is deprecated, use `iteration_range` instead.
## [22:20:48] WARNING: src/c_api/c_api.cc:935: `ntree_limit` is deprecated, use `iteration_range` instead.
## [22:20:48] WARNING: src/c_api/c_api.cc:935: `ntree_limit` is deprecated, use `iteration_range` instead.
## [22:20:48] WARNING: src/c_api/c_api.cc:935: `ntree_limit` is deprecated, use `iteration_range` instead.
## [22:20:48] WARNING: src/c_api/c_api.cc:935: `ntree_limit` is deprecated, use `iteration_range` instead.
## [22:20:48] WARNING: src/c_api/c_api.cc:935: `ntree_limit` is deprecated, use `iteration_range` instead.
## [22:20:49] WARNING: src/c_api/c_api.cc:935: `ntree_limit` is deprecated, use `iteration_range` instead.
## [22:20:49] WARNING: src/c_api/c_api.cc:935: `ntree_limit` is deprecated, use `iteration_range` instead.
## [22:20:49] WARNING: src/c_api/c_api.cc:935: `ntree_limit` is deprecated, use `iteration_range` instead.
## [22:20:49] WARNING: src/c_api/c_api.cc:935: `ntree_limit` is deprecated, use `iteration_range` instead.
## [22:20:50] WARNING: src/c_api/c_api.cc:935: `ntree_limit` is deprecated, use `iteration_range` instead.
## [22:20:50] WARNING: src/c_api/c_api.cc:935: `ntree_limit` is deprecated, use `iteration_range` instead.
## [22:20:50] WARNING: src/c_api/c_api.cc:935: `ntree_limit` is deprecated, use `iteration_range` instead.
## [22:20:50] WARNING: src/c_api/c_api.cc:935: `ntree_limit` is deprecated, use `iteration_range` instead.
## [22:20:51] WARNING: src/c_api/c_api.cc:935: `ntree_limit` is deprecated, use `iteration_range` instead.
## [22:20:51] WARNING: src/c_api/c_api.cc:935: `ntree_limit` is deprecated, use `iteration_range` instead.
## [22:20:51] WARNING: src/c_api/c_api.cc:935: `ntree_limit` is deprecated, use `iteration_range` instead.
## [22:20:51] WARNING: src/c_api/c_api.cc:935: `ntree_limit` is deprecated, use `iteration_range` instead.
## [22:20:52] WARNING: src/c_api/c_api.cc:935: `ntree_limit` is deprecated, use `iteration_range` instead.
## [22:20:52] WARNING: src/c_api/c_api.cc:935: `ntree_limit` is deprecated, use `iteration_range` instead.
## [22:20:52] WARNING: src/c_api/c_api.cc:935: `ntree_limit` is deprecated, use `iteration_range` instead.
## [22:20:52] WARNING: src/c_api/c_api.cc:935: `ntree_limit` is deprecated, use `iteration_range` instead.
## [22:20:53] WARNING: src/c_api/c_api.cc:935: `ntree_limit` is deprecated, use `iteration_range` instead.
## [22:20:53] WARNING: src/c_api/c_api.cc:935: `ntree_limit` is deprecated, use `iteration_range` instead.
## [22:20:54] WARNING: src/c_api/c_api.cc:935: `ntree_limit` is deprecated, use `iteration_range` instead.
## [22:20:54] WARNING: src/c_api/c_api.cc:935: `ntree_limit` is deprecated, use `iteration_range` instead.
## [22:20:55] WARNING: src/c_api/c_api.cc:935: `ntree_limit` is deprecated, use `iteration_range` instead.
## [22:20:55] WARNING: src/c_api/c_api.cc:935: `ntree_limit` is deprecated, use `iteration_range` instead.
## [22:20:55] WARNING: src/c_api/c_api.cc:935: `ntree_limit` is deprecated, use `iteration_range` instead.
## [22:20:55] WARNING: src/c_api/c_api.cc:935: `ntree_limit` is deprecated, use `iteration_range` instead.
## [22:20:55] WARNING: src/c_api/c_api.cc:935: `ntree_limit` is deprecated, use `iteration_range` instead.
## [22:20:55] WARNING: src/c_api/c_api.cc:935: `ntree_limit` is deprecated, use `iteration_range` instead.
## [22:20:56] WARNING: src/c_api/c_api.cc:935: `ntree_limit` is deprecated, use `iteration_range` instead.
## [22:20:56] WARNING: src/c_api/c_api.cc:935: `ntree_limit` is deprecated, use `iteration_range` instead.
## [22:20:56] WARNING: src/c_api/c_api.cc:935: `ntree_limit` is deprecated, use `iteration_range` instead.
## [22:20:56] WARNING: src/c_api/c_api.cc:935: `ntree_limit` is deprecated, use `iteration_range` instead.
## [22:20:56] WARNING: src/c_api/c_api.cc:935: `ntree_limit` is deprecated, use `iteration_range` instead.
## [22:20:56] WARNING: src/c_api/c_api.cc:935: `ntree_limit` is deprecated, use `iteration_range` instead.
## [22:20:56] WARNING: src/c_api/c_api.cc:935: `ntree_limit` is deprecated, use `iteration_range` instead.
## [22:20:56] WARNING: src/c_api/c_api.cc:935: `ntree_limit` is deprecated, use `iteration_range` instead.
## [22:20:57] WARNING: src/c_api/c_api.cc:935: `ntree_limit` is deprecated, use `iteration_range` instead.
## [22:20:57] WARNING: src/c_api/c_api.cc:935: `ntree_limit` is deprecated, use `iteration_range` instead.
## [22:20:57] WARNING: src/c_api/c_api.cc:935: `ntree_limit` is deprecated, use `iteration_range` instead.
## [22:20:57] WARNING: src/c_api/c_api.cc:935: `ntree_limit` is deprecated, use `iteration_range` instead.
## [22:20:58] WARNING: src/c_api/c_api.cc:935: `ntree_limit` is deprecated, use `iteration_range` instead.
## [22:20:58] WARNING: src/c_api/c_api.cc:935: `ntree_limit` is deprecated, use `iteration_range` instead.
## [22:20:58] WARNING: src/c_api/c_api.cc:935: `ntree_limit` is deprecated, use `iteration_range` instead.
## [22:20:58] WARNING: src/c_api/c_api.cc:935: `ntree_limit` is deprecated, use `iteration_range` instead.
## [22:20:59] WARNING: src/c_api/c_api.cc:935: `ntree_limit` is deprecated, use `iteration_range` instead.
## [22:20:59] WARNING: src/c_api/c_api.cc:935: `ntree_limit` is deprecated, use `iteration_range` instead.
## [22:20:59] WARNING: src/c_api/c_api.cc:935: `ntree_limit` is deprecated, use `iteration_range` instead.
## [22:20:59] WARNING: src/c_api/c_api.cc:935: `ntree_limit` is deprecated, use `iteration_range` instead.
## [22:21:00] WARNING: src/c_api/c_api.cc:935: `ntree_limit` is deprecated, use `iteration_range` instead.
## [22:21:00] WARNING: src/c_api/c_api.cc:935: `ntree_limit` is deprecated, use `iteration_range` instead.
## [22:21:00] WARNING: src/c_api/c_api.cc:935: `ntree_limit` is deprecated, use `iteration_range` instead.
## [22:21:00] WARNING: src/c_api/c_api.cc:935: `ntree_limit` is deprecated, use `iteration_range` instead.
## [22:21:01] WARNING: src/c_api/c_api.cc:935: `ntree_limit` is deprecated, use `iteration_range` instead.
## [22:21:01] WARNING: src/c_api/c_api.cc:935: `ntree_limit` is deprecated, use `iteration_range` instead.
## [22:21:01] WARNING: src/c_api/c_api.cc:935: `ntree_limit` is deprecated, use `iteration_range` instead.
## [22:21:01] WARNING: src/c_api/c_api.cc:935: `ntree_limit` is deprecated, use `iteration_range` instead.
## [22:21:02] WARNING: src/c_api/c_api.cc:935: `ntree_limit` is deprecated, use `iteration_range` instead.
## [22:21:02] WARNING: src/c_api/c_api.cc:935: `ntree_limit` is deprecated, use `iteration_range` instead.
## [22:21:03] WARNING: src/c_api/c_api.cc:935: `ntree_limit` is deprecated, use `iteration_range` instead.
## [22:21:03] WARNING: src/c_api/c_api.cc:935: `ntree_limit` is deprecated, use `iteration_range` instead.
## [22:21:03] WARNING: src/c_api/c_api.cc:935: `ntree_limit` is deprecated, use `iteration_range` instead.
## [22:21:03] WARNING: src/c_api/c_api.cc:935: `ntree_limit` is deprecated, use `iteration_range` instead.
## [22:21:03] WARNING: src/c_api/c_api.cc:935: `ntree_limit` is deprecated, use `iteration_range` instead.
## [22:21:03] WARNING: src/c_api/c_api.cc:935: `ntree_limit` is deprecated, use `iteration_range` instead.
## [22:21:04] WARNING: src/c_api/c_api.cc:935: `ntree_limit` is deprecated, use `iteration_range` instead.
## [22:21:04] WARNING: src/c_api/c_api.cc:935: `ntree_limit` is deprecated, use `iteration_range` instead.
## [22:21:04] WARNING: src/c_api/c_api.cc:935: `ntree_limit` is deprecated, use `iteration_range` instead.
## [22:21:04] WARNING: src/c_api/c_api.cc:935: `ntree_limit` is deprecated, use `iteration_range` instead.
## [22:21:04] WARNING: src/c_api/c_api.cc:935: `ntree_limit` is deprecated, use `iteration_range` instead.
## [22:21:04] WARNING: src/c_api/c_api.cc:935: `ntree_limit` is deprecated, use `iteration_range` instead.
## [22:21:04] WARNING: src/c_api/c_api.cc:935: `ntree_limit` is deprecated, use `iteration_range` instead.
## [22:21:04] WARNING: src/c_api/c_api.cc:935: `ntree_limit` is deprecated, use `iteration_range` instead.
## [22:21:05] WARNING: src/c_api/c_api.cc:935: `ntree_limit` is deprecated, use `iteration_range` instead.
## [22:21:05] WARNING: src/c_api/c_api.cc:935: `ntree_limit` is deprecated, use `iteration_range` instead.
## [22:21:05] WARNING: src/c_api/c_api.cc:935: `ntree_limit` is deprecated, use `iteration_range` instead.
## [22:21:05] WARNING: src/c_api/c_api.cc:935: `ntree_limit` is deprecated, use `iteration_range` instead.
## [22:21:05] WARNING: src/c_api/c_api.cc:935: `ntree_limit` is deprecated, use `iteration_range` instead.
## [22:21:05] WARNING: src/c_api/c_api.cc:935: `ntree_limit` is deprecated, use `iteration_range` instead.
## [22:21:06] WARNING: src/c_api/c_api.cc:935: `ntree_limit` is deprecated, use `iteration_range` instead.
## [22:21:06] WARNING: src/c_api/c_api.cc:935: `ntree_limit` is deprecated, use `iteration_range` instead.
## [22:21:06] WARNING: src/c_api/c_api.cc:935: `ntree_limit` is deprecated, use `iteration_range` instead.
## [22:21:06] WARNING: src/c_api/c_api.cc:935: `ntree_limit` is deprecated, use `iteration_range` instead.
## [22:21:07] WARNING: src/c_api/c_api.cc:935: `ntree_limit` is deprecated, use `iteration_range` instead.
## [22:21:07] WARNING: src/c_api/c_api.cc:935: `ntree_limit` is deprecated, use `iteration_range` instead.
## [22:21:07] WARNING: src/c_api/c_api.cc:935: `ntree_limit` is deprecated, use `iteration_range` instead.
## [22:21:07] WARNING: src/c_api/c_api.cc:935: `ntree_limit` is deprecated, use `iteration_range` instead.
## [22:21:08] WARNING: src/c_api/c_api.cc:935: `ntree_limit` is deprecated, use `iteration_range` instead.
## [22:21:08] WARNING: src/c_api/c_api.cc:935: `ntree_limit` is deprecated, use `iteration_range` instead.
## [22:21:09] WARNING: src/c_api/c_api.cc:935: `ntree_limit` is deprecated, use `iteration_range` instead.
## [22:21:09] WARNING: src/c_api/c_api.cc:935: `ntree_limit` is deprecated, use `iteration_range` instead.
## [22:21:09] WARNING: src/c_api/c_api.cc:935: `ntree_limit` is deprecated, use `iteration_range` instead.
## [22:21:09] WARNING: src/c_api/c_api.cc:935: `ntree_limit` is deprecated, use `iteration_range` instead.
## [22:21:10] WARNING: src/c_api/c_api.cc:935: `ntree_limit` is deprecated, use `iteration_range` instead.
## [22:21:10] WARNING: src/c_api/c_api.cc:935: `ntree_limit` is deprecated, use `iteration_range` instead.
## [22:21:11] WARNING: src/c_api/c_api.cc:935: `ntree_limit` is deprecated, use `iteration_range` instead.
## [22:21:11] WARNING: src/c_api/c_api.cc:935: `ntree_limit` is deprecated, use `iteration_range` instead.
## [22:21:11] WARNING: src/c_api/c_api.cc:935: `ntree_limit` is deprecated, use `iteration_range` instead.
## [22:21:11] WARNING: src/c_api/c_api.cc:935: `ntree_limit` is deprecated, use `iteration_range` instead.
## [22:21:11] WARNING: src/c_api/c_api.cc:935: `ntree_limit` is deprecated, use `iteration_range` instead.
## [22:21:11] WARNING: src/c_api/c_api.cc:935: `ntree_limit` is deprecated, use `iteration_range` instead.
## [22:21:11] WARNING: src/c_api/c_api.cc:935: `ntree_limit` is deprecated, use `iteration_range` instead.
## [22:21:11] WARNING: src/c_api/c_api.cc:935: `ntree_limit` is deprecated, use `iteration_range` instead.
## [22:21:12] WARNING: src/c_api/c_api.cc:935: `ntree_limit` is deprecated, use `iteration_range` instead.
## [22:21:12] WARNING: src/c_api/c_api.cc:935: `ntree_limit` is deprecated, use `iteration_range` instead.
## [22:21:12] WARNING: src/c_api/c_api.cc:935: `ntree_limit` is deprecated, use `iteration_range` instead.
## [22:21:12] WARNING: src/c_api/c_api.cc:935: `ntree_limit` is deprecated, use `iteration_range` instead.
## [22:21:12] WARNING: src/c_api/c_api.cc:935: `ntree_limit` is deprecated, use `iteration_range` instead.
## [22:21:12] WARNING: src/c_api/c_api.cc:935: `ntree_limit` is deprecated, use `iteration_range` instead.
## [22:21:13] WARNING: src/c_api/c_api.cc:935: `ntree_limit` is deprecated, use `iteration_range` instead.
## [22:21:13] WARNING: src/c_api/c_api.cc:935: `ntree_limit` is deprecated, use `iteration_range` instead.
## [22:21:13] WARNING: src/c_api/c_api.cc:935: `ntree_limit` is deprecated, use `iteration_range` instead.
## [22:21:13] WARNING: src/c_api/c_api.cc:935: `ntree_limit` is deprecated, use `iteration_range` instead.
## [22:21:13] WARNING: src/c_api/c_api.cc:935: `ntree_limit` is deprecated, use `iteration_range` instead.
## [22:21:13] WARNING: src/c_api/c_api.cc:935: `ntree_limit` is deprecated, use `iteration_range` instead.
## [22:21:14] WARNING: src/c_api/c_api.cc:935: `ntree_limit` is deprecated, use `iteration_range` instead.
## [22:21:14] WARNING: src/c_api/c_api.cc:935: `ntree_limit` is deprecated, use `iteration_range` instead.
## [22:21:14] WARNING: src/c_api/c_api.cc:935: `ntree_limit` is deprecated, use `iteration_range` instead.
## [22:21:14] WARNING: src/c_api/c_api.cc:935: `ntree_limit` is deprecated, use `iteration_range` instead.
## [22:21:15] WARNING: src/c_api/c_api.cc:935: `ntree_limit` is deprecated, use `iteration_range` instead.
## [22:21:15] WARNING: src/c_api/c_api.cc:935: `ntree_limit` is deprecated, use `iteration_range` instead.
## [22:21:15] WARNING: src/c_api/c_api.cc:935: `ntree_limit` is deprecated, use `iteration_range` instead.
## [22:21:15] WARNING: src/c_api/c_api.cc:935: `ntree_limit` is deprecated, use `iteration_range` instead.
## [22:21:16] WARNING: src/c_api/c_api.cc:935: `ntree_limit` is deprecated, use `iteration_range` instead.
## [22:21:16] WARNING: src/c_api/c_api.cc:935: `ntree_limit` is deprecated, use `iteration_range` instead.
## [22:21:17] WARNING: src/c_api/c_api.cc:935: `ntree_limit` is deprecated, use `iteration_range` instead.
## [22:21:17] WARNING: src/c_api/c_api.cc:935: `ntree_limit` is deprecated, use `iteration_range` instead.
## [22:21:17] WARNING: src/c_api/c_api.cc:935: `ntree_limit` is deprecated, use `iteration_range` instead.
## [22:21:17] WARNING: src/c_api/c_api.cc:935: `ntree_limit` is deprecated, use `iteration_range` instead.
## [22:21:18] WARNING: src/c_api/c_api.cc:935: `ntree_limit` is deprecated, use `iteration_range` instead.
## [22:21:18] WARNING: src/c_api/c_api.cc:935: `ntree_limit` is deprecated, use `iteration_range` instead.
## [22:21:19] WARNING: src/c_api/c_api.cc:935: `ntree_limit` is deprecated, use `iteration_range` instead.
## [22:21:19] WARNING: src/c_api/c_api.cc:935: `ntree_limit` is deprecated, use `iteration_range` instead.
```

# Predicted RT

Joining the experimental RT data from the interlaboratory comparison
with the RF predicted retention times.

```
data = data %>%
  left_join(data_NTS_pred)
```

Correlation of the experimental and predicted RT values prior to RTI
calculation and projection.

```
ggplot(data = data) +
  geom_point(mapping = aes(x = RT,
                           y = RT_xgbTREE_pred),
             color = highlightercolor6) +
  facet_wrap(~CS_code) +
  labs(x = "RTI measured", y = "RTI pred") +
  my_theme
```

## Projection of the predicted RT values

Calibrating the RT predictions to the RTI for all labs.

```
data = data %>%
  left_join(data %>%
            filter(sample_type == "cal") %>%
            group_by(CS_code) %>%
            summarise(RTmin = min(RT),
                      RTmax = max(RT)) %>%
            ungroup())

data = data %>%
  group_by(CS_code) %>%
  mutate(RTI = (RT - RTmin)/(RTmax-RTmin)*1000) %>%
  ungroup() %>%
  filter(RTI >= 0 & RTI <= 1000)
```

Projection with GAM for all labs.

```
data_pred = tibble()
for(CS_this in levels(factor(data$CS_code))) {
  data_this = data %>%
    filter(CS_code == CS_this) %>%
    select(CS_code, compound, RTI, RT_xgbTREE_pred, sample_type) %>%
    unique()
    model_this = gam(RTI ~ s(RT_xgbTREE_pred, bs = "cr", k = 6),
                     data = data_this %>%
                       filter(sample_type == "cal"))
    
    data_this = data_this %>%
      mutate(RTI_pred = predict.gam(model_this,
                                      newdata = data_this))
  data_pred = data_pred %>%
    bind_rows(data_this)
}

data_pred = data_pred %>%
  rowwise() %>%
  mutate(RTI_pred = RTI_pred[1])
```

Visualization of the results

```
ggplot(data = data_pred %>%
         filter(sample_type == "sus")) +
  geom_point(mapping = aes(x = RTI,
                           y = RTI_pred),
             color = highlightercolor6) +
  geom_abline(intercept = 0, slope = 1) +
  facet_wrap(~CS_code) +
  ylim(0, 1000) +
  xlim(0, 1000) +
  labs(x = "RTI measured", y = "RTI pred") +
  my_theme +
  theme(axis.text.x = element_text(angle = 90))
```

Statistical overview of the results

```
data_pred %>%
  na.omit() %>%
  group_by(sample_type) %>%
  summarize(RMSE = rmse(RTI, RTI_pred),
            MAD = mad(RTI, RTI_pred),
            Q95 = quantile(abs(RTI - RTI_pred), probs = c(0.95)),
            R2 = cor(RTI, RTI_pred)^2) %>%
  ungroup()
```

```
## # A tibble: 2 × 5
##   sample_type  RMSE   MAD   Q95    R2
##   <chr>       <dbl> <dbl> <dbl> <dbl>
## 1 cal          92.7  74.5  204. 0.871
## 2 sus         149.  150.   290. 0.607
```

```
data_pred %>%
  na.omit() %>%
  filter(sample_type == "sus") %>%
  group_by(CS_code) %>%
  summarize(RMSE = rmse(RTI, RTI_pred),
            MAD = mad(RTI, RTI_pred),
            Q95 = quantile(abs(RTI - RTI_pred), probs = c(0.95)),
            R2 = cor(RTI, RTI_pred)^2) %>%
  ungroup() %>%
  arrange(RMSE)
```

```
## # A tibble: 37 × 5
##    CS_code  RMSE   MAD   Q95    R2
##    <chr>   <dbl> <dbl> <dbl> <dbl>
##  1 DS_AWW   86.8 105.   157. 0.822
##  2 DS_JBQ   93.1  75.9  196. 0.752
##  3 DS_TJQ   99.5 105.   172. 0.755
##  4 DS_JSG  101.  101.   186. 0.764
##  5 DS_QQT  113.  116.   203. 0.796
##  6 DS_QJS  114.   63.6  190. 0.725
##  7 DS_TDF  121.  145.   205. 0.601
##  8 DS_Q    123.  136.   214. 0.584
##  9 DS_STF  130.  170.   208. 0.579
## 10 DS_QBD  131.  112.   239. 0.567
## # ℹ 27 more rows
```

```
data_pred %>%
  na.omit() %>%
  filter(sample_type == "cal") %>%
  group_by(CS_code) %>%
  summarize(RMSE = rmse(RTI, RTI_pred),
            MAD = mad(RTI, RTI_pred),
            Q95 = quantile(abs(RTI - RTI_pred), probs = c(0.95)),
            R2 = cor(RTI, RTI_pred)^2) %>%
  ungroup() %>%
  arrange(RMSE)
```

```
## # A tibble: 37 × 5
##    CS_code  RMSE   MAD   Q95    R2
##    <chr>   <dbl> <dbl> <dbl> <dbl>
##  1 DS_QDJ   50.7  74.7  80.2 0.963
##  2 DS_AWW   52.2  61.8  85.4 0.944
##  3 DS_JBQ   58.4  40.1 110.  0.929
##  4 DS_GS    66.8  41.7 146.  0.897
##  5 DS_GSB   67.9  58.5 129.  0.906
##  6 DS_JSG   68.5  64.0 124.  0.918
##  7 DS_BIJ   72.3  92.6 109.  0.866
##  8 DS_TDF   74.7  48.0 147.  0.872
##  9 DS_YF    76.0  48.9 173.  0.874
## 10 DS_QJS   76.1  42.1 164.  0.878
## # ℹ 27 more rows
```

```
ggplot(data = data_pred %>%
         na.omit() %>%
         group_by(CS_code, sample_type) %>%
         summarize(RMSE = rmse(RTI, RTI_pred)) %>%
         ungroup() %>%
         spread(key = sample_type, value = RMSE)) +
  geom_text(mapping = aes(x = cal,
                          y = sus,
                          label = CS_code),
            size = 3,
            color = "black") +
  my_theme
```

```
data_cor = data_pred %>%
  na.omit() %>%
  group_by(CS_code, sample_type) %>%
  summarize(RMSE = rmse(RTI, RTI_pred)) %>%
  ungroup() %>%
  spread(key = sample_type, value = RMSE)

cor.test(data_cor$sus, data_cor$cal, method = "pearson")
```

```
## 
##  Pearson's product-moment correlation
## 
## data:  data_cor$sus and data_cor$cal
## t = 3.129, df = 35, p-value = 0.003527
## alternative hypothesis: true correlation is not equal to 0
## 95 percent confidence interval:
##  0.1691309 0.6874127
## sample estimates:
##       cor 
## 0.4675285
```

Comparison of R2 values for “prediction only” and
“prediction+projection” approach. COmaprison of other statistics is not
possible due to different gradient profiles implemented in different
CSs.

```
data_pred %>%
  na.omit() %>%
  filter(sample_type == "sus") %>%
  group_by(CS_code) %>%
  summarize(R2pp = cor(RTI, RTI_pred)^2,
            R2p = cor(RTI, RT_xgbTREE_pred)^2,
            diff_R2 = R2pp-R2p) %>%
  ungroup() %>%
  arrange(diff_R2) %>%
  print()
```

```
## # A tibble: 37 × 4
##    CS_code  R2pp   R2p diff_R2
##    <chr>   <dbl> <dbl>   <dbl>
##  1 DS_TL   0.451 0.542 -0.0911
##  2 DS_QDF  0.319 0.382 -0.0625
##  3 DS_VQL  0.440 0.495 -0.0551
##  4 DS_QDJ  0.623 0.673 -0.0497
##  5 DS_TSF  0.488 0.536 -0.0483
##  6 DS_JBQ  0.752 0.800 -0.0481
##  7 DS_QBD  0.567 0.611 -0.0440
##  8 DS_Q    0.584 0.621 -0.0369
##  9 DS_BIJ  0.542 0.573 -0.0308
## 10 DS_HT   0.514 0.541 -0.0276
## # ℹ 27 more rows
```
