# Supplementary material for: Do experimental projection methods outcompete retention time prediction models in non-target screening? A case study on LC/HRMS interlaboratory comparison data
Source: Analyst. 2025 Jul 8;150(16):3567–77. doi: 10.1039/d5an00323g (PMC12268317; doi:10.1039/d5an00323g)
Supplement: AN-150-D5AN00323G-s001 [file AN-150-D5AN00323G-s001.zip › SI/SI4_projection_prediction_comparisson.html]

Comparitsson on RT projection vs prediction approach


# Comparitsson on RT projection vs prediction approach

#### Anneli Kruve

#### 2025-02-14

## Libraries

# Prediction vs Projection

Reading in both data

```
data_projections = read_delim("results/NORMAN_RTI_projection_results.csv")
data_predictions = read_delim("results/NORMAN_RTI_prediction_results.csv")
```

```
data_comb = data_projections %>%
  select(CS_code = CS_x, CS_y, compound, sample_type, RTI_y, RTI_y_pred) %>%
  left_join(data_predictions %>%
              select(CS_code, compound, sample_type, RTI, RTI_pred))

data_sum = data_comb %>%
  na.omit() %>%
  group_by(CS_code, CS_y, sample_type) %>%
  summarize(RMSE_prediction = rmse(RTI, RTI_pred),
            RMSE_projection = RMSE(RTI_y, RTI_y_pred),
            improve = case_when(
              RMSE_projection < RMSE_prediction ~ TRUE,
              TRUE ~ FALSE)) %>%
  ungroup() 

data_sum %>%
  filter(sample_type == "sus") %>%
  arrange(CS_code)
```

```
## # A tibble: 1,369 × 6
##    CS_code CS_y   sample_type RMSE_prediction RMSE_projection improve
##    <chr>   <chr>  <chr>                 <dbl>           <dbl> <lgl>  
##  1 DS_AW   DS_AW  sus                    198.        2.31e-13 TRUE   
##  2 DS_AW   DS_AWW sus                    195.        1.56e+ 2 TRUE   
##  3 DS_AW   DS_BIJ sus                    183.        8.14e+ 1 TRUE   
##  4 DS_AW   DS_BQW sus                    198.        7.18e+ 1 TRUE   
##  5 DS_AW   DS_DBS sus                    198.        2.43e+ 1 TRUE   
##  6 DS_AW   DS_DID sus                    165.        4.99e+ 1 TRUE   
##  7 DS_AW   DS_DP  sus                    202.        3.76e+ 1 TRUE   
##  8 DS_AW   DS_EF  sus                    198.        3.50e+ 1 TRUE   
##  9 DS_AW   DS_GJT sus                    202.        5.61e+ 1 TRUE   
## 10 DS_AW   DS_GS  sus                    198.        4.65e+ 1 TRUE   
## # ℹ 1,359 more rows
```

Statistical comparisson of the RMSE values of projection and
prediction method with F-test.

```
results_var_test = tibble()
for(CS_this in levels(factor(data_comb$CS_code))) {
  for(CS_that in levels(factor(data_comb$CS_y))) {
    data_xy_this_that = data_comb %>%
      filter(CS_code == CS_this & CS_y == CS_that) %>%
      na.omit()
    
    result = var.test(lm(RTI_y ~ 0 + offset(1*RTI_y_pred), 
                         data = data_xy_this_that %>%
                           filter(sample_type == "sus")), 
                      lm(RTI ~ 0 + offset(1*RTI_pred), 
                         data = data_xy_this_that %>%
                           filter(sample_type == "sus")))
    
    results_var_test = results_var_test %>%
      bind_rows(tibble(CS_code = CS_this,
                       CS_y = CS_that,
                       p_value = result$p.value))
  }
}
```

Combining the p-values with previouse data.

```
data_sum = data_sum %>%
  filter(sample_type == "sus") %>%
  left_join(results_var_test) 

data_sum = data_sum %>%
  mutate(stat_sign = case_when(
    p_value < 0.05 ~ TRUE,
    TRUE ~ FALSE),
    better = case_when(
      improve & stat_sign ~ "projection",
      !improve & stat_sign ~ "prediction",
      TRUE ~ "equal"
    ))

data_sum %>%
  filter(CS_code != CS_y) %>%
  group_by(better) %>%
  summarize(count = n()) %>%
  ungroup()
```

```
## # A tibble: 3 × 2
##   better     count
##   <chr>      <int>
## 1 equal        348
## 2 prediction    32
## 3 projection   952
```
