# Supplementary material for: Do experimental projection methods outcompete retention time prediction models in non-target screening? A case study on LC/HRMS interlaboratory comparison data
Source: Analyst. 2025 Jul 8;150(16):3567–77. doi: 10.1039/d5an00323g (PMC12268317; doi:10.1039/d5an00323g)
Supplement: AN-150-D5AN00323G-s001 [file AN-150-D5AN00323G-s001.zip › SI/SI5_prediction_RF_calibrants.html]

model\_training


# model\_training

#### Anneli Kruve

#### 2024-12-09

## Libraries

## Functions for preprocessing and modelling

```
remove_correlated_nearZero = function(data = tibble(),
                                      cutoff = numeric()) {
  data = data %>%
    mutate(Lipinski = as.numeric(Lipinski),
               GhoseFilter = as.numeric(GhoseFilter))

  data = data %>%
    relocate(c(SMILES, RTI, mol, standardized_SMILES, standardized_inchikey, MF), .after = last_col())

  data = data %>%
    select(-nearZeroVar(data %>%
                          select(-c(SMILES, RTI, mol, standardized_SMILES, standardized_inchikey, MF))))

  correlation_matrix = cor(data %>%
                             select(-c(SMILES, RTI, mol, standardized_SMILES, standardized_inchikey, MF)))

  highly_correlated = findCorrelation(correlation_matrix,
                                      cutoff = cutoff)

  data = data %>%
    select(-all_of(highly_correlated))

  data = data %>%
    select(SMILES, RTI, mol, standardized_SMILES, standardized_inchikey, MF, colnames(data))

  return(data)

}

model_smart_combined = function(training_data = tibble(),
                                test_data = tibble(),
                                cutoff_correlated = 0.7,
                                cross_validation_folds = 5) {

  training_data = remove_correlated_nearZero(training_data,
                                             cutoff_correlated)

  test_data = test_data %>%
    mutate(Lipinski = as.numeric(Lipinski),
           GhoseFilter = as.numeric(GhoseFilter))

  test_data = test_data %>%
    select(colnames(training_data)) %>%
    na.omit()

  fitControl = trainControl(method = "repeatedcv",
                           number = 2,
                           repeats = 2)

 model_xgbTREE = train(RTI ~ ., 
                        data = training_data %>%
                          select(-c(SMILES, mol, standardized_SMILES, standardized_inchikey, MF)), 
                        method = "xgbTree", 
                        trControl = fitControl)

  training_data = training_data %>%
    mutate(RTI_xgbTREE_pred = predict(model_xgbTREE,
                                 newdata = training_data)) %>%
    select(SMILES, RTI, mol, standardized_SMILES, standardized_inchikey, MF, RTI_xgbTREE_pred)

  test_data = test_data %>%
    mutate(RTI_xgbTREE_pred = predict(model_xgbTREE,
                                 newdata = test_data)) %>%
    select(SMILES, RTI, mol, standardized_SMILES, standardized_inchikey, MF, RTI_xgbTREE_pred)

  all = list("training_data" = training_data,
             "test_data" = test_data)

  return(all)
}
```

## Data

Experimental RT data from the interlaboratory comparison.

```
data = read_delim("data/NORMAN_interlab_RT_data.csv")
```

Mordred descriptors for the data

```
data_norman = read_delim("data/NORMAN_Mordreds_2024-11-02.tsv")
```

Calculating the RTI values for all CSs.

```
data = data %>%
  left_join(data %>%
            filter(sample_type == "cal") %>%
            group_by(CS_code) %>%
            summarise(RTmin = min(RT),
                      RTmax = max(RT)) %>%
            ungroup())

data = data %>%
  group_by(CS_code) %>%
  mutate(RTI = (RT - RTmin)/(RTmax-RTmin)*1000) %>%
  ungroup() %>%
  select(-RTmax, -RTmin, -RT)
```

## Modelling

```
set.seed(123)
data_prediction = tibble()
for (CS_this in levels(factor(data$CS_code))) {
  data_lab = data %>%
    filter(CS_code == CS_this) %>%
    left_join(data_norman)
  
  training_data = data_lab %>%
    filter(sample_type == "cal") %>%
    select(-CS_code, -compound, -sample_type, -ABC, -ABCGG)
  
  test_data = data_lab %>%
    filter(sample_type == "sus") %>%
    select(-CS_code, -compound, -sample_type, -ABC, -ABCGG)
  
  training_data = training_data %>%
    select_if(~sum(is.na(.)) < 1) %>%
    na.omit()
  
  preProcValues = preProcess(training_data %>%
                               select(-c(SMILES, RTI, mol, standardized_SMILES, standardized_inchikey, MF)),
                             method = c("center", "scale"))

  trainTransformed = predict(preProcValues, training_data)
  testTransformed = predict(preProcValues, test_data)
  
  nr_points = dim(training_data)[1]
  
  model_this = model_smart_combined(training_data = trainTransformed,
                                    test_data = testTransformed,
                                    cutoff_correlated = 0.7)

  data_prediction = data_prediction %>%
    bind_rows(model_this$test_data %>%
                mutate(CS_code = CS_this,
                       nr_points = nr_points))
}
```

```
## [15:49:27] WARNING: src/c_api/c_api.cc:935: `ntree_limit` is deprecated, use `iteration_range` instead.
## [15:49:27] WARNING: src/c_api/c_api.cc:935: `ntree_limit` is deprecated, use `iteration_range` instead.
## [15:49:27] WARNING: src/c_api/c_api.cc:935: `ntree_limit` is deprecated, use `iteration_range` instead.
## [15:49:27] WARNING: src/c_api/c_api.cc:935: `ntree_limit` is deprecated, use `iteration_range` instead.
## [15:49:28] WARNING: src/c_api/c_api.cc:935: `ntree_limit` is deprecated, use `iteration_range` instead.
## [15:49:28] WARNING: src/c_api/c_api.cc:935: `ntree_limit` is deprecated, use `iteration_range` instead.
## [15:49:28] WARNING: src/c_api/c_api.cc:935: `ntree_limit` is deprecated, use `iteration_range` instead.
## [15:49:28] WARNING: src/c_api/c_api.cc:935: `ntree_limit` is deprecated, use `iteration_range` instead.
## [15:49:28] WARNING: src/c_api/c_api.cc:935: `ntree_limit` is deprecated, use `iteration_range` instead.
## [15:49:28] WARNING: src/c_api/c_api.cc:935: `ntree_limit` is deprecated, use `iteration_range` instead.
## [15:49:28] WARNING: src/c_api/c_api.cc:935: `ntree_limit` is deprecated, use `iteration_range` instead.
## [15:49:28] WARNING: src/c_api/c_api.cc:935: `ntree_limit` is deprecated, use `iteration_range` instead.
## [15:49:28] WARNING: src/c_api/c_api.cc:935: `ntree_limit` is deprecated, use `iteration_range` instead.
## [15:49:28] WARNING: src/c_api/c_api.cc:935: `ntree_limit` is deprecated, use `iteration_range` instead.
## [15:49:28] WARNING: src/c_api/c_api.cc:935: `ntree_limit` is deprecated, use `iteration_range` instead.
## [15:49:28] WARNING: src/c_api/c_api.cc:935: `ntree_limit` is deprecated, use `iteration_range` instead.
## [15:49:28] WARNING: src/c_api/c_api.cc:935: `ntree_limit` is deprecated, use `iteration_range` instead.
## [15:49:28] WARNING: src/c_api/c_api.cc:935: `ntree_limit` is deprecated, use `iteration_range` instead.
## [15:49:29] WARNING: src/c_api/c_api.cc:935: `ntree_limit` is deprecated, use `iteration_range` instead.
## [15:49:29] WARNING: src/c_api/c_api.cc:935: `ntree_limit` is deprecated, use `iteration_range` instead.
## [15:49:29] WARNING: src/c_api/c_api.cc:935: `ntree_limit` is deprecated, use `iteration_range` instead.
## [15:49:29] WARNING: src/c_api/c_api.cc:935: `ntree_limit` is deprecated, use `iteration_range` instead.
## [15:49:29] WARNING: src/c_api/c_api.cc:935: `ntree_limit` is deprecated, use `iteration_range` instead.
## [15:49:29] WARNING: src/c_api/c_api.cc:935: `ntree_limit` is deprecated, use `iteration_range` instead.
## [15:49:29] WARNING: src/c_api/c_api.cc:935: `ntree_limit` is deprecated, use `iteration_range` instead.
## [15:49:29] WARNING: src/c_api/c_api.cc:935: `ntree_limit` is deprecated, use `iteration_range` instead.
## [15:49:29] WARNING: src/c_api/c_api.cc:935: `ntree_limit` is deprecated, use `iteration_range` instead.
## [15:49:29] WARNING: src/c_api/c_api.cc:935: `ntree_limit` is deprecated, use `iteration_range` instead.
## [15:49:29] WARNING: src/c_api/c_api.cc:935: `ntree_limit` is deprecated, use `iteration_range` instead.
## [15:49:29] WARNING: src/c_api/c_api.cc:935: `ntree_limit` is deprecated, use `iteration_range` instead.
## [15:49:30] WARNING: src/c_api/c_api.cc:935: `ntree_limit` is deprecated, use `iteration_range` instead.
## [15:49:30] WARNING: src/c_api/c_api.cc:935: `ntree_limit` is deprecated, use `iteration_range` instead.
## [15:49:30] WARNING: src/c_api/c_api.cc:935: `ntree_limit` is deprecated, use `iteration_range` instead.
## [15:49:30] WARNING: src/c_api/c_api.cc:935: `ntree_limit` is deprecated, use `iteration_range` instead.
## [15:49:30] WARNING: src/c_api/c_api.cc:935: `ntree_limit` is deprecated, use `iteration_range` instead.
## [15:49:30] WARNING: src/c_api/c_api.cc:935: `ntree_limit` is deprecated, use `iteration_range` instead.
## [15:49:30] WARNING: src/c_api/c_api.cc:935: `ntree_limit` is deprecated, use `iteration_range` instead.
## [15:49:30] WARNING: src/c_api/c_api.cc:935: `ntree_limit` is deprecated, use `iteration_range` instead.
## [15:49:30] WARNING: src/c_api/c_api.cc:935: `ntree_limit` is deprecated, use `iteration_range` instead.
## [15:49:30] WARNING: src/c_api/c_api.cc:935: `ntree_limit` is deprecated, use `iteration_range` instead.
## [15:49:30] WARNING: src/c_api/c_api.cc:935: `ntree_limit` is deprecated, use `iteration_range` instead.
## [15:49:30] WARNING: src/c_api/c_api.cc:935: `ntree_limit` is deprecated, use `iteration_range` instead.
## [15:49:30] WARNING: src/c_api/c_api.cc:935: `ntree_limit` is deprecated, use `iteration_range` instead.
## [15:49:30] WARNING: src/c_api/c_api.cc:935: `ntree_limit` is deprecated, use `iteration_range` instead.
## [15:49:30] WARNING: src/c_api/c_api.cc:935: `ntree_limit` is deprecated, use `iteration_range` instead.
## [15:49:30] WARNING: src/c_api/c_api.cc:935: `ntree_limit` is deprecated, use `iteration_range` instead.
## [15:49:31] WARNING: src/c_api/c_api.cc:935: `ntree_limit` is deprecated, use `iteration_range` instead.
## [15:49:31] WARNING: src/c_api/c_api.cc:935: `ntree_limit` is deprecated, use `iteration_range` instead.
## [15:49:31] WARNING: src/c_api/c_api.cc:935: `ntree_limit` is deprecated, use `iteration_range` instead.
## [15:49:31] WARNING: src/c_api/c_api.cc:935: `ntree_limit` is deprecated, use `iteration_range` instead.
## [15:49:31] WARNING: src/c_api/c_api.cc:935: `ntree_limit` is deprecated, use `iteration_range` instead.
## [15:49:31] WARNING: src/c_api/c_api.cc:935: `ntree_limit` is deprecated, use `iteration_range` instead.
## [15:49:31] WARNING: src/c_api/c_api.cc:935: `ntree_limit` is deprecated, use `iteration_range` instead.
## [15:49:31] WARNING: src/c_api/c_api.cc:935: `ntree_limit` is deprecated, use `iteration_range` instead.
## [15:49:31] WARNING: src/c_api/c_api.cc:935: `ntree_limit` is deprecated, use `iteration_range` instead.
## [15:49:31] WARNING: src/c_api/c_api.cc:935: `ntree_limit` is deprecated, use `iteration_range` instead.
## [15:49:31] WARNING: src/c_api/c_api.cc:935: `ntree_limit` is deprecated, use `iteration_range` instead.
## [15:49:31] WARNING: src/c_api/c_api.cc:935: `ntree_limit` is deprecated, use `iteration_range` instead.
## [15:49:31] WARNING: src/c_api/c_api.cc:935: `ntree_limit` is deprecated, use `iteration_range` instead.
## [15:49:31] WARNING: src/c_api/c_api.cc:935: `ntree_limit` is deprecated, use `iteration_range` instead.
## [15:49:32] WARNING: src/c_api/c_api.cc:935: `ntree_limit` is deprecated, use `iteration_range` instead.
## [15:49:32] WARNING: src/c_api/c_api.cc:935: `ntree_limit` is deprecated, use `iteration_range` instead.
## [15:49:32] WARNING: src/c_api/c_api.cc:935: `ntree_limit` is deprecated, use `iteration_range` instead.
## [15:49:32] WARNING: src/c_api/c_api.cc:935: `ntree_limit` is deprecated, use `iteration_range` instead.
## [15:49:32] WARNING: src/c_api/c_api.cc:935: `ntree_limit` is deprecated, use `iteration_range` instead.
## [15:49:32] WARNING: src/c_api/c_api.cc:935: `ntree_limit` is deprecated, use `iteration_range` instead.
## [15:49:32] WARNING: src/c_api/c_api.cc:935: `ntree_limit` is deprecated, use `iteration_range` instead.
## [15:49:32] WARNING: src/c_api/c_api.cc:935: `ntree_limit` is deprecated, use `iteration_range` instead.
## [15:49:32] WARNING: src/c_api/c_api.cc:935: `ntree_limit` is deprecated, use `iteration_range` instead.
## [15:49:32] WARNING: src/c_api/c_api.cc:935: `ntree_limit` is deprecated, use `iteration_range` instead.
## [15:49:32] WARNING: src/c_api/c_api.cc:935: `ntree_limit` is deprecated, use `iteration_range` instead.
## [15:49:32] WARNING: src/c_api/c_api.cc:935: `ntree_limit` is deprecated, use `iteration_range` instead.
## [15:49:32] WARNING: src/c_api/c_api.cc:935: `ntree_limit` is deprecated, use `iteration_range` instead.
## [15:49:32] WARNING: src/c_api/c_api.cc:935: `ntree_limit` is deprecated, use `iteration_range` instead.
## [15:49:33] WARNING: src/c_api/c_api.cc:935: `ntree_limit` is deprecated, use `iteration_range` instead.
## [15:49:33] WARNING: src/c_api/c_api.cc:935: `ntree_limit` is deprecated, use `iteration_range` instead.
## [15:49:33] WARNING: src/c_api/c_api.cc:935: `ntree_limit` is deprecated, use `iteration_range` instead.
## [15:49:33] WARNING: src/c_api/c_api.cc:935: `ntree_limit` is deprecated, use `iteration_range` instead.
## [15:49:33] WARNING: src/c_api/c_api.cc:935: `ntree_limit` is deprecated, use `iteration_range` instead.
## [15:49:33] WARNING: src/c_api/c_api.cc:935: `ntree_limit` is deprecated, use `iteration_range` instead.
## [15:49:33] WARNING: src/c_api/c_api.cc:935: `ntree_limit` is deprecated, use `iteration_range` instead.
## [15:49:33] WARNING: src/c_api/c_api.cc:935: `ntree_limit` is deprecated, use `iteration_range` instead.
## [15:49:33] WARNING: src/c_api/c_api.cc:935: `ntree_limit` is deprecated, use `iteration_range` instead.
## [15:49:33] WARNING: src/c_api/c_api.cc:935: `ntree_limit` is deprecated, use `iteration_range` instead.
## [15:49:33] WARNING: src/c_api/c_api.cc:935: `ntree_limit` is deprecated, use `iteration_range` instead.
## [15:49:33] WARNING: src/c_api/c_api.cc:935: `ntree_limit` is deprecated, use `iteration_range` instead.
## [15:49:33] WARNING: src/c_api/c_api.cc:935: `ntree_limit` is deprecated, use `iteration_range` instead.
## [15:49:33] WARNING: src/c_api/c_api.cc:935: `ntree_limit` is deprecated, use `iteration_range` instead.
## [15:49:33] WARNING: src/c_api/c_api.cc:935: `ntree_limit` is deprecated, use `iteration_range` instead.
## [15:49:33] WARNING: src/c_api/c_api.cc:935: `ntree_limit` is deprecated, use `iteration_range` instead.
## [15:49:34] WARNING: src/c_api/c_api.cc:935: `ntree_limit` is deprecated, use `iteration_range` instead.
## [15:49:34] WARNING: src/c_api/c_api.cc:935: `ntree_limit` is deprecated, use `iteration_range` instead.
## [15:49:34] WARNING: src/c_api/c_api.cc:935: `ntree_limit` is deprecated, use `iteration_range` instead.
## [15:49:34] WARNING: src/c_api/c_api.cc:935: `ntree_limit` is deprecated, use `iteration_range` instead.
## [15:49:34] WARNING: src/c_api/c_api.cc:935: `ntree_limit` is deprecated, use `iteration_range` instead.
## [15:49:34] WARNING: src/c_api/c_api.cc:935: `ntree_limit` is deprecated, use `iteration_range` instead.
## [15:49:34] WARNING: src/c_api/c_api.cc:935: `ntree_limit` is deprecated, use `iteration_range` instead.
## [15:49:34] WARNING: src/c_api/c_api.cc:935: `ntree_limit` is deprecated, use `iteration_range` instead.
## [15:49:34] WARNING: src/c_api/c_api.cc:935: `ntree_limit` is deprecated, use `iteration_range` instead.
## [15:49:34] WARNING: src/c_api/c_api.cc:935: `ntree_limit` is deprecated, use `iteration_range` instead.
## [15:49:34] WARNING: src/c_api/c_api.cc:935: `ntree_limit` is deprecated, use `iteration_range` instead.
## [15:49:34] WARNING: src/c_api/c_api.cc:935: `ntree_limit` is deprecated, use `iteration_range` instead.
## [15:49:34] WARNING: src/c_api/c_api.cc:935: `ntree_limit` is deprecated, use `iteration_range` instead.
## [15:49:34] WARNING: src/c_api/c_api.cc:935: `ntree_limit` is deprecated, use `iteration_range` instead.
## [15:49:35] WARNING: src/c_api/c_api.cc:935: `ntree_limit` is deprecated, use `iteration_range` instead.
## [15:49:35] WARNING: src/c_api/c_api.cc:935: `ntree_limit` is deprecated, use `iteration_range` instead.
## [15:49:35] WARNING: src/c_api/c_api.cc:935: `ntree_limit` is deprecated, use `iteration_range` instead.
## [15:49:35] WARNING: src/c_api/c_api.cc:935: `ntree_limit` is deprecated, use `iteration_range` instead.
## [15:49:35] WARNING: src/c_api/c_api.cc:935: `ntree_limit` is deprecated, use `iteration_range` instead.
## [15:49:35] WARNING: src/c_api/c_api.cc:935: `ntree_limit` is deprecated, use `iteration_range` instead.
## [15:49:35] WARNING: src/c_api/c_api.cc:935: `ntree_limit` is deprecated, use `iteration_range` instead.
## [15:49:35] WARNING: src/c_api/c_api.cc:935: `ntree_limit` is deprecated, use `iteration_range` instead.
## [15:49:35] WARNING: src/c_api/c_api.cc:935: `ntree_limit` is deprecated, use `iteration_range` instead.
## [15:49:35] WARNING: src/c_api/c_api.cc:935: `ntree_limit` is deprecated, use `iteration_range` instead.
## [15:49:35] WARNING: src/c_api/c_api.cc:935: `ntree_limit` is deprecated, use `iteration_range` instead.
## [15:49:35] WARNING: src/c_api/c_api.cc:935: `ntree_limit` is deprecated, use `iteration_range` instead.
## [15:49:35] WARNING: src/c_api/c_api.cc:935: `ntree_limit` is deprecated, use `iteration_range` instead.
## [15:49:35] WARNING: src/c_api/c_api.cc:935: `ntree_limit` is deprecated, use `iteration_range` instead.
## [15:49:35] WARNING: src/c_api/c_api.cc:935: `ntree_limit` is deprecated, use `iteration_range` instead.
## [15:49:35] WARNING: src/c_api/c_api.cc:935: `ntree_limit` is deprecated, use `iteration_range` instead.
## [15:49:36] WARNING: src/c_api/c_api.cc:935: `ntree_limit` is deprecated, use `iteration_range` instead.
## [15:49:36] WARNING: src/c_api/c_api.cc:935: `ntree_limit` is deprecated, use `iteration_range` instead.
## [15:49:36] WARNING: src/c_api/c_api.cc:935: `ntree_limit` is deprecated, use `iteration_range` instead.
## [15:49:36] WARNING: src/c_api/c_api.cc:935: `ntree_limit` is deprecated, use `iteration_range` instead.
## [15:49:36] WARNING: src/c_api/c_api.cc:935: `ntree_limit` is deprecated, use `iteration_range` instead.
## [15:49:36] WARNING: src/c_api/c_api.cc:935: `ntree_limit` is deprecated, use `iteration_range` instead.
## [15:49:36] WARNING: src/c_api/c_api.cc:935: `ntree_limit` is deprecated, use `iteration_range` instead.
## [15:49:36] WARNING: src/c_api/c_api.cc:935: `ntree_limit` is deprecated, use `iteration_range` instead.
## [15:49:36] WARNING: src/c_api/c_api.cc:935: `ntree_limit` is deprecated, use `iteration_range` instead.
## [15:49:36] WARNING: src/c_api/c_api.cc:935: `ntree_limit` is deprecated, use `iteration_range` instead.
## [15:49:36] WARNING: src/c_api/c_api.cc:935: `ntree_limit` is deprecated, use `iteration_range` instead.
## [15:49:36] WARNING: src/c_api/c_api.cc:935: `ntree_limit` is deprecated, use `iteration_range` instead.
## [15:49:36] WARNING: src/c_api/c_api.cc:935: `ntree_limit` is deprecated, use `iteration_range` instead.
## [15:49:36] WARNING: src/c_api/c_api.cc:935: `ntree_limit` is deprecated, use `iteration_range` instead.
## [15:49:37] WARNING: src/c_api/c_api.cc:935: `ntree_limit` is deprecated, use `iteration_range` instead.
## [15:49:37] WARNING: src/c_api/c_api.cc:935: `ntree_limit` is deprecated, use `iteration_range` instead.
## [15:49:37] WARNING: src/c_api/c_api.cc:935: `ntree_limit` is deprecated, use `iteration_range` instead.
## [15:49:37] WARNING: src/c_api/c_api.cc:935: `ntree_limit` is deprecated, use `iteration_range` instead.
## [15:49:37] WARNING: src/c_api/c_api.cc:935: `ntree_limit` is deprecated, use `iteration_range` instead.
## [15:49:37] WARNING: src/c_api/c_api.cc:935: `ntree_limit` is deprecated, use `iteration_range` instead.
## [15:49:37] WARNING: src/c_api/c_api.cc:935: `ntree_limit` is deprecated, use `iteration_range` instead.
## [15:49:37] WARNING: src/c_api/c_api.cc:935: `ntree_limit` is deprecated, use `iteration_range` instead.
## [15:49:37] WARNING: src/c_api/c_api.cc:935: `ntree_limit` is deprecated, use `iteration_range` instead.
## [15:49:37] WARNING: src/c_api/c_api.cc:935: `ntree_limit` is deprecated, use `iteration_range` instead.
## [15:49:37] WARNING: src/c_api/c_api.cc:935: `ntree_limit` is deprecated, use `iteration_range` instead.
## [15:49:37] WARNING: src/c_api/c_api.cc:935: `ntree_limit` is deprecated, use `iteration_range` instead.
## [15:49:37] WARNING: src/c_api/c_api.cc:935: `ntree_limit` is deprecated, use `iteration_range` instead.
## [15:49:37] WARNING: src/c_api/c_api.cc:935: `ntree_limit` is deprecated, use `iteration_range` instead.
## [15:49:38] WARNING: src/c_api/c_api.cc:935: `ntree_limit` is deprecated, use `iteration_range` instead.
## [15:49:38] WARNING: src/c_api/c_api.cc:935: `ntree_limit` is deprecated, use `iteration_range` instead.
## [15:49:38] WARNING: src/c_api/c_api.cc:935: `ntree_limit` is deprecated, use `iteration_range` instead.
## [15:49:38] WARNING: src/c_api/c_api.cc:935: `ntree_limit` is deprecated, use `iteration_range` instead.
## [15:49:38] WARNING: src/c_api/c_api.cc:935: `ntree_limit` is deprecated, use `iteration_range` instead.
## [15:49:38] WARNING: src/c_api/c_api.cc:935: `ntree_limit` is deprecated, use `iteration_range` instead.
## [15:49:38] WARNING: src/c_api/c_api.cc:935: `ntree_limit` is deprecated, use `iteration_range` instead.
## [15:49:38] WARNING: src/c_api/c_api.cc:935: `ntree_limit` is deprecated, use `iteration_range` instead.
## [15:49:38] WARNING: src/c_api/c_api.cc:935: `ntree_limit` is deprecated, use `iteration_range` instead.
## [15:49:38] WARNING: src/c_api/c_api.cc:935: `ntree_limit` is deprecated, use `iteration_range` instead.
## [15:49:38] WARNING: src/c_api/c_api.cc:935: `ntree_limit` is deprecated, use `iteration_range` instead.
## [15:49:38] WARNING: src/c_api/c_api.cc:935: `ntree_limit` is deprecated, use `iteration_range` instead.
## [15:49:38] WARNING: src/c_api/c_api.cc:935: `ntree_limit` is deprecated, use `iteration_range` instead.
## [15:49:38] WARNING: src/c_api/c_api.cc:935: `ntree_limit` is deprecated, use `iteration_range` instead.
## [15:49:38] WARNING: src/c_api/c_api.cc:935: `ntree_limit` is deprecated, use `iteration_range` instead.
## [15:49:38] WARNING: src/c_api/c_api.cc:935: `ntree_limit` is deprecated, use `iteration_range` instead.
## [15:49:39] WARNING: src/c_api/c_api.cc:935: `ntree_limit` is deprecated, use `iteration_range` instead.
## [15:49:39] WARNING: src/c_api/c_api.cc:935: `ntree_limit` is deprecated, use `iteration_range` instead.
## [15:49:39] WARNING: src/c_api/c_api.cc:935: `ntree_limit` is deprecated, use `iteration_range` instead.
## [15:49:39] WARNING: src/c_api/c_api.cc:935: `ntree_limit` is deprecated, use `iteration_range` instead.
## [15:49:39] WARNING: src/c_api/c_api.cc:935: `ntree_limit` is deprecated, use `iteration_range` instead.
## [15:49:39] WARNING: src/c_api/c_api.cc:935: `ntree_limit` is deprecated, use `iteration_range` instead.
## [15:49:39] WARNING: src/c_api/c_api.cc:935: `ntree_limit` is deprecated, use `iteration_range` instead.
## [15:49:39] WARNING: src/c_api/c_api.cc:935: `ntree_limit` is deprecated, use `iteration_range` instead.
## [15:49:39] WARNING: src/c_api/c_api.cc:935: `ntree_limit` is deprecated, use `iteration_range` instead.
## [15:49:39] WARNING: src/c_api/c_api.cc:935: `ntree_limit` is deprecated, use `iteration_range` instead.
## [15:49:40] WARNING: src/c_api/c_api.cc:935: `ntree_limit` is deprecated, use `iteration_range` instead.
## [15:49:40] WARNING: src/c_api/c_api.cc:935: `ntree_limit` is deprecated, use `iteration_range` instead.
## [15:49:40] WARNING: src/c_api/c_api.cc:935: `ntree_limit` is deprecated, use `iteration_range` instead.
## [15:49:40] WARNING: src/c_api/c_api.cc:935: `ntree_limit` is deprecated, use `iteration_range` instead.
## [15:49:40] WARNING: src/c_api/c_api.cc:935: `ntree_limit` is deprecated, use `iteration_range` instead.
## [15:49:40] WARNING: src/c_api/c_api.cc:935: `ntree_limit` is deprecated, use `iteration_range` instead.
## [15:49:40] WARNING: src/c_api/c_api.cc:935: `ntree_limit` is deprecated, use `iteration_range` instead.
## [15:49:40] WARNING: src/c_api/c_api.cc:935: `ntree_limit` is deprecated, use `iteration_range` instead.
## [15:49:40] WARNING: src/c_api/c_api.cc:935: `ntree_limit` is deprecated, use `iteration_range` instead.
## [15:49:40] WARNING: src/c_api/c_api.cc:935: `ntree_limit` is deprecated, use `iteration_range` instead.
## [15:49:40] WARNING: src/c_api/c_api.cc:935: `ntree_limit` is deprecated, use `iteration_range` instead.
## [15:49:40] WARNING: src/c_api/c_api.cc:935: `ntree_limit` is deprecated, use `iteration_range` instead.
## [15:49:40] WARNING: src/c_api/c_api.cc:935: `ntree_limit` is deprecated, use `iteration_range` instead.
## [15:49:40] WARNING: src/c_api/c_api.cc:935: `ntree_limit` is deprecated, use `iteration_range` instead.
## [15:49:41] WARNING: src/c_api/c_api.cc:935: `ntree_limit` is deprecated, use `iteration_range` instead.
## [15:49:41] WARNING: src/c_api/c_api.cc:935: `ntree_limit` is deprecated, use `iteration_range` instead.
## [15:49:41] WARNING: src/c_api/c_api.cc:935: `ntree_limit` is deprecated, use `iteration_range` instead.
## [15:49:41] WARNING: src/c_api/c_api.cc:935: `ntree_limit` is deprecated, use `iteration_range` instead.
## [15:49:41] WARNING: src/c_api/c_api.cc:935: `ntree_limit` is deprecated, use `iteration_range` instead.
## [15:49:41] WARNING: src/c_api/c_api.cc:935: `ntree_limit` is deprecated, use `iteration_range` instead.
## [15:49:41] WARNING: src/c_api/c_api.cc:935: `ntree_limit` is deprecated, use `iteration_range` instead.
## [15:49:41] WARNING: src/c_api/c_api.cc:935: `ntree_limit` is deprecated, use `iteration_range` instead.
## [15:49:41] WARNING: src/c_api/c_api.cc:935: `ntree_limit` is deprecated, use `iteration_range` instead.
## [15:49:41] WARNING: src/c_api/c_api.cc:935: `ntree_limit` is deprecated, use `iteration_range` instead.
## [15:49:41] WARNING: src/c_api/c_api.cc:935: `ntree_limit` is deprecated, use `iteration_range` instead.
## [15:49:41] WARNING: src/c_api/c_api.cc:935: `ntree_limit` is deprecated, use `iteration_range` instead.
## [15:49:41] WARNING: src/c_api/c_api.cc:935: `ntree_limit` is deprecated, use `iteration_range` instead.
## [15:49:41] WARNING: src/c_api/c_api.cc:935: `ntree_limit` is deprecated, use `iteration_range` instead.
## [15:49:42] WARNING: src/c_api/c_api.cc:935: `ntree_limit` is deprecated, use `iteration_range` instead.
## [15:49:42] WARNING: src/c_api/c_api.cc:935: `ntree_limit` is deprecated, use `iteration_range` instead.
## [15:49:42] WARNING: src/c_api/c_api.cc:935: `ntree_limit` is deprecated, use `iteration_range` instead.
## [15:49:42] WARNING: src/c_api/c_api.cc:935: `ntree_limit` is deprecated, use `iteration_range` instead.
## [15:49:42] WARNING: src/c_api/c_api.cc:935: `ntree_limit` is deprecated, use `iteration_range` instead.
## [15:49:42] WARNING: src/c_api/c_api.cc:935: `ntree_limit` is deprecated, use `iteration_range` instead.
## [15:49:42] WARNING: src/c_api/c_api.cc:935: `ntree_limit` is deprecated, use `iteration_range` instead.
## [15:49:42] WARNING: src/c_api/c_api.cc:935: `ntree_limit` is deprecated, use `iteration_range` instead.
## [15:49:42] WARNING: src/c_api/c_api.cc:935: `ntree_limit` is deprecated, use `iteration_range` instead.
## [15:49:42] WARNING: src/c_api/c_api.cc:935: `ntree_limit` is deprecated, use `iteration_range` instead.
## [15:49:43] WARNING: src/c_api/c_api.cc:935: `ntree_limit` is deprecated, use `iteration_range` instead.
## [15:49:43] WARNING: src/c_api/c_api.cc:935: `ntree_limit` is deprecated, use `iteration_range` instead.
## [15:49:43] WARNING: src/c_api/c_api.cc:935: `ntree_limit` is deprecated, use `iteration_range` instead.
## [15:49:43] WARNING: src/c_api/c_api.cc:935: `ntree_limit` is deprecated, use `iteration_range` instead.
## [15:49:43] WARNING: src/c_api/c_api.cc:935: `ntree_limit` is deprecated, use `iteration_range` instead.
## [15:49:43] WARNING: src/c_api/c_api.cc:935: `ntree_limit` is deprecated, use `iteration_range` instead.
## [15:49:43] WARNING: src/c_api/c_api.cc:935: `ntree_limit` is deprecated, use `iteration_range` instead.
## [15:49:43] WARNING: src/c_api/c_api.cc:935: `ntree_limit` is deprecated, use `iteration_range` instead.
## [15:49:43] WARNING: src/c_api/c_api.cc:935: `ntree_limit` is deprecated, use `iteration_range` instead.
## [15:49:43] WARNING: src/c_api/c_api.cc:935: `ntree_limit` is deprecated, use `iteration_range` instead.
## [15:49:43] WARNING: src/c_api/c_api.cc:935: `ntree_limit` is deprecated, use `iteration_range` instead.
## [15:49:43] WARNING: src/c_api/c_api.cc:935: `ntree_limit` is deprecated, use `iteration_range` instead.
## [15:49:43] WARNING: src/c_api/c_api.cc:935: `ntree_limit` is deprecated, use `iteration_range` instead.
## [15:49:43] WARNING: src/c_api/c_api.cc:935: `ntree_limit` is deprecated, use `iteration_range` instead.
## [15:49:43] WARNING: src/c_api/c_api.cc:935: `ntree_limit` is deprecated, use `iteration_range` instead.
## [15:49:43] WARNING: src/c_api/c_api.cc:935: `ntree_limit` is deprecated, use `iteration_range` instead.
## [15:49:44] WARNING: src/c_api/c_api.cc:935: `ntree_limit` is deprecated, use `iteration_range` instead.
## [15:49:44] WARNING: src/c_api/c_api.cc:935: `ntree_limit` is deprecated, use `iteration_range` instead.
## [15:49:44] WARNING: src/c_api/c_api.cc:935: `ntree_limit` is deprecated, use `iteration_range` instead.
## [15:49:44] WARNING: src/c_api/c_api.cc:935: `ntree_limit` is deprecated, use `iteration_range` instead.
## [15:49:44] WARNING: src/c_api/c_api.cc:935: `ntree_limit` is deprecated, use `iteration_range` instead.
## [15:49:44] WARNING: src/c_api/c_api.cc:935: `ntree_limit` is deprecated, use `iteration_range` instead.
## [15:49:44] WARNING: src/c_api/c_api.cc:935: `ntree_limit` is deprecated, use `iteration_range` instead.
## [15:49:44] WARNING: src/c_api/c_api.cc:935: `ntree_limit` is deprecated, use `iteration_range` instead.
## [15:49:44] WARNING: src/c_api/c_api.cc:935: `ntree_limit` is deprecated, use `iteration_range` instead.
## [15:49:44] WARNING: src/c_api/c_api.cc:935: `ntree_limit` is deprecated, use `iteration_range` instead.
## [15:49:44] WARNING: src/c_api/c_api.cc:935: `ntree_limit` is deprecated, use `iteration_range` instead.
## [15:49:44] WARNING: src/c_api/c_api.cc:935: `ntree_limit` is deprecated, use `iteration_range` instead.
## [15:49:45] WARNING: src/c_api/c_api.cc:935: `ntree_limit` is deprecated, use `iteration_range` instead.
## [15:49:45] WARNING: src/c_api/c_api.cc:935: `ntree_limit` is deprecated, use `iteration_range` instead.
## [15:49:45] WARNING: src/c_api/c_api.cc:935: `ntree_limit` is deprecated, use `iteration_range` instead.
## [15:49:45] WARNING: src/c_api/c_api.cc:935: `ntree_limit` is deprecated, use `iteration_range` instead.
## [15:49:45] WARNING: src/c_api/c_api.cc:935: `ntree_limit` is deprecated, use `iteration_range` instead.
## [15:49:45] WARNING: src/c_api/c_api.cc:935: `ntree_limit` is deprecated, use `iteration_range` instead.
## [15:49:45] WARNING: src/c_api/c_api.cc:935: `ntree_limit` is deprecated, use `iteration_range` instead.
## [15:49:45] WARNING: src/c_api/c_api.cc:935: `ntree_limit` is deprecated, use `iteration_range` instead.
## [15:49:45] WARNING: src/c_api/c_api.cc:935: `ntree_limit` is deprecated, use `iteration_range` instead.
## [15:49:45] WARNING: src/c_api/c_api.cc:935: `ntree_limit` is deprecated, use `iteration_range` instead.
## [15:49:46] WARNING: src/c_api/c_api.cc:935: `ntree_limit` is deprecated, use `iteration_range` instead.
## [15:49:46] WARNING: src/c_api/c_api.cc:935: `ntree_limit` is deprecated, use `iteration_range` instead.
## [15:49:46] WARNING: src/c_api/c_api.cc:935: `ntree_limit` is deprecated, use `iteration_range` instead.
## [15:49:46] WARNING: src/c_api/c_api.cc:935: `ntree_limit` is deprecated, use `iteration_range` instead.
## [15:49:46] WARNING: src/c_api/c_api.cc:935: `ntree_limit` is deprecated, use `iteration_range` instead.
## [15:49:46] WARNING: src/c_api/c_api.cc:935: `ntree_limit` is deprecated, use `iteration_range` instead.
## [15:49:46] WARNING: src/c_api/c_api.cc:935: `ntree_limit` is deprecated, use `iteration_range` instead.
## [15:49:46] WARNING: src/c_api/c_api.cc:935: `ntree_limit` is deprecated, use `iteration_range` instead.
## [15:49:46] WARNING: src/c_api/c_api.cc:935: `ntree_limit` is deprecated, use `iteration_range` instead.
## [15:49:46] WARNING: src/c_api/c_api.cc:935: `ntree_limit` is deprecated, use `iteration_range` instead.
## [15:49:46] WARNING: src/c_api/c_api.cc:935: `ntree_limit` is deprecated, use `iteration_range` instead.
## [15:49:46] WARNING: src/c_api/c_api.cc:935: `ntree_limit` is deprecated, use `iteration_range` instead.
## [15:49:46] WARNING: src/c_api/c_api.cc:935: `ntree_limit` is deprecated, use `iteration_range` instead.
## [15:49:46] WARNING: src/c_api/c_api.cc:935: `ntree_limit` is deprecated, use `iteration_range` instead.
## [15:49:46] WARNING: src/c_api/c_api.cc:935: `ntree_limit` is deprecated, use `iteration_range` instead.
## [15:49:46] WARNING: src/c_api/c_api.cc:935: `ntree_limit` is deprecated, use `iteration_range` instead.
## [15:49:47] WARNING: src/c_api/c_api.cc:935: `ntree_limit` is deprecated, use `iteration_range` instead.
## [15:49:47] WARNING: src/c_api/c_api.cc:935: `ntree_limit` is deprecated, use `iteration_range` instead.
## [15:49:47] WARNING: src/c_api/c_api.cc:935: `ntree_limit` is deprecated, use `iteration_range` instead.
## [15:49:47] WARNING: src/c_api/c_api.cc:935: `ntree_limit` is deprecated, use `iteration_range` instead.
## [15:49:47] WARNING: src/c_api/c_api.cc:935: `ntree_limit` is deprecated, use `iteration_range` instead.
## [15:49:47] WARNING: src/c_api/c_api.cc:935: `ntree_limit` is deprecated, use `iteration_range` instead.
## [15:49:47] WARNING: src/c_api/c_api.cc:935: `ntree_limit` is deprecated, use `iteration_range` instead.
## [15:49:47] WARNING: src/c_api/c_api.cc:935: `ntree_limit` is deprecated, use `iteration_range` instead.
## [15:49:47] WARNING: src/c_api/c_api.cc:935: `ntree_limit` is deprecated, use `iteration_range` instead.
## [15:49:47] WARNING: src/c_api/c_api.cc:935: `ntree_limit` is deprecated, use `iteration_range` instead.
## [15:49:47] WARNING: src/c_api/c_api.cc:935: `ntree_limit` is deprecated, use `iteration_range` instead.
## [15:49:47] WARNING: src/c_api/c_api.cc:935: `ntree_limit` is deprecated, use `iteration_range` instead.
## [15:49:48] WARNING: src/c_api/c_api.cc:935: `ntree_limit` is deprecated, use `iteration_range` instead.
## [15:49:48] WARNING: src/c_api/c_api.cc:935: `ntree_limit` is deprecated, use `iteration_range` instead.
## [15:49:48] WARNING: src/c_api/c_api.cc:935: `ntree_limit` is deprecated, use `iteration_range` instead.
## [15:49:48] WARNING: src/c_api/c_api.cc:935: `ntree_limit` is deprecated, use `iteration_range` instead.
## [15:49:48] WARNING: src/c_api/c_api.cc:935: `ntree_limit` is deprecated, use `iteration_range` instead.
## [15:49:48] WARNING: src/c_api/c_api.cc:935: `ntree_limit` is deprecated, use `iteration_range` instead.
## [15:49:48] WARNING: src/c_api/c_api.cc:935: `ntree_limit` is deprecated, use `iteration_range` instead.
## [15:49:48] WARNING: src/c_api/c_api.cc:935: `ntree_limit` is deprecated, use `iteration_range` instead.
## [15:49:48] WARNING: src/c_api/c_api.cc:935: `ntree_limit` is deprecated, use `iteration_range` instead.
## [15:49:48] WARNING: src/c_api/c_api.cc:935: `ntree_limit` is deprecated, use `iteration_range` instead.
## [15:49:51] WARNING: src/c_api/c_api.cc:935: `ntree_limit` is deprecated, use `iteration_range` instead.
## [15:49:51] WARNING: src/c_api/c_api.cc:935: `ntree_limit` is deprecated, use `iteration_range` instead.
## [15:49:51] WARNING: src/c_api/c_api.cc:935: `ntree_limit` is deprecated, use `iteration_range` instead.
## [15:49:51] WARNING: src/c_api/c_api.cc:935: `ntree_limit` is deprecated, use `iteration_range` instead.
## [15:49:51] WARNING: src/c_api/c_api.cc:935: `ntree_limit` is deprecated, use `iteration_range` instead.
## [15:49:51] WARNING: src/c_api/c_api.cc:935: `ntree_limit` is deprecated, use `iteration_range` instead.
## [15:49:51] WARNING: src/c_api/c_api.cc:935: `ntree_limit` is deprecated, use `iteration_range` instead.
## [15:49:51] WARNING: src/c_api/c_api.cc:935: `ntree_limit` is deprecated, use `iteration_range` instead.
## [15:49:52] WARNING: src/c_api/c_api.cc:935: `ntree_limit` is deprecated, use `iteration_range` instead.
## [15:49:52] WARNING: src/c_api/c_api.cc:935: `ntree_limit` is deprecated, use `iteration_range` instead.
## [15:49:52] WARNING: src/c_api/c_api.cc:935: `ntree_limit` is deprecated, use `iteration_range` instead.
## [15:49:52] WARNING: src/c_api/c_api.cc:935: `ntree_limit` is deprecated, use `iteration_range` instead.
## [15:49:52] WARNING: src/c_api/c_api.cc:935: `ntree_limit` is deprecated, use `iteration_range` instead.
## [15:49:52] WARNING: src/c_api/c_api.cc:935: `ntree_limit` is deprecated, use `iteration_range` instead.
## [15:49:52] WARNING: src/c_api/c_api.cc:935: `ntree_limit` is deprecated, use `iteration_range` instead.
## [15:49:52] WARNING: src/c_api/c_api.cc:935: `ntree_limit` is deprecated, use `iteration_range` instead.
## [15:49:52] WARNING: src/c_api/c_api.cc:935: `ntree_limit` is deprecated, use `iteration_range` instead.
## [15:49:52] WARNING: src/c_api/c_api.cc:935: `ntree_limit` is deprecated, use `iteration_range` instead.
## [15:49:52] WARNING: src/c_api/c_api.cc:935: `ntree_limit` is deprecated, use `iteration_range` instead.
## [15:49:52] WARNING: src/c_api/c_api.cc:935: `ntree_limit` is deprecated, use `iteration_range` instead.
## [15:49:52] WARNING: src/c_api/c_api.cc:935: `ntree_limit` is deprecated, use `iteration_range` instead.
## [15:49:52] WARNING: src/c_api/c_api.cc:935: `ntree_limit` is deprecated, use `iteration_range` instead.
## [15:49:53] WARNING: src/c_api/c_api.cc:935: `ntree_limit` is deprecated, use `iteration_range` instead.
## [15:49:53] WARNING: src/c_api/c_api.cc:935: `ntree_limit` is deprecated, use `iteration_range` instead.
## [15:49:53] WARNING: src/c_api/c_api.cc:935: `ntree_limit` is deprecated, use `iteration_range` instead.
## [15:49:53] WARNING: src/c_api/c_api.cc:935: `ntree_limit` is deprecated, use `iteration_range` instead.
## [15:49:53] WARNING: src/c_api/c_api.cc:935: `ntree_limit` is deprecated, use `iteration_range` instead.
## [15:49:53] WARNING: src/c_api/c_api.cc:935: `ntree_limit` is deprecated, use `iteration_range` instead.
## [15:49:53] WARNING: src/c_api/c_api.cc:935: `ntree_limit` is deprecated, use `iteration_range` instead.
## [15:49:53] WARNING: src/c_api/c_api.cc:935: `ntree_limit` is deprecated, use `iteration_range` instead.
## [15:49:54] WARNING: src/c_api/c_api.cc:935: `ntree_limit` is deprecated, use `iteration_range` instead.
## [15:49:54] WARNING: src/c_api/c_api.cc:935: `ntree_limit` is deprecated, use `iteration_range` instead.
## [15:49:54] WARNING: src/c_api/c_api.cc:935: `ntree_limit` is deprecated, use `iteration_range` instead.
## [15:49:54] WARNING: src/c_api/c_api.cc:935: `ntree_limit` is deprecated, use `iteration_range` instead.
## [15:49:54] WARNING: src/c_api/c_api.cc:935: `ntree_limit` is deprecated, use `iteration_range` instead.
## [15:49:54] WARNING: src/c_api/c_api.cc:935: `ntree_limit` is deprecated, use `iteration_range` instead.
## [15:49:54] WARNING: src/c_api/c_api.cc:935: `ntree_limit` is deprecated, use `iteration_range` instead.
## [15:49:54] WARNING: src/c_api/c_api.cc:935: `ntree_limit` is deprecated, use `iteration_range` instead.
## [15:49:54] WARNING: src/c_api/c_api.cc:935: `ntree_limit` is deprecated, use `iteration_range` instead.
## [15:49:54] WARNING: src/c_api/c_api.cc:935: `ntree_limit` is deprecated, use `iteration_range` instead.
## [15:49:54] WARNING: src/c_api/c_api.cc:935: `ntree_limit` is deprecated, use `iteration_range` instead.
## [15:49:54] WARNING: src/c_api/c_api.cc:935: `ntree_limit` is deprecated, use `iteration_range` instead.
## [15:49:54] WARNING: src/c_api/c_api.cc:935: `ntree_limit` is deprecated, use `iteration_range` instead.
## [15:49:54] WARNING: src/c_api/c_api.cc:935: `ntree_limit` is deprecated, use `iteration_range` instead.
## [15:49:55] WARNING: src/c_api/c_api.cc:935: `ntree_limit` is deprecated, use `iteration_range` instead.
## [15:49:55] WARNING: src/c_api/c_api.cc:935: `ntree_limit` is deprecated, use `iteration_range` instead.
## [15:49:55] WARNING: src/c_api/c_api.cc:935: `ntree_limit` is deprecated, use `iteration_range` instead.
## [15:49:55] WARNING: src/c_api/c_api.cc:935: `ntree_limit` is deprecated, use `iteration_range` instead.
## [15:49:55] WARNING: src/c_api/c_api.cc:935: `ntree_limit` is deprecated, use `iteration_range` instead.
## [15:49:55] WARNING: src/c_api/c_api.cc:935: `ntree_limit` is deprecated, use `iteration_range` instead.
## [15:49:55] WARNING: src/c_api/c_api.cc:935: `ntree_limit` is deprecated, use `iteration_range` instead.
## [15:49:55] WARNING: src/c_api/c_api.cc:935: `ntree_limit` is deprecated, use `iteration_range` instead.
## [15:49:55] WARNING: src/c_api/c_api.cc:935: `ntree_limit` is deprecated, use `iteration_range` instead.
## [15:49:55] WARNING: src/c_api/c_api.cc:935: `ntree_limit` is deprecated, use `iteration_range` instead.
## [15:49:55] WARNING: src/c_api/c_api.cc:935: `ntree_limit` is deprecated, use `iteration_range` instead.
## [15:49:55] WARNING: src/c_api/c_api.cc:935: `ntree_limit` is deprecated, use `iteration_range` instead.
## [15:49:56] WARNING: src/c_api/c_api.cc:935: `ntree_limit` is deprecated, use `iteration_range` instead.
## [15:49:56] WARNING: src/c_api/c_api.cc:935: `ntree_limit` is deprecated, use `iteration_range` instead.
## [15:49:56] WARNING: src/c_api/c_api.cc:935: `ntree_limit` is deprecated, use `iteration_range` instead.
## [15:49:56] WARNING: src/c_api/c_api.cc:935: `ntree_limit` is deprecated, use `iteration_range` instead.
## [15:49:56] WARNING: src/c_api/c_api.cc:935: `ntree_limit` is deprecated, use `iteration_range` instead.
## [15:49:56] WARNING: src/c_api/c_api.cc:935: `ntree_limit` is deprecated, use `iteration_range` instead.
## [15:49:56] WARNING: src/c_api/c_api.cc:935: `ntree_limit` is deprecated, use `iteration_range` instead.
## [15:49:56] WARNING: src/c_api/c_api.cc:935: `ntree_limit` is deprecated, use `iteration_range` instead.
## [15:49:56] WARNING: src/c_api/c_api.cc:935: `ntree_limit` is deprecated, use `iteration_range` instead.
## [15:49:56] WARNING: src/c_api/c_api.cc:935: `ntree_limit` is deprecated, use `iteration_range` instead.
## [15:49:57] WARNING: src/c_api/c_api.cc:935: `ntree_limit` is deprecated, use `iteration_range` instead.
## [15:49:57] WARNING: src/c_api/c_api.cc:935: `ntree_limit` is deprecated, use `iteration_range` instead.
## [15:49:57] WARNING: src/c_api/c_api.cc:935: `ntree_limit` is deprecated, use `iteration_range` instead.
## [15:49:57] WARNING: src/c_api/c_api.cc:935: `ntree_limit` is deprecated, use `iteration_range` instead.
## [15:49:57] WARNING: src/c_api/c_api.cc:935: `ntree_limit` is deprecated, use `iteration_range` instead.
## [15:49:57] WARNING: src/c_api/c_api.cc:935: `ntree_limit` is deprecated, use `iteration_range` instead.
## [15:49:57] WARNING: src/c_api/c_api.cc:935: `ntree_limit` is deprecated, use `iteration_range` instead.
## [15:49:57] WARNING: src/c_api/c_api.cc:935: `ntree_limit` is deprecated, use `iteration_range` instead.
## [15:49:57] WARNING: src/c_api/c_api.cc:935: `ntree_limit` is deprecated, use `iteration_range` instead.
## [15:49:57] WARNING: src/c_api/c_api.cc:935: `ntree_limit` is deprecated, use `iteration_range` instead.
## [15:49:57] WARNING: src/c_api/c_api.cc:935: `ntree_limit` is deprecated, use `iteration_range` instead.
## [15:49:57] WARNING: src/c_api/c_api.cc:935: `ntree_limit` is deprecated, use `iteration_range` instead.
## [15:49:57] WARNING: src/c_api/c_api.cc:935: `ntree_limit` is deprecated, use `iteration_range` instead.
## [15:49:57] WARNING: src/c_api/c_api.cc:935: `ntree_limit` is deprecated, use `iteration_range` instead.
## [15:49:58] WARNING: src/c_api/c_api.cc:935: `ntree_limit` is deprecated, use `iteration_range` instead.
## [15:49:58] WARNING: src/c_api/c_api.cc:935: `ntree_limit` is deprecated, use `iteration_range` instead.
## [15:49:58] WARNING: src/c_api/c_api.cc:935: `ntree_limit` is deprecated, use `iteration_range` instead.
## [15:49:58] WARNING: src/c_api/c_api.cc:935: `ntree_limit` is deprecated, use `iteration_range` instead.
## [15:49:58] WARNING: src/c_api/c_api.cc:935: `ntree_limit` is deprecated, use `iteration_range` instead.
## [15:49:58] WARNING: src/c_api/c_api.cc:935: `ntree_limit` is deprecated, use `iteration_range` instead.
## [15:49:58] WARNING: src/c_api/c_api.cc:935: `ntree_limit` is deprecated, use `iteration_range` instead.
## [15:49:58] WARNING: src/c_api/c_api.cc:935: `ntree_limit` is deprecated, use `iteration_range` instead.
## [15:49:58] WARNING: src/c_api/c_api.cc:935: `ntree_limit` is deprecated, use `iteration_range` instead.
## [15:49:58] WARNING: src/c_api/c_api.cc:935: `ntree_limit` is deprecated, use `iteration_range` instead.
## [15:49:58] WARNING: src/c_api/c_api.cc:935: `ntree_limit` is deprecated, use `iteration_range` instead.
## [15:49:58] WARNING: src/c_api/c_api.cc:935: `ntree_limit` is deprecated, use `iteration_range` instead.
## [15:49:59] WARNING: src/c_api/c_api.cc:935: `ntree_limit` is deprecated, use `iteration_range` instead.
## [15:49:59] WARNING: src/c_api/c_api.cc:935: `ntree_limit` is deprecated, use `iteration_range` instead.
## [15:49:59] WARNING: src/c_api/c_api.cc:935: `ntree_limit` is deprecated, use `iteration_range` instead.
## [15:49:59] WARNING: src/c_api/c_api.cc:935: `ntree_limit` is deprecated, use `iteration_range` instead.
## [15:49:59] WARNING: src/c_api/c_api.cc:935: `ntree_limit` is deprecated, use `iteration_range` instead.
## [15:49:59] WARNING: src/c_api/c_api.cc:935: `ntree_limit` is deprecated, use `iteration_range` instead.
## [15:49:59] WARNING: src/c_api/c_api.cc:935: `ntree_limit` is deprecated, use `iteration_range` instead.
## [15:49:59] WARNING: src/c_api/c_api.cc:935: `ntree_limit` is deprecated, use `iteration_range` instead.
## [15:49:59] WARNING: src/c_api/c_api.cc:935: `ntree_limit` is deprecated, use `iteration_range` instead.
## [15:49:59] WARNING: src/c_api/c_api.cc:935: `ntree_limit` is deprecated, use `iteration_range` instead.
## [15:50:00] WARNING: src/c_api/c_api.cc:935: `ntree_limit` is deprecated, use `iteration_range` instead.
## [15:50:00] WARNING: src/c_api/c_api.cc:935: `ntree_limit` is deprecated, use `iteration_range` instead.
## [15:50:00] WARNING: src/c_api/c_api.cc:935: `ntree_limit` is deprecated, use `iteration_range` instead.
## [15:50:00] WARNING: src/c_api/c_api.cc:935: `ntree_limit` is deprecated, use `iteration_range` instead.
## [15:50:00] WARNING: src/c_api/c_api.cc:935: `ntree_limit` is deprecated, use `iteration_range` instead.
## [15:50:00] WARNING: src/c_api/c_api.cc:935: `ntree_limit` is deprecated, use `iteration_range` instead.
## [15:50:00] WARNING: src/c_api/c_api.cc:935: `ntree_limit` is deprecated, use `iteration_range` instead.
## [15:50:00] WARNING: src/c_api/c_api.cc:935: `ntree_limit` is deprecated, use `iteration_range` instead.
## [15:50:00] WARNING: src/c_api/c_api.cc:935: `ntree_limit` is deprecated, use `iteration_range` instead.
## [15:50:00] WARNING: src/c_api/c_api.cc:935: `ntree_limit` is deprecated, use `iteration_range` instead.
## [15:50:00] WARNING: src/c_api/c_api.cc:935: `ntree_limit` is deprecated, use `iteration_range` instead.
## [15:50:00] WARNING: src/c_api/c_api.cc:935: `ntree_limit` is deprecated, use `iteration_range` instead.
## [15:50:01] WARNING: src/c_api/c_api.cc:935: `ntree_limit` is deprecated, use `iteration_range` instead.
## [15:50:01] WARNING: src/c_api/c_api.cc:935: `ntree_limit` is deprecated, use `iteration_range` instead.
## [15:50:01] WARNING: src/c_api/c_api.cc:935: `ntree_limit` is deprecated, use `iteration_range` instead.
## [15:50:01] WARNING: src/c_api/c_api.cc:935: `ntree_limit` is deprecated, use `iteration_range` instead.
## [15:50:01] WARNING: src/c_api/c_api.cc:935: `ntree_limit` is deprecated, use `iteration_range` instead.
## [15:50:01] WARNING: src/c_api/c_api.cc:935: `ntree_limit` is deprecated, use `iteration_range` instead.
## [15:50:01] WARNING: src/c_api/c_api.cc:935: `ntree_limit` is deprecated, use `iteration_range` instead.
## [15:50:01] WARNING: src/c_api/c_api.cc:935: `ntree_limit` is deprecated, use `iteration_range` instead.
## [15:50:01] WARNING: src/c_api/c_api.cc:935: `ntree_limit` is deprecated, use `iteration_range` instead.
## [15:50:01] WARNING: src/c_api/c_api.cc:935: `ntree_limit` is deprecated, use `iteration_range` instead.
## [15:50:01] WARNING: src/c_api/c_api.cc:935: `ntree_limit` is deprecated, use `iteration_range` instead.
## [15:50:01] WARNING: src/c_api/c_api.cc:935: `ntree_limit` is deprecated, use `iteration_range` instead.
## [15:50:02] WARNING: src/c_api/c_api.cc:935: `ntree_limit` is deprecated, use `iteration_range` instead.
## [15:50:02] WARNING: src/c_api/c_api.cc:935: `ntree_limit` is deprecated, use `iteration_range` instead.
## [15:50:02] WARNING: src/c_api/c_api.cc:935: `ntree_limit` is deprecated, use `iteration_range` instead.
## [15:50:02] WARNING: src/c_api/c_api.cc:935: `ntree_limit` is deprecated, use `iteration_range` instead.
## [15:50:02] WARNING: src/c_api/c_api.cc:935: `ntree_limit` is deprecated, use `iteration_range` instead.
## [15:50:02] WARNING: src/c_api/c_api.cc:935: `ntree_limit` is deprecated, use `iteration_range` instead.
## [15:50:02] WARNING: src/c_api/c_api.cc:935: `ntree_limit` is deprecated, use `iteration_range` instead.
## [15:50:02] WARNING: src/c_api/c_api.cc:935: `ntree_limit` is deprecated, use `iteration_range` instead.
## [15:50:02] WARNING: src/c_api/c_api.cc:935: `ntree_limit` is deprecated, use `iteration_range` instead.
## [15:50:02] WARNING: src/c_api/c_api.cc:935: `ntree_limit` is deprecated, use `iteration_range` instead.
## [15:50:02] WARNING: src/c_api/c_api.cc:935: `ntree_limit` is deprecated, use `iteration_range` instead.
## [15:50:02] WARNING: src/c_api/c_api.cc:935: `ntree_limit` is deprecated, use `iteration_range` instead.
## [15:50:03] WARNING: src/c_api/c_api.cc:935: `ntree_limit` is deprecated, use `iteration_range` instead.
## [15:50:03] WARNING: src/c_api/c_api.cc:935: `ntree_limit` is deprecated, use `iteration_range` instead.
## [15:50:03] WARNING: src/c_api/c_api.cc:935: `ntree_limit` is deprecated, use `iteration_range` instead.
## [15:50:03] WARNING: src/c_api/c_api.cc:935: `ntree_limit` is deprecated, use `iteration_range` instead.
## [15:50:03] WARNING: src/c_api/c_api.cc:935: `ntree_limit` is deprecated, use `iteration_range` instead.
## [15:50:03] WARNING: src/c_api/c_api.cc:935: `ntree_limit` is deprecated, use `iteration_range` instead.
## [15:50:03] WARNING: src/c_api/c_api.cc:935: `ntree_limit` is deprecated, use `iteration_range` instead.
## [15:50:03] WARNING: src/c_api/c_api.cc:935: `ntree_limit` is deprecated, use `iteration_range` instead.
## [15:50:03] WARNING: src/c_api/c_api.cc:935: `ntree_limit` is deprecated, use `iteration_range` instead.
## [15:50:03] WARNING: src/c_api/c_api.cc:935: `ntree_limit` is deprecated, use `iteration_range` instead.
## [15:50:03] WARNING: src/c_api/c_api.cc:935: `ntree_limit` is deprecated, use `iteration_range` instead.
## [15:50:03] WARNING: src/c_api/c_api.cc:935: `ntree_limit` is deprecated, use `iteration_range` instead.
## [15:50:04] WARNING: src/c_api/c_api.cc:935: `ntree_limit` is deprecated, use `iteration_range` instead.
## [15:50:04] WARNING: src/c_api/c_api.cc:935: `ntree_limit` is deprecated, use `iteration_range` instead.
## [15:50:04] WARNING: src/c_api/c_api.cc:935: `ntree_limit` is deprecated, use `iteration_range` instead.
## [15:50:04] WARNING: src/c_api/c_api.cc:935: `ntree_limit` is deprecated, use `iteration_range` instead.
## [15:50:04] WARNING: src/c_api/c_api.cc:935: `ntree_limit` is deprecated, use `iteration_range` instead.
## [15:50:04] WARNING: src/c_api/c_api.cc:935: `ntree_limit` is deprecated, use `iteration_range` instead.
## [15:50:04] WARNING: src/c_api/c_api.cc:935: `ntree_limit` is deprecated, use `iteration_range` instead.
## [15:50:04] WARNING: src/c_api/c_api.cc:935: `ntree_limit` is deprecated, use `iteration_range` instead.
## [15:50:04] WARNING: src/c_api/c_api.cc:935: `ntree_limit` is deprecated, use `iteration_range` instead.
## [15:50:04] WARNING: src/c_api/c_api.cc:935: `ntree_limit` is deprecated, use `iteration_range` instead.
## [15:50:04] WARNING: src/c_api/c_api.cc:935: `ntree_limit` is deprecated, use `iteration_range` instead.
## [15:50:04] WARNING: src/c_api/c_api.cc:935: `ntree_limit` is deprecated, use `iteration_range` instead.
## [15:50:05] WARNING: src/c_api/c_api.cc:935: `ntree_limit` is deprecated, use `iteration_range` instead.
## [15:50:05] WARNING: src/c_api/c_api.cc:935: `ntree_limit` is deprecated, use `iteration_range` instead.
## [15:50:05] WARNING: src/c_api/c_api.cc:935: `ntree_limit` is deprecated, use `iteration_range` instead.
## [15:50:05] WARNING: src/c_api/c_api.cc:935: `ntree_limit` is deprecated, use `iteration_range` instead.
## [15:50:05] WARNING: src/c_api/c_api.cc:935: `ntree_limit` is deprecated, use `iteration_range` instead.
## [15:50:05] WARNING: src/c_api/c_api.cc:935: `ntree_limit` is deprecated, use `iteration_range` instead.
## [15:50:05] WARNING: src/c_api/c_api.cc:935: `ntree_limit` is deprecated, use `iteration_range` instead.
## [15:50:05] WARNING: src/c_api/c_api.cc:935: `ntree_limit` is deprecated, use `iteration_range` instead.
## [15:50:05] WARNING: src/c_api/c_api.cc:935: `ntree_limit` is deprecated, use `iteration_range` instead.
## [15:50:05] WARNING: src/c_api/c_api.cc:935: `ntree_limit` is deprecated, use `iteration_range` instead.
## [15:50:06] WARNING: src/c_api/c_api.cc:935: `ntree_limit` is deprecated, use `iteration_range` instead.
## [15:50:06] WARNING: src/c_api/c_api.cc:935: `ntree_limit` is deprecated, use `iteration_range` instead.
## [15:50:06] WARNING: src/c_api/c_api.cc:935: `ntree_limit` is deprecated, use `iteration_range` instead.
## [15:50:06] WARNING: src/c_api/c_api.cc:935: `ntree_limit` is deprecated, use `iteration_range` instead.
## [15:50:06] WARNING: src/c_api/c_api.cc:935: `ntree_limit` is deprecated, use `iteration_range` instead.
## [15:50:06] WARNING: src/c_api/c_api.cc:935: `ntree_limit` is deprecated, use `iteration_range` instead.
## [15:50:06] WARNING: src/c_api/c_api.cc:935: `ntree_limit` is deprecated, use `iteration_range` instead.
## [15:50:06] WARNING: src/c_api/c_api.cc:935: `ntree_limit` is deprecated, use `iteration_range` instead.
## [15:50:06] WARNING: src/c_api/c_api.cc:935: `ntree_limit` is deprecated, use `iteration_range` instead.
## [15:50:06] WARNING: src/c_api/c_api.cc:935: `ntree_limit` is deprecated, use `iteration_range` instead.
## [15:50:07] WARNING: src/c_api/c_api.cc:935: `ntree_limit` is deprecated, use `iteration_range` instead.
## [15:50:07] WARNING: src/c_api/c_api.cc:935: `ntree_limit` is deprecated, use `iteration_range` instead.
## [15:50:07] WARNING: src/c_api/c_api.cc:935: `ntree_limit` is deprecated, use `iteration_range` instead.
## [15:50:07] WARNING: src/c_api/c_api.cc:935: `ntree_limit` is deprecated, use `iteration_range` instead.
## [15:50:07] WARNING: src/c_api/c_api.cc:935: `ntree_limit` is deprecated, use `iteration_range` instead.
## [15:50:07] WARNING: src/c_api/c_api.cc:935: `ntree_limit` is deprecated, use `iteration_range` instead.
## [15:50:07] WARNING: src/c_api/c_api.cc:935: `ntree_limit` is deprecated, use `iteration_range` instead.
## [15:50:07] WARNING: src/c_api/c_api.cc:935: `ntree_limit` is deprecated, use `iteration_range` instead.
## [15:50:07] WARNING: src/c_api/c_api.cc:935: `ntree_limit` is deprecated, use `iteration_range` instead.
## [15:50:07] WARNING: src/c_api/c_api.cc:935: `ntree_limit` is deprecated, use `iteration_range` instead.
## [15:50:07] WARNING: src/c_api/c_api.cc:935: `ntree_limit` is deprecated, use `iteration_range` instead.
## [15:50:07] WARNING: src/c_api/c_api.cc:935: `ntree_limit` is deprecated, use `iteration_range` instead.
## [15:50:07] WARNING: src/c_api/c_api.cc:935: `ntree_limit` is deprecated, use `iteration_range` instead.
## [15:50:07] WARNING: src/c_api/c_api.cc:935: `ntree_limit` is deprecated, use `iteration_range` instead.
## [15:50:08] WARNING: src/c_api/c_api.cc:935: `ntree_limit` is deprecated, use `iteration_range` instead.
## [15:50:08] WARNING: src/c_api/c_api.cc:935: `ntree_limit` is deprecated, use `iteration_range` instead.
## [15:50:08] WARNING: src/c_api/c_api.cc:935: `ntree_limit` is deprecated, use `iteration_range` instead.
## [15:50:08] WARNING: src/c_api/c_api.cc:935: `ntree_limit` is deprecated, use `iteration_range` instead.
## [15:50:08] WARNING: src/c_api/c_api.cc:935: `ntree_limit` is deprecated, use `iteration_range` instead.
## [15:50:08] WARNING: src/c_api/c_api.cc:935: `ntree_limit` is deprecated, use `iteration_range` instead.
## [15:50:08] WARNING: src/c_api/c_api.cc:935: `ntree_limit` is deprecated, use `iteration_range` instead.
## [15:50:08] WARNING: src/c_api/c_api.cc:935: `ntree_limit` is deprecated, use `iteration_range` instead.
## [15:50:08] WARNING: src/c_api/c_api.cc:935: `ntree_limit` is deprecated, use `iteration_range` instead.
## [15:50:08] WARNING: src/c_api/c_api.cc:935: `ntree_limit` is deprecated, use `iteration_range` instead.
## [15:50:08] WARNING: src/c_api/c_api.cc:935: `ntree_limit` is deprecated, use `iteration_range` instead.
## [15:50:08] WARNING: src/c_api/c_api.cc:935: `ntree_limit` is deprecated, use `iteration_range` instead.
## [15:50:09] WARNING: src/c_api/c_api.cc:935: `ntree_limit` is deprecated, use `iteration_range` instead.
## [15:50:09] WARNING: src/c_api/c_api.cc:935: `ntree_limit` is deprecated, use `iteration_range` instead.
## [15:50:09] WARNING: src/c_api/c_api.cc:935: `ntree_limit` is deprecated, use `iteration_range` instead.
## [15:50:09] WARNING: src/c_api/c_api.cc:935: `ntree_limit` is deprecated, use `iteration_range` instead.
## [15:50:09] WARNING: src/c_api/c_api.cc:935: `ntree_limit` is deprecated, use `iteration_range` instead.
## [15:50:09] WARNING: src/c_api/c_api.cc:935: `ntree_limit` is deprecated, use `iteration_range` instead.
## [15:50:09] WARNING: src/c_api/c_api.cc:935: `ntree_limit` is deprecated, use `iteration_range` instead.
## [15:50:09] WARNING: src/c_api/c_api.cc:935: `ntree_limit` is deprecated, use `iteration_range` instead.
## [15:50:09] WARNING: src/c_api/c_api.cc:935: `ntree_limit` is deprecated, use `iteration_range` instead.
## [15:50:09] WARNING: src/c_api/c_api.cc:935: `ntree_limit` is deprecated, use `iteration_range` instead.
## [15:50:10] WARNING: src/c_api/c_api.cc:935: `ntree_limit` is deprecated, use `iteration_range` instead.
## [15:50:10] WARNING: src/c_api/c_api.cc:935: `ntree_limit` is deprecated, use `iteration_range` instead.
## [15:50:10] WARNING: src/c_api/c_api.cc:935: `ntree_limit` is deprecated, use `iteration_range` instead.
## [15:50:10] WARNING: src/c_api/c_api.cc:935: `ntree_limit` is deprecated, use `iteration_range` instead.
## [15:50:10] WARNING: src/c_api/c_api.cc:935: `ntree_limit` is deprecated, use `iteration_range` instead.
## [15:50:10] WARNING: src/c_api/c_api.cc:935: `ntree_limit` is deprecated, use `iteration_range` instead.
## [15:50:10] WARNING: src/c_api/c_api.cc:935: `ntree_limit` is deprecated, use `iteration_range` instead.
## [15:50:10] WARNING: src/c_api/c_api.cc:935: `ntree_limit` is deprecated, use `iteration_range` instead.
## [15:50:10] WARNING: src/c_api/c_api.cc:935: `ntree_limit` is deprecated, use `iteration_range` instead.
## [15:50:10] WARNING: src/c_api/c_api.cc:935: `ntree_limit` is deprecated, use `iteration_range` instead.
## [15:50:10] WARNING: src/c_api/c_api.cc:935: `ntree_limit` is deprecated, use `iteration_range` instead.
## [15:50:10] WARNING: src/c_api/c_api.cc:935: `ntree_limit` is deprecated, use `iteration_range` instead.
## [15:50:10] WARNING: src/c_api/c_api.cc:935: `ntree_limit` is deprecated, use `iteration_range` instead.
## [15:50:10] WARNING: src/c_api/c_api.cc:935: `ntree_limit` is deprecated, use `iteration_range` instead.
## [15:50:11] WARNING: src/c_api/c_api.cc:935: `ntree_limit` is deprecated, use `iteration_range` instead.
## [15:50:11] WARNING: src/c_api/c_api.cc:935: `ntree_limit` is deprecated, use `iteration_range` instead.
## [15:50:11] WARNING: src/c_api/c_api.cc:935: `ntree_limit` is deprecated, use `iteration_range` instead.
## [15:50:11] WARNING: src/c_api/c_api.cc:935: `ntree_limit` is deprecated, use `iteration_range` instead.
## [15:50:11] WARNING: src/c_api/c_api.cc:935: `ntree_limit` is deprecated, use `iteration_range` instead.
## [15:50:11] WARNING: src/c_api/c_api.cc:935: `ntree_limit` is deprecated, use `iteration_range` instead.
## [15:50:11] WARNING: src/c_api/c_api.cc:935: `ntree_limit` is deprecated, use `iteration_range` instead.
## [15:50:11] WARNING: src/c_api/c_api.cc:935: `ntree_limit` is deprecated, use `iteration_range` instead.
## [15:50:11] WARNING: src/c_api/c_api.cc:935: `ntree_limit` is deprecated, use `iteration_range` instead.
## [15:50:11] WARNING: src/c_api/c_api.cc:935: `ntree_limit` is deprecated, use `iteration_range` instead.
## [15:50:12] WARNING: src/c_api/c_api.cc:935: `ntree_limit` is deprecated, use `iteration_range` instead.
## [15:50:12] WARNING: src/c_api/c_api.cc:935: `ntree_limit` is deprecated, use `iteration_range` instead.
## [15:50:12] WARNING: src/c_api/c_api.cc:935: `ntree_limit` is deprecated, use `iteration_range` instead.
## [15:50:12] WARNING: src/c_api/c_api.cc:935: `ntree_limit` is deprecated, use `iteration_range` instead.
## [15:50:12] WARNING: src/c_api/c_api.cc:935: `ntree_limit` is deprecated, use `iteration_range` instead.
## [15:50:12] WARNING: src/c_api/c_api.cc:935: `ntree_limit` is deprecated, use `iteration_range` instead.
## [15:50:12] WARNING: src/c_api/c_api.cc:935: `ntree_limit` is deprecated, use `iteration_range` instead.
## [15:50:12] WARNING: src/c_api/c_api.cc:935: `ntree_limit` is deprecated, use `iteration_range` instead.
## [15:50:12] WARNING: src/c_api/c_api.cc:935: `ntree_limit` is deprecated, use `iteration_range` instead.
## [15:50:12] WARNING: src/c_api/c_api.cc:935: `ntree_limit` is deprecated, use `iteration_range` instead.
## [15:50:13] WARNING: src/c_api/c_api.cc:935: `ntree_limit` is deprecated, use `iteration_range` instead.
## [15:50:13] WARNING: src/c_api/c_api.cc:935: `ntree_limit` is deprecated, use `iteration_range` instead.
## [15:50:13] WARNING: src/c_api/c_api.cc:935: `ntree_limit` is deprecated, use `iteration_range` instead.
## [15:50:13] WARNING: src/c_api/c_api.cc:935: `ntree_limit` is deprecated, use `iteration_range` instead.
## [15:50:13] WARNING: src/c_api/c_api.cc:935: `ntree_limit` is deprecated, use `iteration_range` instead.
## [15:50:13] WARNING: src/c_api/c_api.cc:935: `ntree_limit` is deprecated, use `iteration_range` instead.
## [15:50:13] WARNING: src/c_api/c_api.cc:935: `ntree_limit` is deprecated, use `iteration_range` instead.
## [15:50:13] WARNING: src/c_api/c_api.cc:935: `ntree_limit` is deprecated, use `iteration_range` instead.
## [15:50:13] WARNING: src/c_api/c_api.cc:935: `ntree_limit` is deprecated, use `iteration_range` instead.
## [15:50:13] WARNING: src/c_api/c_api.cc:935: `ntree_limit` is deprecated, use `iteration_range` instead.
## [15:50:13] WARNING: src/c_api/c_api.cc:935: `ntree_limit` is deprecated, use `iteration_range` instead.
## [15:50:13] WARNING: src/c_api/c_api.cc:935: `ntree_limit` is deprecated, use `iteration_range` instead.
## [15:50:13] WARNING: src/c_api/c_api.cc:935: `ntree_limit` is deprecated, use `iteration_range` instead.
## [15:50:13] WARNING: src/c_api/c_api.cc:935: `ntree_limit` is deprecated, use `iteration_range` instead.
## [15:50:14] WARNING: src/c_api/c_api.cc:935: `ntree_limit` is deprecated, use `iteration_range` instead.
## [15:50:14] WARNING: src/c_api/c_api.cc:935: `ntree_limit` is deprecated, use `iteration_range` instead.
## [15:50:14] WARNING: src/c_api/c_api.cc:935: `ntree_limit` is deprecated, use `iteration_range` instead.
## [15:50:14] WARNING: src/c_api/c_api.cc:935: `ntree_limit` is deprecated, use `iteration_range` instead.
## [15:50:14] WARNING: src/c_api/c_api.cc:935: `ntree_limit` is deprecated, use `iteration_range` instead.
## [15:50:14] WARNING: src/c_api/c_api.cc:935: `ntree_limit` is deprecated, use `iteration_range` instead.
## [15:50:14] WARNING: src/c_api/c_api.cc:935: `ntree_limit` is deprecated, use `iteration_range` instead.
## [15:50:14] WARNING: src/c_api/c_api.cc:935: `ntree_limit` is deprecated, use `iteration_range` instead.
## [15:50:14] WARNING: src/c_api/c_api.cc:935: `ntree_limit` is deprecated, use `iteration_range` instead.
## [15:50:14] WARNING: src/c_api/c_api.cc:935: `ntree_limit` is deprecated, use `iteration_range` instead.
## [15:50:14] WARNING: src/c_api/c_api.cc:935: `ntree_limit` is deprecated, use `iteration_range` instead.
## [15:50:14] WARNING: src/c_api/c_api.cc:935: `ntree_limit` is deprecated, use `iteration_range` instead.
## [15:50:15] WARNING: src/c_api/c_api.cc:935: `ntree_limit` is deprecated, use `iteration_range` instead.
## [15:50:15] WARNING: src/c_api/c_api.cc:935: `ntree_limit` is deprecated, use `iteration_range` instead.
## [15:50:15] WARNING: src/c_api/c_api.cc:935: `ntree_limit` is deprecated, use `iteration_range` instead.
## [15:50:15] WARNING: src/c_api/c_api.cc:935: `ntree_limit` is deprecated, use `iteration_range` instead.
## [15:50:15] WARNING: src/c_api/c_api.cc:935: `ntree_limit` is deprecated, use `iteration_range` instead.
## [15:50:15] WARNING: src/c_api/c_api.cc:935: `ntree_limit` is deprecated, use `iteration_range` instead.
## [15:50:15] WARNING: src/c_api/c_api.cc:935: `ntree_limit` is deprecated, use `iteration_range` instead.
## [15:50:15] WARNING: src/c_api/c_api.cc:935: `ntree_limit` is deprecated, use `iteration_range` instead.
## [15:50:16] WARNING: src/c_api/c_api.cc:935: `ntree_limit` is deprecated, use `iteration_range` instead.
## [15:50:16] WARNING: src/c_api/c_api.cc:935: `ntree_limit` is deprecated, use `iteration_range` instead.
## [15:50:18] WARNING: src/c_api/c_api.cc:935: `ntree_limit` is deprecated, use `iteration_range` instead.
## [15:50:18] WARNING: src/c_api/c_api.cc:935: `ntree_limit` is deprecated, use `iteration_range` instead.
## [15:50:19] WARNING: src/c_api/c_api.cc:935: `ntree_limit` is deprecated, use `iteration_range` instead.
## [15:50:19] WARNING: src/c_api/c_api.cc:935: `ntree_limit` is deprecated, use `iteration_range` instead.
## [15:50:19] WARNING: src/c_api/c_api.cc:935: `ntree_limit` is deprecated, use `iteration_range` instead.
## [15:50:19] WARNING: src/c_api/c_api.cc:935: `ntree_limit` is deprecated, use `iteration_range` instead.
## [15:50:19] WARNING: src/c_api/c_api.cc:935: `ntree_limit` is deprecated, use `iteration_range` instead.
## [15:50:19] WARNING: src/c_api/c_api.cc:935: `ntree_limit` is deprecated, use `iteration_range` instead.
## [15:50:19] WARNING: src/c_api/c_api.cc:935: `ntree_limit` is deprecated, use `iteration_range` instead.
## [15:50:19] WARNING: src/c_api/c_api.cc:935: `ntree_limit` is deprecated, use `iteration_range` instead.
## [15:50:19] WARNING: src/c_api/c_api.cc:935: `ntree_limit` is deprecated, use `iteration_range` instead.
## [15:50:19] WARNING: src/c_api/c_api.cc:935: `ntree_limit` is deprecated, use `iteration_range` instead.
## [15:50:19] WARNING: src/c_api/c_api.cc:935: `ntree_limit` is deprecated, use `iteration_range` instead.
## [15:50:19] WARNING: src/c_api/c_api.cc:935: `ntree_limit` is deprecated, use `iteration_range` instead.
## [15:50:20] WARNING: src/c_api/c_api.cc:935: `ntree_limit` is deprecated, use `iteration_range` instead.
## [15:50:20] WARNING: src/c_api/c_api.cc:935: `ntree_limit` is deprecated, use `iteration_range` instead.
## [15:50:20] WARNING: src/c_api/c_api.cc:935: `ntree_limit` is deprecated, use `iteration_range` instead.
## [15:50:20] WARNING: src/c_api/c_api.cc:935: `ntree_limit` is deprecated, use `iteration_range` instead.
## [15:50:20] WARNING: src/c_api/c_api.cc:935: `ntree_limit` is deprecated, use `iteration_range` instead.
## [15:50:20] WARNING: src/c_api/c_api.cc:935: `ntree_limit` is deprecated, use `iteration_range` instead.
## [15:50:20] WARNING: src/c_api/c_api.cc:935: `ntree_limit` is deprecated, use `iteration_range` instead.
## [15:50:20] WARNING: src/c_api/c_api.cc:935: `ntree_limit` is deprecated, use `iteration_range` instead.
## [15:50:20] WARNING: src/c_api/c_api.cc:935: `ntree_limit` is deprecated, use `iteration_range` instead.
## [15:50:20] WARNING: src/c_api/c_api.cc:935: `ntree_limit` is deprecated, use `iteration_range` instead.
## [15:50:20] WARNING: src/c_api/c_api.cc:935: `ntree_limit` is deprecated, use `iteration_range` instead.
## [15:50:20] WARNING: src/c_api/c_api.cc:935: `ntree_limit` is deprecated, use `iteration_range` instead.
## [15:50:21] WARNING: src/c_api/c_api.cc:935: `ntree_limit` is deprecated, use `iteration_range` instead.
## [15:50:21] WARNING: src/c_api/c_api.cc:935: `ntree_limit` is deprecated, use `iteration_range` instead.
## [15:50:21] WARNING: src/c_api/c_api.cc:935: `ntree_limit` is deprecated, use `iteration_range` instead.
## [15:50:21] WARNING: src/c_api/c_api.cc:935: `ntree_limit` is deprecated, use `iteration_range` instead.
## [15:50:21] WARNING: src/c_api/c_api.cc:935: `ntree_limit` is deprecated, use `iteration_range` instead.
## [15:50:21] WARNING: src/c_api/c_api.cc:935: `ntree_limit` is deprecated, use `iteration_range` instead.
## [15:50:21] WARNING: src/c_api/c_api.cc:935: `ntree_limit` is deprecated, use `iteration_range` instead.
## [15:50:21] WARNING: src/c_api/c_api.cc:935: `ntree_limit` is deprecated, use `iteration_range` instead.
## [15:50:22] WARNING: src/c_api/c_api.cc:935: `ntree_limit` is deprecated, use `iteration_range` instead.
## [15:50:22] WARNING: src/c_api/c_api.cc:935: `ntree_limit` is deprecated, use `iteration_range` instead.
## [15:50:22] WARNING: src/c_api/c_api.cc:935: `ntree_limit` is deprecated, use `iteration_range` instead.
## [15:50:22] WARNING: src/c_api/c_api.cc:935: `ntree_limit` is deprecated, use `iteration_range` instead.
## [15:50:22] WARNING: src/c_api/c_api.cc:935: `ntree_limit` is deprecated, use `iteration_range` instead.
## [15:50:22] WARNING: src/c_api/c_api.cc:935: `ntree_limit` is deprecated, use `iteration_range` instead.
## [15:50:22] WARNING: src/c_api/c_api.cc:935: `ntree_limit` is deprecated, use `iteration_range` instead.
## [15:50:22] WARNING: src/c_api/c_api.cc:935: `ntree_limit` is deprecated, use `iteration_range` instead.
## [15:50:22] WARNING: src/c_api/c_api.cc:935: `ntree_limit` is deprecated, use `iteration_range` instead.
## [15:50:22] WARNING: src/c_api/c_api.cc:935: `ntree_limit` is deprecated, use `iteration_range` instead.
## [15:50:22] WARNING: src/c_api/c_api.cc:935: `ntree_limit` is deprecated, use `iteration_range` instead.
## [15:50:22] WARNING: src/c_api/c_api.cc:935: `ntree_limit` is deprecated, use `iteration_range` instead.
## [15:50:22] WARNING: src/c_api/c_api.cc:935: `ntree_limit` is deprecated, use `iteration_range` instead.
## [15:50:22] WARNING: src/c_api/c_api.cc:935: `ntree_limit` is deprecated, use `iteration_range` instead.
## [15:50:23] WARNING: src/c_api/c_api.cc:935: `ntree_limit` is deprecated, use `iteration_range` instead.
## [15:50:23] WARNING: src/c_api/c_api.cc:935: `ntree_limit` is deprecated, use `iteration_range` instead.
## [15:50:23] WARNING: src/c_api/c_api.cc:935: `ntree_limit` is deprecated, use `iteration_range` instead.
## [15:50:23] WARNING: src/c_api/c_api.cc:935: `ntree_limit` is deprecated, use `iteration_range` instead.
## [15:50:23] WARNING: src/c_api/c_api.cc:935: `ntree_limit` is deprecated, use `iteration_range` instead.
## [15:50:23] WARNING: src/c_api/c_api.cc:935: `ntree_limit` is deprecated, use `iteration_range` instead.
## [15:50:23] WARNING: src/c_api/c_api.cc:935: `ntree_limit` is deprecated, use `iteration_range` instead.
## [15:50:23] WARNING: src/c_api/c_api.cc:935: `ntree_limit` is deprecated, use `iteration_range` instead.
## [15:50:23] WARNING: src/c_api/c_api.cc:935: `ntree_limit` is deprecated, use `iteration_range` instead.
## [15:50:23] WARNING: src/c_api/c_api.cc:935: `ntree_limit` is deprecated, use `iteration_range` instead.
## [15:50:23] WARNING: src/c_api/c_api.cc:935: `ntree_limit` is deprecated, use `iteration_range` instead.
## [15:50:23] WARNING: src/c_api/c_api.cc:935: `ntree_limit` is deprecated, use `iteration_range` instead.
## [15:50:24] WARNING: src/c_api/c_api.cc:935: `ntree_limit` is deprecated, use `iteration_range` instead.
## [15:50:24] WARNING: src/c_api/c_api.cc:935: `ntree_limit` is deprecated, use `iteration_range` instead.
## [15:50:24] WARNING: src/c_api/c_api.cc:935: `ntree_limit` is deprecated, use `iteration_range` instead.
## [15:50:24] WARNING: src/c_api/c_api.cc:935: `ntree_limit` is deprecated, use `iteration_range` instead.
## [15:50:24] WARNING: src/c_api/c_api.cc:935: `ntree_limit` is deprecated, use `iteration_range` instead.
## [15:50:24] WARNING: src/c_api/c_api.cc:935: `ntree_limit` is deprecated, use `iteration_range` instead.
## [15:50:24] WARNING: src/c_api/c_api.cc:935: `ntree_limit` is deprecated, use `iteration_range` instead.
## [15:50:24] WARNING: src/c_api/c_api.cc:935: `ntree_limit` is deprecated, use `iteration_range` instead.
## [15:50:24] WARNING: src/c_api/c_api.cc:935: `ntree_limit` is deprecated, use `iteration_range` instead.
## [15:50:24] WARNING: src/c_api/c_api.cc:935: `ntree_limit` is deprecated, use `iteration_range` instead.
## [15:50:25] WARNING: src/c_api/c_api.cc:935: `ntree_limit` is deprecated, use `iteration_range` instead.
## [15:50:25] WARNING: src/c_api/c_api.cc:935: `ntree_limit` is deprecated, use `iteration_range` instead.
## [15:50:25] WARNING: src/c_api/c_api.cc:935: `ntree_limit` is deprecated, use `iteration_range` instead.
## [15:50:25] WARNING: src/c_api/c_api.cc:935: `ntree_limit` is deprecated, use `iteration_range` instead.
## [15:50:25] WARNING: src/c_api/c_api.cc:935: `ntree_limit` is deprecated, use `iteration_range` instead.
## [15:50:25] WARNING: src/c_api/c_api.cc:935: `ntree_limit` is deprecated, use `iteration_range` instead.
## [15:50:25] WARNING: src/c_api/c_api.cc:935: `ntree_limit` is deprecated, use `iteration_range` instead.
## [15:50:25] WARNING: src/c_api/c_api.cc:935: `ntree_limit` is deprecated, use `iteration_range` instead.
## [15:50:25] WARNING: src/c_api/c_api.cc:935: `ntree_limit` is deprecated, use `iteration_range` instead.
## [15:50:25] WARNING: src/c_api/c_api.cc:935: `ntree_limit` is deprecated, use `iteration_range` instead.
## [15:50:25] WARNING: src/c_api/c_api.cc:935: `ntree_limit` is deprecated, use `iteration_range` instead.
## [15:50:25] WARNING: src/c_api/c_api.cc:935: `ntree_limit` is deprecated, use `iteration_range` instead.
## [15:50:25] WARNING: src/c_api/c_api.cc:935: `ntree_limit` is deprecated, use `iteration_range` instead.
## [15:50:25] WARNING: src/c_api/c_api.cc:935: `ntree_limit` is deprecated, use `iteration_range` instead.
## [15:50:26] WARNING: src/c_api/c_api.cc:935: `ntree_limit` is deprecated, use `iteration_range` instead.
## [15:50:26] WARNING: src/c_api/c_api.cc:935: `ntree_limit` is deprecated, use `iteration_range` instead.
## [15:50:26] WARNING: src/c_api/c_api.cc:935: `ntree_limit` is deprecated, use `iteration_range` instead.
## [15:50:26] WARNING: src/c_api/c_api.cc:935: `ntree_limit` is deprecated, use `iteration_range` instead.
## [15:50:26] WARNING: src/c_api/c_api.cc:935: `ntree_limit` is deprecated, use `iteration_range` instead.
## [15:50:26] WARNING: src/c_api/c_api.cc:935: `ntree_limit` is deprecated, use `iteration_range` instead.
## [15:50:26] WARNING: src/c_api/c_api.cc:935: `ntree_limit` is deprecated, use `iteration_range` instead.
## [15:50:26] WARNING: src/c_api/c_api.cc:935: `ntree_limit` is deprecated, use `iteration_range` instead.
## [15:50:26] WARNING: src/c_api/c_api.cc:935: `ntree_limit` is deprecated, use `iteration_range` instead.
## [15:50:26] WARNING: src/c_api/c_api.cc:935: `ntree_limit` is deprecated, use `iteration_range` instead.
## [15:50:27] WARNING: src/c_api/c_api.cc:935: `ntree_limit` is deprecated, use `iteration_range` instead.
## [15:50:27] WARNING: src/c_api/c_api.cc:935: `ntree_limit` is deprecated, use `iteration_range` instead.
## [15:50:27] WARNING: src/c_api/c_api.cc:935: `ntree_limit` is deprecated, use `iteration_range` instead.
## [15:50:27] WARNING: src/c_api/c_api.cc:935: `ntree_limit` is deprecated, use `iteration_range` instead.
## [15:50:27] WARNING: src/c_api/c_api.cc:935: `ntree_limit` is deprecated, use `iteration_range` instead.
## [15:50:27] WARNING: src/c_api/c_api.cc:935: `ntree_limit` is deprecated, use `iteration_range` instead.
## [15:50:27] WARNING: src/c_api/c_api.cc:935: `ntree_limit` is deprecated, use `iteration_range` instead.
## [15:50:27] WARNING: src/c_api/c_api.cc:935: `ntree_limit` is deprecated, use `iteration_range` instead.
## [15:50:28] WARNING: src/c_api/c_api.cc:935: `ntree_limit` is deprecated, use `iteration_range` instead.
## [15:50:28] WARNING: src/c_api/c_api.cc:935: `ntree_limit` is deprecated, use `iteration_range` instead.
## [15:50:28] WARNING: src/c_api/c_api.cc:935: `ntree_limit` is deprecated, use `iteration_range` instead.
## [15:50:28] WARNING: src/c_api/c_api.cc:935: `ntree_limit` is deprecated, use `iteration_range` instead.
## [15:50:28] WARNING: src/c_api/c_api.cc:935: `ntree_limit` is deprecated, use `iteration_range` instead.
## [15:50:28] WARNING: src/c_api/c_api.cc:935: `ntree_limit` is deprecated, use `iteration_range` instead.
## [15:50:28] WARNING: src/c_api/c_api.cc:935: `ntree_limit` is deprecated, use `iteration_range` instead.
## [15:50:28] WARNING: src/c_api/c_api.cc:935: `ntree_limit` is deprecated, use `iteration_range` instead.
## [15:50:28] WARNING: src/c_api/c_api.cc:935: `ntree_limit` is deprecated, use `iteration_range` instead.
## [15:50:28] WARNING: src/c_api/c_api.cc:935: `ntree_limit` is deprecated, use `iteration_range` instead.
## [15:50:28] WARNING: src/c_api/c_api.cc:935: `ntree_limit` is deprecated, use `iteration_range` instead.
## [15:50:28] WARNING: src/c_api/c_api.cc:935: `ntree_limit` is deprecated, use `iteration_range` instead.
## [15:50:28] WARNING: src/c_api/c_api.cc:935: `ntree_limit` is deprecated, use `iteration_range` instead.
## [15:50:28] WARNING: src/c_api/c_api.cc:935: `ntree_limit` is deprecated, use `iteration_range` instead.
## [15:50:29] WARNING: src/c_api/c_api.cc:935: `ntree_limit` is deprecated, use `iteration_range` instead.
## [15:50:29] WARNING: src/c_api/c_api.cc:935: `ntree_limit` is deprecated, use `iteration_range` instead.
## [15:50:29] WARNING: src/c_api/c_api.cc:935: `ntree_limit` is deprecated, use `iteration_range` instead.
## [15:50:29] WARNING: src/c_api/c_api.cc:935: `ntree_limit` is deprecated, use `iteration_range` instead.
## [15:50:29] WARNING: src/c_api/c_api.cc:935: `ntree_limit` is deprecated, use `iteration_range` instead.
## [15:50:29] WARNING: src/c_api/c_api.cc:935: `ntree_limit` is deprecated, use `iteration_range` instead.
## [15:50:29] WARNING: src/c_api/c_api.cc:935: `ntree_limit` is deprecated, use `iteration_range` instead.
## [15:50:29] WARNING: src/c_api/c_api.cc:935: `ntree_limit` is deprecated, use `iteration_range` instead.
## [15:50:29] WARNING: src/c_api/c_api.cc:935: `ntree_limit` is deprecated, use `iteration_range` instead.
## [15:50:29] WARNING: src/c_api/c_api.cc:935: `ntree_limit` is deprecated, use `iteration_range` instead.
## [15:50:29] WARNING: src/c_api/c_api.cc:935: `ntree_limit` is deprecated, use `iteration_range` instead.
## [15:50:29] WARNING: src/c_api/c_api.cc:935: `ntree_limit` is deprecated, use `iteration_range` instead.
## [15:50:30] WARNING: src/c_api/c_api.cc:935: `ntree_limit` is deprecated, use `iteration_range` instead.
## [15:50:30] WARNING: src/c_api/c_api.cc:935: `ntree_limit` is deprecated, use `iteration_range` instead.
## [15:50:30] WARNING: src/c_api/c_api.cc:935: `ntree_limit` is deprecated, use `iteration_range` instead.
## [15:50:30] WARNING: src/c_api/c_api.cc:935: `ntree_limit` is deprecated, use `iteration_range` instead.
## [15:50:30] WARNING: src/c_api/c_api.cc:935: `ntree_limit` is deprecated, use `iteration_range` instead.
## [15:50:30] WARNING: src/c_api/c_api.cc:935: `ntree_limit` is deprecated, use `iteration_range` instead.
## [15:50:30] WARNING: src/c_api/c_api.cc:935: `ntree_limit` is deprecated, use `iteration_range` instead.
## [15:50:30] WARNING: src/c_api/c_api.cc:935: `ntree_limit` is deprecated, use `iteration_range` instead.
## [15:50:30] WARNING: src/c_api/c_api.cc:935: `ntree_limit` is deprecated, use `iteration_range` instead.
## [15:50:30] WARNING: src/c_api/c_api.cc:935: `ntree_limit` is deprecated, use `iteration_range` instead.
## [15:50:31] WARNING: src/c_api/c_api.cc:935: `ntree_limit` is deprecated, use `iteration_range` instead.
## [15:50:31] WARNING: src/c_api/c_api.cc:935: `ntree_limit` is deprecated, use `iteration_range` instead.
## [15:50:31] WARNING: src/c_api/c_api.cc:935: `ntree_limit` is deprecated, use `iteration_range` instead.
## [15:50:31] WARNING: src/c_api/c_api.cc:935: `ntree_limit` is deprecated, use `iteration_range` instead.
## [15:50:31] WARNING: src/c_api/c_api.cc:935: `ntree_limit` is deprecated, use `iteration_range` instead.
## [15:50:31] WARNING: src/c_api/c_api.cc:935: `ntree_limit` is deprecated, use `iteration_range` instead.
## [15:50:31] WARNING: src/c_api/c_api.cc:935: `ntree_limit` is deprecated, use `iteration_range` instead.
## [15:50:31] WARNING: src/c_api/c_api.cc:935: `ntree_limit` is deprecated, use `iteration_range` instead.
## [15:50:31] WARNING: src/c_api/c_api.cc:935: `ntree_limit` is deprecated, use `iteration_range` instead.
## [15:50:31] WARNING: src/c_api/c_api.cc:935: `ntree_limit` is deprecated, use `iteration_range` instead.
## [15:50:31] WARNING: src/c_api/c_api.cc:935: `ntree_limit` is deprecated, use `iteration_range` instead.
## [15:50:31] WARNING: src/c_api/c_api.cc:935: `ntree_limit` is deprecated, use `iteration_range` instead.
## [15:50:32] WARNING: src/c_api/c_api.cc:935: `ntree_limit` is deprecated, use `iteration_range` instead.
## [15:50:32] WARNING: src/c_api/c_api.cc:935: `ntree_limit` is deprecated, use `iteration_range` instead.
## [15:50:32] WARNING: src/c_api/c_api.cc:935: `ntree_limit` is deprecated, use `iteration_range` instead.
## [15:50:32] WARNING: src/c_api/c_api.cc:935: `ntree_limit` is deprecated, use `iteration_range` instead.
## [15:50:32] WARNING: src/c_api/c_api.cc:935: `ntree_limit` is deprecated, use `iteration_range` instead.
## [15:50:32] WARNING: src/c_api/c_api.cc:935: `ntree_limit` is deprecated, use `iteration_range` instead.
## [15:50:32] WARNING: src/c_api/c_api.cc:935: `ntree_limit` is deprecated, use `iteration_range` instead.
## [15:50:32] WARNING: src/c_api/c_api.cc:935: `ntree_limit` is deprecated, use `iteration_range` instead.
## [15:50:32] WARNING: src/c_api/c_api.cc:935: `ntree_limit` is deprecated, use `iteration_range` instead.
## [15:50:32] WARNING: src/c_api/c_api.cc:935: `ntree_limit` is deprecated, use `iteration_range` instead.
## [15:50:32] WARNING: src/c_api/c_api.cc:935: `ntree_limit` is deprecated, use `iteration_range` instead.
## [15:50:32] WARNING: src/c_api/c_api.cc:935: `ntree_limit` is deprecated, use `iteration_range` instead.
## [15:50:33] WARNING: src/c_api/c_api.cc:935: `ntree_limit` is deprecated, use `iteration_range` instead.
## [15:50:33] WARNING: src/c_api/c_api.cc:935: `ntree_limit` is deprecated, use `iteration_range` instead.
## [15:50:33] WARNING: src/c_api/c_api.cc:935: `ntree_limit` is deprecated, use `iteration_range` instead.
## [15:50:33] WARNING: src/c_api/c_api.cc:935: `ntree_limit` is deprecated, use `iteration_range` instead.
## [15:50:33] WARNING: src/c_api/c_api.cc:935: `ntree_limit` is deprecated, use `iteration_range` instead.
## [15:50:33] WARNING: src/c_api/c_api.cc:935: `ntree_limit` is deprecated, use `iteration_range` instead.
## [15:50:33] WARNING: src/c_api/c_api.cc:935: `ntree_limit` is deprecated, use `iteration_range` instead.
## [15:50:33] WARNING: src/c_api/c_api.cc:935: `ntree_limit` is deprecated, use `iteration_range` instead.
## [15:50:33] WARNING: src/c_api/c_api.cc:935: `ntree_limit` is deprecated, use `iteration_range` instead.
## [15:50:33] WARNING: src/c_api/c_api.cc:935: `ntree_limit` is deprecated, use `iteration_range` instead.
## [15:50:34] WARNING: src/c_api/c_api.cc:935: `ntree_limit` is deprecated, use `iteration_range` instead.
## [15:50:34] WARNING: src/c_api/c_api.cc:935: `ntree_limit` is deprecated, use `iteration_range` instead.
## [15:50:34] WARNING: src/c_api/c_api.cc:935: `ntree_limit` is deprecated, use `iteration_range` instead.
## [15:50:34] WARNING: src/c_api/c_api.cc:935: `ntree_limit` is deprecated, use `iteration_range` instead.
## [15:50:34] WARNING: src/c_api/c_api.cc:935: `ntree_limit` is deprecated, use `iteration_range` instead.
## [15:50:34] WARNING: src/c_api/c_api.cc:935: `ntree_limit` is deprecated, use `iteration_range` instead.
## [15:50:34] WARNING: src/c_api/c_api.cc:935: `ntree_limit` is deprecated, use `iteration_range` instead.
## [15:50:34] WARNING: src/c_api/c_api.cc:935: `ntree_limit` is deprecated, use `iteration_range` instead.
## [15:50:34] WARNING: src/c_api/c_api.cc:935: `ntree_limit` is deprecated, use `iteration_range` instead.
## [15:50:34] WARNING: src/c_api/c_api.cc:935: `ntree_limit` is deprecated, use `iteration_range` instead.
## [15:50:34] WARNING: src/c_api/c_api.cc:935: `ntree_limit` is deprecated, use `iteration_range` instead.
## [15:50:34] WARNING: src/c_api/c_api.cc:935: `ntree_limit` is deprecated, use `iteration_range` instead.
## [15:50:35] WARNING: src/c_api/c_api.cc:935: `ntree_limit` is deprecated, use `iteration_range` instead.
## [15:50:35] WARNING: src/c_api/c_api.cc:935: `ntree_limit` is deprecated, use `iteration_range` instead.
## [15:50:35] WARNING: src/c_api/c_api.cc:935: `ntree_limit` is deprecated, use `iteration_range` instead.
## [15:50:35] WARNING: src/c_api/c_api.cc:935: `ntree_limit` is deprecated, use `iteration_range` instead.
## [15:50:35] WARNING: src/c_api/c_api.cc:935: `ntree_limit` is deprecated, use `iteration_range` instead.
## [15:50:35] WARNING: src/c_api/c_api.cc:935: `ntree_limit` is deprecated, use `iteration_range` instead.
## [15:50:35] WARNING: src/c_api/c_api.cc:935: `ntree_limit` is deprecated, use `iteration_range` instead.
## [15:50:35] WARNING: src/c_api/c_api.cc:935: `ntree_limit` is deprecated, use `iteration_range` instead.
## [15:50:35] WARNING: src/c_api/c_api.cc:935: `ntree_limit` is deprecated, use `iteration_range` instead.
## [15:50:35] WARNING: src/c_api/c_api.cc:935: `ntree_limit` is deprecated, use `iteration_range` instead.
## [15:50:35] WARNING: src/c_api/c_api.cc:935: `ntree_limit` is deprecated, use `iteration_range` instead.
## [15:50:35] WARNING: src/c_api/c_api.cc:935: `ntree_limit` is deprecated, use `iteration_range` instead.
## [15:50:36] WARNING: src/c_api/c_api.cc:935: `ntree_limit` is deprecated, use `iteration_range` instead.
## [15:50:36] WARNING: src/c_api/c_api.cc:935: `ntree_limit` is deprecated, use `iteration_range` instead.
## [15:50:36] WARNING: src/c_api/c_api.cc:935: `ntree_limit` is deprecated, use `iteration_range` instead.
## [15:50:36] WARNING: src/c_api/c_api.cc:935: `ntree_limit` is deprecated, use `iteration_range` instead.
## [15:50:36] WARNING: src/c_api/c_api.cc:935: `ntree_limit` is deprecated, use `iteration_range` instead.
## [15:50:36] WARNING: src/c_api/c_api.cc:935: `ntree_limit` is deprecated, use `iteration_range` instead.
## [15:50:36] WARNING: src/c_api/c_api.cc:935: `ntree_limit` is deprecated, use `iteration_range` instead.
## [15:50:36] WARNING: src/c_api/c_api.cc:935: `ntree_limit` is deprecated, use `iteration_range` instead.
## [15:50:36] WARNING: src/c_api/c_api.cc:935: `ntree_limit` is deprecated, use `iteration_range` instead.
## [15:50:36] WARNING: src/c_api/c_api.cc:935: `ntree_limit` is deprecated, use `iteration_range` instead.
## [15:50:36] WARNING: src/c_api/c_api.cc:935: `ntree_limit` is deprecated, use `iteration_range` instead.
## [15:50:36] WARNING: src/c_api/c_api.cc:935: `ntree_limit` is deprecated, use `iteration_range` instead.
## [15:50:37] WARNING: src/c_api/c_api.cc:935: `ntree_limit` is deprecated, use `iteration_range` instead.
## [15:50:37] WARNING: src/c_api/c_api.cc:935: `ntree_limit` is deprecated, use `iteration_range` instead.
## [15:50:37] WARNING: src/c_api/c_api.cc:935: `ntree_limit` is deprecated, use `iteration_range` instead.
## [15:50:37] WARNING: src/c_api/c_api.cc:935: `ntree_limit` is deprecated, use `iteration_range` instead.
## [15:50:37] WARNING: src/c_api/c_api.cc:935: `ntree_limit` is deprecated, use `iteration_range` instead.
## [15:50:37] WARNING: src/c_api/c_api.cc:935: `ntree_limit` is deprecated, use `iteration_range` instead.
## [15:50:37] WARNING: src/c_api/c_api.cc:935: `ntree_limit` is deprecated, use `iteration_range` instead.
## [15:50:37] WARNING: src/c_api/c_api.cc:935: `ntree_limit` is deprecated, use `iteration_range` instead.
## [15:50:37] WARNING: src/c_api/c_api.cc:935: `ntree_limit` is deprecated, use `iteration_range` instead.
## [15:50:37] WARNING: src/c_api/c_api.cc:935: `ntree_limit` is deprecated, use `iteration_range` instead.
## [15:50:38] WARNING: src/c_api/c_api.cc:935: `ntree_limit` is deprecated, use `iteration_range` instead.
## [15:50:38] WARNING: src/c_api/c_api.cc:935: `ntree_limit` is deprecated, use `iteration_range` instead.
## [15:50:38] WARNING: src/c_api/c_api.cc:935: `ntree_limit` is deprecated, use `iteration_range` instead.
## [15:50:38] WARNING: src/c_api/c_api.cc:935: `ntree_limit` is deprecated, use `iteration_range` instead.
## [15:50:38] WARNING: src/c_api/c_api.cc:935: `ntree_limit` is deprecated, use `iteration_range` instead.
## [15:50:38] WARNING: src/c_api/c_api.cc:935: `ntree_limit` is deprecated, use `iteration_range` instead.
## [15:50:38] WARNING: src/c_api/c_api.cc:935: `ntree_limit` is deprecated, use `iteration_range` instead.
## [15:50:38] WARNING: src/c_api/c_api.cc:935: `ntree_limit` is deprecated, use `iteration_range` instead.
## [15:50:38] WARNING: src/c_api/c_api.cc:935: `ntree_limit` is deprecated, use `iteration_range` instead.
## [15:50:38] WARNING: src/c_api/c_api.cc:935: `ntree_limit` is deprecated, use `iteration_range` instead.
## [15:50:38] WARNING: src/c_api/c_api.cc:935: `ntree_limit` is deprecated, use `iteration_range` instead.
## [15:50:38] WARNING: src/c_api/c_api.cc:935: `ntree_limit` is deprecated, use `iteration_range` instead.
## [15:50:38] WARNING: src/c_api/c_api.cc:935: `ntree_limit` is deprecated, use `iteration_range` instead.
## [15:50:38] WARNING: src/c_api/c_api.cc:935: `ntree_limit` is deprecated, use `iteration_range` instead.
## [15:50:39] WARNING: src/c_api/c_api.cc:935: `ntree_limit` is deprecated, use `iteration_range` instead.
## [15:50:39] WARNING: src/c_api/c_api.cc:935: `ntree_limit` is deprecated, use `iteration_range` instead.
## [15:50:39] WARNING: src/c_api/c_api.cc:935: `ntree_limit` is deprecated, use `iteration_range` instead.
## [15:50:39] WARNING: src/c_api/c_api.cc:935: `ntree_limit` is deprecated, use `iteration_range` instead.
## [15:50:39] WARNING: src/c_api/c_api.cc:935: `ntree_limit` is deprecated, use `iteration_range` instead.
## [15:50:39] WARNING: src/c_api/c_api.cc:935: `ntree_limit` is deprecated, use `iteration_range` instead.
## [15:50:39] WARNING: src/c_api/c_api.cc:935: `ntree_limit` is deprecated, use `iteration_range` instead.
## [15:50:39] WARNING: src/c_api/c_api.cc:935: `ntree_limit` is deprecated, use `iteration_range` instead.
## [15:50:39] WARNING: src/c_api/c_api.cc:935: `ntree_limit` is deprecated, use `iteration_range` instead.
## [15:50:39] WARNING: src/c_api/c_api.cc:935: `ntree_limit` is deprecated, use `iteration_range` instead.
## [15:50:40] WARNING: src/c_api/c_api.cc:935: `ntree_limit` is deprecated, use `iteration_range` instead.
## [15:50:40] WARNING: src/c_api/c_api.cc:935: `ntree_limit` is deprecated, use `iteration_range` instead.
## [15:50:40] WARNING: src/c_api/c_api.cc:935: `ntree_limit` is deprecated, use `iteration_range` instead.
## [15:50:40] WARNING: src/c_api/c_api.cc:935: `ntree_limit` is deprecated, use `iteration_range` instead.
## [15:50:40] WARNING: src/c_api/c_api.cc:935: `ntree_limit` is deprecated, use `iteration_range` instead.
## [15:50:40] WARNING: src/c_api/c_api.cc:935: `ntree_limit` is deprecated, use `iteration_range` instead.
## [15:50:40] WARNING: src/c_api/c_api.cc:935: `ntree_limit` is deprecated, use `iteration_range` instead.
## [15:50:40] WARNING: src/c_api/c_api.cc:935: `ntree_limit` is deprecated, use `iteration_range` instead.
## [15:50:41] WARNING: src/c_api/c_api.cc:935: `ntree_limit` is deprecated, use `iteration_range` instead.
## [15:50:41] WARNING: src/c_api/c_api.cc:935: `ntree_limit` is deprecated, use `iteration_range` instead.
## [15:50:41] WARNING: src/c_api/c_api.cc:935: `ntree_limit` is deprecated, use `iteration_range` instead.
## [15:50:41] WARNING: src/c_api/c_api.cc:935: `ntree_limit` is deprecated, use `iteration_range` instead.
## [15:50:41] WARNING: src/c_api/c_api.cc:935: `ntree_limit` is deprecated, use `iteration_range` instead.
## [15:50:41] WARNING: src/c_api/c_api.cc:935: `ntree_limit` is deprecated, use `iteration_range` instead.
## [15:50:41] WARNING: src/c_api/c_api.cc:935: `ntree_limit` is deprecated, use `iteration_range` instead.
## [15:50:41] WARNING: src/c_api/c_api.cc:935: `ntree_limit` is deprecated, use `iteration_range` instead.
## [15:50:41] WARNING: src/c_api/c_api.cc:935: `ntree_limit` is deprecated, use `iteration_range` instead.
## [15:50:41] WARNING: src/c_api/c_api.cc:935: `ntree_limit` is deprecated, use `iteration_range` instead.
## [15:50:41] WARNING: src/c_api/c_api.cc:935: `ntree_limit` is deprecated, use `iteration_range` instead.
## [15:50:41] WARNING: src/c_api/c_api.cc:935: `ntree_limit` is deprecated, use `iteration_range` instead.
## [15:50:41] WARNING: src/c_api/c_api.cc:935: `ntree_limit` is deprecated, use `iteration_range` instead.
## [15:50:41] WARNING: src/c_api/c_api.cc:935: `ntree_limit` is deprecated, use `iteration_range` instead.
## [15:50:42] WARNING: src/c_api/c_api.cc:935: `ntree_limit` is deprecated, use `iteration_range` instead.
## [15:50:42] WARNING: src/c_api/c_api.cc:935: `ntree_limit` is deprecated, use `iteration_range` instead.
## [15:50:42] WARNING: src/c_api/c_api.cc:935: `ntree_limit` is deprecated, use `iteration_range` instead.
## [15:50:42] WARNING: src/c_api/c_api.cc:935: `ntree_limit` is deprecated, use `iteration_range` instead.
## [15:50:42] WARNING: src/c_api/c_api.cc:935: `ntree_limit` is deprecated, use `iteration_range` instead.
## [15:50:42] WARNING: src/c_api/c_api.cc:935: `ntree_limit` is deprecated, use `iteration_range` instead.
## [15:50:42] WARNING: src/c_api/c_api.cc:935: `ntree_limit` is deprecated, use `iteration_range` instead.
## [15:50:42] WARNING: src/c_api/c_api.cc:935: `ntree_limit` is deprecated, use `iteration_range` instead.
## [15:50:42] WARNING: src/c_api/c_api.cc:935: `ntree_limit` is deprecated, use `iteration_range` instead.
## [15:50:42] WARNING: src/c_api/c_api.cc:935: `ntree_limit` is deprecated, use `iteration_range` instead.
## [15:50:42] WARNING: src/c_api/c_api.cc:935: `ntree_limit` is deprecated, use `iteration_range` instead.
## [15:50:42] WARNING: src/c_api/c_api.cc:935: `ntree_limit` is deprecated, use `iteration_range` instead.
## [15:50:43] WARNING: src/c_api/c_api.cc:935: `ntree_limit` is deprecated, use `iteration_range` instead.
## [15:50:43] WARNING: src/c_api/c_api.cc:935: `ntree_limit` is deprecated, use `iteration_range` instead.
## [15:50:43] WARNING: src/c_api/c_api.cc:935: `ntree_limit` is deprecated, use `iteration_range` instead.
## [15:50:43] WARNING: src/c_api/c_api.cc:935: `ntree_limit` is deprecated, use `iteration_range` instead.
## [15:50:43] WARNING: src/c_api/c_api.cc:935: `ntree_limit` is deprecated, use `iteration_range` instead.
## [15:50:43] WARNING: src/c_api/c_api.cc:935: `ntree_limit` is deprecated, use `iteration_range` instead.
## [15:50:43] WARNING: src/c_api/c_api.cc:935: `ntree_limit` is deprecated, use `iteration_range` instead.
## [15:50:43] WARNING: src/c_api/c_api.cc:935: `ntree_limit` is deprecated, use `iteration_range` instead.
## [15:50:43] WARNING: src/c_api/c_api.cc:935: `ntree_limit` is deprecated, use `iteration_range` instead.
## [15:50:43] WARNING: src/c_api/c_api.cc:935: `ntree_limit` is deprecated, use `iteration_range` instead.
## [15:50:44] WARNING: src/c_api/c_api.cc:935: `ntree_limit` is deprecated, use `iteration_range` instead.
## [15:50:44] WARNING: src/c_api/c_api.cc:935: `ntree_limit` is deprecated, use `iteration_range` instead.
## [15:50:46] WARNING: src/c_api/c_api.cc:935: `ntree_limit` is deprecated, use `iteration_range` instead.
## [15:50:46] WARNING: src/c_api/c_api.cc:935: `ntree_limit` is deprecated, use `iteration_range` instead.
## [15:50:47] WARNING: src/c_api/c_api.cc:935: `ntree_limit` is deprecated, use `iteration_range` instead.
## [15:50:47] WARNING: src/c_api/c_api.cc:935: `ntree_limit` is deprecated, use `iteration_range` instead.
## [15:50:47] WARNING: src/c_api/c_api.cc:935: `ntree_limit` is deprecated, use `iteration_range` instead.
## [15:50:47] WARNING: src/c_api/c_api.cc:935: `ntree_limit` is deprecated, use `iteration_range` instead.
## [15:50:47] WARNING: src/c_api/c_api.cc:935: `ntree_limit` is deprecated, use `iteration_range` instead.
## [15:50:47] WARNING: src/c_api/c_api.cc:935: `ntree_limit` is deprecated, use `iteration_range` instead.
## [15:50:47] WARNING: src/c_api/c_api.cc:935: `ntree_limit` is deprecated, use `iteration_range` instead.
## [15:50:47] WARNING: src/c_api/c_api.cc:935: `ntree_limit` is deprecated, use `iteration_range` instead.
## [15:50:47] WARNING: src/c_api/c_api.cc:935: `ntree_limit` is deprecated, use `iteration_range` instead.
## [15:50:47] WARNING: src/c_api/c_api.cc:935: `ntree_limit` is deprecated, use `iteration_range` instead.
## [15:50:47] WARNING: src/c_api/c_api.cc:935: `ntree_limit` is deprecated, use `iteration_range` instead.
## [15:50:47] WARNING: src/c_api/c_api.cc:935: `ntree_limit` is deprecated, use `iteration_range` instead.
## [15:50:48] WARNING: src/c_api/c_api.cc:935: `ntree_limit` is deprecated, use `iteration_range` instead.
## [15:50:48] WARNING: src/c_api/c_api.cc:935: `ntree_limit` is deprecated, use `iteration_range` instead.
## [15:50:48] WARNING: src/c_api/c_api.cc:935: `ntree_limit` is deprecated, use `iteration_range` instead.
## [15:50:48] WARNING: src/c_api/c_api.cc:935: `ntree_limit` is deprecated, use `iteration_range` instead.
## [15:50:48] WARNING: src/c_api/c_api.cc:935: `ntree_limit` is deprecated, use `iteration_range` instead.
## [15:50:48] WARNING: src/c_api/c_api.cc:935: `ntree_limit` is deprecated, use `iteration_range` instead.
## [15:50:48] WARNING: src/c_api/c_api.cc:935: `ntree_limit` is deprecated, use `iteration_range` instead.
## [15:50:48] WARNING: src/c_api/c_api.cc:935: `ntree_limit` is deprecated, use `iteration_range` instead.
## [15:50:48] WARNING: src/c_api/c_api.cc:935: `ntree_limit` is deprecated, use `iteration_range` instead.
## [15:50:48] WARNING: src/c_api/c_api.cc:935: `ntree_limit` is deprecated, use `iteration_range` instead.
## [15:50:48] WARNING: src/c_api/c_api.cc:935: `ntree_limit` is deprecated, use `iteration_range` instead.
## [15:50:48] WARNING: src/c_api/c_api.cc:935: `ntree_limit` is deprecated, use `iteration_range` instead.
## [15:50:49] WARNING: src/c_api/c_api.cc:935: `ntree_limit` is deprecated, use `iteration_range` instead.
## [15:50:49] WARNING: src/c_api/c_api.cc:935: `ntree_limit` is deprecated, use `iteration_range` instead.
## [15:50:49] WARNING: src/c_api/c_api.cc:935: `ntree_limit` is deprecated, use `iteration_range` instead.
## [15:50:49] WARNING: src/c_api/c_api.cc:935: `ntree_limit` is deprecated, use `iteration_range` instead.
## [15:50:49] WARNING: src/c_api/c_api.cc:935: `ntree_limit` is deprecated, use `iteration_range` instead.
## [15:50:49] WARNING: src/c_api/c_api.cc:935: `ntree_limit` is deprecated, use `iteration_range` instead.
## [15:50:49] WARNING: src/c_api/c_api.cc:935: `ntree_limit` is deprecated, use `iteration_range` instead.
## [15:50:49] WARNING: src/c_api/c_api.cc:935: `ntree_limit` is deprecated, use `iteration_range` instead.
## [15:50:50] WARNING: src/c_api/c_api.cc:935: `ntree_limit` is deprecated, use `iteration_range` instead.
## [15:50:50] WARNING: src/c_api/c_api.cc:935: `ntree_limit` is deprecated, use `iteration_range` instead.
## [15:50:50] WARNING: src/c_api/c_api.cc:935: `ntree_limit` is deprecated, use `iteration_range` instead.
## [15:50:50] WARNING: src/c_api/c_api.cc:935: `ntree_limit` is deprecated, use `iteration_range` instead.
## [15:50:50] WARNING: src/c_api/c_api.cc:935: `ntree_limit` is deprecated, use `iteration_range` instead.
## [15:50:50] WARNING: src/c_api/c_api.cc:935: `ntree_limit` is deprecated, use `iteration_range` instead.
## [15:50:50] WARNING: src/c_api/c_api.cc:935: `ntree_limit` is deprecated, use `iteration_range` instead.
## [15:50:50] WARNING: src/c_api/c_api.cc:935: `ntree_limit` is deprecated, use `iteration_range` instead.
## [15:50:50] WARNING: src/c_api/c_api.cc:935: `ntree_limit` is deprecated, use `iteration_range` instead.
## [15:50:50] WARNING: src/c_api/c_api.cc:935: `ntree_limit` is deprecated, use `iteration_range` instead.
## [15:50:50] WARNING: src/c_api/c_api.cc:935: `ntree_limit` is deprecated, use `iteration_range` instead.
## [15:50:50] WARNING: src/c_api/c_api.cc:935: `ntree_limit` is deprecated, use `iteration_range` instead.
## [15:50:50] WARNING: src/c_api/c_api.cc:935: `ntree_limit` is deprecated, use `iteration_range` instead.
## [15:50:50] WARNING: src/c_api/c_api.cc:935: `ntree_limit` is deprecated, use `iteration_range` instead.
## [15:50:50] WARNING: src/c_api/c_api.cc:935: `ntree_limit` is deprecated, use `iteration_range` instead.
## [15:50:50] WARNING: src/c_api/c_api.cc:935: `ntree_limit` is deprecated, use `iteration_range` instead.
## [15:50:51] WARNING: src/c_api/c_api.cc:935: `ntree_limit` is deprecated, use `iteration_range` instead.
## [15:50:51] WARNING: src/c_api/c_api.cc:935: `ntree_limit` is deprecated, use `iteration_range` instead.
## [15:50:51] WARNING: src/c_api/c_api.cc:935: `ntree_limit` is deprecated, use `iteration_range` instead.
## [15:50:51] WARNING: src/c_api/c_api.cc:935: `ntree_limit` is deprecated, use `iteration_range` instead.
## [15:50:51] WARNING: src/c_api/c_api.cc:935: `ntree_limit` is deprecated, use `iteration_range` instead.
## [15:50:51] WARNING: src/c_api/c_api.cc:935: `ntree_limit` is deprecated, use `iteration_range` instead.
## [15:50:51] WARNING: src/c_api/c_api.cc:935: `ntree_limit` is deprecated, use `iteration_range` instead.
## [15:50:51] WARNING: src/c_api/c_api.cc:935: `ntree_limit` is deprecated, use `iteration_range` instead.
## [15:50:51] WARNING: src/c_api/c_api.cc:935: `ntree_limit` is deprecated, use `iteration_range` instead.
## [15:50:51] WARNING: src/c_api/c_api.cc:935: `ntree_limit` is deprecated, use `iteration_range` instead.
## [15:50:52] WARNING: src/c_api/c_api.cc:935: `ntree_limit` is deprecated, use `iteration_range` instead.
## [15:50:52] WARNING: src/c_api/c_api.cc:935: `ntree_limit` is deprecated, use `iteration_range` instead.
## [15:50:52] WARNING: src/c_api/c_api.cc:935: `ntree_limit` is deprecated, use `iteration_range` instead.
## [15:50:52] WARNING: src/c_api/c_api.cc:935: `ntree_limit` is deprecated, use `iteration_range` instead.
## [15:50:52] WARNING: src/c_api/c_api.cc:935: `ntree_limit` is deprecated, use `iteration_range` instead.
## [15:50:52] WARNING: src/c_api/c_api.cc:935: `ntree_limit` is deprecated, use `iteration_range` instead.
## [15:50:52] WARNING: src/c_api/c_api.cc:935: `ntree_limit` is deprecated, use `iteration_range` instead.
## [15:50:52] WARNING: src/c_api/c_api.cc:935: `ntree_limit` is deprecated, use `iteration_range` instead.
## [15:50:52] WARNING: src/c_api/c_api.cc:935: `ntree_limit` is deprecated, use `iteration_range` instead.
## [15:50:52] WARNING: src/c_api/c_api.cc:935: `ntree_limit` is deprecated, use `iteration_range` instead.
## [15:50:52] WARNING: src/c_api/c_api.cc:935: `ntree_limit` is deprecated, use `iteration_range` instead.
## [15:50:52] WARNING: src/c_api/c_api.cc:935: `ntree_limit` is deprecated, use `iteration_range` instead.
## [15:50:53] WARNING: src/c_api/c_api.cc:935: `ntree_limit` is deprecated, use `iteration_range` instead.
## [15:50:53] WARNING: src/c_api/c_api.cc:935: `ntree_limit` is deprecated, use `iteration_range` instead.
## [15:50:53] WARNING: src/c_api/c_api.cc:935: `ntree_limit` is deprecated, use `iteration_range` instead.
## [15:50:53] WARNING: src/c_api/c_api.cc:935: `ntree_limit` is deprecated, use `iteration_range` instead.
## [15:50:53] WARNING: src/c_api/c_api.cc:935: `ntree_limit` is deprecated, use `iteration_range` instead.
## [15:50:53] WARNING: src/c_api/c_api.cc:935: `ntree_limit` is deprecated, use `iteration_range` instead.
## [15:50:53] WARNING: src/c_api/c_api.cc:935: `ntree_limit` is deprecated, use `iteration_range` instead.
## [15:50:53] WARNING: src/c_api/c_api.cc:935: `ntree_limit` is deprecated, use `iteration_range` instead.
## [15:50:53] WARNING: src/c_api/c_api.cc:935: `ntree_limit` is deprecated, use `iteration_range` instead.
## [15:50:53] WARNING: src/c_api/c_api.cc:935: `ntree_limit` is deprecated, use `iteration_range` instead.
## [15:50:53] WARNING: src/c_api/c_api.cc:935: `ntree_limit` is deprecated, use `iteration_range` instead.
## [15:50:53] WARNING: src/c_api/c_api.cc:935: `ntree_limit` is deprecated, use `iteration_range` instead.
## [15:50:53] WARNING: src/c_api/c_api.cc:935: `ntree_limit` is deprecated, use `iteration_range` instead.
## [15:50:53] WARNING: src/c_api/c_api.cc:935: `ntree_limit` is deprecated, use `iteration_range` instead.
## [15:50:54] WARNING: src/c_api/c_api.cc:935: `ntree_limit` is deprecated, use `iteration_range` instead.
## [15:50:54] WARNING: src/c_api/c_api.cc:935: `ntree_limit` is deprecated, use `iteration_range` instead.
## [15:50:54] WARNING: src/c_api/c_api.cc:935: `ntree_limit` is deprecated, use `iteration_range` instead.
## [15:50:54] WARNING: src/c_api/c_api.cc:935: `ntree_limit` is deprecated, use `iteration_range` instead.
## [15:50:54] WARNING: src/c_api/c_api.cc:935: `ntree_limit` is deprecated, use `iteration_range` instead.
## [15:50:54] WARNING: src/c_api/c_api.cc:935: `ntree_limit` is deprecated, use `iteration_range` instead.
## [15:50:54] WARNING: src/c_api/c_api.cc:935: `ntree_limit` is deprecated, use `iteration_range` instead.
## [15:50:54] WARNING: src/c_api/c_api.cc:935: `ntree_limit` is deprecated, use `iteration_range` instead.
## [15:50:54] WARNING: src/c_api/c_api.cc:935: `ntree_limit` is deprecated, use `iteration_range` instead.
## [15:50:54] WARNING: src/c_api/c_api.cc:935: `ntree_limit` is deprecated, use `iteration_range` instead.
## [15:50:55] WARNING: src/c_api/c_api.cc:935: `ntree_limit` is deprecated, use `iteration_range` instead.
## [15:50:55] WARNING: src/c_api/c_api.cc:935: `ntree_limit` is deprecated, use `iteration_range` instead.
## [15:50:55] WARNING: src/c_api/c_api.cc:935: `ntree_limit` is deprecated, use `iteration_range` instead.
## [15:50:55] WARNING: src/c_api/c_api.cc:935: `ntree_limit` is deprecated, use `iteration_range` instead.
## [15:50:55] WARNING: src/c_api/c_api.cc:935: `ntree_limit` is deprecated, use `iteration_range` instead.
## [15:50:55] WARNING: src/c_api/c_api.cc:935: `ntree_limit` is deprecated, use `iteration_range` instead.
## [15:50:55] WARNING: src/c_api/c_api.cc:935: `ntree_limit` is deprecated, use `iteration_range` instead.
## [15:50:55] WARNING: src/c_api/c_api.cc:935: `ntree_limit` is deprecated, use `iteration_range` instead.
## [15:50:55] WARNING: src/c_api/c_api.cc:935: `ntree_limit` is deprecated, use `iteration_range` instead.
## [15:50:55] WARNING: src/c_api/c_api.cc:935: `ntree_limit` is deprecated, use `iteration_range` instead.
## [15:50:56] WARNING: src/c_api/c_api.cc:935: `ntree_limit` is deprecated, use `iteration_range` instead.
## [15:50:56] WARNING: src/c_api/c_api.cc:935: `ntree_limit` is deprecated, use `iteration_range` instead.
## [15:50:56] WARNING: src/c_api/c_api.cc:935: `ntree_limit` is deprecated, use `iteration_range` instead.
## [15:50:56] WARNING: src/c_api/c_api.cc:935: `ntree_limit` is deprecated, use `iteration_range` instead.
## [15:50:56] WARNING: src/c_api/c_api.cc:935: `ntree_limit` is deprecated, use `iteration_range` instead.
## [15:50:56] WARNING: src/c_api/c_api.cc:935: `ntree_limit` is deprecated, use `iteration_range` instead.
## [15:50:56] WARNING: src/c_api/c_api.cc:935: `ntree_limit` is deprecated, use `iteration_range` instead.
## [15:50:56] WARNING: src/c_api/c_api.cc:935: `ntree_limit` is deprecated, use `iteration_range` instead.
## [15:50:56] WARNING: src/c_api/c_api.cc:935: `ntree_limit` is deprecated, use `iteration_range` instead.
## [15:50:56] WARNING: src/c_api/c_api.cc:935: `ntree_limit` is deprecated, use `iteration_range` instead.
## [15:50:56] WARNING: src/c_api/c_api.cc:935: `ntree_limit` is deprecated, use `iteration_range` instead.
## [15:50:56] WARNING: src/c_api/c_api.cc:935: `ntree_limit` is deprecated, use `iteration_range` instead.
## [15:50:56] WARNING: src/c_api/c_api.cc:935: `ntree_limit` is deprecated, use `iteration_range` instead.
## [15:50:56] WARNING: src/c_api/c_api.cc:935: `ntree_limit` is deprecated, use `iteration_range` instead.
## [15:50:57] WARNING: src/c_api/c_api.cc:935: `ntree_limit` is deprecated, use `iteration_range` instead.
## [15:50:57] WARNING: src/c_api/c_api.cc:935: `ntree_limit` is deprecated, use `iteration_range` instead.
## [15:50:57] WARNING: src/c_api/c_api.cc:935: `ntree_limit` is deprecated, use `iteration_range` instead.
## [15:50:57] WARNING: src/c_api/c_api.cc:935: `ntree_limit` is deprecated, use `iteration_range` instead.
## [15:50:57] WARNING: src/c_api/c_api.cc:935: `ntree_limit` is deprecated, use `iteration_range` instead.
## [15:50:57] WARNING: src/c_api/c_api.cc:935: `ntree_limit` is deprecated, use `iteration_range` instead.
## [15:50:57] WARNING: src/c_api/c_api.cc:935: `ntree_limit` is deprecated, use `iteration_range` instead.
## [15:50:57] WARNING: src/c_api/c_api.cc:935: `ntree_limit` is deprecated, use `iteration_range` instead.
## [15:50:57] WARNING: src/c_api/c_api.cc:935: `ntree_limit` is deprecated, use `iteration_range` instead.
## [15:50:57] WARNING: src/c_api/c_api.cc:935: `ntree_limit` is deprecated, use `iteration_range` instead.
## [15:50:57] WARNING: src/c_api/c_api.cc:935: `ntree_limit` is deprecated, use `iteration_range` instead.
## [15:50:57] WARNING: src/c_api/c_api.cc:935: `ntree_limit` is deprecated, use `iteration_range` instead.
## [15:50:58] WARNING: src/c_api/c_api.cc:935: `ntree_limit` is deprecated, use `iteration_range` instead.
## [15:50:58] WARNING: src/c_api/c_api.cc:935: `ntree_limit` is deprecated, use `iteration_range` instead.
## [15:50:58] WARNING: src/c_api/c_api.cc:935: `ntree_limit` is deprecated, use `iteration_range` instead.
## [15:50:58] WARNING: src/c_api/c_api.cc:935: `ntree_limit` is deprecated, use `iteration_range` instead.
## [15:50:58] WARNING: src/c_api/c_api.cc:935: `ntree_limit` is deprecated, use `iteration_range` instead.
## [15:50:58] WARNING: src/c_api/c_api.cc:935: `ntree_limit` is deprecated, use `iteration_range` instead.
## [15:50:58] WARNING: src/c_api/c_api.cc:935: `ntree_limit` is deprecated, use `iteration_range` instead.
## [15:50:58] WARNING: src/c_api/c_api.cc:935: `ntree_limit` is deprecated, use `iteration_range` instead.
## [15:50:58] WARNING: src/c_api/c_api.cc:935: `ntree_limit` is deprecated, use `iteration_range` instead.
## [15:50:58] WARNING: src/c_api/c_api.cc:935: `ntree_limit` is deprecated, use `iteration_range` instead.
## [15:50:59] WARNING: src/c_api/c_api.cc:935: `ntree_limit` is deprecated, use `iteration_range` instead.
## [15:50:59] WARNING: src/c_api/c_api.cc:935: `ntree_limit` is deprecated, use `iteration_range` instead.
## [15:50:59] WARNING: src/c_api/c_api.cc:935: `ntree_limit` is deprecated, use `iteration_range` instead.
## [15:50:59] WARNING: src/c_api/c_api.cc:935: `ntree_limit` is deprecated, use `iteration_range` instead.
## [15:50:59] WARNING: src/c_api/c_api.cc:935: `ntree_limit` is deprecated, use `iteration_range` instead.
## [15:50:59] WARNING: src/c_api/c_api.cc:935: `ntree_limit` is deprecated, use `iteration_range` instead.
## [15:50:59] WARNING: src/c_api/c_api.cc:935: `ntree_limit` is deprecated, use `iteration_range` instead.
## [15:50:59] WARNING: src/c_api/c_api.cc:935: `ntree_limit` is deprecated, use `iteration_range` instead.
## [15:50:59] WARNING: src/c_api/c_api.cc:935: `ntree_limit` is deprecated, use `iteration_range` instead.
## [15:50:59] WARNING: src/c_api/c_api.cc:935: `ntree_limit` is deprecated, use `iteration_range` instead.
## [15:50:59] WARNING: src/c_api/c_api.cc:935: `ntree_limit` is deprecated, use `iteration_range` instead.
## [15:50:59] WARNING: src/c_api/c_api.cc:935: `ntree_limit` is deprecated, use `iteration_range` instead.
## [15:50:59] WARNING: src/c_api/c_api.cc:935: `ntree_limit` is deprecated, use `iteration_range` instead.
## [15:50:59] WARNING: src/c_api/c_api.cc:935: `ntree_limit` is deprecated, use `iteration_range` instead.
## [15:51:00] WARNING: src/c_api/c_api.cc:935: `ntree_limit` is deprecated, use `iteration_range` instead.
## [15:51:00] WARNING: src/c_api/c_api.cc:935: `ntree_limit` is deprecated, use `iteration_range` instead.
## [15:51:00] WARNING: src/c_api/c_api.cc:935: `ntree_limit` is deprecated, use `iteration_range` instead.
## [15:51:00] WARNING: src/c_api/c_api.cc:935: `ntree_limit` is deprecated, use `iteration_range` instead.
## [15:51:00] WARNING: src/c_api/c_api.cc:935: `ntree_limit` is deprecated, use `iteration_range` instead.
## [15:51:00] WARNING: src/c_api/c_api.cc:935: `ntree_limit` is deprecated, use `iteration_range` instead.
## [15:51:00] WARNING: src/c_api/c_api.cc:935: `ntree_limit` is deprecated, use `iteration_range` instead.
## [15:51:00] WARNING: src/c_api/c_api.cc:935: `ntree_limit` is deprecated, use `iteration_range` instead.
## [15:51:00] WARNING: src/c_api/c_api.cc:935: `ntree_limit` is deprecated, use `iteration_range` instead.
## [15:51:00] WARNING: src/c_api/c_api.cc:935: `ntree_limit` is deprecated, use `iteration_range` instead.
## [15:51:00] WARNING: src/c_api/c_api.cc:935: `ntree_limit` is deprecated, use `iteration_range` instead.
## [15:51:00] WARNING: src/c_api/c_api.cc:935: `ntree_limit` is deprecated, use `iteration_range` instead.
## [15:51:01] WARNING: src/c_api/c_api.cc:935: `ntree_limit` is deprecated, use `iteration_range` instead.
## [15:51:01] WARNING: src/c_api/c_api.cc:935: `ntree_limit` is deprecated, use `iteration_range` instead.
## [15:51:01] WARNING: src/c_api/c_api.cc:935: `ntree_limit` is deprecated, use `iteration_range` instead.
## [15:51:01] WARNING: src/c_api/c_api.cc:935: `ntree_limit` is deprecated, use `iteration_range` instead.
## [15:51:01] WARNING: src/c_api/c_api.cc:935: `ntree_limit` is deprecated, use `iteration_range` instead.
## [15:51:01] WARNING: src/c_api/c_api.cc:935: `ntree_limit` is deprecated, use `iteration_range` instead.
## [15:51:01] WARNING: src/c_api/c_api.cc:935: `ntree_limit` is deprecated, use `iteration_range` instead.
## [15:51:01] WARNING: src/c_api/c_api.cc:935: `ntree_limit` is deprecated, use `iteration_range` instead.
## [15:51:02] WARNING: src/c_api/c_api.cc:935: `ntree_limit` is deprecated, use `iteration_range` instead.
## [15:51:02] WARNING: src/c_api/c_api.cc:935: `ntree_limit` is deprecated, use `iteration_range` instead.
## [15:51:02] WARNING: src/c_api/c_api.cc:935: `ntree_limit` is deprecated, use `iteration_range` instead.
## [15:51:02] WARNING: src/c_api/c_api.cc:935: `ntree_limit` is deprecated, use `iteration_range` instead.
## [15:51:02] WARNING: src/c_api/c_api.cc:935: `ntree_limit` is deprecated, use `iteration_range` instead.
## [15:51:02] WARNING: src/c_api/c_api.cc:935: `ntree_limit` is deprecated, use `iteration_range` instead.
## [15:51:02] WARNING: src/c_api/c_api.cc:935: `ntree_limit` is deprecated, use `iteration_range` instead.
## [15:51:02] WARNING: src/c_api/c_api.cc:935: `ntree_limit` is deprecated, use `iteration_range` instead.
## [15:51:02] WARNING: src/c_api/c_api.cc:935: `ntree_limit` is deprecated, use `iteration_range` instead.
## [15:51:02] WARNING: src/c_api/c_api.cc:935: `ntree_limit` is deprecated, use `iteration_range` instead.
## [15:51:02] WARNING: src/c_api/c_api.cc:935: `ntree_limit` is deprecated, use `iteration_range` instead.
## [15:51:02] WARNING: src/c_api/c_api.cc:935: `ntree_limit` is deprecated, use `iteration_range` instead.
## [15:51:02] WARNING: src/c_api/c_api.cc:935: `ntree_limit` is deprecated, use `iteration_range` instead.
## [15:51:02] WARNING: src/c_api/c_api.cc:935: `ntree_limit` is deprecated, use `iteration_range` instead.
## [15:51:03] WARNING: src/c_api/c_api.cc:935: `ntree_limit` is deprecated, use `iteration_range` instead.
## [15:51:03] WARNING: src/c_api/c_api.cc:935: `ntree_limit` is deprecated, use `iteration_range` instead.
## [15:51:03] WARNING: src/c_api/c_api.cc:935: `ntree_limit` is deprecated, use `iteration_range` instead.
## [15:51:03] WARNING: src/c_api/c_api.cc:935: `ntree_limit` is deprecated, use `iteration_range` instead.
## [15:51:03] WARNING: src/c_api/c_api.cc:935: `ntree_limit` is deprecated, use `iteration_range` instead.
## [15:51:03] WARNING: src/c_api/c_api.cc:935: `ntree_limit` is deprecated, use `iteration_range` instead.
## [15:51:03] WARNING: src/c_api/c_api.cc:935: `ntree_limit` is deprecated, use `iteration_range` instead.
## [15:51:03] WARNING: src/c_api/c_api.cc:935: `ntree_limit` is deprecated, use `iteration_range` instead.
## [15:51:03] WARNING: src/c_api/c_api.cc:935: `ntree_limit` is deprecated, use `iteration_range` instead.
## [15:51:03] WARNING: src/c_api/c_api.cc:935: `ntree_limit` is deprecated, use `iteration_range` instead.
## [15:51:03] WARNING: src/c_api/c_api.cc:935: `ntree_limit` is deprecated, use `iteration_range` instead.
## [15:51:03] WARNING: src/c_api/c_api.cc:935: `ntree_limit` is deprecated, use `iteration_range` instead.
## [15:51:04] WARNING: src/c_api/c_api.cc:935: `ntree_limit` is deprecated, use `iteration_range` instead.
## [15:51:04] WARNING: src/c_api/c_api.cc:935: `ntree_limit` is deprecated, use `iteration_range` instead.
## [15:51:04] WARNING: src/c_api/c_api.cc:935: `ntree_limit` is deprecated, use `iteration_range` instead.
## [15:51:04] WARNING: src/c_api/c_api.cc:935: `ntree_limit` is deprecated, use `iteration_range` instead.
## [15:51:04] WARNING: src/c_api/c_api.cc:935: `ntree_limit` is deprecated, use `iteration_range` instead.
## [15:51:04] WARNING: src/c_api/c_api.cc:935: `ntree_limit` is deprecated, use `iteration_range` instead.
## [15:51:04] WARNING: src/c_api/c_api.cc:935: `ntree_limit` is deprecated, use `iteration_range` instead.
## [15:51:04] WARNING: src/c_api/c_api.cc:935: `ntree_limit` is deprecated, use `iteration_range` instead.
## [15:51:04] WARNING: src/c_api/c_api.cc:935: `ntree_limit` is deprecated, use `iteration_range` instead.
## [15:51:04] WARNING: src/c_api/c_api.cc:935: `ntree_limit` is deprecated, use `iteration_range` instead.
## [15:51:05] WARNING: src/c_api/c_api.cc:935: `ntree_limit` is deprecated, use `iteration_range` instead.
## [15:51:05] WARNING: src/c_api/c_api.cc:935: `ntree_limit` is deprecated, use `iteration_range` instead.
## [15:51:05] WARNING: src/c_api/c_api.cc:935: `ntree_limit` is deprecated, use `iteration_range` instead.
## [15:51:05] WARNING: src/c_api/c_api.cc:935: `ntree_limit` is deprecated, use `iteration_range` instead.
## [15:51:05] WARNING: src/c_api/c_api.cc:935: `ntree_limit` is deprecated, use `iteration_range` instead.
## [15:51:05] WARNING: src/c_api/c_api.cc:935: `ntree_limit` is deprecated, use `iteration_range` instead.
## [15:51:05] WARNING: src/c_api/c_api.cc:935: `ntree_limit` is deprecated, use `iteration_range` instead.
## [15:51:05] WARNING: src/c_api/c_api.cc:935: `ntree_limit` is deprecated, use `iteration_range` instead.
## [15:51:05] WARNING: src/c_api/c_api.cc:935: `ntree_limit` is deprecated, use `iteration_range` instead.
## [15:51:05] WARNING: src/c_api/c_api.cc:935: `ntree_limit` is deprecated, use `iteration_range` instead.
## [15:51:05] WARNING: src/c_api/c_api.cc:935: `ntree_limit` is deprecated, use `iteration_range` instead.
## [15:51:05] WARNING: src/c_api/c_api.cc:935: `ntree_limit` is deprecated, use `iteration_range` instead.
## [15:51:05] WARNING: src/c_api/c_api.cc:935: `ntree_limit` is deprecated, use `iteration_range` instead.
## [15:51:05] WARNING: src/c_api/c_api.cc:935: `ntree_limit` is deprecated, use `iteration_range` instead.
## [15:51:06] WARNING: src/c_api/c_api.cc:935: `ntree_limit` is deprecated, use `iteration_range` instead.
## [15:51:06] WARNING: src/c_api/c_api.cc:935: `ntree_limit` is deprecated, use `iteration_range` instead.
## [15:51:06] WARNING: src/c_api/c_api.cc:935: `ntree_limit` is deprecated, use `iteration_range` instead.
## [15:51:06] WARNING: src/c_api/c_api.cc:935: `ntree_limit` is deprecated, use `iteration_range` instead.
## [15:51:06] WARNING: src/c_api/c_api.cc:935: `ntree_limit` is deprecated, use `iteration_range` instead.
## [15:51:06] WARNING: src/c_api/c_api.cc:935: `ntree_limit` is deprecated, use `iteration_range` instead.
## [15:51:06] WARNING: src/c_api/c_api.cc:935: `ntree_limit` is deprecated, use `iteration_range` instead.
## [15:51:06] WARNING: src/c_api/c_api.cc:935: `ntree_limit` is deprecated, use `iteration_range` instead.
## [15:51:06] WARNING: src/c_api/c_api.cc:935: `ntree_limit` is deprecated, use `iteration_range` instead.
## [15:51:06] WARNING: src/c_api/c_api.cc:935: `ntree_limit` is deprecated, use `iteration_range` instead.
## [15:51:06] WARNING: src/c_api/c_api.cc:935: `ntree_limit` is deprecated, use `iteration_range` instead.
## [15:51:06] WARNING: src/c_api/c_api.cc:935: `ntree_limit` is deprecated, use `iteration_range` instead.
## [15:51:07] WARNING: src/c_api/c_api.cc:935: `ntree_limit` is deprecated, use `iteration_range` instead.
## [15:51:07] WARNING: src/c_api/c_api.cc:935: `ntree_limit` is deprecated, use `iteration_range` instead.
## [15:51:07] WARNING: src/c_api/c_api.cc:935: `ntree_limit` is deprecated, use `iteration_range` instead.
## [15:51:07] WARNING: src/c_api/c_api.cc:935: `ntree_limit` is deprecated, use `iteration_range` instead.
## [15:51:07] WARNING: src/c_api/c_api.cc:935: `ntree_limit` is deprecated, use `iteration_range` instead.
## [15:51:07] WARNING: src/c_api/c_api.cc:935: `ntree_limit` is deprecated, use `iteration_range` instead.
## [15:51:07] WARNING: src/c_api/c_api.cc:935: `ntree_limit` is deprecated, use `iteration_range` instead.
## [15:51:07] WARNING: src/c_api/c_api.cc:935: `ntree_limit` is deprecated, use `iteration_range` instead.
## [15:51:08] WARNING: src/c_api/c_api.cc:935: `ntree_limit` is deprecated, use `iteration_range` instead.
## [15:51:08] WARNING: src/c_api/c_api.cc:935: `ntree_limit` is deprecated, use `iteration_range` instead.
## [15:51:08] WARNING: src/c_api/c_api.cc:935: `ntree_limit` is deprecated, use `iteration_range` instead.
## [15:51:08] WARNING: src/c_api/c_api.cc:935: `ntree_limit` is deprecated, use `iteration_range` instead.
## [15:51:08] WARNING: src/c_api/c_api.cc:935: `ntree_limit` is deprecated, use `iteration_range` instead.
## [15:51:08] WARNING: src/c_api/c_api.cc:935: `ntree_limit` is deprecated, use `iteration_range` instead.
## [15:51:08] WARNING: src/c_api/c_api.cc:935: `ntree_limit` is deprecated, use `iteration_range` instead.
## [15:51:08] WARNING: src/c_api/c_api.cc:935: `ntree_limit` is deprecated, use `iteration_range` instead.
## [15:51:08] WARNING: src/c_api/c_api.cc:935: `ntree_limit` is deprecated, use `iteration_range` instead.
## [15:51:08] WARNING: src/c_api/c_api.cc:935: `ntree_limit` is deprecated, use `iteration_range` instead.
## [15:51:08] WARNING: src/c_api/c_api.cc:935: `ntree_limit` is deprecated, use `iteration_range` instead.
## [15:51:08] WARNING: src/c_api/c_api.cc:935: `ntree_limit` is deprecated, use `iteration_range` instead.
## [15:51:08] WARNING: src/c_api/c_api.cc:935: `ntree_limit` is deprecated, use `iteration_range` instead.
## [15:51:08] WARNING: src/c_api/c_api.cc:935: `ntree_limit` is deprecated, use `iteration_range` instead.
## [15:51:09] WARNING: src/c_api/c_api.cc:935: `ntree_limit` is deprecated, use `iteration_range` instead.
## [15:51:09] WARNING: src/c_api/c_api.cc:935: `ntree_limit` is deprecated, use `iteration_range` instead.
## [15:51:09] WARNING: src/c_api/c_api.cc:935: `ntree_limit` is deprecated, use `iteration_range` instead.
## [15:51:09] WARNING: src/c_api/c_api.cc:935: `ntree_limit` is deprecated, use `iteration_range` instead.
## [15:51:09] WARNING: src/c_api/c_api.cc:935: `ntree_limit` is deprecated, use `iteration_range` instead.
## [15:51:09] WARNING: src/c_api/c_api.cc:935: `ntree_limit` is deprecated, use `iteration_range` instead.
## [15:51:09] WARNING: src/c_api/c_api.cc:935: `ntree_limit` is deprecated, use `iteration_range` instead.
## [15:51:09] WARNING: src/c_api/c_api.cc:935: `ntree_limit` is deprecated, use `iteration_range` instead.
## [15:51:09] WARNING: src/c_api/c_api.cc:935: `ntree_limit` is deprecated, use `iteration_range` instead.
## [15:51:09] WARNING: src/c_api/c_api.cc:935: `ntree_limit` is deprecated, use `iteration_range` instead.
## [15:51:09] WARNING: src/c_api/c_api.cc:935: `ntree_limit` is deprecated, use `iteration_range` instead.
## [15:51:09] WARNING: src/c_api/c_api.cc:935: `ntree_limit` is deprecated, use `iteration_range` instead.
## [15:51:10] WARNING: src/c_api/c_api.cc:935: `ntree_limit` is deprecated, use `iteration_range` instead.
## [15:51:10] WARNING: src/c_api/c_api.cc:935: `ntree_limit` is deprecated, use `iteration_range` instead.
## [15:51:10] WARNING: src/c_api/c_api.cc:935: `ntree_limit` is deprecated, use `iteration_range` instead.
## [15:51:10] WARNING: src/c_api/c_api.cc:935: `ntree_limit` is deprecated, use `iteration_range` instead.
## [15:51:10] WARNING: src/c_api/c_api.cc:935: `ntree_limit` is deprecated, use `iteration_range` instead.
## [15:51:10] WARNING: src/c_api/c_api.cc:935: `ntree_limit` is deprecated, use `iteration_range` instead.
## [15:51:10] WARNING: src/c_api/c_api.cc:935: `ntree_limit` is deprecated, use `iteration_range` instead.
## [15:51:10] WARNING: src/c_api/c_api.cc:935: `ntree_limit` is deprecated, use `iteration_range` instead.
## [15:51:10] WARNING: src/c_api/c_api.cc:935: `ntree_limit` is deprecated, use `iteration_range` instead.
## [15:51:10] WARNING: src/c_api/c_api.cc:935: `ntree_limit` is deprecated, use `iteration_range` instead.
## [15:51:10] WARNING: src/c_api/c_api.cc:935: `ntree_limit` is deprecated, use `iteration_range` instead.
## [15:51:10] WARNING: src/c_api/c_api.cc:935: `ntree_limit` is deprecated, use `iteration_range` instead.
## [15:51:11] WARNING: src/c_api/c_api.cc:935: `ntree_limit` is deprecated, use `iteration_range` instead.
## [15:51:11] WARNING: src/c_api/c_api.cc:935: `ntree_limit` is deprecated, use `iteration_range` instead.
## [15:51:11] WARNING: src/c_api/c_api.cc:935: `ntree_limit` is deprecated, use `iteration_range` instead.
## [15:51:11] WARNING: src/c_api/c_api.cc:935: `ntree_limit` is deprecated, use `iteration_range` instead.
## [15:51:14] WARNING: src/c_api/c_api.cc:935: `ntree_limit` is deprecated, use `iteration_range` instead.
## [15:51:14] WARNING: src/c_api/c_api.cc:935: `ntree_limit` is deprecated, use `iteration_range` instead.
## [15:51:14] WARNING: src/c_api/c_api.cc:935: `ntree_limit` is deprecated, use `iteration_range` instead.
## [15:51:14] WARNING: src/c_api/c_api.cc:935: `ntree_limit` is deprecated, use `iteration_range` instead.
## [15:51:14] WARNING: src/c_api/c_api.cc:935: `ntree_limit` is deprecated, use `iteration_range` instead.
## [15:51:14] WARNING: src/c_api/c_api.cc:935: `ntree_limit` is deprecated, use `iteration_range` instead.
## [15:51:14] WARNING: src/c_api/c_api.cc:935: `ntree_limit` is deprecated, use `iteration_range` instead.
## [15:51:14] WARNING: src/c_api/c_api.cc:935: `ntree_limit` is deprecated, use `iteration_range` instead.
## [15:51:14] WARNING: src/c_api/c_api.cc:935: `ntree_limit` is deprecated, use `iteration_range` instead.
## [15:51:14] WARNING: src/c_api/c_api.cc:935: `ntree_limit` is deprecated, use `iteration_range` instead.
## [15:51:14] WARNING: src/c_api/c_api.cc:935: `ntree_limit` is deprecated, use `iteration_range` instead.
## [15:51:14] WARNING: src/c_api/c_api.cc:935: `ntree_limit` is deprecated, use `iteration_range` instead.
## [15:51:15] WARNING: src/c_api/c_api.cc:935: `ntree_limit` is deprecated, use `iteration_range` instead.
## [15:51:15] WARNING: src/c_api/c_api.cc:935: `ntree_limit` is deprecated, use `iteration_range` instead.
## [15:51:15] WARNING: src/c_api/c_api.cc:935: `ntree_limit` is deprecated, use `iteration_range` instead.
## [15:51:15] WARNING: src/c_api/c_api.cc:935: `ntree_limit` is deprecated, use `iteration_range` instead.
## [15:51:15] WARNING: src/c_api/c_api.cc:935: `ntree_limit` is deprecated, use `iteration_range` instead.
## [15:51:15] WARNING: src/c_api/c_api.cc:935: `ntree_limit` is deprecated, use `iteration_range` instead.
## [15:51:15] WARNING: src/c_api/c_api.cc:935: `ntree_limit` is deprecated, use `iteration_range` instead.
## [15:51:15] WARNING: src/c_api/c_api.cc:935: `ntree_limit` is deprecated, use `iteration_range` instead.
## [15:51:15] WARNING: src/c_api/c_api.cc:935: `ntree_limit` is deprecated, use `iteration_range` instead.
## [15:51:15] WARNING: src/c_api/c_api.cc:935: `ntree_limit` is deprecated, use `iteration_range` instead.
## [15:51:16] WARNING: src/c_api/c_api.cc:935: `ntree_limit` is deprecated, use `iteration_range` instead.
## [15:51:16] WARNING: src/c_api/c_api.cc:935: `ntree_limit` is deprecated, use `iteration_range` instead.
## [15:51:16] WARNING: src/c_api/c_api.cc:935: `ntree_limit` is deprecated, use `iteration_range` instead.
## [15:51:16] WARNING: src/c_api/c_api.cc:935: `ntree_limit` is deprecated, use `iteration_range` instead.
## [15:51:16] WARNING: src/c_api/c_api.cc:935: `ntree_limit` is deprecated, use `iteration_range` instead.
## [15:51:16] WARNING: src/c_api/c_api.cc:935: `ntree_limit` is deprecated, use `iteration_range` instead.
## [15:51:16] WARNING: src/c_api/c_api.cc:935: `ntree_limit` is deprecated, use `iteration_range` instead.
## [15:51:16] WARNING: src/c_api/c_api.cc:935: `ntree_limit` is deprecated, use `iteration_range` instead.
## [15:51:16] WARNING: src/c_api/c_api.cc:935: `ntree_limit` is deprecated, use `iteration_range` instead.
## [15:51:16] WARNING: src/c_api/c_api.cc:935: `ntree_limit` is deprecated, use `iteration_range` instead.
## [15:51:17] WARNING: src/c_api/c_api.cc:935: `ntree_limit` is deprecated, use `iteration_range` instead.
## [15:51:17] WARNING: src/c_api/c_api.cc:935: `ntree_limit` is deprecated, use `iteration_range` instead.
## [15:51:17] WARNING: src/c_api/c_api.cc:935: `ntree_limit` is deprecated, use `iteration_range` instead.
## [15:51:17] WARNING: src/c_api/c_api.cc:935: `ntree_limit` is deprecated, use `iteration_range` instead.
## [15:51:17] WARNING: src/c_api/c_api.cc:935: `ntree_limit` is deprecated, use `iteration_range` instead.
## [15:51:17] WARNING: src/c_api/c_api.cc:935: `ntree_limit` is deprecated, use `iteration_range` instead.
## [15:51:17] WARNING: src/c_api/c_api.cc:935: `ntree_limit` is deprecated, use `iteration_range` instead.
## [15:51:17] WARNING: src/c_api/c_api.cc:935: `ntree_limit` is deprecated, use `iteration_range` instead.
## [15:51:17] WARNING: src/c_api/c_api.cc:935: `ntree_limit` is deprecated, use `iteration_range` instead.
## [15:51:17] WARNING: src/c_api/c_api.cc:935: `ntree_limit` is deprecated, use `iteration_range` instead.
## [15:51:17] WARNING: src/c_api/c_api.cc:935: `ntree_limit` is deprecated, use `iteration_range` instead.
## [15:51:17] WARNING: src/c_api/c_api.cc:935: `ntree_limit` is deprecated, use `iteration_range` instead.
## [15:51:17] WARNING: src/c_api/c_api.cc:935: `ntree_limit` is deprecated, use `iteration_range` instead.
## [15:51:17] WARNING: src/c_api/c_api.cc:935: `ntree_limit` is deprecated, use `iteration_range` instead.
## [15:51:18] WARNING: src/c_api/c_api.cc:935: `ntree_limit` is deprecated, use `iteration_range` instead.
## [15:51:18] WARNING: src/c_api/c_api.cc:935: `ntree_limit` is deprecated, use `iteration_range` instead.
## [15:51:18] WARNING: src/c_api/c_api.cc:935: `ntree_limit` is deprecated, use `iteration_range` instead.
## [15:51:18] WARNING: src/c_api/c_api.cc:935: `ntree_limit` is deprecated, use `iteration_range` instead.
## [15:51:18] WARNING: src/c_api/c_api.cc:935: `ntree_limit` is deprecated, use `iteration_range` instead.
## [15:51:18] WARNING: src/c_api/c_api.cc:935: `ntree_limit` is deprecated, use `iteration_range` instead.
## [15:51:18] WARNING: src/c_api/c_api.cc:935: `ntree_limit` is deprecated, use `iteration_range` instead.
## [15:51:18] WARNING: src/c_api/c_api.cc:935: `ntree_limit` is deprecated, use `iteration_range` instead.
## [15:51:18] WARNING: src/c_api/c_api.cc:935: `ntree_limit` is deprecated, use `iteration_range` instead.
## [15:51:18] WARNING: src/c_api/c_api.cc:935: `ntree_limit` is deprecated, use `iteration_range` instead.
## [15:51:18] WARNING: src/c_api/c_api.cc:935: `ntree_limit` is deprecated, use `iteration_range` instead.
## [15:51:18] WARNING: src/c_api/c_api.cc:935: `ntree_limit` is deprecated, use `iteration_range` instead.
## [15:51:19] WARNING: src/c_api/c_api.cc:935: `ntree_limit` is deprecated, use `iteration_range` instead.
## [15:51:19] WARNING: src/c_api/c_api.cc:935: `ntree_limit` is deprecated, use `iteration_range` instead.
## [15:51:19] WARNING: src/c_api/c_api.cc:935: `ntree_limit` is deprecated, use `iteration_range` instead.
## [15:51:19] WARNING: src/c_api/c_api.cc:935: `ntree_limit` is deprecated, use `iteration_range` instead.
## [15:51:19] WARNING: src/c_api/c_api.cc:935: `ntree_limit` is deprecated, use `iteration_range` instead.
## [15:51:19] WARNING: src/c_api/c_api.cc:935: `ntree_limit` is deprecated, use `iteration_range` instead.
## [15:51:19] WARNING: src/c_api/c_api.cc:935: `ntree_limit` is deprecated, use `iteration_range` instead.
## [15:51:19] WARNING: src/c_api/c_api.cc:935: `ntree_limit` is deprecated, use `iteration_range` instead.
## [15:51:19] WARNING: src/c_api/c_api.cc:935: `ntree_limit` is deprecated, use `iteration_range` instead.
## [15:51:19] WARNING: src/c_api/c_api.cc:935: `ntree_limit` is deprecated, use `iteration_range` instead.
## [15:51:20] WARNING: src/c_api/c_api.cc:935: `ntree_limit` is deprecated, use `iteration_range` instead.
## [15:51:20] WARNING: src/c_api/c_api.cc:935: `ntree_limit` is deprecated, use `iteration_range` instead.
## [15:51:20] WARNING: src/c_api/c_api.cc:935: `ntree_limit` is deprecated, use `iteration_range` instead.
## [15:51:20] WARNING: src/c_api/c_api.cc:935: `ntree_limit` is deprecated, use `iteration_range` instead.
## [15:51:20] WARNING: src/c_api/c_api.cc:935: `ntree_limit` is deprecated, use `iteration_range` instead.
## [15:51:20] WARNING: src/c_api/c_api.cc:935: `ntree_limit` is deprecated, use `iteration_range` instead.
## [15:51:20] WARNING: src/c_api/c_api.cc:935: `ntree_limit` is deprecated, use `iteration_range` instead.
## [15:51:20] WARNING: src/c_api/c_api.cc:935: `ntree_limit` is deprecated, use `iteration_range` instead.
## [15:51:20] WARNING: src/c_api/c_api.cc:935: `ntree_limit` is deprecated, use `iteration_range` instead.
## [15:51:20] WARNING: src/c_api/c_api.cc:935: `ntree_limit` is deprecated, use `iteration_range` instead.
## [15:51:20] WARNING: src/c_api/c_api.cc:935: `ntree_limit` is deprecated, use `iteration_range` instead.
## [15:51:20] WARNING: src/c_api/c_api.cc:935: `ntree_limit` is deprecated, use `iteration_range` instead.
## [15:51:20] WARNING: src/c_api/c_api.cc:935: `ntree_limit` is deprecated, use `iteration_range` instead.
## [15:51:20] WARNING: src/c_api/c_api.cc:935: `ntree_limit` is deprecated, use `iteration_range` instead.
## [15:51:21] WARNING: src/c_api/c_api.cc:935: `ntree_limit` is deprecated, use `iteration_range` instead.
## [15:51:21] WARNING: src/c_api/c_api.cc:935: `ntree_limit` is deprecated, use `iteration_range` instead.
## [15:51:21] WARNING: src/c_api/c_api.cc:935: `ntree_limit` is deprecated, use `iteration_range` instead.
## [15:51:21] WARNING: src/c_api/c_api.cc:935: `ntree_limit` is deprecated, use `iteration_range` instead.
## [15:51:21] WARNING: src/c_api/c_api.cc:935: `ntree_limit` is deprecated, use `iteration_range` instead.
## [15:51:21] WARNING: src/c_api/c_api.cc:935: `ntree_limit` is deprecated, use `iteration_range` instead.
## [15:51:21] WARNING: src/c_api/c_api.cc:935: `ntree_limit` is deprecated, use `iteration_range` instead.
## [15:51:21] WARNING: src/c_api/c_api.cc:935: `ntree_limit` is deprecated, use `iteration_range` instead.
## [15:51:21] WARNING: src/c_api/c_api.cc:935: `ntree_limit` is deprecated, use `iteration_range` instead.
## [15:51:21] WARNING: src/c_api/c_api.cc:935: `ntree_limit` is deprecated, use `iteration_range` instead.
## [15:51:22] WARNING: src/c_api/c_api.cc:935: `ntree_limit` is deprecated, use `iteration_range` instead.
## [15:51:22] WARNING: src/c_api/c_api.cc:935: `ntree_limit` is deprecated, use `iteration_range` instead.
## [15:51:22] WARNING: src/c_api/c_api.cc:935: `ntree_limit` is deprecated, use `iteration_range` instead.
## [15:51:22] WARNING: src/c_api/c_api.cc:935: `ntree_limit` is deprecated, use `iteration_range` instead.
## [15:51:22] WARNING: src/c_api/c_api.cc:935: `ntree_limit` is deprecated, use `iteration_range` instead.
## [15:51:22] WARNING: src/c_api/c_api.cc:935: `ntree_limit` is deprecated, use `iteration_range` instead.
## [15:51:22] WARNING: src/c_api/c_api.cc:935: `ntree_limit` is deprecated, use `iteration_range` instead.
## [15:51:22] WARNING: src/c_api/c_api.cc:935: `ntree_limit` is deprecated, use `iteration_range` instead.
## [15:51:22] WARNING: src/c_api/c_api.cc:935: `ntree_limit` is deprecated, use `iteration_range` instead.
## [15:51:22] WARNING: src/c_api/c_api.cc:935: `ntree_limit` is deprecated, use `iteration_range` instead.
## [15:51:23] WARNING: src/c_api/c_api.cc:935: `ntree_limit` is deprecated, use `iteration_range` instead.
## [15:51:23] WARNING: src/c_api/c_api.cc:935: `ntree_limit` is deprecated, use `iteration_range` instead.
## [15:51:23] WARNING: src/c_api/c_api.cc:935: `ntree_limit` is deprecated, use `iteration_range` instead.
## [15:51:23] WARNING: src/c_api/c_api.cc:935: `ntree_limit` is deprecated, use `iteration_range` instead.
## [15:51:23] WARNING: src/c_api/c_api.cc:935: `ntree_limit` is deprecated, use `iteration_range` instead.
## [15:51:23] WARNING: src/c_api/c_api.cc:935: `ntree_limit` is deprecated, use `iteration_range` instead.
## [15:51:23] WARNING: src/c_api/c_api.cc:935: `ntree_limit` is deprecated, use `iteration_range` instead.
## [15:51:23] WARNING: src/c_api/c_api.cc:935: `ntree_limit` is deprecated, use `iteration_range` instead.
## [15:51:23] WARNING: src/c_api/c_api.cc:935: `ntree_limit` is deprecated, use `iteration_range` instead.
## [15:51:23] WARNING: src/c_api/c_api.cc:935: `ntree_limit` is deprecated, use `iteration_range` instead.
## [15:51:23] WARNING: src/c_api/c_api.cc:935: `ntree_limit` is deprecated, use `iteration_range` instead.
## [15:51:23] WARNING: src/c_api/c_api.cc:935: `ntree_limit` is deprecated, use `iteration_range` instead.
## [15:51:24] WARNING: src/c_api/c_api.cc:935: `ntree_limit` is deprecated, use `iteration_range` instead.
## [15:51:24] WARNING: src/c_api/c_api.cc:935: `ntree_limit` is deprecated, use `iteration_range` instead.
## [15:51:24] WARNING: src/c_api/c_api.cc:935: `ntree_limit` is deprecated, use `iteration_range` instead.
## [15:51:24] WARNING: src/c_api/c_api.cc:935: `ntree_limit` is deprecated, use `iteration_range` instead.
## [15:51:24] WARNING: src/c_api/c_api.cc:935: `ntree_limit` is deprecated, use `iteration_range` instead.
## [15:51:24] WARNING: src/c_api/c_api.cc:935: `ntree_limit` is deprecated, use `iteration_range` instead.
## [15:51:24] WARNING: src/c_api/c_api.cc:935: `ntree_limit` is deprecated, use `iteration_range` instead.
## [15:51:24] WARNING: src/c_api/c_api.cc:935: `ntree_limit` is deprecated, use `iteration_range` instead.
## [15:51:24] WARNING: src/c_api/c_api.cc:935: `ntree_limit` is deprecated, use `iteration_range` instead.
## [15:51:24] WARNING: src/c_api/c_api.cc:935: `ntree_limit` is deprecated, use `iteration_range` instead.
## [15:51:24] WARNING: src/c_api/c_api.cc:935: `ntree_limit` is deprecated, use `iteration_range` instead.
## [15:51:24] WARNING: src/c_api/c_api.cc:935: `ntree_limit` is deprecated, use `iteration_range` instead.
## [15:51:24] WARNING: src/c_api/c_api.cc:935: `ntree_limit` is deprecated, use `iteration_range` instead.
## [15:51:24] WARNING: src/c_api/c_api.cc:935: `ntree_limit` is deprecated, use `iteration_range` instead.
## [15:51:25] WARNING: src/c_api/c_api.cc:935: `ntree_limit` is deprecated, use `iteration_range` instead.
## [15:51:25] WARNING: src/c_api/c_api.cc:935: `ntree_limit` is deprecated, use `iteration_range` instead.
## [15:51:25] WARNING: src/c_api/c_api.cc:935: `ntree_limit` is deprecated, use `iteration_range` instead.
## [15:51:25] WARNING: src/c_api/c_api.cc:935: `ntree_limit` is deprecated, use `iteration_range` instead.
## [15:51:25] WARNING: src/c_api/c_api.cc:935: `ntree_limit` is deprecated, use `iteration_range` instead.
## [15:51:25] WARNING: src/c_api/c_api.cc:935: `ntree_limit` is deprecated, use `iteration_range` instead.
## [15:51:25] WARNING: src/c_api/c_api.cc:935: `ntree_limit` is deprecated, use `iteration_range` instead.
## [15:51:25] WARNING: src/c_api/c_api.cc:935: `ntree_limit` is deprecated, use `iteration_range` instead.
## [15:51:25] WARNING: src/c_api/c_api.cc:935: `ntree_limit` is deprecated, use `iteration_range` instead.
## [15:51:25] WARNING: src/c_api/c_api.cc:935: `ntree_limit` is deprecated, use `iteration_range` instead.
## [15:51:26] WARNING: src/c_api/c_api.cc:935: `ntree_limit` is deprecated, use `iteration_range` instead.
## [15:51:26] WARNING: src/c_api/c_api.cc:935: `ntree_limit` is deprecated, use `iteration_range` instead.
## [15:51:26] WARNING: src/c_api/c_api.cc:935: `ntree_limit` is deprecated, use `iteration_range` instead.
## [15:51:26] WARNING: src/c_api/c_api.cc:935: `ntree_limit` is deprecated, use `iteration_range` instead.
## [15:51:26] WARNING: src/c_api/c_api.cc:935: `ntree_limit` is deprecated, use `iteration_range` instead.
## [15:51:26] WARNING: src/c_api/c_api.cc:935: `ntree_limit` is deprecated, use `iteration_range` instead.
## [15:51:26] WARNING: src/c_api/c_api.cc:935: `ntree_limit` is deprecated, use `iteration_range` instead.
## [15:51:26] WARNING: src/c_api/c_api.cc:935: `ntree_limit` is deprecated, use `iteration_range` instead.
## [15:51:26] WARNING: src/c_api/c_api.cc:935: `ntree_limit` is deprecated, use `iteration_range` instead.
## [15:51:26] WARNING: src/c_api/c_api.cc:935: `ntree_limit` is deprecated, use `iteration_range` instead.
## [15:51:26] WARNING: src/c_api/c_api.cc:935: `ntree_limit` is deprecated, use `iteration_range` instead.
## [15:51:26] WARNING: src/c_api/c_api.cc:935: `ntree_limit` is deprecated, use `iteration_range` instead.
## [15:51:26] WARNING: src/c_api/c_api.cc:935: `ntree_limit` is deprecated, use `iteration_range` instead.
## [15:51:26] WARNING: src/c_api/c_api.cc:935: `ntree_limit` is deprecated, use `iteration_range` instead.
## [15:51:27] WARNING: src/c_api/c_api.cc:935: `ntree_limit` is deprecated, use `iteration_range` instead.
## [15:51:27] WARNING: src/c_api/c_api.cc:935: `ntree_limit` is deprecated, use `iteration_range` instead.
## [15:51:27] WARNING: src/c_api/c_api.cc:935: `ntree_limit` is deprecated, use `iteration_range` instead.
## [15:51:27] WARNING: src/c_api/c_api.cc:935: `ntree_limit` is deprecated, use `iteration_range` instead.
## [15:51:27] WARNING: src/c_api/c_api.cc:935: `ntree_limit` is deprecated, use `iteration_range` instead.
## [15:51:27] WARNING: src/c_api/c_api.cc:935: `ntree_limit` is deprecated, use `iteration_range` instead.
## [15:51:27] WARNING: src/c_api/c_api.cc:935: `ntree_limit` is deprecated, use `iteration_range` instead.
## [15:51:27] WARNING: src/c_api/c_api.cc:935: `ntree_limit` is deprecated, use `iteration_range` instead.
## [15:51:27] WARNING: src/c_api/c_api.cc:935: `ntree_limit` is deprecated, use `iteration_range` instead.
## [15:51:27] WARNING: src/c_api/c_api.cc:935: `ntree_limit` is deprecated, use `iteration_range` instead.
## [15:51:27] WARNING: src/c_api/c_api.cc:935: `ntree_limit` is deprecated, use `iteration_range` instead.
## [15:51:27] WARNING: src/c_api/c_api.cc:935: `ntree_limit` is deprecated, use `iteration_range` instead.
## [15:51:28] WARNING: src/c_api/c_api.cc:935: `ntree_limit` is deprecated, use `iteration_range` instead.
## [15:51:28] WARNING: src/c_api/c_api.cc:935: `ntree_limit` is deprecated, use `iteration_range` instead.
## [15:51:28] WARNING: src/c_api/c_api.cc:935: `ntree_limit` is deprecated, use `iteration_range` instead.
## [15:51:28] WARNING: src/c_api/c_api.cc:935: `ntree_limit` is deprecated, use `iteration_range` instead.
## [15:51:28] WARNING: src/c_api/c_api.cc:935: `ntree_limit` is deprecated, use `iteration_range` instead.
## [15:51:28] WARNING: src/c_api/c_api.cc:935: `ntree_limit` is deprecated, use `iteration_range` instead.
## [15:51:28] WARNING: src/c_api/c_api.cc:935: `ntree_limit` is deprecated, use `iteration_range` instead.
## [15:51:28] WARNING: src/c_api/c_api.cc:935: `ntree_limit` is deprecated, use `iteration_range` instead.
## [15:51:28] WARNING: src/c_api/c_api.cc:935: `ntree_limit` is deprecated, use `iteration_range` instead.
## [15:51:28] WARNING: src/c_api/c_api.cc:935: `ntree_limit` is deprecated, use `iteration_range` instead.
## [15:51:29] WARNING: src/c_api/c_api.cc:935: `ntree_limit` is deprecated, use `iteration_range` instead.
## [15:51:29] WARNING: src/c_api/c_api.cc:935: `ntree_limit` is deprecated, use `iteration_range` instead.
## [15:51:29] WARNING: src/c_api/c_api.cc:935: `ntree_limit` is deprecated, use `iteration_range` instead.
## [15:51:29] WARNING: src/c_api/c_api.cc:935: `ntree_limit` is deprecated, use `iteration_range` instead.
## [15:51:29] WARNING: src/c_api/c_api.cc:935: `ntree_limit` is deprecated, use `iteration_range` instead.
## [15:51:29] WARNING: src/c_api/c_api.cc:935: `ntree_limit` is deprecated, use `iteration_range` instead.
## [15:51:29] WARNING: src/c_api/c_api.cc:935: `ntree_limit` is deprecated, use `iteration_range` instead.
## [15:51:29] WARNING: src/c_api/c_api.cc:935: `ntree_limit` is deprecated, use `iteration_range` instead.
## [15:51:29] WARNING: src/c_api/c_api.cc:935: `ntree_limit` is deprecated, use `iteration_range` instead.
## [15:51:29] WARNING: src/c_api/c_api.cc:935: `ntree_limit` is deprecated, use `iteration_range` instead.
## [15:51:29] WARNING: src/c_api/c_api.cc:935: `ntree_limit` is deprecated, use `iteration_range` instead.
## [15:51:29] WARNING: src/c_api/c_api.cc:935: `ntree_limit` is deprecated, use `iteration_range` instead.
## [15:51:30] WARNING: src/c_api/c_api.cc:935: `ntree_limit` is deprecated, use `iteration_range` instead.
## [15:51:30] WARNING: src/c_api/c_api.cc:935: `ntree_limit` is deprecated, use `iteration_range` instead.
## [15:51:30] WARNING: src/c_api/c_api.cc:935: `ntree_limit` is deprecated, use `iteration_range` instead.
## [15:51:30] WARNING: src/c_api/c_api.cc:935: `ntree_limit` is deprecated, use `iteration_range` instead.
## [15:51:30] WARNING: src/c_api/c_api.cc:935: `ntree_limit` is deprecated, use `iteration_range` instead.
## [15:51:30] WARNING: src/c_api/c_api.cc:935: `ntree_limit` is deprecated, use `iteration_range` instead.
## [15:51:30] WARNING: src/c_api/c_api.cc:935: `ntree_limit` is deprecated, use `iteration_range` instead.
## [15:51:30] WARNING: src/c_api/c_api.cc:935: `ntree_limit` is deprecated, use `iteration_range` instead.
## [15:51:30] WARNING: src/c_api/c_api.cc:935: `ntree_limit` is deprecated, use `iteration_range` instead.
## [15:51:30] WARNING: src/c_api/c_api.cc:935: `ntree_limit` is deprecated, use `iteration_range` instead.
## [15:51:30] WARNING: src/c_api/c_api.cc:935: `ntree_limit` is deprecated, use `iteration_range` instead.
## [15:51:30] WARNING: src/c_api/c_api.cc:935: `ntree_limit` is deprecated, use `iteration_range` instead.
## [15:51:30] WARNING: src/c_api/c_api.cc:935: `ntree_limit` is deprecated, use `iteration_range` instead.
## [15:51:30] WARNING: src/c_api/c_api.cc:935: `ntree_limit` is deprecated, use `iteration_range` instead.
## [15:51:31] WARNING: src/c_api/c_api.cc:935: `ntree_limit` is deprecated, use `iteration_range` instead.
## [15:51:31] WARNING: src/c_api/c_api.cc:935: `ntree_limit` is deprecated, use `iteration_range` instead.
## [15:51:31] WARNING: src/c_api/c_api.cc:935: `ntree_limit` is deprecated, use `iteration_range` instead.
## [15:51:31] WARNING: src/c_api/c_api.cc:935: `ntree_limit` is deprecated, use `iteration_range` instead.
## [15:51:31] WARNING: src/c_api/c_api.cc:935: `ntree_limit` is deprecated, use `iteration_range` instead.
## [15:51:31] WARNING: src/c_api/c_api.cc:935: `ntree_limit` is deprecated, use `iteration_range` instead.
## [15:51:31] WARNING: src/c_api/c_api.cc:935: `ntree_limit` is deprecated, use `iteration_range` instead.
## [15:51:31] WARNING: src/c_api/c_api.cc:935: `ntree_limit` is deprecated, use `iteration_range` instead.
## [15:51:31] WARNING: src/c_api/c_api.cc:935: `ntree_limit` is deprecated, use `iteration_range` instead.
## [15:51:31] WARNING: src/c_api/c_api.cc:935: `ntree_limit` is deprecated, use `iteration_range` instead.
## [15:51:32] WARNING: src/c_api/c_api.cc:935: `ntree_limit` is deprecated, use `iteration_range` instead.
## [15:51:32] WARNING: src/c_api/c_api.cc:935: `ntree_limit` is deprecated, use `iteration_range` instead.
## [15:51:32] WARNING: src/c_api/c_api.cc:935: `ntree_limit` is deprecated, use `iteration_range` instead.
## [15:51:32] WARNING: src/c_api/c_api.cc:935: `ntree_limit` is deprecated, use `iteration_range` instead.
## [15:51:32] WARNING: src/c_api/c_api.cc:935: `ntree_limit` is deprecated, use `iteration_range` instead.
## [15:51:32] WARNING: src/c_api/c_api.cc:935: `ntree_limit` is deprecated, use `iteration_range` instead.
## [15:51:32] WARNING: src/c_api/c_api.cc:935: `ntree_limit` is deprecated, use `iteration_range` instead.
## [15:51:32] WARNING: src/c_api/c_api.cc:935: `ntree_limit` is deprecated, use `iteration_range` instead.
## [15:51:32] WARNING: src/c_api/c_api.cc:935: `ntree_limit` is deprecated, use `iteration_range` instead.
## [15:51:32] WARNING: src/c_api/c_api.cc:935: `ntree_limit` is deprecated, use `iteration_range` instead.
## [15:51:32] WARNING: src/c_api/c_api.cc:935: `ntree_limit` is deprecated, use `iteration_range` instead.
## [15:51:32] WARNING: src/c_api/c_api.cc:935: `ntree_limit` is deprecated, use `iteration_range` instead.
## [15:51:32] WARNING: src/c_api/c_api.cc:935: `ntree_limit` is deprecated, use `iteration_range` instead.
## [15:51:32] WARNING: src/c_api/c_api.cc:935: `ntree_limit` is deprecated, use `iteration_range` instead.
## [15:51:33] WARNING: src/c_api/c_api.cc:935: `ntree_limit` is deprecated, use `iteration_range` instead.
## [15:51:33] WARNING: src/c_api/c_api.cc:935: `ntree_limit` is deprecated, use `iteration_range` instead.
## [15:51:33] WARNING: src/c_api/c_api.cc:935: `ntree_limit` is deprecated, use `iteration_range` instead.
## [15:51:33] WARNING: src/c_api/c_api.cc:935: `ntree_limit` is deprecated, use `iteration_range` instead.
## [15:51:33] WARNING: src/c_api/c_api.cc:935: `ntree_limit` is deprecated, use `iteration_range` instead.
## [15:51:33] WARNING: src/c_api/c_api.cc:935: `ntree_limit` is deprecated, use `iteration_range` instead.
## [15:51:33] WARNING: src/c_api/c_api.cc:935: `ntree_limit` is deprecated, use `iteration_range` instead.
## [15:51:33] WARNING: src/c_api/c_api.cc:935: `ntree_limit` is deprecated, use `iteration_range` instead.
## [15:51:33] WARNING: src/c_api/c_api.cc:935: `ntree_limit` is deprecated, use `iteration_range` instead.
## [15:51:33] WARNING: src/c_api/c_api.cc:935: `ntree_limit` is deprecated, use `iteration_range` instead.
## [15:51:33] WARNING: src/c_api/c_api.cc:935: `ntree_limit` is deprecated, use `iteration_range` instead.
## [15:51:33] WARNING: src/c_api/c_api.cc:935: `ntree_limit` is deprecated, use `iteration_range` instead.
## [15:51:34] WARNING: src/c_api/c_api.cc:935: `ntree_limit` is deprecated, use `iteration_range` instead.
## [15:51:34] WARNING: src/c_api/c_api.cc:935: `ntree_limit` is deprecated, use `iteration_range` instead.
## [15:51:34] WARNING: src/c_api/c_api.cc:935: `ntree_limit` is deprecated, use `iteration_range` instead.
## [15:51:34] WARNING: src/c_api/c_api.cc:935: `ntree_limit` is deprecated, use `iteration_range` instead.
## [15:51:34] WARNING: src/c_api/c_api.cc:935: `ntree_limit` is deprecated, use `iteration_range` instead.
## [15:51:34] WARNING: src/c_api/c_api.cc:935: `ntree_limit` is deprecated, use `iteration_range` instead.
## [15:51:34] WARNING: src/c_api/c_api.cc:935: `ntree_limit` is deprecated, use `iteration_range` instead.
## [15:51:34] WARNING: src/c_api/c_api.cc:935: `ntree_limit` is deprecated, use `iteration_range` instead.
## [15:51:34] WARNING: src/c_api/c_api.cc:935: `ntree_limit` is deprecated, use `iteration_range` instead.
## [15:51:34] WARNING: src/c_api/c_api.cc:935: `ntree_limit` is deprecated, use `iteration_range` instead.
## [15:51:35] WARNING: src/c_api/c_api.cc:935: `ntree_limit` is deprecated, use `iteration_range` instead.
## [15:51:35] WARNING: src/c_api/c_api.cc:935: `ntree_limit` is deprecated, use `iteration_range` instead.
## [15:51:35] WARNING: src/c_api/c_api.cc:935: `ntree_limit` is deprecated, use `iteration_range` instead.
## [15:51:35] WARNING: src/c_api/c_api.cc:935: `ntree_limit` is deprecated, use `iteration_range` instead.
## [15:51:35] WARNING: src/c_api/c_api.cc:935: `ntree_limit` is deprecated, use `iteration_range` instead.
## [15:51:35] WARNING: src/c_api/c_api.cc:935: `ntree_limit` is deprecated, use `iteration_range` instead.
## [15:51:35] WARNING: src/c_api/c_api.cc:935: `ntree_limit` is deprecated, use `iteration_range` instead.
## [15:51:35] WARNING: src/c_api/c_api.cc:935: `ntree_limit` is deprecated, use `iteration_range` instead.
## [15:51:35] WARNING: src/c_api/c_api.cc:935: `ntree_limit` is deprecated, use `iteration_range` instead.
## [15:51:35] WARNING: src/c_api/c_api.cc:935: `ntree_limit` is deprecated, use `iteration_range` instead.
## [15:51:36] WARNING: src/c_api/c_api.cc:935: `ntree_limit` is deprecated, use `iteration_range` instead.
## [15:51:36] WARNING: src/c_api/c_api.cc:935: `ntree_limit` is deprecated, use `iteration_range` instead.
## [15:51:36] WARNING: src/c_api/c_api.cc:935: `ntree_limit` is deprecated, use `iteration_range` instead.
## [15:51:36] WARNING: src/c_api/c_api.cc:935: `ntree_limit` is deprecated, use `iteration_range` instead.
## [15:51:36] WARNING: src/c_api/c_api.cc:935: `ntree_limit` is deprecated, use `iteration_range` instead.
## [15:51:36] WARNING: src/c_api/c_api.cc:935: `ntree_limit` is deprecated, use `iteration_range` instead.
## [15:51:36] WARNING: src/c_api/c_api.cc:935: `ntree_limit` is deprecated, use `iteration_range` instead.
## [15:51:36] WARNING: src/c_api/c_api.cc:935: `ntree_limit` is deprecated, use `iteration_range` instead.
## [15:51:36] WARNING: src/c_api/c_api.cc:935: `ntree_limit` is deprecated, use `iteration_range` instead.
## [15:51:36] WARNING: src/c_api/c_api.cc:935: `ntree_limit` is deprecated, use `iteration_range` instead.
## [15:51:36] WARNING: src/c_api/c_api.cc:935: `ntree_limit` is deprecated, use `iteration_range` instead.
## [15:51:36] WARNING: src/c_api/c_api.cc:935: `ntree_limit` is deprecated, use `iteration_range` instead.
## [15:51:36] WARNING: src/c_api/c_api.cc:935: `ntree_limit` is deprecated, use `iteration_range` instead.
## [15:51:36] WARNING: src/c_api/c_api.cc:935: `ntree_limit` is deprecated, use `iteration_range` instead.
## [15:51:37] WARNING: src/c_api/c_api.cc:935: `ntree_limit` is deprecated, use `iteration_range` instead.
## [15:51:37] WARNING: src/c_api/c_api.cc:935: `ntree_limit` is deprecated, use `iteration_range` instead.
## [15:51:37] WARNING: src/c_api/c_api.cc:935: `ntree_limit` is deprecated, use `iteration_range` instead.
## [15:51:37] WARNING: src/c_api/c_api.cc:935: `ntree_limit` is deprecated, use `iteration_range` instead.
## [15:51:37] WARNING: src/c_api/c_api.cc:935: `ntree_limit` is deprecated, use `iteration_range` instead.
## [15:51:37] WARNING: src/c_api/c_api.cc:935: `ntree_limit` is deprecated, use `iteration_range` instead.
## [15:51:37] WARNING: src/c_api/c_api.cc:935: `ntree_limit` is deprecated, use `iteration_range` instead.
## [15:51:37] WARNING: src/c_api/c_api.cc:935: `ntree_limit` is deprecated, use `iteration_range` instead.
## [15:51:37] WARNING: src/c_api/c_api.cc:935: `ntree_limit` is deprecated, use `iteration_range` instead.
## [15:51:37] WARNING: src/c_api/c_api.cc:935: `ntree_limit` is deprecated, use `iteration_range` instead.
## [15:51:38] WARNING: src/c_api/c_api.cc:935: `ntree_limit` is deprecated, use `iteration_range` instead.
## [15:51:38] WARNING: src/c_api/c_api.cc:935: `ntree_limit` is deprecated, use `iteration_range` instead.
## [15:51:38] WARNING: src/c_api/c_api.cc:935: `ntree_limit` is deprecated, use `iteration_range` instead.
## [15:51:38] WARNING: src/c_api/c_api.cc:935: `ntree_limit` is deprecated, use `iteration_range` instead.
## [15:51:38] WARNING: src/c_api/c_api.cc:935: `ntree_limit` is deprecated, use `iteration_range` instead.
## [15:51:38] WARNING: src/c_api/c_api.cc:935: `ntree_limit` is deprecated, use `iteration_range` instead.
## [15:51:38] WARNING: src/c_api/c_api.cc:935: `ntree_limit` is deprecated, use `iteration_range` instead.
## [15:51:38] WARNING: src/c_api/c_api.cc:935: `ntree_limit` is deprecated, use `iteration_range` instead.
## [15:51:41] WARNING: src/c_api/c_api.cc:935: `ntree_limit` is deprecated, use `iteration_range` instead.
## [15:51:41] WARNING: src/c_api/c_api.cc:935: `ntree_limit` is deprecated, use `iteration_range` instead.
## [15:51:41] WARNING: src/c_api/c_api.cc:935: `ntree_limit` is deprecated, use `iteration_range` instead.
## [15:51:41] WARNING: src/c_api/c_api.cc:935: `ntree_limit` is deprecated, use `iteration_range` instead.
## [15:51:41] WARNING: src/c_api/c_api.cc:935: `ntree_limit` is deprecated, use `iteration_range` instead.
## [15:51:41] WARNING: src/c_api/c_api.cc:935: `ntree_limit` is deprecated, use `iteration_range` instead.
## [15:51:41] WARNING: src/c_api/c_api.cc:935: `ntree_limit` is deprecated, use `iteration_range` instead.
## [15:51:41] WARNING: src/c_api/c_api.cc:935: `ntree_limit` is deprecated, use `iteration_range` instead.
## [15:51:41] WARNING: src/c_api/c_api.cc:935: `ntree_limit` is deprecated, use `iteration_range` instead.
## [15:51:41] WARNING: src/c_api/c_api.cc:935: `ntree_limit` is deprecated, use `iteration_range` instead.
## [15:51:41] WARNING: src/c_api/c_api.cc:935: `ntree_limit` is deprecated, use `iteration_range` instead.
## [15:51:41] WARNING: src/c_api/c_api.cc:935: `ntree_limit` is deprecated, use `iteration_range` instead.
## [15:51:42] WARNING: src/c_api/c_api.cc:935: `ntree_limit` is deprecated, use `iteration_range` instead.
## [15:51:42] WARNING: src/c_api/c_api.cc:935: `ntree_limit` is deprecated, use `iteration_range` instead.
## [15:51:42] WARNING: src/c_api/c_api.cc:935: `ntree_limit` is deprecated, use `iteration_range` instead.
## [15:51:42] WARNING: src/c_api/c_api.cc:935: `ntree_limit` is deprecated, use `iteration_range` instead.
## [15:51:42] WARNING: src/c_api/c_api.cc:935: `ntree_limit` is deprecated, use `iteration_range` instead.
## [15:51:42] WARNING: src/c_api/c_api.cc:935: `ntree_limit` is deprecated, use `iteration_range` instead.
## [15:51:42] WARNING: src/c_api/c_api.cc:935: `ntree_limit` is deprecated, use `iteration_range` instead.
## [15:51:42] WARNING: src/c_api/c_api.cc:935: `ntree_limit` is deprecated, use `iteration_range` instead.
## [15:51:42] WARNING: src/c_api/c_api.cc:935: `ntree_limit` is deprecated, use `iteration_range` instead.
## [15:51:42] WARNING: src/c_api/c_api.cc:935: `ntree_limit` is deprecated, use `iteration_range` instead.
## [15:51:42] WARNING: src/c_api/c_api.cc:935: `ntree_limit` is deprecated, use `iteration_range` instead.
## [15:51:42] WARNING: src/c_api/c_api.cc:935: `ntree_limit` is deprecated, use `iteration_range` instead.
## [15:51:43] WARNING: src/c_api/c_api.cc:935: `ntree_limit` is deprecated, use `iteration_range` instead.
## [15:51:43] WARNING: src/c_api/c_api.cc:935: `ntree_limit` is deprecated, use `iteration_range` instead.
## [15:51:43] WARNING: src/c_api/c_api.cc:935: `ntree_limit` is deprecated, use `iteration_range` instead.
## [15:51:43] WARNING: src/c_api/c_api.cc:935: `ntree_limit` is deprecated, use `iteration_range` instead.
## [15:51:43] WARNING: src/c_api/c_api.cc:935: `ntree_limit` is deprecated, use `iteration_range` instead.
## [15:51:43] WARNING: src/c_api/c_api.cc:935: `ntree_limit` is deprecated, use `iteration_range` instead.
## [15:51:43] WARNING: src/c_api/c_api.cc:935: `ntree_limit` is deprecated, use `iteration_range` instead.
## [15:51:43] WARNING: src/c_api/c_api.cc:935: `ntree_limit` is deprecated, use `iteration_range` instead.
## [15:51:44] WARNING: src/c_api/c_api.cc:935: `ntree_limit` is deprecated, use `iteration_range` instead.
## [15:51:44] WARNING: src/c_api/c_api.cc:935: `ntree_limit` is deprecated, use `iteration_range` instead.
## [15:51:44] WARNING: src/c_api/c_api.cc:935: `ntree_limit` is deprecated, use `iteration_range` instead.
## [15:51:44] WARNING: src/c_api/c_api.cc:935: `ntree_limit` is deprecated, use `iteration_range` instead.
## [15:51:44] WARNING: src/c_api/c_api.cc:935: `ntree_limit` is deprecated, use `iteration_range` instead.
## [15:51:44] WARNING: src/c_api/c_api.cc:935: `ntree_limit` is deprecated, use `iteration_range` instead.
## [15:51:44] WARNING: src/c_api/c_api.cc:935: `ntree_limit` is deprecated, use `iteration_range` instead.
## [15:51:44] WARNING: src/c_api/c_api.cc:935: `ntree_limit` is deprecated, use `iteration_range` instead.
## [15:51:44] WARNING: src/c_api/c_api.cc:935: `ntree_limit` is deprecated, use `iteration_range` instead.
## [15:51:44] WARNING: src/c_api/c_api.cc:935: `ntree_limit` is deprecated, use `iteration_range` instead.
## [15:51:44] WARNING: src/c_api/c_api.cc:935: `ntree_limit` is deprecated, use `iteration_range` instead.
## [15:51:44] WARNING: src/c_api/c_api.cc:935: `ntree_limit` is deprecated, use `iteration_range` instead.
## [15:51:44] WARNING: src/c_api/c_api.cc:935: `ntree_limit` is deprecated, use `iteration_range` instead.
## [15:51:44] WARNING: src/c_api/c_api.cc:935: `ntree_limit` is deprecated, use `iteration_range` instead.
## [15:51:44] WARNING: src/c_api/c_api.cc:935: `ntree_limit` is deprecated, use `iteration_range` instead.
## [15:51:44] WARNING: src/c_api/c_api.cc:935: `ntree_limit` is deprecated, use `iteration_range` instead.
## [15:51:45] WARNING: src/c_api/c_api.cc:935: `ntree_limit` is deprecated, use `iteration_range` instead.
## [15:51:45] WARNING: src/c_api/c_api.cc:935: `ntree_limit` is deprecated, use `iteration_range` instead.
## [15:51:45] WARNING: src/c_api/c_api.cc:935: `ntree_limit` is deprecated, use `iteration_range` instead.
## [15:51:45] WARNING: src/c_api/c_api.cc:935: `ntree_limit` is deprecated, use `iteration_range` instead.
## [15:51:45] WARNING: src/c_api/c_api.cc:935: `ntree_limit` is deprecated, use `iteration_range` instead.
## [15:51:45] WARNING: src/c_api/c_api.cc:935: `ntree_limit` is deprecated, use `iteration_range` instead.
## [15:51:45] WARNING: src/c_api/c_api.cc:935: `ntree_limit` is deprecated, use `iteration_range` instead.
## [15:51:45] WARNING: src/c_api/c_api.cc:935: `ntree_limit` is deprecated, use `iteration_range` instead.
## [15:51:45] WARNING: src/c_api/c_api.cc:935: `ntree_limit` is deprecated, use `iteration_range` instead.
## [15:51:45] WARNING: src/c_api/c_api.cc:935: `ntree_limit` is deprecated, use `iteration_range` instead.
## [15:51:45] WARNING: src/c_api/c_api.cc:935: `ntree_limit` is deprecated, use `iteration_range` instead.
## [15:51:45] WARNING: src/c_api/c_api.cc:935: `ntree_limit` is deprecated, use `iteration_range` instead.
## [15:51:46] WARNING: src/c_api/c_api.cc:935: `ntree_limit` is deprecated, use `iteration_range` instead.
## [15:51:46] WARNING: src/c_api/c_api.cc:935: `ntree_limit` is deprecated, use `iteration_range` instead.
## [15:51:46] WARNING: src/c_api/c_api.cc:935: `ntree_limit` is deprecated, use `iteration_range` instead.
## [15:51:46] WARNING: src/c_api/c_api.cc:935: `ntree_limit` is deprecated, use `iteration_range` instead.
## [15:51:46] WARNING: src/c_api/c_api.cc:935: `ntree_limit` is deprecated, use `iteration_range` instead.
## [15:51:46] WARNING: src/c_api/c_api.cc:935: `ntree_limit` is deprecated, use `iteration_range` instead.
## [15:51:46] WARNING: src/c_api/c_api.cc:935: `ntree_limit` is deprecated, use `iteration_range` instead.
## [15:51:46] WARNING: src/c_api/c_api.cc:935: `ntree_limit` is deprecated, use `iteration_range` instead.
## [15:51:46] WARNING: src/c_api/c_api.cc:935: `ntree_limit` is deprecated, use `iteration_range` instead.
## [15:51:46] WARNING: src/c_api/c_api.cc:935: `ntree_limit` is deprecated, use `iteration_range` instead.
## [15:51:47] WARNING: src/c_api/c_api.cc:935: `ntree_limit` is deprecated, use `iteration_range` instead.
## [15:51:47] WARNING: src/c_api/c_api.cc:935: `ntree_limit` is deprecated, use `iteration_range` instead.
## [15:51:47] WARNING: src/c_api/c_api.cc:935: `ntree_limit` is deprecated, use `iteration_range` instead.
## [15:51:47] WARNING: src/c_api/c_api.cc:935: `ntree_limit` is deprecated, use `iteration_range` instead.
## [15:51:47] WARNING: src/c_api/c_api.cc:935: `ntree_limit` is deprecated, use `iteration_range` instead.
## [15:51:47] WARNING: src/c_api/c_api.cc:935: `ntree_limit` is deprecated, use `iteration_range` instead.
## [15:51:47] WARNING: src/c_api/c_api.cc:935: `ntree_limit` is deprecated, use `iteration_range` instead.
## [15:51:47] WARNING: src/c_api/c_api.cc:935: `ntree_limit` is deprecated, use `iteration_range` instead.
## [15:51:47] WARNING: src/c_api/c_api.cc:935: `ntree_limit` is deprecated, use `iteration_range` instead.
## [15:51:47] WARNING: src/c_api/c_api.cc:935: `ntree_limit` is deprecated, use `iteration_range` instead.
## [15:51:47] WARNING: src/c_api/c_api.cc:935: `ntree_limit` is deprecated, use `iteration_range` instead.
## [15:51:47] WARNING: src/c_api/c_api.cc:935: `ntree_limit` is deprecated, use `iteration_range` instead.
## [15:51:47] WARNING: src/c_api/c_api.cc:935: `ntree_limit` is deprecated, use `iteration_range` instead.
## [15:51:47] WARNING: src/c_api/c_api.cc:935: `ntree_limit` is deprecated, use `iteration_range` instead.
## [15:51:48] WARNING: src/c_api/c_api.cc:935: `ntree_limit` is deprecated, use `iteration_range` instead.
## [15:51:48] WARNING: src/c_api/c_api.cc:935: `ntree_limit` is deprecated, use `iteration_range` instead.
## [15:51:48] WARNING: src/c_api/c_api.cc:935: `ntree_limit` is deprecated, use `iteration_range` instead.
## [15:51:48] WARNING: src/c_api/c_api.cc:935: `ntree_limit` is deprecated, use `iteration_range` instead.
## [15:51:48] WARNING: src/c_api/c_api.cc:935: `ntree_limit` is deprecated, use `iteration_range` instead.
## [15:51:48] WARNING: src/c_api/c_api.cc:935: `ntree_limit` is deprecated, use `iteration_range` instead.
## [15:51:48] WARNING: src/c_api/c_api.cc:935: `ntree_limit` is deprecated, use `iteration_range` instead.
## [15:51:48] WARNING: src/c_api/c_api.cc:935: `ntree_limit` is deprecated, use `iteration_range` instead.
## [15:51:48] WARNING: src/c_api/c_api.cc:935: `ntree_limit` is deprecated, use `iteration_range` instead.
## [15:51:48] WARNING: src/c_api/c_api.cc:935: `ntree_limit` is deprecated, use `iteration_range` instead.
## [15:51:48] WARNING: src/c_api/c_api.cc:935: `ntree_limit` is deprecated, use `iteration_range` instead.
## [15:51:48] WARNING: src/c_api/c_api.cc:935: `ntree_limit` is deprecated, use `iteration_range` instead.
## [15:51:49] WARNING: src/c_api/c_api.cc:935: `ntree_limit` is deprecated, use `iteration_range` instead.
## [15:51:49] WARNING: src/c_api/c_api.cc:935: `ntree_limit` is deprecated, use `iteration_range` instead.
## [15:51:49] WARNING: src/c_api/c_api.cc:935: `ntree_limit` is deprecated, use `iteration_range` instead.
## [15:51:49] WARNING: src/c_api/c_api.cc:935: `ntree_limit` is deprecated, use `iteration_range` instead.
## [15:51:49] WARNING: src/c_api/c_api.cc:935: `ntree_limit` is deprecated, use `iteration_range` instead.
## [15:51:49] WARNING: src/c_api/c_api.cc:935: `ntree_limit` is deprecated, use `iteration_range` instead.
## [15:51:49] WARNING: src/c_api/c_api.cc:935: `ntree_limit` is deprecated, use `iteration_range` instead.
## [15:51:49] WARNING: src/c_api/c_api.cc:935: `ntree_limit` is deprecated, use `iteration_range` instead.
## [15:51:49] WARNING: src/c_api/c_api.cc:935: `ntree_limit` is deprecated, use `iteration_range` instead.
## [15:51:49] WARNING: src/c_api/c_api.cc:935: `ntree_limit` is deprecated, use `iteration_range` instead.
## [15:51:50] WARNING: src/c_api/c_api.cc:935: `ntree_limit` is deprecated, use `iteration_range` instead.
## [15:51:50] WARNING: src/c_api/c_api.cc:935: `ntree_limit` is deprecated, use `iteration_range` instead.
## [15:51:50] WARNING: src/c_api/c_api.cc:935: `ntree_limit` is deprecated, use `iteration_range` instead.
## [15:51:50] WARNING: src/c_api/c_api.cc:935: `ntree_limit` is deprecated, use `iteration_range` instead.
## [15:51:50] WARNING: src/c_api/c_api.cc:935: `ntree_limit` is deprecated, use `iteration_range` instead.
## [15:51:50] WARNING: src/c_api/c_api.cc:935: `ntree_limit` is deprecated, use `iteration_range` instead.
## [15:51:50] WARNING: src/c_api/c_api.cc:935: `ntree_limit` is deprecated, use `iteration_range` instead.
## [15:51:50] WARNING: src/c_api/c_api.cc:935: `ntree_limit` is deprecated, use `iteration_range` instead.
## [15:51:50] WARNING: src/c_api/c_api.cc:935: `ntree_limit` is deprecated, use `iteration_range` instead.
## [15:51:50] WARNING: src/c_api/c_api.cc:935: `ntree_limit` is deprecated, use `iteration_range` instead.
## [15:51:50] WARNING: src/c_api/c_api.cc:935: `ntree_limit` is deprecated, use `iteration_range` instead.
## [15:51:50] WARNING: src/c_api/c_api.cc:935: `ntree_limit` is deprecated, use `iteration_range` instead.
## [15:51:50] WARNING: src/c_api/c_api.cc:935: `ntree_limit` is deprecated, use `iteration_range` instead.
## [15:51:50] WARNING: src/c_api/c_api.cc:935: `ntree_limit` is deprecated, use `iteration_range` instead.
## [15:51:51] WARNING: src/c_api/c_api.cc:935: `ntree_limit` is deprecated, use `iteration_range` instead.
## [15:51:51] WARNING: src/c_api/c_api.cc:935: `ntree_limit` is deprecated, use `iteration_range` instead.
## [15:51:51] WARNING: src/c_api/c_api.cc:935: `ntree_limit` is deprecated, use `iteration_range` instead.
## [15:51:51] WARNING: src/c_api/c_api.cc:935: `ntree_limit` is deprecated, use `iteration_range` instead.
## [15:51:51] WARNING: src/c_api/c_api.cc:935: `ntree_limit` is deprecated, use `iteration_range` instead.
## [15:51:51] WARNING: src/c_api/c_api.cc:935: `ntree_limit` is deprecated, use `iteration_range` instead.
## [15:51:51] WARNING: src/c_api/c_api.cc:935: `ntree_limit` is deprecated, use `iteration_range` instead.
## [15:51:51] WARNING: src/c_api/c_api.cc:935: `ntree_limit` is deprecated, use `iteration_range` instead.
## [15:51:51] WARNING: src/c_api/c_api.cc:935: `ntree_limit` is deprecated, use `iteration_range` instead.
## [15:51:51] WARNING: src/c_api/c_api.cc:935: `ntree_limit` is deprecated, use `iteration_range` instead.
## [15:51:51] WARNING: src/c_api/c_api.cc:935: `ntree_limit` is deprecated, use `iteration_range` instead.
## [15:51:51] WARNING: src/c_api/c_api.cc:935: `ntree_limit` is deprecated, use `iteration_range` instead.
## [15:51:52] WARNING: src/c_api/c_api.cc:935: `ntree_limit` is deprecated, use `iteration_range` instead.
## [15:51:52] WARNING: src/c_api/c_api.cc:935: `ntree_limit` is deprecated, use `iteration_range` instead.
## [15:51:52] WARNING: src/c_api/c_api.cc:935: `ntree_limit` is deprecated, use `iteration_range` instead.
## [15:51:52] WARNING: src/c_api/c_api.cc:935: `ntree_limit` is deprecated, use `iteration_range` instead.
## [15:51:52] WARNING: src/c_api/c_api.cc:935: `ntree_limit` is deprecated, use `iteration_range` instead.
## [15:51:52] WARNING: src/c_api/c_api.cc:935: `ntree_limit` is deprecated, use `iteration_range` instead.
## [15:51:52] WARNING: src/c_api/c_api.cc:935: `ntree_limit` is deprecated, use `iteration_range` instead.
## [15:51:52] WARNING: src/c_api/c_api.cc:935: `ntree_limit` is deprecated, use `iteration_range` instead.
## [15:51:52] WARNING: src/c_api/c_api.cc:935: `ntree_limit` is deprecated, use `iteration_range` instead.
## [15:51:52] WARNING: src/c_api/c_api.cc:935: `ntree_limit` is deprecated, use `iteration_range` instead.
## [15:51:52] WARNING: src/c_api/c_api.cc:935: `ntree_limit` is deprecated, use `iteration_range` instead.
## [15:51:52] WARNING: src/c_api/c_api.cc:935: `ntree_limit` is deprecated, use `iteration_range` instead.
## [15:51:53] WARNING: src/c_api/c_api.cc:935: `ntree_limit` is deprecated, use `iteration_range` instead.
## [15:51:53] WARNING: src/c_api/c_api.cc:935: `ntree_limit` is deprecated, use `iteration_range` instead.
## [15:51:53] WARNING: src/c_api/c_api.cc:935: `ntree_limit` is deprecated, use `iteration_range` instead.
## [15:51:53] WARNING: src/c_api/c_api.cc:935: `ntree_limit` is deprecated, use `iteration_range` instead.
## [15:51:53] WARNING: src/c_api/c_api.cc:935: `ntree_limit` is deprecated, use `iteration_range` instead.
## [15:51:53] WARNING: src/c_api/c_api.cc:935: `ntree_limit` is deprecated, use `iteration_range` instead.
## [15:51:53] WARNING: src/c_api/c_api.cc:935: `ntree_limit` is deprecated, use `iteration_range` instead.
## [15:51:53] WARNING: src/c_api/c_api.cc:935: `ntree_limit` is deprecated, use `iteration_range` instead.
## [15:51:53] WARNING: src/c_api/c_api.cc:935: `ntree_limit` is deprecated, use `iteration_range` instead.
## [15:51:53] WARNING: src/c_api/c_api.cc:935: `ntree_limit` is deprecated, use `iteration_range` instead.
## [15:51:53] WARNING: src/c_api/c_api.cc:935: `ntree_limit` is deprecated, use `iteration_range` instead.
## [15:51:53] WARNING: src/c_api/c_api.cc:935: `ntree_limit` is deprecated, use `iteration_range` instead.
## [15:51:53] WARNING: src/c_api/c_api.cc:935: `ntree_limit` is deprecated, use `iteration_range` instead.
## [15:51:53] WARNING: src/c_api/c_api.cc:935: `ntree_limit` is deprecated, use `iteration_range` instead.
## [15:51:54] WARNING: src/c_api/c_api.cc:935: `ntree_limit` is deprecated, use `iteration_range` instead.
## [15:51:54] WARNING: src/c_api/c_api.cc:935: `ntree_limit` is deprecated, use `iteration_range` instead.
## [15:51:54] WARNING: src/c_api/c_api.cc:935: `ntree_limit` is deprecated, use `iteration_range` instead.
## [15:51:54] WARNING: src/c_api/c_api.cc:935: `ntree_limit` is deprecated, use `iteration_range` instead.
## [15:51:54] WARNING: src/c_api/c_api.cc:935: `ntree_limit` is deprecated, use `iteration_range` instead.
## [15:51:54] WARNING: src/c_api/c_api.cc:935: `ntree_limit` is deprecated, use `iteration_range` instead.
## [15:51:54] WARNING: src/c_api/c_api.cc:935: `ntree_limit` is deprecated, use `iteration_range` instead.
## [15:51:54] WARNING: src/c_api/c_api.cc:935: `ntree_limit` is deprecated, use `iteration_range` instead.
## [15:51:54] WARNING: src/c_api/c_api.cc:935: `ntree_limit` is deprecated, use `iteration_range` instead.
## [15:51:54] WARNING: src/c_api/c_api.cc:935: `ntree_limit` is deprecated, use `iteration_range` instead.
## [15:51:54] WARNING: src/c_api/c_api.cc:935: `ntree_limit` is deprecated, use `iteration_range` instead.
## [15:51:54] WARNING: src/c_api/c_api.cc:935: `ntree_limit` is deprecated, use `iteration_range` instead.
## [15:51:55] WARNING: src/c_api/c_api.cc:935: `ntree_limit` is deprecated, use `iteration_range` instead.
## [15:51:55] WARNING: src/c_api/c_api.cc:935: `ntree_limit` is deprecated, use `iteration_range` instead.
## [15:51:55] WARNING: src/c_api/c_api.cc:935: `ntree_limit` is deprecated, use `iteration_range` instead.
## [15:51:55] WARNING: src/c_api/c_api.cc:935: `ntree_limit` is deprecated, use `iteration_range` instead.
## [15:51:55] WARNING: src/c_api/c_api.cc:935: `ntree_limit` is deprecated, use `iteration_range` instead.
## [15:51:55] WARNING: src/c_api/c_api.cc:935: `ntree_limit` is deprecated, use `iteration_range` instead.
## [15:51:55] WARNING: src/c_api/c_api.cc:935: `ntree_limit` is deprecated, use `iteration_range` instead.
## [15:51:55] WARNING: src/c_api/c_api.cc:935: `ntree_limit` is deprecated, use `iteration_range` instead.
## [15:51:55] WARNING: src/c_api/c_api.cc:935: `ntree_limit` is deprecated, use `iteration_range` instead.
## [15:51:55] WARNING: src/c_api/c_api.cc:935: `ntree_limit` is deprecated, use `iteration_range` instead.
## [15:51:56] WARNING: src/c_api/c_api.cc:935: `ntree_limit` is deprecated, use `iteration_range` instead.
## [15:51:56] WARNING: src/c_api/c_api.cc:935: `ntree_limit` is deprecated, use `iteration_range` instead.
## [15:51:56] WARNING: src/c_api/c_api.cc:935: `ntree_limit` is deprecated, use `iteration_range` instead.
## [15:51:56] WARNING: src/c_api/c_api.cc:935: `ntree_limit` is deprecated, use `iteration_range` instead.
## [15:51:56] WARNING: src/c_api/c_api.cc:935: `ntree_limit` is deprecated, use `iteration_range` instead.
## [15:51:56] WARNING: src/c_api/c_api.cc:935: `ntree_limit` is deprecated, use `iteration_range` instead.
## [15:51:56] WARNING: src/c_api/c_api.cc:935: `ntree_limit` is deprecated, use `iteration_range` instead.
## [15:51:56] WARNING: src/c_api/c_api.cc:935: `ntree_limit` is deprecated, use `iteration_range` instead.
## [15:51:56] WARNING: src/c_api/c_api.cc:935: `ntree_limit` is deprecated, use `iteration_range` instead.
## [15:51:56] WARNING: src/c_api/c_api.cc:935: `ntree_limit` is deprecated, use `iteration_range` instead.
## [15:51:56] WARNING: src/c_api/c_api.cc:935: `ntree_limit` is deprecated, use `iteration_range` instead.
## [15:51:56] WARNING: src/c_api/c_api.cc:935: `ntree_limit` is deprecated, use `iteration_range` instead.
## [15:51:56] WARNING: src/c_api/c_api.cc:935: `ntree_limit` is deprecated, use `iteration_range` instead.
## [15:51:56] WARNING: src/c_api/c_api.cc:935: `ntree_limit` is deprecated, use `iteration_range` instead.
## [15:51:57] WARNING: src/c_api/c_api.cc:935: `ntree_limit` is deprecated, use `iteration_range` instead.
## [15:51:57] WARNING: src/c_api/c_api.cc:935: `ntree_limit` is deprecated, use `iteration_range` instead.
## [15:51:57] WARNING: src/c_api/c_api.cc:935: `ntree_limit` is deprecated, use `iteration_range` instead.
## [15:51:57] WARNING: src/c_api/c_api.cc:935: `ntree_limit` is deprecated, use `iteration_range` instead.
## [15:51:57] WARNING: src/c_api/c_api.cc:935: `ntree_limit` is deprecated, use `iteration_range` instead.
## [15:51:57] WARNING: src/c_api/c_api.cc:935: `ntree_limit` is deprecated, use `iteration_range` instead.
## [15:51:57] WARNING: src/c_api/c_api.cc:935: `ntree_limit` is deprecated, use `iteration_range` instead.
## [15:51:57] WARNING: src/c_api/c_api.cc:935: `ntree_limit` is deprecated, use `iteration_range` instead.
## [15:51:57] WARNING: src/c_api/c_api.cc:935: `ntree_limit` is deprecated, use `iteration_range` instead.
## [15:51:57] WARNING: src/c_api/c_api.cc:935: `ntree_limit` is deprecated, use `iteration_range` instead.
## [15:51:57] WARNING: src/c_api/c_api.cc:935: `ntree_limit` is deprecated, use `iteration_range` instead.
## [15:51:57] WARNING: src/c_api/c_api.cc:935: `ntree_limit` is deprecated, use `iteration_range` instead.
## [15:51:58] WARNING: src/c_api/c_api.cc:935: `ntree_limit` is deprecated, use `iteration_range` instead.
## [15:51:58] WARNING: src/c_api/c_api.cc:935: `ntree_limit` is deprecated, use `iteration_range` instead.
## [15:51:58] WARNING: src/c_api/c_api.cc:935: `ntree_limit` is deprecated, use `iteration_range` instead.
## [15:51:58] WARNING: src/c_api/c_api.cc:935: `ntree_limit` is deprecated, use `iteration_range` instead.
## [15:51:58] WARNING: src/c_api/c_api.cc:935: `ntree_limit` is deprecated, use `iteration_range` instead.
## [15:51:58] WARNING: src/c_api/c_api.cc:935: `ntree_limit` is deprecated, use `iteration_range` instead.
## [15:51:58] WARNING: src/c_api/c_api.cc:935: `ntree_limit` is deprecated, use `iteration_range` instead.
## [15:51:58] WARNING: src/c_api/c_api.cc:935: `ntree_limit` is deprecated, use `iteration_range` instead.
## [15:51:58] WARNING: src/c_api/c_api.cc:935: `ntree_limit` is deprecated, use `iteration_range` instead.
## [15:51:58] WARNING: src/c_api/c_api.cc:935: `ntree_limit` is deprecated, use `iteration_range` instead.
## [15:51:58] WARNING: src/c_api/c_api.cc:935: `ntree_limit` is deprecated, use `iteration_range` instead.
## [15:51:58] WARNING: src/c_api/c_api.cc:935: `ntree_limit` is deprecated, use `iteration_range` instead.
## [15:51:59] WARNING: src/c_api/c_api.cc:935: `ntree_limit` is deprecated, use `iteration_range` instead.
## [15:51:59] WARNING: src/c_api/c_api.cc:935: `ntree_limit` is deprecated, use `iteration_range` instead.
## [15:51:59] WARNING: src/c_api/c_api.cc:935: `ntree_limit` is deprecated, use `iteration_range` instead.
## [15:51:59] WARNING: src/c_api/c_api.cc:935: `ntree_limit` is deprecated, use `iteration_range` instead.
## [15:51:59] WARNING: src/c_api/c_api.cc:935: `ntree_limit` is deprecated, use `iteration_range` instead.
## [15:51:59] WARNING: src/c_api/c_api.cc:935: `ntree_limit` is deprecated, use `iteration_range` instead.
## [15:51:59] WARNING: src/c_api/c_api.cc:935: `ntree_limit` is deprecated, use `iteration_range` instead.
## [15:51:59] WARNING: src/c_api/c_api.cc:935: `ntree_limit` is deprecated, use `iteration_range` instead.
## [15:51:59] WARNING: src/c_api/c_api.cc:935: `ntree_limit` is deprecated, use `iteration_range` instead.
## [15:51:59] WARNING: src/c_api/c_api.cc:935: `ntree_limit` is deprecated, use `iteration_range` instead.
## [15:51:59] WARNING: src/c_api/c_api.cc:935: `ntree_limit` is deprecated, use `iteration_range` instead.
## [15:51:59] WARNING: src/c_api/c_api.cc:935: `ntree_limit` is deprecated, use `iteration_range` instead.
## [15:51:59] WARNING: src/c_api/c_api.cc:935: `ntree_limit` is deprecated, use `iteration_range` instead.
## [15:51:59] WARNING: src/c_api/c_api.cc:935: `ntree_limit` is deprecated, use `iteration_range` instead.
## [15:52:00] WARNING: src/c_api/c_api.cc:935: `ntree_limit` is deprecated, use `iteration_range` instead.
## [15:52:00] WARNING: src/c_api/c_api.cc:935: `ntree_limit` is deprecated, use `iteration_range` instead.
## [15:52:00] WARNING: src/c_api/c_api.cc:935: `ntree_limit` is deprecated, use `iteration_range` instead.
## [15:52:00] WARNING: src/c_api/c_api.cc:935: `ntree_limit` is deprecated, use `iteration_range` instead.
## [15:52:00] WARNING: src/c_api/c_api.cc:935: `ntree_limit` is deprecated, use `iteration_range` instead.
## [15:52:00] WARNING: src/c_api/c_api.cc:935: `ntree_limit` is deprecated, use `iteration_range` instead.
## [15:52:00] WARNING: src/c_api/c_api.cc:935: `ntree_limit` is deprecated, use `iteration_range` instead.
## [15:52:00] WARNING: src/c_api/c_api.cc:935: `ntree_limit` is deprecated, use `iteration_range` instead.
## [15:52:00] WARNING: src/c_api/c_api.cc:935: `ntree_limit` is deprecated, use `iteration_range` instead.
## [15:52:00] WARNING: src/c_api/c_api.cc:935: `ntree_limit` is deprecated, use `iteration_range` instead.
## [15:52:01] WARNING: src/c_api/c_api.cc:935: `ntree_limit` is deprecated, use `iteration_range` instead.
## [15:52:01] WARNING: src/c_api/c_api.cc:935: `ntree_limit` is deprecated, use `iteration_range` instead.
## [15:52:01] WARNING: src/c_api/c_api.cc:935: `ntree_limit` is deprecated, use `iteration_range` instead.
## [15:52:01] WARNING: src/c_api/c_api.cc:935: `ntree_limit` is deprecated, use `iteration_range` instead.
## [15:52:01] WARNING: src/c_api/c_api.cc:935: `ntree_limit` is deprecated, use `iteration_range` instead.
## [15:52:01] WARNING: src/c_api/c_api.cc:935: `ntree_limit` is deprecated, use `iteration_range` instead.
## [15:52:01] WARNING: src/c_api/c_api.cc:935: `ntree_limit` is deprecated, use `iteration_range` instead.
## [15:52:01] WARNING: src/c_api/c_api.cc:935: `ntree_limit` is deprecated, use `iteration_range` instead.
## [15:52:01] WARNING: src/c_api/c_api.cc:935: `ntree_limit` is deprecated, use `iteration_range` instead.
## [15:52:01] WARNING: src/c_api/c_api.cc:935: `ntree_limit` is deprecated, use `iteration_range` instead.
## [15:52:01] WARNING: src/c_api/c_api.cc:935: `ntree_limit` is deprecated, use `iteration_range` instead.
## [15:52:01] WARNING: src/c_api/c_api.cc:935: `ntree_limit` is deprecated, use `iteration_range` instead.
## [15:52:02] WARNING: src/c_api/c_api.cc:935: `ntree_limit` is deprecated, use `iteration_range` instead.
## [15:52:02] WARNING: src/c_api/c_api.cc:935: `ntree_limit` is deprecated, use `iteration_range` instead.
## [15:52:02] WARNING: src/c_api/c_api.cc:935: `ntree_limit` is deprecated, use `iteration_range` instead.
## [15:52:02] WARNING: src/c_api/c_api.cc:935: `ntree_limit` is deprecated, use `iteration_range` instead.
## [15:52:02] WARNING: src/c_api/c_api.cc:935: `ntree_limit` is deprecated, use `iteration_range` instead.
## [15:52:02] WARNING: src/c_api/c_api.cc:935: `ntree_limit` is deprecated, use `iteration_range` instead.
## [15:52:02] WARNING: src/c_api/c_api.cc:935: `ntree_limit` is deprecated, use `iteration_range` instead.
## [15:52:02] WARNING: src/c_api/c_api.cc:935: `ntree_limit` is deprecated, use `iteration_range` instead.
## [15:52:02] WARNING: src/c_api/c_api.cc:935: `ntree_limit` is deprecated, use `iteration_range` instead.
## [15:52:02] WARNING: src/c_api/c_api.cc:935: `ntree_limit` is deprecated, use `iteration_range` instead.
## [15:52:02] WARNING: src/c_api/c_api.cc:935: `ntree_limit` is deprecated, use `iteration_range` instead.
## [15:52:02] WARNING: src/c_api/c_api.cc:935: `ntree_limit` is deprecated, use `iteration_range` instead.
## [15:52:02] WARNING: src/c_api/c_api.cc:935: `ntree_limit` is deprecated, use `iteration_range` instead.
## [15:52:02] WARNING: src/c_api/c_api.cc:935: `ntree_limit` is deprecated, use `iteration_range` instead.
## [15:52:03] WARNING: src/c_api/c_api.cc:935: `ntree_limit` is deprecated, use `iteration_range` instead.
## [15:52:03] WARNING: src/c_api/c_api.cc:935: `ntree_limit` is deprecated, use `iteration_range` instead.
## [15:52:03] WARNING: src/c_api/c_api.cc:935: `ntree_limit` is deprecated, use `iteration_range` instead.
## [15:52:03] WARNING: src/c_api/c_api.cc:935: `ntree_limit` is deprecated, use `iteration_range` instead.
## [15:52:03] WARNING: src/c_api/c_api.cc:935: `ntree_limit` is deprecated, use `iteration_range` instead.
## [15:52:03] WARNING: src/c_api/c_api.cc:935: `ntree_limit` is deprecated, use `iteration_range` instead.
## [15:52:03] WARNING: src/c_api/c_api.cc:935: `ntree_limit` is deprecated, use `iteration_range` instead.
## [15:52:03] WARNING: src/c_api/c_api.cc:935: `ntree_limit` is deprecated, use `iteration_range` instead.
## [15:52:03] WARNING: src/c_api/c_api.cc:935: `ntree_limit` is deprecated, use `iteration_range` instead.
## [15:52:03] WARNING: src/c_api/c_api.cc:935: `ntree_limit` is deprecated, use `iteration_range` instead.
## [15:52:04] WARNING: src/c_api/c_api.cc:935: `ntree_limit` is deprecated, use `iteration_range` instead.
## [15:52:04] WARNING: src/c_api/c_api.cc:935: `ntree_limit` is deprecated, use `iteration_range` instead.
## [15:52:04] WARNING: src/c_api/c_api.cc:935: `ntree_limit` is deprecated, use `iteration_range` instead.
## [15:52:04] WARNING: src/c_api/c_api.cc:935: `ntree_limit` is deprecated, use `iteration_range` instead.
## [15:52:04] WARNING: src/c_api/c_api.cc:935: `ntree_limit` is deprecated, use `iteration_range` instead.
## [15:52:04] WARNING: src/c_api/c_api.cc:935: `ntree_limit` is deprecated, use `iteration_range` instead.
## [15:52:04] WARNING: src/c_api/c_api.cc:935: `ntree_limit` is deprecated, use `iteration_range` instead.
## [15:52:04] WARNING: src/c_api/c_api.cc:935: `ntree_limit` is deprecated, use `iteration_range` instead.
## [15:52:04] WARNING: src/c_api/c_api.cc:935: `ntree_limit` is deprecated, use `iteration_range` instead.
## [15:52:04] WARNING: src/c_api/c_api.cc:935: `ntree_limit` is deprecated, use `iteration_range` instead.
## [15:52:07] WARNING: src/c_api/c_api.cc:935: `ntree_limit` is deprecated, use `iteration_range` instead.
## [15:52:07] WARNING: src/c_api/c_api.cc:935: `ntree_limit` is deprecated, use `iteration_range` instead.
## [15:52:07] WARNING: src/c_api/c_api.cc:935: `ntree_limit` is deprecated, use `iteration_range` instead.
## [15:52:07] WARNING: src/c_api/c_api.cc:935: `ntree_limit` is deprecated, use `iteration_range` instead.
## [15:52:08] WARNING: src/c_api/c_api.cc:935: `ntree_limit` is deprecated, use `iteration_range` instead.
## [15:52:08] WARNING: src/c_api/c_api.cc:935: `ntree_limit` is deprecated, use `iteration_range` instead.
## [15:52:08] WARNING: src/c_api/c_api.cc:935: `ntree_limit` is deprecated, use `iteration_range` instead.
## [15:52:08] WARNING: src/c_api/c_api.cc:935: `ntree_limit` is deprecated, use `iteration_range` instead.
## [15:52:08] WARNING: src/c_api/c_api.cc:935: `ntree_limit` is deprecated, use `iteration_range` instead.
## [15:52:08] WARNING: src/c_api/c_api.cc:935: `ntree_limit` is deprecated, use `iteration_range` instead.
## [15:52:08] WARNING: src/c_api/c_api.cc:935: `ntree_limit` is deprecated, use `iteration_range` instead.
## [15:52:08] WARNING: src/c_api/c_api.cc:935: `ntree_limit` is deprecated, use `iteration_range` instead.
## [15:52:08] WARNING: src/c_api/c_api.cc:935: `ntree_limit` is deprecated, use `iteration_range` instead.
## [15:52:08] WARNING: src/c_api/c_api.cc:935: `ntree_limit` is deprecated, use `iteration_range` instead.
## [15:52:08] WARNING: src/c_api/c_api.cc:935: `ntree_limit` is deprecated, use `iteration_range` instead.
## [15:52:08] WARNING: src/c_api/c_api.cc:935: `ntree_limit` is deprecated, use `iteration_range` instead.
## [15:52:09] WARNING: src/c_api/c_api.cc:935: `ntree_limit` is deprecated, use `iteration_range` instead.
## [15:52:09] WARNING: src/c_api/c_api.cc:935: `ntree_limit` is deprecated, use `iteration_range` instead.
## [15:52:09] WARNING: src/c_api/c_api.cc:935: `ntree_limit` is deprecated, use `iteration_range` instead.
## [15:52:09] WARNING: src/c_api/c_api.cc:935: `ntree_limit` is deprecated, use `iteration_range` instead.
## [15:52:09] WARNING: src/c_api/c_api.cc:935: `ntree_limit` is deprecated, use `iteration_range` instead.
## [15:52:09] WARNING: src/c_api/c_api.cc:935: `ntree_limit` is deprecated, use `iteration_range` instead.
## [15:52:09] WARNING: src/c_api/c_api.cc:935: `ntree_limit` is deprecated, use `iteration_range` instead.
## [15:52:09] WARNING: src/c_api/c_api.cc:935: `ntree_limit` is deprecated, use `iteration_range` instead.
## [15:52:09] WARNING: src/c_api/c_api.cc:935: `ntree_limit` is deprecated, use `iteration_range` instead.
## [15:52:09] WARNING: src/c_api/c_api.cc:935: `ntree_limit` is deprecated, use `iteration_range` instead.
## [15:52:10] WARNING: src/c_api/c_api.cc:935: `ntree_limit` is deprecated, use `iteration_range` instead.
## [15:52:10] WARNING: src/c_api/c_api.cc:935: `ntree_limit` is deprecated, use `iteration_range` instead.
## [15:52:10] WARNING: src/c_api/c_api.cc:935: `ntree_limit` is deprecated, use `iteration_range` instead.
## [15:52:10] WARNING: src/c_api/c_api.cc:935: `ntree_limit` is deprecated, use `iteration_range` instead.
## [15:52:10] WARNING: src/c_api/c_api.cc:935: `ntree_limit` is deprecated, use `iteration_range` instead.
## [15:52:10] WARNING: src/c_api/c_api.cc:935: `ntree_limit` is deprecated, use `iteration_range` instead.
## [15:52:10] WARNING: src/c_api/c_api.cc:935: `ntree_limit` is deprecated, use `iteration_range` instead.
## [15:52:10] WARNING: src/c_api/c_api.cc:935: `ntree_limit` is deprecated, use `iteration_range` instead.
## [15:52:10] WARNING: src/c_api/c_api.cc:935: `ntree_limit` is deprecated, use `iteration_range` instead.
## [15:52:10] WARNING: src/c_api/c_api.cc:935: `ntree_limit` is deprecated, use `iteration_range` instead.
## [15:52:11] WARNING: src/c_api/c_api.cc:935: `ntree_limit` is deprecated, use `iteration_range` instead.
## [15:52:11] WARNING: src/c_api/c_api.cc:935: `ntree_limit` is deprecated, use `iteration_range` instead.
## [15:52:11] WARNING: src/c_api/c_api.cc:935: `ntree_limit` is deprecated, use `iteration_range` instead.
## [15:52:11] WARNING: src/c_api/c_api.cc:935: `ntree_limit` is deprecated, use `iteration_range` instead.
## [15:52:11] WARNING: src/c_api/c_api.cc:935: `ntree_limit` is deprecated, use `iteration_range` instead.
## [15:52:11] WARNING: src/c_api/c_api.cc:935: `ntree_limit` is deprecated, use `iteration_range` instead.
## [15:52:11] WARNING: src/c_api/c_api.cc:935: `ntree_limit` is deprecated, use `iteration_range` instead.
## [15:52:11] WARNING: src/c_api/c_api.cc:935: `ntree_limit` is deprecated, use `iteration_range` instead.
## [15:52:11] WARNING: src/c_api/c_api.cc:935: `ntree_limit` is deprecated, use `iteration_range` instead.
## [15:52:11] WARNING: src/c_api/c_api.cc:935: `ntree_limit` is deprecated, use `iteration_range` instead.
## [15:52:11] WARNING: src/c_api/c_api.cc:935: `ntree_limit` is deprecated, use `iteration_range` instead.
## [15:52:11] WARNING: src/c_api/c_api.cc:935: `ntree_limit` is deprecated, use `iteration_range` instead.
## [15:52:11] WARNING: src/c_api/c_api.cc:935: `ntree_limit` is deprecated, use `iteration_range` instead.
## [15:52:11] WARNING: src/c_api/c_api.cc:935: `ntree_limit` is deprecated, use `iteration_range` instead.
## [15:52:12] WARNING: src/c_api/c_api.cc:935: `ntree_limit` is deprecated, use `iteration_range` instead.
## [15:52:12] WARNING: src/c_api/c_api.cc:935: `ntree_limit` is deprecated, use `iteration_range` instead.
## [15:52:12] WARNING: src/c_api/c_api.cc:935: `ntree_limit` is deprecated, use `iteration_range` instead.
## [15:52:12] WARNING: src/c_api/c_api.cc:935: `ntree_limit` is deprecated, use `iteration_range` instead.
## [15:52:12] WARNING: src/c_api/c_api.cc:935: `ntree_limit` is deprecated, use `iteration_range` instead.
## [15:52:12] WARNING: src/c_api/c_api.cc:935: `ntree_limit` is deprecated, use `iteration_range` instead.
## [15:52:12] WARNING: src/c_api/c_api.cc:935: `ntree_limit` is deprecated, use `iteration_range` instead.
## [15:52:12] WARNING: src/c_api/c_api.cc:935: `ntree_limit` is deprecated, use `iteration_range` instead.
## [15:52:12] WARNING: src/c_api/c_api.cc:935: `ntree_limit` is deprecated, use `iteration_range` instead.
## [15:52:12] WARNING: src/c_api/c_api.cc:935: `ntree_limit` is deprecated, use `iteration_range` instead.
## [15:52:12] WARNING: src/c_api/c_api.cc:935: `ntree_limit` is deprecated, use `iteration_range` instead.
## [15:52:12] WARNING: src/c_api/c_api.cc:935: `ntree_limit` is deprecated, use `iteration_range` instead.
## [15:52:13] WARNING: src/c_api/c_api.cc:935: `ntree_limit` is deprecated, use `iteration_range` instead.
## [15:52:13] WARNING: src/c_api/c_api.cc:935: `ntree_limit` is deprecated, use `iteration_range` instead.
## [15:52:13] WARNING: src/c_api/c_api.cc:935: `ntree_limit` is deprecated, use `iteration_range` instead.
## [15:52:13] WARNING: src/c_api/c_api.cc:935: `ntree_limit` is deprecated, use `iteration_range` instead.
## [15:52:13] WARNING: src/c_api/c_api.cc:935: `ntree_limit` is deprecated, use `iteration_range` instead.
## [15:52:13] WARNING: src/c_api/c_api.cc:935: `ntree_limit` is deprecated, use `iteration_range` instead.
## [15:52:13] WARNING: src/c_api/c_api.cc:935: `ntree_limit` is deprecated, use `iteration_range` instead.
## [15:52:13] WARNING: src/c_api/c_api.cc:935: `ntree_limit` is deprecated, use `iteration_range` instead.
## [15:52:13] WARNING: src/c_api/c_api.cc:935: `ntree_limit` is deprecated, use `iteration_range` instead.
## [15:52:13] WARNING: src/c_api/c_api.cc:935: `ntree_limit` is deprecated, use `iteration_range` instead.
## [15:52:14] WARNING: src/c_api/c_api.cc:935: `ntree_limit` is deprecated, use `iteration_range` instead.
## [15:52:14] WARNING: src/c_api/c_api.cc:935: `ntree_limit` is deprecated, use `iteration_range` instead.
## [15:52:14] WARNING: src/c_api/c_api.cc:935: `ntree_limit` is deprecated, use `iteration_range` instead.
## [15:52:14] WARNING: src/c_api/c_api.cc:935: `ntree_limit` is deprecated, use `iteration_range` instead.
## [15:52:14] WARNING: src/c_api/c_api.cc:935: `ntree_limit` is deprecated, use `iteration_range` instead.
## [15:52:14] WARNING: src/c_api/c_api.cc:935: `ntree_limit` is deprecated, use `iteration_range` instead.
## [15:52:14] WARNING: src/c_api/c_api.cc:935: `ntree_limit` is deprecated, use `iteration_range` instead.
## [15:52:14] WARNING: src/c_api/c_api.cc:935: `ntree_limit` is deprecated, use `iteration_range` instead.
## [15:52:14] WARNING: src/c_api/c_api.cc:935: `ntree_limit` is deprecated, use `iteration_range` instead.
## [15:52:14] WARNING: src/c_api/c_api.cc:935: `ntree_limit` is deprecated, use `iteration_range` instead.
## [15:52:14] WARNING: src/c_api/c_api.cc:935: `ntree_limit` is deprecated, use `iteration_range` instead.
## [15:52:14] WARNING: src/c_api/c_api.cc:935: `ntree_limit` is deprecated, use `iteration_range` instead.
## [15:52:14] WARNING: src/c_api/c_api.cc:935: `ntree_limit` is deprecated, use `iteration_range` instead.
## [15:52:14] WARNING: src/c_api/c_api.cc:935: `ntree_limit` is deprecated, use `iteration_range` instead.
## [15:52:15] WARNING: src/c_api/c_api.cc:935: `ntree_limit` is deprecated, use `iteration_range` instead.
## [15:52:15] WARNING: src/c_api/c_api.cc:935: `ntree_limit` is deprecated, use `iteration_range` instead.
## [15:52:15] WARNING: src/c_api/c_api.cc:935: `ntree_limit` is deprecated, use `iteration_range` instead.
## [15:52:15] WARNING: src/c_api/c_api.cc:935: `ntree_limit` is deprecated, use `iteration_range` instead.
## [15:52:15] WARNING: src/c_api/c_api.cc:935: `ntree_limit` is deprecated, use `iteration_range` instead.
## [15:52:15] WARNING: src/c_api/c_api.cc:935: `ntree_limit` is deprecated, use `iteration_range` instead.
## [15:52:15] WARNING: src/c_api/c_api.cc:935: `ntree_limit` is deprecated, use `iteration_range` instead.
## [15:52:15] WARNING: src/c_api/c_api.cc:935: `ntree_limit` is deprecated, use `iteration_range` instead.
## [15:52:15] WARNING: src/c_api/c_api.cc:935: `ntree_limit` is deprecated, use `iteration_range` instead.
## [15:52:15] WARNING: src/c_api/c_api.cc:935: `ntree_limit` is deprecated, use `iteration_range` instead.
## [15:52:16] WARNING: src/c_api/c_api.cc:935: `ntree_limit` is deprecated, use `iteration_range` instead.
## [15:52:16] WARNING: src/c_api/c_api.cc:935: `ntree_limit` is deprecated, use `iteration_range` instead.
## [15:52:16] WARNING: src/c_api/c_api.cc:935: `ntree_limit` is deprecated, use `iteration_range` instead.
## [15:52:16] WARNING: src/c_api/c_api.cc:935: `ntree_limit` is deprecated, use `iteration_range` instead.
## [15:52:16] WARNING: src/c_api/c_api.cc:935: `ntree_limit` is deprecated, use `iteration_range` instead.
## [15:52:16] WARNING: src/c_api/c_api.cc:935: `ntree_limit` is deprecated, use `iteration_range` instead.
## [15:52:16] WARNING: src/c_api/c_api.cc:935: `ntree_limit` is deprecated, use `iteration_range` instead.
## [15:52:16] WARNING: src/c_api/c_api.cc:935: `ntree_limit` is deprecated, use `iteration_range` instead.
## [15:52:17] WARNING: src/c_api/c_api.cc:935: `ntree_limit` is deprecated, use `iteration_range` instead.
## [15:52:17] WARNING: src/c_api/c_api.cc:935: `ntree_limit` is deprecated, use `iteration_range` instead.
## [15:52:17] WARNING: src/c_api/c_api.cc:935: `ntree_limit` is deprecated, use `iteration_range` instead.
## [15:52:17] WARNING: src/c_api/c_api.cc:935: `ntree_limit` is deprecated, use `iteration_range` instead.
## [15:52:17] WARNING: src/c_api/c_api.cc:935: `ntree_limit` is deprecated, use `iteration_range` instead.
## [15:52:17] WARNING: src/c_api/c_api.cc:935: `ntree_limit` is deprecated, use `iteration_range` instead.
## [15:52:17] WARNING: src/c_api/c_api.cc:935: `ntree_limit` is deprecated, use `iteration_range` instead.
## [15:52:17] WARNING: src/c_api/c_api.cc:935: `ntree_limit` is deprecated, use `iteration_range` instead.
## [15:52:17] WARNING: src/c_api/c_api.cc:935: `ntree_limit` is deprecated, use `iteration_range` instead.
## [15:52:17] WARNING: src/c_api/c_api.cc:935: `ntree_limit` is deprecated, use `iteration_range` instead.
## [15:52:17] WARNING: src/c_api/c_api.cc:935: `ntree_limit` is deprecated, use `iteration_range` instead.
## [15:52:17] WARNING: src/c_api/c_api.cc:935: `ntree_limit` is deprecated, use `iteration_range` instead.
## [15:52:17] WARNING: src/c_api/c_api.cc:935: `ntree_limit` is deprecated, use `iteration_range` instead.
## [15:52:17] WARNING: src/c_api/c_api.cc:935: `ntree_limit` is deprecated, use `iteration_range` instead.
## [15:52:18] WARNING: src/c_api/c_api.cc:935: `ntree_limit` is deprecated, use `iteration_range` instead.
## [15:52:18] WARNING: src/c_api/c_api.cc:935: `ntree_limit` is deprecated, use `iteration_range` instead.
## [15:52:18] WARNING: src/c_api/c_api.cc:935: `ntree_limit` is deprecated, use `iteration_range` instead.
## [15:52:18] WARNING: src/c_api/c_api.cc:935: `ntree_limit` is deprecated, use `iteration_range` instead.
## [15:52:18] WARNING: src/c_api/c_api.cc:935: `ntree_limit` is deprecated, use `iteration_range` instead.
## [15:52:18] WARNING: src/c_api/c_api.cc:935: `ntree_limit` is deprecated, use `iteration_range` instead.
## [15:52:18] WARNING: src/c_api/c_api.cc:935: `ntree_limit` is deprecated, use `iteration_range` instead.
## [15:52:18] WARNING: src/c_api/c_api.cc:935: `ntree_limit` is deprecated, use `iteration_range` instead.
## [15:52:18] WARNING: src/c_api/c_api.cc:935: `ntree_limit` is deprecated, use `iteration_range` instead.
## [15:52:18] WARNING: src/c_api/c_api.cc:935: `ntree_limit` is deprecated, use `iteration_range` instead.
## [15:52:18] WARNING: src/c_api/c_api.cc:935: `ntree_limit` is deprecated, use `iteration_range` instead.
## [15:52:18] WARNING: src/c_api/c_api.cc:935: `ntree_limit` is deprecated, use `iteration_range` instead.
## [15:52:19] WARNING: src/c_api/c_api.cc:935: `ntree_limit` is deprecated, use `iteration_range` instead.
## [15:52:19] WARNING: src/c_api/c_api.cc:935: `ntree_limit` is deprecated, use `iteration_range` instead.
## [15:52:19] WARNING: src/c_api/c_api.cc:935: `ntree_limit` is deprecated, use `iteration_range` instead.
## [15:52:19] WARNING: src/c_api/c_api.cc:935: `ntree_limit` is deprecated, use `iteration_range` instead.
## [15:52:19] WARNING: src/c_api/c_api.cc:935: `ntree_limit` is deprecated, use `iteration_range` instead.
## [15:52:19] WARNING: src/c_api/c_api.cc:935: `ntree_limit` is deprecated, use `iteration_range` instead.
## [15:52:19] WARNING: src/c_api/c_api.cc:935: `ntree_limit` is deprecated, use `iteration_range` instead.
## [15:52:19] WARNING: src/c_api/c_api.cc:935: `ntree_limit` is deprecated, use `iteration_range` instead.
## [15:52:19] WARNING: src/c_api/c_api.cc:935: `ntree_limit` is deprecated, use `iteration_range` instead.
## [15:52:19] WARNING: src/c_api/c_api.cc:935: `ntree_limit` is deprecated, use `iteration_range` instead.
## [15:52:20] WARNING: src/c_api/c_api.cc:935: `ntree_limit` is deprecated, use `iteration_range` instead.
## [15:52:20] WARNING: src/c_api/c_api.cc:935: `ntree_limit` is deprecated, use `iteration_range` instead.
## [15:52:20] WARNING: src/c_api/c_api.cc:935: `ntree_limit` is deprecated, use `iteration_range` instead.
## [15:52:20] WARNING: src/c_api/c_api.cc:935: `ntree_limit` is deprecated, use `iteration_range` instead.
## [15:52:20] WARNING: src/c_api/c_api.cc:935: `ntree_limit` is deprecated, use `iteration_range` instead.
## [15:52:20] WARNING: src/c_api/c_api.cc:935: `ntree_limit` is deprecated, use `iteration_range` instead.
## [15:52:20] WARNING: src/c_api/c_api.cc:935: `ntree_limit` is deprecated, use `iteration_range` instead.
## [15:52:20] WARNING: src/c_api/c_api.cc:935: `ntree_limit` is deprecated, use `iteration_range` instead.
## [15:52:20] WARNING: src/c_api/c_api.cc:935: `ntree_limit` is deprecated, use `iteration_range` instead.
## [15:52:20] WARNING: src/c_api/c_api.cc:935: `ntree_limit` is deprecated, use `iteration_range` instead.
## [15:52:20] WARNING: src/c_api/c_api.cc:935: `ntree_limit` is deprecated, use `iteration_range` instead.
## [15:52:20] WARNING: src/c_api/c_api.cc:935: `ntree_limit` is deprecated, use `iteration_range` instead.
## [15:52:20] WARNING: src/c_api/c_api.cc:935: `ntree_limit` is deprecated, use `iteration_range` instead.
## [15:52:20] WARNING: src/c_api/c_api.cc:935: `ntree_limit` is deprecated, use `iteration_range` instead.
## [15:52:21] WARNING: src/c_api/c_api.cc:935: `ntree_limit` is deprecated, use `iteration_range` instead.
## [15:52:21] WARNING: src/c_api/c_api.cc:935: `ntree_limit` is deprecated, use `iteration_range` instead.
## [15:52:21] WARNING: src/c_api/c_api.cc:935: `ntree_limit` is deprecated, use `iteration_range` instead.
## [15:52:21] WARNING: src/c_api/c_api.cc:935: `ntree_limit` is deprecated, use `iteration_range` instead.
## [15:52:21] WARNING: src/c_api/c_api.cc:935: `ntree_limit` is deprecated, use `iteration_range` instead.
## [15:52:21] WARNING: src/c_api/c_api.cc:935: `ntree_limit` is deprecated, use `iteration_range` instead.
## [15:52:21] WARNING: src/c_api/c_api.cc:935: `ntree_limit` is deprecated, use `iteration_range` instead.
## [15:52:21] WARNING: src/c_api/c_api.cc:935: `ntree_limit` is deprecated, use `iteration_range` instead.
## [15:52:21] WARNING: src/c_api/c_api.cc:935: `ntree_limit` is deprecated, use `iteration_range` instead.
## [15:52:21] WARNING: src/c_api/c_api.cc:935: `ntree_limit` is deprecated, use `iteration_range` instead.
## [15:52:22] WARNING: src/c_api/c_api.cc:935: `ntree_limit` is deprecated, use `iteration_range` instead.
## [15:52:22] WARNING: src/c_api/c_api.cc:935: `ntree_limit` is deprecated, use `iteration_range` instead.
## [15:52:22] WARNING: src/c_api/c_api.cc:935: `ntree_limit` is deprecated, use `iteration_range` instead.
## [15:52:22] WARNING: src/c_api/c_api.cc:935: `ntree_limit` is deprecated, use `iteration_range` instead.
## [15:52:22] WARNING: src/c_api/c_api.cc:935: `ntree_limit` is deprecated, use `iteration_range` instead.
## [15:52:22] WARNING: src/c_api/c_api.cc:935: `ntree_limit` is deprecated, use `iteration_range` instead.
## [15:52:22] WARNING: src/c_api/c_api.cc:935: `ntree_limit` is deprecated, use `iteration_range` instead.
## [15:52:22] WARNING: src/c_api/c_api.cc:935: `ntree_limit` is deprecated, use `iteration_range` instead.
## [15:52:22] WARNING: src/c_api/c_api.cc:935: `ntree_limit` is deprecated, use `iteration_range` instead.
## [15:52:22] WARNING: src/c_api/c_api.cc:935: `ntree_limit` is deprecated, use `iteration_range` instead.
## [15:52:23] WARNING: src/c_api/c_api.cc:935: `ntree_limit` is deprecated, use `iteration_range` instead.
## [15:52:23] WARNING: src/c_api/c_api.cc:935: `ntree_limit` is deprecated, use `iteration_range` instead.
## [15:52:23] WARNING: src/c_api/c_api.cc:935: `ntree_limit` is deprecated, use `iteration_range` instead.
## [15:52:23] WARNING: src/c_api/c_api.cc:935: `ntree_limit` is deprecated, use `iteration_range` instead.
## [15:52:23] WARNING: src/c_api/c_api.cc:935: `ntree_limit` is deprecated, use `iteration_range` instead.
## [15:52:23] WARNING: src/c_api/c_api.cc:935: `ntree_limit` is deprecated, use `iteration_range` instead.
## [15:52:23] WARNING: src/c_api/c_api.cc:935: `ntree_limit` is deprecated, use `iteration_range` instead.
## [15:52:23] WARNING: src/c_api/c_api.cc:935: `ntree_limit` is deprecated, use `iteration_range` instead.
## [15:52:23] WARNING: src/c_api/c_api.cc:935: `ntree_limit` is deprecated, use `iteration_range` instead.
## [15:52:23] WARNING: src/c_api/c_api.cc:935: `ntree_limit` is deprecated, use `iteration_range` instead.
## [15:52:24] WARNING: src/c_api/c_api.cc:935: `ntree_limit` is deprecated, use `iteration_range` instead.
## [15:52:24] WARNING: src/c_api/c_api.cc:935: `ntree_limit` is deprecated, use `iteration_range` instead.
## [15:52:24] WARNING: src/c_api/c_api.cc:935: `ntree_limit` is deprecated, use `iteration_range` instead.
## [15:52:24] WARNING: src/c_api/c_api.cc:935: `ntree_limit` is deprecated, use `iteration_range` instead.
## [15:52:24] WARNING: src/c_api/c_api.cc:935: `ntree_limit` is deprecated, use `iteration_range` instead.
## [15:52:24] WARNING: src/c_api/c_api.cc:935: `ntree_limit` is deprecated, use `iteration_range` instead.
## [15:52:24] WARNING: src/c_api/c_api.cc:935: `ntree_limit` is deprecated, use `iteration_range` instead.
## [15:52:24] WARNING: src/c_api/c_api.cc:935: `ntree_limit` is deprecated, use `iteration_range` instead.
## [15:52:24] WARNING: src/c_api/c_api.cc:935: `ntree_limit` is deprecated, use `iteration_range` instead.
## [15:52:24] WARNING: src/c_api/c_api.cc:935: `ntree_limit` is deprecated, use `iteration_range` instead.
## [15:52:24] WARNING: src/c_api/c_api.cc:935: `ntree_limit` is deprecated, use `iteration_range` instead.
## [15:52:24] WARNING: src/c_api/c_api.cc:935: `ntree_limit` is deprecated, use `iteration_range` instead.
## [15:52:24] WARNING: src/c_api/c_api.cc:935: `ntree_limit` is deprecated, use `iteration_range` instead.
## [15:52:24] WARNING: src/c_api/c_api.cc:935: `ntree_limit` is deprecated, use `iteration_range` instead.
## [15:52:25] WARNING: src/c_api/c_api.cc:935: `ntree_limit` is deprecated, use `iteration_range` instead.
## [15:52:25] WARNING: src/c_api/c_api.cc:935: `ntree_limit` is deprecated, use `iteration_range` instead.
## [15:52:25] WARNING: src/c_api/c_api.cc:935: `ntree_limit` is deprecated, use `iteration_range` instead.
## [15:52:25] WARNING: src/c_api/c_api.cc:935: `ntree_limit` is deprecated, use `iteration_range` instead.
## [15:52:25] WARNING: src/c_api/c_api.cc:935: `ntree_limit` is deprecated, use `iteration_range` instead.
## [15:52:25] WARNING: src/c_api/c_api.cc:935: `ntree_limit` is deprecated, use `iteration_range` instead.
## [15:52:25] WARNING: src/c_api/c_api.cc:935: `ntree_limit` is deprecated, use `iteration_range` instead.
## [15:52:25] WARNING: src/c_api/c_api.cc:935: `ntree_limit` is deprecated, use `iteration_range` instead.
## [15:52:25] WARNING: src/c_api/c_api.cc:935: `ntree_limit` is deprecated, use `iteration_range` instead.
## [15:52:25] WARNING: src/c_api/c_api.cc:935: `ntree_limit` is deprecated, use `iteration_range` instead.
## [15:52:26] WARNING: src/c_api/c_api.cc:935: `ntree_limit` is deprecated, use `iteration_range` instead.
## [15:52:26] WARNING: src/c_api/c_api.cc:935: `ntree_limit` is deprecated, use `iteration_range` instead.
## [15:52:26] WARNING: src/c_api/c_api.cc:935: `ntree_limit` is deprecated, use `iteration_range` instead.
## [15:52:26] WARNING: src/c_api/c_api.cc:935: `ntree_limit` is deprecated, use `iteration_range` instead.
## [15:52:26] WARNING: src/c_api/c_api.cc:935: `ntree_limit` is deprecated, use `iteration_range` instead.
## [15:52:26] WARNING: src/c_api/c_api.cc:935: `ntree_limit` is deprecated, use `iteration_range` instead.
## [15:52:26] WARNING: src/c_api/c_api.cc:935: `ntree_limit` is deprecated, use `iteration_range` instead.
## [15:52:26] WARNING: src/c_api/c_api.cc:935: `ntree_limit` is deprecated, use `iteration_range` instead.
## [15:52:26] WARNING: src/c_api/c_api.cc:935: `ntree_limit` is deprecated, use `iteration_range` instead.
## [15:52:26] WARNING: src/c_api/c_api.cc:935: `ntree_limit` is deprecated, use `iteration_range` instead.
## [15:52:26] WARNING: src/c_api/c_api.cc:935: `ntree_limit` is deprecated, use `iteration_range` instead.
## [15:52:26] WARNING: src/c_api/c_api.cc:935: `ntree_limit` is deprecated, use `iteration_range` instead.
## [15:52:27] WARNING: src/c_api/c_api.cc:935: `ntree_limit` is deprecated, use `iteration_range` instead.
## [15:52:27] WARNING: src/c_api/c_api.cc:935: `ntree_limit` is deprecated, use `iteration_range` instead.
## [15:52:27] WARNING: src/c_api/c_api.cc:935: `ntree_limit` is deprecated, use `iteration_range` instead.
## [15:52:27] WARNING: src/c_api/c_api.cc:935: `ntree_limit` is deprecated, use `iteration_range` instead.
## [15:52:27] WARNING: src/c_api/c_api.cc:935: `ntree_limit` is deprecated, use `iteration_range` instead.
## [15:52:27] WARNING: src/c_api/c_api.cc:935: `ntree_limit` is deprecated, use `iteration_range` instead.
## [15:52:27] WARNING: src/c_api/c_api.cc:935: `ntree_limit` is deprecated, use `iteration_range` instead.
## [15:52:27] WARNING: src/c_api/c_api.cc:935: `ntree_limit` is deprecated, use `iteration_range` instead.
## [15:52:27] WARNING: src/c_api/c_api.cc:935: `ntree_limit` is deprecated, use `iteration_range` instead.
## [15:52:27] WARNING: src/c_api/c_api.cc:935: `ntree_limit` is deprecated, use `iteration_range` instead.
## [15:52:27] WARNING: src/c_api/c_api.cc:935: `ntree_limit` is deprecated, use `iteration_range` instead.
## [15:52:27] WARNING: src/c_api/c_api.cc:935: `ntree_limit` is deprecated, use `iteration_range` instead.
## [15:52:28] WARNING: src/c_api/c_api.cc:935: `ntree_limit` is deprecated, use `iteration_range` instead.
## [15:52:28] WARNING: src/c_api/c_api.cc:935: `ntree_limit` is deprecated, use `iteration_range` instead.
## [15:52:28] WARNING: src/c_api/c_api.cc:935: `ntree_limit` is deprecated, use `iteration_range` instead.
## [15:52:28] WARNING: src/c_api/c_api.cc:935: `ntree_limit` is deprecated, use `iteration_range` instead.
## [15:52:28] WARNING: src/c_api/c_api.cc:935: `ntree_limit` is deprecated, use `iteration_range` instead.
## [15:52:28] WARNING: src/c_api/c_api.cc:935: `ntree_limit` is deprecated, use `iteration_range` instead.
## [15:52:28] WARNING: src/c_api/c_api.cc:935: `ntree_limit` is deprecated, use `iteration_range` instead.
## [15:52:28] WARNING: src/c_api/c_api.cc:935: `ntree_limit` is deprecated, use `iteration_range` instead.
## [15:52:28] WARNING: src/c_api/c_api.cc:935: `ntree_limit` is deprecated, use `iteration_range` instead.
## [15:52:28] WARNING: src/c_api/c_api.cc:935: `ntree_limit` is deprecated, use `iteration_range` instead.
## [15:52:29] WARNING: src/c_api/c_api.cc:935: `ntree_limit` is deprecated, use `iteration_range` instead.
## [15:52:29] WARNING: src/c_api/c_api.cc:935: `ntree_limit` is deprecated, use `iteration_range` instead.
## [15:52:29] WARNING: src/c_api/c_api.cc:935: `ntree_limit` is deprecated, use `iteration_range` instead.
## [15:52:29] WARNING: src/c_api/c_api.cc:935: `ntree_limit` is deprecated, use `iteration_range` instead.
## [15:52:29] WARNING: src/c_api/c_api.cc:935: `ntree_limit` is deprecated, use `iteration_range` instead.
## [15:52:29] WARNING: src/c_api/c_api.cc:935: `ntree_limit` is deprecated, use `iteration_range` instead.
## [15:52:29] WARNING: src/c_api/c_api.cc:935: `ntree_limit` is deprecated, use `iteration_range` instead.
## [15:52:29] WARNING: src/c_api/c_api.cc:935: `ntree_limit` is deprecated, use `iteration_range` instead.
## [15:52:29] WARNING: src/c_api/c_api.cc:935: `ntree_limit` is deprecated, use `iteration_range` instead.
## [15:52:29] WARNING: src/c_api/c_api.cc:935: `ntree_limit` is deprecated, use `iteration_range` instead.
## [15:52:30] WARNING: src/c_api/c_api.cc:935: `ntree_limit` is deprecated, use `iteration_range` instead.
## [15:52:30] WARNING: src/c_api/c_api.cc:935: `ntree_limit` is deprecated, use `iteration_range` instead.
## [15:52:30] WARNING: src/c_api/c_api.cc:935: `ntree_limit` is deprecated, use `iteration_range` instead.
## [15:52:30] WARNING: src/c_api/c_api.cc:935: `ntree_limit` is deprecated, use `iteration_range` instead.
## [15:52:30] WARNING: src/c_api/c_api.cc:935: `ntree_limit` is deprecated, use `iteration_range` instead.
## [15:52:30] WARNING: src/c_api/c_api.cc:935: `ntree_limit` is deprecated, use `iteration_range` instead.
## [15:52:30] WARNING: src/c_api/c_api.cc:935: `ntree_limit` is deprecated, use `iteration_range` instead.
## [15:52:30] WARNING: src/c_api/c_api.cc:935: `ntree_limit` is deprecated, use `iteration_range` instead.
## [15:52:30] WARNING: src/c_api/c_api.cc:935: `ntree_limit` is deprecated, use `iteration_range` instead.
## [15:52:30] WARNING: src/c_api/c_api.cc:935: `ntree_limit` is deprecated, use `iteration_range` instead.
## [15:52:30] WARNING: src/c_api/c_api.cc:935: `ntree_limit` is deprecated, use `iteration_range` instead.
## [15:52:30] WARNING: src/c_api/c_api.cc:935: `ntree_limit` is deprecated, use `iteration_range` instead.
## [15:52:30] WARNING: src/c_api/c_api.cc:935: `ntree_limit` is deprecated, use `iteration_range` instead.
## [15:52:30] WARNING: src/c_api/c_api.cc:935: `ntree_limit` is deprecated, use `iteration_range` instead.
## [15:52:31] WARNING: src/c_api/c_api.cc:935: `ntree_limit` is deprecated, use `iteration_range` instead.
## [15:52:31] WARNING: src/c_api/c_api.cc:935: `ntree_limit` is deprecated, use `iteration_range` instead.
## [15:52:31] WARNING: src/c_api/c_api.cc:935: `ntree_limit` is deprecated, use `iteration_range` instead.
## [15:52:31] WARNING: src/c_api/c_api.cc:935: `ntree_limit` is deprecated, use `iteration_range` instead.
## [15:52:31] WARNING: src/c_api/c_api.cc:935: `ntree_limit` is deprecated, use `iteration_range` instead.
## [15:52:31] WARNING: src/c_api/c_api.cc:935: `ntree_limit` is deprecated, use `iteration_range` instead.
## [15:52:31] WARNING: src/c_api/c_api.cc:935: `ntree_limit` is deprecated, use `iteration_range` instead.
## [15:52:31] WARNING: src/c_api/c_api.cc:935: `ntree_limit` is deprecated, use `iteration_range` instead.
## [15:52:31] WARNING: src/c_api/c_api.cc:935: `ntree_limit` is deprecated, use `iteration_range` instead.
## [15:52:31] WARNING: src/c_api/c_api.cc:935: `ntree_limit` is deprecated, use `iteration_range` instead.
## [15:52:32] WARNING: src/c_api/c_api.cc:935: `ntree_limit` is deprecated, use `iteration_range` instead.
## [15:52:32] WARNING: src/c_api/c_api.cc:935: `ntree_limit` is deprecated, use `iteration_range` instead.
## [15:52:32] WARNING: src/c_api/c_api.cc:935: `ntree_limit` is deprecated, use `iteration_range` instead.
## [15:52:32] WARNING: src/c_api/c_api.cc:935: `ntree_limit` is deprecated, use `iteration_range` instead.
## [15:52:32] WARNING: src/c_api/c_api.cc:935: `ntree_limit` is deprecated, use `iteration_range` instead.
## [15:52:32] WARNING: src/c_api/c_api.cc:935: `ntree_limit` is deprecated, use `iteration_range` instead.
## [15:52:32] WARNING: src/c_api/c_api.cc:935: `ntree_limit` is deprecated, use `iteration_range` instead.
## [15:52:32] WARNING: src/c_api/c_api.cc:935: `ntree_limit` is deprecated, use `iteration_range` instead.
## [15:52:32] WARNING: src/c_api/c_api.cc:935: `ntree_limit` is deprecated, use `iteration_range` instead.
## [15:52:32] WARNING: src/c_api/c_api.cc:935: `ntree_limit` is deprecated, use `iteration_range` instead.
## [15:52:33] WARNING: src/c_api/c_api.cc:935: `ntree_limit` is deprecated, use `iteration_range` instead.
## [15:52:33] WARNING: src/c_api/c_api.cc:935: `ntree_limit` is deprecated, use `iteration_range` instead.
## [15:52:35] WARNING: src/c_api/c_api.cc:935: `ntree_limit` is deprecated, use `iteration_range` instead.
## [15:52:35] WARNING: src/c_api/c_api.cc:935: `ntree_limit` is deprecated, use `iteration_range` instead.
## [15:52:36] WARNING: src/c_api/c_api.cc:935: `ntree_limit` is deprecated, use `iteration_range` instead.
## [15:52:36] WARNING: src/c_api/c_api.cc:935: `ntree_limit` is deprecated, use `iteration_range` instead.
## [15:52:36] WARNING: src/c_api/c_api.cc:935: `ntree_limit` is deprecated, use `iteration_range` instead.
## [15:52:36] WARNING: src/c_api/c_api.cc:935: `ntree_limit` is deprecated, use `iteration_range` instead.
## [15:52:36] WARNING: src/c_api/c_api.cc:935: `ntree_limit` is deprecated, use `iteration_range` instead.
## [15:52:36] WARNING: src/c_api/c_api.cc:935: `ntree_limit` is deprecated, use `iteration_range` instead.
## [15:52:36] WARNING: src/c_api/c_api.cc:935: `ntree_limit` is deprecated, use `iteration_range` instead.
## [15:52:36] WARNING: src/c_api/c_api.cc:935: `ntree_limit` is deprecated, use `iteration_range` instead.
## [15:52:36] WARNING: src/c_api/c_api.cc:935: `ntree_limit` is deprecated, use `iteration_range` instead.
## [15:52:36] WARNING: src/c_api/c_api.cc:935: `ntree_limit` is deprecated, use `iteration_range` instead.
## [15:52:36] WARNING: src/c_api/c_api.cc:935: `ntree_limit` is deprecated, use `iteration_range` instead.
## [15:52:36] WARNING: src/c_api/c_api.cc:935: `ntree_limit` is deprecated, use `iteration_range` instead.
## [15:52:37] WARNING: src/c_api/c_api.cc:935: `ntree_limit` is deprecated, use `iteration_range` instead.
## [15:52:37] WARNING: src/c_api/c_api.cc:935: `ntree_limit` is deprecated, use `iteration_range` instead.
## [15:52:37] WARNING: src/c_api/c_api.cc:935: `ntree_limit` is deprecated, use `iteration_range` instead.
## [15:52:37] WARNING: src/c_api/c_api.cc:935: `ntree_limit` is deprecated, use `iteration_range` instead.
## [15:52:37] WARNING: src/c_api/c_api.cc:935: `ntree_limit` is deprecated, use `iteration_range` instead.
## [15:52:37] WARNING: src/c_api/c_api.cc:935: `ntree_limit` is deprecated, use `iteration_range` instead.
## [15:52:37] WARNING: src/c_api/c_api.cc:935: `ntree_limit` is deprecated, use `iteration_range` instead.
## [15:52:37] WARNING: src/c_api/c_api.cc:935: `ntree_limit` is deprecated, use `iteration_range` instead.
## [15:52:37] WARNING: src/c_api/c_api.cc:935: `ntree_limit` is deprecated, use `iteration_range` instead.
## [15:52:37] WARNING: src/c_api/c_api.cc:935: `ntree_limit` is deprecated, use `iteration_range` instead.
## [15:52:38] WARNING: src/c_api/c_api.cc:935: `ntree_limit` is deprecated, use `iteration_range` instead.
## [15:52:38] WARNING: src/c_api/c_api.cc:935: `ntree_limit` is deprecated, use `iteration_range` instead.
## [15:52:38] WARNING: src/c_api/c_api.cc:935: `ntree_limit` is deprecated, use `iteration_range` instead.
## [15:52:38] WARNING: src/c_api/c_api.cc:935: `ntree_limit` is deprecated, use `iteration_range` instead.
## [15:52:38] WARNING: src/c_api/c_api.cc:935: `ntree_limit` is deprecated, use `iteration_range` instead.
## [15:52:38] WARNING: src/c_api/c_api.cc:935: `ntree_limit` is deprecated, use `iteration_range` instead.
## [15:52:38] WARNING: src/c_api/c_api.cc:935: `ntree_limit` is deprecated, use `iteration_range` instead.
## [15:52:38] WARNING: src/c_api/c_api.cc:935: `ntree_limit` is deprecated, use `iteration_range` instead.
## [15:52:38] WARNING: src/c_api/c_api.cc:935: `ntree_limit` is deprecated, use `iteration_range` instead.
## [15:52:38] WARNING: src/c_api/c_api.cc:935: `ntree_limit` is deprecated, use `iteration_range` instead.
## [15:52:39] WARNING: src/c_api/c_api.cc:935: `ntree_limit` is deprecated, use `iteration_range` instead.
## [15:52:39] WARNING: src/c_api/c_api.cc:935: `ntree_limit` is deprecated, use `iteration_range` instead.
## [15:52:39] WARNING: src/c_api/c_api.cc:935: `ntree_limit` is deprecated, use `iteration_range` instead.
## [15:52:39] WARNING: src/c_api/c_api.cc:935: `ntree_limit` is deprecated, use `iteration_range` instead.
## [15:52:39] WARNING: src/c_api/c_api.cc:935: `ntree_limit` is deprecated, use `iteration_range` instead.
## [15:52:39] WARNING: src/c_api/c_api.cc:935: `ntree_limit` is deprecated, use `iteration_range` instead.
## [15:52:39] WARNING: src/c_api/c_api.cc:935: `ntree_limit` is deprecated, use `iteration_range` instead.
## [15:52:39] WARNING: src/c_api/c_api.cc:935: `ntree_limit` is deprecated, use `iteration_range` instead.
## [15:52:39] WARNING: src/c_api/c_api.cc:935: `ntree_limit` is deprecated, use `iteration_range` instead.
## [15:52:39] WARNING: src/c_api/c_api.cc:935: `ntree_limit` is deprecated, use `iteration_range` instead.
## [15:52:39] WARNING: src/c_api/c_api.cc:935: `ntree_limit` is deprecated, use `iteration_range` instead.
## [15:52:39] WARNING: src/c_api/c_api.cc:935: `ntree_limit` is deprecated, use `iteration_range` instead.
## [15:52:39] WARNING: src/c_api/c_api.cc:935: `ntree_limit` is deprecated, use `iteration_range` instead.
## [15:52:39] WARNING: src/c_api/c_api.cc:935: `ntree_limit` is deprecated, use `iteration_range` instead.
## [15:52:40] WARNING: src/c_api/c_api.cc:935: `ntree_limit` is deprecated, use `iteration_range` instead.
## [15:52:40] WARNING: src/c_api/c_api.cc:935: `ntree_limit` is deprecated, use `iteration_range` instead.
## [15:52:40] WARNING: src/c_api/c_api.cc:935: `ntree_limit` is deprecated, use `iteration_range` instead.
## [15:52:40] WARNING: src/c_api/c_api.cc:935: `ntree_limit` is deprecated, use `iteration_range` instead.
## [15:52:40] WARNING: src/c_api/c_api.cc:935: `ntree_limit` is deprecated, use `iteration_range` instead.
## [15:52:40] WARNING: src/c_api/c_api.cc:935: `ntree_limit` is deprecated, use `iteration_range` instead.
## [15:52:40] WARNING: src/c_api/c_api.cc:935: `ntree_limit` is deprecated, use `iteration_range` instead.
## [15:52:40] WARNING: src/c_api/c_api.cc:935: `ntree_limit` is deprecated, use `iteration_range` instead.
## [15:52:40] WARNING: src/c_api/c_api.cc:935: `ntree_limit` is deprecated, use `iteration_range` instead.
## [15:52:40] WARNING: src/c_api/c_api.cc:935: `ntree_limit` is deprecated, use `iteration_range` instead.
## [15:52:40] WARNING: src/c_api/c_api.cc:935: `ntree_limit` is deprecated, use `iteration_range` instead.
## [15:52:40] WARNING: src/c_api/c_api.cc:935: `ntree_limit` is deprecated, use `iteration_range` instead.
## [15:52:41] WARNING: src/c_api/c_api.cc:935: `ntree_limit` is deprecated, use `iteration_range` instead.
## [15:52:41] WARNING: src/c_api/c_api.cc:935: `ntree_limit` is deprecated, use `iteration_range` instead.
## [15:52:41] WARNING: src/c_api/c_api.cc:935: `ntree_limit` is deprecated, use `iteration_range` instead.
## [15:52:41] WARNING: src/c_api/c_api.cc:935: `ntree_limit` is deprecated, use `iteration_range` instead.
## [15:52:41] WARNING: src/c_api/c_api.cc:935: `ntree_limit` is deprecated, use `iteration_range` instead.
## [15:52:41] WARNING: src/c_api/c_api.cc:935: `ntree_limit` is deprecated, use `iteration_range` instead.
## [15:52:41] WARNING: src/c_api/c_api.cc:935: `ntree_limit` is deprecated, use `iteration_range` instead.
## [15:52:41] WARNING: src/c_api/c_api.cc:935: `ntree_limit` is deprecated, use `iteration_range` instead.
## [15:52:42] WARNING: src/c_api/c_api.cc:935: `ntree_limit` is deprecated, use `iteration_range` instead.
## [15:52:42] WARNING: src/c_api/c_api.cc:935: `ntree_limit` is deprecated, use `iteration_range` instead.
## [15:52:42] WARNING: src/c_api/c_api.cc:935: `ntree_limit` is deprecated, use `iteration_range` instead.
## [15:52:42] WARNING: src/c_api/c_api.cc:935: `ntree_limit` is deprecated, use `iteration_range` instead.
## [15:52:42] WARNING: src/c_api/c_api.cc:935: `ntree_limit` is deprecated, use `iteration_range` instead.
## [15:52:42] WARNING: src/c_api/c_api.cc:935: `ntree_limit` is deprecated, use `iteration_range` instead.
## [15:52:42] WARNING: src/c_api/c_api.cc:935: `ntree_limit` is deprecated, use `iteration_range` instead.
## [15:52:42] WARNING: src/c_api/c_api.cc:935: `ntree_limit` is deprecated, use `iteration_range` instead.
## [15:52:42] WARNING: src/c_api/c_api.cc:935: `ntree_limit` is deprecated, use `iteration_range` instead.
## [15:52:42] WARNING: src/c_api/c_api.cc:935: `ntree_limit` is deprecated, use `iteration_range` instead.
## [15:52:42] WARNING: src/c_api/c_api.cc:935: `ntree_limit` is deprecated, use `iteration_range` instead.
## [15:52:42] WARNING: src/c_api/c_api.cc:935: `ntree_limit` is deprecated, use `iteration_range` instead.
## [15:52:42] WARNING: src/c_api/c_api.cc:935: `ntree_limit` is deprecated, use `iteration_range` instead.
## [15:52:42] WARNING: src/c_api/c_api.cc:935: `ntree_limit` is deprecated, use `iteration_range` instead.
## [15:52:42] WARNING: src/c_api/c_api.cc:935: `ntree_limit` is deprecated, use `iteration_range` instead.
## [15:52:42] WARNING: src/c_api/c_api.cc:935: `ntree_limit` is deprecated, use `iteration_range` instead.
## [15:52:43] WARNING: src/c_api/c_api.cc:935: `ntree_limit` is deprecated, use `iteration_range` instead.
## [15:52:43] WARNING: src/c_api/c_api.cc:935: `ntree_limit` is deprecated, use `iteration_range` instead.
## [15:52:43] WARNING: src/c_api/c_api.cc:935: `ntree_limit` is deprecated, use `iteration_range` instead.
## [15:52:43] WARNING: src/c_api/c_api.cc:935: `ntree_limit` is deprecated, use `iteration_range` instead.
## [15:52:43] WARNING: src/c_api/c_api.cc:935: `ntree_limit` is deprecated, use `iteration_range` instead.
## [15:52:43] WARNING: src/c_api/c_api.cc:935: `ntree_limit` is deprecated, use `iteration_range` instead.
## [15:52:43] WARNING: src/c_api/c_api.cc:935: `ntree_limit` is deprecated, use `iteration_range` instead.
## [15:52:43] WARNING: src/c_api/c_api.cc:935: `ntree_limit` is deprecated, use `iteration_range` instead.
## [15:52:43] WARNING: src/c_api/c_api.cc:935: `ntree_limit` is deprecated, use `iteration_range` instead.
## [15:52:43] WARNING: src/c_api/c_api.cc:935: `ntree_limit` is deprecated, use `iteration_range` instead.
## [15:52:44] WARNING: src/c_api/c_api.cc:935: `ntree_limit` is deprecated, use `iteration_range` instead.
## [15:52:44] WARNING: src/c_api/c_api.cc:935: `ntree_limit` is deprecated, use `iteration_range` instead.
## [15:52:44] WARNING: src/c_api/c_api.cc:935: `ntree_limit` is deprecated, use `iteration_range` instead.
## [15:52:44] WARNING: src/c_api/c_api.cc:935: `ntree_limit` is deprecated, use `iteration_range` instead.
## [15:52:44] WARNING: src/c_api/c_api.cc:935: `ntree_limit` is deprecated, use `iteration_range` instead.
## [15:52:44] WARNING: src/c_api/c_api.cc:935: `ntree_limit` is deprecated, use `iteration_range` instead.
## [15:52:44] WARNING: src/c_api/c_api.cc:935: `ntree_limit` is deprecated, use `iteration_range` instead.
## [15:52:44] WARNING: src/c_api/c_api.cc:935: `ntree_limit` is deprecated, use `iteration_range` instead.
## [15:52:45] WARNING: src/c_api/c_api.cc:935: `ntree_limit` is deprecated, use `iteration_range` instead.
## [15:52:45] WARNING: src/c_api/c_api.cc:935: `ntree_limit` is deprecated, use `iteration_range` instead.
## [15:52:45] WARNING: src/c_api/c_api.cc:935: `ntree_limit` is deprecated, use `iteration_range` instead.
## [15:52:45] WARNING: src/c_api/c_api.cc:935: `ntree_limit` is deprecated, use `iteration_range` instead.
## [15:52:45] WARNING: src/c_api/c_api.cc:935: `ntree_limit` is deprecated, use `iteration_range` instead.
## [15:52:45] WARNING: src/c_api/c_api.cc:935: `ntree_limit` is deprecated, use `iteration_range` instead.
## [15:52:45] WARNING: src/c_api/c_api.cc:935: `ntree_limit` is deprecated, use `iteration_range` instead.
## [15:52:45] WARNING: src/c_api/c_api.cc:935: `ntree_limit` is deprecated, use `iteration_range` instead.
## [15:52:45] WARNING: src/c_api/c_api.cc:935: `ntree_limit` is deprecated, use `iteration_range` instead.
## [15:52:45] WARNING: src/c_api/c_api.cc:935: `ntree_limit` is deprecated, use `iteration_range` instead.
## [15:52:45] WARNING: src/c_api/c_api.cc:935: `ntree_limit` is deprecated, use `iteration_range` instead.
## [15:52:45] WARNING: src/c_api/c_api.cc:935: `ntree_limit` is deprecated, use `iteration_range` instead.
## [15:52:46] WARNING: src/c_api/c_api.cc:935: `ntree_limit` is deprecated, use `iteration_range` instead.
## [15:52:46] WARNING: src/c_api/c_api.cc:935: `ntree_limit` is deprecated, use `iteration_range` instead.
## [15:52:46] WARNING: src/c_api/c_api.cc:935: `ntree_limit` is deprecated, use `iteration_range` instead.
## [15:52:46] WARNING: src/c_api/c_api.cc:935: `ntree_limit` is deprecated, use `iteration_range` instead.
## [15:52:46] WARNING: src/c_api/c_api.cc:935: `ntree_limit` is deprecated, use `iteration_range` instead.
## [15:52:46] WARNING: src/c_api/c_api.cc:935: `ntree_limit` is deprecated, use `iteration_range` instead.
## [15:52:46] WARNING: src/c_api/c_api.cc:935: `ntree_limit` is deprecated, use `iteration_range` instead.
## [15:52:46] WARNING: src/c_api/c_api.cc:935: `ntree_limit` is deprecated, use `iteration_range` instead.
## [15:52:46] WARNING: src/c_api/c_api.cc:935: `ntree_limit` is deprecated, use `iteration_range` instead.
## [15:52:46] WARNING: src/c_api/c_api.cc:935: `ntree_limit` is deprecated, use `iteration_range` instead.
## [15:52:46] WARNING: src/c_api/c_api.cc:935: `ntree_limit` is deprecated, use `iteration_range` instead.
## [15:52:46] WARNING: src/c_api/c_api.cc:935: `ntree_limit` is deprecated, use `iteration_range` instead.
## [15:52:46] WARNING: src/c_api/c_api.cc:935: `ntree_limit` is deprecated, use `iteration_range` instead.
## [15:52:46] WARNING: src/c_api/c_api.cc:935: `ntree_limit` is deprecated, use `iteration_range` instead.
## [15:52:47] WARNING: src/c_api/c_api.cc:935: `ntree_limit` is deprecated, use `iteration_range` instead.
## [15:52:47] WARNING: src/c_api/c_api.cc:935: `ntree_limit` is deprecated, use `iteration_range` instead.
## [15:52:47] WARNING: src/c_api/c_api.cc:935: `ntree_limit` is deprecated, use `iteration_range` instead.
## [15:52:47] WARNING: src/c_api/c_api.cc:935: `ntree_limit` is deprecated, use `iteration_range` instead.
## [15:52:47] WARNING: src/c_api/c_api.cc:935: `ntree_limit` is deprecated, use `iteration_range` instead.
## [15:52:47] WARNING: src/c_api/c_api.cc:935: `ntree_limit` is deprecated, use `iteration_range` instead.
## [15:52:47] WARNING: src/c_api/c_api.cc:935: `ntree_limit` is deprecated, use `iteration_range` instead.
## [15:52:47] WARNING: src/c_api/c_api.cc:935: `ntree_limit` is deprecated, use `iteration_range` instead.
## [15:52:47] WARNING: src/c_api/c_api.cc:935: `ntree_limit` is deprecated, use `iteration_range` instead.
## [15:52:47] WARNING: src/c_api/c_api.cc:935: `ntree_limit` is deprecated, use `iteration_range` instead.
## [15:52:48] WARNING: src/c_api/c_api.cc:935: `ntree_limit` is deprecated, use `iteration_range` instead.
## [15:52:48] WARNING: src/c_api/c_api.cc:935: `ntree_limit` is deprecated, use `iteration_range` instead.
## [15:52:48] WARNING: src/c_api/c_api.cc:935: `ntree_limit` is deprecated, use `iteration_range` instead.
## [15:52:48] WARNING: src/c_api/c_api.cc:935: `ntree_limit` is deprecated, use `iteration_range` instead.
## [15:52:48] WARNING: src/c_api/c_api.cc:935: `ntree_limit` is deprecated, use `iteration_range` instead.
## [15:52:48] WARNING: src/c_api/c_api.cc:935: `ntree_limit` is deprecated, use `iteration_range` instead.
## [15:52:48] WARNING: src/c_api/c_api.cc:935: `ntree_limit` is deprecated, use `iteration_range` instead.
## [15:52:48] WARNING: src/c_api/c_api.cc:935: `ntree_limit` is deprecated, use `iteration_range` instead.
## [15:52:48] WARNING: src/c_api/c_api.cc:935: `ntree_limit` is deprecated, use `iteration_range` instead.
## [15:52:48] WARNING: src/c_api/c_api.cc:935: `ntree_limit` is deprecated, use `iteration_range` instead.
## [15:52:48] WARNING: src/c_api/c_api.cc:935: `ntree_limit` is deprecated, use `iteration_range` instead.
## [15:52:48] WARNING: src/c_api/c_api.cc:935: `ntree_limit` is deprecated, use `iteration_range` instead.
## [15:52:49] WARNING: src/c_api/c_api.cc:935: `ntree_limit` is deprecated, use `iteration_range` instead.
## [15:52:49] WARNING: src/c_api/c_api.cc:935: `ntree_limit` is deprecated, use `iteration_range` instead.
## [15:52:49] WARNING: src/c_api/c_api.cc:935: `ntree_limit` is deprecated, use `iteration_range` instead.
## [15:52:49] WARNING: src/c_api/c_api.cc:935: `ntree_limit` is deprecated, use `iteration_range` instead.
## [15:52:49] WARNING: src/c_api/c_api.cc:935: `ntree_limit` is deprecated, use `iteration_range` instead.
## [15:52:49] WARNING: src/c_api/c_api.cc:935: `ntree_limit` is deprecated, use `iteration_range` instead.
## [15:52:49] WARNING: src/c_api/c_api.cc:935: `ntree_limit` is deprecated, use `iteration_range` instead.
## [15:52:49] WARNING: src/c_api/c_api.cc:935: `ntree_limit` is deprecated, use `iteration_range` instead.
## [15:52:49] WARNING: src/c_api/c_api.cc:935: `ntree_limit` is deprecated, use `iteration_range` instead.
## [15:52:49] WARNING: src/c_api/c_api.cc:935: `ntree_limit` is deprecated, use `iteration_range` instead.
## [15:52:49] WARNING: src/c_api/c_api.cc:935: `ntree_limit` is deprecated, use `iteration_range` instead.
## [15:52:49] WARNING: src/c_api/c_api.cc:935: `ntree_limit` is deprecated, use `iteration_range` instead.
## [15:52:50] WARNING: src/c_api/c_api.cc:935: `ntree_limit` is deprecated, use `iteration_range` instead.
## [15:52:50] WARNING: src/c_api/c_api.cc:935: `ntree_limit` is deprecated, use `iteration_range` instead.
## [15:52:50] WARNING: src/c_api/c_api.cc:935: `ntree_limit` is deprecated, use `iteration_range` instead.
## [15:52:50] WARNING: src/c_api/c_api.cc:935: `ntree_limit` is deprecated, use `iteration_range` instead.
## [15:52:50] WARNING: src/c_api/c_api.cc:935: `ntree_limit` is deprecated, use `iteration_range` instead.
## [15:52:50] WARNING: src/c_api/c_api.cc:935: `ntree_limit` is deprecated, use `iteration_range` instead.
## [15:52:50] WARNING: src/c_api/c_api.cc:935: `ntree_limit` is deprecated, use `iteration_range` instead.
## [15:52:50] WARNING: src/c_api/c_api.cc:935: `ntree_limit` is deprecated, use `iteration_range` instead.
## [15:52:50] WARNING: src/c_api/c_api.cc:935: `ntree_limit` is deprecated, use `iteration_range` instead.
## [15:52:50] WARNING: src/c_api/c_api.cc:935: `ntree_limit` is deprecated, use `iteration_range` instead.
## [15:52:51] WARNING: src/c_api/c_api.cc:935: `ntree_limit` is deprecated, use `iteration_range` instead.
## [15:52:51] WARNING: src/c_api/c_api.cc:935: `ntree_limit` is deprecated, use `iteration_range` instead.
## [15:52:51] WARNING: src/c_api/c_api.cc:935: `ntree_limit` is deprecated, use `iteration_range` instead.
## [15:52:51] WARNING: src/c_api/c_api.cc:935: `ntree_limit` is deprecated, use `iteration_range` instead.
## [15:52:51] WARNING: src/c_api/c_api.cc:935: `ntree_limit` is deprecated, use `iteration_range` instead.
## [15:52:51] WARNING: src/c_api/c_api.cc:935: `ntree_limit` is deprecated, use `iteration_range` instead.
## [15:52:51] WARNING: src/c_api/c_api.cc:935: `ntree_limit` is deprecated, use `iteration_range` instead.
## [15:52:51] WARNING: src/c_api/c_api.cc:935: `ntree_limit` is deprecated, use `iteration_range` instead.
## [15:52:52] WARNING: src/c_api/c_api.cc:935: `ntree_limit` is deprecated, use `iteration_range` instead.
## [15:52:52] WARNING: src/c_api/c_api.cc:935: `ntree_limit` is deprecated, use `iteration_range` instead.
## [15:52:52] WARNING: src/c_api/c_api.cc:935: `ntree_limit` is deprecated, use `iteration_range` instead.
## [15:52:52] WARNING: src/c_api/c_api.cc:935: `ntree_limit` is deprecated, use `iteration_range` instead.
## [15:52:52] WARNING: src/c_api/c_api.cc:935: `ntree_limit` is deprecated, use `iteration_range` instead.
## [15:52:52] WARNING: src/c_api/c_api.cc:935: `ntree_limit` is deprecated, use `iteration_range` instead.
## [15:52:52] WARNING: src/c_api/c_api.cc:935: `ntree_limit` is deprecated, use `iteration_range` instead.
## [15:52:52] WARNING: src/c_api/c_api.cc:935: `ntree_limit` is deprecated, use `iteration_range` instead.
## [15:52:52] WARNING: src/c_api/c_api.cc:935: `ntree_limit` is deprecated, use `iteration_range` instead.
## [15:52:52] WARNING: src/c_api/c_api.cc:935: `ntree_limit` is deprecated, use `iteration_range` instead.
## [15:52:52] WARNING: src/c_api/c_api.cc:935: `ntree_limit` is deprecated, use `iteration_range` instead.
## [15:52:52] WARNING: src/c_api/c_api.cc:935: `ntree_limit` is deprecated, use `iteration_range` instead.
## [15:52:52] WARNING: src/c_api/c_api.cc:935: `ntree_limit` is deprecated, use `iteration_range` instead.
## [15:52:52] WARNING: src/c_api/c_api.cc:935: `ntree_limit` is deprecated, use `iteration_range` instead.
## [15:52:53] WARNING: src/c_api/c_api.cc:935: `ntree_limit` is deprecated, use `iteration_range` instead.
## [15:52:53] WARNING: src/c_api/c_api.cc:935: `ntree_limit` is deprecated, use `iteration_range` instead.
## [15:52:53] WARNING: src/c_api/c_api.cc:935: `ntree_limit` is deprecated, use `iteration_range` instead.
## [15:52:53] WARNING: src/c_api/c_api.cc:935: `ntree_limit` is deprecated, use `iteration_range` instead.
## [15:52:53] WARNING: src/c_api/c_api.cc:935: `ntree_limit` is deprecated, use `iteration_range` instead.
## [15:52:53] WARNING: src/c_api/c_api.cc:935: `ntree_limit` is deprecated, use `iteration_range` instead.
## [15:52:53] WARNING: src/c_api/c_api.cc:935: `ntree_limit` is deprecated, use `iteration_range` instead.
## [15:52:53] WARNING: src/c_api/c_api.cc:935: `ntree_limit` is deprecated, use `iteration_range` instead.
## [15:52:53] WARNING: src/c_api/c_api.cc:935: `ntree_limit` is deprecated, use `iteration_range` instead.
## [15:52:53] WARNING: src/c_api/c_api.cc:935: `ntree_limit` is deprecated, use `iteration_range` instead.
## [15:52:53] WARNING: src/c_api/c_api.cc:935: `ntree_limit` is deprecated, use `iteration_range` instead.
## [15:52:53] WARNING: src/c_api/c_api.cc:935: `ntree_limit` is deprecated, use `iteration_range` instead.
## [15:52:54] WARNING: src/c_api/c_api.cc:935: `ntree_limit` is deprecated, use `iteration_range` instead.
## [15:52:54] WARNING: src/c_api/c_api.cc:935: `ntree_limit` is deprecated, use `iteration_range` instead.
## [15:52:54] WARNING: src/c_api/c_api.cc:935: `ntree_limit` is deprecated, use `iteration_range` instead.
## [15:52:54] WARNING: src/c_api/c_api.cc:935: `ntree_limit` is deprecated, use `iteration_range` instead.
## [15:52:54] WARNING: src/c_api/c_api.cc:935: `ntree_limit` is deprecated, use `iteration_range` instead.
## [15:52:54] WARNING: src/c_api/c_api.cc:935: `ntree_limit` is deprecated, use `iteration_range` instead.
## [15:52:54] WARNING: src/c_api/c_api.cc:935: `ntree_limit` is deprecated, use `iteration_range` instead.
## [15:52:54] WARNING: src/c_api/c_api.cc:935: `ntree_limit` is deprecated, use `iteration_range` instead.
## [15:52:54] WARNING: src/c_api/c_api.cc:935: `ntree_limit` is deprecated, use `iteration_range` instead.
## [15:52:54] WARNING: src/c_api/c_api.cc:935: `ntree_limit` is deprecated, use `iteration_range` instead.
## [15:52:55] WARNING: src/c_api/c_api.cc:935: `ntree_limit` is deprecated, use `iteration_range` instead.
## [15:52:55] WARNING: src/c_api/c_api.cc:935: `ntree_limit` is deprecated, use `iteration_range` instead.
## [15:52:55] WARNING: src/c_api/c_api.cc:935: `ntree_limit` is deprecated, use `iteration_range` instead.
## [15:52:55] WARNING: src/c_api/c_api.cc:935: `ntree_limit` is deprecated, use `iteration_range` instead.
## [15:52:55] WARNING: src/c_api/c_api.cc:935: `ntree_limit` is deprecated, use `iteration_range` instead.
## [15:52:55] WARNING: src/c_api/c_api.cc:935: `ntree_limit` is deprecated, use `iteration_range` instead.
## [15:52:55] WARNING: src/c_api/c_api.cc:935: `ntree_limit` is deprecated, use `iteration_range` instead.
## [15:52:55] WARNING: src/c_api/c_api.cc:935: `ntree_limit` is deprecated, use `iteration_range` instead.
## [15:52:55] WARNING: src/c_api/c_api.cc:935: `ntree_limit` is deprecated, use `iteration_range` instead.
## [15:52:55] WARNING: src/c_api/c_api.cc:935: `ntree_limit` is deprecated, use `iteration_range` instead.
## [15:52:55] WARNING: src/c_api/c_api.cc:935: `ntree_limit` is deprecated, use `iteration_range` instead.
## [15:52:55] WARNING: src/c_api/c_api.cc:935: `ntree_limit` is deprecated, use `iteration_range` instead.
## [15:52:55] WARNING: src/c_api/c_api.cc:935: `ntree_limit` is deprecated, use `iteration_range` instead.
## [15:52:55] WARNING: src/c_api/c_api.cc:935: `ntree_limit` is deprecated, use `iteration_range` instead.
## [15:52:56] WARNING: src/c_api/c_api.cc:935: `ntree_limit` is deprecated, use `iteration_range` instead.
## [15:52:56] WARNING: src/c_api/c_api.cc:935: `ntree_limit` is deprecated, use `iteration_range` instead.
## [15:52:56] WARNING: src/c_api/c_api.cc:935: `ntree_limit` is deprecated, use `iteration_range` instead.
## [15:52:56] WARNING: src/c_api/c_api.cc:935: `ntree_limit` is deprecated, use `iteration_range` instead.
## [15:52:56] WARNING: src/c_api/c_api.cc:935: `ntree_limit` is deprecated, use `iteration_range` instead.
## [15:52:56] WARNING: src/c_api/c_api.cc:935: `ntree_limit` is deprecated, use `iteration_range` instead.
## [15:52:56] WARNING: src/c_api/c_api.cc:935: `ntree_limit` is deprecated, use `iteration_range` instead.
## [15:52:56] WARNING: src/c_api/c_api.cc:935: `ntree_limit` is deprecated, use `iteration_range` instead.
## [15:52:56] WARNING: src/c_api/c_api.cc:935: `ntree_limit` is deprecated, use `iteration_range` instead.
## [15:52:56] WARNING: src/c_api/c_api.cc:935: `ntree_limit` is deprecated, use `iteration_range` instead.
## [15:52:57] WARNING: src/c_api/c_api.cc:935: `ntree_limit` is deprecated, use `iteration_range` instead.
## [15:52:57] WARNING: src/c_api/c_api.cc:935: `ntree_limit` is deprecated, use `iteration_range` instead.
## [15:52:57] WARNING: src/c_api/c_api.cc:935: `ntree_limit` is deprecated, use `iteration_range` instead.
## [15:52:57] WARNING: src/c_api/c_api.cc:935: `ntree_limit` is deprecated, use `iteration_range` instead.
## [15:52:57] WARNING: src/c_api/c_api.cc:935: `ntree_limit` is deprecated, use `iteration_range` instead.
## [15:52:57] WARNING: src/c_api/c_api.cc:935: `ntree_limit` is deprecated, use `iteration_range` instead.
## [15:52:57] WARNING: src/c_api/c_api.cc:935: `ntree_limit` is deprecated, use `iteration_range` instead.
## [15:52:57] WARNING: src/c_api/c_api.cc:935: `ntree_limit` is deprecated, use `iteration_range` instead.
## [15:52:57] WARNING: src/c_api/c_api.cc:935: `ntree_limit` is deprecated, use `iteration_range` instead.
## [15:52:57] WARNING: src/c_api/c_api.cc:935: `ntree_limit` is deprecated, use `iteration_range` instead.
## [15:52:58] WARNING: src/c_api/c_api.cc:935: `ntree_limit` is deprecated, use `iteration_range` instead.
## [15:52:58] WARNING: src/c_api/c_api.cc:935: `ntree_limit` is deprecated, use `iteration_range` instead.
## [15:52:58] WARNING: src/c_api/c_api.cc:935: `ntree_limit` is deprecated, use `iteration_range` instead.
## [15:52:58] WARNING: src/c_api/c_api.cc:935: `ntree_limit` is deprecated, use `iteration_range` instead.
## [15:52:58] WARNING: src/c_api/c_api.cc:935: `ntree_limit` is deprecated, use `iteration_range` instead.
## [15:52:58] WARNING: src/c_api/c_api.cc:935: `ntree_limit` is deprecated, use `iteration_range` instead.
## [15:52:58] WARNING: src/c_api/c_api.cc:935: `ntree_limit` is deprecated, use `iteration_range` instead.
## [15:52:58] WARNING: src/c_api/c_api.cc:935: `ntree_limit` is deprecated, use `iteration_range` instead.
## [15:52:58] WARNING: src/c_api/c_api.cc:935: `ntree_limit` is deprecated, use `iteration_range` instead.
## [15:52:58] WARNING: src/c_api/c_api.cc:935: `ntree_limit` is deprecated, use `iteration_range` instead.
## [15:52:58] WARNING: src/c_api/c_api.cc:935: `ntree_limit` is deprecated, use `iteration_range` instead.
## [15:52:58] WARNING: src/c_api/c_api.cc:935: `ntree_limit` is deprecated, use `iteration_range` instead.
## [15:52:58] WARNING: src/c_api/c_api.cc:935: `ntree_limit` is deprecated, use `iteration_range` instead.
## [15:52:58] WARNING: src/c_api/c_api.cc:935: `ntree_limit` is deprecated, use `iteration_range` instead.
## [15:52:59] WARNING: src/c_api/c_api.cc:935: `ntree_limit` is deprecated, use `iteration_range` instead.
## [15:52:59] WARNING: src/c_api/c_api.cc:935: `ntree_limit` is deprecated, use `iteration_range` instead.
## [15:52:59] WARNING: src/c_api/c_api.cc:935: `ntree_limit` is deprecated, use `iteration_range` instead.
## [15:52:59] WARNING: src/c_api/c_api.cc:935: `ntree_limit` is deprecated, use `iteration_range` instead.
## [15:52:59] WARNING: src/c_api/c_api.cc:935: `ntree_limit` is deprecated, use `iteration_range` instead.
## [15:52:59] WARNING: src/c_api/c_api.cc:935: `ntree_limit` is deprecated, use `iteration_range` instead.
## [15:52:59] WARNING: src/c_api/c_api.cc:935: `ntree_limit` is deprecated, use `iteration_range` instead.
## [15:52:59] WARNING: src/c_api/c_api.cc:935: `ntree_limit` is deprecated, use `iteration_range` instead.
## [15:52:59] WARNING: src/c_api/c_api.cc:935: `ntree_limit` is deprecated, use `iteration_range` instead.
## [15:52:59] WARNING: src/c_api/c_api.cc:935: `ntree_limit` is deprecated, use `iteration_range` instead.
## [15:53:00] WARNING: src/c_api/c_api.cc:935: `ntree_limit` is deprecated, use `iteration_range` instead.
## [15:53:00] WARNING: src/c_api/c_api.cc:935: `ntree_limit` is deprecated, use `iteration_range` instead.
## [15:53:00] WARNING: src/c_api/c_api.cc:935: `ntree_limit` is deprecated, use `iteration_range` instead.
## [15:53:00] WARNING: src/c_api/c_api.cc:935: `ntree_limit` is deprecated, use `iteration_range` instead.
## [15:53:00] WARNING: src/c_api/c_api.cc:935: `ntree_limit` is deprecated, use `iteration_range` instead.
## [15:53:00] WARNING: src/c_api/c_api.cc:935: `ntree_limit` is deprecated, use `iteration_range` instead.
## [15:53:00] WARNING: src/c_api/c_api.cc:935: `ntree_limit` is deprecated, use `iteration_range` instead.
## [15:53:00] WARNING: src/c_api/c_api.cc:935: `ntree_limit` is deprecated, use `iteration_range` instead.
## [15:53:00] WARNING: src/c_api/c_api.cc:935: `ntree_limit` is deprecated, use `iteration_range` instead.
## [15:53:00] WARNING: src/c_api/c_api.cc:935: `ntree_limit` is deprecated, use `iteration_range` instead.
## [15:53:01] WARNING: src/c_api/c_api.cc:935: `ntree_limit` is deprecated, use `iteration_range` instead.
## [15:53:01] WARNING: src/c_api/c_api.cc:935: `ntree_limit` is deprecated, use `iteration_range` instead.
## [15:53:01] WARNING: src/c_api/c_api.cc:935: `ntree_limit` is deprecated, use `iteration_range` instead.
## [15:53:01] WARNING: src/c_api/c_api.cc:935: `ntree_limit` is deprecated, use `iteration_range` instead.
## [15:53:04] WARNING: src/c_api/c_api.cc:935: `ntree_limit` is deprecated, use `iteration_range` instead.
## [15:53:04] WARNING: src/c_api/c_api.cc:935: `ntree_limit` is deprecated, use `iteration_range` instead.
## [15:53:04] WARNING: src/c_api/c_api.cc:935: `ntree_limit` is deprecated, use `iteration_range` instead.
## [15:53:04] WARNING: src/c_api/c_api.cc:935: `ntree_limit` is deprecated, use `iteration_range` instead.
## [15:53:04] WARNING: src/c_api/c_api.cc:935: `ntree_limit` is deprecated, use `iteration_range` instead.
## [15:53:04] WARNING: src/c_api/c_api.cc:935: `ntree_limit` is deprecated, use `iteration_range` instead.
## [15:53:04] WARNING: src/c_api/c_api.cc:935: `ntree_limit` is deprecated, use `iteration_range` instead.
## [15:53:04] WARNING: src/c_api/c_api.cc:935: `ntree_limit` is deprecated, use `iteration_range` instead.
## [15:53:04] WARNING: src/c_api/c_api.cc:935: `ntree_limit` is deprecated, use `iteration_range` instead.
## [15:53:04] WARNING: src/c_api/c_api.cc:935: `ntree_limit` is deprecated, use `iteration_range` instead.
## [15:53:05] WARNING: src/c_api/c_api.cc:935: `ntree_limit` is deprecated, use `iteration_range` instead.
## [15:53:05] WARNING: src/c_api/c_api.cc:935: `ntree_limit` is deprecated, use `iteration_range` instead.
## [15:53:05] WARNING: src/c_api/c_api.cc:935: `ntree_limit` is deprecated, use `iteration_range` instead.
## [15:53:05] WARNING: src/c_api/c_api.cc:935: `ntree_limit` is deprecated, use `iteration_range` instead.
## [15:53:05] WARNING: src/c_api/c_api.cc:935: `ntree_limit` is deprecated, use `iteration_range` instead.
## [15:53:05] WARNING: src/c_api/c_api.cc:935: `ntree_limit` is deprecated, use `iteration_range` instead.
## [15:53:05] WARNING: src/c_api/c_api.cc:935: `ntree_limit` is deprecated, use `iteration_range` instead.
## [15:53:05] WARNING: src/c_api/c_api.cc:935: `ntree_limit` is deprecated, use `iteration_range` instead.
## [15:53:05] WARNING: src/c_api/c_api.cc:935: `ntree_limit` is deprecated, use `iteration_range` instead.
## [15:53:05] WARNING: src/c_api/c_api.cc:935: `ntree_limit` is deprecated, use `iteration_range` instead.
## [15:53:06] WARNING: src/c_api/c_api.cc:935: `ntree_limit` is deprecated, use `iteration_range` instead.
## [15:53:06] WARNING: src/c_api/c_api.cc:935: `ntree_limit` is deprecated, use `iteration_range` instead.
## [15:53:06] WARNING: src/c_api/c_api.cc:935: `ntree_limit` is deprecated, use `iteration_range` instead.
## [15:53:06] WARNING: src/c_api/c_api.cc:935: `ntree_limit` is deprecated, use `iteration_range` instead.
## [15:53:06] WARNING: src/c_api/c_api.cc:935: `ntree_limit` is deprecated, use `iteration_range` instead.
## [15:53:06] WARNING: src/c_api/c_api.cc:935: `ntree_limit` is deprecated, use `iteration_range` instead.
## [15:53:06] WARNING: src/c_api/c_api.cc:935: `ntree_limit` is deprecated, use `iteration_range` instead.
## [15:53:06] WARNING: src/c_api/c_api.cc:935: `ntree_limit` is deprecated, use `iteration_range` instead.
## [15:53:06] WARNING: src/c_api/c_api.cc:935: `ntree_limit` is deprecated, use `iteration_range` instead.
## [15:53:06] WARNING: src/c_api/c_api.cc:935: `ntree_limit` is deprecated, use `iteration_range` instead.
## [15:53:07] WARNING: src/c_api/c_api.cc:935: `ntree_limit` is deprecated, use `iteration_range` instead.
## [15:53:07] WARNING: src/c_api/c_api.cc:935: `ntree_limit` is deprecated, use `iteration_range` instead.
## [15:53:07] WARNING: src/c_api/c_api.cc:935: `ntree_limit` is deprecated, use `iteration_range` instead.
## [15:53:07] WARNING: src/c_api/c_api.cc:935: `ntree_limit` is deprecated, use `iteration_range` instead.
## [15:53:07] WARNING: src/c_api/c_api.cc:935: `ntree_limit` is deprecated, use `iteration_range` instead.
## [15:53:07] WARNING: src/c_api/c_api.cc:935: `ntree_limit` is deprecated, use `iteration_range` instead.
## [15:53:07] WARNING: src/c_api/c_api.cc:935: `ntree_limit` is deprecated, use `iteration_range` instead.
## [15:53:07] WARNING: src/c_api/c_api.cc:935: `ntree_limit` is deprecated, use `iteration_range` instead.
## [15:53:07] WARNING: src/c_api/c_api.cc:935: `ntree_limit` is deprecated, use `iteration_range` instead.
## [15:53:07] WARNING: src/c_api/c_api.cc:935: `ntree_limit` is deprecated, use `iteration_range` instead.
## [15:53:07] WARNING: src/c_api/c_api.cc:935: `ntree_limit` is deprecated, use `iteration_range` instead.
## [15:53:07] WARNING: src/c_api/c_api.cc:935: `ntree_limit` is deprecated, use `iteration_range` instead.
## [15:53:08] WARNING: src/c_api/c_api.cc:935: `ntree_limit` is deprecated, use `iteration_range` instead.
## [15:53:08] WARNING: src/c_api/c_api.cc:935: `ntree_limit` is deprecated, use `iteration_range` instead.
## [15:53:08] WARNING: src/c_api/c_api.cc:935: `ntree_limit` is deprecated, use `iteration_range` instead.
## [15:53:08] WARNING: src/c_api/c_api.cc:935: `ntree_limit` is deprecated, use `iteration_range` instead.
## [15:53:08] WARNING: src/c_api/c_api.cc:935: `ntree_limit` is deprecated, use `iteration_range` instead.
## [15:53:08] WARNING: src/c_api/c_api.cc:935: `ntree_limit` is deprecated, use `iteration_range` instead.
## [15:53:08] WARNING: src/c_api/c_api.cc:935: `ntree_limit` is deprecated, use `iteration_range` instead.
## [15:53:08] WARNING: src/c_api/c_api.cc:935: `ntree_limit` is deprecated, use `iteration_range` instead.
## [15:53:08] WARNING: src/c_api/c_api.cc:935: `ntree_limit` is deprecated, use `iteration_range` instead.
## [15:53:08] WARNING: src/c_api/c_api.cc:935: `ntree_limit` is deprecated, use `iteration_range` instead.
## [15:53:08] WARNING: src/c_api/c_api.cc:935: `ntree_limit` is deprecated, use `iteration_range` instead.
## [15:53:08] WARNING: src/c_api/c_api.cc:935: `ntree_limit` is deprecated, use `iteration_range` instead.
## [15:53:09] WARNING: src/c_api/c_api.cc:935: `ntree_limit` is deprecated, use `iteration_range` instead.
## [15:53:09] WARNING: src/c_api/c_api.cc:935: `ntree_limit` is deprecated, use `iteration_range` instead.
## [15:53:09] WARNING: src/c_api/c_api.cc:935: `ntree_limit` is deprecated, use `iteration_range` instead.
## [15:53:09] WARNING: src/c_api/c_api.cc:935: `ntree_limit` is deprecated, use `iteration_range` instead.
## [15:53:09] WARNING: src/c_api/c_api.cc:935: `ntree_limit` is deprecated, use `iteration_range` instead.
## [15:53:09] WARNING: src/c_api/c_api.cc:935: `ntree_limit` is deprecated, use `iteration_range` instead.
## [15:53:09] WARNING: src/c_api/c_api.cc:935: `ntree_limit` is deprecated, use `iteration_range` instead.
## [15:53:09] WARNING: src/c_api/c_api.cc:935: `ntree_limit` is deprecated, use `iteration_range` instead.
## [15:53:09] WARNING: src/c_api/c_api.cc:935: `ntree_limit` is deprecated, use `iteration_range` instead.
## [15:53:09] WARNING: src/c_api/c_api.cc:935: `ntree_limit` is deprecated, use `iteration_range` instead.
## [15:53:09] WARNING: src/c_api/c_api.cc:935: `ntree_limit` is deprecated, use `iteration_range` instead.
## [15:53:09] WARNING: src/c_api/c_api.cc:935: `ntree_limit` is deprecated, use `iteration_range` instead.
## [15:53:10] WARNING: src/c_api/c_api.cc:935: `ntree_limit` is deprecated, use `iteration_range` instead.
## [15:53:10] WARNING: src/c_api/c_api.cc:935: `ntree_limit` is deprecated, use `iteration_range` instead.
## [15:53:10] WARNING: src/c_api/c_api.cc:935: `ntree_limit` is deprecated, use `iteration_range` instead.
## [15:53:10] WARNING: src/c_api/c_api.cc:935: `ntree_limit` is deprecated, use `iteration_range` instead.
## [15:53:10] WARNING: src/c_api/c_api.cc:935: `ntree_limit` is deprecated, use `iteration_range` instead.
## [15:53:10] WARNING: src/c_api/c_api.cc:935: `ntree_limit` is deprecated, use `iteration_range` instead.
## [15:53:10] WARNING: src/c_api/c_api.cc:935: `ntree_limit` is deprecated, use `iteration_range` instead.
## [15:53:10] WARNING: src/c_api/c_api.cc:935: `ntree_limit` is deprecated, use `iteration_range` instead.
## [15:53:10] WARNING: src/c_api/c_api.cc:935: `ntree_limit` is deprecated, use `iteration_range` instead.
## [15:53:10] WARNING: src/c_api/c_api.cc:935: `ntree_limit` is deprecated, use `iteration_range` instead.
## [15:53:11] WARNING: src/c_api/c_api.cc:935: `ntree_limit` is deprecated, use `iteration_range` instead.
## [15:53:11] WARNING: src/c_api/c_api.cc:935: `ntree_limit` is deprecated, use `iteration_range` instead.
## [15:53:11] WARNING: src/c_api/c_api.cc:935: `ntree_limit` is deprecated, use `iteration_range` instead.
## [15:53:11] WARNING: src/c_api/c_api.cc:935: `ntree_limit` is deprecated, use `iteration_range` instead.
## [15:53:11] WARNING: src/c_api/c_api.cc:935: `ntree_limit` is deprecated, use `iteration_range` instead.
## [15:53:11] WARNING: src/c_api/c_api.cc:935: `ntree_limit` is deprecated, use `iteration_range` instead.
## [15:53:11] WARNING: src/c_api/c_api.cc:935: `ntree_limit` is deprecated, use `iteration_range` instead.
## [15:53:11] WARNING: src/c_api/c_api.cc:935: `ntree_limit` is deprecated, use `iteration_range` instead.
## [15:53:11] WARNING: src/c_api/c_api.cc:935: `ntree_limit` is deprecated, use `iteration_range` instead.
## [15:53:11] WARNING: src/c_api/c_api.cc:935: `ntree_limit` is deprecated, use `iteration_range` instead.
## [15:53:11] WARNING: src/c_api/c_api.cc:935: `ntree_limit` is deprecated, use `iteration_range` instead.
## [15:53:11] WARNING: src/c_api/c_api.cc:935: `ntree_limit` is deprecated, use `iteration_range` instead.
## [15:53:12] WARNING: src/c_api/c_api.cc:935: `ntree_limit` is deprecated, use `iteration_range` instead.
## [15:53:12] WARNING: src/c_api/c_api.cc:935: `ntree_limit` is deprecated, use `iteration_range` instead.
## [15:53:12] WARNING: src/c_api/c_api.cc:935: `ntree_limit` is deprecated, use `iteration_range` instead.
## [15:53:12] WARNING: src/c_api/c_api.cc:935: `ntree_limit` is deprecated, use `iteration_range` instead.
## [15:53:12] WARNING: src/c_api/c_api.cc:935: `ntree_limit` is deprecated, use `iteration_range` instead.
## [15:53:12] WARNING: src/c_api/c_api.cc:935: `ntree_limit` is deprecated, use `iteration_range` instead.
## [15:53:12] WARNING: src/c_api/c_api.cc:935: `ntree_limit` is deprecated, use `iteration_range` instead.
## [15:53:12] WARNING: src/c_api/c_api.cc:935: `ntree_limit` is deprecated, use `iteration_range` instead.
## [15:53:12] WARNING: src/c_api/c_api.cc:935: `ntree_limit` is deprecated, use `iteration_range` instead.
## [15:53:12] WARNING: src/c_api/c_api.cc:935: `ntree_limit` is deprecated, use `iteration_range` instead.
## [15:53:13] WARNING: src/c_api/c_api.cc:935: `ntree_limit` is deprecated, use `iteration_range` instead.
## [15:53:13] WARNING: src/c_api/c_api.cc:935: `ntree_limit` is deprecated, use `iteration_range` instead.
## [15:53:13] WARNING: src/c_api/c_api.cc:935: `ntree_limit` is deprecated, use `iteration_range` instead.
## [15:53:13] WARNING: src/c_api/c_api.cc:935: `ntree_limit` is deprecated, use `iteration_range` instead.
## [15:53:13] WARNING: src/c_api/c_api.cc:935: `ntree_limit` is deprecated, use `iteration_range` instead.
## [15:53:13] WARNING: src/c_api/c_api.cc:935: `ntree_limit` is deprecated, use `iteration_range` instead.
## [15:53:13] WARNING: src/c_api/c_api.cc:935: `ntree_limit` is deprecated, use `iteration_range` instead.
## [15:53:13] WARNING: src/c_api/c_api.cc:935: `ntree_limit` is deprecated, use `iteration_range` instead.
## [15:53:13] WARNING: src/c_api/c_api.cc:935: `ntree_limit` is deprecated, use `iteration_range` instead.
## [15:53:13] WARNING: src/c_api/c_api.cc:935: `ntree_limit` is deprecated, use `iteration_range` instead.
## [15:53:14] WARNING: src/c_api/c_api.cc:935: `ntree_limit` is deprecated, use `iteration_range` instead.
## [15:53:14] WARNING: src/c_api/c_api.cc:935: `ntree_limit` is deprecated, use `iteration_range` instead.
## [15:53:14] WARNING: src/c_api/c_api.cc:935: `ntree_limit` is deprecated, use `iteration_range` instead.
## [15:53:14] WARNING: src/c_api/c_api.cc:935: `ntree_limit` is deprecated, use `iteration_range` instead.
## [15:53:14] WARNING: src/c_api/c_api.cc:935: `ntree_limit` is deprecated, use `iteration_range` instead.
## [15:53:14] WARNING: src/c_api/c_api.cc:935: `ntree_limit` is deprecated, use `iteration_range` instead.
## [15:53:14] WARNING: src/c_api/c_api.cc:935: `ntree_limit` is deprecated, use `iteration_range` instead.
## [15:53:14] WARNING: src/c_api/c_api.cc:935: `ntree_limit` is deprecated, use `iteration_range` instead.
## [15:53:14] WARNING: src/c_api/c_api.cc:935: `ntree_limit` is deprecated, use `iteration_range` instead.
## [15:53:14] WARNING: src/c_api/c_api.cc:935: `ntree_limit` is deprecated, use `iteration_range` instead.
## [15:53:14] WARNING: src/c_api/c_api.cc:935: `ntree_limit` is deprecated, use `iteration_range` instead.
## [15:53:14] WARNING: src/c_api/c_api.cc:935: `ntree_limit` is deprecated, use `iteration_range` instead.
## [15:53:14] WARNING: src/c_api/c_api.cc:935: `ntree_limit` is deprecated, use `iteration_range` instead.
## [15:53:14] WARNING: src/c_api/c_api.cc:935: `ntree_limit` is deprecated, use `iteration_range` instead.
## [15:53:15] WARNING: src/c_api/c_api.cc:935: `ntree_limit` is deprecated, use `iteration_range` instead.
## [15:53:15] WARNING: src/c_api/c_api.cc:935: `ntree_limit` is deprecated, use `iteration_range` instead.
## [15:53:15] WARNING: src/c_api/c_api.cc:935: `ntree_limit` is deprecated, use `iteration_range` instead.
## [15:53:15] WARNING: src/c_api/c_api.cc:935: `ntree_limit` is deprecated, use `iteration_range` instead.
## [15:53:15] WARNING: src/c_api/c_api.cc:935: `ntree_limit` is deprecated, use `iteration_range` instead.
## [15:53:15] WARNING: src/c_api/c_api.cc:935: `ntree_limit` is deprecated, use `iteration_range` instead.
## [15:53:15] WARNING: src/c_api/c_api.cc:935: `ntree_limit` is deprecated, use `iteration_range` instead.
## [15:53:15] WARNING: src/c_api/c_api.cc:935: `ntree_limit` is deprecated, use `iteration_range` instead.
## [15:53:15] WARNING: src/c_api/c_api.cc:935: `ntree_limit` is deprecated, use `iteration_range` instead.
## [15:53:15] WARNING: src/c_api/c_api.cc:935: `ntree_limit` is deprecated, use `iteration_range` instead.
## [15:53:15] WARNING: src/c_api/c_api.cc:935: `ntree_limit` is deprecated, use `iteration_range` instead.
## [15:53:15] WARNING: src/c_api/c_api.cc:935: `ntree_limit` is deprecated, use `iteration_range` instead.
## [15:53:16] WARNING: src/c_api/c_api.cc:935: `ntree_limit` is deprecated, use `iteration_range` instead.
## [15:53:16] WARNING: src/c_api/c_api.cc:935: `ntree_limit` is deprecated, use `iteration_range` instead.
## [15:53:16] WARNING: src/c_api/c_api.cc:935: `ntree_limit` is deprecated, use `iteration_range` instead.
## [15:53:16] WARNING: src/c_api/c_api.cc:935: `ntree_limit` is deprecated, use `iteration_range` instead.
## [15:53:16] WARNING: src/c_api/c_api.cc:935: `ntree_limit` is deprecated, use `iteration_range` instead.
## [15:53:16] WARNING: src/c_api/c_api.cc:935: `ntree_limit` is deprecated, use `iteration_range` instead.
## [15:53:16] WARNING: src/c_api/c_api.cc:935: `ntree_limit` is deprecated, use `iteration_range` instead.
## [15:53:16] WARNING: src/c_api/c_api.cc:935: `ntree_limit` is deprecated, use `iteration_range` instead.
## [15:53:16] WARNING: src/c_api/c_api.cc:935: `ntree_limit` is deprecated, use `iteration_range` instead.
## [15:53:16] WARNING: src/c_api/c_api.cc:935: `ntree_limit` is deprecated, use `iteration_range` instead.
## [15:53:17] WARNING: src/c_api/c_api.cc:935: `ntree_limit` is deprecated, use `iteration_range` instead.
## [15:53:17] WARNING: src/c_api/c_api.cc:935: `ntree_limit` is deprecated, use `iteration_range` instead.
## [15:53:17] WARNING: src/c_api/c_api.cc:935: `ntree_limit` is deprecated, use `iteration_range` instead.
## [15:53:17] WARNING: src/c_api/c_api.cc:935: `ntree_limit` is deprecated, use `iteration_range` instead.
## [15:53:17] WARNING: src/c_api/c_api.cc:935: `ntree_limit` is deprecated, use `iteration_range` instead.
## [15:53:17] WARNING: src/c_api/c_api.cc:935: `ntree_limit` is deprecated, use `iteration_range` instead.
## [15:53:17] WARNING: src/c_api/c_api.cc:935: `ntree_limit` is deprecated, use `iteration_range` instead.
## [15:53:17] WARNING: src/c_api/c_api.cc:935: `ntree_limit` is deprecated, use `iteration_range` instead.
## [15:53:17] WARNING: src/c_api/c_api.cc:935: `ntree_limit` is deprecated, use `iteration_range` instead.
## [15:53:17] WARNING: src/c_api/c_api.cc:935: `ntree_limit` is deprecated, use `iteration_range` instead.
## [15:53:17] WARNING: src/c_api/c_api.cc:935: `ntree_limit` is deprecated, use `iteration_range` instead.
## [15:53:17] WARNING: src/c_api/c_api.cc:935: `ntree_limit` is deprecated, use `iteration_range` instead.
## [15:53:17] WARNING: src/c_api/c_api.cc:935: `ntree_limit` is deprecated, use `iteration_range` instead.
## [15:53:17] WARNING: src/c_api/c_api.cc:935: `ntree_limit` is deprecated, use `iteration_range` instead.
## [15:53:18] WARNING: src/c_api/c_api.cc:935: `ntree_limit` is deprecated, use `iteration_range` instead.
## [15:53:18] WARNING: src/c_api/c_api.cc:935: `ntree_limit` is deprecated, use `iteration_range` instead.
## [15:53:18] WARNING: src/c_api/c_api.cc:935: `ntree_limit` is deprecated, use `iteration_range` instead.
## [15:53:18] WARNING: src/c_api/c_api.cc:935: `ntree_limit` is deprecated, use `iteration_range` instead.
## [15:53:18] WARNING: src/c_api/c_api.cc:935: `ntree_limit` is deprecated, use `iteration_range` instead.
## [15:53:18] WARNING: src/c_api/c_api.cc:935: `ntree_limit` is deprecated, use `iteration_range` instead.
## [15:53:18] WARNING: src/c_api/c_api.cc:935: `ntree_limit` is deprecated, use `iteration_range` instead.
## [15:53:18] WARNING: src/c_api/c_api.cc:935: `ntree_limit` is deprecated, use `iteration_range` instead.
## [15:53:18] WARNING: src/c_api/c_api.cc:935: `ntree_limit` is deprecated, use `iteration_range` instead.
## [15:53:18] WARNING: src/c_api/c_api.cc:935: `ntree_limit` is deprecated, use `iteration_range` instead.
## [15:53:19] WARNING: src/c_api/c_api.cc:935: `ntree_limit` is deprecated, use `iteration_range` instead.
## [15:53:19] WARNING: src/c_api/c_api.cc:935: `ntree_limit` is deprecated, use `iteration_range` instead.
## [15:53:19] WARNING: src/c_api/c_api.cc:935: `ntree_limit` is deprecated, use `iteration_range` instead.
## [15:53:19] WARNING: src/c_api/c_api.cc:935: `ntree_limit` is deprecated, use `iteration_range` instead.
## [15:53:19] WARNING: src/c_api/c_api.cc:935: `ntree_limit` is deprecated, use `iteration_range` instead.
## [15:53:19] WARNING: src/c_api/c_api.cc:935: `ntree_limit` is deprecated, use `iteration_range` instead.
## [15:53:19] WARNING: src/c_api/c_api.cc:935: `ntree_limit` is deprecated, use `iteration_range` instead.
## [15:53:19] WARNING: src/c_api/c_api.cc:935: `ntree_limit` is deprecated, use `iteration_range` instead.
## [15:53:19] WARNING: src/c_api/c_api.cc:935: `ntree_limit` is deprecated, use `iteration_range` instead.
## [15:53:19] WARNING: src/c_api/c_api.cc:935: `ntree_limit` is deprecated, use `iteration_range` instead.
## [15:53:20] WARNING: src/c_api/c_api.cc:935: `ntree_limit` is deprecated, use `iteration_range` instead.
## [15:53:20] WARNING: src/c_api/c_api.cc:935: `ntree_limit` is deprecated, use `iteration_range` instead.
## [15:53:20] WARNING: src/c_api/c_api.cc:935: `ntree_limit` is deprecated, use `iteration_range` instead.
## [15:53:20] WARNING: src/c_api/c_api.cc:935: `ntree_limit` is deprecated, use `iteration_range` instead.
## [15:53:20] WARNING: src/c_api/c_api.cc:935: `ntree_limit` is deprecated, use `iteration_range` instead.
## [15:53:20] WARNING: src/c_api/c_api.cc:935: `ntree_limit` is deprecated, use `iteration_range` instead.
## [15:53:20] WARNING: src/c_api/c_api.cc:935: `ntree_limit` is deprecated, use `iteration_range` instead.
## [15:53:20] WARNING: src/c_api/c_api.cc:935: `ntree_limit` is deprecated, use `iteration_range` instead.
## [15:53:20] WARNING: src/c_api/c_api.cc:935: `ntree_limit` is deprecated, use `iteration_range` instead.
## [15:53:20] WARNING: src/c_api/c_api.cc:935: `ntree_limit` is deprecated, use `iteration_range` instead.
## [15:53:20] WARNING: src/c_api/c_api.cc:935: `ntree_limit` is deprecated, use `iteration_range` instead.
## [15:53:20] WARNING: src/c_api/c_api.cc:935: `ntree_limit` is deprecated, use `iteration_range` instead.
## [15:53:20] WARNING: src/c_api/c_api.cc:935: `ntree_limit` is deprecated, use `iteration_range` instead.
## [15:53:20] WARNING: src/c_api/c_api.cc:935: `ntree_limit` is deprecated, use `iteration_range` instead.
## [15:53:21] WARNING: src/c_api/c_api.cc:935: `ntree_limit` is deprecated, use `iteration_range` instead.
## [15:53:21] WARNING: src/c_api/c_api.cc:935: `ntree_limit` is deprecated, use `iteration_range` instead.
## [15:53:21] WARNING: src/c_api/c_api.cc:935: `ntree_limit` is deprecated, use `iteration_range` instead.
## [15:53:21] WARNING: src/c_api/c_api.cc:935: `ntree_limit` is deprecated, use `iteration_range` instead.
## [15:53:21] WARNING: src/c_api/c_api.cc:935: `ntree_limit` is deprecated, use `iteration_range` instead.
## [15:53:21] WARNING: src/c_api/c_api.cc:935: `ntree_limit` is deprecated, use `iteration_range` instead.
## [15:53:21] WARNING: src/c_api/c_api.cc:935: `ntree_limit` is deprecated, use `iteration_range` instead.
## [15:53:21] WARNING: src/c_api/c_api.cc:935: `ntree_limit` is deprecated, use `iteration_range` instead.
## [15:53:21] WARNING: src/c_api/c_api.cc:935: `ntree_limit` is deprecated, use `iteration_range` instead.
## [15:53:21] WARNING: src/c_api/c_api.cc:935: `ntree_limit` is deprecated, use `iteration_range` instead.
## [15:53:21] WARNING: src/c_api/c_api.cc:935: `ntree_limit` is deprecated, use `iteration_range` instead.
## [15:53:21] WARNING: src/c_api/c_api.cc:935: `ntree_limit` is deprecated, use `iteration_range` instead.
## [15:53:22] WARNING: src/c_api/c_api.cc:935: `ntree_limit` is deprecated, use `iteration_range` instead.
## [15:53:22] WARNING: src/c_api/c_api.cc:935: `ntree_limit` is deprecated, use `iteration_range` instead.
## [15:53:22] WARNING: src/c_api/c_api.cc:935: `ntree_limit` is deprecated, use `iteration_range` instead.
## [15:53:22] WARNING: src/c_api/c_api.cc:935: `ntree_limit` is deprecated, use `iteration_range` instead.
## [15:53:22] WARNING: src/c_api/c_api.cc:935: `ntree_limit` is deprecated, use `iteration_range` instead.
## [15:53:22] WARNING: src/c_api/c_api.cc:935: `ntree_limit` is deprecated, use `iteration_range` instead.
## [15:53:22] WARNING: src/c_api/c_api.cc:935: `ntree_limit` is deprecated, use `iteration_range` instead.
## [15:53:22] WARNING: src/c_api/c_api.cc:935: `ntree_limit` is deprecated, use `iteration_range` instead.
## [15:53:22] WARNING: src/c_api/c_api.cc:935: `ntree_limit` is deprecated, use `iteration_range` instead.
## [15:53:22] WARNING: src/c_api/c_api.cc:935: `ntree_limit` is deprecated, use `iteration_range` instead.
## [15:53:23] WARNING: src/c_api/c_api.cc:935: `ntree_limit` is deprecated, use `iteration_range` instead.
## [15:53:23] WARNING: src/c_api/c_api.cc:935: `ntree_limit` is deprecated, use `iteration_range` instead.
## [15:53:23] WARNING: src/c_api/c_api.cc:935: `ntree_limit` is deprecated, use `iteration_range` instead.
## [15:53:23] WARNING: src/c_api/c_api.cc:935: `ntree_limit` is deprecated, use `iteration_range` instead.
## [15:53:23] WARNING: src/c_api/c_api.cc:935: `ntree_limit` is deprecated, use `iteration_range` instead.
## [15:53:23] WARNING: src/c_api/c_api.cc:935: `ntree_limit` is deprecated, use `iteration_range` instead.
## [15:53:23] WARNING: src/c_api/c_api.cc:935: `ntree_limit` is deprecated, use `iteration_range` instead.
## [15:53:23] WARNING: src/c_api/c_api.cc:935: `ntree_limit` is deprecated, use `iteration_range` instead.
## [15:53:23] WARNING: src/c_api/c_api.cc:935: `ntree_limit` is deprecated, use `iteration_range` instead.
## [15:53:23] WARNING: src/c_api/c_api.cc:935: `ntree_limit` is deprecated, use `iteration_range` instead.
## [15:53:23] WARNING: src/c_api/c_api.cc:935: `ntree_limit` is deprecated, use `iteration_range` instead.
## [15:53:23] WARNING: src/c_api/c_api.cc:935: `ntree_limit` is deprecated, use `iteration_range` instead.
## [15:53:23] WARNING: src/c_api/c_api.cc:935: `ntree_limit` is deprecated, use `iteration_range` instead.
## [15:53:23] WARNING: src/c_api/c_api.cc:935: `ntree_limit` is deprecated, use `iteration_range` instead.
## [15:53:24] WARNING: src/c_api/c_api.cc:935: `ntree_limit` is deprecated, use `iteration_range` instead.
## [15:53:24] WARNING: src/c_api/c_api.cc:935: `ntree_limit` is deprecated, use `iteration_range` instead.
## [15:53:24] WARNING: src/c_api/c_api.cc:935: `ntree_limit` is deprecated, use `iteration_range` instead.
## [15:53:24] WARNING: src/c_api/c_api.cc:935: `ntree_limit` is deprecated, use `iteration_range` instead.
## [15:53:24] WARNING: src/c_api/c_api.cc:935: `ntree_limit` is deprecated, use `iteration_range` instead.
## [15:53:24] WARNING: src/c_api/c_api.cc:935: `ntree_limit` is deprecated, use `iteration_range` instead.
## [15:53:24] WARNING: src/c_api/c_api.cc:935: `ntree_limit` is deprecated, use `iteration_range` instead.
## [15:53:24] WARNING: src/c_api/c_api.cc:935: `ntree_limit` is deprecated, use `iteration_range` instead.
## [15:53:24] WARNING: src/c_api/c_api.cc:935: `ntree_limit` is deprecated, use `iteration_range` instead.
## [15:53:24] WARNING: src/c_api/c_api.cc:935: `ntree_limit` is deprecated, use `iteration_range` instead.
## [15:53:24] WARNING: src/c_api/c_api.cc:935: `ntree_limit` is deprecated, use `iteration_range` instead.
## [15:53:24] WARNING: src/c_api/c_api.cc:935: `ntree_limit` is deprecated, use `iteration_range` instead.
## [15:53:25] WARNING: src/c_api/c_api.cc:935: `ntree_limit` is deprecated, use `iteration_range` instead.
## [15:53:25] WARNING: src/c_api/c_api.cc:935: `ntree_limit` is deprecated, use `iteration_range` instead.
## [15:53:25] WARNING: src/c_api/c_api.cc:935: `ntree_limit` is deprecated, use `iteration_range` instead.
## [15:53:25] WARNING: src/c_api/c_api.cc:935: `ntree_limit` is deprecated, use `iteration_range` instead.
## [15:53:25] WARNING: src/c_api/c_api.cc:935: `ntree_limit` is deprecated, use `iteration_range` instead.
## [15:53:25] WARNING: src/c_api/c_api.cc:935: `ntree_limit` is deprecated, use `iteration_range` instead.
## [15:53:25] WARNING: src/c_api/c_api.cc:935: `ntree_limit` is deprecated, use `iteration_range` instead.
## [15:53:25] WARNING: src/c_api/c_api.cc:935: `ntree_limit` is deprecated, use `iteration_range` instead.
## [15:53:26] WARNING: src/c_api/c_api.cc:935: `ntree_limit` is deprecated, use `iteration_range` instead.
## [15:53:26] WARNING: src/c_api/c_api.cc:935: `ntree_limit` is deprecated, use `iteration_range` instead.
## [15:53:26] WARNING: src/c_api/c_api.cc:935: `ntree_limit` is deprecated, use `iteration_range` instead.
## [15:53:26] WARNING: src/c_api/c_api.cc:935: `ntree_limit` is deprecated, use `iteration_range` instead.
## [15:53:26] WARNING: src/c_api/c_api.cc:935: `ntree_limit` is deprecated, use `iteration_range` instead.
## [15:53:26] WARNING: src/c_api/c_api.cc:935: `ntree_limit` is deprecated, use `iteration_range` instead.
## [15:53:26] WARNING: src/c_api/c_api.cc:935: `ntree_limit` is deprecated, use `iteration_range` instead.
## [15:53:26] WARNING: src/c_api/c_api.cc:935: `ntree_limit` is deprecated, use `iteration_range` instead.
## [15:53:26] WARNING: src/c_api/c_api.cc:935: `ntree_limit` is deprecated, use `iteration_range` instead.
## [15:53:26] WARNING: src/c_api/c_api.cc:935: `ntree_limit` is deprecated, use `iteration_range` instead.
## [15:53:26] WARNING: src/c_api/c_api.cc:935: `ntree_limit` is deprecated, use `iteration_range` instead.
## [15:53:26] WARNING: src/c_api/c_api.cc:935: `ntree_limit` is deprecated, use `iteration_range` instead.
## [15:53:26] WARNING: src/c_api/c_api.cc:935: `ntree_limit` is deprecated, use `iteration_range` instead.
## [15:53:26] WARNING: src/c_api/c_api.cc:935: `ntree_limit` is deprecated, use `iteration_range` instead.
## [15:53:27] WARNING: src/c_api/c_api.cc:935: `ntree_limit` is deprecated, use `iteration_range` instead.
## [15:53:27] WARNING: src/c_api/c_api.cc:935: `ntree_limit` is deprecated, use `iteration_range` instead.
## [15:53:27] WARNING: src/c_api/c_api.cc:935: `ntree_limit` is deprecated, use `iteration_range` instead.
## [15:53:27] WARNING: src/c_api/c_api.cc:935: `ntree_limit` is deprecated, use `iteration_range` instead.
## [15:53:27] WARNING: src/c_api/c_api.cc:935: `ntree_limit` is deprecated, use `iteration_range` instead.
## [15:53:27] WARNING: src/c_api/c_api.cc:935: `ntree_limit` is deprecated, use `iteration_range` instead.
## [15:53:27] WARNING: src/c_api/c_api.cc:935: `ntree_limit` is deprecated, use `iteration_range` instead.
## [15:53:27] WARNING: src/c_api/c_api.cc:935: `ntree_limit` is deprecated, use `iteration_range` instead.
## [15:53:27] WARNING: src/c_api/c_api.cc:935: `ntree_limit` is deprecated, use `iteration_range` instead.
## [15:53:27] WARNING: src/c_api/c_api.cc:935: `ntree_limit` is deprecated, use `iteration_range` instead.
## [15:53:27] WARNING: src/c_api/c_api.cc:935: `ntree_limit` is deprecated, use `iteration_range` instead.
## [15:53:27] WARNING: src/c_api/c_api.cc:935: `ntree_limit` is deprecated, use `iteration_range` instead.
## [15:53:28] WARNING: src/c_api/c_api.cc:935: `ntree_limit` is deprecated, use `iteration_range` instead.
## [15:53:28] WARNING: src/c_api/c_api.cc:935: `ntree_limit` is deprecated, use `iteration_range` instead.
## [15:53:28] WARNING: src/c_api/c_api.cc:935: `ntree_limit` is deprecated, use `iteration_range` instead.
## [15:53:28] WARNING: src/c_api/c_api.cc:935: `ntree_limit` is deprecated, use `iteration_range` instead.
## [15:53:28] WARNING: src/c_api/c_api.cc:935: `ntree_limit` is deprecated, use `iteration_range` instead.
## [15:53:28] WARNING: src/c_api/c_api.cc:935: `ntree_limit` is deprecated, use `iteration_range` instead.
## [15:53:28] WARNING: src/c_api/c_api.cc:935: `ntree_limit` is deprecated, use `iteration_range` instead.
## [15:53:28] WARNING: src/c_api/c_api.cc:935: `ntree_limit` is deprecated, use `iteration_range` instead.
## [15:53:28] WARNING: src/c_api/c_api.cc:935: `ntree_limit` is deprecated, use `iteration_range` instead.
## [15:53:28] WARNING: src/c_api/c_api.cc:935: `ntree_limit` is deprecated, use `iteration_range` instead.
## [15:53:29] WARNING: src/c_api/c_api.cc:935: `ntree_limit` is deprecated, use `iteration_range` instead.
## [15:53:29] WARNING: src/c_api/c_api.cc:935: `ntree_limit` is deprecated, use `iteration_range` instead.
## [15:53:29] WARNING: src/c_api/c_api.cc:935: `ntree_limit` is deprecated, use `iteration_range` instead.
## [15:53:29] WARNING: src/c_api/c_api.cc:935: `ntree_limit` is deprecated, use `iteration_range` instead.
## [15:53:32] WARNING: src/c_api/c_api.cc:935: `ntree_limit` is deprecated, use `iteration_range` instead.
## [15:53:32] WARNING: src/c_api/c_api.cc:935: `ntree_limit` is deprecated, use `iteration_range` instead.
## [15:53:32] WARNING: src/c_api/c_api.cc:935: `ntree_limit` is deprecated, use `iteration_range` instead.
## [15:53:32] WARNING: src/c_api/c_api.cc:935: `ntree_limit` is deprecated, use `iteration_range` instead.
## [15:53:32] WARNING: src/c_api/c_api.cc:935: `ntree_limit` is deprecated, use `iteration_range` instead.
## [15:53:32] WARNING: src/c_api/c_api.cc:935: `ntree_limit` is deprecated, use `iteration_range` instead.
## [15:53:32] WARNING: src/c_api/c_api.cc:935: `ntree_limit` is deprecated, use `iteration_range` instead.
## [15:53:32] WARNING: src/c_api/c_api.cc:935: `ntree_limit` is deprecated, use `iteration_range` instead.
## [15:53:32] WARNING: src/c_api/c_api.cc:935: `ntree_limit` is deprecated, use `iteration_range` instead.
## [15:53:32] WARNING: src/c_api/c_api.cc:935: `ntree_limit` is deprecated, use `iteration_range` instead.
## [15:53:32] WARNING: src/c_api/c_api.cc:935: `ntree_limit` is deprecated, use `iteration_range` instead.
## [15:53:32] WARNING: src/c_api/c_api.cc:935: `ntree_limit` is deprecated, use `iteration_range` instead.
## [15:53:32] WARNING: src/c_api/c_api.cc:935: `ntree_limit` is deprecated, use `iteration_range` instead.
## [15:53:32] WARNING: src/c_api/c_api.cc:935: `ntree_limit` is deprecated, use `iteration_range` instead.
## [15:53:33] WARNING: src/c_api/c_api.cc:935: `ntree_limit` is deprecated, use `iteration_range` instead.
## [15:53:33] WARNING: src/c_api/c_api.cc:935: `ntree_limit` is deprecated, use `iteration_range` instead.
## [15:53:33] WARNING: src/c_api/c_api.cc:935: `ntree_limit` is deprecated, use `iteration_range` instead.
## [15:53:33] WARNING: src/c_api/c_api.cc:935: `ntree_limit` is deprecated, use `iteration_range` instead.
## [15:53:33] WARNING: src/c_api/c_api.cc:935: `ntree_limit` is deprecated, use `iteration_range` instead.
## [15:53:33] WARNING: src/c_api/c_api.cc:935: `ntree_limit` is deprecated, use `iteration_range` instead.
## [15:53:33] WARNING: src/c_api/c_api.cc:935: `ntree_limit` is deprecated, use `iteration_range` instead.
## [15:53:33] WARNING: src/c_api/c_api.cc:935: `ntree_limit` is deprecated, use `iteration_range` instead.
## [15:53:33] WARNING: src/c_api/c_api.cc:935: `ntree_limit` is deprecated, use `iteration_range` instead.
## [15:53:33] WARNING: src/c_api/c_api.cc:935: `ntree_limit` is deprecated, use `iteration_range` instead.
## [15:53:34] WARNING: src/c_api/c_api.cc:935: `ntree_limit` is deprecated, use `iteration_range` instead.
## [15:53:34] WARNING: src/c_api/c_api.cc:935: `ntree_limit` is deprecated, use `iteration_range` instead.
## [15:53:34] WARNING: src/c_api/c_api.cc:935: `ntree_limit` is deprecated, use `iteration_range` instead.
## [15:53:34] WARNING: src/c_api/c_api.cc:935: `ntree_limit` is deprecated, use `iteration_range` instead.
## [15:53:34] WARNING: src/c_api/c_api.cc:935: `ntree_limit` is deprecated, use `iteration_range` instead.
## [15:53:34] WARNING: src/c_api/c_api.cc:935: `ntree_limit` is deprecated, use `iteration_range` instead.
## [15:53:34] WARNING: src/c_api/c_api.cc:935: `ntree_limit` is deprecated, use `iteration_range` instead.
## [15:53:34] WARNING: src/c_api/c_api.cc:935: `ntree_limit` is deprecated, use `iteration_range` instead.
## [15:53:35] WARNING: src/c_api/c_api.cc:935: `ntree_limit` is deprecated, use `iteration_range` instead.
## [15:53:35] WARNING: src/c_api/c_api.cc:935: `ntree_limit` is deprecated, use `iteration_range` instead.
## [15:53:35] WARNING: src/c_api/c_api.cc:935: `ntree_limit` is deprecated, use `iteration_range` instead.
## [15:53:35] WARNING: src/c_api/c_api.cc:935: `ntree_limit` is deprecated, use `iteration_range` instead.
## [15:53:35] WARNING: src/c_api/c_api.cc:935: `ntree_limit` is deprecated, use `iteration_range` instead.
## [15:53:35] WARNING: src/c_api/c_api.cc:935: `ntree_limit` is deprecated, use `iteration_range` instead.
## [15:53:35] WARNING: src/c_api/c_api.cc:935: `ntree_limit` is deprecated, use `iteration_range` instead.
## [15:53:35] WARNING: src/c_api/c_api.cc:935: `ntree_limit` is deprecated, use `iteration_range` instead.
## [15:53:35] WARNING: src/c_api/c_api.cc:935: `ntree_limit` is deprecated, use `iteration_range` instead.
## [15:53:35] WARNING: src/c_api/c_api.cc:935: `ntree_limit` is deprecated, use `iteration_range` instead.
## [15:53:35] WARNING: src/c_api/c_api.cc:935: `ntree_limit` is deprecated, use `iteration_range` instead.
## [15:53:35] WARNING: src/c_api/c_api.cc:935: `ntree_limit` is deprecated, use `iteration_range` instead.
## [15:53:35] WARNING: src/c_api/c_api.cc:935: `ntree_limit` is deprecated, use `iteration_range` instead.
## [15:53:35] WARNING: src/c_api/c_api.cc:935: `ntree_limit` is deprecated, use `iteration_range` instead.
## [15:53:36] WARNING: src/c_api/c_api.cc:935: `ntree_limit` is deprecated, use `iteration_range` instead.
## [15:53:36] WARNING: src/c_api/c_api.cc:935: `ntree_limit` is deprecated, use `iteration_range` instead.
## [15:53:36] WARNING: src/c_api/c_api.cc:935: `ntree_limit` is deprecated, use `iteration_range` instead.
## [15:53:36] WARNING: src/c_api/c_api.cc:935: `ntree_limit` is deprecated, use `iteration_range` instead.
## [15:53:36] WARNING: src/c_api/c_api.cc:935: `ntree_limit` is deprecated, use `iteration_range` instead.
## [15:53:36] WARNING: src/c_api/c_api.cc:935: `ntree_limit` is deprecated, use `iteration_range` instead.
## [15:53:36] WARNING: src/c_api/c_api.cc:935: `ntree_limit` is deprecated, use `iteration_range` instead.
## [15:53:36] WARNING: src/c_api/c_api.cc:935: `ntree_limit` is deprecated, use `iteration_range` instead.
## [15:53:36] WARNING: src/c_api/c_api.cc:935: `ntree_limit` is deprecated, use `iteration_range` instead.
## [15:53:36] WARNING: src/c_api/c_api.cc:935: `ntree_limit` is deprecated, use `iteration_range` instead.
## [15:53:36] WARNING: src/c_api/c_api.cc:935: `ntree_limit` is deprecated, use `iteration_range` instead.
## [15:53:36] WARNING: src/c_api/c_api.cc:935: `ntree_limit` is deprecated, use `iteration_range` instead.
## [15:53:37] WARNING: src/c_api/c_api.cc:935: `ntree_limit` is deprecated, use `iteration_range` instead.
## [15:53:37] WARNING: src/c_api/c_api.cc:935: `ntree_limit` is deprecated, use `iteration_range` instead.
## [15:53:37] WARNING: src/c_api/c_api.cc:935: `ntree_limit` is deprecated, use `iteration_range` instead.
## [15:53:37] WARNING: src/c_api/c_api.cc:935: `ntree_limit` is deprecated, use `iteration_range` instead.
## [15:53:37] WARNING: src/c_api/c_api.cc:935: `ntree_limit` is deprecated, use `iteration_range` instead.
## [15:53:37] WARNING: src/c_api/c_api.cc:935: `ntree_limit` is deprecated, use `iteration_range` instead.
## [15:53:37] WARNING: src/c_api/c_api.cc:935: `ntree_limit` is deprecated, use `iteration_range` instead.
## [15:53:37] WARNING: src/c_api/c_api.cc:935: `ntree_limit` is deprecated, use `iteration_range` instead.
## [15:53:38] WARNING: src/c_api/c_api.cc:935: `ntree_limit` is deprecated, use `iteration_range` instead.
## [15:53:38] WARNING: src/c_api/c_api.cc:935: `ntree_limit` is deprecated, use `iteration_range` instead.
## [15:53:38] WARNING: src/c_api/c_api.cc:935: `ntree_limit` is deprecated, use `iteration_range` instead.
## [15:53:38] WARNING: src/c_api/c_api.cc:935: `ntree_limit` is deprecated, use `iteration_range` instead.
## [15:53:38] WARNING: src/c_api/c_api.cc:935: `ntree_limit` is deprecated, use `iteration_range` instead.
## [15:53:38] WARNING: src/c_api/c_api.cc:935: `ntree_limit` is deprecated, use `iteration_range` instead.
## [15:53:38] WARNING: src/c_api/c_api.cc:935: `ntree_limit` is deprecated, use `iteration_range` instead.
## [15:53:38] WARNING: src/c_api/c_api.cc:935: `ntree_limit` is deprecated, use `iteration_range` instead.
## [15:53:38] WARNING: src/c_api/c_api.cc:935: `ntree_limit` is deprecated, use `iteration_range` instead.
## [15:53:38] WARNING: src/c_api/c_api.cc:935: `ntree_limit` is deprecated, use `iteration_range` instead.
## [15:53:38] WARNING: src/c_api/c_api.cc:935: `ntree_limit` is deprecated, use `iteration_range` instead.
## [15:53:38] WARNING: src/c_api/c_api.cc:935: `ntree_limit` is deprecated, use `iteration_range` instead.
## [15:53:39] WARNING: src/c_api/c_api.cc:935: `ntree_limit` is deprecated, use `iteration_range` instead.
## [15:53:39] WARNING: src/c_api/c_api.cc:935: `ntree_limit` is deprecated, use `iteration_range` instead.
## [15:53:39] WARNING: src/c_api/c_api.cc:935: `ntree_limit` is deprecated, use `iteration_range` instead.
## [15:53:39] WARNING: src/c_api/c_api.cc:935: `ntree_limit` is deprecated, use `iteration_range` instead.
## [15:53:39] WARNING: src/c_api/c_api.cc:935: `ntree_limit` is deprecated, use `iteration_range` instead.
## [15:53:39] WARNING: src/c_api/c_api.cc:935: `ntree_limit` is deprecated, use `iteration_range` instead.
## [15:53:39] WARNING: src/c_api/c_api.cc:935: `ntree_limit` is deprecated, use `iteration_range` instead.
## [15:53:39] WARNING: src/c_api/c_api.cc:935: `ntree_limit` is deprecated, use `iteration_range` instead.
## [15:53:39] WARNING: src/c_api/c_api.cc:935: `ntree_limit` is deprecated, use `iteration_range` instead.
## [15:53:39] WARNING: src/c_api/c_api.cc:935: `ntree_limit` is deprecated, use `iteration_range` instead.
## [15:53:39] WARNING: src/c_api/c_api.cc:935: `ntree_limit` is deprecated, use `iteration_range` instead.
## [15:53:39] WARNING: src/c_api/c_api.cc:935: `ntree_limit` is deprecated, use `iteration_range` instead.
## [15:53:40] WARNING: src/c_api/c_api.cc:935: `ntree_limit` is deprecated, use `iteration_range` instead.
## [15:53:40] WARNING: src/c_api/c_api.cc:935: `ntree_limit` is deprecated, use `iteration_range` instead.
## [15:53:40] WARNING: src/c_api/c_api.cc:935: `ntree_limit` is deprecated, use `iteration_range` instead.
## [15:53:40] WARNING: src/c_api/c_api.cc:935: `ntree_limit` is deprecated, use `iteration_range` instead.
## [15:53:40] WARNING: src/c_api/c_api.cc:935: `ntree_limit` is deprecated, use `iteration_range` instead.
## [15:53:40] WARNING: src/c_api/c_api.cc:935: `ntree_limit` is deprecated, use `iteration_range` instead.
## [15:53:40] WARNING: src/c_api/c_api.cc:935: `ntree_limit` is deprecated, use `iteration_range` instead.
## [15:53:40] WARNING: src/c_api/c_api.cc:935: `ntree_limit` is deprecated, use `iteration_range` instead.
## [15:53:40] WARNING: src/c_api/c_api.cc:935: `ntree_limit` is deprecated, use `iteration_range` instead.
## [15:53:40] WARNING: src/c_api/c_api.cc:935: `ntree_limit` is deprecated, use `iteration_range` instead.
## [15:53:41] WARNING: src/c_api/c_api.cc:935: `ntree_limit` is deprecated, use `iteration_range` instead.
## [15:53:41] WARNING: src/c_api/c_api.cc:935: `ntree_limit` is deprecated, use `iteration_range` instead.
## [15:53:41] WARNING: src/c_api/c_api.cc:935: `ntree_limit` is deprecated, use `iteration_range` instead.
## [15:53:41] WARNING: src/c_api/c_api.cc:935: `ntree_limit` is deprecated, use `iteration_range` instead.
## [15:53:41] WARNING: src/c_api/c_api.cc:935: `ntree_limit` is deprecated, use `iteration_range` instead.
## [15:53:41] WARNING: src/c_api/c_api.cc:935: `ntree_limit` is deprecated, use `iteration_range` instead.
## [15:53:41] WARNING: src/c_api/c_api.cc:935: `ntree_limit` is deprecated, use `iteration_range` instead.
## [15:53:41] WARNING: src/c_api/c_api.cc:935: `ntree_limit` is deprecated, use `iteration_range` instead.
## [15:53:42] WARNING: src/c_api/c_api.cc:935: `ntree_limit` is deprecated, use `iteration_range` instead.
## [15:53:42] WARNING: src/c_api/c_api.cc:935: `ntree_limit` is deprecated, use `iteration_range` instead.
## [15:53:42] WARNING: src/c_api/c_api.cc:935: `ntree_limit` is deprecated, use `iteration_range` instead.
## [15:53:42] WARNING: src/c_api/c_api.cc:935: `ntree_limit` is deprecated, use `iteration_range` instead.
## [15:53:42] WARNING: src/c_api/c_api.cc:935: `ntree_limit` is deprecated, use `iteration_range` instead.
## [15:53:42] WARNING: src/c_api/c_api.cc:935: `ntree_limit` is deprecated, use `iteration_range` instead.
## [15:53:42] WARNING: src/c_api/c_api.cc:935: `ntree_limit` is deprecated, use `iteration_range` instead.
## [15:53:42] WARNING: src/c_api/c_api.cc:935: `ntree_limit` is deprecated, use `iteration_range` instead.
## [15:53:42] WARNING: src/c_api/c_api.cc:935: `ntree_limit` is deprecated, use `iteration_range` instead.
## [15:53:42] WARNING: src/c_api/c_api.cc:935: `ntree_limit` is deprecated, use `iteration_range` instead.
## [15:53:42] WARNING: src/c_api/c_api.cc:935: `ntree_limit` is deprecated, use `iteration_range` instead.
## [15:53:42] WARNING: src/c_api/c_api.cc:935: `ntree_limit` is deprecated, use `iteration_range` instead.
## [15:53:42] WARNING: src/c_api/c_api.cc:935: `ntree_limit` is deprecated, use `iteration_range` instead.
## [15:53:42] WARNING: src/c_api/c_api.cc:935: `ntree_limit` is deprecated, use `iteration_range` instead.
## [15:53:43] WARNING: src/c_api/c_api.cc:935: `ntree_limit` is deprecated, use `iteration_range` instead.
## [15:53:43] WARNING: src/c_api/c_api.cc:935: `ntree_limit` is deprecated, use `iteration_range` instead.
## [15:53:43] WARNING: src/c_api/c_api.cc:935: `ntree_limit` is deprecated, use `iteration_range` instead.
## [15:53:43] WARNING: src/c_api/c_api.cc:935: `ntree_limit` is deprecated, use `iteration_range` instead.
## [15:53:43] WARNING: src/c_api/c_api.cc:935: `ntree_limit` is deprecated, use `iteration_range` instead.
## [15:53:43] WARNING: src/c_api/c_api.cc:935: `ntree_limit` is deprecated, use `iteration_range` instead.
## [15:53:43] WARNING: src/c_api/c_api.cc:935: `ntree_limit` is deprecated, use `iteration_range` instead.
## [15:53:43] WARNING: src/c_api/c_api.cc:935: `ntree_limit` is deprecated, use `iteration_range` instead.
## [15:53:43] WARNING: src/c_api/c_api.cc:935: `ntree_limit` is deprecated, use `iteration_range` instead.
## [15:53:43] WARNING: src/c_api/c_api.cc:935: `ntree_limit` is deprecated, use `iteration_range` instead.
## [15:53:44] WARNING: src/c_api/c_api.cc:935: `ntree_limit` is deprecated, use `iteration_range` instead.
## [15:53:44] WARNING: src/c_api/c_api.cc:935: `ntree_limit` is deprecated, use `iteration_range` instead.
## [15:53:44] WARNING: src/c_api/c_api.cc:935: `ntree_limit` is deprecated, use `iteration_range` instead.
## [15:53:44] WARNING: src/c_api/c_api.cc:935: `ntree_limit` is deprecated, use `iteration_range` instead.
## [15:53:44] WARNING: src/c_api/c_api.cc:935: `ntree_limit` is deprecated, use `iteration_range` instead.
## [15:53:44] WARNING: src/c_api/c_api.cc:935: `ntree_limit` is deprecated, use `iteration_range` instead.
## [15:53:44] WARNING: src/c_api/c_api.cc:935: `ntree_limit` is deprecated, use `iteration_range` instead.
## [15:53:44] WARNING: src/c_api/c_api.cc:935: `ntree_limit` is deprecated, use `iteration_range` instead.
## [15:53:44] WARNING: src/c_api/c_api.cc:935: `ntree_limit` is deprecated, use `iteration_range` instead.
## [15:53:44] WARNING: src/c_api/c_api.cc:935: `ntree_limit` is deprecated, use `iteration_range` instead.
## [15:53:45] WARNING: src/c_api/c_api.cc:935: `ntree_limit` is deprecated, use `iteration_range` instead.
## [15:53:45] WARNING: src/c_api/c_api.cc:935: `ntree_limit` is deprecated, use `iteration_range` instead.
## [15:53:45] WARNING: src/c_api/c_api.cc:935: `ntree_limit` is deprecated, use `iteration_range` instead.
## [15:53:45] WARNING: src/c_api/c_api.cc:935: `ntree_limit` is deprecated, use `iteration_range` instead.
## [15:53:45] WARNING: src/c_api/c_api.cc:935: `ntree_limit` is deprecated, use `iteration_range` instead.
## [15:53:45] WARNING: src/c_api/c_api.cc:935: `ntree_limit` is deprecated, use `iteration_range` instead.
## [15:53:45] WARNING: src/c_api/c_api.cc:935: `ntree_limit` is deprecated, use `iteration_range` instead.
## [15:53:45] WARNING: src/c_api/c_api.cc:935: `ntree_limit` is deprecated, use `iteration_range` instead.
## [15:53:45] WARNING: src/c_api/c_api.cc:935: `ntree_limit` is deprecated, use `iteration_range` instead.
## [15:53:45] WARNING: src/c_api/c_api.cc:935: `ntree_limit` is deprecated, use `iteration_range` instead.
## [15:53:45] WARNING: src/c_api/c_api.cc:935: `ntree_limit` is deprecated, use `iteration_range` instead.
## [15:53:45] WARNING: src/c_api/c_api.cc:935: `ntree_limit` is deprecated, use `iteration_range` instead.
## [15:53:45] WARNING: src/c_api/c_api.cc:935: `ntree_limit` is deprecated, use `iteration_range` instead.
## [15:53:45] WARNING: src/c_api/c_api.cc:935: `ntree_limit` is deprecated, use `iteration_range` instead.
## [15:53:46] WARNING: src/c_api/c_api.cc:935: `ntree_limit` is deprecated, use `iteration_range` instead.
## [15:53:46] WARNING: src/c_api/c_api.cc:935: `ntree_limit` is deprecated, use `iteration_range` instead.
## [15:53:46] WARNING: src/c_api/c_api.cc:935: `ntree_limit` is deprecated, use `iteration_range` instead.
## [15:53:46] WARNING: src/c_api/c_api.cc:935: `ntree_limit` is deprecated, use `iteration_range` instead.
## [15:53:46] WARNING: src/c_api/c_api.cc:935: `ntree_limit` is deprecated, use `iteration_range` instead.
## [15:53:46] WARNING: src/c_api/c_api.cc:935: `ntree_limit` is deprecated, use `iteration_range` instead.
## [15:53:46] WARNING: src/c_api/c_api.cc:935: `ntree_limit` is deprecated, use `iteration_range` instead.
## [15:53:46] WARNING: src/c_api/c_api.cc:935: `ntree_limit` is deprecated, use `iteration_range` instead.
## [15:53:46] WARNING: src/c_api/c_api.cc:935: `ntree_limit` is deprecated, use `iteration_range` instead.
## [15:53:46] WARNING: src/c_api/c_api.cc:935: `ntree_limit` is deprecated, use `iteration_range` instead.
## [15:53:47] WARNING: src/c_api/c_api.cc:935: `ntree_limit` is deprecated, use `iteration_range` instead.
## [15:53:47] WARNING: src/c_api/c_api.cc:935: `ntree_limit` is deprecated, use `iteration_range` instead.
## [15:53:47] WARNING: src/c_api/c_api.cc:935: `ntree_limit` is deprecated, use `iteration_range` instead.
## [15:53:47] WARNING: src/c_api/c_api.cc:935: `ntree_limit` is deprecated, use `iteration_range` instead.
## [15:53:47] WARNING: src/c_api/c_api.cc:935: `ntree_limit` is deprecated, use `iteration_range` instead.
## [15:53:47] WARNING: src/c_api/c_api.cc:935: `ntree_limit` is deprecated, use `iteration_range` instead.
## [15:53:47] WARNING: src/c_api/c_api.cc:935: `ntree_limit` is deprecated, use `iteration_range` instead.
## [15:53:47] WARNING: src/c_api/c_api.cc:935: `ntree_limit` is deprecated, use `iteration_range` instead.
## [15:53:48] WARNING: src/c_api/c_api.cc:935: `ntree_limit` is deprecated, use `iteration_range` instead.
## [15:53:48] WARNING: src/c_api/c_api.cc:935: `ntree_limit` is deprecated, use `iteration_range` instead.
## [15:53:48] WARNING: src/c_api/c_api.cc:935: `ntree_limit` is deprecated, use `iteration_range` instead.
## [15:53:48] WARNING: src/c_api/c_api.cc:935: `ntree_limit` is deprecated, use `iteration_range` instead.
## [15:53:48] WARNING: src/c_api/c_api.cc:935: `ntree_limit` is deprecated, use `iteration_range` instead.
## [15:53:48] WARNING: src/c_api/c_api.cc:935: `ntree_limit` is deprecated, use `iteration_range` instead.
## [15:53:48] WARNING: src/c_api/c_api.cc:935: `ntree_limit` is deprecated, use `iteration_range` instead.
## [15:53:48] WARNING: src/c_api/c_api.cc:935: `ntree_limit` is deprecated, use `iteration_range` instead.
## [15:53:48] WARNING: src/c_api/c_api.cc:935: `ntree_limit` is deprecated, use `iteration_range` instead.
## [15:53:48] WARNING: src/c_api/c_api.cc:935: `ntree_limit` is deprecated, use `iteration_range` instead.
## [15:53:48] WARNING: src/c_api/c_api.cc:935: `ntree_limit` is deprecated, use `iteration_range` instead.
## [15:53:48] WARNING: src/c_api/c_api.cc:935: `ntree_limit` is deprecated, use `iteration_range` instead.
## [15:53:49] WARNING: src/c_api/c_api.cc:935: `ntree_limit` is deprecated, use `iteration_range` instead.
## [15:53:49] WARNING: src/c_api/c_api.cc:935: `ntree_limit` is deprecated, use `iteration_range` instead.
## [15:53:49] WARNING: src/c_api/c_api.cc:935: `ntree_limit` is deprecated, use `iteration_range` instead.
## [15:53:49] WARNING: src/c_api/c_api.cc:935: `ntree_limit` is deprecated, use `iteration_range` instead.
## [15:53:49] WARNING: src/c_api/c_api.cc:935: `ntree_limit` is deprecated, use `iteration_range` instead.
## [15:53:49] WARNING: src/c_api/c_api.cc:935: `ntree_limit` is deprecated, use `iteration_range` instead.
## [15:53:49] WARNING: src/c_api/c_api.cc:935: `ntree_limit` is deprecated, use `iteration_range` instead.
## [15:53:49] WARNING: src/c_api/c_api.cc:935: `ntree_limit` is deprecated, use `iteration_range` instead.
## [15:53:49] WARNING: src/c_api/c_api.cc:935: `ntree_limit` is deprecated, use `iteration_range` instead.
## [15:53:49] WARNING: src/c_api/c_api.cc:935: `ntree_limit` is deprecated, use `iteration_range` instead.
## [15:53:49] WARNING: src/c_api/c_api.cc:935: `ntree_limit` is deprecated, use `iteration_range` instead.
## [15:53:49] WARNING: src/c_api/c_api.cc:935: `ntree_limit` is deprecated, use `iteration_range` instead.
## [15:53:50] WARNING: src/c_api/c_api.cc:935: `ntree_limit` is deprecated, use `iteration_range` instead.
## [15:53:50] WARNING: src/c_api/c_api.cc:935: `ntree_limit` is deprecated, use `iteration_range` instead.
## [15:53:50] WARNING: src/c_api/c_api.cc:935: `ntree_limit` is deprecated, use `iteration_range` instead.
## [15:53:50] WARNING: src/c_api/c_api.cc:935: `ntree_limit` is deprecated, use `iteration_range` instead.
## [15:53:50] WARNING: src/c_api/c_api.cc:935: `ntree_limit` is deprecated, use `iteration_range` instead.
## [15:53:50] WARNING: src/c_api/c_api.cc:935: `ntree_limit` is deprecated, use `iteration_range` instead.
## [15:53:50] WARNING: src/c_api/c_api.cc:935: `ntree_limit` is deprecated, use `iteration_range` instead.
## [15:53:50] WARNING: src/c_api/c_api.cc:935: `ntree_limit` is deprecated, use `iteration_range` instead.
## [15:53:50] WARNING: src/c_api/c_api.cc:935: `ntree_limit` is deprecated, use `iteration_range` instead.
## [15:53:50] WARNING: src/c_api/c_api.cc:935: `ntree_limit` is deprecated, use `iteration_range` instead.
## [15:53:50] WARNING: src/c_api/c_api.cc:935: `ntree_limit` is deprecated, use `iteration_range` instead.
## [15:53:50] WARNING: src/c_api/c_api.cc:935: `ntree_limit` is deprecated, use `iteration_range` instead.
## [15:53:51] WARNING: src/c_api/c_api.cc:935: `ntree_limit` is deprecated, use `iteration_range` instead.
## [15:53:51] WARNING: src/c_api/c_api.cc:935: `ntree_limit` is deprecated, use `iteration_range` instead.
## [15:53:51] WARNING: src/c_api/c_api.cc:935: `ntree_limit` is deprecated, use `iteration_range` instead.
## [15:53:51] WARNING: src/c_api/c_api.cc:935: `ntree_limit` is deprecated, use `iteration_range` instead.
## [15:53:51] WARNING: src/c_api/c_api.cc:935: `ntree_limit` is deprecated, use `iteration_range` instead.
## [15:53:51] WARNING: src/c_api/c_api.cc:935: `ntree_limit` is deprecated, use `iteration_range` instead.
## [15:53:51] WARNING: src/c_api/c_api.cc:935: `ntree_limit` is deprecated, use `iteration_range` instead.
## [15:53:51] WARNING: src/c_api/c_api.cc:935: `ntree_limit` is deprecated, use `iteration_range` instead.
## [15:53:51] WARNING: src/c_api/c_api.cc:935: `ntree_limit` is deprecated, use `iteration_range` instead.
## [15:53:51] WARNING: src/c_api/c_api.cc:935: `ntree_limit` is deprecated, use `iteration_range` instead.
## [15:53:52] WARNING: src/c_api/c_api.cc:935: `ntree_limit` is deprecated, use `iteration_range` instead.
## [15:53:52] WARNING: src/c_api/c_api.cc:935: `ntree_limit` is deprecated, use `iteration_range` instead.
## [15:53:52] WARNING: src/c_api/c_api.cc:935: `ntree_limit` is deprecated, use `iteration_range` instead.
## [15:53:52] WARNING: src/c_api/c_api.cc:935: `ntree_limit` is deprecated, use `iteration_range` instead.
## [15:53:52] WARNING: src/c_api/c_api.cc:935: `ntree_limit` is deprecated, use `iteration_range` instead.
## [15:53:52] WARNING: src/c_api/c_api.cc:935: `ntree_limit` is deprecated, use `iteration_range` instead.
## [15:53:52] WARNING: src/c_api/c_api.cc:935: `ntree_limit` is deprecated, use `iteration_range` instead.
## [15:53:52] WARNING: src/c_api/c_api.cc:935: `ntree_limit` is deprecated, use `iteration_range` instead.
## [15:53:52] WARNING: src/c_api/c_api.cc:935: `ntree_limit` is deprecated, use `iteration_range` instead.
## [15:53:52] WARNING: src/c_api/c_api.cc:935: `ntree_limit` is deprecated, use `iteration_range` instead.
## [15:53:52] WARNING: src/c_api/c_api.cc:935: `ntree_limit` is deprecated, use `iteration_range` instead.
## [15:53:52] WARNING: src/c_api/c_api.cc:935: `ntree_limit` is deprecated, use `iteration_range` instead.
## [15:53:52] WARNING: src/c_api/c_api.cc:935: `ntree_limit` is deprecated, use `iteration_range` instead.
## [15:53:52] WARNING: src/c_api/c_api.cc:935: `ntree_limit` is deprecated, use `iteration_range` instead.
## [15:53:53] WARNING: src/c_api/c_api.cc:935: `ntree_limit` is deprecated, use `iteration_range` instead.
## [15:53:53] WARNING: src/c_api/c_api.cc:935: `ntree_limit` is deprecated, use `iteration_range` instead.
## [15:53:53] WARNING: src/c_api/c_api.cc:935: `ntree_limit` is deprecated, use `iteration_range` instead.
## [15:53:53] WARNING: src/c_api/c_api.cc:935: `ntree_limit` is deprecated, use `iteration_range` instead.
## [15:53:53] WARNING: src/c_api/c_api.cc:935: `ntree_limit` is deprecated, use `iteration_range` instead.
## [15:53:53] WARNING: src/c_api/c_api.cc:935: `ntree_limit` is deprecated, use `iteration_range` instead.
## [15:53:53] WARNING: src/c_api/c_api.cc:935: `ntree_limit` is deprecated, use `iteration_range` instead.
## [15:53:53] WARNING: src/c_api/c_api.cc:935: `ntree_limit` is deprecated, use `iteration_range` instead.
## [15:53:54] WARNING: src/c_api/c_api.cc:935: `ntree_limit` is deprecated, use `iteration_range` instead.
## [15:53:54] WARNING: src/c_api/c_api.cc:935: `ntree_limit` is deprecated, use `iteration_range` instead.
## [15:53:54] WARNING: src/c_api/c_api.cc:935: `ntree_limit` is deprecated, use `iteration_range` instead.
## [15:53:54] WARNING: src/c_api/c_api.cc:935: `ntree_limit` is deprecated, use `iteration_range` instead.
## [15:53:54] WARNING: src/c_api/c_api.cc:935: `ntree_limit` is deprecated, use `iteration_range` instead.
## [15:53:54] WARNING: src/c_api/c_api.cc:935: `ntree_limit` is deprecated, use `iteration_range` instead.
## [15:53:54] WARNING: src/c_api/c_api.cc:935: `ntree_limit` is deprecated, use `iteration_range` instead.
## [15:53:54] WARNING: src/c_api/c_api.cc:935: `ntree_limit` is deprecated, use `iteration_range` instead.
## [15:53:54] WARNING: src/c_api/c_api.cc:935: `ntree_limit` is deprecated, use `iteration_range` instead.
## [15:53:54] WARNING: src/c_api/c_api.cc:935: `ntree_limit` is deprecated, use `iteration_range` instead.
## [15:53:55] WARNING: src/c_api/c_api.cc:935: `ntree_limit` is deprecated, use `iteration_range` instead.
## [15:53:55] WARNING: src/c_api/c_api.cc:935: `ntree_limit` is deprecated, use `iteration_range` instead.
## [15:53:55] WARNING: src/c_api/c_api.cc:935: `ntree_limit` is deprecated, use `iteration_range` instead.
## [15:53:55] WARNING: src/c_api/c_api.cc:935: `ntree_limit` is deprecated, use `iteration_range` instead.
## [15:53:55] WARNING: src/c_api/c_api.cc:935: `ntree_limit` is deprecated, use `iteration_range` instead.
## [15:53:55] WARNING: src/c_api/c_api.cc:935: `ntree_limit` is deprecated, use `iteration_range` instead.
## [15:53:55] WARNING: src/c_api/c_api.cc:935: `ntree_limit` is deprecated, use `iteration_range` instead.
## [15:53:55] WARNING: src/c_api/c_api.cc:935: `ntree_limit` is deprecated, use `iteration_range` instead.
## [15:53:55] WARNING: src/c_api/c_api.cc:935: `ntree_limit` is deprecated, use `iteration_range` instead.
## [15:53:55] WARNING: src/c_api/c_api.cc:935: `ntree_limit` is deprecated, use `iteration_range` instead.
## [15:53:55] WARNING: src/c_api/c_api.cc:935: `ntree_limit` is deprecated, use `iteration_range` instead.
## [15:53:55] WARNING: src/c_api/c_api.cc:935: `ntree_limit` is deprecated, use `iteration_range` instead.
## [15:53:55] WARNING: src/c_api/c_api.cc:935: `ntree_limit` is deprecated, use `iteration_range` instead.
## [15:53:55] WARNING: src/c_api/c_api.cc:935: `ntree_limit` is deprecated, use `iteration_range` instead.
## [15:53:56] WARNING: src/c_api/c_api.cc:935: `ntree_limit` is deprecated, use `iteration_range` instead.
## [15:53:56] WARNING: src/c_api/c_api.cc:935: `ntree_limit` is deprecated, use `iteration_range` instead.
## [15:53:56] WARNING: src/c_api/c_api.cc:935: `ntree_limit` is deprecated, use `iteration_range` instead.
## [15:53:56] WARNING: src/c_api/c_api.cc:935: `ntree_limit` is deprecated, use `iteration_range` instead.
## [15:53:56] WARNING: src/c_api/c_api.cc:935: `ntree_limit` is deprecated, use `iteration_range` instead.
## [15:53:56] WARNING: src/c_api/c_api.cc:935: `ntree_limit` is deprecated, use `iteration_range` instead.
## [15:53:56] WARNING: src/c_api/c_api.cc:935: `ntree_limit` is deprecated, use `iteration_range` instead.
## [15:53:56] WARNING: src/c_api/c_api.cc:935: `ntree_limit` is deprecated, use `iteration_range` instead.
## [15:53:56] WARNING: src/c_api/c_api.cc:935: `ntree_limit` is deprecated, use `iteration_range` instead.
## [15:53:56] WARNING: src/c_api/c_api.cc:935: `ntree_limit` is deprecated, use `iteration_range` instead.
## [15:53:56] WARNING: src/c_api/c_api.cc:935: `ntree_limit` is deprecated, use `iteration_range` instead.
## [15:53:56] WARNING: src/c_api/c_api.cc:935: `ntree_limit` is deprecated, use `iteration_range` instead.
## [15:53:57] WARNING: src/c_api/c_api.cc:935: `ntree_limit` is deprecated, use `iteration_range` instead.
## [15:53:57] WARNING: src/c_api/c_api.cc:935: `ntree_limit` is deprecated, use `iteration_range` instead.
## [15:53:57] WARNING: src/c_api/c_api.cc:935: `ntree_limit` is deprecated, use `iteration_range` instead.
## [15:53:57] WARNING: src/c_api/c_api.cc:935: `ntree_limit` is deprecated, use `iteration_range` instead.
## [15:53:57] WARNING: src/c_api/c_api.cc:935: `ntree_limit` is deprecated, use `iteration_range` instead.
## [15:53:57] WARNING: src/c_api/c_api.cc:935: `ntree_limit` is deprecated, use `iteration_range` instead.
## [15:53:57] WARNING: src/c_api/c_api.cc:935: `ntree_limit` is deprecated, use `iteration_range` instead.
## [15:53:57] WARNING: src/c_api/c_api.cc:935: `ntree_limit` is deprecated, use `iteration_range` instead.
## [15:53:58] WARNING: src/c_api/c_api.cc:935: `ntree_limit` is deprecated, use `iteration_range` instead.
## [15:53:58] WARNING: src/c_api/c_api.cc:935: `ntree_limit` is deprecated, use `iteration_range` instead.
## [15:54:00] WARNING: src/c_api/c_api.cc:935: `ntree_limit` is deprecated, use `iteration_range` instead.
## [15:54:00] WARNING: src/c_api/c_api.cc:935: `ntree_limit` is deprecated, use `iteration_range` instead.
## [15:54:00] WARNING: src/c_api/c_api.cc:935: `ntree_limit` is deprecated, use `iteration_range` instead.
## [15:54:00] WARNING: src/c_api/c_api.cc:935: `ntree_limit` is deprecated, use `iteration_range` instead.
## [15:54:01] WARNING: src/c_api/c_api.cc:935: `ntree_limit` is deprecated, use `iteration_range` instead.
## [15:54:01] WARNING: src/c_api/c_api.cc:935: `ntree_limit` is deprecated, use `iteration_range` instead.
## [15:54:01] WARNING: src/c_api/c_api.cc:935: `ntree_limit` is deprecated, use `iteration_range` instead.
## [15:54:01] WARNING: src/c_api/c_api.cc:935: `ntree_limit` is deprecated, use `iteration_range` instead.
## [15:54:01] WARNING: src/c_api/c_api.cc:935: `ntree_limit` is deprecated, use `iteration_range` instead.
## [15:54:01] WARNING: src/c_api/c_api.cc:935: `ntree_limit` is deprecated, use `iteration_range` instead.
## [15:54:01] WARNING: src/c_api/c_api.cc:935: `ntree_limit` is deprecated, use `iteration_range` instead.
## [15:54:01] WARNING: src/c_api/c_api.cc:935: `ntree_limit` is deprecated, use `iteration_range` instead.
## [15:54:01] WARNING: src/c_api/c_api.cc:935: `ntree_limit` is deprecated, use `iteration_range` instead.
## [15:54:01] WARNING: src/c_api/c_api.cc:935: `ntree_limit` is deprecated, use `iteration_range` instead.
## [15:54:01] WARNING: src/c_api/c_api.cc:935: `ntree_limit` is deprecated, use `iteration_range` instead.
## [15:54:01] WARNING: src/c_api/c_api.cc:935: `ntree_limit` is deprecated, use `iteration_range` instead.
## [15:54:02] WARNING: src/c_api/c_api.cc:935: `ntree_limit` is deprecated, use `iteration_range` instead.
## [15:54:02] WARNING: src/c_api/c_api.cc:935: `ntree_limit` is deprecated, use `iteration_range` instead.
## [15:54:02] WARNING: src/c_api/c_api.cc:935: `ntree_limit` is deprecated, use `iteration_range` instead.
## [15:54:02] WARNING: src/c_api/c_api.cc:935: `ntree_limit` is deprecated, use `iteration_range` instead.
## [15:54:02] WARNING: src/c_api/c_api.cc:935: `ntree_limit` is deprecated, use `iteration_range` instead.
## [15:54:02] WARNING: src/c_api/c_api.cc:935: `ntree_limit` is deprecated, use `iteration_range` instead.
## [15:54:02] WARNING: src/c_api/c_api.cc:935: `ntree_limit` is deprecated, use `iteration_range` instead.
## [15:54:02] WARNING: src/c_api/c_api.cc:935: `ntree_limit` is deprecated, use `iteration_range` instead.
## [15:54:02] WARNING: src/c_api/c_api.cc:935: `ntree_limit` is deprecated, use `iteration_range` instead.
## [15:54:02] WARNING: src/c_api/c_api.cc:935: `ntree_limit` is deprecated, use `iteration_range` instead.
## [15:54:02] WARNING: src/c_api/c_api.cc:935: `ntree_limit` is deprecated, use `iteration_range` instead.
## [15:54:02] WARNING: src/c_api/c_api.cc:935: `ntree_limit` is deprecated, use `iteration_range` instead.
## [15:54:03] WARNING: src/c_api/c_api.cc:935: `ntree_limit` is deprecated, use `iteration_range` instead.
## [15:54:03] WARNING: src/c_api/c_api.cc:935: `ntree_limit` is deprecated, use `iteration_range` instead.
## [15:54:03] WARNING: src/c_api/c_api.cc:935: `ntree_limit` is deprecated, use `iteration_range` instead.
## [15:54:03] WARNING: src/c_api/c_api.cc:935: `ntree_limit` is deprecated, use `iteration_range` instead.
## [15:54:03] WARNING: src/c_api/c_api.cc:935: `ntree_limit` is deprecated, use `iteration_range` instead.
## [15:54:03] WARNING: src/c_api/c_api.cc:935: `ntree_limit` is deprecated, use `iteration_range` instead.
## [15:54:03] WARNING: src/c_api/c_api.cc:935: `ntree_limit` is deprecated, use `iteration_range` instead.
## [15:54:03] WARNING: src/c_api/c_api.cc:935: `ntree_limit` is deprecated, use `iteration_range` instead.
## [15:54:03] WARNING: src/c_api/c_api.cc:935: `ntree_limit` is deprecated, use `iteration_range` instead.
## [15:54:03] WARNING: src/c_api/c_api.cc:935: `ntree_limit` is deprecated, use `iteration_range` instead.
## [15:54:04] WARNING: src/c_api/c_api.cc:935: `ntree_limit` is deprecated, use `iteration_range` instead.
## [15:54:04] WARNING: src/c_api/c_api.cc:935: `ntree_limit` is deprecated, use `iteration_range` instead.
## [15:54:04] WARNING: src/c_api/c_api.cc:935: `ntree_limit` is deprecated, use `iteration_range` instead.
## [15:54:04] WARNING: src/c_api/c_api.cc:935: `ntree_limit` is deprecated, use `iteration_range` instead.
## [15:54:04] WARNING: src/c_api/c_api.cc:935: `ntree_limit` is deprecated, use `iteration_range` instead.
## [15:54:04] WARNING: src/c_api/c_api.cc:935: `ntree_limit` is deprecated, use `iteration_range` instead.
## [15:54:04] WARNING: src/c_api/c_api.cc:935: `ntree_limit` is deprecated, use `iteration_range` instead.
## [15:54:04] WARNING: src/c_api/c_api.cc:935: `ntree_limit` is deprecated, use `iteration_range` instead.
## [15:54:04] WARNING: src/c_api/c_api.cc:935: `ntree_limit` is deprecated, use `iteration_range` instead.
## [15:54:04] WARNING: src/c_api/c_api.cc:935: `ntree_limit` is deprecated, use `iteration_range` instead.
## [15:54:04] WARNING: src/c_api/c_api.cc:935: `ntree_limit` is deprecated, use `iteration_range` instead.
## [15:54:04] WARNING: src/c_api/c_api.cc:935: `ntree_limit` is deprecated, use `iteration_range` instead.
## [15:54:04] WARNING: src/c_api/c_api.cc:935: `ntree_limit` is deprecated, use `iteration_range` instead.
## [15:54:04] WARNING: src/c_api/c_api.cc:935: `ntree_limit` is deprecated, use `iteration_range` instead.
## [15:54:05] WARNING: src/c_api/c_api.cc:935: `ntree_limit` is deprecated, use `iteration_range` instead.
## [15:54:05] WARNING: src/c_api/c_api.cc:935: `ntree_limit` is deprecated, use `iteration_range` instead.
## [15:54:05] WARNING: src/c_api/c_api.cc:935: `ntree_limit` is deprecated, use `iteration_range` instead.
## [15:54:05] WARNING: src/c_api/c_api.cc:935: `ntree_limit` is deprecated, use `iteration_range` instead.
## [15:54:05] WARNING: src/c_api/c_api.cc:935: `ntree_limit` is deprecated, use `iteration_range` instead.
## [15:54:05] WARNING: src/c_api/c_api.cc:935: `ntree_limit` is deprecated, use `iteration_range` instead.
## [15:54:05] WARNING: src/c_api/c_api.cc:935: `ntree_limit` is deprecated, use `iteration_range` instead.
## [15:54:05] WARNING: src/c_api/c_api.cc:935: `ntree_limit` is deprecated, use `iteration_range` instead.
## [15:54:05] WARNING: src/c_api/c_api.cc:935: `ntree_limit` is deprecated, use `iteration_range` instead.
## [15:54:05] WARNING: src/c_api/c_api.cc:935: `ntree_limit` is deprecated, use `iteration_range` instead.
## [15:54:05] WARNING: src/c_api/c_api.cc:935: `ntree_limit` is deprecated, use `iteration_range` instead.
## [15:54:05] WARNING: src/c_api/c_api.cc:935: `ntree_limit` is deprecated, use `iteration_range` instead.
## [15:54:06] WARNING: src/c_api/c_api.cc:935: `ntree_limit` is deprecated, use `iteration_range` instead.
## [15:54:06] WARNING: src/c_api/c_api.cc:935: `ntree_limit` is deprecated, use `iteration_range` instead.
## [15:54:06] WARNING: src/c_api/c_api.cc:935: `ntree_limit` is deprecated, use `iteration_range` instead.
## [15:54:06] WARNING: src/c_api/c_api.cc:935: `ntree_limit` is deprecated, use `iteration_range` instead.
## [15:54:06] WARNING: src/c_api/c_api.cc:935: `ntree_limit` is deprecated, use `iteration_range` instead.
## [15:54:06] WARNING: src/c_api/c_api.cc:935: `ntree_limit` is deprecated, use `iteration_range` instead.
## [15:54:06] WARNING: src/c_api/c_api.cc:935: `ntree_limit` is deprecated, use `iteration_range` instead.
## [15:54:06] WARNING: src/c_api/c_api.cc:935: `ntree_limit` is deprecated, use `iteration_range` instead.
## [15:54:06] WARNING: src/c_api/c_api.cc:935: `ntree_limit` is deprecated, use `iteration_range` instead.
## [15:54:06] WARNING: src/c_api/c_api.cc:935: `ntree_limit` is deprecated, use `iteration_range` instead.
## [15:54:06] WARNING: src/c_api/c_api.cc:935: `ntree_limit` is deprecated, use `iteration_range` instead.
## [15:54:06] WARNING: src/c_api/c_api.cc:935: `ntree_limit` is deprecated, use `iteration_range` instead.
## [15:54:07] WARNING: src/c_api/c_api.cc:935: `ntree_limit` is deprecated, use `iteration_range` instead.
## [15:54:07] WARNING: src/c_api/c_api.cc:935: `ntree_limit` is deprecated, use `iteration_range` instead.
## [15:54:07] WARNING: src/c_api/c_api.cc:935: `ntree_limit` is deprecated, use `iteration_range` instead.
## [15:54:07] WARNING: src/c_api/c_api.cc:935: `ntree_limit` is deprecated, use `iteration_range` instead.
## [15:54:07] WARNING: src/c_api/c_api.cc:935: `ntree_limit` is deprecated, use `iteration_range` instead.
## [15:54:07] WARNING: src/c_api/c_api.cc:935: `ntree_limit` is deprecated, use `iteration_range` instead.
## [15:54:07] WARNING: src/c_api/c_api.cc:935: `ntree_limit` is deprecated, use `iteration_range` instead.
## [15:54:07] WARNING: src/c_api/c_api.cc:935: `ntree_limit` is deprecated, use `iteration_range` instead.
## [15:54:07] WARNING: src/c_api/c_api.cc:935: `ntree_limit` is deprecated, use `iteration_range` instead.
## [15:54:07] WARNING: src/c_api/c_api.cc:935: `ntree_limit` is deprecated, use `iteration_range` instead.
## [15:54:07] WARNING: src/c_api/c_api.cc:935: `ntree_limit` is deprecated, use `iteration_range` instead.
## [15:54:07] WARNING: src/c_api/c_api.cc:935: `ntree_limit` is deprecated, use `iteration_range` instead.
## [15:54:07] WARNING: src/c_api/c_api.cc:935: `ntree_limit` is deprecated, use `iteration_range` instead.
## [15:54:07] WARNING: src/c_api/c_api.cc:935: `ntree_limit` is deprecated, use `iteration_range` instead.
## [15:54:08] WARNING: src/c_api/c_api.cc:935: `ntree_limit` is deprecated, use `iteration_range` instead.
## [15:54:08] WARNING: src/c_api/c_api.cc:935: `ntree_limit` is deprecated, use `iteration_range` instead.
## [15:54:08] WARNING: src/c_api/c_api.cc:935: `ntree_limit` is deprecated, use `iteration_range` instead.
## [15:54:08] WARNING: src/c_api/c_api.cc:935: `ntree_limit` is deprecated, use `iteration_range` instead.
## [15:54:08] WARNING: src/c_api/c_api.cc:935: `ntree_limit` is deprecated, use `iteration_range` instead.
## [15:54:08] WARNING: src/c_api/c_api.cc:935: `ntree_limit` is deprecated, use `iteration_range` instead.
## [15:54:08] WARNING: src/c_api/c_api.cc:935: `ntree_limit` is deprecated, use `iteration_range` instead.
## [15:54:08] WARNING: src/c_api/c_api.cc:935: `ntree_limit` is deprecated, use `iteration_range` instead.
## [15:54:08] WARNING: src/c_api/c_api.cc:935: `ntree_limit` is deprecated, use `iteration_range` instead.
## [15:54:08] WARNING: src/c_api/c_api.cc:935: `ntree_limit` is deprecated, use `iteration_range` instead.
## [15:54:09] WARNING: src/c_api/c_api.cc:935: `ntree_limit` is deprecated, use `iteration_range` instead.
## [15:54:09] WARNING: src/c_api/c_api.cc:935: `ntree_limit` is deprecated, use `iteration_range` instead.
## [15:54:09] WARNING: src/c_api/c_api.cc:935: `ntree_limit` is deprecated, use `iteration_range` instead.
## [15:54:09] WARNING: src/c_api/c_api.cc:935: `ntree_limit` is deprecated, use `iteration_range` instead.
## [15:54:09] WARNING: src/c_api/c_api.cc:935: `ntree_limit` is deprecated, use `iteration_range` instead.
## [15:54:09] WARNING: src/c_api/c_api.cc:935: `ntree_limit` is deprecated, use `iteration_range` instead.
## [15:54:09] WARNING: src/c_api/c_api.cc:935: `ntree_limit` is deprecated, use `iteration_range` instead.
## [15:54:09] WARNING: src/c_api/c_api.cc:935: `ntree_limit` is deprecated, use `iteration_range` instead.
## [15:54:09] WARNING: src/c_api/c_api.cc:935: `ntree_limit` is deprecated, use `iteration_range` instead.
## [15:54:09] WARNING: src/c_api/c_api.cc:935: `ntree_limit` is deprecated, use `iteration_range` instead.
## [15:54:10] WARNING: src/c_api/c_api.cc:935: `ntree_limit` is deprecated, use `iteration_range` instead.
## [15:54:10] WARNING: src/c_api/c_api.cc:935: `ntree_limit` is deprecated, use `iteration_range` instead.
## [15:54:10] WARNING: src/c_api/c_api.cc:935: `ntree_limit` is deprecated, use `iteration_range` instead.
## [15:54:10] WARNING: src/c_api/c_api.cc:935: `ntree_limit` is deprecated, use `iteration_range` instead.
## [15:54:10] WARNING: src/c_api/c_api.cc:935: `ntree_limit` is deprecated, use `iteration_range` instead.
## [15:54:10] WARNING: src/c_api/c_api.cc:935: `ntree_limit` is deprecated, use `iteration_range` instead.
## [15:54:10] WARNING: src/c_api/c_api.cc:935: `ntree_limit` is deprecated, use `iteration_range` instead.
## [15:54:10] WARNING: src/c_api/c_api.cc:935: `ntree_limit` is deprecated, use `iteration_range` instead.
## [15:54:10] WARNING: src/c_api/c_api.cc:935: `ntree_limit` is deprecated, use `iteration_range` instead.
## [15:54:10] WARNING: src/c_api/c_api.cc:935: `ntree_limit` is deprecated, use `iteration_range` instead.
## [15:54:10] WARNING: src/c_api/c_api.cc:935: `ntree_limit` is deprecated, use `iteration_range` instead.
## [15:54:10] WARNING: src/c_api/c_api.cc:935: `ntree_limit` is deprecated, use `iteration_range` instead.
## [15:54:10] WARNING: src/c_api/c_api.cc:935: `ntree_limit` is deprecated, use `iteration_range` instead.
## [15:54:10] WARNING: src/c_api/c_api.cc:935: `ntree_limit` is deprecated, use `iteration_range` instead.
## [15:54:11] WARNING: src/c_api/c_api.cc:935: `ntree_limit` is deprecated, use `iteration_range` instead.
## [15:54:11] WARNING: src/c_api/c_api.cc:935: `ntree_limit` is deprecated, use `iteration_range` instead.
## [15:54:11] WARNING: src/c_api/c_api.cc:935: `ntree_limit` is deprecated, use `iteration_range` instead.
## [15:54:11] WARNING: src/c_api/c_api.cc:935: `ntree_limit` is deprecated, use `iteration_range` instead.
## [15:54:11] WARNING: src/c_api/c_api.cc:935: `ntree_limit` is deprecated, use `iteration_range` instead.
## [15:54:11] WARNING: src/c_api/c_api.cc:935: `ntree_limit` is deprecated, use `iteration_range` instead.
## [15:54:11] WARNING: src/c_api/c_api.cc:935: `ntree_limit` is deprecated, use `iteration_range` instead.
## [15:54:11] WARNING: src/c_api/c_api.cc:935: `ntree_limit` is deprecated, use `iteration_range` instead.
## [15:54:11] WARNING: src/c_api/c_api.cc:935: `ntree_limit` is deprecated, use `iteration_range` instead.
## [15:54:11] WARNING: src/c_api/c_api.cc:935: `ntree_limit` is deprecated, use `iteration_range` instead.
## [15:54:11] WARNING: src/c_api/c_api.cc:935: `ntree_limit` is deprecated, use `iteration_range` instead.
## [15:54:11] WARNING: src/c_api/c_api.cc:935: `ntree_limit` is deprecated, use `iteration_range` instead.
## [15:54:12] WARNING: src/c_api/c_api.cc:935: `ntree_limit` is deprecated, use `iteration_range` instead.
## [15:54:12] WARNING: src/c_api/c_api.cc:935: `ntree_limit` is deprecated, use `iteration_range` instead.
## [15:54:12] WARNING: src/c_api/c_api.cc:935: `ntree_limit` is deprecated, use `iteration_range` instead.
## [15:54:12] WARNING: src/c_api/c_api.cc:935: `ntree_limit` is deprecated, use `iteration_range` instead.
## [15:54:12] WARNING: src/c_api/c_api.cc:935: `ntree_limit` is deprecated, use `iteration_range` instead.
## [15:54:12] WARNING: src/c_api/c_api.cc:935: `ntree_limit` is deprecated, use `iteration_range` instead.
## [15:54:12] WARNING: src/c_api/c_api.cc:935: `ntree_limit` is deprecated, use `iteration_range` instead.
## [15:54:12] WARNING: src/c_api/c_api.cc:935: `ntree_limit` is deprecated, use `iteration_range` instead.
## [15:54:12] WARNING: src/c_api/c_api.cc:935: `ntree_limit` is deprecated, use `iteration_range` instead.
## [15:54:12] WARNING: src/c_api/c_api.cc:935: `ntree_limit` is deprecated, use `iteration_range` instead.
## [15:54:12] WARNING: src/c_api/c_api.cc:935: `ntree_limit` is deprecated, use `iteration_range` instead.
## [15:54:12] WARNING: src/c_api/c_api.cc:935: `ntree_limit` is deprecated, use `iteration_range` instead.
## [15:54:13] WARNING: src/c_api/c_api.cc:935: `ntree_limit` is deprecated, use `iteration_range` instead.
## [15:54:13] WARNING: src/c_api/c_api.cc:935: `ntree_limit` is deprecated, use `iteration_range` instead.
## [15:54:13] WARNING: src/c_api/c_api.cc:935: `ntree_limit` is deprecated, use `iteration_range` instead.
## [15:54:13] WARNING: src/c_api/c_api.cc:935: `ntree_limit` is deprecated, use `iteration_range` instead.
## [15:54:13] WARNING: src/c_api/c_api.cc:935: `ntree_limit` is deprecated, use `iteration_range` instead.
## [15:54:13] WARNING: src/c_api/c_api.cc:935: `ntree_limit` is deprecated, use `iteration_range` instead.
## [15:54:13] WARNING: src/c_api/c_api.cc:935: `ntree_limit` is deprecated, use `iteration_range` instead.
## [15:54:13] WARNING: src/c_api/c_api.cc:935: `ntree_limit` is deprecated, use `iteration_range` instead.
## [15:54:13] WARNING: src/c_api/c_api.cc:935: `ntree_limit` is deprecated, use `iteration_range` instead.
## [15:54:13] WARNING: src/c_api/c_api.cc:935: `ntree_limit` is deprecated, use `iteration_range` instead.
## [15:54:13] WARNING: src/c_api/c_api.cc:935: `ntree_limit` is deprecated, use `iteration_range` instead.
## [15:54:13] WARNING: src/c_api/c_api.cc:935: `ntree_limit` is deprecated, use `iteration_range` instead.
## [15:54:14] WARNING: src/c_api/c_api.cc:935: `ntree_limit` is deprecated, use `iteration_range` instead.
## [15:54:14] WARNING: src/c_api/c_api.cc:935: `ntree_limit` is deprecated, use `iteration_range` instead.
## [15:54:14] WARNING: src/c_api/c_api.cc:935: `ntree_limit` is deprecated, use `iteration_range` instead.
## [15:54:14] WARNING: src/c_api/c_api.cc:935: `ntree_limit` is deprecated, use `iteration_range` instead.
## [15:54:14] WARNING: src/c_api/c_api.cc:935: `ntree_limit` is deprecated, use `iteration_range` instead.
## [15:54:14] WARNING: src/c_api/c_api.cc:935: `ntree_limit` is deprecated, use `iteration_range` instead.
## [15:54:14] WARNING: src/c_api/c_api.cc:935: `ntree_limit` is deprecated, use `iteration_range` instead.
## [15:54:14] WARNING: src/c_api/c_api.cc:935: `ntree_limit` is deprecated, use `iteration_range` instead.
## [15:54:14] WARNING: src/c_api/c_api.cc:935: `ntree_limit` is deprecated, use `iteration_range` instead.
## [15:54:14] WARNING: src/c_api/c_api.cc:935: `ntree_limit` is deprecated, use `iteration_range` instead.
## [15:54:15] WARNING: src/c_api/c_api.cc:935: `ntree_limit` is deprecated, use `iteration_range` instead.
## [15:54:15] WARNING: src/c_api/c_api.cc:935: `ntree_limit` is deprecated, use `iteration_range` instead.
## [15:54:15] WARNING: src/c_api/c_api.cc:935: `ntree_limit` is deprecated, use `iteration_range` instead.
## [15:54:15] WARNING: src/c_api/c_api.cc:935: `ntree_limit` is deprecated, use `iteration_range` instead.
## [15:54:15] WARNING: src/c_api/c_api.cc:935: `ntree_limit` is deprecated, use `iteration_range` instead.
## [15:54:15] WARNING: src/c_api/c_api.cc:935: `ntree_limit` is deprecated, use `iteration_range` instead.
## [15:54:15] WARNING: src/c_api/c_api.cc:935: `ntree_limit` is deprecated, use `iteration_range` instead.
## [15:54:15] WARNING: src/c_api/c_api.cc:935: `ntree_limit` is deprecated, use `iteration_range` instead.
## [15:54:15] WARNING: src/c_api/c_api.cc:935: `ntree_limit` is deprecated, use `iteration_range` instead.
## [15:54:15] WARNING: src/c_api/c_api.cc:935: `ntree_limit` is deprecated, use `iteration_range` instead.
## [15:54:16] WARNING: src/c_api/c_api.cc:935: `ntree_limit` is deprecated, use `iteration_range` instead.
## [15:54:16] WARNING: src/c_api/c_api.cc:935: `ntree_limit` is deprecated, use `iteration_range` instead.
## [15:54:16] WARNING: src/c_api/c_api.cc:935: `ntree_limit` is deprecated, use `iteration_range` instead.
## [15:54:16] WARNING: src/c_api/c_api.cc:935: `ntree_limit` is deprecated, use `iteration_range` instead.
## [15:54:16] WARNING: src/c_api/c_api.cc:935: `ntree_limit` is deprecated, use `iteration_range` instead.
## [15:54:16] WARNING: src/c_api/c_api.cc:935: `ntree_limit` is deprecated, use `iteration_range` instead.
## [15:54:16] WARNING: src/c_api/c_api.cc:935: `ntree_limit` is deprecated, use `iteration_range` instead.
## [15:54:16] WARNING: src/c_api/c_api.cc:935: `ntree_limit` is deprecated, use `iteration_range` instead.
## [15:54:16] WARNING: src/c_api/c_api.cc:935: `ntree_limit` is deprecated, use `iteration_range` instead.
## [15:54:16] WARNING: src/c_api/c_api.cc:935: `ntree_limit` is deprecated, use `iteration_range` instead.
## [15:54:16] WARNING: src/c_api/c_api.cc:935: `ntree_limit` is deprecated, use `iteration_range` instead.
## [15:54:16] WARNING: src/c_api/c_api.cc:935: `ntree_limit` is deprecated, use `iteration_range` instead.
## [15:54:16] WARNING: src/c_api/c_api.cc:935: `ntree_limit` is deprecated, use `iteration_range` instead.
## [15:54:16] WARNING: src/c_api/c_api.cc:935: `ntree_limit` is deprecated, use `iteration_range` instead.
## [15:54:16] WARNING: src/c_api/c_api.cc:935: `ntree_limit` is deprecated, use `iteration_range` instead.
## [15:54:16] WARNING: src/c_api/c_api.cc:935: `ntree_limit` is deprecated, use `iteration_range` instead.
## [15:54:17] WARNING: src/c_api/c_api.cc:935: `ntree_limit` is deprecated, use `iteration_range` instead.
## [15:54:17] WARNING: src/c_api/c_api.cc:935: `ntree_limit` is deprecated, use `iteration_range` instead.
## [15:54:17] WARNING: src/c_api/c_api.cc:935: `ntree_limit` is deprecated, use `iteration_range` instead.
## [15:54:17] WARNING: src/c_api/c_api.cc:935: `ntree_limit` is deprecated, use `iteration_range` instead.
## [15:54:17] WARNING: src/c_api/c_api.cc:935: `ntree_limit` is deprecated, use `iteration_range` instead.
## [15:54:17] WARNING: src/c_api/c_api.cc:935: `ntree_limit` is deprecated, use `iteration_range` instead.
## [15:54:17] WARNING: src/c_api/c_api.cc:935: `ntree_limit` is deprecated, use `iteration_range` instead.
## [15:54:17] WARNING: src/c_api/c_api.cc:935: `ntree_limit` is deprecated, use `iteration_range` instead.
## [15:54:17] WARNING: src/c_api/c_api.cc:935: `ntree_limit` is deprecated, use `iteration_range` instead.
## [15:54:17] WARNING: src/c_api/c_api.cc:935: `ntree_limit` is deprecated, use `iteration_range` instead.
## [15:54:18] WARNING: src/c_api/c_api.cc:935: `ntree_limit` is deprecated, use `iteration_range` instead.
## [15:54:18] WARNING: src/c_api/c_api.cc:935: `ntree_limit` is deprecated, use `iteration_range` instead.
## [15:54:18] WARNING: src/c_api/c_api.cc:935: `ntree_limit` is deprecated, use `iteration_range` instead.
## [15:54:18] WARNING: src/c_api/c_api.cc:935: `ntree_limit` is deprecated, use `iteration_range` instead.
## [15:54:18] WARNING: src/c_api/c_api.cc:935: `ntree_limit` is deprecated, use `iteration_range` instead.
## [15:54:18] WARNING: src/c_api/c_api.cc:935: `ntree_limit` is deprecated, use `iteration_range` instead.
## [15:54:18] WARNING: src/c_api/c_api.cc:935: `ntree_limit` is deprecated, use `iteration_range` instead.
## [15:54:18] WARNING: src/c_api/c_api.cc:935: `ntree_limit` is deprecated, use `iteration_range` instead.
## [15:54:18] WARNING: src/c_api/c_api.cc:935: `ntree_limit` is deprecated, use `iteration_range` instead.
## [15:54:18] WARNING: src/c_api/c_api.cc:935: `ntree_limit` is deprecated, use `iteration_range` instead.
## [15:54:18] WARNING: src/c_api/c_api.cc:935: `ntree_limit` is deprecated, use `iteration_range` instead.
## [15:54:18] WARNING: src/c_api/c_api.cc:935: `ntree_limit` is deprecated, use `iteration_range` instead.
## [15:54:19] WARNING: src/c_api/c_api.cc:935: `ntree_limit` is deprecated, use `iteration_range` instead.
## [15:54:19] WARNING: src/c_api/c_api.cc:935: `ntree_limit` is deprecated, use `iteration_range` instead.
## [15:54:19] WARNING: src/c_api/c_api.cc:935: `ntree_limit` is deprecated, use `iteration_range` instead.
## [15:54:19] WARNING: src/c_api/c_api.cc:935: `ntree_limit` is deprecated, use `iteration_range` instead.
## [15:54:19] WARNING: src/c_api/c_api.cc:935: `ntree_limit` is deprecated, use `iteration_range` instead.
## [15:54:19] WARNING: src/c_api/c_api.cc:935: `ntree_limit` is deprecated, use `iteration_range` instead.
## [15:54:19] WARNING: src/c_api/c_api.cc:935: `ntree_limit` is deprecated, use `iteration_range` instead.
## [15:54:19] WARNING: src/c_api/c_api.cc:935: `ntree_limit` is deprecated, use `iteration_range` instead.
## [15:54:19] WARNING: src/c_api/c_api.cc:935: `ntree_limit` is deprecated, use `iteration_range` instead.
## [15:54:19] WARNING: src/c_api/c_api.cc:935: `ntree_limit` is deprecated, use `iteration_range` instead.
## [15:54:19] WARNING: src/c_api/c_api.cc:935: `ntree_limit` is deprecated, use `iteration_range` instead.
## [15:54:19] WARNING: src/c_api/c_api.cc:935: `ntree_limit` is deprecated, use `iteration_range` instead.
## [15:54:20] WARNING: src/c_api/c_api.cc:935: `ntree_limit` is deprecated, use `iteration_range` instead.
## [15:54:20] WARNING: src/c_api/c_api.cc:935: `ntree_limit` is deprecated, use `iteration_range` instead.
## [15:54:20] WARNING: src/c_api/c_api.cc:935: `ntree_limit` is deprecated, use `iteration_range` instead.
## [15:54:20] WARNING: src/c_api/c_api.cc:935: `ntree_limit` is deprecated, use `iteration_range` instead.
## [15:54:20] WARNING: src/c_api/c_api.cc:935: `ntree_limit` is deprecated, use `iteration_range` instead.
## [15:54:20] WARNING: src/c_api/c_api.cc:935: `ntree_limit` is deprecated, use `iteration_range` instead.
## [15:54:20] WARNING: src/c_api/c_api.cc:935: `ntree_limit` is deprecated, use `iteration_range` instead.
## [15:54:20] WARNING: src/c_api/c_api.cc:935: `ntree_limit` is deprecated, use `iteration_range` instead.
## [15:54:20] WARNING: src/c_api/c_api.cc:935: `ntree_limit` is deprecated, use `iteration_range` instead.
## [15:54:20] WARNING: src/c_api/c_api.cc:935: `ntree_limit` is deprecated, use `iteration_range` instead.
## [15:54:20] WARNING: src/c_api/c_api.cc:935: `ntree_limit` is deprecated, use `iteration_range` instead.
## [15:54:20] WARNING: src/c_api/c_api.cc:935: `ntree_limit` is deprecated, use `iteration_range` instead.
## [15:54:21] WARNING: src/c_api/c_api.cc:935: `ntree_limit` is deprecated, use `iteration_range` instead.
## [15:54:21] WARNING: src/c_api/c_api.cc:935: `ntree_limit` is deprecated, use `iteration_range` instead.
## [15:54:21] WARNING: src/c_api/c_api.cc:935: `ntree_limit` is deprecated, use `iteration_range` instead.
## [15:54:21] WARNING: src/c_api/c_api.cc:935: `ntree_limit` is deprecated, use `iteration_range` instead.
## [15:54:21] WARNING: src/c_api/c_api.cc:935: `ntree_limit` is deprecated, use `iteration_range` instead.
## [15:54:21] WARNING: src/c_api/c_api.cc:935: `ntree_limit` is deprecated, use `iteration_range` instead.
## [15:54:21] WARNING: src/c_api/c_api.cc:935: `ntree_limit` is deprecated, use `iteration_range` instead.
## [15:54:21] WARNING: src/c_api/c_api.cc:935: `ntree_limit` is deprecated, use `iteration_range` instead.
## [15:54:22] WARNING: src/c_api/c_api.cc:935: `ntree_limit` is deprecated, use `iteration_range` instead.
## [15:54:22] WARNING: src/c_api/c_api.cc:935: `ntree_limit` is deprecated, use `iteration_range` instead.
## [15:54:22] WARNING: src/c_api/c_api.cc:935: `ntree_limit` is deprecated, use `iteration_range` instead.
## [15:54:22] WARNING: src/c_api/c_api.cc:935: `ntree_limit` is deprecated, use `iteration_range` instead.
## [15:54:22] WARNING: src/c_api/c_api.cc:935: `ntree_limit` is deprecated, use `iteration_range` instead.
## [15:54:22] WARNING: src/c_api/c_api.cc:935: `ntree_limit` is deprecated, use `iteration_range` instead.
## [15:54:22] WARNING: src/c_api/c_api.cc:935: `ntree_limit` is deprecated, use `iteration_range` instead.
## [15:54:22] WARNING: src/c_api/c_api.cc:935: `ntree_limit` is deprecated, use `iteration_range` instead.
## [15:54:22] WARNING: src/c_api/c_api.cc:935: `ntree_limit` is deprecated, use `iteration_range` instead.
## [15:54:22] WARNING: src/c_api/c_api.cc:935: `ntree_limit` is deprecated, use `iteration_range` instead.
## [15:54:22] WARNING: src/c_api/c_api.cc:935: `ntree_limit` is deprecated, use `iteration_range` instead.
## [15:54:22] WARNING: src/c_api/c_api.cc:935: `ntree_limit` is deprecated, use `iteration_range` instead.
## [15:54:22] WARNING: src/c_api/c_api.cc:935: `ntree_limit` is deprecated, use `iteration_range` instead.
## [15:54:22] WARNING: src/c_api/c_api.cc:935: `ntree_limit` is deprecated, use `iteration_range` instead.
## [15:54:22] WARNING: src/c_api/c_api.cc:935: `ntree_limit` is deprecated, use `iteration_range` instead.
## [15:54:22] WARNING: src/c_api/c_api.cc:935: `ntree_limit` is deprecated, use `iteration_range` instead.
## [15:54:23] WARNING: src/c_api/c_api.cc:935: `ntree_limit` is deprecated, use `iteration_range` instead.
## [15:54:23] WARNING: src/c_api/c_api.cc:935: `ntree_limit` is deprecated, use `iteration_range` instead.
## [15:54:23] WARNING: src/c_api/c_api.cc:935: `ntree_limit` is deprecated, use `iteration_range` instead.
## [15:54:23] WARNING: src/c_api/c_api.cc:935: `ntree_limit` is deprecated, use `iteration_range` instead.
## [15:54:23] WARNING: src/c_api/c_api.cc:935: `ntree_limit` is deprecated, use `iteration_range` instead.
## [15:54:23] WARNING: src/c_api/c_api.cc:935: `ntree_limit` is deprecated, use `iteration_range` instead.
## [15:54:23] WARNING: src/c_api/c_api.cc:935: `ntree_limit` is deprecated, use `iteration_range` instead.
## [15:54:23] WARNING: src/c_api/c_api.cc:935: `ntree_limit` is deprecated, use `iteration_range` instead.
## [15:54:23] WARNING: src/c_api/c_api.cc:935: `ntree_limit` is deprecated, use `iteration_range` instead.
## [15:54:23] WARNING: src/c_api/c_api.cc:935: `ntree_limit` is deprecated, use `iteration_range` instead.
## [15:54:24] WARNING: src/c_api/c_api.cc:935: `ntree_limit` is deprecated, use `iteration_range` instead.
## [15:54:24] WARNING: src/c_api/c_api.cc:935: `ntree_limit` is deprecated, use `iteration_range` instead.
## [15:54:24] WARNING: src/c_api/c_api.cc:935: `ntree_limit` is deprecated, use `iteration_range` instead.
## [15:54:24] WARNING: src/c_api/c_api.cc:935: `ntree_limit` is deprecated, use `iteration_range` instead.
## [15:54:24] WARNING: src/c_api/c_api.cc:935: `ntree_limit` is deprecated, use `iteration_range` instead.
## [15:54:24] WARNING: src/c_api/c_api.cc:935: `ntree_limit` is deprecated, use `iteration_range` instead.
## [15:54:24] WARNING: src/c_api/c_api.cc:935: `ntree_limit` is deprecated, use `iteration_range` instead.
## [15:54:24] WARNING: src/c_api/c_api.cc:935: `ntree_limit` is deprecated, use `iteration_range` instead.
## [15:54:24] WARNING: src/c_api/c_api.cc:935: `ntree_limit` is deprecated, use `iteration_range` instead.
## [15:54:24] WARNING: src/c_api/c_api.cc:935: `ntree_limit` is deprecated, use `iteration_range` instead.
## [15:54:25] WARNING: src/c_api/c_api.cc:935: `ntree_limit` is deprecated, use `iteration_range` instead.
## [15:54:25] WARNING: src/c_api/c_api.cc:935: `ntree_limit` is deprecated, use `iteration_range` instead.
## [15:54:27] WARNING: src/c_api/c_api.cc:935: `ntree_limit` is deprecated, use `iteration_range` instead.
## [15:54:27] WARNING: src/c_api/c_api.cc:935: `ntree_limit` is deprecated, use `iteration_range` instead.
## [15:54:28] WARNING: src/c_api/c_api.cc:935: `ntree_limit` is deprecated, use `iteration_range` instead.
## [15:54:28] WARNING: src/c_api/c_api.cc:935: `ntree_limit` is deprecated, use `iteration_range` instead.
## [15:54:28] WARNING: src/c_api/c_api.cc:935: `ntree_limit` is deprecated, use `iteration_range` instead.
## [15:54:28] WARNING: src/c_api/c_api.cc:935: `ntree_limit` is deprecated, use `iteration_range` instead.
## [15:54:28] WARNING: src/c_api/c_api.cc:935: `ntree_limit` is deprecated, use `iteration_range` instead.
## [15:54:28] WARNING: src/c_api/c_api.cc:935: `ntree_limit` is deprecated, use `iteration_range` instead.
## [15:54:28] WARNING: src/c_api/c_api.cc:935: `ntree_limit` is deprecated, use `iteration_range` instead.
## [15:54:28] WARNING: src/c_api/c_api.cc:935: `ntree_limit` is deprecated, use `iteration_range` instead.
## [15:54:28] WARNING: src/c_api/c_api.cc:935: `ntree_limit` is deprecated, use `iteration_range` instead.
## [15:54:28] WARNING: src/c_api/c_api.cc:935: `ntree_limit` is deprecated, use `iteration_range` instead.
## [15:54:28] WARNING: src/c_api/c_api.cc:935: `ntree_limit` is deprecated, use `iteration_range` instead.
## [15:54:28] WARNING: src/c_api/c_api.cc:935: `ntree_limit` is deprecated, use `iteration_range` instead.
## [15:54:28] WARNING: src/c_api/c_api.cc:935: `ntree_limit` is deprecated, use `iteration_range` instead.
## [15:54:28] WARNING: src/c_api/c_api.cc:935: `ntree_limit` is deprecated, use `iteration_range` instead.
## [15:54:29] WARNING: src/c_api/c_api.cc:935: `ntree_limit` is deprecated, use `iteration_range` instead.
## [15:54:29] WARNING: src/c_api/c_api.cc:935: `ntree_limit` is deprecated, use `iteration_range` instead.
## [15:54:29] WARNING: src/c_api/c_api.cc:935: `ntree_limit` is deprecated, use `iteration_range` instead.
## [15:54:29] WARNING: src/c_api/c_api.cc:935: `ntree_limit` is deprecated, use `iteration_range` instead.
## [15:54:29] WARNING: src/c_api/c_api.cc:935: `ntree_limit` is deprecated, use `iteration_range` instead.
## [15:54:29] WARNING: src/c_api/c_api.cc:935: `ntree_limit` is deprecated, use `iteration_range` instead.
## [15:54:29] WARNING: src/c_api/c_api.cc:935: `ntree_limit` is deprecated, use `iteration_range` instead.
## [15:54:29] WARNING: src/c_api/c_api.cc:935: `ntree_limit` is deprecated, use `iteration_range` instead.
## [15:54:29] WARNING: src/c_api/c_api.cc:935: `ntree_limit` is deprecated, use `iteration_range` instead.
## [15:54:29] WARNING: src/c_api/c_api.cc:935: `ntree_limit` is deprecated, use `iteration_range` instead.
## [15:54:30] WARNING: src/c_api/c_api.cc:935: `ntree_limit` is deprecated, use `iteration_range` instead.
## [15:54:30] WARNING: src/c_api/c_api.cc:935: `ntree_limit` is deprecated, use `iteration_range` instead.
## [15:54:30] WARNING: src/c_api/c_api.cc:935: `ntree_limit` is deprecated, use `iteration_range` instead.
## [15:54:30] WARNING: src/c_api/c_api.cc:935: `ntree_limit` is deprecated, use `iteration_range` instead.
## [15:54:30] WARNING: src/c_api/c_api.cc:935: `ntree_limit` is deprecated, use `iteration_range` instead.
## [15:54:30] WARNING: src/c_api/c_api.cc:935: `ntree_limit` is deprecated, use `iteration_range` instead.
## [15:54:30] WARNING: src/c_api/c_api.cc:935: `ntree_limit` is deprecated, use `iteration_range` instead.
## [15:54:30] WARNING: src/c_api/c_api.cc:935: `ntree_limit` is deprecated, use `iteration_range` instead.
## [15:54:31] WARNING: src/c_api/c_api.cc:935: `ntree_limit` is deprecated, use `iteration_range` instead.
## [15:54:31] WARNING: src/c_api/c_api.cc:935: `ntree_limit` is deprecated, use `iteration_range` instead.
## [15:54:31] WARNING: src/c_api/c_api.cc:935: `ntree_limit` is deprecated, use `iteration_range` instead.
## [15:54:31] WARNING: src/c_api/c_api.cc:935: `ntree_limit` is deprecated, use `iteration_range` instead.
## [15:54:31] WARNING: src/c_api/c_api.cc:935: `ntree_limit` is deprecated, use `iteration_range` instead.
## [15:54:31] WARNING: src/c_api/c_api.cc:935: `ntree_limit` is deprecated, use `iteration_range` instead.
## [15:54:31] WARNING: src/c_api/c_api.cc:935: `ntree_limit` is deprecated, use `iteration_range` instead.
## [15:54:31] WARNING: src/c_api/c_api.cc:935: `ntree_limit` is deprecated, use `iteration_range` instead.
## [15:54:31] WARNING: src/c_api/c_api.cc:935: `ntree_limit` is deprecated, use `iteration_range` instead.
## [15:54:31] WARNING: src/c_api/c_api.cc:935: `ntree_limit` is deprecated, use `iteration_range` instead.
## [15:54:31] WARNING: src/c_api/c_api.cc:935: `ntree_limit` is deprecated, use `iteration_range` instead.
## [15:54:31] WARNING: src/c_api/c_api.cc:935: `ntree_limit` is deprecated, use `iteration_range` instead.
## [15:54:31] WARNING: src/c_api/c_api.cc:935: `ntree_limit` is deprecated, use `iteration_range` instead.
## [15:54:31] WARNING: src/c_api/c_api.cc:935: `ntree_limit` is deprecated, use `iteration_range` instead.
## [15:54:32] WARNING: src/c_api/c_api.cc:935: `ntree_limit` is deprecated, use `iteration_range` instead.
## [15:54:32] WARNING: src/c_api/c_api.cc:935: `ntree_limit` is deprecated, use `iteration_range` instead.
## [15:54:32] WARNING: src/c_api/c_api.cc:935: `ntree_limit` is deprecated, use `iteration_range` instead.
## [15:54:32] WARNING: src/c_api/c_api.cc:935: `ntree_limit` is deprecated, use `iteration_range` instead.
## [15:54:32] WARNING: src/c_api/c_api.cc:935: `ntree_limit` is deprecated, use `iteration_range` instead.
## [15:54:32] WARNING: src/c_api/c_api.cc:935: `ntree_limit` is deprecated, use `iteration_range` instead.
## [15:54:32] WARNING: src/c_api/c_api.cc:935: `ntree_limit` is deprecated, use `iteration_range` instead.
## [15:54:32] WARNING: src/c_api/c_api.cc:935: `ntree_limit` is deprecated, use `iteration_range` instead.
## [15:54:32] WARNING: src/c_api/c_api.cc:935: `ntree_limit` is deprecated, use `iteration_range` instead.
## [15:54:32] WARNING: src/c_api/c_api.cc:935: `ntree_limit` is deprecated, use `iteration_range` instead.
## [15:54:32] WARNING: src/c_api/c_api.cc:935: `ntree_limit` is deprecated, use `iteration_range` instead.
## [15:54:32] WARNING: src/c_api/c_api.cc:935: `ntree_limit` is deprecated, use `iteration_range` instead.
## [15:54:33] WARNING: src/c_api/c_api.cc:935: `ntree_limit` is deprecated, use `iteration_range` instead.
## [15:54:33] WARNING: src/c_api/c_api.cc:935: `ntree_limit` is deprecated, use `iteration_range` instead.
## [15:54:33] WARNING: src/c_api/c_api.cc:935: `ntree_limit` is deprecated, use `iteration_range` instead.
## [15:54:33] WARNING: src/c_api/c_api.cc:935: `ntree_limit` is deprecated, use `iteration_range` instead.
## [15:54:33] WARNING: src/c_api/c_api.cc:935: `ntree_limit` is deprecated, use `iteration_range` instead.
## [15:54:33] WARNING: src/c_api/c_api.cc:935: `ntree_limit` is deprecated, use `iteration_range` instead.
## [15:54:33] WARNING: src/c_api/c_api.cc:935: `ntree_limit` is deprecated, use `iteration_range` instead.
## [15:54:33] WARNING: src/c_api/c_api.cc:935: `ntree_limit` is deprecated, use `iteration_range` instead.
## [15:54:34] WARNING: src/c_api/c_api.cc:935: `ntree_limit` is deprecated, use `iteration_range` instead.
## [15:54:34] WARNING: src/c_api/c_api.cc:935: `ntree_limit` is deprecated, use `iteration_range` instead.
## [15:54:34] WARNING: src/c_api/c_api.cc:935: `ntree_limit` is deprecated, use `iteration_range` instead.
## [15:54:34] WARNING: src/c_api/c_api.cc:935: `ntree_limit` is deprecated, use `iteration_range` instead.
## [15:54:34] WARNING: src/c_api/c_api.cc:935: `ntree_limit` is deprecated, use `iteration_range` instead.
## [15:54:34] WARNING: src/c_api/c_api.cc:935: `ntree_limit` is deprecated, use `iteration_range` instead.
## [15:54:34] WARNING: src/c_api/c_api.cc:935: `ntree_limit` is deprecated, use `iteration_range` instead.
## [15:54:34] WARNING: src/c_api/c_api.cc:935: `ntree_limit` is deprecated, use `iteration_range` instead.
## [15:54:34] WARNING: src/c_api/c_api.cc:935: `ntree_limit` is deprecated, use `iteration_range` instead.
## [15:54:34] WARNING: src/c_api/c_api.cc:935: `ntree_limit` is deprecated, use `iteration_range` instead.
## [15:54:34] WARNING: src/c_api/c_api.cc:935: `ntree_limit` is deprecated, use `iteration_range` instead.
## [15:54:34] WARNING: src/c_api/c_api.cc:935: `ntree_limit` is deprecated, use `iteration_range` instead.
## [15:54:35] WARNING: src/c_api/c_api.cc:935: `ntree_limit` is deprecated, use `iteration_range` instead.
## [15:54:35] WARNING: src/c_api/c_api.cc:935: `ntree_limit` is deprecated, use `iteration_range` instead.
## [15:54:35] WARNING: src/c_api/c_api.cc:935: `ntree_limit` is deprecated, use `iteration_range` instead.
## [15:54:35] WARNING: src/c_api/c_api.cc:935: `ntree_limit` is deprecated, use `iteration_range` instead.
## [15:54:35] WARNING: src/c_api/c_api.cc:935: `ntree_limit` is deprecated, use `iteration_range` instead.
## [15:54:35] WARNING: src/c_api/c_api.cc:935: `ntree_limit` is deprecated, use `iteration_range` instead.
## [15:54:35] WARNING: src/c_api/c_api.cc:935: `ntree_limit` is deprecated, use `iteration_range` instead.
## [15:54:35] WARNING: src/c_api/c_api.cc:935: `ntree_limit` is deprecated, use `iteration_range` instead.
## [15:54:35] WARNING: src/c_api/c_api.cc:935: `ntree_limit` is deprecated, use `iteration_range` instead.
## [15:54:35] WARNING: src/c_api/c_api.cc:935: `ntree_limit` is deprecated, use `iteration_range` instead.
## [15:54:35] WARNING: src/c_api/c_api.cc:935: `ntree_limit` is deprecated, use `iteration_range` instead.
## [15:54:35] WARNING: src/c_api/c_api.cc:935: `ntree_limit` is deprecated, use `iteration_range` instead.
## [15:54:36] WARNING: src/c_api/c_api.cc:935: `ntree_limit` is deprecated, use `iteration_range` instead.
## [15:54:36] WARNING: src/c_api/c_api.cc:935: `ntree_limit` is deprecated, use `iteration_range` instead.
## [15:54:36] WARNING: src/c_api/c_api.cc:935: `ntree_limit` is deprecated, use `iteration_range` instead.
## [15:54:36] WARNING: src/c_api/c_api.cc:935: `ntree_limit` is deprecated, use `iteration_range` instead.
## [15:54:36] WARNING: src/c_api/c_api.cc:935: `ntree_limit` is deprecated, use `iteration_range` instead.
## [15:54:36] WARNING: src/c_api/c_api.cc:935: `ntree_limit` is deprecated, use `iteration_range` instead.
## [15:54:36] WARNING: src/c_api/c_api.cc:935: `ntree_limit` is deprecated, use `iteration_range` instead.
## [15:54:36] WARNING: src/c_api/c_api.cc:935: `ntree_limit` is deprecated, use `iteration_range` instead.
## [15:54:37] WARNING: src/c_api/c_api.cc:935: `ntree_limit` is deprecated, use `iteration_range` instead.
## [15:54:37] WARNING: src/c_api/c_api.cc:935: `ntree_limit` is deprecated, use `iteration_range` instead.
## [15:54:37] WARNING: src/c_api/c_api.cc:935: `ntree_limit` is deprecated, use `iteration_range` instead.
## [15:54:37] WARNING: src/c_api/c_api.cc:935: `ntree_limit` is deprecated, use `iteration_range` instead.
## [15:54:37] WARNING: src/c_api/c_api.cc:935: `ntree_limit` is deprecated, use `iteration_range` instead.
## [15:54:37] WARNING: src/c_api/c_api.cc:935: `ntree_limit` is deprecated, use `iteration_range` instead.
## [15:54:37] WARNING: src/c_api/c_api.cc:935: `ntree_limit` is deprecated, use `iteration_range` instead.
## [15:54:37] WARNING: src/c_api/c_api.cc:935: `ntree_limit` is deprecated, use `iteration_range` instead.
## [15:54:37] WARNING: src/c_api/c_api.cc:935: `ntree_limit` is deprecated, use `iteration_range` instead.
## [15:54:37] WARNING: src/c_api/c_api.cc:935: `ntree_limit` is deprecated, use `iteration_range` instead.
## [15:54:38] WARNING: src/c_api/c_api.cc:935: `ntree_limit` is deprecated, use `iteration_range` instead.
## [15:54:38] WARNING: src/c_api/c_api.cc:935: `ntree_limit` is deprecated, use `iteration_range` instead.
## [15:54:38] WARNING: src/c_api/c_api.cc:935: `ntree_limit` is deprecated, use `iteration_range` instead.
## [15:54:38] WARNING: src/c_api/c_api.cc:935: `ntree_limit` is deprecated, use `iteration_range` instead.
## [15:54:38] WARNING: src/c_api/c_api.cc:935: `ntree_limit` is deprecated, use `iteration_range` instead.
## [15:54:38] WARNING: src/c_api/c_api.cc:935: `ntree_limit` is deprecated, use `iteration_range` instead.
## [15:54:38] WARNING: src/c_api/c_api.cc:935: `ntree_limit` is deprecated, use `iteration_range` instead.
## [15:54:38] WARNING: src/c_api/c_api.cc:935: `ntree_limit` is deprecated, use `iteration_range` instead.
## [15:54:38] WARNING: src/c_api/c_api.cc:935: `ntree_limit` is deprecated, use `iteration_range` instead.
## [15:54:38] WARNING: src/c_api/c_api.cc:935: `ntree_limit` is deprecated, use `iteration_range` instead.
## [15:54:38] WARNING: src/c_api/c_api.cc:935: `ntree_limit` is deprecated, use `iteration_range` instead.
## [15:54:38] WARNING: src/c_api/c_api.cc:935: `ntree_limit` is deprecated, use `iteration_range` instead.
## [15:54:38] WARNING: src/c_api/c_api.cc:935: `ntree_limit` is deprecated, use `iteration_range` instead.
## [15:54:38] WARNING: src/c_api/c_api.cc:935: `ntree_limit` is deprecated, use `iteration_range` instead.
## [15:54:39] WARNING: src/c_api/c_api.cc:935: `ntree_limit` is deprecated, use `iteration_range` instead.
## [15:54:39] WARNING: src/c_api/c_api.cc:935: `ntree_limit` is deprecated, use `iteration_range` instead.
## [15:54:39] WARNING: src/c_api/c_api.cc:935: `ntree_limit` is deprecated, use `iteration_range` instead.
## [15:54:39] WARNING: src/c_api/c_api.cc:935: `ntree_limit` is deprecated, use `iteration_range` instead.
## [15:54:39] WARNING: src/c_api/c_api.cc:935: `ntree_limit` is deprecated, use `iteration_range` instead.
## [15:54:39] WARNING: src/c_api/c_api.cc:935: `ntree_limit` is deprecated, use `iteration_range` instead.
## [15:54:39] WARNING: src/c_api/c_api.cc:935: `ntree_limit` is deprecated, use `iteration_range` instead.
## [15:54:39] WARNING: src/c_api/c_api.cc:935: `ntree_limit` is deprecated, use `iteration_range` instead.
## [15:54:39] WARNING: src/c_api/c_api.cc:935: `ntree_limit` is deprecated, use `iteration_range` instead.
## [15:54:39] WARNING: src/c_api/c_api.cc:935: `ntree_limit` is deprecated, use `iteration_range` instead.
## [15:54:40] WARNING: src/c_api/c_api.cc:935: `ntree_limit` is deprecated, use `iteration_range` instead.
## [15:54:40] WARNING: src/c_api/c_api.cc:935: `ntree_limit` is deprecated, use `iteration_range` instead.
## [15:54:40] WARNING: src/c_api/c_api.cc:935: `ntree_limit` is deprecated, use `iteration_range` instead.
## [15:54:40] WARNING: src/c_api/c_api.cc:935: `ntree_limit` is deprecated, use `iteration_range` instead.
## [15:54:40] WARNING: src/c_api/c_api.cc:935: `ntree_limit` is deprecated, use `iteration_range` instead.
## [15:54:40] WARNING: src/c_api/c_api.cc:935: `ntree_limit` is deprecated, use `iteration_range` instead.
## [15:54:40] WARNING: src/c_api/c_api.cc:935: `ntree_limit` is deprecated, use `iteration_range` instead.
## [15:54:40] WARNING: src/c_api/c_api.cc:935: `ntree_limit` is deprecated, use `iteration_range` instead.
## [15:54:41] WARNING: src/c_api/c_api.cc:935: `ntree_limit` is deprecated, use `iteration_range` instead.
## [15:54:41] WARNING: src/c_api/c_api.cc:935: `ntree_limit` is deprecated, use `iteration_range` instead.
## [15:54:41] WARNING: src/c_api/c_api.cc:935: `ntree_limit` is deprecated, use `iteration_range` instead.
## [15:54:41] WARNING: src/c_api/c_api.cc:935: `ntree_limit` is deprecated, use `iteration_range` instead.
## [15:54:41] WARNING: src/c_api/c_api.cc:935: `ntree_limit` is deprecated, use `iteration_range` instead.
## [15:54:41] WARNING: src/c_api/c_api.cc:935: `ntree_limit` is deprecated, use `iteration_range` instead.
## [15:54:41] WARNING: src/c_api/c_api.cc:935: `ntree_limit` is deprecated, use `iteration_range` instead.
## [15:54:41] WARNING: src/c_api/c_api.cc:935: `ntree_limit` is deprecated, use `iteration_range` instead.
## [15:54:41] WARNING: src/c_api/c_api.cc:935: `ntree_limit` is deprecated, use `iteration_range` instead.
## [15:54:41] WARNING: src/c_api/c_api.cc:935: `ntree_limit` is deprecated, use `iteration_range` instead.
## [15:54:41] WARNING: src/c_api/c_api.cc:935: `ntree_limit` is deprecated, use `iteration_range` instead.
## [15:54:41] WARNING: src/c_api/c_api.cc:935: `ntree_limit` is deprecated, use `iteration_range` instead.
## [15:54:41] WARNING: src/c_api/c_api.cc:935: `ntree_limit` is deprecated, use `iteration_range` instead.
## [15:54:41] WARNING: src/c_api/c_api.cc:935: `ntree_limit` is deprecated, use `iteration_range` instead.
## [15:54:42] WARNING: src/c_api/c_api.cc:935: `ntree_limit` is deprecated, use `iteration_range` instead.
## [15:54:42] WARNING: src/c_api/c_api.cc:935: `ntree_limit` is deprecated, use `iteration_range` instead.
## [15:54:42] WARNING: src/c_api/c_api.cc:935: `ntree_limit` is deprecated, use `iteration_range` instead.
## [15:54:42] WARNING: src/c_api/c_api.cc:935: `ntree_limit` is deprecated, use `iteration_range` instead.
## [15:54:42] WARNING: src/c_api/c_api.cc:935: `ntree_limit` is deprecated, use `iteration_range` instead.
## [15:54:42] WARNING: src/c_api/c_api.cc:935: `ntree_limit` is deprecated, use `iteration_range` instead.
## [15:54:42] WARNING: src/c_api/c_api.cc:935: `ntree_limit` is deprecated, use `iteration_range` instead.
## [15:54:42] WARNING: src/c_api/c_api.cc:935: `ntree_limit` is deprecated, use `iteration_range` instead.
## [15:54:42] WARNING: src/c_api/c_api.cc:935: `ntree_limit` is deprecated, use `iteration_range` instead.
## [15:54:42] WARNING: src/c_api/c_api.cc:935: `ntree_limit` is deprecated, use `iteration_range` instead.
## [15:54:43] WARNING: src/c_api/c_api.cc:935: `ntree_limit` is deprecated, use `iteration_range` instead.
## [15:54:43] WARNING: src/c_api/c_api.cc:935: `ntree_limit` is deprecated, use `iteration_range` instead.
## [15:54:43] WARNING: src/c_api/c_api.cc:935: `ntree_limit` is deprecated, use `iteration_range` instead.
## [15:54:43] WARNING: src/c_api/c_api.cc:935: `ntree_limit` is deprecated, use `iteration_range` instead.
## [15:54:43] WARNING: src/c_api/c_api.cc:935: `ntree_limit` is deprecated, use `iteration_range` instead.
## [15:54:43] WARNING: src/c_api/c_api.cc:935: `ntree_limit` is deprecated, use `iteration_range` instead.
## [15:54:43] WARNING: src/c_api/c_api.cc:935: `ntree_limit` is deprecated, use `iteration_range` instead.
## [15:54:43] WARNING: src/c_api/c_api.cc:935: `ntree_limit` is deprecated, use `iteration_range` instead.
## [15:54:44] WARNING: src/c_api/c_api.cc:935: `ntree_limit` is deprecated, use `iteration_range` instead.
## [15:54:44] WARNING: src/c_api/c_api.cc:935: `ntree_limit` is deprecated, use `iteration_range` instead.
## [15:54:44] WARNING: src/c_api/c_api.cc:935: `ntree_limit` is deprecated, use `iteration_range` instead.
## [15:54:44] WARNING: src/c_api/c_api.cc:935: `ntree_limit` is deprecated, use `iteration_range` instead.
## [15:54:44] WARNING: src/c_api/c_api.cc:935: `ntree_limit` is deprecated, use `iteration_range` instead.
## [15:54:44] WARNING: src/c_api/c_api.cc:935: `ntree_limit` is deprecated, use `iteration_range` instead.
## [15:54:44] WARNING: src/c_api/c_api.cc:935: `ntree_limit` is deprecated, use `iteration_range` instead.
## [15:54:44] WARNING: src/c_api/c_api.cc:935: `ntree_limit` is deprecated, use `iteration_range` instead.
## [15:54:44] WARNING: src/c_api/c_api.cc:935: `ntree_limit` is deprecated, use `iteration_range` instead.
## [15:54:44] WARNING: src/c_api/c_api.cc:935: `ntree_limit` is deprecated, use `iteration_range` instead.
## [15:54:44] WARNING: src/c_api/c_api.cc:935: `ntree_limit` is deprecated, use `iteration_range` instead.
## [15:54:44] WARNING: src/c_api/c_api.cc:935: `ntree_limit` is deprecated, use `iteration_range` instead.
## [15:54:44] WARNING: src/c_api/c_api.cc:935: `ntree_limit` is deprecated, use `iteration_range` instead.
## [15:54:44] WARNING: src/c_api/c_api.cc:935: `ntree_limit` is deprecated, use `iteration_range` instead.
## [15:54:45] WARNING: src/c_api/c_api.cc:935: `ntree_limit` is deprecated, use `iteration_range` instead.
## [15:54:45] WARNING: src/c_api/c_api.cc:935: `ntree_limit` is deprecated, use `iteration_range` instead.
## [15:54:45] WARNING: src/c_api/c_api.cc:935: `ntree_limit` is deprecated, use `iteration_range` instead.
## [15:54:45] WARNING: src/c_api/c_api.cc:935: `ntree_limit` is deprecated, use `iteration_range` instead.
## [15:54:45] WARNING: src/c_api/c_api.cc:935: `ntree_limit` is deprecated, use `iteration_range` instead.
## [15:54:45] WARNING: src/c_api/c_api.cc:935: `ntree_limit` is deprecated, use `iteration_range` instead.
## [15:54:45] WARNING: src/c_api/c_api.cc:935: `ntree_limit` is deprecated, use `iteration_range` instead.
## [15:54:45] WARNING: src/c_api/c_api.cc:935: `ntree_limit` is deprecated, use `iteration_range` instead.
## [15:54:45] WARNING: src/c_api/c_api.cc:935: `ntree_limit` is deprecated, use `iteration_range` instead.
## [15:54:45] WARNING: src/c_api/c_api.cc:935: `ntree_limit` is deprecated, use `iteration_range` instead.
## [15:54:46] WARNING: src/c_api/c_api.cc:935: `ntree_limit` is deprecated, use `iteration_range` instead.
## [15:54:46] WARNING: src/c_api/c_api.cc:935: `ntree_limit` is deprecated, use `iteration_range` instead.
## [15:54:46] WARNING: src/c_api/c_api.cc:935: `ntree_limit` is deprecated, use `iteration_range` instead.
## [15:54:46] WARNING: src/c_api/c_api.cc:935: `ntree_limit` is deprecated, use `iteration_range` instead.
## [15:54:46] WARNING: src/c_api/c_api.cc:935: `ntree_limit` is deprecated, use `iteration_range` instead.
## [15:54:46] WARNING: src/c_api/c_api.cc:935: `ntree_limit` is deprecated, use `iteration_range` instead.
## [15:54:46] WARNING: src/c_api/c_api.cc:935: `ntree_limit` is deprecated, use `iteration_range` instead.
## [15:54:46] WARNING: src/c_api/c_api.cc:935: `ntree_limit` is deprecated, use `iteration_range` instead.
## [15:54:46] WARNING: src/c_api/c_api.cc:935: `ntree_limit` is deprecated, use `iteration_range` instead.
## [15:54:46] WARNING: src/c_api/c_api.cc:935: `ntree_limit` is deprecated, use `iteration_range` instead.
## [15:54:46] WARNING: src/c_api/c_api.cc:935: `ntree_limit` is deprecated, use `iteration_range` instead.
## [15:54:46] WARNING: src/c_api/c_api.cc:935: `ntree_limit` is deprecated, use `iteration_range` instead.
## [15:54:47] WARNING: src/c_api/c_api.cc:935: `ntree_limit` is deprecated, use `iteration_range` instead.
## [15:54:47] WARNING: src/c_api/c_api.cc:935: `ntree_limit` is deprecated, use `iteration_range` instead.
## [15:54:47] WARNING: src/c_api/c_api.cc:935: `ntree_limit` is deprecated, use `iteration_range` instead.
## [15:54:47] WARNING: src/c_api/c_api.cc:935: `ntree_limit` is deprecated, use `iteration_range` instead.
## [15:54:47] WARNING: src/c_api/c_api.cc:935: `ntree_limit` is deprecated, use `iteration_range` instead.
## [15:54:47] WARNING: src/c_api/c_api.cc:935: `ntree_limit` is deprecated, use `iteration_range` instead.
## [15:54:47] WARNING: src/c_api/c_api.cc:935: `ntree_limit` is deprecated, use `iteration_range` instead.
## [15:54:47] WARNING: src/c_api/c_api.cc:935: `ntree_limit` is deprecated, use `iteration_range` instead.
## [15:54:47] WARNING: src/c_api/c_api.cc:935: `ntree_limit` is deprecated, use `iteration_range` instead.
## [15:54:47] WARNING: src/c_api/c_api.cc:935: `ntree_limit` is deprecated, use `iteration_range` instead.
## [15:54:48] WARNING: src/c_api/c_api.cc:935: `ntree_limit` is deprecated, use `iteration_range` instead.
## [15:54:48] WARNING: src/c_api/c_api.cc:935: `ntree_limit` is deprecated, use `iteration_range` instead.
## [15:54:48] WARNING: src/c_api/c_api.cc:935: `ntree_limit` is deprecated, use `iteration_range` instead.
## [15:54:48] WARNING: src/c_api/c_api.cc:935: `ntree_limit` is deprecated, use `iteration_range` instead.
## [15:54:48] WARNING: src/c_api/c_api.cc:935: `ntree_limit` is deprecated, use `iteration_range` instead.
## [15:54:48] WARNING: src/c_api/c_api.cc:935: `ntree_limit` is deprecated, use `iteration_range` instead.
## [15:54:48] WARNING: src/c_api/c_api.cc:935: `ntree_limit` is deprecated, use `iteration_range` instead.
## [15:54:48] WARNING: src/c_api/c_api.cc:935: `ntree_limit` is deprecated, use `iteration_range` instead.
## [15:54:48] WARNING: src/c_api/c_api.cc:935: `ntree_limit` is deprecated, use `iteration_range` instead.
## [15:54:48] WARNING: src/c_api/c_api.cc:935: `ntree_limit` is deprecated, use `iteration_range` instead.
## [15:54:48] WARNING: src/c_api/c_api.cc:935: `ntree_limit` is deprecated, use `iteration_range` instead.
## [15:54:48] WARNING: src/c_api/c_api.cc:935: `ntree_limit` is deprecated, use `iteration_range` instead.
## [15:54:48] WARNING: src/c_api/c_api.cc:935: `ntree_limit` is deprecated, use `iteration_range` instead.
## [15:54:48] WARNING: src/c_api/c_api.cc:935: `ntree_limit` is deprecated, use `iteration_range` instead.
## [15:54:49] WARNING: src/c_api/c_api.cc:935: `ntree_limit` is deprecated, use `iteration_range` instead.
## [15:54:49] WARNING: src/c_api/c_api.cc:935: `ntree_limit` is deprecated, use `iteration_range` instead.
## [15:54:49] WARNING: src/c_api/c_api.cc:935: `ntree_limit` is deprecated, use `iteration_range` instead.
## [15:54:49] WARNING: src/c_api/c_api.cc:935: `ntree_limit` is deprecated, use `iteration_range` instead.
## [15:54:49] WARNING: src/c_api/c_api.cc:935: `ntree_limit` is deprecated, use `iteration_range` instead.
## [15:54:49] WARNING: src/c_api/c_api.cc:935: `ntree_limit` is deprecated, use `iteration_range` instead.
## [15:54:49] WARNING: src/c_api/c_api.cc:935: `ntree_limit` is deprecated, use `iteration_range` instead.
## [15:54:49] WARNING: src/c_api/c_api.cc:935: `ntree_limit` is deprecated, use `iteration_range` instead.
## [15:54:50] WARNING: src/c_api/c_api.cc:935: `ntree_limit` is deprecated, use `iteration_range` instead.
## [15:54:50] WARNING: src/c_api/c_api.cc:935: `ntree_limit` is deprecated, use `iteration_range` instead.
## [15:54:50] WARNING: src/c_api/c_api.cc:935: `ntree_limit` is deprecated, use `iteration_range` instead.
## [15:54:50] WARNING: src/c_api/c_api.cc:935: `ntree_limit` is deprecated, use `iteration_range` instead.
## [15:54:50] WARNING: src/c_api/c_api.cc:935: `ntree_limit` is deprecated, use `iteration_range` instead.
## [15:54:50] WARNING: src/c_api/c_api.cc:935: `ntree_limit` is deprecated, use `iteration_range` instead.
## [15:54:50] WARNING: src/c_api/c_api.cc:935: `ntree_limit` is deprecated, use `iteration_range` instead.
## [15:54:50] WARNING: src/c_api/c_api.cc:935: `ntree_limit` is deprecated, use `iteration_range` instead.
## [15:54:50] WARNING: src/c_api/c_api.cc:935: `ntree_limit` is deprecated, use `iteration_range` instead.
## [15:54:50] WARNING: src/c_api/c_api.cc:935: `ntree_limit` is deprecated, use `iteration_range` instead.
## [15:54:51] WARNING: src/c_api/c_api.cc:935: `ntree_limit` is deprecated, use `iteration_range` instead.
## [15:54:51] WARNING: src/c_api/c_api.cc:935: `ntree_limit` is deprecated, use `iteration_range` instead.
## [15:54:51] WARNING: src/c_api/c_api.cc:935: `ntree_limit` is deprecated, use `iteration_range` instead.
## [15:54:51] WARNING: src/c_api/c_api.cc:935: `ntree_limit` is deprecated, use `iteration_range` instead.
## [15:54:51] WARNING: src/c_api/c_api.cc:935: `ntree_limit` is deprecated, use `iteration_range` instead.
## [15:54:51] WARNING: src/c_api/c_api.cc:935: `ntree_limit` is deprecated, use `iteration_range` instead.
## [15:54:51] WARNING: src/c_api/c_api.cc:935: `ntree_limit` is deprecated, use `iteration_range` instead.
## [15:54:51] WARNING: src/c_api/c_api.cc:935: `ntree_limit` is deprecated, use `iteration_range` instead.
## [15:54:51] WARNING: src/c_api/c_api.cc:935: `ntree_limit` is deprecated, use `iteration_range` instead.
## [15:54:51] WARNING: src/c_api/c_api.cc:935: `ntree_limit` is deprecated, use `iteration_range` instead.
## [15:54:51] WARNING: src/c_api/c_api.cc:935: `ntree_limit` is deprecated, use `iteration_range` instead.
## [15:54:51] WARNING: src/c_api/c_api.cc:935: `ntree_limit` is deprecated, use `iteration_range` instead.
## [15:54:51] WARNING: src/c_api/c_api.cc:935: `ntree_limit` is deprecated, use `iteration_range` instead.
## [15:54:51] WARNING: src/c_api/c_api.cc:935: `ntree_limit` is deprecated, use `iteration_range` instead.
## [15:54:52] WARNING: src/c_api/c_api.cc:935: `ntree_limit` is deprecated, use `iteration_range` instead.
## [15:54:52] WARNING: src/c_api/c_api.cc:935: `ntree_limit` is deprecated, use `iteration_range` instead.
## [15:54:52] WARNING: src/c_api/c_api.cc:935: `ntree_limit` is deprecated, use `iteration_range` instead.
## [15:54:52] WARNING: src/c_api/c_api.cc:935: `ntree_limit` is deprecated, use `iteration_range` instead.
## [15:54:52] WARNING: src/c_api/c_api.cc:935: `ntree_limit` is deprecated, use `iteration_range` instead.
## [15:54:52] WARNING: src/c_api/c_api.cc:935: `ntree_limit` is deprecated, use `iteration_range` instead.
## [15:54:52] WARNING: src/c_api/c_api.cc:935: `ntree_limit` is deprecated, use `iteration_range` instead.
## [15:54:52] WARNING: src/c_api/c_api.cc:935: `ntree_limit` is deprecated, use `iteration_range` instead.
## [15:54:52] WARNING: src/c_api/c_api.cc:935: `ntree_limit` is deprecated, use `iteration_range` instead.
## [15:54:52] WARNING: src/c_api/c_api.cc:935: `ntree_limit` is deprecated, use `iteration_range` instead.
## [15:54:53] WARNING: src/c_api/c_api.cc:935: `ntree_limit` is deprecated, use `iteration_range` instead.
## [15:54:53] WARNING: src/c_api/c_api.cc:935: `ntree_limit` is deprecated, use `iteration_range` instead.
## [15:54:53] WARNING: src/c_api/c_api.cc:935: `ntree_limit` is deprecated, use `iteration_range` instead.
## [15:54:53] WARNING: src/c_api/c_api.cc:935: `ntree_limit` is deprecated, use `iteration_range` instead.
## [15:54:53] WARNING: src/c_api/c_api.cc:935: `ntree_limit` is deprecated, use `iteration_range` instead.
## [15:54:53] WARNING: src/c_api/c_api.cc:935: `ntree_limit` is deprecated, use `iteration_range` instead.
## [15:54:53] WARNING: src/c_api/c_api.cc:935: `ntree_limit` is deprecated, use `iteration_range` instead.
## [15:54:53] WARNING: src/c_api/c_api.cc:935: `ntree_limit` is deprecated, use `iteration_range` instead.
## [15:54:53] WARNING: src/c_api/c_api.cc:935: `ntree_limit` is deprecated, use `iteration_range` instead.
## [15:54:53] WARNING: src/c_api/c_api.cc:935: `ntree_limit` is deprecated, use `iteration_range` instead.
## [15:54:54] WARNING: src/c_api/c_api.cc:935: `ntree_limit` is deprecated, use `iteration_range` instead.
## [15:54:54] WARNING: src/c_api/c_api.cc:935: `ntree_limit` is deprecated, use `iteration_range` instead.
## [15:54:56] WARNING: src/c_api/c_api.cc:935: `ntree_limit` is deprecated, use `iteration_range` instead.
## [15:54:56] WARNING: src/c_api/c_api.cc:935: `ntree_limit` is deprecated, use `iteration_range` instead.
## [15:54:56] WARNING: src/c_api/c_api.cc:935: `ntree_limit` is deprecated, use `iteration_range` instead.
## [15:54:56] WARNING: src/c_api/c_api.cc:935: `ntree_limit` is deprecated, use `iteration_range` instead.
## [15:54:57] WARNING: src/c_api/c_api.cc:935: `ntree_limit` is deprecated, use `iteration_range` instead.
## [15:54:57] WARNING: src/c_api/c_api.cc:935: `ntree_limit` is deprecated, use `iteration_range` instead.
## [15:54:57] WARNING: src/c_api/c_api.cc:935: `ntree_limit` is deprecated, use `iteration_range` instead.
## [15:54:57] WARNING: src/c_api/c_api.cc:935: `ntree_limit` is deprecated, use `iteration_range` instead.
## [15:54:57] WARNING: src/c_api/c_api.cc:935: `ntree_limit` is deprecated, use `iteration_range` instead.
## [15:54:57] WARNING: src/c_api/c_api.cc:935: `ntree_limit` is deprecated, use `iteration_range` instead.
## [15:54:57] WARNING: src/c_api/c_api.cc:935: `ntree_limit` is deprecated, use `iteration_range` instead.
## [15:54:57] WARNING: src/c_api/c_api.cc:935: `ntree_limit` is deprecated, use `iteration_range` instead.
## [15:54:57] WARNING: src/c_api/c_api.cc:935: `ntree_limit` is deprecated, use `iteration_range` instead.
## [15:54:57] WARNING: src/c_api/c_api.cc:935: `ntree_limit` is deprecated, use `iteration_range` instead.
## [15:54:57] WARNING: src/c_api/c_api.cc:935: `ntree_limit` is deprecated, use `iteration_range` instead.
## [15:54:57] WARNING: src/c_api/c_api.cc:935: `ntree_limit` is deprecated, use `iteration_range` instead.
## [15:54:58] WARNING: src/c_api/c_api.cc:935: `ntree_limit` is deprecated, use `iteration_range` instead.
## [15:54:58] WARNING: src/c_api/c_api.cc:935: `ntree_limit` is deprecated, use `iteration_range` instead.
## [15:54:58] WARNING: src/c_api/c_api.cc:935: `ntree_limit` is deprecated, use `iteration_range` instead.
## [15:54:58] WARNING: src/c_api/c_api.cc:935: `ntree_limit` is deprecated, use `iteration_range` instead.
## [15:54:58] WARNING: src/c_api/c_api.cc:935: `ntree_limit` is deprecated, use `iteration_range` instead.
## [15:54:58] WARNING: src/c_api/c_api.cc:935: `ntree_limit` is deprecated, use `iteration_range` instead.
## [15:54:58] WARNING: src/c_api/c_api.cc:935: `ntree_limit` is deprecated, use `iteration_range` instead.
## [15:54:58] WARNING: src/c_api/c_api.cc:935: `ntree_limit` is deprecated, use `iteration_range` instead.
## [15:54:58] WARNING: src/c_api/c_api.cc:935: `ntree_limit` is deprecated, use `iteration_range` instead.
## [15:54:58] WARNING: src/c_api/c_api.cc:935: `ntree_limit` is deprecated, use `iteration_range` instead.
## [15:54:59] WARNING: src/c_api/c_api.cc:935: `ntree_limit` is deprecated, use `iteration_range` instead.
## [15:54:59] WARNING: src/c_api/c_api.cc:935: `ntree_limit` is deprecated, use `iteration_range` instead.
## [15:54:59] WARNING: src/c_api/c_api.cc:935: `ntree_limit` is deprecated, use `iteration_range` instead.
## [15:54:59] WARNING: src/c_api/c_api.cc:935: `ntree_limit` is deprecated, use `iteration_range` instead.
## [15:54:59] WARNING: src/c_api/c_api.cc:935: `ntree_limit` is deprecated, use `iteration_range` instead.
## [15:54:59] WARNING: src/c_api/c_api.cc:935: `ntree_limit` is deprecated, use `iteration_range` instead.
## [15:54:59] WARNING: src/c_api/c_api.cc:935: `ntree_limit` is deprecated, use `iteration_range` instead.
## [15:54:59] WARNING: src/c_api/c_api.cc:935: `ntree_limit` is deprecated, use `iteration_range` instead.
## [15:54:59] WARNING: src/c_api/c_api.cc:935: `ntree_limit` is deprecated, use `iteration_range` instead.
## [15:54:59] WARNING: src/c_api/c_api.cc:935: `ntree_limit` is deprecated, use `iteration_range` instead.
## [15:55:00] WARNING: src/c_api/c_api.cc:935: `ntree_limit` is deprecated, use `iteration_range` instead.
## [15:55:00] WARNING: src/c_api/c_api.cc:935: `ntree_limit` is deprecated, use `iteration_range` instead.
## [15:55:00] WARNING: src/c_api/c_api.cc:935: `ntree_limit` is deprecated, use `iteration_range` instead.
## [15:55:00] WARNING: src/c_api/c_api.cc:935: `ntree_limit` is deprecated, use `iteration_range` instead.
## [15:55:00] WARNING: src/c_api/c_api.cc:935: `ntree_limit` is deprecated, use `iteration_range` instead.
## [15:55:00] WARNING: src/c_api/c_api.cc:935: `ntree_limit` is deprecated, use `iteration_range` instead.
## [15:55:00] WARNING: src/c_api/c_api.cc:935: `ntree_limit` is deprecated, use `iteration_range` instead.
## [15:55:00] WARNING: src/c_api/c_api.cc:935: `ntree_limit` is deprecated, use `iteration_range` instead.
## [15:55:00] WARNING: src/c_api/c_api.cc:935: `ntree_limit` is deprecated, use `iteration_range` instead.
## [15:55:00] WARNING: src/c_api/c_api.cc:935: `ntree_limit` is deprecated, use `iteration_range` instead.
## [15:55:00] WARNING: src/c_api/c_api.cc:935: `ntree_limit` is deprecated, use `iteration_range` instead.
## [15:55:00] WARNING: src/c_api/c_api.cc:935: `ntree_limit` is deprecated, use `iteration_range` instead.
## [15:55:00] WARNING: src/c_api/c_api.cc:935: `ntree_limit` is deprecated, use `iteration_range` instead.
## [15:55:00] WARNING: src/c_api/c_api.cc:935: `ntree_limit` is deprecated, use `iteration_range` instead.
## [15:55:01] WARNING: src/c_api/c_api.cc:935: `ntree_limit` is deprecated, use `iteration_range` instead.
## [15:55:01] WARNING: src/c_api/c_api.cc:935: `ntree_limit` is deprecated, use `iteration_range` instead.
## [15:55:01] WARNING: src/c_api/c_api.cc:935: `ntree_limit` is deprecated, use `iteration_range` instead.
## [15:55:01] WARNING: src/c_api/c_api.cc:935: `ntree_limit` is deprecated, use `iteration_range` instead.
## [15:55:01] WARNING: src/c_api/c_api.cc:935: `ntree_limit` is deprecated, use `iteration_range` instead.
## [15:55:01] WARNING: src/c_api/c_api.cc:935: `ntree_limit` is deprecated, use `iteration_range` instead.
## [15:55:01] WARNING: src/c_api/c_api.cc:935: `ntree_limit` is deprecated, use `iteration_range` instead.
## [15:55:01] WARNING: src/c_api/c_api.cc:935: `ntree_limit` is deprecated, use `iteration_range` instead.
## [15:55:01] WARNING: src/c_api/c_api.cc:935: `ntree_limit` is deprecated, use `iteration_range` instead.
## [15:55:01] WARNING: src/c_api/c_api.cc:935: `ntree_limit` is deprecated, use `iteration_range` instead.
## [15:55:02] WARNING: src/c_api/c_api.cc:935: `ntree_limit` is deprecated, use `iteration_range` instead.
## [15:55:02] WARNING: src/c_api/c_api.cc:935: `ntree_limit` is deprecated, use `iteration_range` instead.
## [15:55:02] WARNING: src/c_api/c_api.cc:935: `ntree_limit` is deprecated, use `iteration_range` instead.
## [15:55:02] WARNING: src/c_api/c_api.cc:935: `ntree_limit` is deprecated, use `iteration_range` instead.
## [15:55:02] WARNING: src/c_api/c_api.cc:935: `ntree_limit` is deprecated, use `iteration_range` instead.
## [15:55:02] WARNING: src/c_api/c_api.cc:935: `ntree_limit` is deprecated, use `iteration_range` instead.
## [15:55:02] WARNING: src/c_api/c_api.cc:935: `ntree_limit` is deprecated, use `iteration_range` instead.
## [15:55:02] WARNING: src/c_api/c_api.cc:935: `ntree_limit` is deprecated, use `iteration_range` instead.
## [15:55:02] WARNING: src/c_api/c_api.cc:935: `ntree_limit` is deprecated, use `iteration_range` instead.
## [15:55:02] WARNING: src/c_api/c_api.cc:935: `ntree_limit` is deprecated, use `iteration_range` instead.
## [15:55:03] WARNING: src/c_api/c_api.cc:935: `ntree_limit` is deprecated, use `iteration_range` instead.
## [15:55:03] WARNING: src/c_api/c_api.cc:935: `ntree_limit` is deprecated, use `iteration_range` instead.
## [15:55:03] WARNING: src/c_api/c_api.cc:935: `ntree_limit` is deprecated, use `iteration_range` instead.
## [15:55:03] WARNING: src/c_api/c_api.cc:935: `ntree_limit` is deprecated, use `iteration_range` instead.
## [15:55:03] WARNING: src/c_api/c_api.cc:935: `ntree_limit` is deprecated, use `iteration_range` instead.
## [15:55:03] WARNING: src/c_api/c_api.cc:935: `ntree_limit` is deprecated, use `iteration_range` instead.
## [15:55:03] WARNING: src/c_api/c_api.cc:935: `ntree_limit` is deprecated, use `iteration_range` instead.
## [15:55:03] WARNING: src/c_api/c_api.cc:935: `ntree_limit` is deprecated, use `iteration_range` instead.
## [15:55:03] WARNING: src/c_api/c_api.cc:935: `ntree_limit` is deprecated, use `iteration_range` instead.
## [15:55:03] WARNING: src/c_api/c_api.cc:935: `ntree_limit` is deprecated, use `iteration_range` instead.
## [15:55:03] WARNING: src/c_api/c_api.cc:935: `ntree_limit` is deprecated, use `iteration_range` instead.
## [15:55:03] WARNING: src/c_api/c_api.cc:935: `ntree_limit` is deprecated, use `iteration_range` instead.
## [15:55:03] WARNING: src/c_api/c_api.cc:935: `ntree_limit` is deprecated, use `iteration_range` instead.
## [15:55:03] WARNING: src/c_api/c_api.cc:935: `ntree_limit` is deprecated, use `iteration_range` instead.
## [15:55:04] WARNING: src/c_api/c_api.cc:935: `ntree_limit` is deprecated, use `iteration_range` instead.
## [15:55:04] WARNING: src/c_api/c_api.cc:935: `ntree_limit` is deprecated, use `iteration_range` instead.
## [15:55:04] WARNING: src/c_api/c_api.cc:935: `ntree_limit` is deprecated, use `iteration_range` instead.
## [15:55:04] WARNING: src/c_api/c_api.cc:935: `ntree_limit` is deprecated, use `iteration_range` instead.
## [15:55:04] WARNING: src/c_api/c_api.cc:935: `ntree_limit` is deprecated, use `iteration_range` instead.
## [15:55:04] WARNING: src/c_api/c_api.cc:935: `ntree_limit` is deprecated, use `iteration_range` instead.
## [15:55:04] WARNING: src/c_api/c_api.cc:935: `ntree_limit` is deprecated, use `iteration_range` instead.
## [15:55:04] WARNING: src/c_api/c_api.cc:935: `ntree_limit` is deprecated, use `iteration_range` instead.
## [15:55:04] WARNING: src/c_api/c_api.cc:935: `ntree_limit` is deprecated, use `iteration_range` instead.
## [15:55:04] WARNING: src/c_api/c_api.cc:935: `ntree_limit` is deprecated, use `iteration_range` instead.
## [15:55:05] WARNING: src/c_api/c_api.cc:935: `ntree_limit` is deprecated, use `iteration_range` instead.
## [15:55:05] WARNING: src/c_api/c_api.cc:935: `ntree_limit` is deprecated, use `iteration_range` instead.
## [15:55:05] WARNING: src/c_api/c_api.cc:935: `ntree_limit` is deprecated, use `iteration_range` instead.
## [15:55:05] WARNING: src/c_api/c_api.cc:935: `ntree_limit` is deprecated, use `iteration_range` instead.
## [15:55:05] WARNING: src/c_api/c_api.cc:935: `ntree_limit` is deprecated, use `iteration_range` instead.
## [15:55:05] WARNING: src/c_api/c_api.cc:935: `ntree_limit` is deprecated, use `iteration_range` instead.
## [15:55:05] WARNING: src/c_api/c_api.cc:935: `ntree_limit` is deprecated, use `iteration_range` instead.
## [15:55:05] WARNING: src/c_api/c_api.cc:935: `ntree_limit` is deprecated, use `iteration_range` instead.
## [15:55:06] WARNING: src/c_api/c_api.cc:935: `ntree_limit` is deprecated, use `iteration_range` instead.
## [15:55:06] WARNING: src/c_api/c_api.cc:935: `ntree_limit` is deprecated, use `iteration_range` instead.
## [15:55:06] WARNING: src/c_api/c_api.cc:935: `ntree_limit` is deprecated, use `iteration_range` instead.
## [15:55:06] WARNING: src/c_api/c_api.cc:935: `ntree_limit` is deprecated, use `iteration_range` instead.
## [15:55:06] WARNING: src/c_api/c_api.cc:935: `ntree_limit` is deprecated, use `iteration_range` instead.
## [15:55:06] WARNING: src/c_api/c_api.cc:935: `ntree_limit` is deprecated, use `iteration_range` instead.
## [15:55:06] WARNING: src/c_api/c_api.cc:935: `ntree_limit` is deprecated, use `iteration_range` instead.
## [15:55:06] WARNING: src/c_api/c_api.cc:935: `ntree_limit` is deprecated, use `iteration_range` instead.
## [15:55:06] WARNING: src/c_api/c_api.cc:935: `ntree_limit` is deprecated, use `iteration_range` instead.
## [15:55:06] WARNING: src/c_api/c_api.cc:935: `ntree_limit` is deprecated, use `iteration_range` instead.
## [15:55:06] WARNING: src/c_api/c_api.cc:935: `ntree_limit` is deprecated, use `iteration_range` instead.
## [15:55:06] WARNING: src/c_api/c_api.cc:935: `ntree_limit` is deprecated, use `iteration_range` instead.
## [15:55:07] WARNING: src/c_api/c_api.cc:935: `ntree_limit` is deprecated, use `iteration_range` instead.
## [15:55:07] WARNING: src/c_api/c_api.cc:935: `ntree_limit` is deprecated, use `iteration_range` instead.
## [15:55:07] WARNING: src/c_api/c_api.cc:935: `ntree_limit` is deprecated, use `iteration_range` instead.
## [15:55:07] WARNING: src/c_api/c_api.cc:935: `ntree_limit` is deprecated, use `iteration_range` instead.
## [15:55:07] WARNING: src/c_api/c_api.cc:935: `ntree_limit` is deprecated, use `iteration_range` instead.
## [15:55:07] WARNING: src/c_api/c_api.cc:935: `ntree_limit` is deprecated, use `iteration_range` instead.
## [15:55:07] WARNING: src/c_api/c_api.cc:935: `ntree_limit` is deprecated, use `iteration_range` instead.
## [15:55:07] WARNING: src/c_api/c_api.cc:935: `ntree_limit` is deprecated, use `iteration_range` instead.
## [15:55:07] WARNING: src/c_api/c_api.cc:935: `ntree_limit` is deprecated, use `iteration_range` instead.
## [15:55:07] WARNING: src/c_api/c_api.cc:935: `ntree_limit` is deprecated, use `iteration_range` instead.
## [15:55:07] WARNING: src/c_api/c_api.cc:935: `ntree_limit` is deprecated, use `iteration_range` instead.
## [15:55:07] WARNING: src/c_api/c_api.cc:935: `ntree_limit` is deprecated, use `iteration_range` instead.
## [15:55:08] WARNING: src/c_api/c_api.cc:935: `ntree_limit` is deprecated, use `iteration_range` instead.
## [15:55:08] WARNING: src/c_api/c_api.cc:935: `ntree_limit` is deprecated, use `iteration_range` instead.
## [15:55:08] WARNING: src/c_api/c_api.cc:935: `ntree_limit` is deprecated, use `iteration_range` instead.
## [15:55:08] WARNING: src/c_api/c_api.cc:935: `ntree_limit` is deprecated, use `iteration_range` instead.
## [15:55:08] WARNING: src/c_api/c_api.cc:935: `ntree_limit` is deprecated, use `iteration_range` instead.
## [15:55:08] WARNING: src/c_api/c_api.cc:935: `ntree_limit` is deprecated, use `iteration_range` instead.
## [15:55:08] WARNING: src/c_api/c_api.cc:935: `ntree_limit` is deprecated, use `iteration_range` instead.
## [15:55:08] WARNING: src/c_api/c_api.cc:935: `ntree_limit` is deprecated, use `iteration_range` instead.
## [15:55:08] WARNING: src/c_api/c_api.cc:935: `ntree_limit` is deprecated, use `iteration_range` instead.
## [15:55:08] WARNING: src/c_api/c_api.cc:935: `ntree_limit` is deprecated, use `iteration_range` instead.
## [15:55:09] WARNING: src/c_api/c_api.cc:935: `ntree_limit` is deprecated, use `iteration_range` instead.
## [15:55:09] WARNING: src/c_api/c_api.cc:935: `ntree_limit` is deprecated, use `iteration_range` instead.
## [15:55:09] WARNING: src/c_api/c_api.cc:935: `ntree_limit` is deprecated, use `iteration_range` instead.
## [15:55:09] WARNING: src/c_api/c_api.cc:935: `ntree_limit` is deprecated, use `iteration_range` instead.
## [15:55:09] WARNING: src/c_api/c_api.cc:935: `ntree_limit` is deprecated, use `iteration_range` instead.
## [15:55:09] WARNING: src/c_api/c_api.cc:935: `ntree_limit` is deprecated, use `iteration_range` instead.
## [15:55:09] WARNING: src/c_api/c_api.cc:935: `ntree_limit` is deprecated, use `iteration_range` instead.
## [15:55:09] WARNING: src/c_api/c_api.cc:935: `ntree_limit` is deprecated, use `iteration_range` instead.
## [15:55:09] WARNING: src/c_api/c_api.cc:935: `ntree_limit` is deprecated, use `iteration_range` instead.
## [15:55:09] WARNING: src/c_api/c_api.cc:935: `ntree_limit` is deprecated, use `iteration_range` instead.
## [15:55:10] WARNING: src/c_api/c_api.cc:935: `ntree_limit` is deprecated, use `iteration_range` instead.
## [15:55:10] WARNING: src/c_api/c_api.cc:935: `ntree_limit` is deprecated, use `iteration_range` instead.
## [15:55:10] WARNING: src/c_api/c_api.cc:935: `ntree_limit` is deprecated, use `iteration_range` instead.
## [15:55:10] WARNING: src/c_api/c_api.cc:935: `ntree_limit` is deprecated, use `iteration_range` instead.
## [15:55:10] WARNING: src/c_api/c_api.cc:935: `ntree_limit` is deprecated, use `iteration_range` instead.
## [15:55:10] WARNING: src/c_api/c_api.cc:935: `ntree_limit` is deprecated, use `iteration_range` instead.
## [15:55:10] WARNING: src/c_api/c_api.cc:935: `ntree_limit` is deprecated, use `iteration_range` instead.
## [15:55:10] WARNING: src/c_api/c_api.cc:935: `ntree_limit` is deprecated, use `iteration_range` instead.
## [15:55:10] WARNING: src/c_api/c_api.cc:935: `ntree_limit` is deprecated, use `iteration_range` instead.
## [15:55:10] WARNING: src/c_api/c_api.cc:935: `ntree_limit` is deprecated, use `iteration_range` instead.
## [15:55:10] WARNING: src/c_api/c_api.cc:935: `ntree_limit` is deprecated, use `iteration_range` instead.
## [15:55:10] WARNING: src/c_api/c_api.cc:935: `ntree_limit` is deprecated, use `iteration_range` instead.
## [15:55:10] WARNING: src/c_api/c_api.cc:935: `ntree_limit` is deprecated, use `iteration_range` instead.
## [15:55:10] WARNING: src/c_api/c_api.cc:935: `ntree_limit` is deprecated, use `iteration_range` instead.
## [15:55:11] WARNING: src/c_api/c_api.cc:935: `ntree_limit` is deprecated, use `iteration_range` instead.
## [15:55:11] WARNING: src/c_api/c_api.cc:935: `ntree_limit` is deprecated, use `iteration_range` instead.
## [15:55:11] WARNING: src/c_api/c_api.cc:935: `ntree_limit` is deprecated, use `iteration_range` instead.
## [15:55:11] WARNING: src/c_api/c_api.cc:935: `ntree_limit` is deprecated, use `iteration_range` instead.
## [15:55:11] WARNING: src/c_api/c_api.cc:935: `ntree_limit` is deprecated, use `iteration_range` instead.
## [15:55:11] WARNING: src/c_api/c_api.cc:935: `ntree_limit` is deprecated, use `iteration_range` instead.
## [15:55:11] WARNING: src/c_api/c_api.cc:935: `ntree_limit` is deprecated, use `iteration_range` instead.
## [15:55:11] WARNING: src/c_api/c_api.cc:935: `ntree_limit` is deprecated, use `iteration_range` instead.
## [15:55:11] WARNING: src/c_api/c_api.cc:935: `ntree_limit` is deprecated, use `iteration_range` instead.
## [15:55:11] WARNING: src/c_api/c_api.cc:935: `ntree_limit` is deprecated, use `iteration_range` instead.
## [15:55:12] WARNING: src/c_api/c_api.cc:935: `ntree_limit` is deprecated, use `iteration_range` instead.
## [15:55:12] WARNING: src/c_api/c_api.cc:935: `ntree_limit` is deprecated, use `iteration_range` instead.
## [15:55:12] WARNING: src/c_api/c_api.cc:935: `ntree_limit` is deprecated, use `iteration_range` instead.
## [15:55:12] WARNING: src/c_api/c_api.cc:935: `ntree_limit` is deprecated, use `iteration_range` instead.
## [15:55:12] WARNING: src/c_api/c_api.cc:935: `ntree_limit` is deprecated, use `iteration_range` instead.
## [15:55:12] WARNING: src/c_api/c_api.cc:935: `ntree_limit` is deprecated, use `iteration_range` instead.
## [15:55:12] WARNING: src/c_api/c_api.cc:935: `ntree_limit` is deprecated, use `iteration_range` instead.
## [15:55:12] WARNING: src/c_api/c_api.cc:935: `ntree_limit` is deprecated, use `iteration_range` instead.
## [15:55:13] WARNING: src/c_api/c_api.cc:935: `ntree_limit` is deprecated, use `iteration_range` instead.
## [15:55:13] WARNING: src/c_api/c_api.cc:935: `ntree_limit` is deprecated, use `iteration_range` instead.
## [15:55:13] WARNING: src/c_api/c_api.cc:935: `ntree_limit` is deprecated, use `iteration_range` instead.
## [15:55:13] WARNING: src/c_api/c_api.cc:935: `ntree_limit` is deprecated, use `iteration_range` instead.
## [15:55:13] WARNING: src/c_api/c_api.cc:935: `ntree_limit` is deprecated, use `iteration_range` instead.
## [15:55:13] WARNING: src/c_api/c_api.cc:935: `ntree_limit` is deprecated, use `iteration_range` instead.
## [15:55:13] WARNING: src/c_api/c_api.cc:935: `ntree_limit` is deprecated, use `iteration_range` instead.
## [15:55:13] WARNING: src/c_api/c_api.cc:935: `ntree_limit` is deprecated, use `iteration_range` instead.
## [15:55:13] WARNING: src/c_api/c_api.cc:935: `ntree_limit` is deprecated, use `iteration_range` instead.
## [15:55:13] WARNING: src/c_api/c_api.cc:935: `ntree_limit` is deprecated, use `iteration_range` instead.
## [15:55:13] WARNING: src/c_api/c_api.cc:935: `ntree_limit` is deprecated, use `iteration_range` instead.
## [15:55:13] WARNING: src/c_api/c_api.cc:935: `ntree_limit` is deprecated, use `iteration_range` instead.
## [15:55:13] WARNING: src/c_api/c_api.cc:935: `ntree_limit` is deprecated, use `iteration_range` instead.
## [15:55:13] WARNING: src/c_api/c_api.cc:935: `ntree_limit` is deprecated, use `iteration_range` instead.
## [15:55:14] WARNING: src/c_api/c_api.cc:935: `ntree_limit` is deprecated, use `iteration_range` instead.
## [15:55:14] WARNING: src/c_api/c_api.cc:935: `ntree_limit` is deprecated, use `iteration_range` instead.
## [15:55:14] WARNING: src/c_api/c_api.cc:935: `ntree_limit` is deprecated, use `iteration_range` instead.
## [15:55:14] WARNING: src/c_api/c_api.cc:935: `ntree_limit` is deprecated, use `iteration_range` instead.
## [15:55:14] WARNING: src/c_api/c_api.cc:935: `ntree_limit` is deprecated, use `iteration_range` instead.
## [15:55:14] WARNING: src/c_api/c_api.cc:935: `ntree_limit` is deprecated, use `iteration_range` instead.
## [15:55:14] WARNING: src/c_api/c_api.cc:935: `ntree_limit` is deprecated, use `iteration_range` instead.
## [15:55:14] WARNING: src/c_api/c_api.cc:935: `ntree_limit` is deprecated, use `iteration_range` instead.
## [15:55:14] WARNING: src/c_api/c_api.cc:935: `ntree_limit` is deprecated, use `iteration_range` instead.
## [15:55:14] WARNING: src/c_api/c_api.cc:935: `ntree_limit` is deprecated, use `iteration_range` instead.
## [15:55:15] WARNING: src/c_api/c_api.cc:935: `ntree_limit` is deprecated, use `iteration_range` instead.
## [15:55:15] WARNING: src/c_api/c_api.cc:935: `ntree_limit` is deprecated, use `iteration_range` instead.
## [15:55:15] WARNING: src/c_api/c_api.cc:935: `ntree_limit` is deprecated, use `iteration_range` instead.
## [15:55:15] WARNING: src/c_api/c_api.cc:935: `ntree_limit` is deprecated, use `iteration_range` instead.
## [15:55:15] WARNING: src/c_api/c_api.cc:935: `ntree_limit` is deprecated, use `iteration_range` instead.
## [15:55:15] WARNING: src/c_api/c_api.cc:935: `ntree_limit` is deprecated, use `iteration_range` instead.
## [15:55:15] WARNING: src/c_api/c_api.cc:935: `ntree_limit` is deprecated, use `iteration_range` instead.
## [15:55:15] WARNING: src/c_api/c_api.cc:935: `ntree_limit` is deprecated, use `iteration_range` instead.
## [15:55:15] WARNING: src/c_api/c_api.cc:935: `ntree_limit` is deprecated, use `iteration_range` instead.
## [15:55:15] WARNING: src/c_api/c_api.cc:935: `ntree_limit` is deprecated, use `iteration_range` instead.
## [15:55:16] WARNING: src/c_api/c_api.cc:935: `ntree_limit` is deprecated, use `iteration_range` instead.
## [15:55:16] WARNING: src/c_api/c_api.cc:935: `ntree_limit` is deprecated, use `iteration_range` instead.
## [15:55:16] WARNING: src/c_api/c_api.cc:935: `ntree_limit` is deprecated, use `iteration_range` instead.
## [15:55:16] WARNING: src/c_api/c_api.cc:935: `ntree_limit` is deprecated, use `iteration_range` instead.
## [15:55:16] WARNING: src/c_api/c_api.cc:935: `ntree_limit` is deprecated, use `iteration_range` instead.
## [15:55:16] WARNING: src/c_api/c_api.cc:935: `ntree_limit` is deprecated, use `iteration_range` instead.
## [15:55:16] WARNING: src/c_api/c_api.cc:935: `ntree_limit` is deprecated, use `iteration_range` instead.
## [15:55:16] WARNING: src/c_api/c_api.cc:935: `ntree_limit` is deprecated, use `iteration_range` instead.
## [15:55:16] WARNING: src/c_api/c_api.cc:935: `ntree_limit` is deprecated, use `iteration_range` instead.
## [15:55:16] WARNING: src/c_api/c_api.cc:935: `ntree_limit` is deprecated, use `iteration_range` instead.
## [15:55:16] WARNING: src/c_api/c_api.cc:935: `ntree_limit` is deprecated, use `iteration_range` instead.
## [15:55:16] WARNING: src/c_api/c_api.cc:935: `ntree_limit` is deprecated, use `iteration_range` instead.
## [15:55:16] WARNING: src/c_api/c_api.cc:935: `ntree_limit` is deprecated, use `iteration_range` instead.
## [15:55:16] WARNING: src/c_api/c_api.cc:935: `ntree_limit` is deprecated, use `iteration_range` instead.
## [15:55:17] WARNING: src/c_api/c_api.cc:935: `ntree_limit` is deprecated, use `iteration_range` instead.
## [15:55:17] WARNING: src/c_api/c_api.cc:935: `ntree_limit` is deprecated, use `iteration_range` instead.
## [15:55:17] WARNING: src/c_api/c_api.cc:935: `ntree_limit` is deprecated, use `iteration_range` instead.
## [15:55:17] WARNING: src/c_api/c_api.cc:935: `ntree_limit` is deprecated, use `iteration_range` instead.
## [15:55:17] WARNING: src/c_api/c_api.cc:935: `ntree_limit` is deprecated, use `iteration_range` instead.
## [15:55:17] WARNING: src/c_api/c_api.cc:935: `ntree_limit` is deprecated, use `iteration_range` instead.
## [15:55:17] WARNING: src/c_api/c_api.cc:935: `ntree_limit` is deprecated, use `iteration_range` instead.
## [15:55:17] WARNING: src/c_api/c_api.cc:935: `ntree_limit` is deprecated, use `iteration_range` instead.
## [15:55:17] WARNING: src/c_api/c_api.cc:935: `ntree_limit` is deprecated, use `iteration_range` instead.
## [15:55:17] WARNING: src/c_api/c_api.cc:935: `ntree_limit` is deprecated, use `iteration_range` instead.
## [15:55:18] WARNING: src/c_api/c_api.cc:935: `ntree_limit` is deprecated, use `iteration_range` instead.
## [15:55:18] WARNING: src/c_api/c_api.cc:935: `ntree_limit` is deprecated, use `iteration_range` instead.
## [15:55:18] WARNING: src/c_api/c_api.cc:935: `ntree_limit` is deprecated, use `iteration_range` instead.
## [15:55:18] WARNING: src/c_api/c_api.cc:935: `ntree_limit` is deprecated, use `iteration_range` instead.
## [15:55:18] WARNING: src/c_api/c_api.cc:935: `ntree_limit` is deprecated, use `iteration_range` instead.
## [15:55:18] WARNING: src/c_api/c_api.cc:935: `ntree_limit` is deprecated, use `iteration_range` instead.
## [15:55:18] WARNING: src/c_api/c_api.cc:935: `ntree_limit` is deprecated, use `iteration_range` instead.
## [15:55:18] WARNING: src/c_api/c_api.cc:935: `ntree_limit` is deprecated, use `iteration_range` instead.
## [15:55:18] WARNING: src/c_api/c_api.cc:935: `ntree_limit` is deprecated, use `iteration_range` instead.
## [15:55:18] WARNING: src/c_api/c_api.cc:935: `ntree_limit` is deprecated, use `iteration_range` instead.
## [15:55:19] WARNING: src/c_api/c_api.cc:935: `ntree_limit` is deprecated, use `iteration_range` instead.
## [15:55:19] WARNING: src/c_api/c_api.cc:935: `ntree_limit` is deprecated, use `iteration_range` instead.
## [15:55:19] WARNING: src/c_api/c_api.cc:935: `ntree_limit` is deprecated, use `iteration_range` instead.
## [15:55:19] WARNING: src/c_api/c_api.cc:935: `ntree_limit` is deprecated, use `iteration_range` instead.
## [15:55:19] WARNING: src/c_api/c_api.cc:935: `ntree_limit` is deprecated, use `iteration_range` instead.
## [15:55:19] WARNING: src/c_api/c_api.cc:935: `ntree_limit` is deprecated, use `iteration_range` instead.
## [15:55:19] WARNING: src/c_api/c_api.cc:935: `ntree_limit` is deprecated, use `iteration_range` instead.
## [15:55:19] WARNING: src/c_api/c_api.cc:935: `ntree_limit` is deprecated, use `iteration_range` instead.
## [15:55:19] WARNING: src/c_api/c_api.cc:935: `ntree_limit` is deprecated, use `iteration_range` instead.
## [15:55:19] WARNING: src/c_api/c_api.cc:935: `ntree_limit` is deprecated, use `iteration_range` instead.
## [15:55:20] WARNING: src/c_api/c_api.cc:935: `ntree_limit` is deprecated, use `iteration_range` instead.
## [15:55:20] WARNING: src/c_api/c_api.cc:935: `ntree_limit` is deprecated, use `iteration_range` instead.
## [15:55:20] WARNING: src/c_api/c_api.cc:935: `ntree_limit` is deprecated, use `iteration_range` instead.
## [15:55:20] WARNING: src/c_api/c_api.cc:935: `ntree_limit` is deprecated, use `iteration_range` instead.
## [15:55:20] WARNING: src/c_api/c_api.cc:935: `ntree_limit` is deprecated, use `iteration_range` instead.
## [15:55:20] WARNING: src/c_api/c_api.cc:935: `ntree_limit` is deprecated, use `iteration_range` instead.
## [15:55:20] WARNING: src/c_api/c_api.cc:935: `ntree_limit` is deprecated, use `iteration_range` instead.
## [15:55:20] WARNING: src/c_api/c_api.cc:935: `ntree_limit` is deprecated, use `iteration_range` instead.
## [15:55:20] WARNING: src/c_api/c_api.cc:935: `ntree_limit` is deprecated, use `iteration_range` instead.
## [15:55:20] WARNING: src/c_api/c_api.cc:935: `ntree_limit` is deprecated, use `iteration_range` instead.
## [15:55:20] WARNING: src/c_api/c_api.cc:935: `ntree_limit` is deprecated, use `iteration_range` instead.
## [15:55:20] WARNING: src/c_api/c_api.cc:935: `ntree_limit` is deprecated, use `iteration_range` instead.
## [15:55:21] WARNING: src/c_api/c_api.cc:935: `ntree_limit` is deprecated, use `iteration_range` instead.
## [15:55:21] WARNING: src/c_api/c_api.cc:935: `ntree_limit` is deprecated, use `iteration_range` instead.
## [15:55:21] WARNING: src/c_api/c_api.cc:935: `ntree_limit` is deprecated, use `iteration_range` instead.
## [15:55:21] WARNING: src/c_api/c_api.cc:935: `ntree_limit` is deprecated, use `iteration_range` instead.
## [15:55:21] WARNING: src/c_api/c_api.cc:935: `ntree_limit` is deprecated, use `iteration_range` instead.
## [15:55:21] WARNING: src/c_api/c_api.cc:935: `ntree_limit` is deprecated, use `iteration_range` instead.
## [15:55:21] WARNING: src/c_api/c_api.cc:935: `ntree_limit` is deprecated, use `iteration_range` instead.
## [15:55:21] WARNING: src/c_api/c_api.cc:935: `ntree_limit` is deprecated, use `iteration_range` instead.
## [15:55:21] WARNING: src/c_api/c_api.cc:935: `ntree_limit` is deprecated, use `iteration_range` instead.
## [15:55:21] WARNING: src/c_api/c_api.cc:935: `ntree_limit` is deprecated, use `iteration_range` instead.
## [15:55:22] WARNING: src/c_api/c_api.cc:935: `ntree_limit` is deprecated, use `iteration_range` instead.
## [15:55:22] WARNING: src/c_api/c_api.cc:935: `ntree_limit` is deprecated, use `iteration_range` instead.
## [15:55:22] WARNING: src/c_api/c_api.cc:935: `ntree_limit` is deprecated, use `iteration_range` instead.
## [15:55:22] WARNING: src/c_api/c_api.cc:935: `ntree_limit` is deprecated, use `iteration_range` instead.
## [15:55:22] WARNING: src/c_api/c_api.cc:935: `ntree_limit` is deprecated, use `iteration_range` instead.
## [15:55:22] WARNING: src/c_api/c_api.cc:935: `ntree_limit` is deprecated, use `iteration_range` instead.
## [15:55:22] WARNING: src/c_api/c_api.cc:935: `ntree_limit` is deprecated, use `iteration_range` instead.
## [15:55:22] WARNING: src/c_api/c_api.cc:935: `ntree_limit` is deprecated, use `iteration_range` instead.
## [15:55:22] WARNING: src/c_api/c_api.cc:935: `ntree_limit` is deprecated, use `iteration_range` instead.
## [15:55:22] WARNING: src/c_api/c_api.cc:935: `ntree_limit` is deprecated, use `iteration_range` instead.
## [15:55:25] WARNING: src/c_api/c_api.cc:935: `ntree_limit` is deprecated, use `iteration_range` instead.
## [15:55:25] WARNING: src/c_api/c_api.cc:935: `ntree_limit` is deprecated, use `iteration_range` instead.
## [15:55:25] WARNING: src/c_api/c_api.cc:935: `ntree_limit` is deprecated, use `iteration_range` instead.
## [15:55:25] WARNING: src/c_api/c_api.cc:935: `ntree_limit` is deprecated, use `iteration_range` instead.
## [15:55:25] WARNING: src/c_api/c_api.cc:935: `ntree_limit` is deprecated, use `iteration_range` instead.
## [15:55:25] WARNING: src/c_api/c_api.cc:935: `ntree_limit` is deprecated, use `iteration_range` instead.
## [15:55:26] WARNING: src/c_api/c_api.cc:935: `ntree_limit` is deprecated, use `iteration_range` instead.
## [15:55:26] WARNING: src/c_api/c_api.cc:935: `ntree_limit` is deprecated, use `iteration_range` instead.
## [15:55:26] WARNING: src/c_api/c_api.cc:935: `ntree_limit` is deprecated, use `iteration_range` instead.
## [15:55:26] WARNING: src/c_api/c_api.cc:935: `ntree_limit` is deprecated, use `iteration_range` instead.
## [15:55:26] WARNING: src/c_api/c_api.cc:935: `ntree_limit` is deprecated, use `iteration_range` instead.
## [15:55:26] WARNING: src/c_api/c_api.cc:935: `ntree_limit` is deprecated, use `iteration_range` instead.
## [15:55:26] WARNING: src/c_api/c_api.cc:935: `ntree_limit` is deprecated, use `iteration_range` instead.
## [15:55:26] WARNING: src/c_api/c_api.cc:935: `ntree_limit` is deprecated, use `iteration_range` instead.
## [15:55:26] WARNING: src/c_api/c_api.cc:935: `ntree_limit` is deprecated, use `iteration_range` instead.
## [15:55:26] WARNING: src/c_api/c_api.cc:935: `ntree_limit` is deprecated, use `iteration_range` instead.
## [15:55:26] WARNING: src/c_api/c_api.cc:935: `ntree_limit` is deprecated, use `iteration_range` instead.
## [15:55:26] WARNING: src/c_api/c_api.cc:935: `ntree_limit` is deprecated, use `iteration_range` instead.
## [15:55:27] WARNING: src/c_api/c_api.cc:935: `ntree_limit` is deprecated, use `iteration_range` instead.
## [15:55:27] WARNING: src/c_api/c_api.cc:935: `ntree_limit` is deprecated, use `iteration_range` instead.
## [15:55:27] WARNING: src/c_api/c_api.cc:935: `ntree_limit` is deprecated, use `iteration_range` instead.
## [15:55:27] WARNING: src/c_api/c_api.cc:935: `ntree_limit` is deprecated, use `iteration_range` instead.
## [15:55:27] WARNING: src/c_api/c_api.cc:935: `ntree_limit` is deprecated, use `iteration_range` instead.
## [15:55:27] WARNING: src/c_api/c_api.cc:935: `ntree_limit` is deprecated, use `iteration_range` instead.
## [15:55:27] WARNING: src/c_api/c_api.cc:935: `ntree_limit` is deprecated, use `iteration_range` instead.
## [15:55:27] WARNING: src/c_api/c_api.cc:935: `ntree_limit` is deprecated, use `iteration_range` instead.
## [15:55:27] WARNING: src/c_api/c_api.cc:935: `ntree_limit` is deprecated, use `iteration_range` instead.
## [15:55:27] WARNING: src/c_api/c_api.cc:935: `ntree_limit` is deprecated, use `iteration_range` instead.
## [15:55:28] WARNING: src/c_api/c_api.cc:935: `ntree_limit` is deprecated, use `iteration_range` instead.
## [15:55:28] WARNING: src/c_api/c_api.cc:935: `ntree_limit` is deprecated, use `iteration_range` instead.
## [15:55:28] WARNING: src/c_api/c_api.cc:935: `ntree_limit` is deprecated, use `iteration_range` instead.
## [15:55:28] WARNING: src/c_api/c_api.cc:935: `ntree_limit` is deprecated, use `iteration_range` instead.
## [15:55:28] WARNING: src/c_api/c_api.cc:935: `ntree_limit` is deprecated, use `iteration_range` instead.
## [15:55:28] WARNING: src/c_api/c_api.cc:935: `ntree_limit` is deprecated, use `iteration_range` instead.
## [15:55:28] WARNING: src/c_api/c_api.cc:935: `ntree_limit` is deprecated, use `iteration_range` instead.
## [15:55:28] WARNING: src/c_api/c_api.cc:935: `ntree_limit` is deprecated, use `iteration_range` instead.
## [15:55:28] WARNING: src/c_api/c_api.cc:935: `ntree_limit` is deprecated, use `iteration_range` instead.
## [15:55:28] WARNING: src/c_api/c_api.cc:935: `ntree_limit` is deprecated, use `iteration_range` instead.
## [15:55:28] WARNING: src/c_api/c_api.cc:935: `ntree_limit` is deprecated, use `iteration_range` instead.
## [15:55:28] WARNING: src/c_api/c_api.cc:935: `ntree_limit` is deprecated, use `iteration_range` instead.
## [15:55:29] WARNING: src/c_api/c_api.cc:935: `ntree_limit` is deprecated, use `iteration_range` instead.
## [15:55:29] WARNING: src/c_api/c_api.cc:935: `ntree_limit` is deprecated, use `iteration_range` instead.
## [15:55:29] WARNING: src/c_api/c_api.cc:935: `ntree_limit` is deprecated, use `iteration_range` instead.
## [15:55:29] WARNING: src/c_api/c_api.cc:935: `ntree_limit` is deprecated, use `iteration_range` instead.
## [15:55:29] WARNING: src/c_api/c_api.cc:935: `ntree_limit` is deprecated, use `iteration_range` instead.
## [15:55:29] WARNING: src/c_api/c_api.cc:935: `ntree_limit` is deprecated, use `iteration_range` instead.
## [15:55:29] WARNING: src/c_api/c_api.cc:935: `ntree_limit` is deprecated, use `iteration_range` instead.
## [15:55:29] WARNING: src/c_api/c_api.cc:935: `ntree_limit` is deprecated, use `iteration_range` instead.
## [15:55:29] WARNING: src/c_api/c_api.cc:935: `ntree_limit` is deprecated, use `iteration_range` instead.
## [15:55:29] WARNING: src/c_api/c_api.cc:935: `ntree_limit` is deprecated, use `iteration_range` instead.
## [15:55:29] WARNING: src/c_api/c_api.cc:935: `ntree_limit` is deprecated, use `iteration_range` instead.
## [15:55:29] WARNING: src/c_api/c_api.cc:935: `ntree_limit` is deprecated, use `iteration_range` instead.
## [15:55:30] WARNING: src/c_api/c_api.cc:935: `ntree_limit` is deprecated, use `iteration_range` instead.
## [15:55:30] WARNING: src/c_api/c_api.cc:935: `ntree_limit` is deprecated, use `iteration_range` instead.
## [15:55:30] WARNING: src/c_api/c_api.cc:935: `ntree_limit` is deprecated, use `iteration_range` instead.
## [15:55:30] WARNING: src/c_api/c_api.cc:935: `ntree_limit` is deprecated, use `iteration_range` instead.
## [15:55:30] WARNING: src/c_api/c_api.cc:935: `ntree_limit` is deprecated, use `iteration_range` instead.
## [15:55:30] WARNING: src/c_api/c_api.cc:935: `ntree_limit` is deprecated, use `iteration_range` instead.
## [15:55:30] WARNING: src/c_api/c_api.cc:935: `ntree_limit` is deprecated, use `iteration_range` instead.
## [15:55:30] WARNING: src/c_api/c_api.cc:935: `ntree_limit` is deprecated, use `iteration_range` instead.
## [15:55:30] WARNING: src/c_api/c_api.cc:935: `ntree_limit` is deprecated, use `iteration_range` instead.
## [15:55:30] WARNING: src/c_api/c_api.cc:935: `ntree_limit` is deprecated, use `iteration_range` instead.
## [15:55:30] WARNING: src/c_api/c_api.cc:935: `ntree_limit` is deprecated, use `iteration_range` instead.
## [15:55:30] WARNING: src/c_api/c_api.cc:935: `ntree_limit` is deprecated, use `iteration_range` instead.
## [15:55:31] WARNING: src/c_api/c_api.cc:935: `ntree_limit` is deprecated, use `iteration_range` instead.
## [15:55:31] WARNING: src/c_api/c_api.cc:935: `ntree_limit` is deprecated, use `iteration_range` instead.
## [15:55:31] WARNING: src/c_api/c_api.cc:935: `ntree_limit` is deprecated, use `iteration_range` instead.
## [15:55:31] WARNING: src/c_api/c_api.cc:935: `ntree_limit` is deprecated, use `iteration_range` instead.
## [15:55:31] WARNING: src/c_api/c_api.cc:935: `ntree_limit` is deprecated, use `iteration_range` instead.
## [15:55:31] WARNING: src/c_api/c_api.cc:935: `ntree_limit` is deprecated, use `iteration_range` instead.
## [15:55:31] WARNING: src/c_api/c_api.cc:935: `ntree_limit` is deprecated, use `iteration_range` instead.
## [15:55:31] WARNING: src/c_api/c_api.cc:935: `ntree_limit` is deprecated, use `iteration_range` instead.
## [15:55:31] WARNING: src/c_api/c_api.cc:935: `ntree_limit` is deprecated, use `iteration_range` instead.
## [15:55:31] WARNING: src/c_api/c_api.cc:935: `ntree_limit` is deprecated, use `iteration_range` instead.
## [15:55:32] WARNING: src/c_api/c_api.cc:935: `ntree_limit` is deprecated, use `iteration_range` instead.
## [15:55:32] WARNING: src/c_api/c_api.cc:935: `ntree_limit` is deprecated, use `iteration_range` instead.
## [15:55:32] WARNING: src/c_api/c_api.cc:935: `ntree_limit` is deprecated, use `iteration_range` instead.
## [15:55:32] WARNING: src/c_api/c_api.cc:935: `ntree_limit` is deprecated, use `iteration_range` instead.
## [15:55:32] WARNING: src/c_api/c_api.cc:935: `ntree_limit` is deprecated, use `iteration_range` instead.
## [15:55:32] WARNING: src/c_api/c_api.cc:935: `ntree_limit` is deprecated, use `iteration_range` instead.
## [15:55:32] WARNING: src/c_api/c_api.cc:935: `ntree_limit` is deprecated, use `iteration_range` instead.
## [15:55:32] WARNING: src/c_api/c_api.cc:935: `ntree_limit` is deprecated, use `iteration_range` instead.
## [15:55:32] WARNING: src/c_api/c_api.cc:935: `ntree_limit` is deprecated, use `iteration_range` instead.
## [15:55:32] WARNING: src/c_api/c_api.cc:935: `ntree_limit` is deprecated, use `iteration_range` instead.
## [15:55:32] WARNING: src/c_api/c_api.cc:935: `ntree_limit` is deprecated, use `iteration_range` instead.
## [15:55:32] WARNING: src/c_api/c_api.cc:935: `ntree_limit` is deprecated, use `iteration_range` instead.
## [15:55:32] WARNING: src/c_api/c_api.cc:935: `ntree_limit` is deprecated, use `iteration_range` instead.
## [15:55:32] WARNING: src/c_api/c_api.cc:935: `ntree_limit` is deprecated, use `iteration_range` instead.
## [15:55:33] WARNING: src/c_api/c_api.cc:935: `ntree_limit` is deprecated, use `iteration_range` instead.
## [15:55:33] WARNING: src/c_api/c_api.cc:935: `ntree_limit` is deprecated, use `iteration_range` instead.
## [15:55:33] WARNING: src/c_api/c_api.cc:935: `ntree_limit` is deprecated, use `iteration_range` instead.
## [15:55:33] WARNING: src/c_api/c_api.cc:935: `ntree_limit` is deprecated, use `iteration_range` instead.
## [15:55:33] WARNING: src/c_api/c_api.cc:935: `ntree_limit` is deprecated, use `iteration_range` instead.
## [15:55:33] WARNING: src/c_api/c_api.cc:935: `ntree_limit` is deprecated, use `iteration_range` instead.
## [15:55:33] WARNING: src/c_api/c_api.cc:935: `ntree_limit` is deprecated, use `iteration_range` instead.
## [15:55:33] WARNING: src/c_api/c_api.cc:935: `ntree_limit` is deprecated, use `iteration_range` instead.
## [15:55:33] WARNING: src/c_api/c_api.cc:935: `ntree_limit` is deprecated, use `iteration_range` instead.
## [15:55:33] WARNING: src/c_api/c_api.cc:935: `ntree_limit` is deprecated, use `iteration_range` instead.
## [15:55:34] WARNING: src/c_api/c_api.cc:935: `ntree_limit` is deprecated, use `iteration_range` instead.
## [15:55:34] WARNING: src/c_api/c_api.cc:935: `ntree_limit` is deprecated, use `iteration_range` instead.
## [15:55:34] WARNING: src/c_api/c_api.cc:935: `ntree_limit` is deprecated, use `iteration_range` instead.
## [15:55:34] WARNING: src/c_api/c_api.cc:935: `ntree_limit` is deprecated, use `iteration_range` instead.
## [15:55:34] WARNING: src/c_api/c_api.cc:935: `ntree_limit` is deprecated, use `iteration_range` instead.
## [15:55:34] WARNING: src/c_api/c_api.cc:935: `ntree_limit` is deprecated, use `iteration_range` instead.
## [15:55:34] WARNING: src/c_api/c_api.cc:935: `ntree_limit` is deprecated, use `iteration_range` instead.
## [15:55:34] WARNING: src/c_api/c_api.cc:935: `ntree_limit` is deprecated, use `iteration_range` instead.
## [15:55:35] WARNING: src/c_api/c_api.cc:935: `ntree_limit` is deprecated, use `iteration_range` instead.
## [15:55:35] WARNING: src/c_api/c_api.cc:935: `ntree_limit` is deprecated, use `iteration_range` instead.
## [15:55:35] WARNING: src/c_api/c_api.cc:935: `ntree_limit` is deprecated, use `iteration_range` instead.
## [15:55:35] WARNING: src/c_api/c_api.cc:935: `ntree_limit` is deprecated, use `iteration_range` instead.
## [15:55:35] WARNING: src/c_api/c_api.cc:935: `ntree_limit` is deprecated, use `iteration_range` instead.
## [15:55:35] WARNING: src/c_api/c_api.cc:935: `ntree_limit` is deprecated, use `iteration_range` instead.
## [15:55:35] WARNING: src/c_api/c_api.cc:935: `ntree_limit` is deprecated, use `iteration_range` instead.
## [15:55:35] WARNING: src/c_api/c_api.cc:935: `ntree_limit` is deprecated, use `iteration_range` instead.
## [15:55:35] WARNING: src/c_api/c_api.cc:935: `ntree_limit` is deprecated, use `iteration_range` instead.
## [15:55:35] WARNING: src/c_api/c_api.cc:935: `ntree_limit` is deprecated, use `iteration_range` instead.
## [15:55:35] WARNING: src/c_api/c_api.cc:935: `ntree_limit` is deprecated, use `iteration_range` instead.
## [15:55:35] WARNING: src/c_api/c_api.cc:935: `ntree_limit` is deprecated, use `iteration_range` instead.
## [15:55:35] WARNING: src/c_api/c_api.cc:935: `ntree_limit` is deprecated, use `iteration_range` instead.
## [15:55:35] WARNING: src/c_api/c_api.cc:935: `ntree_limit` is deprecated, use `iteration_range` instead.
## [15:55:36] WARNING: src/c_api/c_api.cc:935: `ntree_limit` is deprecated, use `iteration_range` instead.
## [15:55:36] WARNING: src/c_api/c_api.cc:935: `ntree_limit` is deprecated, use `iteration_range` instead.
## [15:55:36] WARNING: src/c_api/c_api.cc:935: `ntree_limit` is deprecated, use `iteration_range` instead.
## [15:55:36] WARNING: src/c_api/c_api.cc:935: `ntree_limit` is deprecated, use `iteration_range` instead.
## [15:55:36] WARNING: src/c_api/c_api.cc:935: `ntree_limit` is deprecated, use `iteration_range` instead.
## [15:55:36] WARNING: src/c_api/c_api.cc:935: `ntree_limit` is deprecated, use `iteration_range` instead.
## [15:55:36] WARNING: src/c_api/c_api.cc:935: `ntree_limit` is deprecated, use `iteration_range` instead.
## [15:55:36] WARNING: src/c_api/c_api.cc:935: `ntree_limit` is deprecated, use `iteration_range` instead.
## [15:55:36] WARNING: src/c_api/c_api.cc:935: `ntree_limit` is deprecated, use `iteration_range` instead.
## [15:55:36] WARNING: src/c_api/c_api.cc:935: `ntree_limit` is deprecated, use `iteration_range` instead.
## [15:55:36] WARNING: src/c_api/c_api.cc:935: `ntree_limit` is deprecated, use `iteration_range` instead.
## [15:55:36] WARNING: src/c_api/c_api.cc:935: `ntree_limit` is deprecated, use `iteration_range` instead.
## [15:55:37] WARNING: src/c_api/c_api.cc:935: `ntree_limit` is deprecated, use `iteration_range` instead.
## [15:55:37] WARNING: src/c_api/c_api.cc:935: `ntree_limit` is deprecated, use `iteration_range` instead.
## [15:55:37] WARNING: src/c_api/c_api.cc:935: `ntree_limit` is deprecated, use `iteration_range` instead.
## [15:55:37] WARNING: src/c_api/c_api.cc:935: `ntree_limit` is deprecated, use `iteration_range` instead.
## [15:55:37] WARNING: src/c_api/c_api.cc:935: `ntree_limit` is deprecated, use `iteration_range` instead.
## [15:55:37] WARNING: src/c_api/c_api.cc:935: `ntree_limit` is deprecated, use `iteration_range` instead.
## [15:55:37] WARNING: src/c_api/c_api.cc:935: `ntree_limit` is deprecated, use `iteration_range` instead.
## [15:55:37] WARNING: src/c_api/c_api.cc:935: `ntree_limit` is deprecated, use `iteration_range` instead.
## [15:55:37] WARNING: src/c_api/c_api.cc:935: `ntree_limit` is deprecated, use `iteration_range` instead.
## [15:55:37] WARNING: src/c_api/c_api.cc:935: `ntree_limit` is deprecated, use `iteration_range` instead.
## [15:55:38] WARNING: src/c_api/c_api.cc:935: `ntree_limit` is deprecated, use `iteration_range` instead.
## [15:55:38] WARNING: src/c_api/c_api.cc:935: `ntree_limit` is deprecated, use `iteration_range` instead.
## [15:55:38] WARNING: src/c_api/c_api.cc:935: `ntree_limit` is deprecated, use `iteration_range` instead.
## [15:55:38] WARNING: src/c_api/c_api.cc:935: `ntree_limit` is deprecated, use `iteration_range` instead.
## [15:55:38] WARNING: src/c_api/c_api.cc:935: `ntree_limit` is deprecated, use `iteration_range` instead.
## [15:55:38] WARNING: src/c_api/c_api.cc:935: `ntree_limit` is deprecated, use `iteration_range` instead.
## [15:55:38] WARNING: src/c_api/c_api.cc:935: `ntree_limit` is deprecated, use `iteration_range` instead.
## [15:55:38] WARNING: src/c_api/c_api.cc:935: `ntree_limit` is deprecated, use `iteration_range` instead.
## [15:55:38] WARNING: src/c_api/c_api.cc:935: `ntree_limit` is deprecated, use `iteration_range` instead.
## [15:55:38] WARNING: src/c_api/c_api.cc:935: `ntree_limit` is deprecated, use `iteration_range` instead.
## [15:55:38] WARNING: src/c_api/c_api.cc:935: `ntree_limit` is deprecated, use `iteration_range` instead.
## [15:55:38] WARNING: src/c_api/c_api.cc:935: `ntree_limit` is deprecated, use `iteration_range` instead.
## [15:55:38] WARNING: src/c_api/c_api.cc:935: `ntree_limit` is deprecated, use `iteration_range` instead.
## [15:55:38] WARNING: src/c_api/c_api.cc:935: `ntree_limit` is deprecated, use `iteration_range` instead.
## [15:55:39] WARNING: src/c_api/c_api.cc:935: `ntree_limit` is deprecated, use `iteration_range` instead.
## [15:55:39] WARNING: src/c_api/c_api.cc:935: `ntree_limit` is deprecated, use `iteration_range` instead.
## [15:55:39] WARNING: src/c_api/c_api.cc:935: `ntree_limit` is deprecated, use `iteration_range` instead.
## [15:55:39] WARNING: src/c_api/c_api.cc:935: `ntree_limit` is deprecated, use `iteration_range` instead.
## [15:55:39] WARNING: src/c_api/c_api.cc:935: `ntree_limit` is deprecated, use `iteration_range` instead.
## [15:55:39] WARNING: src/c_api/c_api.cc:935: `ntree_limit` is deprecated, use `iteration_range` instead.
## [15:55:39] WARNING: src/c_api/c_api.cc:935: `ntree_limit` is deprecated, use `iteration_range` instead.
## [15:55:39] WARNING: src/c_api/c_api.cc:935: `ntree_limit` is deprecated, use `iteration_range` instead.
## [15:55:39] WARNING: src/c_api/c_api.cc:935: `ntree_limit` is deprecated, use `iteration_range` instead.
## [15:55:39] WARNING: src/c_api/c_api.cc:935: `ntree_limit` is deprecated, use `iteration_range` instead.
## [15:55:40] WARNING: src/c_api/c_api.cc:935: `ntree_limit` is deprecated, use `iteration_range` instead.
## [15:55:40] WARNING: src/c_api/c_api.cc:935: `ntree_limit` is deprecated, use `iteration_range` instead.
## [15:55:40] WARNING: src/c_api/c_api.cc:935: `ntree_limit` is deprecated, use `iteration_range` instead.
## [15:55:40] WARNING: src/c_api/c_api.cc:935: `ntree_limit` is deprecated, use `iteration_range` instead.
## [15:55:40] WARNING: src/c_api/c_api.cc:935: `ntree_limit` is deprecated, use `iteration_range` instead.
## [15:55:40] WARNING: src/c_api/c_api.cc:935: `ntree_limit` is deprecated, use `iteration_range` instead.
## [15:55:40] WARNING: src/c_api/c_api.cc:935: `ntree_limit` is deprecated, use `iteration_range` instead.
## [15:55:40] WARNING: src/c_api/c_api.cc:935: `ntree_limit` is deprecated, use `iteration_range` instead.
## [15:55:40] WARNING: src/c_api/c_api.cc:935: `ntree_limit` is deprecated, use `iteration_range` instead.
## [15:55:40] WARNING: src/c_api/c_api.cc:935: `ntree_limit` is deprecated, use `iteration_range` instead.
## [15:55:41] WARNING: src/c_api/c_api.cc:935: `ntree_limit` is deprecated, use `iteration_range` instead.
## [15:55:41] WARNING: src/c_api/c_api.cc:935: `ntree_limit` is deprecated, use `iteration_range` instead.
## [15:55:41] WARNING: src/c_api/c_api.cc:935: `ntree_limit` is deprecated, use `iteration_range` instead.
## [15:55:41] WARNING: src/c_api/c_api.cc:935: `ntree_limit` is deprecated, use `iteration_range` instead.
## [15:55:41] WARNING: src/c_api/c_api.cc:935: `ntree_limit` is deprecated, use `iteration_range` instead.
## [15:55:41] WARNING: src/c_api/c_api.cc:935: `ntree_limit` is deprecated, use `iteration_range` instead.
## [15:55:41] WARNING: src/c_api/c_api.cc:935: `ntree_limit` is deprecated, use `iteration_range` instead.
## [15:55:41] WARNING: src/c_api/c_api.cc:935: `ntree_limit` is deprecated, use `iteration_range` instead.
## [15:55:41] WARNING: src/c_api/c_api.cc:935: `ntree_limit` is deprecated, use `iteration_range` instead.
## [15:55:41] WARNING: src/c_api/c_api.cc:935: `ntree_limit` is deprecated, use `iteration_range` instead.
## [15:55:41] WARNING: src/c_api/c_api.cc:935: `ntree_limit` is deprecated, use `iteration_range` instead.
## [15:55:41] WARNING: src/c_api/c_api.cc:935: `ntree_limit` is deprecated, use `iteration_range` instead.
## [15:55:42] WARNING: src/c_api/c_api.cc:935: `ntree_limit` is deprecated, use `iteration_range` instead.
## [15:55:42] WARNING: src/c_api/c_api.cc:935: `ntree_limit` is deprecated, use `iteration_range` instead.
## [15:55:42] WARNING: src/c_api/c_api.cc:935: `ntree_limit` is deprecated, use `iteration_range` instead.
## [15:55:42] WARNING: src/c_api/c_api.cc:935: `ntree_limit` is deprecated, use `iteration_range` instead.
## [15:55:42] WARNING: src/c_api/c_api.cc:935: `ntree_limit` is deprecated, use `iteration_range` instead.
## [15:55:42] WARNING: src/c_api/c_api.cc:935: `ntree_limit` is deprecated, use `iteration_range` instead.
## [15:55:42] WARNING: src/c_api/c_api.cc:935: `ntree_limit` is deprecated, use `iteration_range` instead.
## [15:55:42] WARNING: src/c_api/c_api.cc:935: `ntree_limit` is deprecated, use `iteration_range` instead.
## [15:55:42] WARNING: src/c_api/c_api.cc:935: `ntree_limit` is deprecated, use `iteration_range` instead.
## [15:55:42] WARNING: src/c_api/c_api.cc:935: `ntree_limit` is deprecated, use `iteration_range` instead.
## [15:55:42] WARNING: src/c_api/c_api.cc:935: `ntree_limit` is deprecated, use `iteration_range` instead.
## [15:55:42] WARNING: src/c_api/c_api.cc:935: `ntree_limit` is deprecated, use `iteration_range` instead.
## [15:55:43] WARNING: src/c_api/c_api.cc:935: `ntree_limit` is deprecated, use `iteration_range` instead.
## [15:55:43] WARNING: src/c_api/c_api.cc:935: `ntree_limit` is deprecated, use `iteration_range` instead.
## [15:55:43] WARNING: src/c_api/c_api.cc:935: `ntree_limit` is deprecated, use `iteration_range` instead.
## [15:55:43] WARNING: src/c_api/c_api.cc:935: `ntree_limit` is deprecated, use `iteration_range` instead.
## [15:55:43] WARNING: src/c_api/c_api.cc:935: `ntree_limit` is deprecated, use `iteration_range` instead.
## [15:55:43] WARNING: src/c_api/c_api.cc:935: `ntree_limit` is deprecated, use `iteration_range` instead.
## [15:55:43] WARNING: src/c_api/c_api.cc:935: `ntree_limit` is deprecated, use `iteration_range` instead.
## [15:55:43] WARNING: src/c_api/c_api.cc:935: `ntree_limit` is deprecated, use `iteration_range` instead.
## [15:55:44] WARNING: src/c_api/c_api.cc:935: `ntree_limit` is deprecated, use `iteration_range` instead.
## [15:55:44] WARNING: src/c_api/c_api.cc:935: `ntree_limit` is deprecated, use `iteration_range` instead.
## [15:55:44] WARNING: src/c_api/c_api.cc:935: `ntree_limit` is deprecated, use `iteration_range` instead.
## [15:55:44] WARNING: src/c_api/c_api.cc:935: `ntree_limit` is deprecated, use `iteration_range` instead.
## [15:55:44] WARNING: src/c_api/c_api.cc:935: `ntree_limit` is deprecated, use `iteration_range` instead.
## [15:55:44] WARNING: src/c_api/c_api.cc:935: `ntree_limit` is deprecated, use `iteration_range` instead.
## [15:55:44] WARNING: src/c_api/c_api.cc:935: `ntree_limit` is deprecated, use `iteration_range` instead.
## [15:55:44] WARNING: src/c_api/c_api.cc:935: `ntree_limit` is deprecated, use `iteration_range` instead.
## [15:55:44] WARNING: src/c_api/c_api.cc:935: `ntree_limit` is deprecated, use `iteration_range` instead.
## [15:55:44] WARNING: src/c_api/c_api.cc:935: `ntree_limit` is deprecated, use `iteration_range` instead.
## [15:55:44] WARNING: src/c_api/c_api.cc:935: `ntree_limit` is deprecated, use `iteration_range` instead.
## [15:55:44] WARNING: src/c_api/c_api.cc:935: `ntree_limit` is deprecated, use `iteration_range` instead.
## [15:55:45] WARNING: src/c_api/c_api.cc:935: `ntree_limit` is deprecated, use `iteration_range` instead.
## [15:55:45] WARNING: src/c_api/c_api.cc:935: `ntree_limit` is deprecated, use `iteration_range` instead.
## [15:55:45] WARNING: src/c_api/c_api.cc:935: `ntree_limit` is deprecated, use `iteration_range` instead.
## [15:55:45] WARNING: src/c_api/c_api.cc:935: `ntree_limit` is deprecated, use `iteration_range` instead.
## [15:55:45] WARNING: src/c_api/c_api.cc:935: `ntree_limit` is deprecated, use `iteration_range` instead.
## [15:55:45] WARNING: src/c_api/c_api.cc:935: `ntree_limit` is deprecated, use `iteration_range` instead.
## [15:55:45] WARNING: src/c_api/c_api.cc:935: `ntree_limit` is deprecated, use `iteration_range` instead.
## [15:55:45] WARNING: src/c_api/c_api.cc:935: `ntree_limit` is deprecated, use `iteration_range` instead.
## [15:55:45] WARNING: src/c_api/c_api.cc:935: `ntree_limit` is deprecated, use `iteration_range` instead.
## [15:55:45] WARNING: src/c_api/c_api.cc:935: `ntree_limit` is deprecated, use `iteration_range` instead.
## [15:55:45] WARNING: src/c_api/c_api.cc:935: `ntree_limit` is deprecated, use `iteration_range` instead.
## [15:55:45] WARNING: src/c_api/c_api.cc:935: `ntree_limit` is deprecated, use `iteration_range` instead.
## [15:55:46] WARNING: src/c_api/c_api.cc:935: `ntree_limit` is deprecated, use `iteration_range` instead.
## [15:55:46] WARNING: src/c_api/c_api.cc:935: `ntree_limit` is deprecated, use `iteration_range` instead.
## [15:55:46] WARNING: src/c_api/c_api.cc:935: `ntree_limit` is deprecated, use `iteration_range` instead.
## [15:55:46] WARNING: src/c_api/c_api.cc:935: `ntree_limit` is deprecated, use `iteration_range` instead.
## [15:55:46] WARNING: src/c_api/c_api.cc:935: `ntree_limit` is deprecated, use `iteration_range` instead.
## [15:55:46] WARNING: src/c_api/c_api.cc:935: `ntree_limit` is deprecated, use `iteration_range` instead.
## [15:55:46] WARNING: src/c_api/c_api.cc:935: `ntree_limit` is deprecated, use `iteration_range` instead.
## [15:55:46] WARNING: src/c_api/c_api.cc:935: `ntree_limit` is deprecated, use `iteration_range` instead.
## [15:55:46] WARNING: src/c_api/c_api.cc:935: `ntree_limit` is deprecated, use `iteration_range` instead.
## [15:55:46] WARNING: src/c_api/c_api.cc:935: `ntree_limit` is deprecated, use `iteration_range` instead.
## [15:55:47] WARNING: src/c_api/c_api.cc:935: `ntree_limit` is deprecated, use `iteration_range` instead.
## [15:55:47] WARNING: src/c_api/c_api.cc:935: `ntree_limit` is deprecated, use `iteration_range` instead.
## [15:55:47] WARNING: src/c_api/c_api.cc:935: `ntree_limit` is deprecated, use `iteration_range` instead.
## [15:55:47] WARNING: src/c_api/c_api.cc:935: `ntree_limit` is deprecated, use `iteration_range` instead.
## [15:55:47] WARNING: src/c_api/c_api.cc:935: `ntree_limit` is deprecated, use `iteration_range` instead.
## [15:55:47] WARNING: src/c_api/c_api.cc:935: `ntree_limit` is deprecated, use `iteration_range` instead.
## [15:55:47] WARNING: src/c_api/c_api.cc:935: `ntree_limit` is deprecated, use `iteration_range` instead.
## [15:55:47] WARNING: src/c_api/c_api.cc:935: `ntree_limit` is deprecated, use `iteration_range` instead.
## [15:55:47] WARNING: src/c_api/c_api.cc:935: `ntree_limit` is deprecated, use `iteration_range` instead.
## [15:55:47] WARNING: src/c_api/c_api.cc:935: `ntree_limit` is deprecated, use `iteration_range` instead.
## [15:55:48] WARNING: src/c_api/c_api.cc:935: `ntree_limit` is deprecated, use `iteration_range` instead.
## [15:55:48] WARNING: src/c_api/c_api.cc:935: `ntree_limit` is deprecated, use `iteration_range` instead.
## [15:55:48] WARNING: src/c_api/c_api.cc:935: `ntree_limit` is deprecated, use `iteration_range` instead.
## [15:55:48] WARNING: src/c_api/c_api.cc:935: `ntree_limit` is deprecated, use `iteration_range` instead.
## [15:55:48] WARNING: src/c_api/c_api.cc:935: `ntree_limit` is deprecated, use `iteration_range` instead.
## [15:55:48] WARNING: src/c_api/c_api.cc:935: `ntree_limit` is deprecated, use `iteration_range` instead.
## [15:55:48] WARNING: src/c_api/c_api.cc:935: `ntree_limit` is deprecated, use `iteration_range` instead.
## [15:55:48] WARNING: src/c_api/c_api.cc:935: `ntree_limit` is deprecated, use `iteration_range` instead.
## [15:55:48] WARNING: src/c_api/c_api.cc:935: `ntree_limit` is deprecated, use `iteration_range` instead.
## [15:55:48] WARNING: src/c_api/c_api.cc:935: `ntree_limit` is deprecated, use `iteration_range` instead.
## [15:55:48] WARNING: src/c_api/c_api.cc:935: `ntree_limit` is deprecated, use `iteration_range` instead.
## [15:55:48] WARNING: src/c_api/c_api.cc:935: `ntree_limit` is deprecated, use `iteration_range` instead.
## [15:55:48] WARNING: src/c_api/c_api.cc:935: `ntree_limit` is deprecated, use `iteration_range` instead.
## [15:55:48] WARNING: src/c_api/c_api.cc:935: `ntree_limit` is deprecated, use `iteration_range` instead.
## [15:55:49] WARNING: src/c_api/c_api.cc:935: `ntree_limit` is deprecated, use `iteration_range` instead.
## [15:55:49] WARNING: src/c_api/c_api.cc:935: `ntree_limit` is deprecated, use `iteration_range` instead.
## [15:55:49] WARNING: src/c_api/c_api.cc:935: `ntree_limit` is deprecated, use `iteration_range` instead.
## [15:55:49] WARNING: src/c_api/c_api.cc:935: `ntree_limit` is deprecated, use `iteration_range` instead.
## [15:55:49] WARNING: src/c_api/c_api.cc:935: `ntree_limit` is deprecated, use `iteration_range` instead.
## [15:55:49] WARNING: src/c_api/c_api.cc:935: `ntree_limit` is deprecated, use `iteration_range` instead.
## [15:55:49] WARNING: src/c_api/c_api.cc:935: `ntree_limit` is deprecated, use `iteration_range` instead.
## [15:55:49] WARNING: src/c_api/c_api.cc:935: `ntree_limit` is deprecated, use `iteration_range` instead.
## [15:55:49] WARNING: src/c_api/c_api.cc:935: `ntree_limit` is deprecated, use `iteration_range` instead.
## [15:55:49] WARNING: src/c_api/c_api.cc:935: `ntree_limit` is deprecated, use `iteration_range` instead.
## [15:55:49] WARNING: src/c_api/c_api.cc:935: `ntree_limit` is deprecated, use `iteration_range` instead.
## [15:55:49] WARNING: src/c_api/c_api.cc:935: `ntree_limit` is deprecated, use `iteration_range` instead.
## [15:55:50] WARNING: src/c_api/c_api.cc:935: `ntree_limit` is deprecated, use `iteration_range` instead.
## [15:55:50] WARNING: src/c_api/c_api.cc:935: `ntree_limit` is deprecated, use `iteration_range` instead.
## [15:55:50] WARNING: src/c_api/c_api.cc:935: `ntree_limit` is deprecated, use `iteration_range` instead.
## [15:55:50] WARNING: src/c_api/c_api.cc:935: `ntree_limit` is deprecated, use `iteration_range` instead.
## [15:55:50] WARNING: src/c_api/c_api.cc:935: `ntree_limit` is deprecated, use `iteration_range` instead.
## [15:55:50] WARNING: src/c_api/c_api.cc:935: `ntree_limit` is deprecated, use `iteration_range` instead.
## [15:55:50] WARNING: src/c_api/c_api.cc:935: `ntree_limit` is deprecated, use `iteration_range` instead.
## [15:55:50] WARNING: src/c_api/c_api.cc:935: `ntree_limit` is deprecated, use `iteration_range` instead.
## [15:55:50] WARNING: src/c_api/c_api.cc:935: `ntree_limit` is deprecated, use `iteration_range` instead.
## [15:55:50] WARNING: src/c_api/c_api.cc:935: `ntree_limit` is deprecated, use `iteration_range` instead.
## [15:55:53] WARNING: src/c_api/c_api.cc:935: `ntree_limit` is deprecated, use `iteration_range` instead.
## [15:55:53] WARNING: src/c_api/c_api.cc:935: `ntree_limit` is deprecated, use `iteration_range` instead.
## [15:55:53] WARNING: src/c_api/c_api.cc:935: `ntree_limit` is deprecated, use `iteration_range` instead.
## [15:55:53] WARNING: src/c_api/c_api.cc:935: `ntree_limit` is deprecated, use `iteration_range` instead.
## [15:55:54] WARNING: src/c_api/c_api.cc:935: `ntree_limit` is deprecated, use `iteration_range` instead.
## [15:55:54] WARNING: src/c_api/c_api.cc:935: `ntree_limit` is deprecated, use `iteration_range` instead.
## [15:55:54] WARNING: src/c_api/c_api.cc:935: `ntree_limit` is deprecated, use `iteration_range` instead.
## [15:55:54] WARNING: src/c_api/c_api.cc:935: `ntree_limit` is deprecated, use `iteration_range` instead.
## [15:55:54] WARNING: src/c_api/c_api.cc:935: `ntree_limit` is deprecated, use `iteration_range` instead.
## [15:55:54] WARNING: src/c_api/c_api.cc:935: `ntree_limit` is deprecated, use `iteration_range` instead.
## [15:55:54] WARNING: src/c_api/c_api.cc:935: `ntree_limit` is deprecated, use `iteration_range` instead.
## [15:55:54] WARNING: src/c_api/c_api.cc:935: `ntree_limit` is deprecated, use `iteration_range` instead.
## [15:55:54] WARNING: src/c_api/c_api.cc:935: `ntree_limit` is deprecated, use `iteration_range` instead.
## [15:55:54] WARNING: src/c_api/c_api.cc:935: `ntree_limit` is deprecated, use `iteration_range` instead.
## [15:55:54] WARNING: src/c_api/c_api.cc:935: `ntree_limit` is deprecated, use `iteration_range` instead.
## [15:55:54] WARNING: src/c_api/c_api.cc:935: `ntree_limit` is deprecated, use `iteration_range` instead.
## [15:55:54] WARNING: src/c_api/c_api.cc:935: `ntree_limit` is deprecated, use `iteration_range` instead.
## [15:55:54] WARNING: src/c_api/c_api.cc:935: `ntree_limit` is deprecated, use `iteration_range` instead.
## [15:55:55] WARNING: src/c_api/c_api.cc:935: `ntree_limit` is deprecated, use `iteration_range` instead.
## [15:55:55] WARNING: src/c_api/c_api.cc:935: `ntree_limit` is deprecated, use `iteration_range` instead.
## [15:55:55] WARNING: src/c_api/c_api.cc:935: `ntree_limit` is deprecated, use `iteration_range` instead.
## [15:55:55] WARNING: src/c_api/c_api.cc:935: `ntree_limit` is deprecated, use `iteration_range` instead.
## [15:55:55] WARNING: src/c_api/c_api.cc:935: `ntree_limit` is deprecated, use `iteration_range` instead.
## [15:55:55] WARNING: src/c_api/c_api.cc:935: `ntree_limit` is deprecated, use `iteration_range` instead.
## [15:55:55] WARNING: src/c_api/c_api.cc:935: `ntree_limit` is deprecated, use `iteration_range` instead.
## [15:55:55] WARNING: src/c_api/c_api.cc:935: `ntree_limit` is deprecated, use `iteration_range` instead.
## [15:55:56] WARNING: src/c_api/c_api.cc:935: `ntree_limit` is deprecated, use `iteration_range` instead.
## [15:55:56] WARNING: src/c_api/c_api.cc:935: `ntree_limit` is deprecated, use `iteration_range` instead.
## [15:55:56] WARNING: src/c_api/c_api.cc:935: `ntree_limit` is deprecated, use `iteration_range` instead.
## [15:55:56] WARNING: src/c_api/c_api.cc:935: `ntree_limit` is deprecated, use `iteration_range` instead.
## [15:55:56] WARNING: src/c_api/c_api.cc:935: `ntree_limit` is deprecated, use `iteration_range` instead.
## [15:55:56] WARNING: src/c_api/c_api.cc:935: `ntree_limit` is deprecated, use `iteration_range` instead.
## [15:55:56] WARNING: src/c_api/c_api.cc:935: `ntree_limit` is deprecated, use `iteration_range` instead.
## [15:55:56] WARNING: src/c_api/c_api.cc:935: `ntree_limit` is deprecated, use `iteration_range` instead.
## [15:55:57] WARNING: src/c_api/c_api.cc:935: `ntree_limit` is deprecated, use `iteration_range` instead.
## [15:55:57] WARNING: src/c_api/c_api.cc:935: `ntree_limit` is deprecated, use `iteration_range` instead.
## [15:55:57] WARNING: src/c_api/c_api.cc:935: `ntree_limit` is deprecated, use `iteration_range` instead.
## [15:55:57] WARNING: src/c_api/c_api.cc:935: `ntree_limit` is deprecated, use `iteration_range` instead.
## [15:55:57] WARNING: src/c_api/c_api.cc:935: `ntree_limit` is deprecated, use `iteration_range` instead.
## [15:55:57] WARNING: src/c_api/c_api.cc:935: `ntree_limit` is deprecated, use `iteration_range` instead.
## [15:55:57] WARNING: src/c_api/c_api.cc:935: `ntree_limit` is deprecated, use `iteration_range` instead.
## [15:55:57] WARNING: src/c_api/c_api.cc:935: `ntree_limit` is deprecated, use `iteration_range` instead.
## [15:55:57] WARNING: src/c_api/c_api.cc:935: `ntree_limit` is deprecated, use `iteration_range` instead.
## [15:55:57] WARNING: src/c_api/c_api.cc:935: `ntree_limit` is deprecated, use `iteration_range` instead.
## [15:55:57] WARNING: src/c_api/c_api.cc:935: `ntree_limit` is deprecated, use `iteration_range` instead.
## [15:55:57] WARNING: src/c_api/c_api.cc:935: `ntree_limit` is deprecated, use `iteration_range` instead.
## [15:55:57] WARNING: src/c_api/c_api.cc:935: `ntree_limit` is deprecated, use `iteration_range` instead.
## [15:55:57] WARNING: src/c_api/c_api.cc:935: `ntree_limit` is deprecated, use `iteration_range` instead.
## [15:55:58] WARNING: src/c_api/c_api.cc:935: `ntree_limit` is deprecated, use `iteration_range` instead.
## [15:55:58] WARNING: src/c_api/c_api.cc:935: `ntree_limit` is deprecated, use `iteration_range` instead.
## [15:55:58] WARNING: src/c_api/c_api.cc:935: `ntree_limit` is deprecated, use `iteration_range` instead.
## [15:55:58] WARNING: src/c_api/c_api.cc:935: `ntree_limit` is deprecated, use `iteration_range` instead.
## [15:55:58] WARNING: src/c_api/c_api.cc:935: `ntree_limit` is deprecated, use `iteration_range` instead.
## [15:55:58] WARNING: src/c_api/c_api.cc:935: `ntree_limit` is deprecated, use `iteration_range` instead.
## [15:55:58] WARNING: src/c_api/c_api.cc:935: `ntree_limit` is deprecated, use `iteration_range` instead.
## [15:55:58] WARNING: src/c_api/c_api.cc:935: `ntree_limit` is deprecated, use `iteration_range` instead.
## [15:55:58] WARNING: src/c_api/c_api.cc:935: `ntree_limit` is deprecated, use `iteration_range` instead.
## [15:55:58] WARNING: src/c_api/c_api.cc:935: `ntree_limit` is deprecated, use `iteration_range` instead.
## [15:55:58] WARNING: src/c_api/c_api.cc:935: `ntree_limit` is deprecated, use `iteration_range` instead.
## [15:55:58] WARNING: src/c_api/c_api.cc:935: `ntree_limit` is deprecated, use `iteration_range` instead.
## [15:55:59] WARNING: src/c_api/c_api.cc:935: `ntree_limit` is deprecated, use `iteration_range` instead.
## [15:55:59] WARNING: src/c_api/c_api.cc:935: `ntree_limit` is deprecated, use `iteration_range` instead.
## [15:55:59] WARNING: src/c_api/c_api.cc:935: `ntree_limit` is deprecated, use `iteration_range` instead.
## [15:55:59] WARNING: src/c_api/c_api.cc:935: `ntree_limit` is deprecated, use `iteration_range` instead.
## [15:55:59] WARNING: src/c_api/c_api.cc:935: `ntree_limit` is deprecated, use `iteration_range` instead.
## [15:55:59] WARNING: src/c_api/c_api.cc:935: `ntree_limit` is deprecated, use `iteration_range` instead.
## [15:55:59] WARNING: src/c_api/c_api.cc:935: `ntree_limit` is deprecated, use `iteration_range` instead.
## [15:55:59] WARNING: src/c_api/c_api.cc:935: `ntree_limit` is deprecated, use `iteration_range` instead.
## [15:56:00] WARNING: src/c_api/c_api.cc:935: `ntree_limit` is deprecated, use `iteration_range` instead.
## [15:56:00] WARNING: src/c_api/c_api.cc:935: `ntree_limit` is deprecated, use `iteration_range` instead.
## [15:56:00] WARNING: src/c_api/c_api.cc:935: `ntree_limit` is deprecated, use `iteration_range` instead.
## [15:56:00] WARNING: src/c_api/c_api.cc:935: `ntree_limit` is deprecated, use `iteration_range` instead.
## [15:56:00] WARNING: src/c_api/c_api.cc:935: `ntree_limit` is deprecated, use `iteration_range` instead.
## [15:56:00] WARNING: src/c_api/c_api.cc:935: `ntree_limit` is deprecated, use `iteration_range` instead.
## [15:56:00] WARNING: src/c_api/c_api.cc:935: `ntree_limit` is deprecated, use `iteration_range` instead.
## [15:56:00] WARNING: src/c_api/c_api.cc:935: `ntree_limit` is deprecated, use `iteration_range` instead.
## [15:56:00] WARNING: src/c_api/c_api.cc:935: `ntree_limit` is deprecated, use `iteration_range` instead.
## [15:56:00] WARNING: src/c_api/c_api.cc:935: `ntree_limit` is deprecated, use `iteration_range` instead.
## [15:56:00] WARNING: src/c_api/c_api.cc:935: `ntree_limit` is deprecated, use `iteration_range` instead.
## [15:56:00] WARNING: src/c_api/c_api.cc:935: `ntree_limit` is deprecated, use `iteration_range` instead.
## [15:56:00] WARNING: src/c_api/c_api.cc:935: `ntree_limit` is deprecated, use `iteration_range` instead.
## [15:56:00] WARNING: src/c_api/c_api.cc:935: `ntree_limit` is deprecated, use `iteration_range` instead.
## [15:56:00] WARNING: src/c_api/c_api.cc:935: `ntree_limit` is deprecated, use `iteration_range` instead.
## [15:56:00] WARNING: src/c_api/c_api.cc:935: `ntree_limit` is deprecated, use `iteration_range` instead.
## [15:56:01] WARNING: src/c_api/c_api.cc:935: `ntree_limit` is deprecated, use `iteration_range` instead.
## [15:56:01] WARNING: src/c_api/c_api.cc:935: `ntree_limit` is deprecated, use `iteration_range` instead.
## [15:56:01] WARNING: src/c_api/c_api.cc:935: `ntree_limit` is deprecated, use `iteration_range` instead.
## [15:56:01] WARNING: src/c_api/c_api.cc:935: `ntree_limit` is deprecated, use `iteration_range` instead.
## [15:56:01] WARNING: src/c_api/c_api.cc:935: `ntree_limit` is deprecated, use `iteration_range` instead.
## [15:56:01] WARNING: src/c_api/c_api.cc:935: `ntree_limit` is deprecated, use `iteration_range` instead.
## [15:56:01] WARNING: src/c_api/c_api.cc:935: `ntree_limit` is deprecated, use `iteration_range` instead.
## [15:56:01] WARNING: src/c_api/c_api.cc:935: `ntree_limit` is deprecated, use `iteration_range` instead.
## [15:56:01] WARNING: src/c_api/c_api.cc:935: `ntree_limit` is deprecated, use `iteration_range` instead.
## [15:56:01] WARNING: src/c_api/c_api.cc:935: `ntree_limit` is deprecated, use `iteration_range` instead.
## [15:56:02] WARNING: src/c_api/c_api.cc:935: `ntree_limit` is deprecated, use `iteration_range` instead.
## [15:56:02] WARNING: src/c_api/c_api.cc:935: `ntree_limit` is deprecated, use `iteration_range` instead.
## [15:56:02] WARNING: src/c_api/c_api.cc:935: `ntree_limit` is deprecated, use `iteration_range` instead.
## [15:56:02] WARNING: src/c_api/c_api.cc:935: `ntree_limit` is deprecated, use `iteration_range` instead.
## [15:56:02] WARNING: src/c_api/c_api.cc:935: `ntree_limit` is deprecated, use `iteration_range` instead.
## [15:56:02] WARNING: src/c_api/c_api.cc:935: `ntree_limit` is deprecated, use `iteration_range` instead.
## [15:56:02] WARNING: src/c_api/c_api.cc:935: `ntree_limit` is deprecated, use `iteration_range` instead.
## [15:56:02] WARNING: src/c_api/c_api.cc:935: `ntree_limit` is deprecated, use `iteration_range` instead.
## [15:56:03] WARNING: src/c_api/c_api.cc:935: `ntree_limit` is deprecated, use `iteration_range` instead.
## [15:56:03] WARNING: src/c_api/c_api.cc:935: `ntree_limit` is deprecated, use `iteration_range` instead.
## [15:56:03] WARNING: src/c_api/c_api.cc:935: `ntree_limit` is deprecated, use `iteration_range` instead.
## [15:56:03] WARNING: src/c_api/c_api.cc:935: `ntree_limit` is deprecated, use `iteration_range` instead.
## [15:56:03] WARNING: src/c_api/c_api.cc:935: `ntree_limit` is deprecated, use `iteration_range` instead.
## [15:56:03] WARNING: src/c_api/c_api.cc:935: `ntree_limit` is deprecated, use `iteration_range` instead.
## [15:56:03] WARNING: src/c_api/c_api.cc:935: `ntree_limit` is deprecated, use `iteration_range` instead.
## [15:56:03] WARNING: src/c_api/c_api.cc:935: `ntree_limit` is deprecated, use `iteration_range` instead.
## [15:56:03] WARNING: src/c_api/c_api.cc:935: `ntree_limit` is deprecated, use `iteration_range` instead.
## [15:56:03] WARNING: src/c_api/c_api.cc:935: `ntree_limit` is deprecated, use `iteration_range` instead.
## [15:56:03] WARNING: src/c_api/c_api.cc:935: `ntree_limit` is deprecated, use `iteration_range` instead.
## [15:56:03] WARNING: src/c_api/c_api.cc:935: `ntree_limit` is deprecated, use `iteration_range` instead.
## [15:56:04] WARNING: src/c_api/c_api.cc:935: `ntree_limit` is deprecated, use `iteration_range` instead.
## [15:56:04] WARNING: src/c_api/c_api.cc:935: `ntree_limit` is deprecated, use `iteration_range` instead.
## [15:56:04] WARNING: src/c_api/c_api.cc:935: `ntree_limit` is deprecated, use `iteration_range` instead.
## [15:56:04] WARNING: src/c_api/c_api.cc:935: `ntree_limit` is deprecated, use `iteration_range` instead.
## [15:56:04] WARNING: src/c_api/c_api.cc:935: `ntree_limit` is deprecated, use `iteration_range` instead.
## [15:56:04] WARNING: src/c_api/c_api.cc:935: `ntree_limit` is deprecated, use `iteration_range` instead.
## [15:56:04] WARNING: src/c_api/c_api.cc:935: `ntree_limit` is deprecated, use `iteration_range` instead.
## [15:56:04] WARNING: src/c_api/c_api.cc:935: `ntree_limit` is deprecated, use `iteration_range` instead.
## [15:56:04] WARNING: src/c_api/c_api.cc:935: `ntree_limit` is deprecated, use `iteration_range` instead.
## [15:56:04] WARNING: src/c_api/c_api.cc:935: `ntree_limit` is deprecated, use `iteration_range` instead.
## [15:56:04] WARNING: src/c_api/c_api.cc:935: `ntree_limit` is deprecated, use `iteration_range` instead.
## [15:56:04] WARNING: src/c_api/c_api.cc:935: `ntree_limit` is deprecated, use `iteration_range` instead.
## [15:56:05] WARNING: src/c_api/c_api.cc:935: `ntree_limit` is deprecated, use `iteration_range` instead.
## [15:56:05] WARNING: src/c_api/c_api.cc:935: `ntree_limit` is deprecated, use `iteration_range` instead.
## [15:56:05] WARNING: src/c_api/c_api.cc:935: `ntree_limit` is deprecated, use `iteration_range` instead.
## [15:56:05] WARNING: src/c_api/c_api.cc:935: `ntree_limit` is deprecated, use `iteration_range` instead.
## [15:56:05] WARNING: src/c_api/c_api.cc:935: `ntree_limit` is deprecated, use `iteration_range` instead.
## [15:56:05] WARNING: src/c_api/c_api.cc:935: `ntree_limit` is deprecated, use `iteration_range` instead.
## [15:56:05] WARNING: src/c_api/c_api.cc:935: `ntree_limit` is deprecated, use `iteration_range` instead.
## [15:56:05] WARNING: src/c_api/c_api.cc:935: `ntree_limit` is deprecated, use `iteration_range` instead.
## [15:56:05] WARNING: src/c_api/c_api.cc:935: `ntree_limit` is deprecated, use `iteration_range` instead.
## [15:56:05] WARNING: src/c_api/c_api.cc:935: `ntree_limit` is deprecated, use `iteration_range` instead.
## [15:56:06] WARNING: src/c_api/c_api.cc:935: `ntree_limit` is deprecated, use `iteration_range` instead.
## [15:56:06] WARNING: src/c_api/c_api.cc:935: `ntree_limit` is deprecated, use `iteration_range` instead.
## [15:56:06] WARNING: src/c_api/c_api.cc:935: `ntree_limit` is deprecated, use `iteration_range` instead.
## [15:56:06] WARNING: src/c_api/c_api.cc:935: `ntree_limit` is deprecated, use `iteration_range` instead.
## [15:56:06] WARNING: src/c_api/c_api.cc:935: `ntree_limit` is deprecated, use `iteration_range` instead.
## [15:56:06] WARNING: src/c_api/c_api.cc:935: `ntree_limit` is deprecated, use `iteration_range` instead.
## [15:56:06] WARNING: src/c_api/c_api.cc:935: `ntree_limit` is deprecated, use `iteration_range` instead.
## [15:56:06] WARNING: src/c_api/c_api.cc:935: `ntree_limit` is deprecated, use `iteration_range` instead.
## [15:56:06] WARNING: src/c_api/c_api.cc:935: `ntree_limit` is deprecated, use `iteration_range` instead.
## [15:56:06] WARNING: src/c_api/c_api.cc:935: `ntree_limit` is deprecated, use `iteration_range` instead.
## [15:56:06] WARNING: src/c_api/c_api.cc:935: `ntree_limit` is deprecated, use `iteration_range` instead.
## [15:56:06] WARNING: src/c_api/c_api.cc:935: `ntree_limit` is deprecated, use `iteration_range` instead.
## [15:56:07] WARNING: src/c_api/c_api.cc:935: `ntree_limit` is deprecated, use `iteration_range` instead.
## [15:56:07] WARNING: src/c_api/c_api.cc:935: `ntree_limit` is deprecated, use `iteration_range` instead.
## [15:56:07] WARNING: src/c_api/c_api.cc:935: `ntree_limit` is deprecated, use `iteration_range` instead.
## [15:56:07] WARNING: src/c_api/c_api.cc:935: `ntree_limit` is deprecated, use `iteration_range` instead.
## [15:56:07] WARNING: src/c_api/c_api.cc:935: `ntree_limit` is deprecated, use `iteration_range` instead.
## [15:56:07] WARNING: src/c_api/c_api.cc:935: `ntree_limit` is deprecated, use `iteration_range` instead.
## [15:56:07] WARNING: src/c_api/c_api.cc:935: `ntree_limit` is deprecated, use `iteration_range` instead.
## [15:56:07] WARNING: src/c_api/c_api.cc:935: `ntree_limit` is deprecated, use `iteration_range` instead.
## [15:56:07] WARNING: src/c_api/c_api.cc:935: `ntree_limit` is deprecated, use `iteration_range` instead.
## [15:56:07] WARNING: src/c_api/c_api.cc:935: `ntree_limit` is deprecated, use `iteration_range` instead.
## [15:56:07] WARNING: src/c_api/c_api.cc:935: `ntree_limit` is deprecated, use `iteration_range` instead.
## [15:56:07] WARNING: src/c_api/c_api.cc:935: `ntree_limit` is deprecated, use `iteration_range` instead.
## [15:56:08] WARNING: src/c_api/c_api.cc:935: `ntree_limit` is deprecated, use `iteration_range` instead.
## [15:56:08] WARNING: src/c_api/c_api.cc:935: `ntree_limit` is deprecated, use `iteration_range` instead.
## [15:56:08] WARNING: src/c_api/c_api.cc:935: `ntree_limit` is deprecated, use `iteration_range` instead.
## [15:56:08] WARNING: src/c_api/c_api.cc:935: `ntree_limit` is deprecated, use `iteration_range` instead.
## [15:56:08] WARNING: src/c_api/c_api.cc:935: `ntree_limit` is deprecated, use `iteration_range` instead.
## [15:56:08] WARNING: src/c_api/c_api.cc:935: `ntree_limit` is deprecated, use `iteration_range` instead.
## [15:56:08] WARNING: src/c_api/c_api.cc:935: `ntree_limit` is deprecated, use `iteration_range` instead.
## [15:56:08] WARNING: src/c_api/c_api.cc:935: `ntree_limit` is deprecated, use `iteration_range` instead.
## [15:56:08] WARNING: src/c_api/c_api.cc:935: `ntree_limit` is deprecated, use `iteration_range` instead.
## [15:56:08] WARNING: src/c_api/c_api.cc:935: `ntree_limit` is deprecated, use `iteration_range` instead.
## [15:56:09] WARNING: src/c_api/c_api.cc:935: `ntree_limit` is deprecated, use `iteration_range` instead.
## [15:56:09] WARNING: src/c_api/c_api.cc:935: `ntree_limit` is deprecated, use `iteration_range` instead.
## [15:56:09] WARNING: src/c_api/c_api.cc:935: `ntree_limit` is deprecated, use `iteration_range` instead.
## [15:56:09] WARNING: src/c_api/c_api.cc:935: `ntree_limit` is deprecated, use `iteration_range` instead.
## [15:56:09] WARNING: src/c_api/c_api.cc:935: `ntree_limit` is deprecated, use `iteration_range` instead.
## [15:56:09] WARNING: src/c_api/c_api.cc:935: `ntree_limit` is deprecated, use `iteration_range` instead.
## [15:56:09] WARNING: src/c_api/c_api.cc:935: `ntree_limit` is deprecated, use `iteration_range` instead.
## [15:56:09] WARNING: src/c_api/c_api.cc:935: `ntree_limit` is deprecated, use `iteration_range` instead.
## [15:56:10] WARNING: src/c_api/c_api.cc:935: `ntree_limit` is deprecated, use `iteration_range` instead.
## [15:56:10] WARNING: src/c_api/c_api.cc:935: `ntree_limit` is deprecated, use `iteration_range` instead.
## [15:56:10] WARNING: src/c_api/c_api.cc:935: `ntree_limit` is deprecated, use `iteration_range` instead.
## [15:56:10] WARNING: src/c_api/c_api.cc:935: `ntree_limit` is deprecated, use `iteration_range` instead.
## [15:56:10] WARNING: src/c_api/c_api.cc:935: `ntree_limit` is deprecated, use `iteration_range` instead.
## [15:56:10] WARNING: src/c_api/c_api.cc:935: `ntree_limit` is deprecated, use `iteration_range` instead.
## [15:56:10] WARNING: src/c_api/c_api.cc:935: `ntree_limit` is deprecated, use `iteration_range` instead.
## [15:56:10] WARNING: src/c_api/c_api.cc:935: `ntree_limit` is deprecated, use `iteration_range` instead.
## [15:56:10] WARNING: src/c_api/c_api.cc:935: `ntree_limit` is deprecated, use `iteration_range` instead.
## [15:56:10] WARNING: src/c_api/c_api.cc:935: `ntree_limit` is deprecated, use `iteration_range` instead.
## [15:56:10] WARNING: src/c_api/c_api.cc:935: `ntree_limit` is deprecated, use `iteration_range` instead.
## [15:56:10] WARNING: src/c_api/c_api.cc:935: `ntree_limit` is deprecated, use `iteration_range` instead.
## [15:56:10] WARNING: src/c_api/c_api.cc:935: `ntree_limit` is deprecated, use `iteration_range` instead.
## [15:56:10] WARNING: src/c_api/c_api.cc:935: `ntree_limit` is deprecated, use `iteration_range` instead.
## [15:56:11] WARNING: src/c_api/c_api.cc:935: `ntree_limit` is deprecated, use `iteration_range` instead.
## [15:56:11] WARNING: src/c_api/c_api.cc:935: `ntree_limit` is deprecated, use `iteration_range` instead.
## [15:56:11] WARNING: src/c_api/c_api.cc:935: `ntree_limit` is deprecated, use `iteration_range` instead.
## [15:56:11] WARNING: src/c_api/c_api.cc:935: `ntree_limit` is deprecated, use `iteration_range` instead.
## [15:56:11] WARNING: src/c_api/c_api.cc:935: `ntree_limit` is deprecated, use `iteration_range` instead.
## [15:56:11] WARNING: src/c_api/c_api.cc:935: `ntree_limit` is deprecated, use `iteration_range` instead.
## [15:56:11] WARNING: src/c_api/c_api.cc:935: `ntree_limit` is deprecated, use `iteration_range` instead.
## [15:56:11] WARNING: src/c_api/c_api.cc:935: `ntree_limit` is deprecated, use `iteration_range` instead.
## [15:56:11] WARNING: src/c_api/c_api.cc:935: `ntree_limit` is deprecated, use `iteration_range` instead.
## [15:56:11] WARNING: src/c_api/c_api.cc:935: `ntree_limit` is deprecated, use `iteration_range` instead.
## [15:56:11] WARNING: src/c_api/c_api.cc:935: `ntree_limit` is deprecated, use `iteration_range` instead.
## [15:56:11] WARNING: src/c_api/c_api.cc:935: `ntree_limit` is deprecated, use `iteration_range` instead.
## [15:56:12] WARNING: src/c_api/c_api.cc:935: `ntree_limit` is deprecated, use `iteration_range` instead.
## [15:56:12] WARNING: src/c_api/c_api.cc:935: `ntree_limit` is deprecated, use `iteration_range` instead.
## [15:56:12] WARNING: src/c_api/c_api.cc:935: `ntree_limit` is deprecated, use `iteration_range` instead.
## [15:56:12] WARNING: src/c_api/c_api.cc:935: `ntree_limit` is deprecated, use `iteration_range` instead.
## [15:56:12] WARNING: src/c_api/c_api.cc:935: `ntree_limit` is deprecated, use `iteration_range` instead.
## [15:56:12] WARNING: src/c_api/c_api.cc:935: `ntree_limit` is deprecated, use `iteration_range` instead.
## [15:56:12] WARNING: src/c_api/c_api.cc:935: `ntree_limit` is deprecated, use `iteration_range` instead.
## [15:56:12] WARNING: src/c_api/c_api.cc:935: `ntree_limit` is deprecated, use `iteration_range` instead.
## [15:56:13] WARNING: src/c_api/c_api.cc:935: `ntree_limit` is deprecated, use `iteration_range` instead.
## [15:56:13] WARNING: src/c_api/c_api.cc:935: `ntree_limit` is deprecated, use `iteration_range` instead.
## [15:56:13] WARNING: src/c_api/c_api.cc:935: `ntree_limit` is deprecated, use `iteration_range` instead.
## [15:56:13] WARNING: src/c_api/c_api.cc:935: `ntree_limit` is deprecated, use `iteration_range` instead.
## [15:56:13] WARNING: src/c_api/c_api.cc:935: `ntree_limit` is deprecated, use `iteration_range` instead.
## [15:56:13] WARNING: src/c_api/c_api.cc:935: `ntree_limit` is deprecated, use `iteration_range` instead.
## [15:56:13] WARNING: src/c_api/c_api.cc:935: `ntree_limit` is deprecated, use `iteration_range` instead.
## [15:56:13] WARNING: src/c_api/c_api.cc:935: `ntree_limit` is deprecated, use `iteration_range` instead.
## [15:56:13] WARNING: src/c_api/c_api.cc:935: `ntree_limit` is deprecated, use `iteration_range` instead.
## [15:56:13] WARNING: src/c_api/c_api.cc:935: `ntree_limit` is deprecated, use `iteration_range` instead.
## [15:56:13] WARNING: src/c_api/c_api.cc:935: `ntree_limit` is deprecated, use `iteration_range` instead.
## [15:56:13] WARNING: src/c_api/c_api.cc:935: `ntree_limit` is deprecated, use `iteration_range` instead.
## [15:56:13] WARNING: src/c_api/c_api.cc:935: `ntree_limit` is deprecated, use `iteration_range` instead.
## [15:56:13] WARNING: src/c_api/c_api.cc:935: `ntree_limit` is deprecated, use `iteration_range` instead.
## [15:56:14] WARNING: src/c_api/c_api.cc:935: `ntree_limit` is deprecated, use `iteration_range` instead.
## [15:56:14] WARNING: src/c_api/c_api.cc:935: `ntree_limit` is deprecated, use `iteration_range` instead.
## [15:56:14] WARNING: src/c_api/c_api.cc:935: `ntree_limit` is deprecated, use `iteration_range` instead.
## [15:56:14] WARNING: src/c_api/c_api.cc:935: `ntree_limit` is deprecated, use `iteration_range` instead.
## [15:56:14] WARNING: src/c_api/c_api.cc:935: `ntree_limit` is deprecated, use `iteration_range` instead.
## [15:56:14] WARNING: src/c_api/c_api.cc:935: `ntree_limit` is deprecated, use `iteration_range` instead.
## [15:56:14] WARNING: src/c_api/c_api.cc:935: `ntree_limit` is deprecated, use `iteration_range` instead.
## [15:56:14] WARNING: src/c_api/c_api.cc:935: `ntree_limit` is deprecated, use `iteration_range` instead.
## [15:56:14] WARNING: src/c_api/c_api.cc:935: `ntree_limit` is deprecated, use `iteration_range` instead.
## [15:56:14] WARNING: src/c_api/c_api.cc:935: `ntree_limit` is deprecated, use `iteration_range` instead.
## [15:56:14] WARNING: src/c_api/c_api.cc:935: `ntree_limit` is deprecated, use `iteration_range` instead.
## [15:56:14] WARNING: src/c_api/c_api.cc:935: `ntree_limit` is deprecated, use `iteration_range` instead.
## [15:56:15] WARNING: src/c_api/c_api.cc:935: `ntree_limit` is deprecated, use `iteration_range` instead.
## [15:56:15] WARNING: src/c_api/c_api.cc:935: `ntree_limit` is deprecated, use `iteration_range` instead.
## [15:56:15] WARNING: src/c_api/c_api.cc:935: `ntree_limit` is deprecated, use `iteration_range` instead.
## [15:56:15] WARNING: src/c_api/c_api.cc:935: `ntree_limit` is deprecated, use `iteration_range` instead.
## [15:56:15] WARNING: src/c_api/c_api.cc:935: `ntree_limit` is deprecated, use `iteration_range` instead.
## [15:56:15] WARNING: src/c_api/c_api.cc:935: `ntree_limit` is deprecated, use `iteration_range` instead.
## [15:56:15] WARNING: src/c_api/c_api.cc:935: `ntree_limit` is deprecated, use `iteration_range` instead.
## [15:56:15] WARNING: src/c_api/c_api.cc:935: `ntree_limit` is deprecated, use `iteration_range` instead.
## [15:56:16] WARNING: src/c_api/c_api.cc:935: `ntree_limit` is deprecated, use `iteration_range` instead.
## [15:56:16] WARNING: src/c_api/c_api.cc:935: `ntree_limit` is deprecated, use `iteration_range` instead.
## [15:56:16] WARNING: src/c_api/c_api.cc:935: `ntree_limit` is deprecated, use `iteration_range` instead.
## [15:56:16] WARNING: src/c_api/c_api.cc:935: `ntree_limit` is deprecated, use `iteration_range` instead.
## [15:56:16] WARNING: src/c_api/c_api.cc:935: `ntree_limit` is deprecated, use `iteration_range` instead.
## [15:56:16] WARNING: src/c_api/c_api.cc:935: `ntree_limit` is deprecated, use `iteration_range` instead.
## [15:56:16] WARNING: src/c_api/c_api.cc:935: `ntree_limit` is deprecated, use `iteration_range` instead.
## [15:56:16] WARNING: src/c_api/c_api.cc:935: `ntree_limit` is deprecated, use `iteration_range` instead.
## [15:56:16] WARNING: src/c_api/c_api.cc:935: `ntree_limit` is deprecated, use `iteration_range` instead.
## [15:56:16] WARNING: src/c_api/c_api.cc:935: `ntree_limit` is deprecated, use `iteration_range` instead.
## [15:56:17] WARNING: src/c_api/c_api.cc:935: `ntree_limit` is deprecated, use `iteration_range` instead.
## [15:56:17] WARNING: src/c_api/c_api.cc:935: `ntree_limit` is deprecated, use `iteration_range` instead.
## [15:56:17] WARNING: src/c_api/c_api.cc:935: `ntree_limit` is deprecated, use `iteration_range` instead.
## [15:56:17] WARNING: src/c_api/c_api.cc:935: `ntree_limit` is deprecated, use `iteration_range` instead.
## [15:56:17] WARNING: src/c_api/c_api.cc:935: `ntree_limit` is deprecated, use `iteration_range` instead.
## [15:56:17] WARNING: src/c_api/c_api.cc:935: `ntree_limit` is deprecated, use `iteration_range` instead.
## [15:56:17] WARNING: src/c_api/c_api.cc:935: `ntree_limit` is deprecated, use `iteration_range` instead.
## [15:56:17] WARNING: src/c_api/c_api.cc:935: `ntree_limit` is deprecated, use `iteration_range` instead.
## [15:56:17] WARNING: src/c_api/c_api.cc:935: `ntree_limit` is deprecated, use `iteration_range` instead.
## [15:56:17] WARNING: src/c_api/c_api.cc:935: `ntree_limit` is deprecated, use `iteration_range` instead.
## [15:56:17] WARNING: src/c_api/c_api.cc:935: `ntree_limit` is deprecated, use `iteration_range` instead.
## [15:56:17] WARNING: src/c_api/c_api.cc:935: `ntree_limit` is deprecated, use `iteration_range` instead.
## [15:56:18] WARNING: src/c_api/c_api.cc:935: `ntree_limit` is deprecated, use `iteration_range` instead.
## [15:56:18] WARNING: src/c_api/c_api.cc:935: `ntree_limit` is deprecated, use `iteration_range` instead.
## [15:56:18] WARNING: src/c_api/c_api.cc:935: `ntree_limit` is deprecated, use `iteration_range` instead.
## [15:56:18] WARNING: src/c_api/c_api.cc:935: `ntree_limit` is deprecated, use `iteration_range` instead.
## [15:56:18] WARNING: src/c_api/c_api.cc:935: `ntree_limit` is deprecated, use `iteration_range` instead.
## [15:56:18] WARNING: src/c_api/c_api.cc:935: `ntree_limit` is deprecated, use `iteration_range` instead.
## [15:56:18] WARNING: src/c_api/c_api.cc:935: `ntree_limit` is deprecated, use `iteration_range` instead.
## [15:56:18] WARNING: src/c_api/c_api.cc:935: `ntree_limit` is deprecated, use `iteration_range` instead.
## [15:56:18] WARNING: src/c_api/c_api.cc:935: `ntree_limit` is deprecated, use `iteration_range` instead.
## [15:56:18] WARNING: src/c_api/c_api.cc:935: `ntree_limit` is deprecated, use `iteration_range` instead.
## [15:56:18] WARNING: src/c_api/c_api.cc:935: `ntree_limit` is deprecated, use `iteration_range` instead.
## [15:56:18] WARNING: src/c_api/c_api.cc:935: `ntree_limit` is deprecated, use `iteration_range` instead.
## [15:56:19] WARNING: src/c_api/c_api.cc:935: `ntree_limit` is deprecated, use `iteration_range` instead.
## [15:56:19] WARNING: src/c_api/c_api.cc:935: `ntree_limit` is deprecated, use `iteration_range` instead.
## [15:56:19] WARNING: src/c_api/c_api.cc:935: `ntree_limit` is deprecated, use `iteration_range` instead.
## [15:56:19] WARNING: src/c_api/c_api.cc:935: `ntree_limit` is deprecated, use `iteration_range` instead.
## [15:56:19] WARNING: src/c_api/c_api.cc:935: `ntree_limit` is deprecated, use `iteration_range` instead.
## [15:56:19] WARNING: src/c_api/c_api.cc:935: `ntree_limit` is deprecated, use `iteration_range` instead.
## [15:56:19] WARNING: src/c_api/c_api.cc:935: `ntree_limit` is deprecated, use `iteration_range` instead.
## [15:56:19] WARNING: src/c_api/c_api.cc:935: `ntree_limit` is deprecated, use `iteration_range` instead.
## [15:56:22] WARNING: src/c_api/c_api.cc:935: `ntree_limit` is deprecated, use `iteration_range` instead.
## [15:56:22] WARNING: src/c_api/c_api.cc:935: `ntree_limit` is deprecated, use `iteration_range` instead.
## [15:56:22] WARNING: src/c_api/c_api.cc:935: `ntree_limit` is deprecated, use `iteration_range` instead.
## [15:56:22] WARNING: src/c_api/c_api.cc:935: `ntree_limit` is deprecated, use `iteration_range` instead.
## [15:56:22] WARNING: src/c_api/c_api.cc:935: `ntree_limit` is deprecated, use `iteration_range` instead.
## [15:56:22] WARNING: src/c_api/c_api.cc:935: `ntree_limit` is deprecated, use `iteration_range` instead.
## [15:56:23] WARNING: src/c_api/c_api.cc:935: `ntree_limit` is deprecated, use `iteration_range` instead.
## [15:56:23] WARNING: src/c_api/c_api.cc:935: `ntree_limit` is deprecated, use `iteration_range` instead.
## [15:56:23] WARNING: src/c_api/c_api.cc:935: `ntree_limit` is deprecated, use `iteration_range` instead.
## [15:56:23] WARNING: src/c_api/c_api.cc:935: `ntree_limit` is deprecated, use `iteration_range` instead.
## [15:56:23] WARNING: src/c_api/c_api.cc:935: `ntree_limit` is deprecated, use `iteration_range` instead.
## [15:56:23] WARNING: src/c_api/c_api.cc:935: `ntree_limit` is deprecated, use `iteration_range` instead.
## [15:56:23] WARNING: src/c_api/c_api.cc:935: `ntree_limit` is deprecated, use `iteration_range` instead.
## [15:56:23] WARNING: src/c_api/c_api.cc:935: `ntree_limit` is deprecated, use `iteration_range` instead.
## [15:56:23] WARNING: src/c_api/c_api.cc:935: `ntree_limit` is deprecated, use `iteration_range` instead.
## [15:56:23] WARNING: src/c_api/c_api.cc:935: `ntree_limit` is deprecated, use `iteration_range` instead.
## [15:56:23] WARNING: src/c_api/c_api.cc:935: `ntree_limit` is deprecated, use `iteration_range` instead.
## [15:56:23] WARNING: src/c_api/c_api.cc:935: `ntree_limit` is deprecated, use `iteration_range` instead.
## [15:56:24] WARNING: src/c_api/c_api.cc:935: `ntree_limit` is deprecated, use `iteration_range` instead.
## [15:56:24] WARNING: src/c_api/c_api.cc:935: `ntree_limit` is deprecated, use `iteration_range` instead.
## [15:56:24] WARNING: src/c_api/c_api.cc:935: `ntree_limit` is deprecated, use `iteration_range` instead.
## [15:56:24] WARNING: src/c_api/c_api.cc:935: `ntree_limit` is deprecated, use `iteration_range` instead.
## [15:56:24] WARNING: src/c_api/c_api.cc:935: `ntree_limit` is deprecated, use `iteration_range` instead.
## [15:56:24] WARNING: src/c_api/c_api.cc:935: `ntree_limit` is deprecated, use `iteration_range` instead.
## [15:56:24] WARNING: src/c_api/c_api.cc:935: `ntree_limit` is deprecated, use `iteration_range` instead.
## [15:56:24] WARNING: src/c_api/c_api.cc:935: `ntree_limit` is deprecated, use `iteration_range` instead.
## [15:56:24] WARNING: src/c_api/c_api.cc:935: `ntree_limit` is deprecated, use `iteration_range` instead.
## [15:56:24] WARNING: src/c_api/c_api.cc:935: `ntree_limit` is deprecated, use `iteration_range` instead.
## [15:56:24] WARNING: src/c_api/c_api.cc:935: `ntree_limit` is deprecated, use `iteration_range` instead.
## [15:56:24] WARNING: src/c_api/c_api.cc:935: `ntree_limit` is deprecated, use `iteration_range` instead.
## [15:56:25] WARNING: src/c_api/c_api.cc:935: `ntree_limit` is deprecated, use `iteration_range` instead.
## [15:56:25] WARNING: src/c_api/c_api.cc:935: `ntree_limit` is deprecated, use `iteration_range` instead.
## [15:56:25] WARNING: src/c_api/c_api.cc:935: `ntree_limit` is deprecated, use `iteration_range` instead.
## [15:56:25] WARNING: src/c_api/c_api.cc:935: `ntree_limit` is deprecated, use `iteration_range` instead.
## [15:56:25] WARNING: src/c_api/c_api.cc:935: `ntree_limit` is deprecated, use `iteration_range` instead.
## [15:56:25] WARNING: src/c_api/c_api.cc:935: `ntree_limit` is deprecated, use `iteration_range` instead.
## [15:56:25] WARNING: src/c_api/c_api.cc:935: `ntree_limit` is deprecated, use `iteration_range` instead.
## [15:56:25] WARNING: src/c_api/c_api.cc:935: `ntree_limit` is deprecated, use `iteration_range` instead.
## [15:56:25] WARNING: src/c_api/c_api.cc:935: `ntree_limit` is deprecated, use `iteration_range` instead.
## [15:56:25] WARNING: src/c_api/c_api.cc:935: `ntree_limit` is deprecated, use `iteration_range` instead.
## [15:56:26] WARNING: src/c_api/c_api.cc:935: `ntree_limit` is deprecated, use `iteration_range` instead.
## [15:56:26] WARNING: src/c_api/c_api.cc:935: `ntree_limit` is deprecated, use `iteration_range` instead.
## [15:56:26] WARNING: src/c_api/c_api.cc:935: `ntree_limit` is deprecated, use `iteration_range` instead.
## [15:56:26] WARNING: src/c_api/c_api.cc:935: `ntree_limit` is deprecated, use `iteration_range` instead.
## [15:56:26] WARNING: src/c_api/c_api.cc:935: `ntree_limit` is deprecated, use `iteration_range` instead.
## [15:56:26] WARNING: src/c_api/c_api.cc:935: `ntree_limit` is deprecated, use `iteration_range` instead.
## [15:56:26] WARNING: src/c_api/c_api.cc:935: `ntree_limit` is deprecated, use `iteration_range` instead.
## [15:56:26] WARNING: src/c_api/c_api.cc:935: `ntree_limit` is deprecated, use `iteration_range` instead.
## [15:56:26] WARNING: src/c_api/c_api.cc:935: `ntree_limit` is deprecated, use `iteration_range` instead.
## [15:56:26] WARNING: src/c_api/c_api.cc:935: `ntree_limit` is deprecated, use `iteration_range` instead.
## [15:56:26] WARNING: src/c_api/c_api.cc:935: `ntree_limit` is deprecated, use `iteration_range` instead.
## [15:56:26] WARNING: src/c_api/c_api.cc:935: `ntree_limit` is deprecated, use `iteration_range` instead.
## [15:56:26] WARNING: src/c_api/c_api.cc:935: `ntree_limit` is deprecated, use `iteration_range` instead.
## [15:56:26] WARNING: src/c_api/c_api.cc:935: `ntree_limit` is deprecated, use `iteration_range` instead.
## [15:56:27] WARNING: src/c_api/c_api.cc:935: `ntree_limit` is deprecated, use `iteration_range` instead.
## [15:56:27] WARNING: src/c_api/c_api.cc:935: `ntree_limit` is deprecated, use `iteration_range` instead.
## [15:56:27] WARNING: src/c_api/c_api.cc:935: `ntree_limit` is deprecated, use `iteration_range` instead.
## [15:56:27] WARNING: src/c_api/c_api.cc:935: `ntree_limit` is deprecated, use `iteration_range` instead.
## [15:56:27] WARNING: src/c_api/c_api.cc:935: `ntree_limit` is deprecated, use `iteration_range` instead.
## [15:56:27] WARNING: src/c_api/c_api.cc:935: `ntree_limit` is deprecated, use `iteration_range` instead.
## [15:56:27] WARNING: src/c_api/c_api.cc:935: `ntree_limit` is deprecated, use `iteration_range` instead.
## [15:56:27] WARNING: src/c_api/c_api.cc:935: `ntree_limit` is deprecated, use `iteration_range` instead.
## [15:56:27] WARNING: src/c_api/c_api.cc:935: `ntree_limit` is deprecated, use `iteration_range` instead.
## [15:56:27] WARNING: src/c_api/c_api.cc:935: `ntree_limit` is deprecated, use `iteration_range` instead.
## [15:56:28] WARNING: src/c_api/c_api.cc:935: `ntree_limit` is deprecated, use `iteration_range` instead.
## [15:56:28] WARNING: src/c_api/c_api.cc:935: `ntree_limit` is deprecated, use `iteration_range` instead.
## [15:56:28] WARNING: src/c_api/c_api.cc:935: `ntree_limit` is deprecated, use `iteration_range` instead.
## [15:56:28] WARNING: src/c_api/c_api.cc:935: `ntree_limit` is deprecated, use `iteration_range` instead.
## [15:56:28] WARNING: src/c_api/c_api.cc:935: `ntree_limit` is deprecated, use `iteration_range` instead.
## [15:56:28] WARNING: src/c_api/c_api.cc:935: `ntree_limit` is deprecated, use `iteration_range` instead.
## [15:56:28] WARNING: src/c_api/c_api.cc:935: `ntree_limit` is deprecated, use `iteration_range` instead.
## [15:56:28] WARNING: src/c_api/c_api.cc:935: `ntree_limit` is deprecated, use `iteration_range` instead.
## [15:56:28] WARNING: src/c_api/c_api.cc:935: `ntree_limit` is deprecated, use `iteration_range` instead.
## [15:56:28] WARNING: src/c_api/c_api.cc:935: `ntree_limit` is deprecated, use `iteration_range` instead.
## [15:56:28] WARNING: src/c_api/c_api.cc:935: `ntree_limit` is deprecated, use `iteration_range` instead.
## [15:56:28] WARNING: src/c_api/c_api.cc:935: `ntree_limit` is deprecated, use `iteration_range` instead.
## [15:56:29] WARNING: src/c_api/c_api.cc:935: `ntree_limit` is deprecated, use `iteration_range` instead.
## [15:56:29] WARNING: src/c_api/c_api.cc:935: `ntree_limit` is deprecated, use `iteration_range` instead.
## [15:56:29] WARNING: src/c_api/c_api.cc:935: `ntree_limit` is deprecated, use `iteration_range` instead.
## [15:56:29] WARNING: src/c_api/c_api.cc:935: `ntree_limit` is deprecated, use `iteration_range` instead.
## [15:56:29] WARNING: src/c_api/c_api.cc:935: `ntree_limit` is deprecated, use `iteration_range` instead.
## [15:56:29] WARNING: src/c_api/c_api.cc:935: `ntree_limit` is deprecated, use `iteration_range` instead.
## [15:56:29] WARNING: src/c_api/c_api.cc:935: `ntree_limit` is deprecated, use `iteration_range` instead.
## [15:56:29] WARNING: src/c_api/c_api.cc:935: `ntree_limit` is deprecated, use `iteration_range` instead.
## [15:56:29] WARNING: src/c_api/c_api.cc:935: `ntree_limit` is deprecated, use `iteration_range` instead.
## [15:56:29] WARNING: src/c_api/c_api.cc:935: `ntree_limit` is deprecated, use `iteration_range` instead.
## [15:56:29] WARNING: src/c_api/c_api.cc:935: `ntree_limit` is deprecated, use `iteration_range` instead.
## [15:56:29] WARNING: src/c_api/c_api.cc:935: `ntree_limit` is deprecated, use `iteration_range` instead.
## [15:56:30] WARNING: src/c_api/c_api.cc:935: `ntree_limit` is deprecated, use `iteration_range` instead.
## [15:56:30] WARNING: src/c_api/c_api.cc:935: `ntree_limit` is deprecated, use `iteration_range` instead.
## [15:56:30] WARNING: src/c_api/c_api.cc:935: `ntree_limit` is deprecated, use `iteration_range` instead.
## [15:56:30] WARNING: src/c_api/c_api.cc:935: `ntree_limit` is deprecated, use `iteration_range` instead.
## [15:56:30] WARNING: src/c_api/c_api.cc:935: `ntree_limit` is deprecated, use `iteration_range` instead.
## [15:56:30] WARNING: src/c_api/c_api.cc:935: `ntree_limit` is deprecated, use `iteration_range` instead.
## [15:56:30] WARNING: src/c_api/c_api.cc:935: `ntree_limit` is deprecated, use `iteration_range` instead.
## [15:56:30] WARNING: src/c_api/c_api.cc:935: `ntree_limit` is deprecated, use `iteration_range` instead.
## [15:56:30] WARNING: src/c_api/c_api.cc:935: `ntree_limit` is deprecated, use `iteration_range` instead.
## [15:56:30] WARNING: src/c_api/c_api.cc:935: `ntree_limit` is deprecated, use `iteration_range` instead.
## [15:56:30] WARNING: src/c_api/c_api.cc:935: `ntree_limit` is deprecated, use `iteration_range` instead.
## [15:56:30] WARNING: src/c_api/c_api.cc:935: `ntree_limit` is deprecated, use `iteration_range` instead.
## [15:56:31] WARNING: src/c_api/c_api.cc:935: `ntree_limit` is deprecated, use `iteration_range` instead.
## [15:56:31] WARNING: src/c_api/c_api.cc:935: `ntree_limit` is deprecated, use `iteration_range` instead.
## [15:56:31] WARNING: src/c_api/c_api.cc:935: `ntree_limit` is deprecated, use `iteration_range` instead.
## [15:56:31] WARNING: src/c_api/c_api.cc:935: `ntree_limit` is deprecated, use `iteration_range` instead.
## [15:56:31] WARNING: src/c_api/c_api.cc:935: `ntree_limit` is deprecated, use `iteration_range` instead.
## [15:56:31] WARNING: src/c_api/c_api.cc:935: `ntree_limit` is deprecated, use `iteration_range` instead.
## [15:56:31] WARNING: src/c_api/c_api.cc:935: `ntree_limit` is deprecated, use `iteration_range` instead.
## [15:56:31] WARNING: src/c_api/c_api.cc:935: `ntree_limit` is deprecated, use `iteration_range` instead.
## [15:56:32] WARNING: src/c_api/c_api.cc:935: `ntree_limit` is deprecated, use `iteration_range` instead.
## [15:56:32] WARNING: src/c_api/c_api.cc:935: `ntree_limit` is deprecated, use `iteration_range` instead.
## [15:56:32] WARNING: src/c_api/c_api.cc:935: `ntree_limit` is deprecated, use `iteration_range` instead.
## [15:56:32] WARNING: src/c_api/c_api.cc:935: `ntree_limit` is deprecated, use `iteration_range` instead.
## [15:56:32] WARNING: src/c_api/c_api.cc:935: `ntree_limit` is deprecated, use `iteration_range` instead.
## [15:56:32] WARNING: src/c_api/c_api.cc:935: `ntree_limit` is deprecated, use `iteration_range` instead.
## [15:56:32] WARNING: src/c_api/c_api.cc:935: `ntree_limit` is deprecated, use `iteration_range` instead.
## [15:56:32] WARNING: src/c_api/c_api.cc:935: `ntree_limit` is deprecated, use `iteration_range` instead.
## [15:56:32] WARNING: src/c_api/c_api.cc:935: `ntree_limit` is deprecated, use `iteration_range` instead.
## [15:56:32] WARNING: src/c_api/c_api.cc:935: `ntree_limit` is deprecated, use `iteration_range` instead.
## [15:56:32] WARNING: src/c_api/c_api.cc:935: `ntree_limit` is deprecated, use `iteration_range` instead.
## [15:56:32] WARNING: src/c_api/c_api.cc:935: `ntree_limit` is deprecated, use `iteration_range` instead.
## [15:56:32] WARNING: src/c_api/c_api.cc:935: `ntree_limit` is deprecated, use `iteration_range` instead.
## [15:56:32] WARNING: src/c_api/c_api.cc:935: `ntree_limit` is deprecated, use `iteration_range` instead.
## [15:56:32] WARNING: src/c_api/c_api.cc:935: `ntree_limit` is deprecated, use `iteration_range` instead.
## [15:56:32] WARNING: src/c_api/c_api.cc:935: `ntree_limit` is deprecated, use `iteration_range` instead.
## [15:56:33] WARNING: src/c_api/c_api.cc:935: `ntree_limit` is deprecated, use `iteration_range` instead.
## [15:56:33] WARNING: src/c_api/c_api.cc:935: `ntree_limit` is deprecated, use `iteration_range` instead.
## [15:56:33] WARNING: src/c_api/c_api.cc:935: `ntree_limit` is deprecated, use `iteration_range` instead.
## [15:56:33] WARNING: src/c_api/c_api.cc:935: `ntree_limit` is deprecated, use `iteration_range` instead.
## [15:56:33] WARNING: src/c_api/c_api.cc:935: `ntree_limit` is deprecated, use `iteration_range` instead.
## [15:56:33] WARNING: src/c_api/c_api.cc:935: `ntree_limit` is deprecated, use `iteration_range` instead.
## [15:56:33] WARNING: src/c_api/c_api.cc:935: `ntree_limit` is deprecated, use `iteration_range` instead.
## [15:56:33] WARNING: src/c_api/c_api.cc:935: `ntree_limit` is deprecated, use `iteration_range` instead.
## [15:56:33] WARNING: src/c_api/c_api.cc:935: `ntree_limit` is deprecated, use `iteration_range` instead.
## [15:56:33] WARNING: src/c_api/c_api.cc:935: `ntree_limit` is deprecated, use `iteration_range` instead.
## [15:56:34] WARNING: src/c_api/c_api.cc:935: `ntree_limit` is deprecated, use `iteration_range` instead.
## [15:56:34] WARNING: src/c_api/c_api.cc:935: `ntree_limit` is deprecated, use `iteration_range` instead.
## [15:56:34] WARNING: src/c_api/c_api.cc:935: `ntree_limit` is deprecated, use `iteration_range` instead.
## [15:56:34] WARNING: src/c_api/c_api.cc:935: `ntree_limit` is deprecated, use `iteration_range` instead.
## [15:56:34] WARNING: src/c_api/c_api.cc:935: `ntree_limit` is deprecated, use `iteration_range` instead.
## [15:56:34] WARNING: src/c_api/c_api.cc:935: `ntree_limit` is deprecated, use `iteration_range` instead.
## [15:56:34] WARNING: src/c_api/c_api.cc:935: `ntree_limit` is deprecated, use `iteration_range` instead.
## [15:56:34] WARNING: src/c_api/c_api.cc:935: `ntree_limit` is deprecated, use `iteration_range` instead.
## [15:56:34] WARNING: src/c_api/c_api.cc:935: `ntree_limit` is deprecated, use `iteration_range` instead.
## [15:56:34] WARNING: src/c_api/c_api.cc:935: `ntree_limit` is deprecated, use `iteration_range` instead.
## [15:56:34] WARNING: src/c_api/c_api.cc:935: `ntree_limit` is deprecated, use `iteration_range` instead.
## [15:56:34] WARNING: src/c_api/c_api.cc:935: `ntree_limit` is deprecated, use `iteration_range` instead.
## [15:56:35] WARNING: src/c_api/c_api.cc:935: `ntree_limit` is deprecated, use `iteration_range` instead.
## [15:56:35] WARNING: src/c_api/c_api.cc:935: `ntree_limit` is deprecated, use `iteration_range` instead.
## [15:56:35] WARNING: src/c_api/c_api.cc:935: `ntree_limit` is deprecated, use `iteration_range` instead.
## [15:56:35] WARNING: src/c_api/c_api.cc:935: `ntree_limit` is deprecated, use `iteration_range` instead.
## [15:56:35] WARNING: src/c_api/c_api.cc:935: `ntree_limit` is deprecated, use `iteration_range` instead.
## [15:56:35] WARNING: src/c_api/c_api.cc:935: `ntree_limit` is deprecated, use `iteration_range` instead.
## [15:56:35] WARNING: src/c_api/c_api.cc:935: `ntree_limit` is deprecated, use `iteration_range` instead.
## [15:56:35] WARNING: src/c_api/c_api.cc:935: `ntree_limit` is deprecated, use `iteration_range` instead.
## [15:56:35] WARNING: src/c_api/c_api.cc:935: `ntree_limit` is deprecated, use `iteration_range` instead.
## [15:56:35] WARNING: src/c_api/c_api.cc:935: `ntree_limit` is deprecated, use `iteration_range` instead.
## [15:56:35] WARNING: src/c_api/c_api.cc:935: `ntree_limit` is deprecated, use `iteration_range` instead.
## [15:56:35] WARNING: src/c_api/c_api.cc:935: `ntree_limit` is deprecated, use `iteration_range` instead.
## [15:56:36] WARNING: src/c_api/c_api.cc:935: `ntree_limit` is deprecated, use `iteration_range` instead.
## [15:56:36] WARNING: src/c_api/c_api.cc:935: `ntree_limit` is deprecated, use `iteration_range` instead.
## [15:56:36] WARNING: src/c_api/c_api.cc:935: `ntree_limit` is deprecated, use `iteration_range` instead.
## [15:56:36] WARNING: src/c_api/c_api.cc:935: `ntree_limit` is deprecated, use `iteration_range` instead.
## [15:56:36] WARNING: src/c_api/c_api.cc:935: `ntree_limit` is deprecated, use `iteration_range` instead.
## [15:56:36] WARNING: src/c_api/c_api.cc:935: `ntree_limit` is deprecated, use `iteration_range` instead.
## [15:56:36] WARNING: src/c_api/c_api.cc:935: `ntree_limit` is deprecated, use `iteration_range` instead.
## [15:56:36] WARNING: src/c_api/c_api.cc:935: `ntree_limit` is deprecated, use `iteration_range` instead.
## [15:56:36] WARNING: src/c_api/c_api.cc:935: `ntree_limit` is deprecated, use `iteration_range` instead.
## [15:56:36] WARNING: src/c_api/c_api.cc:935: `ntree_limit` is deprecated, use `iteration_range` instead.
## [15:56:36] WARNING: src/c_api/c_api.cc:935: `ntree_limit` is deprecated, use `iteration_range` instead.
## [15:56:36] WARNING: src/c_api/c_api.cc:935: `ntree_limit` is deprecated, use `iteration_range` instead.
## [15:56:37] WARNING: src/c_api/c_api.cc:935: `ntree_limit` is deprecated, use `iteration_range` instead.
## [15:56:37] WARNING: src/c_api/c_api.cc:935: `ntree_limit` is deprecated, use `iteration_range` instead.
## [15:56:37] WARNING: src/c_api/c_api.cc:935: `ntree_limit` is deprecated, use `iteration_range` instead.
## [15:56:37] WARNING: src/c_api/c_api.cc:935: `ntree_limit` is deprecated, use `iteration_range` instead.
## [15:56:37] WARNING: src/c_api/c_api.cc:935: `ntree_limit` is deprecated, use `iteration_range` instead.
## [15:56:37] WARNING: src/c_api/c_api.cc:935: `ntree_limit` is deprecated, use `iteration_range` instead.
## [15:56:37] WARNING: src/c_api/c_api.cc:935: `ntree_limit` is deprecated, use `iteration_range` instead.
## [15:56:37] WARNING: src/c_api/c_api.cc:935: `ntree_limit` is deprecated, use `iteration_range` instead.
## [15:56:38] WARNING: src/c_api/c_api.cc:935: `ntree_limit` is deprecated, use `iteration_range` instead.
## [15:56:38] WARNING: src/c_api/c_api.cc:935: `ntree_limit` is deprecated, use `iteration_range` instead.
## [15:56:38] WARNING: src/c_api/c_api.cc:935: `ntree_limit` is deprecated, use `iteration_range` instead.
## [15:56:38] WARNING: src/c_api/c_api.cc:935: `ntree_limit` is deprecated, use `iteration_range` instead.
## [15:56:38] WARNING: src/c_api/c_api.cc:935: `ntree_limit` is deprecated, use `iteration_range` instead.
## [15:56:38] WARNING: src/c_api/c_api.cc:935: `ntree_limit` is deprecated, use `iteration_range` instead.
## [15:56:38] WARNING: src/c_api/c_api.cc:935: `ntree_limit` is deprecated, use `iteration_range` instead.
## [15:56:38] WARNING: src/c_api/c_api.cc:935: `ntree_limit` is deprecated, use `iteration_range` instead.
## [15:56:38] WARNING: src/c_api/c_api.cc:935: `ntree_limit` is deprecated, use `iteration_range` instead.
## [15:56:38] WARNING: src/c_api/c_api.cc:935: `ntree_limit` is deprecated, use `iteration_range` instead.
## [15:56:38] WARNING: src/c_api/c_api.cc:935: `ntree_limit` is deprecated, use `iteration_range` instead.
## [15:56:38] WARNING: src/c_api/c_api.cc:935: `ntree_limit` is deprecated, use `iteration_range` instead.
## [15:56:38] WARNING: src/c_api/c_api.cc:935: `ntree_limit` is deprecated, use `iteration_range` instead.
## [15:56:38] WARNING: src/c_api/c_api.cc:935: `ntree_limit` is deprecated, use `iteration_range` instead.
## [15:56:39] WARNING: src/c_api/c_api.cc:935: `ntree_limit` is deprecated, use `iteration_range` instead.
## [15:56:39] WARNING: src/c_api/c_api.cc:935: `ntree_limit` is deprecated, use `iteration_range` instead.
## [15:56:39] WARNING: src/c_api/c_api.cc:935: `ntree_limit` is deprecated, use `iteration_range` instead.
## [15:56:39] WARNING: src/c_api/c_api.cc:935: `ntree_limit` is deprecated, use `iteration_range` instead.
## [15:56:39] WARNING: src/c_api/c_api.cc:935: `ntree_limit` is deprecated, use `iteration_range` instead.
## [15:56:39] WARNING: src/c_api/c_api.cc:935: `ntree_limit` is deprecated, use `iteration_range` instead.
## [15:56:39] WARNING: src/c_api/c_api.cc:935: `ntree_limit` is deprecated, use `iteration_range` instead.
## [15:56:39] WARNING: src/c_api/c_api.cc:935: `ntree_limit` is deprecated, use `iteration_range` instead.
## [15:56:39] WARNING: src/c_api/c_api.cc:935: `ntree_limit` is deprecated, use `iteration_range` instead.
## [15:56:39] WARNING: src/c_api/c_api.cc:935: `ntree_limit` is deprecated, use `iteration_range` instead.
## [15:56:39] WARNING: src/c_api/c_api.cc:935: `ntree_limit` is deprecated, use `iteration_range` instead.
## [15:56:39] WARNING: src/c_api/c_api.cc:935: `ntree_limit` is deprecated, use `iteration_range` instead.
## [15:56:40] WARNING: src/c_api/c_api.cc:935: `ntree_limit` is deprecated, use `iteration_range` instead.
## [15:56:40] WARNING: src/c_api/c_api.cc:935: `ntree_limit` is deprecated, use `iteration_range` instead.
## [15:56:40] WARNING: src/c_api/c_api.cc:935: `ntree_limit` is deprecated, use `iteration_range` instead.
## [15:56:40] WARNING: src/c_api/c_api.cc:935: `ntree_limit` is deprecated, use `iteration_range` instead.
## [15:56:40] WARNING: src/c_api/c_api.cc:935: `ntree_limit` is deprecated, use `iteration_range` instead.
## [15:56:40] WARNING: src/c_api/c_api.cc:935: `ntree_limit` is deprecated, use `iteration_range` instead.
## [15:56:40] WARNING: src/c_api/c_api.cc:935: `ntree_limit` is deprecated, use `iteration_range` instead.
## [15:56:40] WARNING: src/c_api/c_api.cc:935: `ntree_limit` is deprecated, use `iteration_range` instead.
## [15:56:40] WARNING: src/c_api/c_api.cc:935: `ntree_limit` is deprecated, use `iteration_range` instead.
## [15:56:40] WARNING: src/c_api/c_api.cc:935: `ntree_limit` is deprecated, use `iteration_range` instead.
## [15:56:41] WARNING: src/c_api/c_api.cc:935: `ntree_limit` is deprecated, use `iteration_range` instead.
## [15:56:41] WARNING: src/c_api/c_api.cc:935: `ntree_limit` is deprecated, use `iteration_range` instead.
## [15:56:41] WARNING: src/c_api/c_api.cc:935: `ntree_limit` is deprecated, use `iteration_range` instead.
## [15:56:41] WARNING: src/c_api/c_api.cc:935: `ntree_limit` is deprecated, use `iteration_range` instead.
## [15:56:41] WARNING: src/c_api/c_api.cc:935: `ntree_limit` is deprecated, use `iteration_range` instead.
## [15:56:41] WARNING: src/c_api/c_api.cc:935: `ntree_limit` is deprecated, use `iteration_range` instead.
## [15:56:41] WARNING: src/c_api/c_api.cc:935: `ntree_limit` is deprecated, use `iteration_range` instead.
## [15:56:41] WARNING: src/c_api/c_api.cc:935: `ntree_limit` is deprecated, use `iteration_range` instead.
## [15:56:41] WARNING: src/c_api/c_api.cc:935: `ntree_limit` is deprecated, use `iteration_range` instead.
## [15:56:41] WARNING: src/c_api/c_api.cc:935: `ntree_limit` is deprecated, use `iteration_range` instead.
## [15:56:41] WARNING: src/c_api/c_api.cc:935: `ntree_limit` is deprecated, use `iteration_range` instead.
## [15:56:41] WARNING: src/c_api/c_api.cc:935: `ntree_limit` is deprecated, use `iteration_range` instead.
## [15:56:41] WARNING: src/c_api/c_api.cc:935: `ntree_limit` is deprecated, use `iteration_range` instead.
## [15:56:41] WARNING: src/c_api/c_api.cc:935: `ntree_limit` is deprecated, use `iteration_range` instead.
## [15:56:42] WARNING: src/c_api/c_api.cc:935: `ntree_limit` is deprecated, use `iteration_range` instead.
## [15:56:42] WARNING: src/c_api/c_api.cc:935: `ntree_limit` is deprecated, use `iteration_range` instead.
## [15:56:42] WARNING: src/c_api/c_api.cc:935: `ntree_limit` is deprecated, use `iteration_range` instead.
## [15:56:42] WARNING: src/c_api/c_api.cc:935: `ntree_limit` is deprecated, use `iteration_range` instead.
## [15:56:42] WARNING: src/c_api/c_api.cc:935: `ntree_limit` is deprecated, use `iteration_range` instead.
## [15:56:42] WARNING: src/c_api/c_api.cc:935: `ntree_limit` is deprecated, use `iteration_range` instead.
## [15:56:42] WARNING: src/c_api/c_api.cc:935: `ntree_limit` is deprecated, use `iteration_range` instead.
## [15:56:42] WARNING: src/c_api/c_api.cc:935: `ntree_limit` is deprecated, use `iteration_range` instead.
## [15:56:42] WARNING: src/c_api/c_api.cc:935: `ntree_limit` is deprecated, use `iteration_range` instead.
## [15:56:42] WARNING: src/c_api/c_api.cc:935: `ntree_limit` is deprecated, use `iteration_range` instead.
## [15:56:42] WARNING: src/c_api/c_api.cc:935: `ntree_limit` is deprecated, use `iteration_range` instead.
## [15:56:42] WARNING: src/c_api/c_api.cc:935: `ntree_limit` is deprecated, use `iteration_range` instead.
## [15:56:43] WARNING: src/c_api/c_api.cc:935: `ntree_limit` is deprecated, use `iteration_range` instead.
## [15:56:43] WARNING: src/c_api/c_api.cc:935: `ntree_limit` is deprecated, use `iteration_range` instead.
## [15:56:43] WARNING: src/c_api/c_api.cc:935: `ntree_limit` is deprecated, use `iteration_range` instead.
## [15:56:43] WARNING: src/c_api/c_api.cc:935: `ntree_limit` is deprecated, use `iteration_range` instead.
## [15:56:43] WARNING: src/c_api/c_api.cc:935: `ntree_limit` is deprecated, use `iteration_range` instead.
## [15:56:43] WARNING: src/c_api/c_api.cc:935: `ntree_limit` is deprecated, use `iteration_range` instead.
## [15:56:43] WARNING: src/c_api/c_api.cc:935: `ntree_limit` is deprecated, use `iteration_range` instead.
## [15:56:43] WARNING: src/c_api/c_api.cc:935: `ntree_limit` is deprecated, use `iteration_range` instead.
## [15:56:44] WARNING: src/c_api/c_api.cc:935: `ntree_limit` is deprecated, use `iteration_range` instead.
## [15:56:44] WARNING: src/c_api/c_api.cc:935: `ntree_limit` is deprecated, use `iteration_range` instead.
## [15:56:44] WARNING: src/c_api/c_api.cc:935: `ntree_limit` is deprecated, use `iteration_range` instead.
## [15:56:44] WARNING: src/c_api/c_api.cc:935: `ntree_limit` is deprecated, use `iteration_range` instead.
## [15:56:44] WARNING: src/c_api/c_api.cc:935: `ntree_limit` is deprecated, use `iteration_range` instead.
## [15:56:44] WARNING: src/c_api/c_api.cc:935: `ntree_limit` is deprecated, use `iteration_range` instead.
## [15:56:44] WARNING: src/c_api/c_api.cc:935: `ntree_limit` is deprecated, use `iteration_range` instead.
## [15:56:44] WARNING: src/c_api/c_api.cc:935: `ntree_limit` is deprecated, use `iteration_range` instead.
## [15:56:44] WARNING: src/c_api/c_api.cc:935: `ntree_limit` is deprecated, use `iteration_range` instead.
## [15:56:44] WARNING: src/c_api/c_api.cc:935: `ntree_limit` is deprecated, use `iteration_range` instead.
## [15:56:44] WARNING: src/c_api/c_api.cc:935: `ntree_limit` is deprecated, use `iteration_range` instead.
## [15:56:44] WARNING: src/c_api/c_api.cc:935: `ntree_limit` is deprecated, use `iteration_range` instead.
## [15:56:44] WARNING: src/c_api/c_api.cc:935: `ntree_limit` is deprecated, use `iteration_range` instead.
## [15:56:44] WARNING: src/c_api/c_api.cc:935: `ntree_limit` is deprecated, use `iteration_range` instead.
## [15:56:45] WARNING: src/c_api/c_api.cc:935: `ntree_limit` is deprecated, use `iteration_range` instead.
## [15:56:45] WARNING: src/c_api/c_api.cc:935: `ntree_limit` is deprecated, use `iteration_range` instead.
## [15:56:45] WARNING: src/c_api/c_api.cc:935: `ntree_limit` is deprecated, use `iteration_range` instead.
## [15:56:45] WARNING: src/c_api/c_api.cc:935: `ntree_limit` is deprecated, use `iteration_range` instead.
## [15:56:45] WARNING: src/c_api/c_api.cc:935: `ntree_limit` is deprecated, use `iteration_range` instead.
## [15:56:45] WARNING: src/c_api/c_api.cc:935: `ntree_limit` is deprecated, use `iteration_range` instead.
## [15:56:45] WARNING: src/c_api/c_api.cc:935: `ntree_limit` is deprecated, use `iteration_range` instead.
## [15:56:45] WARNING: src/c_api/c_api.cc:935: `ntree_limit` is deprecated, use `iteration_range` instead.
## [15:56:45] WARNING: src/c_api/c_api.cc:935: `ntree_limit` is deprecated, use `iteration_range` instead.
## [15:56:45] WARNING: src/c_api/c_api.cc:935: `ntree_limit` is deprecated, use `iteration_range` instead.
## [15:56:45] WARNING: src/c_api/c_api.cc:935: `ntree_limit` is deprecated, use `iteration_range` instead.
## [15:56:45] WARNING: src/c_api/c_api.cc:935: `ntree_limit` is deprecated, use `iteration_range` instead.
## [15:56:46] WARNING: src/c_api/c_api.cc:935: `ntree_limit` is deprecated, use `iteration_range` instead.
## [15:56:46] WARNING: src/c_api/c_api.cc:935: `ntree_limit` is deprecated, use `iteration_range` instead.
## [15:56:46] WARNING: src/c_api/c_api.cc:935: `ntree_limit` is deprecated, use `iteration_range` instead.
## [15:56:46] WARNING: src/c_api/c_api.cc:935: `ntree_limit` is deprecated, use `iteration_range` instead.
## [15:56:46] WARNING: src/c_api/c_api.cc:935: `ntree_limit` is deprecated, use `iteration_range` instead.
## [15:56:46] WARNING: src/c_api/c_api.cc:935: `ntree_limit` is deprecated, use `iteration_range` instead.
## [15:56:46] WARNING: src/c_api/c_api.cc:935: `ntree_limit` is deprecated, use `iteration_range` instead.
## [15:56:46] WARNING: src/c_api/c_api.cc:935: `ntree_limit` is deprecated, use `iteration_range` instead.
## [15:56:46] WARNING: src/c_api/c_api.cc:935: `ntree_limit` is deprecated, use `iteration_range` instead.
## [15:56:46] WARNING: src/c_api/c_api.cc:935: `ntree_limit` is deprecated, use `iteration_range` instead.
## [15:56:47] WARNING: src/c_api/c_api.cc:935: `ntree_limit` is deprecated, use `iteration_range` instead.
## [15:56:47] WARNING: src/c_api/c_api.cc:935: `ntree_limit` is deprecated, use `iteration_range` instead.
## [15:56:47] WARNING: src/c_api/c_api.cc:935: `ntree_limit` is deprecated, use `iteration_range` instead.
## [15:56:47] WARNING: src/c_api/c_api.cc:935: `ntree_limit` is deprecated, use `iteration_range` instead.
## [15:56:50] WARNING: src/c_api/c_api.cc:935: `ntree_limit` is deprecated, use `iteration_range` instead.
## [15:56:50] WARNING: src/c_api/c_api.cc:935: `ntree_limit` is deprecated, use `iteration_range` instead.
## [15:56:50] WARNING: src/c_api/c_api.cc:935: `ntree_limit` is deprecated, use `iteration_range` instead.
## [15:56:50] WARNING: src/c_api/c_api.cc:935: `ntree_limit` is deprecated, use `iteration_range` instead.
## [15:56:50] WARNING: src/c_api/c_api.cc:935: `ntree_limit` is deprecated, use `iteration_range` instead.
## [15:56:50] WARNING: src/c_api/c_api.cc:935: `ntree_limit` is deprecated, use `iteration_range` instead.
## [15:56:50] WARNING: src/c_api/c_api.cc:935: `ntree_limit` is deprecated, use `iteration_range` instead.
## [15:56:50] WARNING: src/c_api/c_api.cc:935: `ntree_limit` is deprecated, use `iteration_range` instead.
## [15:56:50] WARNING: src/c_api/c_api.cc:935: `ntree_limit` is deprecated, use `iteration_range` instead.
## [15:56:50] WARNING: src/c_api/c_api.cc:935: `ntree_limit` is deprecated, use `iteration_range` instead.
## [15:56:50] WARNING: src/c_api/c_api.cc:935: `ntree_limit` is deprecated, use `iteration_range` instead.
## [15:56:50] WARNING: src/c_api/c_api.cc:935: `ntree_limit` is deprecated, use `iteration_range` instead.
## [15:56:51] WARNING: src/c_api/c_api.cc:935: `ntree_limit` is deprecated, use `iteration_range` instead.
## [15:56:51] WARNING: src/c_api/c_api.cc:935: `ntree_limit` is deprecated, use `iteration_range` instead.
## [15:56:51] WARNING: src/c_api/c_api.cc:935: `ntree_limit` is deprecated, use `iteration_range` instead.
## [15:56:51] WARNING: src/c_api/c_api.cc:935: `ntree_limit` is deprecated, use `iteration_range` instead.
## [15:56:51] WARNING: src/c_api/c_api.cc:935: `ntree_limit` is deprecated, use `iteration_range` instead.
## [15:56:51] WARNING: src/c_api/c_api.cc:935: `ntree_limit` is deprecated, use `iteration_range` instead.
## [15:56:51] WARNING: src/c_api/c_api.cc:935: `ntree_limit` is deprecated, use `iteration_range` instead.
## [15:56:51] WARNING: src/c_api/c_api.cc:935: `ntree_limit` is deprecated, use `iteration_range` instead.
## [15:56:51] WARNING: src/c_api/c_api.cc:935: `ntree_limit` is deprecated, use `iteration_range` instead.
## [15:56:51] WARNING: src/c_api/c_api.cc:935: `ntree_limit` is deprecated, use `iteration_range` instead.
## [15:56:51] WARNING: src/c_api/c_api.cc:935: `ntree_limit` is deprecated, use `iteration_range` instead.
## [15:56:51] WARNING: src/c_api/c_api.cc:935: `ntree_limit` is deprecated, use `iteration_range` instead.
## [15:56:52] WARNING: src/c_api/c_api.cc:935: `ntree_limit` is deprecated, use `iteration_range` instead.
## [15:56:52] WARNING: src/c_api/c_api.cc:935: `ntree_limit` is deprecated, use `iteration_range` instead.
## [15:56:52] WARNING: src/c_api/c_api.cc:935: `ntree_limit` is deprecated, use `iteration_range` instead.
## [15:56:52] WARNING: src/c_api/c_api.cc:935: `ntree_limit` is deprecated, use `iteration_range` instead.
## [15:56:52] WARNING: src/c_api/c_api.cc:935: `ntree_limit` is deprecated, use `iteration_range` instead.
## [15:56:52] WARNING: src/c_api/c_api.cc:935: `ntree_limit` is deprecated, use `iteration_range` instead.
## [15:56:52] WARNING: src/c_api/c_api.cc:935: `ntree_limit` is deprecated, use `iteration_range` instead.
## [15:56:52] WARNING: src/c_api/c_api.cc:935: `ntree_limit` is deprecated, use `iteration_range` instead.
## [15:56:53] WARNING: src/c_api/c_api.cc:935: `ntree_limit` is deprecated, use `iteration_range` instead.
## [15:56:53] WARNING: src/c_api/c_api.cc:935: `ntree_limit` is deprecated, use `iteration_range` instead.
## [15:56:53] WARNING: src/c_api/c_api.cc:935: `ntree_limit` is deprecated, use `iteration_range` instead.
## [15:56:53] WARNING: src/c_api/c_api.cc:935: `ntree_limit` is deprecated, use `iteration_range` instead.
## [15:56:53] WARNING: src/c_api/c_api.cc:935: `ntree_limit` is deprecated, use `iteration_range` instead.
## [15:56:53] WARNING: src/c_api/c_api.cc:935: `ntree_limit` is deprecated, use `iteration_range` instead.
## [15:56:53] WARNING: src/c_api/c_api.cc:935: `ntree_limit` is deprecated, use `iteration_range` instead.
## [15:56:53] WARNING: src/c_api/c_api.cc:935: `ntree_limit` is deprecated, use `iteration_range` instead.
## [15:56:53] WARNING: src/c_api/c_api.cc:935: `ntree_limit` is deprecated, use `iteration_range` instead.
## [15:56:53] WARNING: src/c_api/c_api.cc:935: `ntree_limit` is deprecated, use `iteration_range` instead.
## [15:56:53] WARNING: src/c_api/c_api.cc:935: `ntree_limit` is deprecated, use `iteration_range` instead.
## [15:56:53] WARNING: src/c_api/c_api.cc:935: `ntree_limit` is deprecated, use `iteration_range` instead.
## [15:56:54] WARNING: src/c_api/c_api.cc:935: `ntree_limit` is deprecated, use `iteration_range` instead.
## [15:56:54] WARNING: src/c_api/c_api.cc:935: `ntree_limit` is deprecated, use `iteration_range` instead.
## [15:56:54] WARNING: src/c_api/c_api.cc:935: `ntree_limit` is deprecated, use `iteration_range` instead.
## [15:56:54] WARNING: src/c_api/c_api.cc:935: `ntree_limit` is deprecated, use `iteration_range` instead.
## [15:56:54] WARNING: src/c_api/c_api.cc:935: `ntree_limit` is deprecated, use `iteration_range` instead.
## [15:56:54] WARNING: src/c_api/c_api.cc:935: `ntree_limit` is deprecated, use `iteration_range` instead.
## [15:56:54] WARNING: src/c_api/c_api.cc:935: `ntree_limit` is deprecated, use `iteration_range` instead.
## [15:56:54] WARNING: src/c_api/c_api.cc:935: `ntree_limit` is deprecated, use `iteration_range` instead.
## [15:56:54] WARNING: src/c_api/c_api.cc:935: `ntree_limit` is deprecated, use `iteration_range` instead.
## [15:56:54] WARNING: src/c_api/c_api.cc:935: `ntree_limit` is deprecated, use `iteration_range` instead.
## [15:56:54] WARNING: src/c_api/c_api.cc:935: `ntree_limit` is deprecated, use `iteration_range` instead.
## [15:56:54] WARNING: src/c_api/c_api.cc:935: `ntree_limit` is deprecated, use `iteration_range` instead.
## [15:56:55] WARNING: src/c_api/c_api.cc:935: `ntree_limit` is deprecated, use `iteration_range` instead.
## [15:56:55] WARNING: src/c_api/c_api.cc:935: `ntree_limit` is deprecated, use `iteration_range` instead.
## [15:56:55] WARNING: src/c_api/c_api.cc:935: `ntree_limit` is deprecated, use `iteration_range` instead.
## [15:56:55] WARNING: src/c_api/c_api.cc:935: `ntree_limit` is deprecated, use `iteration_range` instead.
## [15:56:55] WARNING: src/c_api/c_api.cc:935: `ntree_limit` is deprecated, use `iteration_range` instead.
## [15:56:55] WARNING: src/c_api/c_api.cc:935: `ntree_limit` is deprecated, use `iteration_range` instead.
## [15:56:55] WARNING: src/c_api/c_api.cc:935: `ntree_limit` is deprecated, use `iteration_range` instead.
## [15:56:55] WARNING: src/c_api/c_api.cc:935: `ntree_limit` is deprecated, use `iteration_range` instead.
## [15:56:55] WARNING: src/c_api/c_api.cc:935: `ntree_limit` is deprecated, use `iteration_range` instead.
## [15:56:55] WARNING: src/c_api/c_api.cc:935: `ntree_limit` is deprecated, use `iteration_range` instead.
## [15:56:56] WARNING: src/c_api/c_api.cc:935: `ntree_limit` is deprecated, use `iteration_range` instead.
## [15:56:56] WARNING: src/c_api/c_api.cc:935: `ntree_limit` is deprecated, use `iteration_range` instead.
## [15:56:56] WARNING: src/c_api/c_api.cc:935: `ntree_limit` is deprecated, use `iteration_range` instead.
## [15:56:56] WARNING: src/c_api/c_api.cc:935: `ntree_limit` is deprecated, use `iteration_range` instead.
## [15:56:56] WARNING: src/c_api/c_api.cc:935: `ntree_limit` is deprecated, use `iteration_range` instead.
## [15:56:56] WARNING: src/c_api/c_api.cc:935: `ntree_limit` is deprecated, use `iteration_range` instead.
## [15:56:56] WARNING: src/c_api/c_api.cc:935: `ntree_limit` is deprecated, use `iteration_range` instead.
## [15:56:56] WARNING: src/c_api/c_api.cc:935: `ntree_limit` is deprecated, use `iteration_range` instead.
## [15:56:56] WARNING: src/c_api/c_api.cc:935: `ntree_limit` is deprecated, use `iteration_range` instead.
## [15:56:56] WARNING: src/c_api/c_api.cc:935: `ntree_limit` is deprecated, use `iteration_range` instead.
## [15:56:56] WARNING: src/c_api/c_api.cc:935: `ntree_limit` is deprecated, use `iteration_range` instead.
## [15:56:56] WARNING: src/c_api/c_api.cc:935: `ntree_limit` is deprecated, use `iteration_range` instead.
## [15:56:57] WARNING: src/c_api/c_api.cc:935: `ntree_limit` is deprecated, use `iteration_range` instead.
## [15:56:57] WARNING: src/c_api/c_api.cc:935: `ntree_limit` is deprecated, use `iteration_range` instead.
## [15:56:57] WARNING: src/c_api/c_api.cc:935: `ntree_limit` is deprecated, use `iteration_range` instead.
## [15:56:57] WARNING: src/c_api/c_api.cc:935: `ntree_limit` is deprecated, use `iteration_range` instead.
## [15:56:57] WARNING: src/c_api/c_api.cc:935: `ntree_limit` is deprecated, use `iteration_range` instead.
## [15:56:57] WARNING: src/c_api/c_api.cc:935: `ntree_limit` is deprecated, use `iteration_range` instead.
## [15:56:57] WARNING: src/c_api/c_api.cc:935: `ntree_limit` is deprecated, use `iteration_range` instead.
## [15:56:57] WARNING: src/c_api/c_api.cc:935: `ntree_limit` is deprecated, use `iteration_range` instead.
## [15:56:57] WARNING: src/c_api/c_api.cc:935: `ntree_limit` is deprecated, use `iteration_range` instead.
## [15:56:57] WARNING: src/c_api/c_api.cc:935: `ntree_limit` is deprecated, use `iteration_range` instead.
## [15:56:57] WARNING: src/c_api/c_api.cc:935: `ntree_limit` is deprecated, use `iteration_range` instead.
## [15:56:57] WARNING: src/c_api/c_api.cc:935: `ntree_limit` is deprecated, use `iteration_range` instead.
## [15:56:58] WARNING: src/c_api/c_api.cc:935: `ntree_limit` is deprecated, use `iteration_range` instead.
## [15:56:58] WARNING: src/c_api/c_api.cc:935: `ntree_limit` is deprecated, use `iteration_range` instead.
## [15:56:58] WARNING: src/c_api/c_api.cc:935: `ntree_limit` is deprecated, use `iteration_range` instead.
## [15:56:58] WARNING: src/c_api/c_api.cc:935: `ntree_limit` is deprecated, use `iteration_range` instead.
## [15:56:58] WARNING: src/c_api/c_api.cc:935: `ntree_limit` is deprecated, use `iteration_range` instead.
## [15:56:58] WARNING: src/c_api/c_api.cc:935: `ntree_limit` is deprecated, use `iteration_range` instead.
## [15:56:58] WARNING: src/c_api/c_api.cc:935: `ntree_limit` is deprecated, use `iteration_range` instead.
## [15:56:58] WARNING: src/c_api/c_api.cc:935: `ntree_limit` is deprecated, use `iteration_range` instead.
## [15:56:58] WARNING: src/c_api/c_api.cc:935: `ntree_limit` is deprecated, use `iteration_range` instead.
## [15:56:58] WARNING: src/c_api/c_api.cc:935: `ntree_limit` is deprecated, use `iteration_range` instead.
## [15:56:59] WARNING: src/c_api/c_api.cc:935: `ntree_limit` is deprecated, use `iteration_range` instead.
## [15:56:59] WARNING: src/c_api/c_api.cc:935: `ntree_limit` is deprecated, use `iteration_range` instead.
## [15:56:59] WARNING: src/c_api/c_api.cc:935: `ntree_limit` is deprecated, use `iteration_range` instead.
## [15:56:59] WARNING: src/c_api/c_api.cc:935: `ntree_limit` is deprecated, use `iteration_range` instead.
## [15:56:59] WARNING: src/c_api/c_api.cc:935: `ntree_limit` is deprecated, use `iteration_range` instead.
## [15:56:59] WARNING: src/c_api/c_api.cc:935: `ntree_limit` is deprecated, use `iteration_range` instead.
## [15:56:59] WARNING: src/c_api/c_api.cc:935: `ntree_limit` is deprecated, use `iteration_range` instead.
## [15:56:59] WARNING: src/c_api/c_api.cc:935: `ntree_limit` is deprecated, use `iteration_range` instead.
## [15:56:59] WARNING: src/c_api/c_api.cc:935: `ntree_limit` is deprecated, use `iteration_range` instead.
## [15:56:59] WARNING: src/c_api/c_api.cc:935: `ntree_limit` is deprecated, use `iteration_range` instead.
## [15:57:00] WARNING: src/c_api/c_api.cc:935: `ntree_limit` is deprecated, use `iteration_range` instead.
## [15:57:00] WARNING: src/c_api/c_api.cc:935: `ntree_limit` is deprecated, use `iteration_range` instead.
## [15:57:00] WARNING: src/c_api/c_api.cc:935: `ntree_limit` is deprecated, use `iteration_range` instead.
## [15:57:00] WARNING: src/c_api/c_api.cc:935: `ntree_limit` is deprecated, use `iteration_range` instead.
## [15:57:00] WARNING: src/c_api/c_api.cc:935: `ntree_limit` is deprecated, use `iteration_range` instead.
## [15:57:00] WARNING: src/c_api/c_api.cc:935: `ntree_limit` is deprecated, use `iteration_range` instead.
## [15:57:00] WARNING: src/c_api/c_api.cc:935: `ntree_limit` is deprecated, use `iteration_range` instead.
## [15:57:00] WARNING: src/c_api/c_api.cc:935: `ntree_limit` is deprecated, use `iteration_range` instead.
## [15:57:00] WARNING: src/c_api/c_api.cc:935: `ntree_limit` is deprecated, use `iteration_range` instead.
## [15:57:00] WARNING: src/c_api/c_api.cc:935: `ntree_limit` is deprecated, use `iteration_range` instead.
## [15:57:00] WARNING: src/c_api/c_api.cc:935: `ntree_limit` is deprecated, use `iteration_range` instead.
## [15:57:00] WARNING: src/c_api/c_api.cc:935: `ntree_limit` is deprecated, use `iteration_range` instead.
## [15:57:00] WARNING: src/c_api/c_api.cc:935: `ntree_limit` is deprecated, use `iteration_range` instead.
## [15:57:00] WARNING: src/c_api/c_api.cc:935: `ntree_limit` is deprecated, use `iteration_range` instead.
## [15:57:01] WARNING: src/c_api/c_api.cc:935: `ntree_limit` is deprecated, use `iteration_range` instead.
## [15:57:01] WARNING: src/c_api/c_api.cc:935: `ntree_limit` is deprecated, use `iteration_range` instead.
## [15:57:01] WARNING: src/c_api/c_api.cc:935: `ntree_limit` is deprecated, use `iteration_range` instead.
## [15:57:01] WARNING: src/c_api/c_api.cc:935: `ntree_limit` is deprecated, use `iteration_range` instead.
## [15:57:01] WARNING: src/c_api/c_api.cc:935: `ntree_limit` is deprecated, use `iteration_range` instead.
## [15:57:01] WARNING: src/c_api/c_api.cc:935: `ntree_limit` is deprecated, use `iteration_range` instead.
## [15:57:01] WARNING: src/c_api/c_api.cc:935: `ntree_limit` is deprecated, use `iteration_range` instead.
## [15:57:01] WARNING: src/c_api/c_api.cc:935: `ntree_limit` is deprecated, use `iteration_range` instead.
## [15:57:01] WARNING: src/c_api/c_api.cc:935: `ntree_limit` is deprecated, use `iteration_range` instead.
## [15:57:01] WARNING: src/c_api/c_api.cc:935: `ntree_limit` is deprecated, use `iteration_range` instead.
## [15:57:02] WARNING: src/c_api/c_api.cc:935: `ntree_limit` is deprecated, use `iteration_range` instead.
## [15:57:02] WARNING: src/c_api/c_api.cc:935: `ntree_limit` is deprecated, use `iteration_range` instead.
## [15:57:02] WARNING: src/c_api/c_api.cc:935: `ntree_limit` is deprecated, use `iteration_range` instead.
## [15:57:02] WARNING: src/c_api/c_api.cc:935: `ntree_limit` is deprecated, use `iteration_range` instead.
## [15:57:02] WARNING: src/c_api/c_api.cc:935: `ntree_limit` is deprecated, use `iteration_range` instead.
## [15:57:02] WARNING: src/c_api/c_api.cc:935: `ntree_limit` is deprecated, use `iteration_range` instead.
## [15:57:02] WARNING: src/c_api/c_api.cc:935: `ntree_limit` is deprecated, use `iteration_range` instead.
## [15:57:02] WARNING: src/c_api/c_api.cc:935: `ntree_limit` is deprecated, use `iteration_range` instead.
## [15:57:02] WARNING: src/c_api/c_api.cc:935: `ntree_limit` is deprecated, use `iteration_range` instead.
## [15:57:02] WARNING: src/c_api/c_api.cc:935: `ntree_limit` is deprecated, use `iteration_range` instead.
## [15:57:03] WARNING: src/c_api/c_api.cc:935: `ntree_limit` is deprecated, use `iteration_range` instead.
## [15:57:03] WARNING: src/c_api/c_api.cc:935: `ntree_limit` is deprecated, use `iteration_range` instead.
## [15:57:03] WARNING: src/c_api/c_api.cc:935: `ntree_limit` is deprecated, use `iteration_range` instead.
## [15:57:03] WARNING: src/c_api/c_api.cc:935: `ntree_limit` is deprecated, use `iteration_range` instead.
## [15:57:03] WARNING: src/c_api/c_api.cc:935: `ntree_limit` is deprecated, use `iteration_range` instead.
## [15:57:03] WARNING: src/c_api/c_api.cc:935: `ntree_limit` is deprecated, use `iteration_range` instead.
## [15:57:03] WARNING: src/c_api/c_api.cc:935: `ntree_limit` is deprecated, use `iteration_range` instead.
## [15:57:03] WARNING: src/c_api/c_api.cc:935: `ntree_limit` is deprecated, use `iteration_range` instead.
## [15:57:03] WARNING: src/c_api/c_api.cc:935: `ntree_limit` is deprecated, use `iteration_range` instead.
## [15:57:03] WARNING: src/c_api/c_api.cc:935: `ntree_limit` is deprecated, use `iteration_range` instead.
## [15:57:03] WARNING: src/c_api/c_api.cc:935: `ntree_limit` is deprecated, use `iteration_range` instead.
## [15:57:03] WARNING: src/c_api/c_api.cc:935: `ntree_limit` is deprecated, use `iteration_range` instead.
## [15:57:04] WARNING: src/c_api/c_api.cc:935: `ntree_limit` is deprecated, use `iteration_range` instead.
## [15:57:04] WARNING: src/c_api/c_api.cc:935: `ntree_limit` is deprecated, use `iteration_range` instead.
## [15:57:04] WARNING: src/c_api/c_api.cc:935: `ntree_limit` is deprecated, use `iteration_range` instead.
## [15:57:04] WARNING: src/c_api/c_api.cc:935: `ntree_limit` is deprecated, use `iteration_range` instead.
## [15:57:04] WARNING: src/c_api/c_api.cc:935: `ntree_limit` is deprecated, use `iteration_range` instead.
## [15:57:04] WARNING: src/c_api/c_api.cc:935: `ntree_limit` is deprecated, use `iteration_range` instead.
## [15:57:04] WARNING: src/c_api/c_api.cc:935: `ntree_limit` is deprecated, use `iteration_range` instead.
## [15:57:04] WARNING: src/c_api/c_api.cc:935: `ntree_limit` is deprecated, use `iteration_range` instead.
## [15:57:04] WARNING: src/c_api/c_api.cc:935: `ntree_limit` is deprecated, use `iteration_range` instead.
## [15:57:04] WARNING: src/c_api/c_api.cc:935: `ntree_limit` is deprecated, use `iteration_range` instead.
## [15:57:05] WARNING: src/c_api/c_api.cc:935: `ntree_limit` is deprecated, use `iteration_range` instead.
## [15:57:05] WARNING: src/c_api/c_api.cc:935: `ntree_limit` is deprecated, use `iteration_range` instead.
## [15:57:05] WARNING: src/c_api/c_api.cc:935: `ntree_limit` is deprecated, use `iteration_range` instead.
## [15:57:05] WARNING: src/c_api/c_api.cc:935: `ntree_limit` is deprecated, use `iteration_range` instead.
## [15:57:05] WARNING: src/c_api/c_api.cc:935: `ntree_limit` is deprecated, use `iteration_range` instead.
## [15:57:05] WARNING: src/c_api/c_api.cc:935: `ntree_limit` is deprecated, use `iteration_range` instead.
## [15:57:05] WARNING: src/c_api/c_api.cc:935: `ntree_limit` is deprecated, use `iteration_range` instead.
## [15:57:05] WARNING: src/c_api/c_api.cc:935: `ntree_limit` is deprecated, use `iteration_range` instead.
## [15:57:06] WARNING: src/c_api/c_api.cc:935: `ntree_limit` is deprecated, use `iteration_range` instead.
## [15:57:06] WARNING: src/c_api/c_api.cc:935: `ntree_limit` is deprecated, use `iteration_range` instead.
## [15:57:06] WARNING: src/c_api/c_api.cc:935: `ntree_limit` is deprecated, use `iteration_range` instead.
## [15:57:06] WARNING: src/c_api/c_api.cc:935: `ntree_limit` is deprecated, use `iteration_range` instead.
## [15:57:06] WARNING: src/c_api/c_api.cc:935: `ntree_limit` is deprecated, use `iteration_range` instead.
## [15:57:06] WARNING: src/c_api/c_api.cc:935: `ntree_limit` is deprecated, use `iteration_range` instead.
## [15:57:06] WARNING: src/c_api/c_api.cc:935: `ntree_limit` is deprecated, use `iteration_range` instead.
## [15:57:06] WARNING: src/c_api/c_api.cc:935: `ntree_limit` is deprecated, use `iteration_range` instead.
## [15:57:06] WARNING: src/c_api/c_api.cc:935: `ntree_limit` is deprecated, use `iteration_range` instead.
## [15:57:06] WARNING: src/c_api/c_api.cc:935: `ntree_limit` is deprecated, use `iteration_range` instead.
## [15:57:06] WARNING: src/c_api/c_api.cc:935: `ntree_limit` is deprecated, use `iteration_range` instead.
## [15:57:06] WARNING: src/c_api/c_api.cc:935: `ntree_limit` is deprecated, use `iteration_range` instead.
## [15:57:06] WARNING: src/c_api/c_api.cc:935: `ntree_limit` is deprecated, use `iteration_range` instead.
## [15:57:06] WARNING: src/c_api/c_api.cc:935: `ntree_limit` is deprecated, use `iteration_range` instead.
## [15:57:07] WARNING: src/c_api/c_api.cc:935: `ntree_limit` is deprecated, use `iteration_range` instead.
## [15:57:07] WARNING: src/c_api/c_api.cc:935: `ntree_limit` is deprecated, use `iteration_range` instead.
## [15:57:07] WARNING: src/c_api/c_api.cc:935: `ntree_limit` is deprecated, use `iteration_range` instead.
## [15:57:07] WARNING: src/c_api/c_api.cc:935: `ntree_limit` is deprecated, use `iteration_range` instead.
## [15:57:07] WARNING: src/c_api/c_api.cc:935: `ntree_limit` is deprecated, use `iteration_range` instead.
## [15:57:07] WARNING: src/c_api/c_api.cc:935: `ntree_limit` is deprecated, use `iteration_range` instead.
## [15:57:07] WARNING: src/c_api/c_api.cc:935: `ntree_limit` is deprecated, use `iteration_range` instead.
## [15:57:07] WARNING: src/c_api/c_api.cc:935: `ntree_limit` is deprecated, use `iteration_range` instead.
## [15:57:07] WARNING: src/c_api/c_api.cc:935: `ntree_limit` is deprecated, use `iteration_range` instead.
## [15:57:07] WARNING: src/c_api/c_api.cc:935: `ntree_limit` is deprecated, use `iteration_range` instead.
## [15:57:07] WARNING: src/c_api/c_api.cc:935: `ntree_limit` is deprecated, use `iteration_range` instead.
## [15:57:07] WARNING: src/c_api/c_api.cc:935: `ntree_limit` is deprecated, use `iteration_range` instead.
## [15:57:08] WARNING: src/c_api/c_api.cc:935: `ntree_limit` is deprecated, use `iteration_range` instead.
## [15:57:08] WARNING: src/c_api/c_api.cc:935: `ntree_limit` is deprecated, use `iteration_range` instead.
## [15:57:08] WARNING: src/c_api/c_api.cc:935: `ntree_limit` is deprecated, use `iteration_range` instead.
## [15:57:08] WARNING: src/c_api/c_api.cc:935: `ntree_limit` is deprecated, use `iteration_range` instead.
## [15:57:08] WARNING: src/c_api/c_api.cc:935: `ntree_limit` is deprecated, use `iteration_range` instead.
## [15:57:08] WARNING: src/c_api/c_api.cc:935: `ntree_limit` is deprecated, use `iteration_range` instead.
## [15:57:08] WARNING: src/c_api/c_api.cc:935: `ntree_limit` is deprecated, use `iteration_range` instead.
## [15:57:08] WARNING: src/c_api/c_api.cc:935: `ntree_limit` is deprecated, use `iteration_range` instead.
## [15:57:08] WARNING: src/c_api/c_api.cc:935: `ntree_limit` is deprecated, use `iteration_range` instead.
## [15:57:08] WARNING: src/c_api/c_api.cc:935: `ntree_limit` is deprecated, use `iteration_range` instead.
## [15:57:09] WARNING: src/c_api/c_api.cc:935: `ntree_limit` is deprecated, use `iteration_range` instead.
## [15:57:09] WARNING: src/c_api/c_api.cc:935: `ntree_limit` is deprecated, use `iteration_range` instead.
## [15:57:09] WARNING: src/c_api/c_api.cc:935: `ntree_limit` is deprecated, use `iteration_range` instead.
## [15:57:09] WARNING: src/c_api/c_api.cc:935: `ntree_limit` is deprecated, use `iteration_range` instead.
## [15:57:09] WARNING: src/c_api/c_api.cc:935: `ntree_limit` is deprecated, use `iteration_range` instead.
## [15:57:09] WARNING: src/c_api/c_api.cc:935: `ntree_limit` is deprecated, use `iteration_range` instead.
## [15:57:09] WARNING: src/c_api/c_api.cc:935: `ntree_limit` is deprecated, use `iteration_range` instead.
## [15:57:09] WARNING: src/c_api/c_api.cc:935: `ntree_limit` is deprecated, use `iteration_range` instead.
## [15:57:09] WARNING: src/c_api/c_api.cc:935: `ntree_limit` is deprecated, use `iteration_range` instead.
## [15:57:09] WARNING: src/c_api/c_api.cc:935: `ntree_limit` is deprecated, use `iteration_range` instead.
## [15:57:10] WARNING: src/c_api/c_api.cc:935: `ntree_limit` is deprecated, use `iteration_range` instead.
## [15:57:10] WARNING: src/c_api/c_api.cc:935: `ntree_limit` is deprecated, use `iteration_range` instead.
## [15:57:10] WARNING: src/c_api/c_api.cc:935: `ntree_limit` is deprecated, use `iteration_range` instead.
## [15:57:10] WARNING: src/c_api/c_api.cc:935: `ntree_limit` is deprecated, use `iteration_range` instead.
## [15:57:10] WARNING: src/c_api/c_api.cc:935: `ntree_limit` is deprecated, use `iteration_range` instead.
## [15:57:10] WARNING: src/c_api/c_api.cc:935: `ntree_limit` is deprecated, use `iteration_range` instead.
## [15:57:10] WARNING: src/c_api/c_api.cc:935: `ntree_limit` is deprecated, use `iteration_range` instead.
## [15:57:10] WARNING: src/c_api/c_api.cc:935: `ntree_limit` is deprecated, use `iteration_range` instead.
## [15:57:10] WARNING: src/c_api/c_api.cc:935: `ntree_limit` is deprecated, use `iteration_range` instead.
## [15:57:10] WARNING: src/c_api/c_api.cc:935: `ntree_limit` is deprecated, use `iteration_range` instead.
## [15:57:10] WARNING: src/c_api/c_api.cc:935: `ntree_limit` is deprecated, use `iteration_range` instead.
## [15:57:10] WARNING: src/c_api/c_api.cc:935: `ntree_limit` is deprecated, use `iteration_range` instead.
## [15:57:10] WARNING: src/c_api/c_api.cc:935: `ntree_limit` is deprecated, use `iteration_range` instead.
## [15:57:10] WARNING: src/c_api/c_api.cc:935: `ntree_limit` is deprecated, use `iteration_range` instead.
## [15:57:11] WARNING: src/c_api/c_api.cc:935: `ntree_limit` is deprecated, use `iteration_range` instead.
## [15:57:11] WARNING: src/c_api/c_api.cc:935: `ntree_limit` is deprecated, use `iteration_range` instead.
## [15:57:11] WARNING: src/c_api/c_api.cc:935: `ntree_limit` is deprecated, use `iteration_range` instead.
## [15:57:11] WARNING: src/c_api/c_api.cc:935: `ntree_limit` is deprecated, use `iteration_range` instead.
## [15:57:11] WARNING: src/c_api/c_api.cc:935: `ntree_limit` is deprecated, use `iteration_range` instead.
## [15:57:11] WARNING: src/c_api/c_api.cc:935: `ntree_limit` is deprecated, use `iteration_range` instead.
## [15:57:11] WARNING: src/c_api/c_api.cc:935: `ntree_limit` is deprecated, use `iteration_range` instead.
## [15:57:11] WARNING: src/c_api/c_api.cc:935: `ntree_limit` is deprecated, use `iteration_range` instead.
## [15:57:11] WARNING: src/c_api/c_api.cc:935: `ntree_limit` is deprecated, use `iteration_range` instead.
## [15:57:11] WARNING: src/c_api/c_api.cc:935: `ntree_limit` is deprecated, use `iteration_range` instead.
## [15:57:12] WARNING: src/c_api/c_api.cc:935: `ntree_limit` is deprecated, use `iteration_range` instead.
## [15:57:12] WARNING: src/c_api/c_api.cc:935: `ntree_limit` is deprecated, use `iteration_range` instead.
## [15:57:12] WARNING: src/c_api/c_api.cc:935: `ntree_limit` is deprecated, use `iteration_range` instead.
## [15:57:12] WARNING: src/c_api/c_api.cc:935: `ntree_limit` is deprecated, use `iteration_range` instead.
## [15:57:12] WARNING: src/c_api/c_api.cc:935: `ntree_limit` is deprecated, use `iteration_range` instead.
## [15:57:12] WARNING: src/c_api/c_api.cc:935: `ntree_limit` is deprecated, use `iteration_range` instead.
## [15:57:12] WARNING: src/c_api/c_api.cc:935: `ntree_limit` is deprecated, use `iteration_range` instead.
## [15:57:12] WARNING: src/c_api/c_api.cc:935: `ntree_limit` is deprecated, use `iteration_range` instead.
## [15:57:13] WARNING: src/c_api/c_api.cc:935: `ntree_limit` is deprecated, use `iteration_range` instead.
## [15:57:13] WARNING: src/c_api/c_api.cc:935: `ntree_limit` is deprecated, use `iteration_range` instead.
## [15:57:13] WARNING: src/c_api/c_api.cc:935: `ntree_limit` is deprecated, use `iteration_range` instead.
## [15:57:13] WARNING: src/c_api/c_api.cc:935: `ntree_limit` is deprecated, use `iteration_range` instead.
## [15:57:13] WARNING: src/c_api/c_api.cc:935: `ntree_limit` is deprecated, use `iteration_range` instead.
## [15:57:13] WARNING: src/c_api/c_api.cc:935: `ntree_limit` is deprecated, use `iteration_range` instead.
## [15:57:13] WARNING: src/c_api/c_api.cc:935: `ntree_limit` is deprecated, use `iteration_range` instead.
## [15:57:13] WARNING: src/c_api/c_api.cc:935: `ntree_limit` is deprecated, use `iteration_range` instead.
## [15:57:13] WARNING: src/c_api/c_api.cc:935: `ntree_limit` is deprecated, use `iteration_range` instead.
## [15:57:13] WARNING: src/c_api/c_api.cc:935: `ntree_limit` is deprecated, use `iteration_range` instead.
## [15:57:13] WARNING: src/c_api/c_api.cc:935: `ntree_limit` is deprecated, use `iteration_range` instead.
## [15:57:13] WARNING: src/c_api/c_api.cc:935: `ntree_limit` is deprecated, use `iteration_range` instead.
## [15:57:13] WARNING: src/c_api/c_api.cc:935: `ntree_limit` is deprecated, use `iteration_range` instead.
## [15:57:13] WARNING: src/c_api/c_api.cc:935: `ntree_limit` is deprecated, use `iteration_range` instead.
## [15:57:14] WARNING: src/c_api/c_api.cc:935: `ntree_limit` is deprecated, use `iteration_range` instead.
## [15:57:14] WARNING: src/c_api/c_api.cc:935: `ntree_limit` is deprecated, use `iteration_range` instead.
## [15:57:14] WARNING: src/c_api/c_api.cc:935: `ntree_limit` is deprecated, use `iteration_range` instead.
## [15:57:14] WARNING: src/c_api/c_api.cc:935: `ntree_limit` is deprecated, use `iteration_range` instead.
## [15:57:14] WARNING: src/c_api/c_api.cc:935: `ntree_limit` is deprecated, use `iteration_range` instead.
## [15:57:14] WARNING: src/c_api/c_api.cc:935: `ntree_limit` is deprecated, use `iteration_range` instead.
## [15:57:14] WARNING: src/c_api/c_api.cc:935: `ntree_limit` is deprecated, use `iteration_range` instead.
## [15:57:14] WARNING: src/c_api/c_api.cc:935: `ntree_limit` is deprecated, use `iteration_range` instead.
## [15:57:14] WARNING: src/c_api/c_api.cc:935: `ntree_limit` is deprecated, use `iteration_range` instead.
## [15:57:14] WARNING: src/c_api/c_api.cc:935: `ntree_limit` is deprecated, use `iteration_range` instead.
## [15:57:15] WARNING: src/c_api/c_api.cc:935: `ntree_limit` is deprecated, use `iteration_range` instead.
## [15:57:15] WARNING: src/c_api/c_api.cc:935: `ntree_limit` is deprecated, use `iteration_range` instead.
## [15:57:15] WARNING: src/c_api/c_api.cc:935: `ntree_limit` is deprecated, use `iteration_range` instead.
## [15:57:15] WARNING: src/c_api/c_api.cc:935: `ntree_limit` is deprecated, use `iteration_range` instead.
## [15:57:15] WARNING: src/c_api/c_api.cc:935: `ntree_limit` is deprecated, use `iteration_range` instead.
## [15:57:15] WARNING: src/c_api/c_api.cc:935: `ntree_limit` is deprecated, use `iteration_range` instead.
## [15:57:15] WARNING: src/c_api/c_api.cc:935: `ntree_limit` is deprecated, use `iteration_range` instead.
## [15:57:15] WARNING: src/c_api/c_api.cc:935: `ntree_limit` is deprecated, use `iteration_range` instead.
## [15:57:15] WARNING: src/c_api/c_api.cc:935: `ntree_limit` is deprecated, use `iteration_range` instead.
## [15:57:15] WARNING: src/c_api/c_api.cc:935: `ntree_limit` is deprecated, use `iteration_range` instead.
## [15:57:16] WARNING: src/c_api/c_api.cc:935: `ntree_limit` is deprecated, use `iteration_range` instead.
## [15:57:16] WARNING: src/c_api/c_api.cc:935: `ntree_limit` is deprecated, use `iteration_range` instead.
## [15:57:19] WARNING: src/c_api/c_api.cc:935: `ntree_limit` is deprecated, use `iteration_range` instead.
## [15:57:19] WARNING: src/c_api/c_api.cc:935: `ntree_limit` is deprecated, use `iteration_range` instead.
## [15:57:19] WARNING: src/c_api/c_api.cc:935: `ntree_limit` is deprecated, use `iteration_range` instead.
## [15:57:19] WARNING: src/c_api/c_api.cc:935: `ntree_limit` is deprecated, use `iteration_range` instead.
## [15:57:19] WARNING: src/c_api/c_api.cc:935: `ntree_limit` is deprecated, use `iteration_range` instead.
## [15:57:19] WARNING: src/c_api/c_api.cc:935: `ntree_limit` is deprecated, use `iteration_range` instead.
## [15:57:19] WARNING: src/c_api/c_api.cc:935: `ntree_limit` is deprecated, use `iteration_range` instead.
## [15:57:19] WARNING: src/c_api/c_api.cc:935: `ntree_limit` is deprecated, use `iteration_range` instead.
## [15:57:19] WARNING: src/c_api/c_api.cc:935: `ntree_limit` is deprecated, use `iteration_range` instead.
## [15:57:19] WARNING: src/c_api/c_api.cc:935: `ntree_limit` is deprecated, use `iteration_range` instead.
## [15:57:19] WARNING: src/c_api/c_api.cc:935: `ntree_limit` is deprecated, use `iteration_range` instead.
## [15:57:19] WARNING: src/c_api/c_api.cc:935: `ntree_limit` is deprecated, use `iteration_range` instead.
## [15:57:19] WARNING: src/c_api/c_api.cc:935: `ntree_limit` is deprecated, use `iteration_range` instead.
## [15:57:19] WARNING: src/c_api/c_api.cc:935: `ntree_limit` is deprecated, use `iteration_range` instead.
## [15:57:20] WARNING: src/c_api/c_api.cc:935: `ntree_limit` is deprecated, use `iteration_range` instead.
## [15:57:20] WARNING: src/c_api/c_api.cc:935: `ntree_limit` is deprecated, use `iteration_range` instead.
## [15:57:20] WARNING: src/c_api/c_api.cc:935: `ntree_limit` is deprecated, use `iteration_range` instead.
## [15:57:20] WARNING: src/c_api/c_api.cc:935: `ntree_limit` is deprecated, use `iteration_range` instead.
## [15:57:20] WARNING: src/c_api/c_api.cc:935: `ntree_limit` is deprecated, use `iteration_range` instead.
## [15:57:20] WARNING: src/c_api/c_api.cc:935: `ntree_limit` is deprecated, use `iteration_range` instead.
## [15:57:20] WARNING: src/c_api/c_api.cc:935: `ntree_limit` is deprecated, use `iteration_range` instead.
## [15:57:20] WARNING: src/c_api/c_api.cc:935: `ntree_limit` is deprecated, use `iteration_range` instead.
## [15:57:20] WARNING: src/c_api/c_api.cc:935: `ntree_limit` is deprecated, use `iteration_range` instead.
## [15:57:20] WARNING: src/c_api/c_api.cc:935: `ntree_limit` is deprecated, use `iteration_range` instead.
## [15:57:21] WARNING: src/c_api/c_api.cc:935: `ntree_limit` is deprecated, use `iteration_range` instead.
## [15:57:21] WARNING: src/c_api/c_api.cc:935: `ntree_limit` is deprecated, use `iteration_range` instead.
## [15:57:21] WARNING: src/c_api/c_api.cc:935: `ntree_limit` is deprecated, use `iteration_range` instead.
## [15:57:21] WARNING: src/c_api/c_api.cc:935: `ntree_limit` is deprecated, use `iteration_range` instead.
## [15:57:21] WARNING: src/c_api/c_api.cc:935: `ntree_limit` is deprecated, use `iteration_range` instead.
## [15:57:21] WARNING: src/c_api/c_api.cc:935: `ntree_limit` is deprecated, use `iteration_range` instead.
## [15:57:21] WARNING: src/c_api/c_api.cc:935: `ntree_limit` is deprecated, use `iteration_range` instead.
## [15:57:21] WARNING: src/c_api/c_api.cc:935: `ntree_limit` is deprecated, use `iteration_range` instead.
## [15:57:21] WARNING: src/c_api/c_api.cc:935: `ntree_limit` is deprecated, use `iteration_range` instead.
## [15:57:21] WARNING: src/c_api/c_api.cc:935: `ntree_limit` is deprecated, use `iteration_range` instead.
## [15:57:22] WARNING: src/c_api/c_api.cc:935: `ntree_limit` is deprecated, use `iteration_range` instead.
## [15:57:22] WARNING: src/c_api/c_api.cc:935: `ntree_limit` is deprecated, use `iteration_range` instead.
## [15:57:22] WARNING: src/c_api/c_api.cc:935: `ntree_limit` is deprecated, use `iteration_range` instead.
## [15:57:22] WARNING: src/c_api/c_api.cc:935: `ntree_limit` is deprecated, use `iteration_range` instead.
## [15:57:22] WARNING: src/c_api/c_api.cc:935: `ntree_limit` is deprecated, use `iteration_range` instead.
## [15:57:22] WARNING: src/c_api/c_api.cc:935: `ntree_limit` is deprecated, use `iteration_range` instead.
## [15:57:22] WARNING: src/c_api/c_api.cc:935: `ntree_limit` is deprecated, use `iteration_range` instead.
## [15:57:22] WARNING: src/c_api/c_api.cc:935: `ntree_limit` is deprecated, use `iteration_range` instead.
## [15:57:22] WARNING: src/c_api/c_api.cc:935: `ntree_limit` is deprecated, use `iteration_range` instead.
## [15:57:22] WARNING: src/c_api/c_api.cc:935: `ntree_limit` is deprecated, use `iteration_range` instead.
## [15:57:22] WARNING: src/c_api/c_api.cc:935: `ntree_limit` is deprecated, use `iteration_range` instead.
## [15:57:22] WARNING: src/c_api/c_api.cc:935: `ntree_limit` is deprecated, use `iteration_range` instead.
## [15:57:22] WARNING: src/c_api/c_api.cc:935: `ntree_limit` is deprecated, use `iteration_range` instead.
## [15:57:22] WARNING: src/c_api/c_api.cc:935: `ntree_limit` is deprecated, use `iteration_range` instead.
## [15:57:23] WARNING: src/c_api/c_api.cc:935: `ntree_limit` is deprecated, use `iteration_range` instead.
## [15:57:23] WARNING: src/c_api/c_api.cc:935: `ntree_limit` is deprecated, use `iteration_range` instead.
## [15:57:23] WARNING: src/c_api/c_api.cc:935: `ntree_limit` is deprecated, use `iteration_range` instead.
## [15:57:23] WARNING: src/c_api/c_api.cc:935: `ntree_limit` is deprecated, use `iteration_range` instead.
## [15:57:23] WARNING: src/c_api/c_api.cc:935: `ntree_limit` is deprecated, use `iteration_range` instead.
## [15:57:23] WARNING: src/c_api/c_api.cc:935: `ntree_limit` is deprecated, use `iteration_range` instead.
## [15:57:23] WARNING: src/c_api/c_api.cc:935: `ntree_limit` is deprecated, use `iteration_range` instead.
## [15:57:23] WARNING: src/c_api/c_api.cc:935: `ntree_limit` is deprecated, use `iteration_range` instead.
## [15:57:23] WARNING: src/c_api/c_api.cc:935: `ntree_limit` is deprecated, use `iteration_range` instead.
## [15:57:23] WARNING: src/c_api/c_api.cc:935: `ntree_limit` is deprecated, use `iteration_range` instead.
## [15:57:24] WARNING: src/c_api/c_api.cc:935: `ntree_limit` is deprecated, use `iteration_range` instead.
## [15:57:24] WARNING: src/c_api/c_api.cc:935: `ntree_limit` is deprecated, use `iteration_range` instead.
## [15:57:24] WARNING: src/c_api/c_api.cc:935: `ntree_limit` is deprecated, use `iteration_range` instead.
## [15:57:24] WARNING: src/c_api/c_api.cc:935: `ntree_limit` is deprecated, use `iteration_range` instead.
## [15:57:24] WARNING: src/c_api/c_api.cc:935: `ntree_limit` is deprecated, use `iteration_range` instead.
## [15:57:24] WARNING: src/c_api/c_api.cc:935: `ntree_limit` is deprecated, use `iteration_range` instead.
## [15:57:24] WARNING: src/c_api/c_api.cc:935: `ntree_limit` is deprecated, use `iteration_range` instead.
## [15:57:24] WARNING: src/c_api/c_api.cc:935: `ntree_limit` is deprecated, use `iteration_range` instead.
## [15:57:25] WARNING: src/c_api/c_api.cc:935: `ntree_limit` is deprecated, use `iteration_range` instead.
## [15:57:25] WARNING: src/c_api/c_api.cc:935: `ntree_limit` is deprecated, use `iteration_range` instead.
## [15:57:25] WARNING: src/c_api/c_api.cc:935: `ntree_limit` is deprecated, use `iteration_range` instead.
## [15:57:25] WARNING: src/c_api/c_api.cc:935: `ntree_limit` is deprecated, use `iteration_range` instead.
## [15:57:25] WARNING: src/c_api/c_api.cc:935: `ntree_limit` is deprecated, use `iteration_range` instead.
## [15:57:25] WARNING: src/c_api/c_api.cc:935: `ntree_limit` is deprecated, use `iteration_range` instead.
## [15:57:25] WARNING: src/c_api/c_api.cc:935: `ntree_limit` is deprecated, use `iteration_range` instead.
## [15:57:25] WARNING: src/c_api/c_api.cc:935: `ntree_limit` is deprecated, use `iteration_range` instead.
## [15:57:25] WARNING: src/c_api/c_api.cc:935: `ntree_limit` is deprecated, use `iteration_range` instead.
## [15:57:25] WARNING: src/c_api/c_api.cc:935: `ntree_limit` is deprecated, use `iteration_range` instead.
## [15:57:25] WARNING: src/c_api/c_api.cc:935: `ntree_limit` is deprecated, use `iteration_range` instead.
## [15:57:25] WARNING: src/c_api/c_api.cc:935: `ntree_limit` is deprecated, use `iteration_range` instead.
## [15:57:25] WARNING: src/c_api/c_api.cc:935: `ntree_limit` is deprecated, use `iteration_range` instead.
## [15:57:25] WARNING: src/c_api/c_api.cc:935: `ntree_limit` is deprecated, use `iteration_range` instead.
## [15:57:26] WARNING: src/c_api/c_api.cc:935: `ntree_limit` is deprecated, use `iteration_range` instead.
## [15:57:26] WARNING: src/c_api/c_api.cc:935: `ntree_limit` is deprecated, use `iteration_range` instead.
## [15:57:26] WARNING: src/c_api/c_api.cc:935: `ntree_limit` is deprecated, use `iteration_range` instead.
## [15:57:26] WARNING: src/c_api/c_api.cc:935: `ntree_limit` is deprecated, use `iteration_range` instead.
## [15:57:26] WARNING: src/c_api/c_api.cc:935: `ntree_limit` is deprecated, use `iteration_range` instead.
## [15:57:26] WARNING: src/c_api/c_api.cc:935: `ntree_limit` is deprecated, use `iteration_range` instead.
## [15:57:26] WARNING: src/c_api/c_api.cc:935: `ntree_limit` is deprecated, use `iteration_range` instead.
## [15:57:26] WARNING: src/c_api/c_api.cc:935: `ntree_limit` is deprecated, use `iteration_range` instead.
## [15:57:26] WARNING: src/c_api/c_api.cc:935: `ntree_limit` is deprecated, use `iteration_range` instead.
## [15:57:26] WARNING: src/c_api/c_api.cc:935: `ntree_limit` is deprecated, use `iteration_range` instead.
## [15:57:26] WARNING: src/c_api/c_api.cc:935: `ntree_limit` is deprecated, use `iteration_range` instead.
## [15:57:26] WARNING: src/c_api/c_api.cc:935: `ntree_limit` is deprecated, use `iteration_range` instead.
## [15:57:27] WARNING: src/c_api/c_api.cc:935: `ntree_limit` is deprecated, use `iteration_range` instead.
## [15:57:27] WARNING: src/c_api/c_api.cc:935: `ntree_limit` is deprecated, use `iteration_range` instead.
## [15:57:27] WARNING: src/c_api/c_api.cc:935: `ntree_limit` is deprecated, use `iteration_range` instead.
## [15:57:27] WARNING: src/c_api/c_api.cc:935: `ntree_limit` is deprecated, use `iteration_range` instead.
## [15:57:27] WARNING: src/c_api/c_api.cc:935: `ntree_limit` is deprecated, use `iteration_range` instead.
## [15:57:27] WARNING: src/c_api/c_api.cc:935: `ntree_limit` is deprecated, use `iteration_range` instead.
## [15:57:27] WARNING: src/c_api/c_api.cc:935: `ntree_limit` is deprecated, use `iteration_range` instead.
## [15:57:27] WARNING: src/c_api/c_api.cc:935: `ntree_limit` is deprecated, use `iteration_range` instead.
## [15:57:27] WARNING: src/c_api/c_api.cc:935: `ntree_limit` is deprecated, use `iteration_range` instead.
## [15:57:27] WARNING: src/c_api/c_api.cc:935: `ntree_limit` is deprecated, use `iteration_range` instead.
## [15:57:28] WARNING: src/c_api/c_api.cc:935: `ntree_limit` is deprecated, use `iteration_range` instead.
## [15:57:28] WARNING: src/c_api/c_api.cc:935: `ntree_limit` is deprecated, use `iteration_range` instead.
## [15:57:28] WARNING: src/c_api/c_api.cc:935: `ntree_limit` is deprecated, use `iteration_range` instead.
## [15:57:28] WARNING: src/c_api/c_api.cc:935: `ntree_limit` is deprecated, use `iteration_range` instead.
## [15:57:28] WARNING: src/c_api/c_api.cc:935: `ntree_limit` is deprecated, use `iteration_range` instead.
## [15:57:28] WARNING: src/c_api/c_api.cc:935: `ntree_limit` is deprecated, use `iteration_range` instead.
## [15:57:28] WARNING: src/c_api/c_api.cc:935: `ntree_limit` is deprecated, use `iteration_range` instead.
## [15:57:28] WARNING: src/c_api/c_api.cc:935: `ntree_limit` is deprecated, use `iteration_range` instead.
## [15:57:28] WARNING: src/c_api/c_api.cc:935: `ntree_limit` is deprecated, use `iteration_range` instead.
## [15:57:28] WARNING: src/c_api/c_api.cc:935: `ntree_limit` is deprecated, use `iteration_range` instead.
## [15:57:29] WARNING: src/c_api/c_api.cc:935: `ntree_limit` is deprecated, use `iteration_range` instead.
## [15:57:29] WARNING: src/c_api/c_api.cc:935: `ntree_limit` is deprecated, use `iteration_range` instead.
## [15:57:29] WARNING: src/c_api/c_api.cc:935: `ntree_limit` is deprecated, use `iteration_range` instead.
## [15:57:29] WARNING: src/c_api/c_api.cc:935: `ntree_limit` is deprecated, use `iteration_range` instead.
## [15:57:29] WARNING: src/c_api/c_api.cc:935: `ntree_limit` is deprecated, use `iteration_range` instead.
## [15:57:29] WARNING: src/c_api/c_api.cc:935: `ntree_limit` is deprecated, use `iteration_range` instead.
## [15:57:29] WARNING: src/c_api/c_api.cc:935: `ntree_limit` is deprecated, use `iteration_range` instead.
## [15:57:29] WARNING: src/c_api/c_api.cc:935: `ntree_limit` is deprecated, use `iteration_range` instead.
## [15:57:29] WARNING: src/c_api/c_api.cc:935: `ntree_limit` is deprecated, use `iteration_range` instead.
## [15:57:29] WARNING: src/c_api/c_api.cc:935: `ntree_limit` is deprecated, use `iteration_range` instead.
## [15:57:29] WARNING: src/c_api/c_api.cc:935: `ntree_limit` is deprecated, use `iteration_range` instead.
## [15:57:29] WARNING: src/c_api/c_api.cc:935: `ntree_limit` is deprecated, use `iteration_range` instead.
## [15:57:30] WARNING: src/c_api/c_api.cc:935: `ntree_limit` is deprecated, use `iteration_range` instead.
## [15:57:30] WARNING: src/c_api/c_api.cc:935: `ntree_limit` is deprecated, use `iteration_range` instead.
## [15:57:30] WARNING: src/c_api/c_api.cc:935: `ntree_limit` is deprecated, use `iteration_range` instead.
## [15:57:30] WARNING: src/c_api/c_api.cc:935: `ntree_limit` is deprecated, use `iteration_range` instead.
## [15:57:30] WARNING: src/c_api/c_api.cc:935: `ntree_limit` is deprecated, use `iteration_range` instead.
## [15:57:30] WARNING: src/c_api/c_api.cc:935: `ntree_limit` is deprecated, use `iteration_range` instead.
## [15:57:30] WARNING: src/c_api/c_api.cc:935: `ntree_limit` is deprecated, use `iteration_range` instead.
## [15:57:30] WARNING: src/c_api/c_api.cc:935: `ntree_limit` is deprecated, use `iteration_range` instead.
## [15:57:30] WARNING: src/c_api/c_api.cc:935: `ntree_limit` is deprecated, use `iteration_range` instead.
## [15:57:30] WARNING: src/c_api/c_api.cc:935: `ntree_limit` is deprecated, use `iteration_range` instead.
## [15:57:31] WARNING: src/c_api/c_api.cc:935: `ntree_limit` is deprecated, use `iteration_range` instead.
## [15:57:31] WARNING: src/c_api/c_api.cc:935: `ntree_limit` is deprecated, use `iteration_range` instead.
## [15:57:31] WARNING: src/c_api/c_api.cc:935: `ntree_limit` is deprecated, use `iteration_range` instead.
## [15:57:31] WARNING: src/c_api/c_api.cc:935: `ntree_limit` is deprecated, use `iteration_range` instead.
## [15:57:31] WARNING: src/c_api/c_api.cc:935: `ntree_limit` is deprecated, use `iteration_range` instead.
## [15:57:31] WARNING: src/c_api/c_api.cc:935: `ntree_limit` is deprecated, use `iteration_range` instead.
## [15:57:31] WARNING: src/c_api/c_api.cc:935: `ntree_limit` is deprecated, use `iteration_range` instead.
## [15:57:31] WARNING: src/c_api/c_api.cc:935: `ntree_limit` is deprecated, use `iteration_range` instead.
## [15:57:31] WARNING: src/c_api/c_api.cc:935: `ntree_limit` is deprecated, use `iteration_range` instead.
## [15:57:31] WARNING: src/c_api/c_api.cc:935: `ntree_limit` is deprecated, use `iteration_range` instead.
## [15:57:32] WARNING: src/c_api/c_api.cc:935: `ntree_limit` is deprecated, use `iteration_range` instead.
## [15:57:32] WARNING: src/c_api/c_api.cc:935: `ntree_limit` is deprecated, use `iteration_range` instead.
## [15:57:32] WARNING: src/c_api/c_api.cc:935: `ntree_limit` is deprecated, use `iteration_range` instead.
## [15:57:32] WARNING: src/c_api/c_api.cc:935: `ntree_limit` is deprecated, use `iteration_range` instead.
## [15:57:32] WARNING: src/c_api/c_api.cc:935: `ntree_limit` is deprecated, use `iteration_range` instead.
## [15:57:32] WARNING: src/c_api/c_api.cc:935: `ntree_limit` is deprecated, use `iteration_range` instead.
## [15:57:32] WARNING: src/c_api/c_api.cc:935: `ntree_limit` is deprecated, use `iteration_range` instead.
## [15:57:32] WARNING: src/c_api/c_api.cc:935: `ntree_limit` is deprecated, use `iteration_range` instead.
## [15:57:32] WARNING: src/c_api/c_api.cc:935: `ntree_limit` is deprecated, use `iteration_range` instead.
## [15:57:32] WARNING: src/c_api/c_api.cc:935: `ntree_limit` is deprecated, use `iteration_range` instead.
## [15:57:32] WARNING: src/c_api/c_api.cc:935: `ntree_limit` is deprecated, use `iteration_range` instead.
## [15:57:32] WARNING: src/c_api/c_api.cc:935: `ntree_limit` is deprecated, use `iteration_range` instead.
## [15:57:32] WARNING: src/c_api/c_api.cc:935: `ntree_limit` is deprecated, use `iteration_range` instead.
## [15:57:32] WARNING: src/c_api/c_api.cc:935: `ntree_limit` is deprecated, use `iteration_range` instead.
## [15:57:33] WARNING: src/c_api/c_api.cc:935: `ntree_limit` is deprecated, use `iteration_range` instead.
## [15:57:33] WARNING: src/c_api/c_api.cc:935: `ntree_limit` is deprecated, use `iteration_range` instead.
## [15:57:33] WARNING: src/c_api/c_api.cc:935: `ntree_limit` is deprecated, use `iteration_range` instead.
## [15:57:33] WARNING: src/c_api/c_api.cc:935: `ntree_limit` is deprecated, use `iteration_range` instead.
## [15:57:33] WARNING: src/c_api/c_api.cc:935: `ntree_limit` is deprecated, use `iteration_range` instead.
## [15:57:33] WARNING: src/c_api/c_api.cc:935: `ntree_limit` is deprecated, use `iteration_range` instead.
## [15:57:33] WARNING: src/c_api/c_api.cc:935: `ntree_limit` is deprecated, use `iteration_range` instead.
## [15:57:33] WARNING: src/c_api/c_api.cc:935: `ntree_limit` is deprecated, use `iteration_range` instead.
## [15:57:33] WARNING: src/c_api/c_api.cc:935: `ntree_limit` is deprecated, use `iteration_range` instead.
## [15:57:33] WARNING: src/c_api/c_api.cc:935: `ntree_limit` is deprecated, use `iteration_range` instead.
## [15:57:34] WARNING: src/c_api/c_api.cc:935: `ntree_limit` is deprecated, use `iteration_range` instead.
## [15:57:34] WARNING: src/c_api/c_api.cc:935: `ntree_limit` is deprecated, use `iteration_range` instead.
## [15:57:34] WARNING: src/c_api/c_api.cc:935: `ntree_limit` is deprecated, use `iteration_range` instead.
## [15:57:34] WARNING: src/c_api/c_api.cc:935: `ntree_limit` is deprecated, use `iteration_range` instead.
## [15:57:34] WARNING: src/c_api/c_api.cc:935: `ntree_limit` is deprecated, use `iteration_range` instead.
## [15:57:34] WARNING: src/c_api/c_api.cc:935: `ntree_limit` is deprecated, use `iteration_range` instead.
## [15:57:34] WARNING: src/c_api/c_api.cc:935: `ntree_limit` is deprecated, use `iteration_range` instead.
## [15:57:34] WARNING: src/c_api/c_api.cc:935: `ntree_limit` is deprecated, use `iteration_range` instead.
## [15:57:35] WARNING: src/c_api/c_api.cc:935: `ntree_limit` is deprecated, use `iteration_range` instead.
## [15:57:35] WARNING: src/c_api/c_api.cc:935: `ntree_limit` is deprecated, use `iteration_range` instead.
## [15:57:35] WARNING: src/c_api/c_api.cc:935: `ntree_limit` is deprecated, use `iteration_range` instead.
## [15:57:35] WARNING: src/c_api/c_api.cc:935: `ntree_limit` is deprecated, use `iteration_range` instead.
## [15:57:35] WARNING: src/c_api/c_api.cc:935: `ntree_limit` is deprecated, use `iteration_range` instead.
## [15:57:35] WARNING: src/c_api/c_api.cc:935: `ntree_limit` is deprecated, use `iteration_range` instead.
## [15:57:35] WARNING: src/c_api/c_api.cc:935: `ntree_limit` is deprecated, use `iteration_range` instead.
## [15:57:35] WARNING: src/c_api/c_api.cc:935: `ntree_limit` is deprecated, use `iteration_range` instead.
## [15:57:35] WARNING: src/c_api/c_api.cc:935: `ntree_limit` is deprecated, use `iteration_range` instead.
## [15:57:35] WARNING: src/c_api/c_api.cc:935: `ntree_limit` is deprecated, use `iteration_range` instead.
## [15:57:35] WARNING: src/c_api/c_api.cc:935: `ntree_limit` is deprecated, use `iteration_range` instead.
## [15:57:35] WARNING: src/c_api/c_api.cc:935: `ntree_limit` is deprecated, use `iteration_range` instead.
## [15:57:35] WARNING: src/c_api/c_api.cc:935: `ntree_limit` is deprecated, use `iteration_range` instead.
## [15:57:35] WARNING: src/c_api/c_api.cc:935: `ntree_limit` is deprecated, use `iteration_range` instead.
## [15:57:36] WARNING: src/c_api/c_api.cc:935: `ntree_limit` is deprecated, use `iteration_range` instead.
## [15:57:36] WARNING: src/c_api/c_api.cc:935: `ntree_limit` is deprecated, use `iteration_range` instead.
## [15:57:36] WARNING: src/c_api/c_api.cc:935: `ntree_limit` is deprecated, use `iteration_range` instead.
## [15:57:36] WARNING: src/c_api/c_api.cc:935: `ntree_limit` is deprecated, use `iteration_range` instead.
## [15:57:36] WARNING: src/c_api/c_api.cc:935: `ntree_limit` is deprecated, use `iteration_range` instead.
## [15:57:36] WARNING: src/c_api/c_api.cc:935: `ntree_limit` is deprecated, use `iteration_range` instead.
## [15:57:36] WARNING: src/c_api/c_api.cc:935: `ntree_limit` is deprecated, use `iteration_range` instead.
## [15:57:36] WARNING: src/c_api/c_api.cc:935: `ntree_limit` is deprecated, use `iteration_range` instead.
## [15:57:36] WARNING: src/c_api/c_api.cc:935: `ntree_limit` is deprecated, use `iteration_range` instead.
## [15:57:36] WARNING: src/c_api/c_api.cc:935: `ntree_limit` is deprecated, use `iteration_range` instead.
## [15:57:36] WARNING: src/c_api/c_api.cc:935: `ntree_limit` is deprecated, use `iteration_range` instead.
## [15:57:36] WARNING: src/c_api/c_api.cc:935: `ntree_limit` is deprecated, use `iteration_range` instead.
## [15:57:37] WARNING: src/c_api/c_api.cc:935: `ntree_limit` is deprecated, use `iteration_range` instead.
## [15:57:37] WARNING: src/c_api/c_api.cc:935: `ntree_limit` is deprecated, use `iteration_range` instead.
## [15:57:37] WARNING: src/c_api/c_api.cc:935: `ntree_limit` is deprecated, use `iteration_range` instead.
## [15:57:37] WARNING: src/c_api/c_api.cc:935: `ntree_limit` is deprecated, use `iteration_range` instead.
## [15:57:37] WARNING: src/c_api/c_api.cc:935: `ntree_limit` is deprecated, use `iteration_range` instead.
## [15:57:37] WARNING: src/c_api/c_api.cc:935: `ntree_limit` is deprecated, use `iteration_range` instead.
## [15:57:37] WARNING: src/c_api/c_api.cc:935: `ntree_limit` is deprecated, use `iteration_range` instead.
## [15:57:37] WARNING: src/c_api/c_api.cc:935: `ntree_limit` is deprecated, use `iteration_range` instead.
## [15:57:38] WARNING: src/c_api/c_api.cc:935: `ntree_limit` is deprecated, use `iteration_range` instead.
## [15:57:38] WARNING: src/c_api/c_api.cc:935: `ntree_limit` is deprecated, use `iteration_range` instead.
## [15:57:38] WARNING: src/c_api/c_api.cc:935: `ntree_limit` is deprecated, use `iteration_range` instead.
## [15:57:38] WARNING: src/c_api/c_api.cc:935: `ntree_limit` is deprecated, use `iteration_range` instead.
## [15:57:38] WARNING: src/c_api/c_api.cc:935: `ntree_limit` is deprecated, use `iteration_range` instead.
## [15:57:38] WARNING: src/c_api/c_api.cc:935: `ntree_limit` is deprecated, use `iteration_range` instead.
## [15:57:38] WARNING: src/c_api/c_api.cc:935: `ntree_limit` is deprecated, use `iteration_range` instead.
## [15:57:38] WARNING: src/c_api/c_api.cc:935: `ntree_limit` is deprecated, use `iteration_range` instead.
## [15:57:38] WARNING: src/c_api/c_api.cc:935: `ntree_limit` is deprecated, use `iteration_range` instead.
## [15:57:38] WARNING: src/c_api/c_api.cc:935: `ntree_limit` is deprecated, use `iteration_range` instead.
## [15:57:38] WARNING: src/c_api/c_api.cc:935: `ntree_limit` is deprecated, use `iteration_range` instead.
## [15:57:38] WARNING: src/c_api/c_api.cc:935: `ntree_limit` is deprecated, use `iteration_range` instead.
## [15:57:38] WARNING: src/c_api/c_api.cc:935: `ntree_limit` is deprecated, use `iteration_range` instead.
## [15:57:38] WARNING: src/c_api/c_api.cc:935: `ntree_limit` is deprecated, use `iteration_range` instead.
## [15:57:39] WARNING: src/c_api/c_api.cc:935: `ntree_limit` is deprecated, use `iteration_range` instead.
## [15:57:39] WARNING: src/c_api/c_api.cc:935: `ntree_limit` is deprecated, use `iteration_range` instead.
## [15:57:39] WARNING: src/c_api/c_api.cc:935: `ntree_limit` is deprecated, use `iteration_range` instead.
## [15:57:39] WARNING: src/c_api/c_api.cc:935: `ntree_limit` is deprecated, use `iteration_range` instead.
## [15:57:39] WARNING: src/c_api/c_api.cc:935: `ntree_limit` is deprecated, use `iteration_range` instead.
## [15:57:39] WARNING: src/c_api/c_api.cc:935: `ntree_limit` is deprecated, use `iteration_range` instead.
## [15:57:39] WARNING: src/c_api/c_api.cc:935: `ntree_limit` is deprecated, use `iteration_range` instead.
## [15:57:39] WARNING: src/c_api/c_api.cc:935: `ntree_limit` is deprecated, use `iteration_range` instead.
## [15:57:39] WARNING: src/c_api/c_api.cc:935: `ntree_limit` is deprecated, use `iteration_range` instead.
## [15:57:39] WARNING: src/c_api/c_api.cc:935: `ntree_limit` is deprecated, use `iteration_range` instead.
## [15:57:40] WARNING: src/c_api/c_api.cc:935: `ntree_limit` is deprecated, use `iteration_range` instead.
## [15:57:40] WARNING: src/c_api/c_api.cc:935: `ntree_limit` is deprecated, use `iteration_range` instead.
## [15:57:40] WARNING: src/c_api/c_api.cc:935: `ntree_limit` is deprecated, use `iteration_range` instead.
## [15:57:40] WARNING: src/c_api/c_api.cc:935: `ntree_limit` is deprecated, use `iteration_range` instead.
## [15:57:40] WARNING: src/c_api/c_api.cc:935: `ntree_limit` is deprecated, use `iteration_range` instead.
## [15:57:40] WARNING: src/c_api/c_api.cc:935: `ntree_limit` is deprecated, use `iteration_range` instead.
## [15:57:40] WARNING: src/c_api/c_api.cc:935: `ntree_limit` is deprecated, use `iteration_range` instead.
## [15:57:40] WARNING: src/c_api/c_api.cc:935: `ntree_limit` is deprecated, use `iteration_range` instead.
## [15:57:40] WARNING: src/c_api/c_api.cc:935: `ntree_limit` is deprecated, use `iteration_range` instead.
## [15:57:40] WARNING: src/c_api/c_api.cc:935: `ntree_limit` is deprecated, use `iteration_range` instead.
## [15:57:41] WARNING: src/c_api/c_api.cc:935: `ntree_limit` is deprecated, use `iteration_range` instead.
## [15:57:41] WARNING: src/c_api/c_api.cc:935: `ntree_limit` is deprecated, use `iteration_range` instead.
## [15:57:41] WARNING: src/c_api/c_api.cc:935: `ntree_limit` is deprecated, use `iteration_range` instead.
## [15:57:41] WARNING: src/c_api/c_api.cc:935: `ntree_limit` is deprecated, use `iteration_range` instead.
## [15:57:41] WARNING: src/c_api/c_api.cc:935: `ntree_limit` is deprecated, use `iteration_range` instead.
## [15:57:41] WARNING: src/c_api/c_api.cc:935: `ntree_limit` is deprecated, use `iteration_range` instead.
## [15:57:41] WARNING: src/c_api/c_api.cc:935: `ntree_limit` is deprecated, use `iteration_range` instead.
## [15:57:41] WARNING: src/c_api/c_api.cc:935: `ntree_limit` is deprecated, use `iteration_range` instead.
## [15:57:41] WARNING: src/c_api/c_api.cc:935: `ntree_limit` is deprecated, use `iteration_range` instead.
## [15:57:41] WARNING: src/c_api/c_api.cc:935: `ntree_limit` is deprecated, use `iteration_range` instead.
## [15:57:42] WARNING: src/c_api/c_api.cc:935: `ntree_limit` is deprecated, use `iteration_range` instead.
## [15:57:42] WARNING: src/c_api/c_api.cc:935: `ntree_limit` is deprecated, use `iteration_range` instead.
## [15:57:42] WARNING: src/c_api/c_api.cc:935: `ntree_limit` is deprecated, use `iteration_range` instead.
## [15:57:42] WARNING: src/c_api/c_api.cc:935: `ntree_limit` is deprecated, use `iteration_range` instead.
## [15:57:42] WARNING: src/c_api/c_api.cc:935: `ntree_limit` is deprecated, use `iteration_range` instead.
## [15:57:42] WARNING: src/c_api/c_api.cc:935: `ntree_limit` is deprecated, use `iteration_range` instead.
## [15:57:42] WARNING: src/c_api/c_api.cc:935: `ntree_limit` is deprecated, use `iteration_range` instead.
## [15:57:42] WARNING: src/c_api/c_api.cc:935: `ntree_limit` is deprecated, use `iteration_range` instead.
## [15:57:42] WARNING: src/c_api/c_api.cc:935: `ntree_limit` is deprecated, use `iteration_range` instead.
## [15:57:42] WARNING: src/c_api/c_api.cc:935: `ntree_limit` is deprecated, use `iteration_range` instead.
## [15:57:42] WARNING: src/c_api/c_api.cc:935: `ntree_limit` is deprecated, use `iteration_range` instead.
## [15:57:42] WARNING: src/c_api/c_api.cc:935: `ntree_limit` is deprecated, use `iteration_range` instead.
## [15:57:42] WARNING: src/c_api/c_api.cc:935: `ntree_limit` is deprecated, use `iteration_range` instead.
## [15:57:42] WARNING: src/c_api/c_api.cc:935: `ntree_limit` is deprecated, use `iteration_range` instead.
## [15:57:43] WARNING: src/c_api/c_api.cc:935: `ntree_limit` is deprecated, use `iteration_range` instead.
## [15:57:43] WARNING: src/c_api/c_api.cc:935: `ntree_limit` is deprecated, use `iteration_range` instead.
## [15:57:43] WARNING: src/c_api/c_api.cc:935: `ntree_limit` is deprecated, use `iteration_range` instead.
## [15:57:43] WARNING: src/c_api/c_api.cc:935: `ntree_limit` is deprecated, use `iteration_range` instead.
## [15:57:43] WARNING: src/c_api/c_api.cc:935: `ntree_limit` is deprecated, use `iteration_range` instead.
## [15:57:43] WARNING: src/c_api/c_api.cc:935: `ntree_limit` is deprecated, use `iteration_range` instead.
## [15:57:43] WARNING: src/c_api/c_api.cc:935: `ntree_limit` is deprecated, use `iteration_range` instead.
## [15:57:43] WARNING: src/c_api/c_api.cc:935: `ntree_limit` is deprecated, use `iteration_range` instead.
## [15:57:43] WARNING: src/c_api/c_api.cc:935: `ntree_limit` is deprecated, use `iteration_range` instead.
## [15:57:43] WARNING: src/c_api/c_api.cc:935: `ntree_limit` is deprecated, use `iteration_range` instead.
## [15:57:44] WARNING: src/c_api/c_api.cc:935: `ntree_limit` is deprecated, use `iteration_range` instead.
## [15:57:44] WARNING: src/c_api/c_api.cc:935: `ntree_limit` is deprecated, use `iteration_range` instead.
## [15:57:44] WARNING: src/c_api/c_api.cc:935: `ntree_limit` is deprecated, use `iteration_range` instead.
## [15:57:44] WARNING: src/c_api/c_api.cc:935: `ntree_limit` is deprecated, use `iteration_range` instead.
## [15:57:44] WARNING: src/c_api/c_api.cc:935: `ntree_limit` is deprecated, use `iteration_range` instead.
## [15:57:44] WARNING: src/c_api/c_api.cc:935: `ntree_limit` is deprecated, use `iteration_range` instead.
## [15:57:44] WARNING: src/c_api/c_api.cc:935: `ntree_limit` is deprecated, use `iteration_range` instead.
## [15:57:44] WARNING: src/c_api/c_api.cc:935: `ntree_limit` is deprecated, use `iteration_range` instead.
## [15:57:44] WARNING: src/c_api/c_api.cc:935: `ntree_limit` is deprecated, use `iteration_range` instead.
## [15:57:44] WARNING: src/c_api/c_api.cc:935: `ntree_limit` is deprecated, use `iteration_range` instead.
## [15:57:47] WARNING: src/c_api/c_api.cc:935: `ntree_limit` is deprecated, use `iteration_range` instead.
## [15:57:47] WARNING: src/c_api/c_api.cc:935: `ntree_limit` is deprecated, use `iteration_range` instead.
## [15:57:47] WARNING: src/c_api/c_api.cc:935: `ntree_limit` is deprecated, use `iteration_range` instead.
## [15:57:47] WARNING: src/c_api/c_api.cc:935: `ntree_limit` is deprecated, use `iteration_range` instead.
## [15:57:48] WARNING: src/c_api/c_api.cc:935: `ntree_limit` is deprecated, use `iteration_range` instead.
## [15:57:48] WARNING: src/c_api/c_api.cc:935: `ntree_limit` is deprecated, use `iteration_range` instead.
## [15:57:48] WARNING: src/c_api/c_api.cc:935: `ntree_limit` is deprecated, use `iteration_range` instead.
## [15:57:48] WARNING: src/c_api/c_api.cc:935: `ntree_limit` is deprecated, use `iteration_range` instead.
## [15:57:48] WARNING: src/c_api/c_api.cc:935: `ntree_limit` is deprecated, use `iteration_range` instead.
## [15:57:48] WARNING: src/c_api/c_api.cc:935: `ntree_limit` is deprecated, use `iteration_range` instead.
## [15:57:48] WARNING: src/c_api/c_api.cc:935: `ntree_limit` is deprecated, use `iteration_range` instead.
## [15:57:48] WARNING: src/c_api/c_api.cc:935: `ntree_limit` is deprecated, use `iteration_range` instead.
## [15:57:48] WARNING: src/c_api/c_api.cc:935: `ntree_limit` is deprecated, use `iteration_range` instead.
## [15:57:48] WARNING: src/c_api/c_api.cc:935: `ntree_limit` is deprecated, use `iteration_range` instead.
## [15:57:48] WARNING: src/c_api/c_api.cc:935: `ntree_limit` is deprecated, use `iteration_range` instead.
## [15:57:48] WARNING: src/c_api/c_api.cc:935: `ntree_limit` is deprecated, use `iteration_range` instead.
## [15:57:48] WARNING: src/c_api/c_api.cc:935: `ntree_limit` is deprecated, use `iteration_range` instead.
## [15:57:48] WARNING: src/c_api/c_api.cc:935: `ntree_limit` is deprecated, use `iteration_range` instead.
## [15:57:49] WARNING: src/c_api/c_api.cc:935: `ntree_limit` is deprecated, use `iteration_range` instead.
## [15:57:49] WARNING: src/c_api/c_api.cc:935: `ntree_limit` is deprecated, use `iteration_range` instead.
## [15:57:49] WARNING: src/c_api/c_api.cc:935: `ntree_limit` is deprecated, use `iteration_range` instead.
## [15:57:49] WARNING: src/c_api/c_api.cc:935: `ntree_limit` is deprecated, use `iteration_range` instead.
## [15:57:49] WARNING: src/c_api/c_api.cc:935: `ntree_limit` is deprecated, use `iteration_range` instead.
## [15:57:49] WARNING: src/c_api/c_api.cc:935: `ntree_limit` is deprecated, use `iteration_range` instead.
## [15:57:49] WARNING: src/c_api/c_api.cc:935: `ntree_limit` is deprecated, use `iteration_range` instead.
## [15:57:49] WARNING: src/c_api/c_api.cc:935: `ntree_limit` is deprecated, use `iteration_range` instead.
## [15:57:50] WARNING: src/c_api/c_api.cc:935: `ntree_limit` is deprecated, use `iteration_range` instead.
## [15:57:50] WARNING: src/c_api/c_api.cc:935: `ntree_limit` is deprecated, use `iteration_range` instead.
## [15:57:50] WARNING: src/c_api/c_api.cc:935: `ntree_limit` is deprecated, use `iteration_range` instead.
## [15:57:50] WARNING: src/c_api/c_api.cc:935: `ntree_limit` is deprecated, use `iteration_range` instead.
## [15:57:50] WARNING: src/c_api/c_api.cc:935: `ntree_limit` is deprecated, use `iteration_range` instead.
## [15:57:50] WARNING: src/c_api/c_api.cc:935: `ntree_limit` is deprecated, use `iteration_range` instead.
## [15:57:50] WARNING: src/c_api/c_api.cc:935: `ntree_limit` is deprecated, use `iteration_range` instead.
## [15:57:50] WARNING: src/c_api/c_api.cc:935: `ntree_limit` is deprecated, use `iteration_range` instead.
## [15:57:50] WARNING: src/c_api/c_api.cc:935: `ntree_limit` is deprecated, use `iteration_range` instead.
## [15:57:50] WARNING: src/c_api/c_api.cc:935: `ntree_limit` is deprecated, use `iteration_range` instead.
## [15:57:51] WARNING: src/c_api/c_api.cc:935: `ntree_limit` is deprecated, use `iteration_range` instead.
## [15:57:51] WARNING: src/c_api/c_api.cc:935: `ntree_limit` is deprecated, use `iteration_range` instead.
## [15:57:51] WARNING: src/c_api/c_api.cc:935: `ntree_limit` is deprecated, use `iteration_range` instead.
## [15:57:51] WARNING: src/c_api/c_api.cc:935: `ntree_limit` is deprecated, use `iteration_range` instead.
## [15:57:51] WARNING: src/c_api/c_api.cc:935: `ntree_limit` is deprecated, use `iteration_range` instead.
## [15:57:51] WARNING: src/c_api/c_api.cc:935: `ntree_limit` is deprecated, use `iteration_range` instead.
## [15:57:51] WARNING: src/c_api/c_api.cc:935: `ntree_limit` is deprecated, use `iteration_range` instead.
## [15:57:51] WARNING: src/c_api/c_api.cc:935: `ntree_limit` is deprecated, use `iteration_range` instead.
## [15:57:51] WARNING: src/c_api/c_api.cc:935: `ntree_limit` is deprecated, use `iteration_range` instead.
## [15:57:51] WARNING: src/c_api/c_api.cc:935: `ntree_limit` is deprecated, use `iteration_range` instead.
## [15:57:51] WARNING: src/c_api/c_api.cc:935: `ntree_limit` is deprecated, use `iteration_range` instead.
## [15:57:51] WARNING: src/c_api/c_api.cc:935: `ntree_limit` is deprecated, use `iteration_range` instead.
## [15:57:51] WARNING: src/c_api/c_api.cc:935: `ntree_limit` is deprecated, use `iteration_range` instead.
## [15:57:51] WARNING: src/c_api/c_api.cc:935: `ntree_limit` is deprecated, use `iteration_range` instead.
## [15:57:52] WARNING: src/c_api/c_api.cc:935: `ntree_limit` is deprecated, use `iteration_range` instead.
## [15:57:52] WARNING: src/c_api/c_api.cc:935: `ntree_limit` is deprecated, use `iteration_range` instead.
## [15:57:52] WARNING: src/c_api/c_api.cc:935: `ntree_limit` is deprecated, use `iteration_range` instead.
## [15:57:52] WARNING: src/c_api/c_api.cc:935: `ntree_limit` is deprecated, use `iteration_range` instead.
## [15:57:52] WARNING: src/c_api/c_api.cc:935: `ntree_limit` is deprecated, use `iteration_range` instead.
## [15:57:52] WARNING: src/c_api/c_api.cc:935: `ntree_limit` is deprecated, use `iteration_range` instead.
## [15:57:52] WARNING: src/c_api/c_api.cc:935: `ntree_limit` is deprecated, use `iteration_range` instead.
## [15:57:52] WARNING: src/c_api/c_api.cc:935: `ntree_limit` is deprecated, use `iteration_range` instead.
## [15:57:52] WARNING: src/c_api/c_api.cc:935: `ntree_limit` is deprecated, use `iteration_range` instead.
## [15:57:52] WARNING: src/c_api/c_api.cc:935: `ntree_limit` is deprecated, use `iteration_range` instead.
## [15:57:52] WARNING: src/c_api/c_api.cc:935: `ntree_limit` is deprecated, use `iteration_range` instead.
## [15:57:52] WARNING: src/c_api/c_api.cc:935: `ntree_limit` is deprecated, use `iteration_range` instead.
## [15:57:53] WARNING: src/c_api/c_api.cc:935: `ntree_limit` is deprecated, use `iteration_range` instead.
## [15:57:53] WARNING: src/c_api/c_api.cc:935: `ntree_limit` is deprecated, use `iteration_range` instead.
## [15:57:53] WARNING: src/c_api/c_api.cc:935: `ntree_limit` is deprecated, use `iteration_range` instead.
## [15:57:53] WARNING: src/c_api/c_api.cc:935: `ntree_limit` is deprecated, use `iteration_range` instead.
## [15:57:53] WARNING: src/c_api/c_api.cc:935: `ntree_limit` is deprecated, use `iteration_range` instead.
## [15:57:53] WARNING: src/c_api/c_api.cc:935: `ntree_limit` is deprecated, use `iteration_range` instead.
## [15:57:53] WARNING: src/c_api/c_api.cc:935: `ntree_limit` is deprecated, use `iteration_range` instead.
## [15:57:53] WARNING: src/c_api/c_api.cc:935: `ntree_limit` is deprecated, use `iteration_range` instead.
## [15:57:53] WARNING: src/c_api/c_api.cc:935: `ntree_limit` is deprecated, use `iteration_range` instead.
## [15:57:53] WARNING: src/c_api/c_api.cc:935: `ntree_limit` is deprecated, use `iteration_range` instead.
## [15:57:54] WARNING: src/c_api/c_api.cc:935: `ntree_limit` is deprecated, use `iteration_range` instead.
## [15:57:54] WARNING: src/c_api/c_api.cc:935: `ntree_limit` is deprecated, use `iteration_range` instead.
## [15:57:54] WARNING: src/c_api/c_api.cc:935: `ntree_limit` is deprecated, use `iteration_range` instead.
## [15:57:54] WARNING: src/c_api/c_api.cc:935: `ntree_limit` is deprecated, use `iteration_range` instead.
## [15:57:54] WARNING: src/c_api/c_api.cc:935: `ntree_limit` is deprecated, use `iteration_range` instead.
## [15:57:54] WARNING: src/c_api/c_api.cc:935: `ntree_limit` is deprecated, use `iteration_range` instead.
## [15:57:54] WARNING: src/c_api/c_api.cc:935: `ntree_limit` is deprecated, use `iteration_range` instead.
## [15:57:54] WARNING: src/c_api/c_api.cc:935: `ntree_limit` is deprecated, use `iteration_range` instead.
## [15:57:54] WARNING: src/c_api/c_api.cc:935: `ntree_limit` is deprecated, use `iteration_range` instead.
## [15:57:54] WARNING: src/c_api/c_api.cc:935: `ntree_limit` is deprecated, use `iteration_range` instead.
## [15:57:54] WARNING: src/c_api/c_api.cc:935: `ntree_limit` is deprecated, use `iteration_range` instead.
## [15:57:54] WARNING: src/c_api/c_api.cc:935: `ntree_limit` is deprecated, use `iteration_range` instead.
## [15:57:54] WARNING: src/c_api/c_api.cc:935: `ntree_limit` is deprecated, use `iteration_range` instead.
## [15:57:54] WARNING: src/c_api/c_api.cc:935: `ntree_limit` is deprecated, use `iteration_range` instead.
## [15:57:55] WARNING: src/c_api/c_api.cc:935: `ntree_limit` is deprecated, use `iteration_range` instead.
## [15:57:55] WARNING: src/c_api/c_api.cc:935: `ntree_limit` is deprecated, use `iteration_range` instead.
## [15:57:55] WARNING: src/c_api/c_api.cc:935: `ntree_limit` is deprecated, use `iteration_range` instead.
## [15:57:55] WARNING: src/c_api/c_api.cc:935: `ntree_limit` is deprecated, use `iteration_range` instead.
## [15:57:55] WARNING: src/c_api/c_api.cc:935: `ntree_limit` is deprecated, use `iteration_range` instead.
## [15:57:55] WARNING: src/c_api/c_api.cc:935: `ntree_limit` is deprecated, use `iteration_range` instead.
## [15:57:55] WARNING: src/c_api/c_api.cc:935: `ntree_limit` is deprecated, use `iteration_range` instead.
## [15:57:55] WARNING: src/c_api/c_api.cc:935: `ntree_limit` is deprecated, use `iteration_range` instead.
## [15:57:55] WARNING: src/c_api/c_api.cc:935: `ntree_limit` is deprecated, use `iteration_range` instead.
## [15:57:55] WARNING: src/c_api/c_api.cc:935: `ntree_limit` is deprecated, use `iteration_range` instead.
## [15:57:56] WARNING: src/c_api/c_api.cc:935: `ntree_limit` is deprecated, use `iteration_range` instead.
## [15:57:56] WARNING: src/c_api/c_api.cc:935: `ntree_limit` is deprecated, use `iteration_range` instead.
## [15:57:56] WARNING: src/c_api/c_api.cc:935: `ntree_limit` is deprecated, use `iteration_range` instead.
## [15:57:56] WARNING: src/c_api/c_api.cc:935: `ntree_limit` is deprecated, use `iteration_range` instead.
## [15:57:56] WARNING: src/c_api/c_api.cc:935: `ntree_limit` is deprecated, use `iteration_range` instead.
## [15:57:56] WARNING: src/c_api/c_api.cc:935: `ntree_limit` is deprecated, use `iteration_range` instead.
## [15:57:56] WARNING: src/c_api/c_api.cc:935: `ntree_limit` is deprecated, use `iteration_range` instead.
## [15:57:56] WARNING: src/c_api/c_api.cc:935: `ntree_limit` is deprecated, use `iteration_range` instead.
## [15:57:56] WARNING: src/c_api/c_api.cc:935: `ntree_limit` is deprecated, use `iteration_range` instead.
## [15:57:56] WARNING: src/c_api/c_api.cc:935: `ntree_limit` is deprecated, use `iteration_range` instead.
## [15:57:57] WARNING: src/c_api/c_api.cc:935: `ntree_limit` is deprecated, use `iteration_range` instead.
## [15:57:57] WARNING: src/c_api/c_api.cc:935: `ntree_limit` is deprecated, use `iteration_range` instead.
## [15:57:57] WARNING: src/c_api/c_api.cc:935: `ntree_limit` is deprecated, use `iteration_range` instead.
## [15:57:57] WARNING: src/c_api/c_api.cc:935: `ntree_limit` is deprecated, use `iteration_range` instead.
## [15:57:57] WARNING: src/c_api/c_api.cc:935: `ntree_limit` is deprecated, use `iteration_range` instead.
## [15:57:57] WARNING: src/c_api/c_api.cc:935: `ntree_limit` is deprecated, use `iteration_range` instead.
## [15:57:57] WARNING: src/c_api/c_api.cc:935: `ntree_limit` is deprecated, use `iteration_range` instead.
## [15:57:57] WARNING: src/c_api/c_api.cc:935: `ntree_limit` is deprecated, use `iteration_range` instead.
## [15:57:57] WARNING: src/c_api/c_api.cc:935: `ntree_limit` is deprecated, use `iteration_range` instead.
## [15:57:57] WARNING: src/c_api/c_api.cc:935: `ntree_limit` is deprecated, use `iteration_range` instead.
## [15:57:57] WARNING: src/c_api/c_api.cc:935: `ntree_limit` is deprecated, use `iteration_range` instead.
## [15:57:57] WARNING: src/c_api/c_api.cc:935: `ntree_limit` is deprecated, use `iteration_range` instead.
## [15:57:57] WARNING: src/c_api/c_api.cc:935: `ntree_limit` is deprecated, use `iteration_range` instead.
## [15:57:57] WARNING: src/c_api/c_api.cc:935: `ntree_limit` is deprecated, use `iteration_range` instead.
## [15:57:58] WARNING: src/c_api/c_api.cc:935: `ntree_limit` is deprecated, use `iteration_range` instead.
## [15:57:58] WARNING: src/c_api/c_api.cc:935: `ntree_limit` is deprecated, use `iteration_range` instead.
## [15:57:58] WARNING: src/c_api/c_api.cc:935: `ntree_limit` is deprecated, use `iteration_range` instead.
## [15:57:58] WARNING: src/c_api/c_api.cc:935: `ntree_limit` is deprecated, use `iteration_range` instead.
## [15:57:58] WARNING: src/c_api/c_api.cc:935: `ntree_limit` is deprecated, use `iteration_range` instead.
## [15:57:58] WARNING: src/c_api/c_api.cc:935: `ntree_limit` is deprecated, use `iteration_range` instead.
## [15:57:58] WARNING: src/c_api/c_api.cc:935: `ntree_limit` is deprecated, use `iteration_range` instead.
## [15:57:58] WARNING: src/c_api/c_api.cc:935: `ntree_limit` is deprecated, use `iteration_range` instead.
## [15:57:58] WARNING: src/c_api/c_api.cc:935: `ntree_limit` is deprecated, use `iteration_range` instead.
## [15:57:58] WARNING: src/c_api/c_api.cc:935: `ntree_limit` is deprecated, use `iteration_range` instead.
## [15:57:58] WARNING: src/c_api/c_api.cc:935: `ntree_limit` is deprecated, use `iteration_range` instead.
## [15:57:58] WARNING: src/c_api/c_api.cc:935: `ntree_limit` is deprecated, use `iteration_range` instead.
## [15:57:59] WARNING: src/c_api/c_api.cc:935: `ntree_limit` is deprecated, use `iteration_range` instead.
## [15:57:59] WARNING: src/c_api/c_api.cc:935: `ntree_limit` is deprecated, use `iteration_range` instead.
## [15:57:59] WARNING: src/c_api/c_api.cc:935: `ntree_limit` is deprecated, use `iteration_range` instead.
## [15:57:59] WARNING: src/c_api/c_api.cc:935: `ntree_limit` is deprecated, use `iteration_range` instead.
## [15:57:59] WARNING: src/c_api/c_api.cc:935: `ntree_limit` is deprecated, use `iteration_range` instead.
## [15:57:59] WARNING: src/c_api/c_api.cc:935: `ntree_limit` is deprecated, use `iteration_range` instead.
## [15:57:59] WARNING: src/c_api/c_api.cc:935: `ntree_limit` is deprecated, use `iteration_range` instead.
## [15:57:59] WARNING: src/c_api/c_api.cc:935: `ntree_limit` is deprecated, use `iteration_range` instead.
## [15:57:59] WARNING: src/c_api/c_api.cc:935: `ntree_limit` is deprecated, use `iteration_range` instead.
## [15:57:59] WARNING: src/c_api/c_api.cc:935: `ntree_limit` is deprecated, use `iteration_range` instead.
## [15:58:00] WARNING: src/c_api/c_api.cc:935: `ntree_limit` is deprecated, use `iteration_range` instead.
## [15:58:00] WARNING: src/c_api/c_api.cc:935: `ntree_limit` is deprecated, use `iteration_range` instead.
## [15:58:00] WARNING: src/c_api/c_api.cc:935: `ntree_limit` is deprecated, use `iteration_range` instead.
## [15:58:00] WARNING: src/c_api/c_api.cc:935: `ntree_limit` is deprecated, use `iteration_range` instead.
## [15:58:00] WARNING: src/c_api/c_api.cc:935: `ntree_limit` is deprecated, use `iteration_range` instead.
## [15:58:00] WARNING: src/c_api/c_api.cc:935: `ntree_limit` is deprecated, use `iteration_range` instead.
## [15:58:00] WARNING: src/c_api/c_api.cc:935: `ntree_limit` is deprecated, use `iteration_range` instead.
## [15:58:00] WARNING: src/c_api/c_api.cc:935: `ntree_limit` is deprecated, use `iteration_range` instead.
## [15:58:00] WARNING: src/c_api/c_api.cc:935: `ntree_limit` is deprecated, use `iteration_range` instead.
## [15:58:00] WARNING: src/c_api/c_api.cc:935: `ntree_limit` is deprecated, use `iteration_range` instead.
## [15:58:00] WARNING: src/c_api/c_api.cc:935: `ntree_limit` is deprecated, use `iteration_range` instead.
## [15:58:00] WARNING: src/c_api/c_api.cc:935: `ntree_limit` is deprecated, use `iteration_range` instead.
## [15:58:00] WARNING: src/c_api/c_api.cc:935: `ntree_limit` is deprecated, use `iteration_range` instead.
## [15:58:00] WARNING: src/c_api/c_api.cc:935: `ntree_limit` is deprecated, use `iteration_range` instead.
## [15:58:01] WARNING: src/c_api/c_api.cc:935: `ntree_limit` is deprecated, use `iteration_range` instead.
## [15:58:01] WARNING: src/c_api/c_api.cc:935: `ntree_limit` is deprecated, use `iteration_range` instead.
## [15:58:01] WARNING: src/c_api/c_api.cc:935: `ntree_limit` is deprecated, use `iteration_range` instead.
## [15:58:01] WARNING: src/c_api/c_api.cc:935: `ntree_limit` is deprecated, use `iteration_range` instead.
## [15:58:01] WARNING: src/c_api/c_api.cc:935: `ntree_limit` is deprecated, use `iteration_range` instead.
## [15:58:01] WARNING: src/c_api/c_api.cc:935: `ntree_limit` is deprecated, use `iteration_range` instead.
## [15:58:01] WARNING: src/c_api/c_api.cc:935: `ntree_limit` is deprecated, use `iteration_range` instead.
## [15:58:01] WARNING: src/c_api/c_api.cc:935: `ntree_limit` is deprecated, use `iteration_range` instead.
## [15:58:01] WARNING: src/c_api/c_api.cc:935: `ntree_limit` is deprecated, use `iteration_range` instead.
## [15:58:01] WARNING: src/c_api/c_api.cc:935: `ntree_limit` is deprecated, use `iteration_range` instead.
## [15:58:01] WARNING: src/c_api/c_api.cc:935: `ntree_limit` is deprecated, use `iteration_range` instead.
## [15:58:01] WARNING: src/c_api/c_api.cc:935: `ntree_limit` is deprecated, use `iteration_range` instead.
## [15:58:02] WARNING: src/c_api/c_api.cc:935: `ntree_limit` is deprecated, use `iteration_range` instead.
## [15:58:02] WARNING: src/c_api/c_api.cc:935: `ntree_limit` is deprecated, use `iteration_range` instead.
## [15:58:02] WARNING: src/c_api/c_api.cc:935: `ntree_limit` is deprecated, use `iteration_range` instead.
## [15:58:02] WARNING: src/c_api/c_api.cc:935: `ntree_limit` is deprecated, use `iteration_range` instead.
## [15:58:02] WARNING: src/c_api/c_api.cc:935: `ntree_limit` is deprecated, use `iteration_range` instead.
## [15:58:02] WARNING: src/c_api/c_api.cc:935: `ntree_limit` is deprecated, use `iteration_range` instead.
## [15:58:02] WARNING: src/c_api/c_api.cc:935: `ntree_limit` is deprecated, use `iteration_range` instead.
## [15:58:02] WARNING: src/c_api/c_api.cc:935: `ntree_limit` is deprecated, use `iteration_range` instead.
## [15:58:03] WARNING: src/c_api/c_api.cc:935: `ntree_limit` is deprecated, use `iteration_range` instead.
## [15:58:03] WARNING: src/c_api/c_api.cc:935: `ntree_limit` is deprecated, use `iteration_range` instead.
## [15:58:03] WARNING: src/c_api/c_api.cc:935: `ntree_limit` is deprecated, use `iteration_range` instead.
## [15:58:03] WARNING: src/c_api/c_api.cc:935: `ntree_limit` is deprecated, use `iteration_range` instead.
## [15:58:03] WARNING: src/c_api/c_api.cc:935: `ntree_limit` is deprecated, use `iteration_range` instead.
## [15:58:03] WARNING: src/c_api/c_api.cc:935: `ntree_limit` is deprecated, use `iteration_range` instead.
## [15:58:03] WARNING: src/c_api/c_api.cc:935: `ntree_limit` is deprecated, use `iteration_range` instead.
## [15:58:03] WARNING: src/c_api/c_api.cc:935: `ntree_limit` is deprecated, use `iteration_range` instead.
## [15:58:03] WARNING: src/c_api/c_api.cc:935: `ntree_limit` is deprecated, use `iteration_range` instead.
## [15:58:03] WARNING: src/c_api/c_api.cc:935: `ntree_limit` is deprecated, use `iteration_range` instead.
## [15:58:03] WARNING: src/c_api/c_api.cc:935: `ntree_limit` is deprecated, use `iteration_range` instead.
## [15:58:03] WARNING: src/c_api/c_api.cc:935: `ntree_limit` is deprecated, use `iteration_range` instead.
## [15:58:03] WARNING: src/c_api/c_api.cc:935: `ntree_limit` is deprecated, use `iteration_range` instead.
## [15:58:03] WARNING: src/c_api/c_api.cc:935: `ntree_limit` is deprecated, use `iteration_range` instead.
## [15:58:04] WARNING: src/c_api/c_api.cc:935: `ntree_limit` is deprecated, use `iteration_range` instead.
## [15:58:04] WARNING: src/c_api/c_api.cc:935: `ntree_limit` is deprecated, use `iteration_range` instead.
## [15:58:04] WARNING: src/c_api/c_api.cc:935: `ntree_limit` is deprecated, use `iteration_range` instead.
## [15:58:04] WARNING: src/c_api/c_api.cc:935: `ntree_limit` is deprecated, use `iteration_range` instead.
## [15:58:04] WARNING: src/c_api/c_api.cc:935: `ntree_limit` is deprecated, use `iteration_range` instead.
## [15:58:04] WARNING: src/c_api/c_api.cc:935: `ntree_limit` is deprecated, use `iteration_range` instead.
## [15:58:04] WARNING: src/c_api/c_api.cc:935: `ntree_limit` is deprecated, use `iteration_range` instead.
## [15:58:04] WARNING: src/c_api/c_api.cc:935: `ntree_limit` is deprecated, use `iteration_range` instead.
## [15:58:04] WARNING: src/c_api/c_api.cc:935: `ntree_limit` is deprecated, use `iteration_range` instead.
## [15:58:04] WARNING: src/c_api/c_api.cc:935: `ntree_limit` is deprecated, use `iteration_range` instead.
## [15:58:04] WARNING: src/c_api/c_api.cc:935: `ntree_limit` is deprecated, use `iteration_range` instead.
## [15:58:04] WARNING: src/c_api/c_api.cc:935: `ntree_limit` is deprecated, use `iteration_range` instead.
## [15:58:05] WARNING: src/c_api/c_api.cc:935: `ntree_limit` is deprecated, use `iteration_range` instead.
## [15:58:05] WARNING: src/c_api/c_api.cc:935: `ntree_limit` is deprecated, use `iteration_range` instead.
## [15:58:05] WARNING: src/c_api/c_api.cc:935: `ntree_limit` is deprecated, use `iteration_range` instead.
## [15:58:05] WARNING: src/c_api/c_api.cc:935: `ntree_limit` is deprecated, use `iteration_range` instead.
## [15:58:05] WARNING: src/c_api/c_api.cc:935: `ntree_limit` is deprecated, use `iteration_range` instead.
## [15:58:05] WARNING: src/c_api/c_api.cc:935: `ntree_limit` is deprecated, use `iteration_range` instead.
## [15:58:05] WARNING: src/c_api/c_api.cc:935: `ntree_limit` is deprecated, use `iteration_range` instead.
## [15:58:05] WARNING: src/c_api/c_api.cc:935: `ntree_limit` is deprecated, use `iteration_range` instead.
## [15:58:05] WARNING: src/c_api/c_api.cc:935: `ntree_limit` is deprecated, use `iteration_range` instead.
## [15:58:05] WARNING: src/c_api/c_api.cc:935: `ntree_limit` is deprecated, use `iteration_range` instead.
## [15:58:06] WARNING: src/c_api/c_api.cc:935: `ntree_limit` is deprecated, use `iteration_range` instead.
## [15:58:06] WARNING: src/c_api/c_api.cc:935: `ntree_limit` is deprecated, use `iteration_range` instead.
## [15:58:06] WARNING: src/c_api/c_api.cc:935: `ntree_limit` is deprecated, use `iteration_range` instead.
## [15:58:06] WARNING: src/c_api/c_api.cc:935: `ntree_limit` is deprecated, use `iteration_range` instead.
## [15:58:06] WARNING: src/c_api/c_api.cc:935: `ntree_limit` is deprecated, use `iteration_range` instead.
## [15:58:06] WARNING: src/c_api/c_api.cc:935: `ntree_limit` is deprecated, use `iteration_range` instead.
## [15:58:06] WARNING: src/c_api/c_api.cc:935: `ntree_limit` is deprecated, use `iteration_range` instead.
## [15:58:06] WARNING: src/c_api/c_api.cc:935: `ntree_limit` is deprecated, use `iteration_range` instead.
## [15:58:06] WARNING: src/c_api/c_api.cc:935: `ntree_limit` is deprecated, use `iteration_range` instead.
## [15:58:06] WARNING: src/c_api/c_api.cc:935: `ntree_limit` is deprecated, use `iteration_range` instead.
## [15:58:06] WARNING: src/c_api/c_api.cc:935: `ntree_limit` is deprecated, use `iteration_range` instead.
## [15:58:06] WARNING: src/c_api/c_api.cc:935: `ntree_limit` is deprecated, use `iteration_range` instead.
## [15:58:06] WARNING: src/c_api/c_api.cc:935: `ntree_limit` is deprecated, use `iteration_range` instead.
## [15:58:06] WARNING: src/c_api/c_api.cc:935: `ntree_limit` is deprecated, use `iteration_range` instead.
## [15:58:07] WARNING: src/c_api/c_api.cc:935: `ntree_limit` is deprecated, use `iteration_range` instead.
## [15:58:07] WARNING: src/c_api/c_api.cc:935: `ntree_limit` is deprecated, use `iteration_range` instead.
## [15:58:07] WARNING: src/c_api/c_api.cc:935: `ntree_limit` is deprecated, use `iteration_range` instead.
## [15:58:07] WARNING: src/c_api/c_api.cc:935: `ntree_limit` is deprecated, use `iteration_range` instead.
## [15:58:07] WARNING: src/c_api/c_api.cc:935: `ntree_limit` is deprecated, use `iteration_range` instead.
## [15:58:07] WARNING: src/c_api/c_api.cc:935: `ntree_limit` is deprecated, use `iteration_range` instead.
## [15:58:07] WARNING: src/c_api/c_api.cc:935: `ntree_limit` is deprecated, use `iteration_range` instead.
## [15:58:07] WARNING: src/c_api/c_api.cc:935: `ntree_limit` is deprecated, use `iteration_range` instead.
## [15:58:07] WARNING: src/c_api/c_api.cc:935: `ntree_limit` is deprecated, use `iteration_range` instead.
## [15:58:07] WARNING: src/c_api/c_api.cc:935: `ntree_limit` is deprecated, use `iteration_range` instead.
## [15:58:08] WARNING: src/c_api/c_api.cc:935: `ntree_limit` is deprecated, use `iteration_range` instead.
## [15:58:08] WARNING: src/c_api/c_api.cc:935: `ntree_limit` is deprecated, use `iteration_range` instead.
## [15:58:08] WARNING: src/c_api/c_api.cc:935: `ntree_limit` is deprecated, use `iteration_range` instead.
## [15:58:08] WARNING: src/c_api/c_api.cc:935: `ntree_limit` is deprecated, use `iteration_range` instead.
## [15:58:08] WARNING: src/c_api/c_api.cc:935: `ntree_limit` is deprecated, use `iteration_range` instead.
## [15:58:08] WARNING: src/c_api/c_api.cc:935: `ntree_limit` is deprecated, use `iteration_range` instead.
## [15:58:08] WARNING: src/c_api/c_api.cc:935: `ntree_limit` is deprecated, use `iteration_range` instead.
## [15:58:08] WARNING: src/c_api/c_api.cc:935: `ntree_limit` is deprecated, use `iteration_range` instead.
## [15:58:08] WARNING: src/c_api/c_api.cc:935: `ntree_limit` is deprecated, use `iteration_range` instead.
## [15:58:08] WARNING: src/c_api/c_api.cc:935: `ntree_limit` is deprecated, use `iteration_range` instead.
## [15:58:09] WARNING: src/c_api/c_api.cc:935: `ntree_limit` is deprecated, use `iteration_range` instead.
## [15:58:09] WARNING: src/c_api/c_api.cc:935: `ntree_limit` is deprecated, use `iteration_range` instead.
## [15:58:09] WARNING: src/c_api/c_api.cc:935: `ntree_limit` is deprecated, use `iteration_range` instead.
## [15:58:09] WARNING: src/c_api/c_api.cc:935: `ntree_limit` is deprecated, use `iteration_range` instead.
## [15:58:09] WARNING: src/c_api/c_api.cc:935: `ntree_limit` is deprecated, use `iteration_range` instead.
## [15:58:09] WARNING: src/c_api/c_api.cc:935: `ntree_limit` is deprecated, use `iteration_range` instead.
## [15:58:09] WARNING: src/c_api/c_api.cc:935: `ntree_limit` is deprecated, use `iteration_range` instead.
## [15:58:09] WARNING: src/c_api/c_api.cc:935: `ntree_limit` is deprecated, use `iteration_range` instead.
## [15:58:09] WARNING: src/c_api/c_api.cc:935: `ntree_limit` is deprecated, use `iteration_range` instead.
## [15:58:09] WARNING: src/c_api/c_api.cc:935: `ntree_limit` is deprecated, use `iteration_range` instead.
## [15:58:09] WARNING: src/c_api/c_api.cc:935: `ntree_limit` is deprecated, use `iteration_range` instead.
## [15:58:09] WARNING: src/c_api/c_api.cc:935: `ntree_limit` is deprecated, use `iteration_range` instead.
## [15:58:10] WARNING: src/c_api/c_api.cc:935: `ntree_limit` is deprecated, use `iteration_range` instead.
## [15:58:10] WARNING: src/c_api/c_api.cc:935: `ntree_limit` is deprecated, use `iteration_range` instead.
## [15:58:10] WARNING: src/c_api/c_api.cc:935: `ntree_limit` is deprecated, use `iteration_range` instead.
## [15:58:10] WARNING: src/c_api/c_api.cc:935: `ntree_limit` is deprecated, use `iteration_range` instead.
## [15:58:10] WARNING: src/c_api/c_api.cc:935: `ntree_limit` is deprecated, use `iteration_range` instead.
## [15:58:10] WARNING: src/c_api/c_api.cc:935: `ntree_limit` is deprecated, use `iteration_range` instead.
## [15:58:10] WARNING: src/c_api/c_api.cc:935: `ntree_limit` is deprecated, use `iteration_range` instead.
## [15:58:10] WARNING: src/c_api/c_api.cc:935: `ntree_limit` is deprecated, use `iteration_range` instead.
## [15:58:10] WARNING: src/c_api/c_api.cc:935: `ntree_limit` is deprecated, use `iteration_range` instead.
## [15:58:10] WARNING: src/c_api/c_api.cc:935: `ntree_limit` is deprecated, use `iteration_range` instead.
## [15:58:10] WARNING: src/c_api/c_api.cc:935: `ntree_limit` is deprecated, use `iteration_range` instead.
## [15:58:10] WARNING: src/c_api/c_api.cc:935: `ntree_limit` is deprecated, use `iteration_range` instead.
## [15:58:10] WARNING: src/c_api/c_api.cc:935: `ntree_limit` is deprecated, use `iteration_range` instead.
## [15:58:10] WARNING: src/c_api/c_api.cc:935: `ntree_limit` is deprecated, use `iteration_range` instead.
## [15:58:11] WARNING: src/c_api/c_api.cc:935: `ntree_limit` is deprecated, use `iteration_range` instead.
## [15:58:11] WARNING: src/c_api/c_api.cc:935: `ntree_limit` is deprecated, use `iteration_range` instead.
## [15:58:11] WARNING: src/c_api/c_api.cc:935: `ntree_limit` is deprecated, use `iteration_range` instead.
## [15:58:11] WARNING: src/c_api/c_api.cc:935: `ntree_limit` is deprecated, use `iteration_range` instead.
## [15:58:11] WARNING: src/c_api/c_api.cc:935: `ntree_limit` is deprecated, use `iteration_range` instead.
## [15:58:11] WARNING: src/c_api/c_api.cc:935: `ntree_limit` is deprecated, use `iteration_range` instead.
## [15:58:11] WARNING: src/c_api/c_api.cc:935: `ntree_limit` is deprecated, use `iteration_range` instead.
## [15:58:11] WARNING: src/c_api/c_api.cc:935: `ntree_limit` is deprecated, use `iteration_range` instead.
## [15:58:11] WARNING: src/c_api/c_api.cc:935: `ntree_limit` is deprecated, use `iteration_range` instead.
## [15:58:11] WARNING: src/c_api/c_api.cc:935: `ntree_limit` is deprecated, use `iteration_range` instead.
## [15:58:12] WARNING: src/c_api/c_api.cc:935: `ntree_limit` is deprecated, use `iteration_range` instead.
## [15:58:12] WARNING: src/c_api/c_api.cc:935: `ntree_limit` is deprecated, use `iteration_range` instead.
## [15:58:12] WARNING: src/c_api/c_api.cc:935: `ntree_limit` is deprecated, use `iteration_range` instead.
## [15:58:12] WARNING: src/c_api/c_api.cc:935: `ntree_limit` is deprecated, use `iteration_range` instead.
## [15:58:12] WARNING: src/c_api/c_api.cc:935: `ntree_limit` is deprecated, use `iteration_range` instead.
## [15:58:12] WARNING: src/c_api/c_api.cc:935: `ntree_limit` is deprecated, use `iteration_range` instead.
## [15:58:15] WARNING: src/c_api/c_api.cc:935: `ntree_limit` is deprecated, use `iteration_range` instead.
## [15:58:15] WARNING: src/c_api/c_api.cc:935: `ntree_limit` is deprecated, use `iteration_range` instead.
## [15:58:15] WARNING: src/c_api/c_api.cc:935: `ntree_limit` is deprecated, use `iteration_range` instead.
## [15:58:15] WARNING: src/c_api/c_api.cc:935: `ntree_limit` is deprecated, use `iteration_range` instead.
## [15:58:15] WARNING: src/c_api/c_api.cc:935: `ntree_limit` is deprecated, use `iteration_range` instead.
## [15:58:15] WARNING: src/c_api/c_api.cc:935: `ntree_limit` is deprecated, use `iteration_range` instead.
## [15:58:15] WARNING: src/c_api/c_api.cc:935: `ntree_limit` is deprecated, use `iteration_range` instead.
## [15:58:15] WARNING: src/c_api/c_api.cc:935: `ntree_limit` is deprecated, use `iteration_range` instead.
## [15:58:15] WARNING: src/c_api/c_api.cc:935: `ntree_limit` is deprecated, use `iteration_range` instead.
## [15:58:15] WARNING: src/c_api/c_api.cc:935: `ntree_limit` is deprecated, use `iteration_range` instead.
## [15:58:15] WARNING: src/c_api/c_api.cc:935: `ntree_limit` is deprecated, use `iteration_range` instead.
## [15:58:15] WARNING: src/c_api/c_api.cc:935: `ntree_limit` is deprecated, use `iteration_range` instead.
## [15:58:16] WARNING: src/c_api/c_api.cc:935: `ntree_limit` is deprecated, use `iteration_range` instead.
## [15:58:16] WARNING: src/c_api/c_api.cc:935: `ntree_limit` is deprecated, use `iteration_range` instead.
## [15:58:16] WARNING: src/c_api/c_api.cc:935: `ntree_limit` is deprecated, use `iteration_range` instead.
## [15:58:16] WARNING: src/c_api/c_api.cc:935: `ntree_limit` is deprecated, use `iteration_range` instead.
## [15:58:16] WARNING: src/c_api/c_api.cc:935: `ntree_limit` is deprecated, use `iteration_range` instead.
## [15:58:16] WARNING: src/c_api/c_api.cc:935: `ntree_limit` is deprecated, use `iteration_range` instead.
## [15:58:16] WARNING: src/c_api/c_api.cc:935: `ntree_limit` is deprecated, use `iteration_range` instead.
## [15:58:16] WARNING: src/c_api/c_api.cc:935: `ntree_limit` is deprecated, use `iteration_range` instead.
## [15:58:16] WARNING: src/c_api/c_api.cc:935: `ntree_limit` is deprecated, use `iteration_range` instead.
## [15:58:16] WARNING: src/c_api/c_api.cc:935: `ntree_limit` is deprecated, use `iteration_range` instead.
## [15:58:16] WARNING: src/c_api/c_api.cc:935: `ntree_limit` is deprecated, use `iteration_range` instead.
## [15:58:16] WARNING: src/c_api/c_api.cc:935: `ntree_limit` is deprecated, use `iteration_range` instead.
## [15:58:17] WARNING: src/c_api/c_api.cc:935: `ntree_limit` is deprecated, use `iteration_range` instead.
## [15:58:17] WARNING: src/c_api/c_api.cc:935: `ntree_limit` is deprecated, use `iteration_range` instead.
## [15:58:17] WARNING: src/c_api/c_api.cc:935: `ntree_limit` is deprecated, use `iteration_range` instead.
## [15:58:17] WARNING: src/c_api/c_api.cc:935: `ntree_limit` is deprecated, use `iteration_range` instead.
## [15:58:17] WARNING: src/c_api/c_api.cc:935: `ntree_limit` is deprecated, use `iteration_range` instead.
## [15:58:17] WARNING: src/c_api/c_api.cc:935: `ntree_limit` is deprecated, use `iteration_range` instead.
## [15:58:17] WARNING: src/c_api/c_api.cc:935: `ntree_limit` is deprecated, use `iteration_range` instead.
## [15:58:17] WARNING: src/c_api/c_api.cc:935: `ntree_limit` is deprecated, use `iteration_range` instead.
## [15:58:17] WARNING: src/c_api/c_api.cc:935: `ntree_limit` is deprecated, use `iteration_range` instead.
## [15:58:17] WARNING: src/c_api/c_api.cc:935: `ntree_limit` is deprecated, use `iteration_range` instead.
## [15:58:18] WARNING: src/c_api/c_api.cc:935: `ntree_limit` is deprecated, use `iteration_range` instead.
## [15:58:18] WARNING: src/c_api/c_api.cc:935: `ntree_limit` is deprecated, use `iteration_range` instead.
## [15:58:18] WARNING: src/c_api/c_api.cc:935: `ntree_limit` is deprecated, use `iteration_range` instead.
## [15:58:18] WARNING: src/c_api/c_api.cc:935: `ntree_limit` is deprecated, use `iteration_range` instead.
## [15:58:18] WARNING: src/c_api/c_api.cc:935: `ntree_limit` is deprecated, use `iteration_range` instead.
## [15:58:18] WARNING: src/c_api/c_api.cc:935: `ntree_limit` is deprecated, use `iteration_range` instead.
## [15:58:18] WARNING: src/c_api/c_api.cc:935: `ntree_limit` is deprecated, use `iteration_range` instead.
## [15:58:18] WARNING: src/c_api/c_api.cc:935: `ntree_limit` is deprecated, use `iteration_range` instead.
## [15:58:18] WARNING: src/c_api/c_api.cc:935: `ntree_limit` is deprecated, use `iteration_range` instead.
## [15:58:18] WARNING: src/c_api/c_api.cc:935: `ntree_limit` is deprecated, use `iteration_range` instead.
## [15:58:18] WARNING: src/c_api/c_api.cc:935: `ntree_limit` is deprecated, use `iteration_range` instead.
## [15:58:18] WARNING: src/c_api/c_api.cc:935: `ntree_limit` is deprecated, use `iteration_range` instead.
## [15:58:18] WARNING: src/c_api/c_api.cc:935: `ntree_limit` is deprecated, use `iteration_range` instead.
## [15:58:18] WARNING: src/c_api/c_api.cc:935: `ntree_limit` is deprecated, use `iteration_range` instead.
## [15:58:18] WARNING: src/c_api/c_api.cc:935: `ntree_limit` is deprecated, use `iteration_range` instead.
## [15:58:18] WARNING: src/c_api/c_api.cc:935: `ntree_limit` is deprecated, use `iteration_range` instead.
## [15:58:19] WARNING: src/c_api/c_api.cc:935: `ntree_limit` is deprecated, use `iteration_range` instead.
## [15:58:19] WARNING: src/c_api/c_api.cc:935: `ntree_limit` is deprecated, use `iteration_range` instead.
## [15:58:19] WARNING: src/c_api/c_api.cc:935: `ntree_limit` is deprecated, use `iteration_range` instead.
## [15:58:19] WARNING: src/c_api/c_api.cc:935: `ntree_limit` is deprecated, use `iteration_range` instead.
## [15:58:19] WARNING: src/c_api/c_api.cc:935: `ntree_limit` is deprecated, use `iteration_range` instead.
## [15:58:19] WARNING: src/c_api/c_api.cc:935: `ntree_limit` is deprecated, use `iteration_range` instead.
## [15:58:19] WARNING: src/c_api/c_api.cc:935: `ntree_limit` is deprecated, use `iteration_range` instead.
## [15:58:19] WARNING: src/c_api/c_api.cc:935: `ntree_limit` is deprecated, use `iteration_range` instead.
## [15:58:19] WARNING: src/c_api/c_api.cc:935: `ntree_limit` is deprecated, use `iteration_range` instead.
## [15:58:19] WARNING: src/c_api/c_api.cc:935: `ntree_limit` is deprecated, use `iteration_range` instead.
## [15:58:20] WARNING: src/c_api/c_api.cc:935: `ntree_limit` is deprecated, use `iteration_range` instead.
## [15:58:20] WARNING: src/c_api/c_api.cc:935: `ntree_limit` is deprecated, use `iteration_range` instead.
## [15:58:20] WARNING: src/c_api/c_api.cc:935: `ntree_limit` is deprecated, use `iteration_range` instead.
## [15:58:20] WARNING: src/c_api/c_api.cc:935: `ntree_limit` is deprecated, use `iteration_range` instead.
## [15:58:20] WARNING: src/c_api/c_api.cc:935: `ntree_limit` is deprecated, use `iteration_range` instead.
## [15:58:20] WARNING: src/c_api/c_api.cc:935: `ntree_limit` is deprecated, use `iteration_range` instead.
## [15:58:20] WARNING: src/c_api/c_api.cc:935: `ntree_limit` is deprecated, use `iteration_range` instead.
## [15:58:20] WARNING: src/c_api/c_api.cc:935: `ntree_limit` is deprecated, use `iteration_range` instead.
## [15:58:20] WARNING: src/c_api/c_api.cc:935: `ntree_limit` is deprecated, use `iteration_range` instead.
## [15:58:20] WARNING: src/c_api/c_api.cc:935: `ntree_limit` is deprecated, use `iteration_range` instead.
## [15:58:20] WARNING: src/c_api/c_api.cc:935: `ntree_limit` is deprecated, use `iteration_range` instead.
## [15:58:20] WARNING: src/c_api/c_api.cc:935: `ntree_limit` is deprecated, use `iteration_range` instead.
## [15:58:21] WARNING: src/c_api/c_api.cc:935: `ntree_limit` is deprecated, use `iteration_range` instead.
## [15:58:21] WARNING: src/c_api/c_api.cc:935: `ntree_limit` is deprecated, use `iteration_range` instead.
## [15:58:21] WARNING: src/c_api/c_api.cc:935: `ntree_limit` is deprecated, use `iteration_range` instead.
## [15:58:21] WARNING: src/c_api/c_api.cc:935: `ntree_limit` is deprecated, use `iteration_range` instead.
## [15:58:21] WARNING: src/c_api/c_api.cc:935: `ntree_limit` is deprecated, use `iteration_range` instead.
## [15:58:21] WARNING: src/c_api/c_api.cc:935: `ntree_limit` is deprecated, use `iteration_range` instead.
## [15:58:21] WARNING: src/c_api/c_api.cc:935: `ntree_limit` is deprecated, use `iteration_range` instead.
## [15:58:21] WARNING: src/c_api/c_api.cc:935: `ntree_limit` is deprecated, use `iteration_range` instead.
## [15:58:21] WARNING: src/c_api/c_api.cc:935: `ntree_limit` is deprecated, use `iteration_range` instead.
## [15:58:21] WARNING: src/c_api/c_api.cc:935: `ntree_limit` is deprecated, use `iteration_range` instead.
## [15:58:21] WARNING: src/c_api/c_api.cc:935: `ntree_limit` is deprecated, use `iteration_range` instead.
## [15:58:21] WARNING: src/c_api/c_api.cc:935: `ntree_limit` is deprecated, use `iteration_range` instead.
## [15:58:21] WARNING: src/c_api/c_api.cc:935: `ntree_limit` is deprecated, use `iteration_range` instead.
## [15:58:21] WARNING: src/c_api/c_api.cc:935: `ntree_limit` is deprecated, use `iteration_range` instead.
## [15:58:22] WARNING: src/c_api/c_api.cc:935: `ntree_limit` is deprecated, use `iteration_range` instead.
## [15:58:22] WARNING: src/c_api/c_api.cc:935: `ntree_limit` is deprecated, use `iteration_range` instead.
## [15:58:22] WARNING: src/c_api/c_api.cc:935: `ntree_limit` is deprecated, use `iteration_range` instead.
## [15:58:22] WARNING: src/c_api/c_api.cc:935: `ntree_limit` is deprecated, use `iteration_range` instead.
## [15:58:22] WARNING: src/c_api/c_api.cc:935: `ntree_limit` is deprecated, use `iteration_range` instead.
## [15:58:22] WARNING: src/c_api/c_api.cc:935: `ntree_limit` is deprecated, use `iteration_range` instead.
## [15:58:22] WARNING: src/c_api/c_api.cc:935: `ntree_limit` is deprecated, use `iteration_range` instead.
## [15:58:22] WARNING: src/c_api/c_api.cc:935: `ntree_limit` is deprecated, use `iteration_range` instead.
## [15:58:22] WARNING: src/c_api/c_api.cc:935: `ntree_limit` is deprecated, use `iteration_range` instead.
## [15:58:22] WARNING: src/c_api/c_api.cc:935: `ntree_limit` is deprecated, use `iteration_range` instead.
## [15:58:23] WARNING: src/c_api/c_api.cc:935: `ntree_limit` is deprecated, use `iteration_range` instead.
## [15:58:23] WARNING: src/c_api/c_api.cc:935: `ntree_limit` is deprecated, use `iteration_range` instead.
## [15:58:23] WARNING: src/c_api/c_api.cc:935: `ntree_limit` is deprecated, use `iteration_range` instead.
## [15:58:23] WARNING: src/c_api/c_api.cc:935: `ntree_limit` is deprecated, use `iteration_range` instead.
## [15:58:23] WARNING: src/c_api/c_api.cc:935: `ntree_limit` is deprecated, use `iteration_range` instead.
## [15:58:23] WARNING: src/c_api/c_api.cc:935: `ntree_limit` is deprecated, use `iteration_range` instead.
## [15:58:23] WARNING: src/c_api/c_api.cc:935: `ntree_limit` is deprecated, use `iteration_range` instead.
## [15:58:23] WARNING: src/c_api/c_api.cc:935: `ntree_limit` is deprecated, use `iteration_range` instead.
## [15:58:23] WARNING: src/c_api/c_api.cc:935: `ntree_limit` is deprecated, use `iteration_range` instead.
## [15:58:23] WARNING: src/c_api/c_api.cc:935: `ntree_limit` is deprecated, use `iteration_range` instead.
## [15:58:24] WARNING: src/c_api/c_api.cc:935: `ntree_limit` is deprecated, use `iteration_range` instead.
## [15:58:24] WARNING: src/c_api/c_api.cc:935: `ntree_limit` is deprecated, use `iteration_range` instead.
## [15:58:24] WARNING: src/c_api/c_api.cc:935: `ntree_limit` is deprecated, use `iteration_range` instead.
## [15:58:24] WARNING: src/c_api/c_api.cc:935: `ntree_limit` is deprecated, use `iteration_range` instead.
## [15:58:24] WARNING: src/c_api/c_api.cc:935: `ntree_limit` is deprecated, use `iteration_range` instead.
## [15:58:24] WARNING: src/c_api/c_api.cc:935: `ntree_limit` is deprecated, use `iteration_range` instead.
## [15:58:24] WARNING: src/c_api/c_api.cc:935: `ntree_limit` is deprecated, use `iteration_range` instead.
## [15:58:24] WARNING: src/c_api/c_api.cc:935: `ntree_limit` is deprecated, use `iteration_range` instead.
## [15:58:24] WARNING: src/c_api/c_api.cc:935: `ntree_limit` is deprecated, use `iteration_range` instead.
## [15:58:24] WARNING: src/c_api/c_api.cc:935: `ntree_limit` is deprecated, use `iteration_range` instead.
## [15:58:24] WARNING: src/c_api/c_api.cc:935: `ntree_limit` is deprecated, use `iteration_range` instead.
## [15:58:24] WARNING: src/c_api/c_api.cc:935: `ntree_limit` is deprecated, use `iteration_range` instead.
## [15:58:24] WARNING: src/c_api/c_api.cc:935: `ntree_limit` is deprecated, use `iteration_range` instead.
## [15:58:24] WARNING: src/c_api/c_api.cc:935: `ntree_limit` is deprecated, use `iteration_range` instead.
## [15:58:25] WARNING: src/c_api/c_api.cc:935: `ntree_limit` is deprecated, use `iteration_range` instead.
## [15:58:25] WARNING: src/c_api/c_api.cc:935: `ntree_limit` is deprecated, use `iteration_range` instead.
## [15:58:25] WARNING: src/c_api/c_api.cc:935: `ntree_limit` is deprecated, use `iteration_range` instead.
## [15:58:25] WARNING: src/c_api/c_api.cc:935: `ntree_limit` is deprecated, use `iteration_range` instead.
## [15:58:25] WARNING: src/c_api/c_api.cc:935: `ntree_limit` is deprecated, use `iteration_range` instead.
## [15:58:25] WARNING: src/c_api/c_api.cc:935: `ntree_limit` is deprecated, use `iteration_range` instead.
## [15:58:25] WARNING: src/c_api/c_api.cc:935: `ntree_limit` is deprecated, use `iteration_range` instead.
## [15:58:25] WARNING: src/c_api/c_api.cc:935: `ntree_limit` is deprecated, use `iteration_range` instead.
## [15:58:25] WARNING: src/c_api/c_api.cc:935: `ntree_limit` is deprecated, use `iteration_range` instead.
## [15:58:25] WARNING: src/c_api/c_api.cc:935: `ntree_limit` is deprecated, use `iteration_range` instead.
## [15:58:25] WARNING: src/c_api/c_api.cc:935: `ntree_limit` is deprecated, use `iteration_range` instead.
## [15:58:25] WARNING: src/c_api/c_api.cc:935: `ntree_limit` is deprecated, use `iteration_range` instead.
## [15:58:26] WARNING: src/c_api/c_api.cc:935: `ntree_limit` is deprecated, use `iteration_range` instead.
## [15:58:26] WARNING: src/c_api/c_api.cc:935: `ntree_limit` is deprecated, use `iteration_range` instead.
## [15:58:26] WARNING: src/c_api/c_api.cc:935: `ntree_limit` is deprecated, use `iteration_range` instead.
## [15:58:26] WARNING: src/c_api/c_api.cc:935: `ntree_limit` is deprecated, use `iteration_range` instead.
## [15:58:26] WARNING: src/c_api/c_api.cc:935: `ntree_limit` is deprecated, use `iteration_range` instead.
## [15:58:26] WARNING: src/c_api/c_api.cc:935: `ntree_limit` is deprecated, use `iteration_range` instead.
## [15:58:26] WARNING: src/c_api/c_api.cc:935: `ntree_limit` is deprecated, use `iteration_range` instead.
## [15:58:26] WARNING: src/c_api/c_api.cc:935: `ntree_limit` is deprecated, use `iteration_range` instead.
## [15:58:26] WARNING: src/c_api/c_api.cc:935: `ntree_limit` is deprecated, use `iteration_range` instead.
## [15:58:26] WARNING: src/c_api/c_api.cc:935: `ntree_limit` is deprecated, use `iteration_range` instead.
## [15:58:27] WARNING: src/c_api/c_api.cc:935: `ntree_limit` is deprecated, use `iteration_range` instead.
## [15:58:27] WARNING: src/c_api/c_api.cc:935: `ntree_limit` is deprecated, use `iteration_range` instead.
## [15:58:27] WARNING: src/c_api/c_api.cc:935: `ntree_limit` is deprecated, use `iteration_range` instead.
## [15:58:27] WARNING: src/c_api/c_api.cc:935: `ntree_limit` is deprecated, use `iteration_range` instead.
## [15:58:27] WARNING: src/c_api/c_api.cc:935: `ntree_limit` is deprecated, use `iteration_range` instead.
## [15:58:27] WARNING: src/c_api/c_api.cc:935: `ntree_limit` is deprecated, use `iteration_range` instead.
## [15:58:27] WARNING: src/c_api/c_api.cc:935: `ntree_limit` is deprecated, use `iteration_range` instead.
## [15:58:27] WARNING: src/c_api/c_api.cc:935: `ntree_limit` is deprecated, use `iteration_range` instead.
## [15:58:27] WARNING: src/c_api/c_api.cc:935: `ntree_limit` is deprecated, use `iteration_range` instead.
## [15:58:27] WARNING: src/c_api/c_api.cc:935: `ntree_limit` is deprecated, use `iteration_range` instead.
## [15:58:27] WARNING: src/c_api/c_api.cc:935: `ntree_limit` is deprecated, use `iteration_range` instead.
## [15:58:27] WARNING: src/c_api/c_api.cc:935: `ntree_limit` is deprecated, use `iteration_range` instead.
## [15:58:27] WARNING: src/c_api/c_api.cc:935: `ntree_limit` is deprecated, use `iteration_range` instead.
## [15:58:27] WARNING: src/c_api/c_api.cc:935: `ntree_limit` is deprecated, use `iteration_range` instead.
## [15:58:28] WARNING: src/c_api/c_api.cc:935: `ntree_limit` is deprecated, use `iteration_range` instead.
## [15:58:28] WARNING: src/c_api/c_api.cc:935: `ntree_limit` is deprecated, use `iteration_range` instead.
## [15:58:28] WARNING: src/c_api/c_api.cc:935: `ntree_limit` is deprecated, use `iteration_range` instead.
## [15:58:28] WARNING: src/c_api/c_api.cc:935: `ntree_limit` is deprecated, use `iteration_range` instead.
## [15:58:28] WARNING: src/c_api/c_api.cc:935: `ntree_limit` is deprecated, use `iteration_range` instead.
## [15:58:28] WARNING: src/c_api/c_api.cc:935: `ntree_limit` is deprecated, use `iteration_range` instead.
## [15:58:28] WARNING: src/c_api/c_api.cc:935: `ntree_limit` is deprecated, use `iteration_range` instead.
## [15:58:28] WARNING: src/c_api/c_api.cc:935: `ntree_limit` is deprecated, use `iteration_range` instead.
## [15:58:28] WARNING: src/c_api/c_api.cc:935: `ntree_limit` is deprecated, use `iteration_range` instead.
## [15:58:28] WARNING: src/c_api/c_api.cc:935: `ntree_limit` is deprecated, use `iteration_range` instead.
## [15:58:28] WARNING: src/c_api/c_api.cc:935: `ntree_limit` is deprecated, use `iteration_range` instead.
## [15:58:28] WARNING: src/c_api/c_api.cc:935: `ntree_limit` is deprecated, use `iteration_range` instead.
## [15:58:29] WARNING: src/c_api/c_api.cc:935: `ntree_limit` is deprecated, use `iteration_range` instead.
## [15:58:29] WARNING: src/c_api/c_api.cc:935: `ntree_limit` is deprecated, use `iteration_range` instead.
## [15:58:29] WARNING: src/c_api/c_api.cc:935: `ntree_limit` is deprecated, use `iteration_range` instead.
## [15:58:29] WARNING: src/c_api/c_api.cc:935: `ntree_limit` is deprecated, use `iteration_range` instead.
## [15:58:29] WARNING: src/c_api/c_api.cc:935: `ntree_limit` is deprecated, use `iteration_range` instead.
## [15:58:29] WARNING: src/c_api/c_api.cc:935: `ntree_limit` is deprecated, use `iteration_range` instead.
## [15:58:29] WARNING: src/c_api/c_api.cc:935: `ntree_limit` is deprecated, use `iteration_range` instead.
## [15:58:29] WARNING: src/c_api/c_api.cc:935: `ntree_limit` is deprecated, use `iteration_range` instead.
## [15:58:29] WARNING: src/c_api/c_api.cc:935: `ntree_limit` is deprecated, use `iteration_range` instead.
## [15:58:29] WARNING: src/c_api/c_api.cc:935: `ntree_limit` is deprecated, use `iteration_range` instead.
## [15:58:30] WARNING: src/c_api/c_api.cc:935: `ntree_limit` is deprecated, use `iteration_range` instead.
## [15:58:30] WARNING: src/c_api/c_api.cc:935: `ntree_limit` is deprecated, use `iteration_range` instead.
## [15:58:30] WARNING: src/c_api/c_api.cc:935: `ntree_limit` is deprecated, use `iteration_range` instead.
## [15:58:30] WARNING: src/c_api/c_api.cc:935: `ntree_limit` is deprecated, use `iteration_range` instead.
## [15:58:30] WARNING: src/c_api/c_api.cc:935: `ntree_limit` is deprecated, use `iteration_range` instead.
## [15:58:30] WARNING: src/c_api/c_api.cc:935: `ntree_limit` is deprecated, use `iteration_range` instead.
## [15:58:30] WARNING: src/c_api/c_api.cc:935: `ntree_limit` is deprecated, use `iteration_range` instead.
## [15:58:30] WARNING: src/c_api/c_api.cc:935: `ntree_limit` is deprecated, use `iteration_range` instead.
## [15:58:30] WARNING: src/c_api/c_api.cc:935: `ntree_limit` is deprecated, use `iteration_range` instead.
## [15:58:30] WARNING: src/c_api/c_api.cc:935: `ntree_limit` is deprecated, use `iteration_range` instead.
## [15:58:30] WARNING: src/c_api/c_api.cc:935: `ntree_limit` is deprecated, use `iteration_range` instead.
## [15:58:30] WARNING: src/c_api/c_api.cc:935: `ntree_limit` is deprecated, use `iteration_range` instead.
## [15:58:30] WARNING: src/c_api/c_api.cc:935: `ntree_limit` is deprecated, use `iteration_range` instead.
## [15:58:30] WARNING: src/c_api/c_api.cc:935: `ntree_limit` is deprecated, use `iteration_range` instead.
## [15:58:30] WARNING: src/c_api/c_api.cc:935: `ntree_limit` is deprecated, use `iteration_range` instead.
## [15:58:31] WARNING: src/c_api/c_api.cc:935: `ntree_limit` is deprecated, use `iteration_range` instead.
## [15:58:31] WARNING: src/c_api/c_api.cc:935: `ntree_limit` is deprecated, use `iteration_range` instead.
## [15:58:31] WARNING: src/c_api/c_api.cc:935: `ntree_limit` is deprecated, use `iteration_range` instead.
## [15:58:31] WARNING: src/c_api/c_api.cc:935: `ntree_limit` is deprecated, use `iteration_range` instead.
## [15:58:31] WARNING: src/c_api/c_api.cc:935: `ntree_limit` is deprecated, use `iteration_range` instead.
## [15:58:31] WARNING: src/c_api/c_api.cc:935: `ntree_limit` is deprecated, use `iteration_range` instead.
## [15:58:31] WARNING: src/c_api/c_api.cc:935: `ntree_limit` is deprecated, use `iteration_range` instead.
## [15:58:31] WARNING: src/c_api/c_api.cc:935: `ntree_limit` is deprecated, use `iteration_range` instead.
## [15:58:31] WARNING: src/c_api/c_api.cc:935: `ntree_limit` is deprecated, use `iteration_range` instead.
## [15:58:31] WARNING: src/c_api/c_api.cc:935: `ntree_limit` is deprecated, use `iteration_range` instead.
## [15:58:31] WARNING: src/c_api/c_api.cc:935: `ntree_limit` is deprecated, use `iteration_range` instead.
## [15:58:32] WARNING: src/c_api/c_api.cc:935: `ntree_limit` is deprecated, use `iteration_range` instead.
## [15:58:32] WARNING: src/c_api/c_api.cc:935: `ntree_limit` is deprecated, use `iteration_range` instead.
## [15:58:32] WARNING: src/c_api/c_api.cc:935: `ntree_limit` is deprecated, use `iteration_range` instead.
## [15:58:32] WARNING: src/c_api/c_api.cc:935: `ntree_limit` is deprecated, use `iteration_range` instead.
## [15:58:32] WARNING: src/c_api/c_api.cc:935: `ntree_limit` is deprecated, use `iteration_range` instead.
## [15:58:32] WARNING: src/c_api/c_api.cc:935: `ntree_limit` is deprecated, use `iteration_range` instead.
## [15:58:32] WARNING: src/c_api/c_api.cc:935: `ntree_limit` is deprecated, use `iteration_range` instead.
## [15:58:32] WARNING: src/c_api/c_api.cc:935: `ntree_limit` is deprecated, use `iteration_range` instead.
## [15:58:32] WARNING: src/c_api/c_api.cc:935: `ntree_limit` is deprecated, use `iteration_range` instead.
## [15:58:32] WARNING: src/c_api/c_api.cc:935: `ntree_limit` is deprecated, use `iteration_range` instead.
## [15:58:32] WARNING: src/c_api/c_api.cc:935: `ntree_limit` is deprecated, use `iteration_range` instead.
## [15:58:32] WARNING: src/c_api/c_api.cc:935: `ntree_limit` is deprecated, use `iteration_range` instead.
## [15:58:33] WARNING: src/c_api/c_api.cc:935: `ntree_limit` is deprecated, use `iteration_range` instead.
## [15:58:33] WARNING: src/c_api/c_api.cc:935: `ntree_limit` is deprecated, use `iteration_range` instead.
## [15:58:33] WARNING: src/c_api/c_api.cc:935: `ntree_limit` is deprecated, use `iteration_range` instead.
## [15:58:33] WARNING: src/c_api/c_api.cc:935: `ntree_limit` is deprecated, use `iteration_range` instead.
## [15:58:33] WARNING: src/c_api/c_api.cc:935: `ntree_limit` is deprecated, use `iteration_range` instead.
## [15:58:33] WARNING: src/c_api/c_api.cc:935: `ntree_limit` is deprecated, use `iteration_range` instead.
## [15:58:33] WARNING: src/c_api/c_api.cc:935: `ntree_limit` is deprecated, use `iteration_range` instead.
## [15:58:33] WARNING: src/c_api/c_api.cc:935: `ntree_limit` is deprecated, use `iteration_range` instead.
## [15:58:33] WARNING: src/c_api/c_api.cc:935: `ntree_limit` is deprecated, use `iteration_range` instead.
## [15:58:33] WARNING: src/c_api/c_api.cc:935: `ntree_limit` is deprecated, use `iteration_range` instead.
## [15:58:33] WARNING: src/c_api/c_api.cc:935: `ntree_limit` is deprecated, use `iteration_range` instead.
## [15:58:33] WARNING: src/c_api/c_api.cc:935: `ntree_limit` is deprecated, use `iteration_range` instead.
## [15:58:33] WARNING: src/c_api/c_api.cc:935: `ntree_limit` is deprecated, use `iteration_range` instead.
## [15:58:33] WARNING: src/c_api/c_api.cc:935: `ntree_limit` is deprecated, use `iteration_range` instead.
## [15:58:34] WARNING: src/c_api/c_api.cc:935: `ntree_limit` is deprecated, use `iteration_range` instead.
## [15:58:34] WARNING: src/c_api/c_api.cc:935: `ntree_limit` is deprecated, use `iteration_range` instead.
## [15:58:34] WARNING: src/c_api/c_api.cc:935: `ntree_limit` is deprecated, use `iteration_range` instead.
## [15:58:34] WARNING: src/c_api/c_api.cc:935: `ntree_limit` is deprecated, use `iteration_range` instead.
## [15:58:34] WARNING: src/c_api/c_api.cc:935: `ntree_limit` is deprecated, use `iteration_range` instead.
## [15:58:34] WARNING: src/c_api/c_api.cc:935: `ntree_limit` is deprecated, use `iteration_range` instead.
## [15:58:34] WARNING: src/c_api/c_api.cc:935: `ntree_limit` is deprecated, use `iteration_range` instead.
## [15:58:34] WARNING: src/c_api/c_api.cc:935: `ntree_limit` is deprecated, use `iteration_range` instead.
## [15:58:34] WARNING: src/c_api/c_api.cc:935: `ntree_limit` is deprecated, use `iteration_range` instead.
## [15:58:34] WARNING: src/c_api/c_api.cc:935: `ntree_limit` is deprecated, use `iteration_range` instead.
## [15:58:34] WARNING: src/c_api/c_api.cc:935: `ntree_limit` is deprecated, use `iteration_range` instead.
## [15:58:34] WARNING: src/c_api/c_api.cc:935: `ntree_limit` is deprecated, use `iteration_range` instead.
## [15:58:35] WARNING: src/c_api/c_api.cc:935: `ntree_limit` is deprecated, use `iteration_range` instead.
## [15:58:35] WARNING: src/c_api/c_api.cc:935: `ntree_limit` is deprecated, use `iteration_range` instead.
## [15:58:35] WARNING: src/c_api/c_api.cc:935: `ntree_limit` is deprecated, use `iteration_range` instead.
## [15:58:35] WARNING: src/c_api/c_api.cc:935: `ntree_limit` is deprecated, use `iteration_range` instead.
## [15:58:35] WARNING: src/c_api/c_api.cc:935: `ntree_limit` is deprecated, use `iteration_range` instead.
## [15:58:35] WARNING: src/c_api/c_api.cc:935: `ntree_limit` is deprecated, use `iteration_range` instead.
## [15:58:35] WARNING: src/c_api/c_api.cc:935: `ntree_limit` is deprecated, use `iteration_range` instead.
## [15:58:35] WARNING: src/c_api/c_api.cc:935: `ntree_limit` is deprecated, use `iteration_range` instead.
## [15:58:35] WARNING: src/c_api/c_api.cc:935: `ntree_limit` is deprecated, use `iteration_range` instead.
## [15:58:35] WARNING: src/c_api/c_api.cc:935: `ntree_limit` is deprecated, use `iteration_range` instead.
## [15:58:36] WARNING: src/c_api/c_api.cc:935: `ntree_limit` is deprecated, use `iteration_range` instead.
## [15:58:36] WARNING: src/c_api/c_api.cc:935: `ntree_limit` is deprecated, use `iteration_range` instead.
## [15:58:36] WARNING: src/c_api/c_api.cc:935: `ntree_limit` is deprecated, use `iteration_range` instead.
## [15:58:36] WARNING: src/c_api/c_api.cc:935: `ntree_limit` is deprecated, use `iteration_range` instead.
## [15:58:36] WARNING: src/c_api/c_api.cc:935: `ntree_limit` is deprecated, use `iteration_range` instead.
## [15:58:36] WARNING: src/c_api/c_api.cc:935: `ntree_limit` is deprecated, use `iteration_range` instead.
## [15:58:36] WARNING: src/c_api/c_api.cc:935: `ntree_limit` is deprecated, use `iteration_range` instead.
## [15:58:36] WARNING: src/c_api/c_api.cc:935: `ntree_limit` is deprecated, use `iteration_range` instead.
## [15:58:36] WARNING: src/c_api/c_api.cc:935: `ntree_limit` is deprecated, use `iteration_range` instead.
## [15:58:36] WARNING: src/c_api/c_api.cc:935: `ntree_limit` is deprecated, use `iteration_range` instead.
## [15:58:36] WARNING: src/c_api/c_api.cc:935: `ntree_limit` is deprecated, use `iteration_range` instead.
## [15:58:36] WARNING: src/c_api/c_api.cc:935: `ntree_limit` is deprecated, use `iteration_range` instead.
## [15:58:36] WARNING: src/c_api/c_api.cc:935: `ntree_limit` is deprecated, use `iteration_range` instead.
## [15:58:36] WARNING: src/c_api/c_api.cc:935: `ntree_limit` is deprecated, use `iteration_range` instead.
## [15:58:36] WARNING: src/c_api/c_api.cc:935: `ntree_limit` is deprecated, use `iteration_range` instead.
## [15:58:36] WARNING: src/c_api/c_api.cc:935: `ntree_limit` is deprecated, use `iteration_range` instead.
## [15:58:37] WARNING: src/c_api/c_api.cc:935: `ntree_limit` is deprecated, use `iteration_range` instead.
## [15:58:37] WARNING: src/c_api/c_api.cc:935: `ntree_limit` is deprecated, use `iteration_range` instead.
## [15:58:37] WARNING: src/c_api/c_api.cc:935: `ntree_limit` is deprecated, use `iteration_range` instead.
## [15:58:37] WARNING: src/c_api/c_api.cc:935: `ntree_limit` is deprecated, use `iteration_range` instead.
## [15:58:37] WARNING: src/c_api/c_api.cc:935: `ntree_limit` is deprecated, use `iteration_range` instead.
## [15:58:37] WARNING: src/c_api/c_api.cc:935: `ntree_limit` is deprecated, use `iteration_range` instead.
## [15:58:37] WARNING: src/c_api/c_api.cc:935: `ntree_limit` is deprecated, use `iteration_range` instead.
## [15:58:37] WARNING: src/c_api/c_api.cc:935: `ntree_limit` is deprecated, use `iteration_range` instead.
## [15:58:37] WARNING: src/c_api/c_api.cc:935: `ntree_limit` is deprecated, use `iteration_range` instead.
## [15:58:37] WARNING: src/c_api/c_api.cc:935: `ntree_limit` is deprecated, use `iteration_range` instead.
## [15:58:38] WARNING: src/c_api/c_api.cc:935: `ntree_limit` is deprecated, use `iteration_range` instead.
## [15:58:38] WARNING: src/c_api/c_api.cc:935: `ntree_limit` is deprecated, use `iteration_range` instead.
## [15:58:38] WARNING: src/c_api/c_api.cc:935: `ntree_limit` is deprecated, use `iteration_range` instead.
## [15:58:38] WARNING: src/c_api/c_api.cc:935: `ntree_limit` is deprecated, use `iteration_range` instead.
## [15:58:38] WARNING: src/c_api/c_api.cc:935: `ntree_limit` is deprecated, use `iteration_range` instead.
## [15:58:38] WARNING: src/c_api/c_api.cc:935: `ntree_limit` is deprecated, use `iteration_range` instead.
## [15:58:38] WARNING: src/c_api/c_api.cc:935: `ntree_limit` is deprecated, use `iteration_range` instead.
## [15:58:38] WARNING: src/c_api/c_api.cc:935: `ntree_limit` is deprecated, use `iteration_range` instead.
## [15:58:38] WARNING: src/c_api/c_api.cc:935: `ntree_limit` is deprecated, use `iteration_range` instead.
## [15:58:38] WARNING: src/c_api/c_api.cc:935: `ntree_limit` is deprecated, use `iteration_range` instead.
## [15:58:41] WARNING: src/c_api/c_api.cc:935: `ntree_limit` is deprecated, use `iteration_range` instead.
## [15:58:41] WARNING: src/c_api/c_api.cc:935: `ntree_limit` is deprecated, use `iteration_range` instead.
## [15:58:41] WARNING: src/c_api/c_api.cc:935: `ntree_limit` is deprecated, use `iteration_range` instead.
## [15:58:41] WARNING: src/c_api/c_api.cc:935: `ntree_limit` is deprecated, use `iteration_range` instead.
## [15:58:41] WARNING: src/c_api/c_api.cc:935: `ntree_limit` is deprecated, use `iteration_range` instead.
## [15:58:41] WARNING: src/c_api/c_api.cc:935: `ntree_limit` is deprecated, use `iteration_range` instead.
## [15:58:41] WARNING: src/c_api/c_api.cc:935: `ntree_limit` is deprecated, use `iteration_range` instead.
## [15:58:41] WARNING: src/c_api/c_api.cc:935: `ntree_limit` is deprecated, use `iteration_range` instead.
## [15:58:42] WARNING: src/c_api/c_api.cc:935: `ntree_limit` is deprecated, use `iteration_range` instead.
## [15:58:42] WARNING: src/c_api/c_api.cc:935: `ntree_limit` is deprecated, use `iteration_range` instead.
## [15:58:42] WARNING: src/c_api/c_api.cc:935: `ntree_limit` is deprecated, use `iteration_range` instead.
## [15:58:42] WARNING: src/c_api/c_api.cc:935: `ntree_limit` is deprecated, use `iteration_range` instead.
## [15:58:42] WARNING: src/c_api/c_api.cc:935: `ntree_limit` is deprecated, use `iteration_range` instead.
## [15:58:42] WARNING: src/c_api/c_api.cc:935: `ntree_limit` is deprecated, use `iteration_range` instead.
## [15:58:42] WARNING: src/c_api/c_api.cc:935: `ntree_limit` is deprecated, use `iteration_range` instead.
## [15:58:42] WARNING: src/c_api/c_api.cc:935: `ntree_limit` is deprecated, use `iteration_range` instead.
## [15:58:42] WARNING: src/c_api/c_api.cc:935: `ntree_limit` is deprecated, use `iteration_range` instead.
## [15:58:42] WARNING: src/c_api/c_api.cc:935: `ntree_limit` is deprecated, use `iteration_range` instead.
## [15:58:42] WARNING: src/c_api/c_api.cc:935: `ntree_limit` is deprecated, use `iteration_range` instead.
## [15:58:42] WARNING: src/c_api/c_api.cc:935: `ntree_limit` is deprecated, use `iteration_range` instead.
## [15:58:43] WARNING: src/c_api/c_api.cc:935: `ntree_limit` is deprecated, use `iteration_range` instead.
## [15:58:43] WARNING: src/c_api/c_api.cc:935: `ntree_limit` is deprecated, use `iteration_range` instead.
## [15:58:43] WARNING: src/c_api/c_api.cc:935: `ntree_limit` is deprecated, use `iteration_range` instead.
## [15:58:43] WARNING: src/c_api/c_api.cc:935: `ntree_limit` is deprecated, use `iteration_range` instead.
## [15:58:43] WARNING: src/c_api/c_api.cc:935: `ntree_limit` is deprecated, use `iteration_range` instead.
## [15:58:43] WARNING: src/c_api/c_api.cc:935: `ntree_limit` is deprecated, use `iteration_range` instead.
## [15:58:43] WARNING: src/c_api/c_api.cc:935: `ntree_limit` is deprecated, use `iteration_range` instead.
## [15:58:43] WARNING: src/c_api/c_api.cc:935: `ntree_limit` is deprecated, use `iteration_range` instead.
## [15:58:44] WARNING: src/c_api/c_api.cc:935: `ntree_limit` is deprecated, use `iteration_range` instead.
## [15:58:44] WARNING: src/c_api/c_api.cc:935: `ntree_limit` is deprecated, use `iteration_range` instead.
## [15:58:44] WARNING: src/c_api/c_api.cc:935: `ntree_limit` is deprecated, use `iteration_range` instead.
## [15:58:44] WARNING: src/c_api/c_api.cc:935: `ntree_limit` is deprecated, use `iteration_range` instead.
## [15:58:44] WARNING: src/c_api/c_api.cc:935: `ntree_limit` is deprecated, use `iteration_range` instead.
## [15:58:44] WARNING: src/c_api/c_api.cc:935: `ntree_limit` is deprecated, use `iteration_range` instead.
## [15:58:44] WARNING: src/c_api/c_api.cc:935: `ntree_limit` is deprecated, use `iteration_range` instead.
## [15:58:44] WARNING: src/c_api/c_api.cc:935: `ntree_limit` is deprecated, use `iteration_range` instead.
## [15:58:44] WARNING: src/c_api/c_api.cc:935: `ntree_limit` is deprecated, use `iteration_range` instead.
## [15:58:44] WARNING: src/c_api/c_api.cc:935: `ntree_limit` is deprecated, use `iteration_range` instead.
## [15:58:45] WARNING: src/c_api/c_api.cc:935: `ntree_limit` is deprecated, use `iteration_range` instead.
## [15:58:45] WARNING: src/c_api/c_api.cc:935: `ntree_limit` is deprecated, use `iteration_range` instead.
## [15:58:45] WARNING: src/c_api/c_api.cc:935: `ntree_limit` is deprecated, use `iteration_range` instead.
## [15:58:45] WARNING: src/c_api/c_api.cc:935: `ntree_limit` is deprecated, use `iteration_range` instead.
## [15:58:45] WARNING: src/c_api/c_api.cc:935: `ntree_limit` is deprecated, use `iteration_range` instead.
## [15:58:45] WARNING: src/c_api/c_api.cc:935: `ntree_limit` is deprecated, use `iteration_range` instead.
## [15:58:45] WARNING: src/c_api/c_api.cc:935: `ntree_limit` is deprecated, use `iteration_range` instead.
## [15:58:45] WARNING: src/c_api/c_api.cc:935: `ntree_limit` is deprecated, use `iteration_range` instead.
## [15:58:45] WARNING: src/c_api/c_api.cc:935: `ntree_limit` is deprecated, use `iteration_range` instead.
## [15:58:45] WARNING: src/c_api/c_api.cc:935: `ntree_limit` is deprecated, use `iteration_range` instead.
## [15:58:45] WARNING: src/c_api/c_api.cc:935: `ntree_limit` is deprecated, use `iteration_range` instead.
## [15:58:45] WARNING: src/c_api/c_api.cc:935: `ntree_limit` is deprecated, use `iteration_range` instead.
## [15:58:45] WARNING: src/c_api/c_api.cc:935: `ntree_limit` is deprecated, use `iteration_range` instead.
## [15:58:45] WARNING: src/c_api/c_api.cc:935: `ntree_limit` is deprecated, use `iteration_range` instead.
## [15:58:46] WARNING: src/c_api/c_api.cc:935: `ntree_limit` is deprecated, use `iteration_range` instead.
## [15:58:46] WARNING: src/c_api/c_api.cc:935: `ntree_limit` is deprecated, use `iteration_range` instead.
## [15:58:46] WARNING: src/c_api/c_api.cc:935: `ntree_limit` is deprecated, use `iteration_range` instead.
## [15:58:46] WARNING: src/c_api/c_api.cc:935: `ntree_limit` is deprecated, use `iteration_range` instead.
## [15:58:46] WARNING: src/c_api/c_api.cc:935: `ntree_limit` is deprecated, use `iteration_range` instead.
## [15:58:46] WARNING: src/c_api/c_api.cc:935: `ntree_limit` is deprecated, use `iteration_range` instead.
## [15:58:46] WARNING: src/c_api/c_api.cc:935: `ntree_limit` is deprecated, use `iteration_range` instead.
## [15:58:46] WARNING: src/c_api/c_api.cc:935: `ntree_limit` is deprecated, use `iteration_range` instead.
## [15:58:46] WARNING: src/c_api/c_api.cc:935: `ntree_limit` is deprecated, use `iteration_range` instead.
## [15:58:46] WARNING: src/c_api/c_api.cc:935: `ntree_limit` is deprecated, use `iteration_range` instead.
## [15:58:47] WARNING: src/c_api/c_api.cc:935: `ntree_limit` is deprecated, use `iteration_range` instead.
## [15:58:47] WARNING: src/c_api/c_api.cc:935: `ntree_limit` is deprecated, use `iteration_range` instead.
## [15:58:47] WARNING: src/c_api/c_api.cc:935: `ntree_limit` is deprecated, use `iteration_range` instead.
## [15:58:47] WARNING: src/c_api/c_api.cc:935: `ntree_limit` is deprecated, use `iteration_range` instead.
## [15:58:47] WARNING: src/c_api/c_api.cc:935: `ntree_limit` is deprecated, use `iteration_range` instead.
## [15:58:47] WARNING: src/c_api/c_api.cc:935: `ntree_limit` is deprecated, use `iteration_range` instead.
## [15:58:47] WARNING: src/c_api/c_api.cc:935: `ntree_limit` is deprecated, use `iteration_range` instead.
## [15:58:47] WARNING: src/c_api/c_api.cc:935: `ntree_limit` is deprecated, use `iteration_range` instead.
## [15:58:47] WARNING: src/c_api/c_api.cc:935: `ntree_limit` is deprecated, use `iteration_range` instead.
## [15:58:47] WARNING: src/c_api/c_api.cc:935: `ntree_limit` is deprecated, use `iteration_range` instead.
## [15:58:48] WARNING: src/c_api/c_api.cc:935: `ntree_limit` is deprecated, use `iteration_range` instead.
## [15:58:48] WARNING: src/c_api/c_api.cc:935: `ntree_limit` is deprecated, use `iteration_range` instead.
## [15:58:48] WARNING: src/c_api/c_api.cc:935: `ntree_limit` is deprecated, use `iteration_range` instead.
## [15:58:48] WARNING: src/c_api/c_api.cc:935: `ntree_limit` is deprecated, use `iteration_range` instead.
## [15:58:48] WARNING: src/c_api/c_api.cc:935: `ntree_limit` is deprecated, use `iteration_range` instead.
## [15:58:48] WARNING: src/c_api/c_api.cc:935: `ntree_limit` is deprecated, use `iteration_range` instead.
## [15:58:48] WARNING: src/c_api/c_api.cc:935: `ntree_limit` is deprecated, use `iteration_range` instead.
## [15:58:48] WARNING: src/c_api/c_api.cc:935: `ntree_limit` is deprecated, use `iteration_range` instead.
## [15:58:48] WARNING: src/c_api/c_api.cc:935: `ntree_limit` is deprecated, use `iteration_range` instead.
## [15:58:48] WARNING: src/c_api/c_api.cc:935: `ntree_limit` is deprecated, use `iteration_range` instead.
## [15:58:48] WARNING: src/c_api/c_api.cc:935: `ntree_limit` is deprecated, use `iteration_range` instead.
## [15:58:48] WARNING: src/c_api/c_api.cc:935: `ntree_limit` is deprecated, use `iteration_range` instead.
## [15:58:48] WARNING: src/c_api/c_api.cc:935: `ntree_limit` is deprecated, use `iteration_range` instead.
## [15:58:48] WARNING: src/c_api/c_api.cc:935: `ntree_limit` is deprecated, use `iteration_range` instead.
## [15:58:49] WARNING: src/c_api/c_api.cc:935: `ntree_limit` is deprecated, use `iteration_range` instead.
## [15:58:49] WARNING: src/c_api/c_api.cc:935: `ntree_limit` is deprecated, use `iteration_range` instead.
## [15:58:49] WARNING: src/c_api/c_api.cc:935: `ntree_limit` is deprecated, use `iteration_range` instead.
## [15:58:49] WARNING: src/c_api/c_api.cc:935: `ntree_limit` is deprecated, use `iteration_range` instead.
## [15:58:49] WARNING: src/c_api/c_api.cc:935: `ntree_limit` is deprecated, use `iteration_range` instead.
## [15:58:49] WARNING: src/c_api/c_api.cc:935: `ntree_limit` is deprecated, use `iteration_range` instead.
## [15:58:49] WARNING: src/c_api/c_api.cc:935: `ntree_limit` is deprecated, use `iteration_range` instead.
## [15:58:49] WARNING: src/c_api/c_api.cc:935: `ntree_limit` is deprecated, use `iteration_range` instead.
## [15:58:49] WARNING: src/c_api/c_api.cc:935: `ntree_limit` is deprecated, use `iteration_range` instead.
## [15:58:49] WARNING: src/c_api/c_api.cc:935: `ntree_limit` is deprecated, use `iteration_range` instead.
## [15:58:50] WARNING: src/c_api/c_api.cc:935: `ntree_limit` is deprecated, use `iteration_range` instead.
## [15:58:50] WARNING: src/c_api/c_api.cc:935: `ntree_limit` is deprecated, use `iteration_range` instead.
## [15:58:50] WARNING: src/c_api/c_api.cc:935: `ntree_limit` is deprecated, use `iteration_range` instead.
## [15:58:50] WARNING: src/c_api/c_api.cc:935: `ntree_limit` is deprecated, use `iteration_range` instead.
## [15:58:50] WARNING: src/c_api/c_api.cc:935: `ntree_limit` is deprecated, use `iteration_range` instead.
## [15:58:50] WARNING: src/c_api/c_api.cc:935: `ntree_limit` is deprecated, use `iteration_range` instead.
## [15:58:50] WARNING: src/c_api/c_api.cc:935: `ntree_limit` is deprecated, use `iteration_range` instead.
## [15:58:50] WARNING: src/c_api/c_api.cc:935: `ntree_limit` is deprecated, use `iteration_range` instead.
## [15:58:50] WARNING: src/c_api/c_api.cc:935: `ntree_limit` is deprecated, use `iteration_range` instead.
## [15:58:50] WARNING: src/c_api/c_api.cc:935: `ntree_limit` is deprecated, use `iteration_range` instead.
## [15:58:51] WARNING: src/c_api/c_api.cc:935: `ntree_limit` is deprecated, use `iteration_range` instead.
## [15:58:51] WARNING: src/c_api/c_api.cc:935: `ntree_limit` is deprecated, use `iteration_range` instead.
## [15:58:51] WARNING: src/c_api/c_api.cc:935: `ntree_limit` is deprecated, use `iteration_range` instead.
## [15:58:51] WARNING: src/c_api/c_api.cc:935: `ntree_limit` is deprecated, use `iteration_range` instead.
## [15:58:51] WARNING: src/c_api/c_api.cc:935: `ntree_limit` is deprecated, use `iteration_range` instead.
## [15:58:51] WARNING: src/c_api/c_api.cc:935: `ntree_limit` is deprecated, use `iteration_range` instead.
## [15:58:51] WARNING: src/c_api/c_api.cc:935: `ntree_limit` is deprecated, use `iteration_range` instead.
## [15:58:51] WARNING: src/c_api/c_api.cc:935: `ntree_limit` is deprecated, use `iteration_range` instead.
## [15:58:51] WARNING: src/c_api/c_api.cc:935: `ntree_limit` is deprecated, use `iteration_range` instead.
## [15:58:51] WARNING: src/c_api/c_api.cc:935: `ntree_limit` is deprecated, use `iteration_range` instead.
## [15:58:51] WARNING: src/c_api/c_api.cc:935: `ntree_limit` is deprecated, use `iteration_range` instead.
## [15:58:51] WARNING: src/c_api/c_api.cc:935: `ntree_limit` is deprecated, use `iteration_range` instead.
## [15:58:52] WARNING: src/c_api/c_api.cc:935: `ntree_limit` is deprecated, use `iteration_range` instead.
## [15:58:52] WARNING: src/c_api/c_api.cc:935: `ntree_limit` is deprecated, use `iteration_range` instead.
## [15:58:52] WARNING: src/c_api/c_api.cc:935: `ntree_limit` is deprecated, use `iteration_range` instead.
## [15:58:52] WARNING: src/c_api/c_api.cc:935: `ntree_limit` is deprecated, use `iteration_range` instead.
## [15:58:52] WARNING: src/c_api/c_api.cc:935: `ntree_limit` is deprecated, use `iteration_range` instead.
## [15:58:52] WARNING: src/c_api/c_api.cc:935: `ntree_limit` is deprecated, use `iteration_range` instead.
## [15:58:52] WARNING: src/c_api/c_api.cc:935: `ntree_limit` is deprecated, use `iteration_range` instead.
## [15:58:52] WARNING: src/c_api/c_api.cc:935: `ntree_limit` is deprecated, use `iteration_range` instead.
## [15:58:52] WARNING: src/c_api/c_api.cc:935: `ntree_limit` is deprecated, use `iteration_range` instead.
## [15:58:52] WARNING: src/c_api/c_api.cc:935: `ntree_limit` is deprecated, use `iteration_range` instead.
## [15:58:52] WARNING: src/c_api/c_api.cc:935: `ntree_limit` is deprecated, use `iteration_range` instead.
## [15:58:52] WARNING: src/c_api/c_api.cc:935: `ntree_limit` is deprecated, use `iteration_range` instead.
## [15:58:53] WARNING: src/c_api/c_api.cc:935: `ntree_limit` is deprecated, use `iteration_range` instead.
## [15:58:53] WARNING: src/c_api/c_api.cc:935: `ntree_limit` is deprecated, use `iteration_range` instead.
## [15:58:53] WARNING: src/c_api/c_api.cc:935: `ntree_limit` is deprecated, use `iteration_range` instead.
## [15:58:53] WARNING: src/c_api/c_api.cc:935: `ntree_limit` is deprecated, use `iteration_range` instead.
## [15:58:53] WARNING: src/c_api/c_api.cc:935: `ntree_limit` is deprecated, use `iteration_range` instead.
## [15:58:53] WARNING: src/c_api/c_api.cc:935: `ntree_limit` is deprecated, use `iteration_range` instead.
## [15:58:53] WARNING: src/c_api/c_api.cc:935: `ntree_limit` is deprecated, use `iteration_range` instead.
## [15:58:53] WARNING: src/c_api/c_api.cc:935: `ntree_limit` is deprecated, use `iteration_range` instead.
## [15:58:53] WARNING: src/c_api/c_api.cc:935: `ntree_limit` is deprecated, use `iteration_range` instead.
## [15:58:53] WARNING: src/c_api/c_api.cc:935: `ntree_limit` is deprecated, use `iteration_range` instead.
## [15:58:54] WARNING: src/c_api/c_api.cc:935: `ntree_limit` is deprecated, use `iteration_range` instead.
## [15:58:54] WARNING: src/c_api/c_api.cc:935: `ntree_limit` is deprecated, use `iteration_range` instead.
## [15:58:54] WARNING: src/c_api/c_api.cc:935: `ntree_limit` is deprecated, use `iteration_range` instead.
## [15:58:54] WARNING: src/c_api/c_api.cc:935: `ntree_limit` is deprecated, use `iteration_range` instead.
## [15:58:54] WARNING: src/c_api/c_api.cc:935: `ntree_limit` is deprecated, use `iteration_range` instead.
## [15:58:54] WARNING: src/c_api/c_api.cc:935: `ntree_limit` is deprecated, use `iteration_range` instead.
## [15:58:54] WARNING: src/c_api/c_api.cc:935: `ntree_limit` is deprecated, use `iteration_range` instead.
## [15:58:54] WARNING: src/c_api/c_api.cc:935: `ntree_limit` is deprecated, use `iteration_range` instead.
## [15:58:54] WARNING: src/c_api/c_api.cc:935: `ntree_limit` is deprecated, use `iteration_range` instead.
## [15:58:54] WARNING: src/c_api/c_api.cc:935: `ntree_limit` is deprecated, use `iteration_range` instead.
## [15:58:54] WARNING: src/c_api/c_api.cc:935: `ntree_limit` is deprecated, use `iteration_range` instead.
## [15:58:54] WARNING: src/c_api/c_api.cc:935: `ntree_limit` is deprecated, use `iteration_range` instead.
## [15:58:55] WARNING: src/c_api/c_api.cc:935: `ntree_limit` is deprecated, use `iteration_range` instead.
## [15:58:55] WARNING: src/c_api/c_api.cc:935: `ntree_limit` is deprecated, use `iteration_range` instead.
## [15:58:55] WARNING: src/c_api/c_api.cc:935: `ntree_limit` is deprecated, use `iteration_range` instead.
## [15:58:55] WARNING: src/c_api/c_api.cc:935: `ntree_limit` is deprecated, use `iteration_range` instead.
## [15:58:55] WARNING: src/c_api/c_api.cc:935: `ntree_limit` is deprecated, use `iteration_range` instead.
## [15:58:55] WARNING: src/c_api/c_api.cc:935: `ntree_limit` is deprecated, use `iteration_range` instead.
## [15:58:55] WARNING: src/c_api/c_api.cc:935: `ntree_limit` is deprecated, use `iteration_range` instead.
## [15:58:55] WARNING: src/c_api/c_api.cc:935: `ntree_limit` is deprecated, use `iteration_range` instead.
## [15:58:55] WARNING: src/c_api/c_api.cc:935: `ntree_limit` is deprecated, use `iteration_range` instead.
## [15:58:55] WARNING: src/c_api/c_api.cc:935: `ntree_limit` is deprecated, use `iteration_range` instead.
## [15:58:55] WARNING: src/c_api/c_api.cc:935: `ntree_limit` is deprecated, use `iteration_range` instead.
## [15:58:55] WARNING: src/c_api/c_api.cc:935: `ntree_limit` is deprecated, use `iteration_range` instead.
## [15:58:56] WARNING: src/c_api/c_api.cc:935: `ntree_limit` is deprecated, use `iteration_range` instead.
## [15:58:56] WARNING: src/c_api/c_api.cc:935: `ntree_limit` is deprecated, use `iteration_range` instead.
## [15:58:56] WARNING: src/c_api/c_api.cc:935: `ntree_limit` is deprecated, use `iteration_range` instead.
## [15:58:56] WARNING: src/c_api/c_api.cc:935: `ntree_limit` is deprecated, use `iteration_range` instead.
## [15:58:56] WARNING: src/c_api/c_api.cc:935: `ntree_limit` is deprecated, use `iteration_range` instead.
## [15:58:56] WARNING: src/c_api/c_api.cc:935: `ntree_limit` is deprecated, use `iteration_range` instead.
## [15:58:56] WARNING: src/c_api/c_api.cc:935: `ntree_limit` is deprecated, use `iteration_range` instead.
## [15:58:56] WARNING: src/c_api/c_api.cc:935: `ntree_limit` is deprecated, use `iteration_range` instead.
## [15:58:56] WARNING: src/c_api/c_api.cc:935: `ntree_limit` is deprecated, use `iteration_range` instead.
## [15:58:56] WARNING: src/c_api/c_api.cc:935: `ntree_limit` is deprecated, use `iteration_range` instead.
## [15:58:57] WARNING: src/c_api/c_api.cc:935: `ntree_limit` is deprecated, use `iteration_range` instead.
## [15:58:57] WARNING: src/c_api/c_api.cc:935: `ntree_limit` is deprecated, use `iteration_range` instead.
## [15:58:57] WARNING: src/c_api/c_api.cc:935: `ntree_limit` is deprecated, use `iteration_range` instead.
## [15:58:57] WARNING: src/c_api/c_api.cc:935: `ntree_limit` is deprecated, use `iteration_range` instead.
## [15:58:57] WARNING: src/c_api/c_api.cc:935: `ntree_limit` is deprecated, use `iteration_range` instead.
## [15:58:57] WARNING: src/c_api/c_api.cc:935: `ntree_limit` is deprecated, use `iteration_range` instead.
## [15:58:57] WARNING: src/c_api/c_api.cc:935: `ntree_limit` is deprecated, use `iteration_range` instead.
## [15:58:57] WARNING: src/c_api/c_api.cc:935: `ntree_limit` is deprecated, use `iteration_range` instead.
## [15:58:57] WARNING: src/c_api/c_api.cc:935: `ntree_limit` is deprecated, use `iteration_range` instead.
## [15:58:57] WARNING: src/c_api/c_api.cc:935: `ntree_limit` is deprecated, use `iteration_range` instead.
## [15:58:58] WARNING: src/c_api/c_api.cc:935: `ntree_limit` is deprecated, use `iteration_range` instead.
## [15:58:58] WARNING: src/c_api/c_api.cc:935: `ntree_limit` is deprecated, use `iteration_range` instead.
## [15:58:58] WARNING: src/c_api/c_api.cc:935: `ntree_limit` is deprecated, use `iteration_range` instead.
## [15:58:58] WARNING: src/c_api/c_api.cc:935: `ntree_limit` is deprecated, use `iteration_range` instead.
## [15:58:58] WARNING: src/c_api/c_api.cc:935: `ntree_limit` is deprecated, use `iteration_range` instead.
## [15:58:58] WARNING: src/c_api/c_api.cc:935: `ntree_limit` is deprecated, use `iteration_range` instead.
## [15:58:58] WARNING: src/c_api/c_api.cc:935: `ntree_limit` is deprecated, use `iteration_range` instead.
## [15:58:58] WARNING: src/c_api/c_api.cc:935: `ntree_limit` is deprecated, use `iteration_range` instead.
## [15:58:58] WARNING: src/c_api/c_api.cc:935: `ntree_limit` is deprecated, use `iteration_range` instead.
## [15:58:58] WARNING: src/c_api/c_api.cc:935: `ntree_limit` is deprecated, use `iteration_range` instead.
## [15:58:58] WARNING: src/c_api/c_api.cc:935: `ntree_limit` is deprecated, use `iteration_range` instead.
## [15:58:58] WARNING: src/c_api/c_api.cc:935: `ntree_limit` is deprecated, use `iteration_range` instead.
## [15:58:58] WARNING: src/c_api/c_api.cc:935: `ntree_limit` is deprecated, use `iteration_range` instead.
## [15:58:58] WARNING: src/c_api/c_api.cc:935: `ntree_limit` is deprecated, use `iteration_range` instead.
## [15:58:59] WARNING: src/c_api/c_api.cc:935: `ntree_limit` is deprecated, use `iteration_range` instead.
## [15:58:59] WARNING: src/c_api/c_api.cc:935: `ntree_limit` is deprecated, use `iteration_range` instead.
## [15:58:59] WARNING: src/c_api/c_api.cc:935: `ntree_limit` is deprecated, use `iteration_range` instead.
## [15:58:59] WARNING: src/c_api/c_api.cc:935: `ntree_limit` is deprecated, use `iteration_range` instead.
## [15:58:59] WARNING: src/c_api/c_api.cc:935: `ntree_limit` is deprecated, use `iteration_range` instead.
## [15:58:59] WARNING: src/c_api/c_api.cc:935: `ntree_limit` is deprecated, use `iteration_range` instead.
## [15:58:59] WARNING: src/c_api/c_api.cc:935: `ntree_limit` is deprecated, use `iteration_range` instead.
## [15:58:59] WARNING: src/c_api/c_api.cc:935: `ntree_limit` is deprecated, use `iteration_range` instead.
## [15:58:59] WARNING: src/c_api/c_api.cc:935: `ntree_limit` is deprecated, use `iteration_range` instead.
## [15:58:59] WARNING: src/c_api/c_api.cc:935: `ntree_limit` is deprecated, use `iteration_range` instead.
## [15:59:00] WARNING: src/c_api/c_api.cc:935: `ntree_limit` is deprecated, use `iteration_range` instead.
## [15:59:00] WARNING: src/c_api/c_api.cc:935: `ntree_limit` is deprecated, use `iteration_range` instead.
## [15:59:00] WARNING: src/c_api/c_api.cc:935: `ntree_limit` is deprecated, use `iteration_range` instead.
## [15:59:00] WARNING: src/c_api/c_api.cc:935: `ntree_limit` is deprecated, use `iteration_range` instead.
## [15:59:00] WARNING: src/c_api/c_api.cc:935: `ntree_limit` is deprecated, use `iteration_range` instead.
## [15:59:00] WARNING: src/c_api/c_api.cc:935: `ntree_limit` is deprecated, use `iteration_range` instead.
## [15:59:00] WARNING: src/c_api/c_api.cc:935: `ntree_limit` is deprecated, use `iteration_range` instead.
## [15:59:00] WARNING: src/c_api/c_api.cc:935: `ntree_limit` is deprecated, use `iteration_range` instead.
## [15:59:00] WARNING: src/c_api/c_api.cc:935: `ntree_limit` is deprecated, use `iteration_range` instead.
## [15:59:00] WARNING: src/c_api/c_api.cc:935: `ntree_limit` is deprecated, use `iteration_range` instead.
## [15:59:01] WARNING: src/c_api/c_api.cc:935: `ntree_limit` is deprecated, use `iteration_range` instead.
## [15:59:01] WARNING: src/c_api/c_api.cc:935: `ntree_limit` is deprecated, use `iteration_range` instead.
## [15:59:01] WARNING: src/c_api/c_api.cc:935: `ntree_limit` is deprecated, use `iteration_range` instead.
## [15:59:01] WARNING: src/c_api/c_api.cc:935: `ntree_limit` is deprecated, use `iteration_range` instead.
## [15:59:01] WARNING: src/c_api/c_api.cc:935: `ntree_limit` is deprecated, use `iteration_range` instead.
## [15:59:01] WARNING: src/c_api/c_api.cc:935: `ntree_limit` is deprecated, use `iteration_range` instead.
## [15:59:01] WARNING: src/c_api/c_api.cc:935: `ntree_limit` is deprecated, use `iteration_range` instead.
## [15:59:01] WARNING: src/c_api/c_api.cc:935: `ntree_limit` is deprecated, use `iteration_range` instead.
## [15:59:01] WARNING: src/c_api/c_api.cc:935: `ntree_limit` is deprecated, use `iteration_range` instead.
## [15:59:01] WARNING: src/c_api/c_api.cc:935: `ntree_limit` is deprecated, use `iteration_range` instead.
## [15:59:01] WARNING: src/c_api/c_api.cc:935: `ntree_limit` is deprecated, use `iteration_range` instead.
## [15:59:01] WARNING: src/c_api/c_api.cc:935: `ntree_limit` is deprecated, use `iteration_range` instead.
## [15:59:02] WARNING: src/c_api/c_api.cc:935: `ntree_limit` is deprecated, use `iteration_range` instead.
## [15:59:02] WARNING: src/c_api/c_api.cc:935: `ntree_limit` is deprecated, use `iteration_range` instead.
## [15:59:02] WARNING: src/c_api/c_api.cc:935: `ntree_limit` is deprecated, use `iteration_range` instead.
## [15:59:02] WARNING: src/c_api/c_api.cc:935: `ntree_limit` is deprecated, use `iteration_range` instead.
## [15:59:02] WARNING: src/c_api/c_api.cc:935: `ntree_limit` is deprecated, use `iteration_range` instead.
## [15:59:02] WARNING: src/c_api/c_api.cc:935: `ntree_limit` is deprecated, use `iteration_range` instead.
## [15:59:02] WARNING: src/c_api/c_api.cc:935: `ntree_limit` is deprecated, use `iteration_range` instead.
## [15:59:02] WARNING: src/c_api/c_api.cc:935: `ntree_limit` is deprecated, use `iteration_range` instead.
## [15:59:02] WARNING: src/c_api/c_api.cc:935: `ntree_limit` is deprecated, use `iteration_range` instead.
## [15:59:02] WARNING: src/c_api/c_api.cc:935: `ntree_limit` is deprecated, use `iteration_range` instead.
## [15:59:02] WARNING: src/c_api/c_api.cc:935: `ntree_limit` is deprecated, use `iteration_range` instead.
## [15:59:02] WARNING: src/c_api/c_api.cc:935: `ntree_limit` is deprecated, use `iteration_range` instead.
## [15:59:03] WARNING: src/c_api/c_api.cc:935: `ntree_limit` is deprecated, use `iteration_range` instead.
## [15:59:03] WARNING: src/c_api/c_api.cc:935: `ntree_limit` is deprecated, use `iteration_range` instead.
## [15:59:03] WARNING: src/c_api/c_api.cc:935: `ntree_limit` is deprecated, use `iteration_range` instead.
## [15:59:03] WARNING: src/c_api/c_api.cc:935: `ntree_limit` is deprecated, use `iteration_range` instead.
## [15:59:03] WARNING: src/c_api/c_api.cc:935: `ntree_limit` is deprecated, use `iteration_range` instead.
## [15:59:03] WARNING: src/c_api/c_api.cc:935: `ntree_limit` is deprecated, use `iteration_range` instead.
## [15:59:03] WARNING: src/c_api/c_api.cc:935: `ntree_limit` is deprecated, use `iteration_range` instead.
## [15:59:03] WARNING: src/c_api/c_api.cc:935: `ntree_limit` is deprecated, use `iteration_range` instead.
## [15:59:04] WARNING: src/c_api/c_api.cc:935: `ntree_limit` is deprecated, use `iteration_range` instead.
## [15:59:04] WARNING: src/c_api/c_api.cc:935: `ntree_limit` is deprecated, use `iteration_range` instead.
## [15:59:04] WARNING: src/c_api/c_api.cc:935: `ntree_limit` is deprecated, use `iteration_range` instead.
## [15:59:04] WARNING: src/c_api/c_api.cc:935: `ntree_limit` is deprecated, use `iteration_range` instead.
## [15:59:04] WARNING: src/c_api/c_api.cc:935: `ntree_limit` is deprecated, use `iteration_range` instead.
## [15:59:04] WARNING: src/c_api/c_api.cc:935: `ntree_limit` is deprecated, use `iteration_range` instead.
## [15:59:04] WARNING: src/c_api/c_api.cc:935: `ntree_limit` is deprecated, use `iteration_range` instead.
## [15:59:04] WARNING: src/c_api/c_api.cc:935: `ntree_limit` is deprecated, use `iteration_range` instead.
## [15:59:04] WARNING: src/c_api/c_api.cc:935: `ntree_limit` is deprecated, use `iteration_range` instead.
## [15:59:04] WARNING: src/c_api/c_api.cc:935: `ntree_limit` is deprecated, use `iteration_range` instead.
## [15:59:04] WARNING: src/c_api/c_api.cc:935: `ntree_limit` is deprecated, use `iteration_range` instead.
## [15:59:04] WARNING: src/c_api/c_api.cc:935: `ntree_limit` is deprecated, use `iteration_range` instead.
## [15:59:04] WARNING: src/c_api/c_api.cc:935: `ntree_limit` is deprecated, use `iteration_range` instead.
## [15:59:04] WARNING: src/c_api/c_api.cc:935: `ntree_limit` is deprecated, use `iteration_range` instead.
## [15:59:05] WARNING: src/c_api/c_api.cc:935: `ntree_limit` is deprecated, use `iteration_range` instead.
## [15:59:05] WARNING: src/c_api/c_api.cc:935: `ntree_limit` is deprecated, use `iteration_range` instead.
## [15:59:05] WARNING: src/c_api/c_api.cc:935: `ntree_limit` is deprecated, use `iteration_range` instead.
## [15:59:05] WARNING: src/c_api/c_api.cc:935: `ntree_limit` is deprecated, use `iteration_range` instead.
## [15:59:05] WARNING: src/c_api/c_api.cc:935: `ntree_limit` is deprecated, use `iteration_range` instead.
## [15:59:05] WARNING: src/c_api/c_api.cc:935: `ntree_limit` is deprecated, use `iteration_range` instead.
## [15:59:05] WARNING: src/c_api/c_api.cc:935: `ntree_limit` is deprecated, use `iteration_range` instead.
## [15:59:05] WARNING: src/c_api/c_api.cc:935: `ntree_limit` is deprecated, use `iteration_range` instead.
## [15:59:05] WARNING: src/c_api/c_api.cc:935: `ntree_limit` is deprecated, use `iteration_range` instead.
## [15:59:05] WARNING: src/c_api/c_api.cc:935: `ntree_limit` is deprecated, use `iteration_range` instead.
## [15:59:05] WARNING: src/c_api/c_api.cc:935: `ntree_limit` is deprecated, use `iteration_range` instead.
## [15:59:05] WARNING: src/c_api/c_api.cc:935: `ntree_limit` is deprecated, use `iteration_range` instead.
## [15:59:06] WARNING: src/c_api/c_api.cc:935: `ntree_limit` is deprecated, use `iteration_range` instead.
## [15:59:06] WARNING: src/c_api/c_api.cc:935: `ntree_limit` is deprecated, use `iteration_range` instead.
## [15:59:06] WARNING: src/c_api/c_api.cc:935: `ntree_limit` is deprecated, use `iteration_range` instead.
## [15:59:06] WARNING: src/c_api/c_api.cc:935: `ntree_limit` is deprecated, use `iteration_range` instead.
## [15:59:06] WARNING: src/c_api/c_api.cc:935: `ntree_limit` is deprecated, use `iteration_range` instead.
## [15:59:06] WARNING: src/c_api/c_api.cc:935: `ntree_limit` is deprecated, use `iteration_range` instead.
## [15:59:06] WARNING: src/c_api/c_api.cc:935: `ntree_limit` is deprecated, use `iteration_range` instead.
## [15:59:06] WARNING: src/c_api/c_api.cc:935: `ntree_limit` is deprecated, use `iteration_range` instead.
## [15:59:06] WARNING: src/c_api/c_api.cc:935: `ntree_limit` is deprecated, use `iteration_range` instead.
## [15:59:06] WARNING: src/c_api/c_api.cc:935: `ntree_limit` is deprecated, use `iteration_range` instead.
## [15:59:07] WARNING: src/c_api/c_api.cc:935: `ntree_limit` is deprecated, use `iteration_range` instead.
## [15:59:07] WARNING: src/c_api/c_api.cc:935: `ntree_limit` is deprecated, use `iteration_range` instead.
## [15:59:10] WARNING: src/c_api/c_api.cc:935: `ntree_limit` is deprecated, use `iteration_range` instead.
## [15:59:10] WARNING: src/c_api/c_api.cc:935: `ntree_limit` is deprecated, use `iteration_range` instead.
## [15:59:10] WARNING: src/c_api/c_api.cc:935: `ntree_limit` is deprecated, use `iteration_range` instead.
## [15:59:10] WARNING: src/c_api/c_api.cc:935: `ntree_limit` is deprecated, use `iteration_range` instead.
## [15:59:10] WARNING: src/c_api/c_api.cc:935: `ntree_limit` is deprecated, use `iteration_range` instead.
## [15:59:10] WARNING: src/c_api/c_api.cc:935: `ntree_limit` is deprecated, use `iteration_range` instead.
## [15:59:10] WARNING: src/c_api/c_api.cc:935: `ntree_limit` is deprecated, use `iteration_range` instead.
## [15:59:10] WARNING: src/c_api/c_api.cc:935: `ntree_limit` is deprecated, use `iteration_range` instead.
## [15:59:10] WARNING: src/c_api/c_api.cc:935: `ntree_limit` is deprecated, use `iteration_range` instead.
## [15:59:10] WARNING: src/c_api/c_api.cc:935: `ntree_limit` is deprecated, use `iteration_range` instead.
## [15:59:10] WARNING: src/c_api/c_api.cc:935: `ntree_limit` is deprecated, use `iteration_range` instead.
## [15:59:10] WARNING: src/c_api/c_api.cc:935: `ntree_limit` is deprecated, use `iteration_range` instead.
## [15:59:11] WARNING: src/c_api/c_api.cc:935: `ntree_limit` is deprecated, use `iteration_range` instead.
## [15:59:11] WARNING: src/c_api/c_api.cc:935: `ntree_limit` is deprecated, use `iteration_range` instead.
## [15:59:11] WARNING: src/c_api/c_api.cc:935: `ntree_limit` is deprecated, use `iteration_range` instead.
## [15:59:11] WARNING: src/c_api/c_api.cc:935: `ntree_limit` is deprecated, use `iteration_range` instead.
## [15:59:11] WARNING: src/c_api/c_api.cc:935: `ntree_limit` is deprecated, use `iteration_range` instead.
## [15:59:11] WARNING: src/c_api/c_api.cc:935: `ntree_limit` is deprecated, use `iteration_range` instead.
## [15:59:11] WARNING: src/c_api/c_api.cc:935: `ntree_limit` is deprecated, use `iteration_range` instead.
## [15:59:11] WARNING: src/c_api/c_api.cc:935: `ntree_limit` is deprecated, use `iteration_range` instead.
## [15:59:11] WARNING: src/c_api/c_api.cc:935: `ntree_limit` is deprecated, use `iteration_range` instead.
## [15:59:11] WARNING: src/c_api/c_api.cc:935: `ntree_limit` is deprecated, use `iteration_range` instead.
## [15:59:11] WARNING: src/c_api/c_api.cc:935: `ntree_limit` is deprecated, use `iteration_range` instead.
## [15:59:11] WARNING: src/c_api/c_api.cc:935: `ntree_limit` is deprecated, use `iteration_range` instead.
## [15:59:12] WARNING: src/c_api/c_api.cc:935: `ntree_limit` is deprecated, use `iteration_range` instead.
## [15:59:12] WARNING: src/c_api/c_api.cc:935: `ntree_limit` is deprecated, use `iteration_range` instead.
## [15:59:12] WARNING: src/c_api/c_api.cc:935: `ntree_limit` is deprecated, use `iteration_range` instead.
## [15:59:12] WARNING: src/c_api/c_api.cc:935: `ntree_limit` is deprecated, use `iteration_range` instead.
## [15:59:12] WARNING: src/c_api/c_api.cc:935: `ntree_limit` is deprecated, use `iteration_range` instead.
## [15:59:12] WARNING: src/c_api/c_api.cc:935: `ntree_limit` is deprecated, use `iteration_range` instead.
## [15:59:12] WARNING: src/c_api/c_api.cc:935: `ntree_limit` is deprecated, use `iteration_range` instead.
## [15:59:12] WARNING: src/c_api/c_api.cc:935: `ntree_limit` is deprecated, use `iteration_range` instead.
## [15:59:13] WARNING: src/c_api/c_api.cc:935: `ntree_limit` is deprecated, use `iteration_range` instead.
## [15:59:13] WARNING: src/c_api/c_api.cc:935: `ntree_limit` is deprecated, use `iteration_range` instead.
## [15:59:13] WARNING: src/c_api/c_api.cc:935: `ntree_limit` is deprecated, use `iteration_range` instead.
## [15:59:13] WARNING: src/c_api/c_api.cc:935: `ntree_limit` is deprecated, use `iteration_range` instead.
## [15:59:13] WARNING: src/c_api/c_api.cc:935: `ntree_limit` is deprecated, use `iteration_range` instead.
## [15:59:13] WARNING: src/c_api/c_api.cc:935: `ntree_limit` is deprecated, use `iteration_range` instead.
## [15:59:13] WARNING: src/c_api/c_api.cc:935: `ntree_limit` is deprecated, use `iteration_range` instead.
## [15:59:13] WARNING: src/c_api/c_api.cc:935: `ntree_limit` is deprecated, use `iteration_range` instead.
## [15:59:13] WARNING: src/c_api/c_api.cc:935: `ntree_limit` is deprecated, use `iteration_range` instead.
## [15:59:13] WARNING: src/c_api/c_api.cc:935: `ntree_limit` is deprecated, use `iteration_range` instead.
## [15:59:13] WARNING: src/c_api/c_api.cc:935: `ntree_limit` is deprecated, use `iteration_range` instead.
## [15:59:13] WARNING: src/c_api/c_api.cc:935: `ntree_limit` is deprecated, use `iteration_range` instead.
## [15:59:14] WARNING: src/c_api/c_api.cc:935: `ntree_limit` is deprecated, use `iteration_range` instead.
## [15:59:14] WARNING: src/c_api/c_api.cc:935: `ntree_limit` is deprecated, use `iteration_range` instead.
## [15:59:14] WARNING: src/c_api/c_api.cc:935: `ntree_limit` is deprecated, use `iteration_range` instead.
## [15:59:14] WARNING: src/c_api/c_api.cc:935: `ntree_limit` is deprecated, use `iteration_range` instead.
## [15:59:14] WARNING: src/c_api/c_api.cc:935: `ntree_limit` is deprecated, use `iteration_range` instead.
## [15:59:14] WARNING: src/c_api/c_api.cc:935: `ntree_limit` is deprecated, use `iteration_range` instead.
## [15:59:14] WARNING: src/c_api/c_api.cc:935: `ntree_limit` is deprecated, use `iteration_range` instead.
## [15:59:14] WARNING: src/c_api/c_api.cc:935: `ntree_limit` is deprecated, use `iteration_range` instead.
## [15:59:14] WARNING: src/c_api/c_api.cc:935: `ntree_limit` is deprecated, use `iteration_range` instead.
## [15:59:14] WARNING: src/c_api/c_api.cc:935: `ntree_limit` is deprecated, use `iteration_range` instead.
## [15:59:14] WARNING: src/c_api/c_api.cc:935: `ntree_limit` is deprecated, use `iteration_range` instead.
## [15:59:14] WARNING: src/c_api/c_api.cc:935: `ntree_limit` is deprecated, use `iteration_range` instead.
## [15:59:15] WARNING: src/c_api/c_api.cc:935: `ntree_limit` is deprecated, use `iteration_range` instead.
## [15:59:15] WARNING: src/c_api/c_api.cc:935: `ntree_limit` is deprecated, use `iteration_range` instead.
## [15:59:15] WARNING: src/c_api/c_api.cc:935: `ntree_limit` is deprecated, use `iteration_range` instead.
## [15:59:15] WARNING: src/c_api/c_api.cc:935: `ntree_limit` is deprecated, use `iteration_range` instead.
## [15:59:15] WARNING: src/c_api/c_api.cc:935: `ntree_limit` is deprecated, use `iteration_range` instead.
## [15:59:15] WARNING: src/c_api/c_api.cc:935: `ntree_limit` is deprecated, use `iteration_range` instead.
## [15:59:15] WARNING: src/c_api/c_api.cc:935: `ntree_limit` is deprecated, use `iteration_range` instead.
## [15:59:15] WARNING: src/c_api/c_api.cc:935: `ntree_limit` is deprecated, use `iteration_range` instead.
## [15:59:16] WARNING: src/c_api/c_api.cc:935: `ntree_limit` is deprecated, use `iteration_range` instead.
## [15:59:16] WARNING: src/c_api/c_api.cc:935: `ntree_limit` is deprecated, use `iteration_range` instead.
## [15:59:16] WARNING: src/c_api/c_api.cc:935: `ntree_limit` is deprecated, use `iteration_range` instead.
## [15:59:16] WARNING: src/c_api/c_api.cc:935: `ntree_limit` is deprecated, use `iteration_range` instead.
## [15:59:16] WARNING: src/c_api/c_api.cc:935: `ntree_limit` is deprecated, use `iteration_range` instead.
## [15:59:16] WARNING: src/c_api/c_api.cc:935: `ntree_limit` is deprecated, use `iteration_range` instead.
## [15:59:16] WARNING: src/c_api/c_api.cc:935: `ntree_limit` is deprecated, use `iteration_range` instead.
## [15:59:16] WARNING: src/c_api/c_api.cc:935: `ntree_limit` is deprecated, use `iteration_range` instead.
## [15:59:16] WARNING: src/c_api/c_api.cc:935: `ntree_limit` is deprecated, use `iteration_range` instead.
## [15:59:16] WARNING: src/c_api/c_api.cc:935: `ntree_limit` is deprecated, use `iteration_range` instead.
## [15:59:16] WARNING: src/c_api/c_api.cc:935: `ntree_limit` is deprecated, use `iteration_range` instead.
## [15:59:16] WARNING: src/c_api/c_api.cc:935: `ntree_limit` is deprecated, use `iteration_range` instead.
## [15:59:17] WARNING: src/c_api/c_api.cc:935: `ntree_limit` is deprecated, use `iteration_range` instead.
## [15:59:17] WARNING: src/c_api/c_api.cc:935: `ntree_limit` is deprecated, use `iteration_range` instead.
## [15:59:17] WARNING: src/c_api/c_api.cc:935: `ntree_limit` is deprecated, use `iteration_range` instead.
## [15:59:17] WARNING: src/c_api/c_api.cc:935: `ntree_limit` is deprecated, use `iteration_range` instead.
## [15:59:17] WARNING: src/c_api/c_api.cc:935: `ntree_limit` is deprecated, use `iteration_range` instead.
## [15:59:17] WARNING: src/c_api/c_api.cc:935: `ntree_limit` is deprecated, use `iteration_range` instead.
## [15:59:17] WARNING: src/c_api/c_api.cc:935: `ntree_limit` is deprecated, use `iteration_range` instead.
## [15:59:17] WARNING: src/c_api/c_api.cc:935: `ntree_limit` is deprecated, use `iteration_range` instead.
## [15:59:17] WARNING: src/c_api/c_api.cc:935: `ntree_limit` is deprecated, use `iteration_range` instead.
## [15:59:17] WARNING: src/c_api/c_api.cc:935: `ntree_limit` is deprecated, use `iteration_range` instead.
## [15:59:17] WARNING: src/c_api/c_api.cc:935: `ntree_limit` is deprecated, use `iteration_range` instead.
## [15:59:17] WARNING: src/c_api/c_api.cc:935: `ntree_limit` is deprecated, use `iteration_range` instead.
## [15:59:18] WARNING: src/c_api/c_api.cc:935: `ntree_limit` is deprecated, use `iteration_range` instead.
## [15:59:18] WARNING: src/c_api/c_api.cc:935: `ntree_limit` is deprecated, use `iteration_range` instead.
## [15:59:18] WARNING: src/c_api/c_api.cc:935: `ntree_limit` is deprecated, use `iteration_range` instead.
## [15:59:18] WARNING: src/c_api/c_api.cc:935: `ntree_limit` is deprecated, use `iteration_range` instead.
## [15:59:18] WARNING: src/c_api/c_api.cc:935: `ntree_limit` is deprecated, use `iteration_range` instead.
## [15:59:18] WARNING: src/c_api/c_api.cc:935: `ntree_limit` is deprecated, use `iteration_range` instead.
## [15:59:18] WARNING: src/c_api/c_api.cc:935: `ntree_limit` is deprecated, use `iteration_range` instead.
## [15:59:18] WARNING: src/c_api/c_api.cc:935: `ntree_limit` is deprecated, use `iteration_range` instead.
## [15:59:18] WARNING: src/c_api/c_api.cc:935: `ntree_limit` is deprecated, use `iteration_range` instead.
## [15:59:18] WARNING: src/c_api/c_api.cc:935: `ntree_limit` is deprecated, use `iteration_range` instead.
## [15:59:19] WARNING: src/c_api/c_api.cc:935: `ntree_limit` is deprecated, use `iteration_range` instead.
## [15:59:19] WARNING: src/c_api/c_api.cc:935: `ntree_limit` is deprecated, use `iteration_range` instead.
## [15:59:19] WARNING: src/c_api/c_api.cc:935: `ntree_limit` is deprecated, use `iteration_range` instead.
## [15:59:19] WARNING: src/c_api/c_api.cc:935: `ntree_limit` is deprecated, use `iteration_range` instead.
## [15:59:19] WARNING: src/c_api/c_api.cc:935: `ntree_limit` is deprecated, use `iteration_range` instead.
## [15:59:19] WARNING: src/c_api/c_api.cc:935: `ntree_limit` is deprecated, use `iteration_range` instead.
## [15:59:19] WARNING: src/c_api/c_api.cc:935: `ntree_limit` is deprecated, use `iteration_range` instead.
## [15:59:19] WARNING: src/c_api/c_api.cc:935: `ntree_limit` is deprecated, use `iteration_range` instead.
## [15:59:20] WARNING: src/c_api/c_api.cc:935: `ntree_limit` is deprecated, use `iteration_range` instead.
## [15:59:20] WARNING: src/c_api/c_api.cc:935: `ntree_limit` is deprecated, use `iteration_range` instead.
## [15:59:20] WARNING: src/c_api/c_api.cc:935: `ntree_limit` is deprecated, use `iteration_range` instead.
## [15:59:20] WARNING: src/c_api/c_api.cc:935: `ntree_limit` is deprecated, use `iteration_range` instead.
## [15:59:20] WARNING: src/c_api/c_api.cc:935: `ntree_limit` is deprecated, use `iteration_range` instead.
## [15:59:20] WARNING: src/c_api/c_api.cc:935: `ntree_limit` is deprecated, use `iteration_range` instead.
## [15:59:20] WARNING: src/c_api/c_api.cc:935: `ntree_limit` is deprecated, use `iteration_range` instead.
## [15:59:20] WARNING: src/c_api/c_api.cc:935: `ntree_limit` is deprecated, use `iteration_range` instead.
## [15:59:20] WARNING: src/c_api/c_api.cc:935: `ntree_limit` is deprecated, use `iteration_range` instead.
## [15:59:20] WARNING: src/c_api/c_api.cc:935: `ntree_limit` is deprecated, use `iteration_range` instead.
## [15:59:20] WARNING: src/c_api/c_api.cc:935: `ntree_limit` is deprecated, use `iteration_range` instead.
## [15:59:20] WARNING: src/c_api/c_api.cc:935: `ntree_limit` is deprecated, use `iteration_range` instead.
## [15:59:20] WARNING: src/c_api/c_api.cc:935: `ntree_limit` is deprecated, use `iteration_range` instead.
## [15:59:20] WARNING: src/c_api/c_api.cc:935: `ntree_limit` is deprecated, use `iteration_range` instead.
## [15:59:21] WARNING: src/c_api/c_api.cc:935: `ntree_limit` is deprecated, use `iteration_range` instead.
## [15:59:21] WARNING: src/c_api/c_api.cc:935: `ntree_limit` is deprecated, use `iteration_range` instead.
## [15:59:21] WARNING: src/c_api/c_api.cc:935: `ntree_limit` is deprecated, use `iteration_range` instead.
## [15:59:21] WARNING: src/c_api/c_api.cc:935: `ntree_limit` is deprecated, use `iteration_range` instead.
## [15:59:21] WARNING: src/c_api/c_api.cc:935: `ntree_limit` is deprecated, use `iteration_range` instead.
## [15:59:21] WARNING: src/c_api/c_api.cc:935: `ntree_limit` is deprecated, use `iteration_range` instead.
## [15:59:21] WARNING: src/c_api/c_api.cc:935: `ntree_limit` is deprecated, use `iteration_range` instead.
## [15:59:21] WARNING: src/c_api/c_api.cc:935: `ntree_limit` is deprecated, use `iteration_range` instead.
## [15:59:21] WARNING: src/c_api/c_api.cc:935: `ntree_limit` is deprecated, use `iteration_range` instead.
## [15:59:21] WARNING: src/c_api/c_api.cc:935: `ntree_limit` is deprecated, use `iteration_range` instead.
## [15:59:21] WARNING: src/c_api/c_api.cc:935: `ntree_limit` is deprecated, use `iteration_range` instead.
## [15:59:21] WARNING: src/c_api/c_api.cc:935: `ntree_limit` is deprecated, use `iteration_range` instead.
## [15:59:22] WARNING: src/c_api/c_api.cc:935: `ntree_limit` is deprecated, use `iteration_range` instead.
## [15:59:22] WARNING: src/c_api/c_api.cc:935: `ntree_limit` is deprecated, use `iteration_range` instead.
## [15:59:22] WARNING: src/c_api/c_api.cc:935: `ntree_limit` is deprecated, use `iteration_range` instead.
## [15:59:22] WARNING: src/c_api/c_api.cc:935: `ntree_limit` is deprecated, use `iteration_range` instead.
## [15:59:22] WARNING: src/c_api/c_api.cc:935: `ntree_limit` is deprecated, use `iteration_range` instead.
## [15:59:22] WARNING: src/c_api/c_api.cc:935: `ntree_limit` is deprecated, use `iteration_range` instead.
## [15:59:22] WARNING: src/c_api/c_api.cc:935: `ntree_limit` is deprecated, use `iteration_range` instead.
## [15:59:22] WARNING: src/c_api/c_api.cc:935: `ntree_limit` is deprecated, use `iteration_range` instead.
## [15:59:22] WARNING: src/c_api/c_api.cc:935: `ntree_limit` is deprecated, use `iteration_range` instead.
## [15:59:22] WARNING: src/c_api/c_api.cc:935: `ntree_limit` is deprecated, use `iteration_range` instead.
## [15:59:23] WARNING: src/c_api/c_api.cc:935: `ntree_limit` is deprecated, use `iteration_range` instead.
## [15:59:23] WARNING: src/c_api/c_api.cc:935: `ntree_limit` is deprecated, use `iteration_range` instead.
## [15:59:23] WARNING: src/c_api/c_api.cc:935: `ntree_limit` is deprecated, use `iteration_range` instead.
## [15:59:23] WARNING: src/c_api/c_api.cc:935: `ntree_limit` is deprecated, use `iteration_range` instead.
## [15:59:23] WARNING: src/c_api/c_api.cc:935: `ntree_limit` is deprecated, use `iteration_range` instead.
## [15:59:23] WARNING: src/c_api/c_api.cc:935: `ntree_limit` is deprecated, use `iteration_range` instead.
## [15:59:23] WARNING: src/c_api/c_api.cc:935: `ntree_limit` is deprecated, use `iteration_range` instead.
## [15:59:23] WARNING: src/c_api/c_api.cc:935: `ntree_limit` is deprecated, use `iteration_range` instead.
## [15:59:23] WARNING: src/c_api/c_api.cc:935: `ntree_limit` is deprecated, use `iteration_range` instead.
## [15:59:23] WARNING: src/c_api/c_api.cc:935: `ntree_limit` is deprecated, use `iteration_range` instead.
## [15:59:23] WARNING: src/c_api/c_api.cc:935: `ntree_limit` is deprecated, use `iteration_range` instead.
## [15:59:23] WARNING: src/c_api/c_api.cc:935: `ntree_limit` is deprecated, use `iteration_range` instead.
## [15:59:24] WARNING: src/c_api/c_api.cc:935: `ntree_limit` is deprecated, use `iteration_range` instead.
## [15:59:24] WARNING: src/c_api/c_api.cc:935: `ntree_limit` is deprecated, use `iteration_range` instead.
## [15:59:24] WARNING: src/c_api/c_api.cc:935: `ntree_limit` is deprecated, use `iteration_range` instead.
## [15:59:24] WARNING: src/c_api/c_api.cc:935: `ntree_limit` is deprecated, use `iteration_range` instead.
## [15:59:24] WARNING: src/c_api/c_api.cc:935: `ntree_limit` is deprecated, use `iteration_range` instead.
## [15:59:24] WARNING: src/c_api/c_api.cc:935: `ntree_limit` is deprecated, use `iteration_range` instead.
## [15:59:24] WARNING: src/c_api/c_api.cc:935: `ntree_limit` is deprecated, use `iteration_range` instead.
## [15:59:24] WARNING: src/c_api/c_api.cc:935: `ntree_limit` is deprecated, use `iteration_range` instead.
## [15:59:24] WARNING: src/c_api/c_api.cc:935: `ntree_limit` is deprecated, use `iteration_range` instead.
## [15:59:24] WARNING: src/c_api/c_api.cc:935: `ntree_limit` is deprecated, use `iteration_range` instead.
## [15:59:25] WARNING: src/c_api/c_api.cc:935: `ntree_limit` is deprecated, use `iteration_range` instead.
## [15:59:25] WARNING: src/c_api/c_api.cc:935: `ntree_limit` is deprecated, use `iteration_range` instead.
## [15:59:25] WARNING: src/c_api/c_api.cc:935: `ntree_limit` is deprecated, use `iteration_range` instead.
## [15:59:25] WARNING: src/c_api/c_api.cc:935: `ntree_limit` is deprecated, use `iteration_range` instead.
## [15:59:25] WARNING: src/c_api/c_api.cc:935: `ntree_limit` is deprecated, use `iteration_range` instead.
## [15:59:25] WARNING: src/c_api/c_api.cc:935: `ntree_limit` is deprecated, use `iteration_range` instead.
## [15:59:25] WARNING: src/c_api/c_api.cc:935: `ntree_limit` is deprecated, use `iteration_range` instead.
## [15:59:25] WARNING: src/c_api/c_api.cc:935: `ntree_limit` is deprecated, use `iteration_range` instead.
## [15:59:26] WARNING: src/c_api/c_api.cc:935: `ntree_limit` is deprecated, use `iteration_range` instead.
## [15:59:26] WARNING: src/c_api/c_api.cc:935: `ntree_limit` is deprecated, use `iteration_range` instead.
## [15:59:26] WARNING: src/c_api/c_api.cc:935: `ntree_limit` is deprecated, use `iteration_range` instead.
## [15:59:26] WARNING: src/c_api/c_api.cc:935: `ntree_limit` is deprecated, use `iteration_range` instead.
## [15:59:26] WARNING: src/c_api/c_api.cc:935: `ntree_limit` is deprecated, use `iteration_range` instead.
## [15:59:26] WARNING: src/c_api/c_api.cc:935: `ntree_limit` is deprecated, use `iteration_range` instead.
## [15:59:26] WARNING: src/c_api/c_api.cc:935: `ntree_limit` is deprecated, use `iteration_range` instead.
## [15:59:26] WARNING: src/c_api/c_api.cc:935: `ntree_limit` is deprecated, use `iteration_range` instead.
## [15:59:26] WARNING: src/c_api/c_api.cc:935: `ntree_limit` is deprecated, use `iteration_range` instead.
## [15:59:26] WARNING: src/c_api/c_api.cc:935: `ntree_limit` is deprecated, use `iteration_range` instead.
## [15:59:27] WARNING: src/c_api/c_api.cc:935: `ntree_limit` is deprecated, use `iteration_range` instead.
## [15:59:27] WARNING: src/c_api/c_api.cc:935: `ntree_limit` is deprecated, use `iteration_range` instead.
## [15:59:27] WARNING: src/c_api/c_api.cc:935: `ntree_limit` is deprecated, use `iteration_range` instead.
## [15:59:27] WARNING: src/c_api/c_api.cc:935: `ntree_limit` is deprecated, use `iteration_range` instead.
## [15:59:27] WARNING: src/c_api/c_api.cc:935: `ntree_limit` is deprecated, use `iteration_range` instead.
## [15:59:27] WARNING: src/c_api/c_api.cc:935: `ntree_limit` is deprecated, use `iteration_range` instead.
## [15:59:27] WARNING: src/c_api/c_api.cc:935: `ntree_limit` is deprecated, use `iteration_range` instead.
## [15:59:27] WARNING: src/c_api/c_api.cc:935: `ntree_limit` is deprecated, use `iteration_range` instead.
## [15:59:27] WARNING: src/c_api/c_api.cc:935: `ntree_limit` is deprecated, use `iteration_range` instead.
## [15:59:27] WARNING: src/c_api/c_api.cc:935: `ntree_limit` is deprecated, use `iteration_range` instead.
## [15:59:27] WARNING: src/c_api/c_api.cc:935: `ntree_limit` is deprecated, use `iteration_range` instead.
## [15:59:27] WARNING: src/c_api/c_api.cc:935: `ntree_limit` is deprecated, use `iteration_range` instead.
## [15:59:27] WARNING: src/c_api/c_api.cc:935: `ntree_limit` is deprecated, use `iteration_range` instead.
## [15:59:27] WARNING: src/c_api/c_api.cc:935: `ntree_limit` is deprecated, use `iteration_range` instead.
## [15:59:28] WARNING: src/c_api/c_api.cc:935: `ntree_limit` is deprecated, use `iteration_range` instead.
## [15:59:28] WARNING: src/c_api/c_api.cc:935: `ntree_limit` is deprecated, use `iteration_range` instead.
## [15:59:28] WARNING: src/c_api/c_api.cc:935: `ntree_limit` is deprecated, use `iteration_range` instead.
## [15:59:28] WARNING: src/c_api/c_api.cc:935: `ntree_limit` is deprecated, use `iteration_range` instead.
## [15:59:28] WARNING: src/c_api/c_api.cc:935: `ntree_limit` is deprecated, use `iteration_range` instead.
## [15:59:28] WARNING: src/c_api/c_api.cc:935: `ntree_limit` is deprecated, use `iteration_range` instead.
## [15:59:28] WARNING: src/c_api/c_api.cc:935: `ntree_limit` is deprecated, use `iteration_range` instead.
## [15:59:28] WARNING: src/c_api/c_api.cc:935: `ntree_limit` is deprecated, use `iteration_range` instead.
## [15:59:28] WARNING: src/c_api/c_api.cc:935: `ntree_limit` is deprecated, use `iteration_range` instead.
## [15:59:28] WARNING: src/c_api/c_api.cc:935: `ntree_limit` is deprecated, use `iteration_range` instead.
## [15:59:29] WARNING: src/c_api/c_api.cc:935: `ntree_limit` is deprecated, use `iteration_range` instead.
## [15:59:29] WARNING: src/c_api/c_api.cc:935: `ntree_limit` is deprecated, use `iteration_range` instead.
## [15:59:29] WARNING: src/c_api/c_api.cc:935: `ntree_limit` is deprecated, use `iteration_range` instead.
## [15:59:29] WARNING: src/c_api/c_api.cc:935: `ntree_limit` is deprecated, use `iteration_range` instead.
## [15:59:29] WARNING: src/c_api/c_api.cc:935: `ntree_limit` is deprecated, use `iteration_range` instead.
## [15:59:29] WARNING: src/c_api/c_api.cc:935: `ntree_limit` is deprecated, use `iteration_range` instead.
## [15:59:29] WARNING: src/c_api/c_api.cc:935: `ntree_limit` is deprecated, use `iteration_range` instead.
## [15:59:29] WARNING: src/c_api/c_api.cc:935: `ntree_limit` is deprecated, use `iteration_range` instead.
## [15:59:29] WARNING: src/c_api/c_api.cc:935: `ntree_limit` is deprecated, use `iteration_range` instead.
## [15:59:29] WARNING: src/c_api/c_api.cc:935: `ntree_limit` is deprecated, use `iteration_range` instead.
## [15:59:30] WARNING: src/c_api/c_api.cc:935: `ntree_limit` is deprecated, use `iteration_range` instead.
## [15:59:30] WARNING: src/c_api/c_api.cc:935: `ntree_limit` is deprecated, use `iteration_range` instead.
## [15:59:30] WARNING: src/c_api/c_api.cc:935: `ntree_limit` is deprecated, use `iteration_range` instead.
## [15:59:30] WARNING: src/c_api/c_api.cc:935: `ntree_limit` is deprecated, use `iteration_range` instead.
## [15:59:30] WARNING: src/c_api/c_api.cc:935: `ntree_limit` is deprecated, use `iteration_range` instead.
## [15:59:30] WARNING: src/c_api/c_api.cc:935: `ntree_limit` is deprecated, use `iteration_range` instead.
## [15:59:30] WARNING: src/c_api/c_api.cc:935: `ntree_limit` is deprecated, use `iteration_range` instead.
## [15:59:30] WARNING: src/c_api/c_api.cc:935: `ntree_limit` is deprecated, use `iteration_range` instead.
## [15:59:30] WARNING: src/c_api/c_api.cc:935: `ntree_limit` is deprecated, use `iteration_range` instead.
## [15:59:30] WARNING: src/c_api/c_api.cc:935: `ntree_limit` is deprecated, use `iteration_range` instead.
## [15:59:30] WARNING: src/c_api/c_api.cc:935: `ntree_limit` is deprecated, use `iteration_range` instead.
## [15:59:30] WARNING: src/c_api/c_api.cc:935: `ntree_limit` is deprecated, use `iteration_range` instead.
## [15:59:31] WARNING: src/c_api/c_api.cc:935: `ntree_limit` is deprecated, use `iteration_range` instead.
## [15:59:31] WARNING: src/c_api/c_api.cc:935: `ntree_limit` is deprecated, use `iteration_range` instead.
## [15:59:31] WARNING: src/c_api/c_api.cc:935: `ntree_limit` is deprecated, use `iteration_range` instead.
## [15:59:31] WARNING: src/c_api/c_api.cc:935: `ntree_limit` is deprecated, use `iteration_range` instead.
## [15:59:31] WARNING: src/c_api/c_api.cc:935: `ntree_limit` is deprecated, use `iteration_range` instead.
## [15:59:31] WARNING: src/c_api/c_api.cc:935: `ntree_limit` is deprecated, use `iteration_range` instead.
## [15:59:31] WARNING: src/c_api/c_api.cc:935: `ntree_limit` is deprecated, use `iteration_range` instead.
## [15:59:31] WARNING: src/c_api/c_api.cc:935: `ntree_limit` is deprecated, use `iteration_range` instead.
## [15:59:31] WARNING: src/c_api/c_api.cc:935: `ntree_limit` is deprecated, use `iteration_range` instead.
## [15:59:31] WARNING: src/c_api/c_api.cc:935: `ntree_limit` is deprecated, use `iteration_range` instead.
## [15:59:32] WARNING: src/c_api/c_api.cc:935: `ntree_limit` is deprecated, use `iteration_range` instead.
## [15:59:32] WARNING: src/c_api/c_api.cc:935: `ntree_limit` is deprecated, use `iteration_range` instead.
## [15:59:32] WARNING: src/c_api/c_api.cc:935: `ntree_limit` is deprecated, use `iteration_range` instead.
## [15:59:32] WARNING: src/c_api/c_api.cc:935: `ntree_limit` is deprecated, use `iteration_range` instead.
## [15:59:32] WARNING: src/c_api/c_api.cc:935: `ntree_limit` is deprecated, use `iteration_range` instead.
## [15:59:32] WARNING: src/c_api/c_api.cc:935: `ntree_limit` is deprecated, use `iteration_range` instead.
## [15:59:32] WARNING: src/c_api/c_api.cc:935: `ntree_limit` is deprecated, use `iteration_range` instead.
## [15:59:32] WARNING: src/c_api/c_api.cc:935: `ntree_limit` is deprecated, use `iteration_range` instead.
## [15:59:33] WARNING: src/c_api/c_api.cc:935: `ntree_limit` is deprecated, use `iteration_range` instead.
## [15:59:33] WARNING: src/c_api/c_api.cc:935: `ntree_limit` is deprecated, use `iteration_range` instead.
## [15:59:33] WARNING: src/c_api/c_api.cc:935: `ntree_limit` is deprecated, use `iteration_range` instead.
## [15:59:33] WARNING: src/c_api/c_api.cc:935: `ntree_limit` is deprecated, use `iteration_range` instead.
## [15:59:33] WARNING: src/c_api/c_api.cc:935: `ntree_limit` is deprecated, use `iteration_range` instead.
## [15:59:33] WARNING: src/c_api/c_api.cc:935: `ntree_limit` is deprecated, use `iteration_range` instead.
## [15:59:33] WARNING: src/c_api/c_api.cc:935: `ntree_limit` is deprecated, use `iteration_range` instead.
## [15:59:33] WARNING: src/c_api/c_api.cc:935: `ntree_limit` is deprecated, use `iteration_range` instead.
## [15:59:33] WARNING: src/c_api/c_api.cc:935: `ntree_limit` is deprecated, use `iteration_range` instead.
## [15:59:33] WARNING: src/c_api/c_api.cc:935: `ntree_limit` is deprecated, use `iteration_range` instead.
## [15:59:33] WARNING: src/c_api/c_api.cc:935: `ntree_limit` is deprecated, use `iteration_range` instead.
## [15:59:33] WARNING: src/c_api/c_api.cc:935: `ntree_limit` is deprecated, use `iteration_range` instead.
## [15:59:34] WARNING: src/c_api/c_api.cc:935: `ntree_limit` is deprecated, use `iteration_range` instead.
## [15:59:34] WARNING: src/c_api/c_api.cc:935: `ntree_limit` is deprecated, use `iteration_range` instead.
## [15:59:34] WARNING: src/c_api/c_api.cc:935: `ntree_limit` is deprecated, use `iteration_range` instead.
## [15:59:34] WARNING: src/c_api/c_api.cc:935: `ntree_limit` is deprecated, use `iteration_range` instead.
## [15:59:34] WARNING: src/c_api/c_api.cc:935: `ntree_limit` is deprecated, use `iteration_range` instead.
## [15:59:34] WARNING: src/c_api/c_api.cc:935: `ntree_limit` is deprecated, use `iteration_range` instead.
## [15:59:34] WARNING: src/c_api/c_api.cc:935: `ntree_limit` is deprecated, use `iteration_range` instead.
## [15:59:34] WARNING: src/c_api/c_api.cc:935: `ntree_limit` is deprecated, use `iteration_range` instead.
## [15:59:34] WARNING: src/c_api/c_api.cc:935: `ntree_limit` is deprecated, use `iteration_range` instead.
## [15:59:34] WARNING: src/c_api/c_api.cc:935: `ntree_limit` is deprecated, use `iteration_range` instead.
## [15:59:34] WARNING: src/c_api/c_api.cc:935: `ntree_limit` is deprecated, use `iteration_range` instead.
## [15:59:34] WARNING: src/c_api/c_api.cc:935: `ntree_limit` is deprecated, use `iteration_range` instead.
## [15:59:34] WARNING: src/c_api/c_api.cc:935: `ntree_limit` is deprecated, use `iteration_range` instead.
## [15:59:34] WARNING: src/c_api/c_api.cc:935: `ntree_limit` is deprecated, use `iteration_range` instead.
## [15:59:35] WARNING: src/c_api/c_api.cc:935: `ntree_limit` is deprecated, use `iteration_range` instead.
## [15:59:35] WARNING: src/c_api/c_api.cc:935: `ntree_limit` is deprecated, use `iteration_range` instead.
## [15:59:35] WARNING: src/c_api/c_api.cc:935: `ntree_limit` is deprecated, use `iteration_range` instead.
## [15:59:35] WARNING: src/c_api/c_api.cc:935: `ntree_limit` is deprecated, use `iteration_range` instead.
## [15:59:35] WARNING: src/c_api/c_api.cc:935: `ntree_limit` is deprecated, use `iteration_range` instead.
## [15:59:35] WARNING: src/c_api/c_api.cc:935: `ntree_limit` is deprecated, use `iteration_range` instead.
## [15:59:35] WARNING: src/c_api/c_api.cc:935: `ntree_limit` is deprecated, use `iteration_range` instead.
## [15:59:35] WARNING: src/c_api/c_api.cc:935: `ntree_limit` is deprecated, use `iteration_range` instead.
## [15:59:35] WARNING: src/c_api/c_api.cc:935: `ntree_limit` is deprecated, use `iteration_range` instead.
## [15:59:35] WARNING: src/c_api/c_api.cc:935: `ntree_limit` is deprecated, use `iteration_range` instead.
## [15:59:36] WARNING: src/c_api/c_api.cc:935: `ntree_limit` is deprecated, use `iteration_range` instead.
## [15:59:36] WARNING: src/c_api/c_api.cc:935: `ntree_limit` is deprecated, use `iteration_range` instead.
## [15:59:36] WARNING: src/c_api/c_api.cc:935: `ntree_limit` is deprecated, use `iteration_range` instead.
## [15:59:36] WARNING: src/c_api/c_api.cc:935: `ntree_limit` is deprecated, use `iteration_range` instead.
## [15:59:36] WARNING: src/c_api/c_api.cc:935: `ntree_limit` is deprecated, use `iteration_range` instead.
## [15:59:36] WARNING: src/c_api/c_api.cc:935: `ntree_limit` is deprecated, use `iteration_range` instead.
## [15:59:39] WARNING: src/c_api/c_api.cc:935: `ntree_limit` is deprecated, use `iteration_range` instead.
## [15:59:39] WARNING: src/c_api/c_api.cc:935: `ntree_limit` is deprecated, use `iteration_range` instead.
## [15:59:39] WARNING: src/c_api/c_api.cc:935: `ntree_limit` is deprecated, use `iteration_range` instead.
## [15:59:39] WARNING: src/c_api/c_api.cc:935: `ntree_limit` is deprecated, use `iteration_range` instead.
## [15:59:39] WARNING: src/c_api/c_api.cc:935: `ntree_limit` is deprecated, use `iteration_range` instead.
## [15:59:39] WARNING: src/c_api/c_api.cc:935: `ntree_limit` is deprecated, use `iteration_range` instead.
## [15:59:39] WARNING: src/c_api/c_api.cc:935: `ntree_limit` is deprecated, use `iteration_range` instead.
## [15:59:39] WARNING: src/c_api/c_api.cc:935: `ntree_limit` is deprecated, use `iteration_range` instead.
## [15:59:39] WARNING: src/c_api/c_api.cc:935: `ntree_limit` is deprecated, use `iteration_range` instead.
## [15:59:39] WARNING: src/c_api/c_api.cc:935: `ntree_limit` is deprecated, use `iteration_range` instead.
## [15:59:40] WARNING: src/c_api/c_api.cc:935: `ntree_limit` is deprecated, use `iteration_range` instead.
## [15:59:40] WARNING: src/c_api/c_api.cc:935: `ntree_limit` is deprecated, use `iteration_range` instead.
## [15:59:40] WARNING: src/c_api/c_api.cc:935: `ntree_limit` is deprecated, use `iteration_range` instead.
## [15:59:40] WARNING: src/c_api/c_api.cc:935: `ntree_limit` is deprecated, use `iteration_range` instead.
## [15:59:40] WARNING: src/c_api/c_api.cc:935: `ntree_limit` is deprecated, use `iteration_range` instead.
## [15:59:40] WARNING: src/c_api/c_api.cc:935: `ntree_limit` is deprecated, use `iteration_range` instead.
## [15:59:40] WARNING: src/c_api/c_api.cc:935: `ntree_limit` is deprecated, use `iteration_range` instead.
## [15:59:40] WARNING: src/c_api/c_api.cc:935: `ntree_limit` is deprecated, use `iteration_range` instead.
## [15:59:40] WARNING: src/c_api/c_api.cc:935: `ntree_limit` is deprecated, use `iteration_range` instead.
## [15:59:40] WARNING: src/c_api/c_api.cc:935: `ntree_limit` is deprecated, use `iteration_range` instead.
## [15:59:41] WARNING: src/c_api/c_api.cc:935: `ntree_limit` is deprecated, use `iteration_range` instead.
## [15:59:41] WARNING: src/c_api/c_api.cc:935: `ntree_limit` is deprecated, use `iteration_range` instead.
## [15:59:41] WARNING: src/c_api/c_api.cc:935: `ntree_limit` is deprecated, use `iteration_range` instead.
## [15:59:41] WARNING: src/c_api/c_api.cc:935: `ntree_limit` is deprecated, use `iteration_range` instead.
## [15:59:41] WARNING: src/c_api/c_api.cc:935: `ntree_limit` is deprecated, use `iteration_range` instead.
## [15:59:41] WARNING: src/c_api/c_api.cc:935: `ntree_limit` is deprecated, use `iteration_range` instead.
## [15:59:41] WARNING: src/c_api/c_api.cc:935: `ntree_limit` is deprecated, use `iteration_range` instead.
## [15:59:41] WARNING: src/c_api/c_api.cc:935: `ntree_limit` is deprecated, use `iteration_range` instead.
## [15:59:41] WARNING: src/c_api/c_api.cc:935: `ntree_limit` is deprecated, use `iteration_range` instead.
## [15:59:41] WARNING: src/c_api/c_api.cc:935: `ntree_limit` is deprecated, use `iteration_range` instead.
## [15:59:42] WARNING: src/c_api/c_api.cc:935: `ntree_limit` is deprecated, use `iteration_range` instead.
## [15:59:42] WARNING: src/c_api/c_api.cc:935: `ntree_limit` is deprecated, use `iteration_range` instead.
## [15:59:42] WARNING: src/c_api/c_api.cc:935: `ntree_limit` is deprecated, use `iteration_range` instead.
## [15:59:42] WARNING: src/c_api/c_api.cc:935: `ntree_limit` is deprecated, use `iteration_range` instead.
## [15:59:42] WARNING: src/c_api/c_api.cc:935: `ntree_limit` is deprecated, use `iteration_range` instead.
## [15:59:42] WARNING: src/c_api/c_api.cc:935: `ntree_limit` is deprecated, use `iteration_range` instead.
## [15:59:42] WARNING: src/c_api/c_api.cc:935: `ntree_limit` is deprecated, use `iteration_range` instead.
## [15:59:42] WARNING: src/c_api/c_api.cc:935: `ntree_limit` is deprecated, use `iteration_range` instead.
## [15:59:42] WARNING: src/c_api/c_api.cc:935: `ntree_limit` is deprecated, use `iteration_range` instead.
## [15:59:42] WARNING: src/c_api/c_api.cc:935: `ntree_limit` is deprecated, use `iteration_range` instead.
## [15:59:43] WARNING: src/c_api/c_api.cc:935: `ntree_limit` is deprecated, use `iteration_range` instead.
## [15:59:43] WARNING: src/c_api/c_api.cc:935: `ntree_limit` is deprecated, use `iteration_range` instead.
## [15:59:43] WARNING: src/c_api/c_api.cc:935: `ntree_limit` is deprecated, use `iteration_range` instead.
## [15:59:43] WARNING: src/c_api/c_api.cc:935: `ntree_limit` is deprecated, use `iteration_range` instead.
## [15:59:43] WARNING: src/c_api/c_api.cc:935: `ntree_limit` is deprecated, use `iteration_range` instead.
## [15:59:43] WARNING: src/c_api/c_api.cc:935: `ntree_limit` is deprecated, use `iteration_range` instead.
## [15:59:43] WARNING: src/c_api/c_api.cc:935: `ntree_limit` is deprecated, use `iteration_range` instead.
## [15:59:43] WARNING: src/c_api/c_api.cc:935: `ntree_limit` is deprecated, use `iteration_range` instead.
## [15:59:43] WARNING: src/c_api/c_api.cc:935: `ntree_limit` is deprecated, use `iteration_range` instead.
## [15:59:43] WARNING: src/c_api/c_api.cc:935: `ntree_limit` is deprecated, use `iteration_range` instead.
## [15:59:43] WARNING: src/c_api/c_api.cc:935: `ntree_limit` is deprecated, use `iteration_range` instead.
## [15:59:43] WARNING: src/c_api/c_api.cc:935: `ntree_limit` is deprecated, use `iteration_range` instead.
## [15:59:43] WARNING: src/c_api/c_api.cc:935: `ntree_limit` is deprecated, use `iteration_range` instead.
## [15:59:43] WARNING: src/c_api/c_api.cc:935: `ntree_limit` is deprecated, use `iteration_range` instead.
## [15:59:44] WARNING: src/c_api/c_api.cc:935: `ntree_limit` is deprecated, use `iteration_range` instead.
## [15:59:44] WARNING: src/c_api/c_api.cc:935: `ntree_limit` is deprecated, use `iteration_range` instead.
## [15:59:44] WARNING: src/c_api/c_api.cc:935: `ntree_limit` is deprecated, use `iteration_range` instead.
## [15:59:44] WARNING: src/c_api/c_api.cc:935: `ntree_limit` is deprecated, use `iteration_range` instead.
## [15:59:44] WARNING: src/c_api/c_api.cc:935: `ntree_limit` is deprecated, use `iteration_range` instead.
## [15:59:44] WARNING: src/c_api/c_api.cc:935: `ntree_limit` is deprecated, use `iteration_range` instead.
## [15:59:44] WARNING: src/c_api/c_api.cc:935: `ntree_limit` is deprecated, use `iteration_range` instead.
## [15:59:44] WARNING: src/c_api/c_api.cc:935: `ntree_limit` is deprecated, use `iteration_range` instead.
## [15:59:44] WARNING: src/c_api/c_api.cc:935: `ntree_limit` is deprecated, use `iteration_range` instead.
## [15:59:44] WARNING: src/c_api/c_api.cc:935: `ntree_limit` is deprecated, use `iteration_range` instead.
## [15:59:45] WARNING: src/c_api/c_api.cc:935: `ntree_limit` is deprecated, use `iteration_range` instead.
## [15:59:45] WARNING: src/c_api/c_api.cc:935: `ntree_limit` is deprecated, use `iteration_range` instead.
## [15:59:45] WARNING: src/c_api/c_api.cc:935: `ntree_limit` is deprecated, use `iteration_range` instead.
## [15:59:45] WARNING: src/c_api/c_api.cc:935: `ntree_limit` is deprecated, use `iteration_range` instead.
## [15:59:45] WARNING: src/c_api/c_api.cc:935: `ntree_limit` is deprecated, use `iteration_range` instead.
## [15:59:45] WARNING: src/c_api/c_api.cc:935: `ntree_limit` is deprecated, use `iteration_range` instead.
## [15:59:45] WARNING: src/c_api/c_api.cc:935: `ntree_limit` is deprecated, use `iteration_range` instead.
## [15:59:45] WARNING: src/c_api/c_api.cc:935: `ntree_limit` is deprecated, use `iteration_range` instead.
## [15:59:45] WARNING: src/c_api/c_api.cc:935: `ntree_limit` is deprecated, use `iteration_range` instead.
## [15:59:45] WARNING: src/c_api/c_api.cc:935: `ntree_limit` is deprecated, use `iteration_range` instead.
## [15:59:46] WARNING: src/c_api/c_api.cc:935: `ntree_limit` is deprecated, use `iteration_range` instead.
## [15:59:46] WARNING: src/c_api/c_api.cc:935: `ntree_limit` is deprecated, use `iteration_range` instead.
## [15:59:46] WARNING: src/c_api/c_api.cc:935: `ntree_limit` is deprecated, use `iteration_range` instead.
## [15:59:46] WARNING: src/c_api/c_api.cc:935: `ntree_limit` is deprecated, use `iteration_range` instead.
## [15:59:46] WARNING: src/c_api/c_api.cc:935: `ntree_limit` is deprecated, use `iteration_range` instead.
## [15:59:46] WARNING: src/c_api/c_api.cc:935: `ntree_limit` is deprecated, use `iteration_range` instead.
## [15:59:46] WARNING: src/c_api/c_api.cc:935: `ntree_limit` is deprecated, use `iteration_range` instead.
## [15:59:46] WARNING: src/c_api/c_api.cc:935: `ntree_limit` is deprecated, use `iteration_range` instead.
## [15:59:46] WARNING: src/c_api/c_api.cc:935: `ntree_limit` is deprecated, use `iteration_range` instead.
## [15:59:46] WARNING: src/c_api/c_api.cc:935: `ntree_limit` is deprecated, use `iteration_range` instead.
## [15:59:46] WARNING: src/c_api/c_api.cc:935: `ntree_limit` is deprecated, use `iteration_range` instead.
## [15:59:46] WARNING: src/c_api/c_api.cc:935: `ntree_limit` is deprecated, use `iteration_range` instead.
## [15:59:46] WARNING: src/c_api/c_api.cc:935: `ntree_limit` is deprecated, use `iteration_range` instead.
## [15:59:46] WARNING: src/c_api/c_api.cc:935: `ntree_limit` is deprecated, use `iteration_range` instead.
## [15:59:47] WARNING: src/c_api/c_api.cc:935: `ntree_limit` is deprecated, use `iteration_range` instead.
## [15:59:47] WARNING: src/c_api/c_api.cc:935: `ntree_limit` is deprecated, use `iteration_range` instead.
## [15:59:47] WARNING: src/c_api/c_api.cc:935: `ntree_limit` is deprecated, use `iteration_range` instead.
## [15:59:47] WARNING: src/c_api/c_api.cc:935: `ntree_limit` is deprecated, use `iteration_range` instead.
## [15:59:47] WARNING: src/c_api/c_api.cc:935: `ntree_limit` is deprecated, use `iteration_range` instead.
## [15:59:47] WARNING: src/c_api/c_api.cc:935: `ntree_limit` is deprecated, use `iteration_range` instead.
## [15:59:47] WARNING: src/c_api/c_api.cc:935: `ntree_limit` is deprecated, use `iteration_range` instead.
## [15:59:47] WARNING: src/c_api/c_api.cc:935: `ntree_limit` is deprecated, use `iteration_range` instead.
## [15:59:48] WARNING: src/c_api/c_api.cc:935: `ntree_limit` is deprecated, use `iteration_range` instead.
## [15:59:48] WARNING: src/c_api/c_api.cc:935: `ntree_limit` is deprecated, use `iteration_range` instead.
## [15:59:48] WARNING: src/c_api/c_api.cc:935: `ntree_limit` is deprecated, use `iteration_range` instead.
## [15:59:48] WARNING: src/c_api/c_api.cc:935: `ntree_limit` is deprecated, use `iteration_range` instead.
## [15:59:48] WARNING: src/c_api/c_api.cc:935: `ntree_limit` is deprecated, use `iteration_range` instead.
## [15:59:48] WARNING: src/c_api/c_api.cc:935: `ntree_limit` is deprecated, use `iteration_range` instead.
## [15:59:48] WARNING: src/c_api/c_api.cc:935: `ntree_limit` is deprecated, use `iteration_range` instead.
## [15:59:48] WARNING: src/c_api/c_api.cc:935: `ntree_limit` is deprecated, use `iteration_range` instead.
## [15:59:48] WARNING: src/c_api/c_api.cc:935: `ntree_limit` is deprecated, use `iteration_range` instead.
## [15:59:48] WARNING: src/c_api/c_api.cc:935: `ntree_limit` is deprecated, use `iteration_range` instead.
## [15:59:49] WARNING: src/c_api/c_api.cc:935: `ntree_limit` is deprecated, use `iteration_range` instead.
## [15:59:49] WARNING: src/c_api/c_api.cc:935: `ntree_limit` is deprecated, use `iteration_range` instead.
## [15:59:49] WARNING: src/c_api/c_api.cc:935: `ntree_limit` is deprecated, use `iteration_range` instead.
## [15:59:49] WARNING: src/c_api/c_api.cc:935: `ntree_limit` is deprecated, use `iteration_range` instead.
## [15:59:49] WARNING: src/c_api/c_api.cc:935: `ntree_limit` is deprecated, use `iteration_range` instead.
## [15:59:49] WARNING: src/c_api/c_api.cc:935: `ntree_limit` is deprecated, use `iteration_range` instead.
## [15:59:49] WARNING: src/c_api/c_api.cc:935: `ntree_limit` is deprecated, use `iteration_range` instead.
## [15:59:49] WARNING: src/c_api/c_api.cc:935: `ntree_limit` is deprecated, use `iteration_range` instead.
## [15:59:49] WARNING: src/c_api/c_api.cc:935: `ntree_limit` is deprecated, use `iteration_range` instead.
## [15:59:49] WARNING: src/c_api/c_api.cc:935: `ntree_limit` is deprecated, use `iteration_range` instead.
## [15:59:49] WARNING: src/c_api/c_api.cc:935: `ntree_limit` is deprecated, use `iteration_range` instead.
## [15:59:49] WARNING: src/c_api/c_api.cc:935: `ntree_limit` is deprecated, use `iteration_range` instead.
## [15:59:50] WARNING: src/c_api/c_api.cc:935: `ntree_limit` is deprecated, use `iteration_range` instead.
## [15:59:50] WARNING: src/c_api/c_api.cc:935: `ntree_limit` is deprecated, use `iteration_range` instead.
## [15:59:50] WARNING: src/c_api/c_api.cc:935: `ntree_limit` is deprecated, use `iteration_range` instead.
## [15:59:50] WARNING: src/c_api/c_api.cc:935: `ntree_limit` is deprecated, use `iteration_range` instead.
## [15:59:50] WARNING: src/c_api/c_api.cc:935: `ntree_limit` is deprecated, use `iteration_range` instead.
## [15:59:50] WARNING: src/c_api/c_api.cc:935: `ntree_limit` is deprecated, use `iteration_range` instead.
## [15:59:50] WARNING: src/c_api/c_api.cc:935: `ntree_limit` is deprecated, use `iteration_range` instead.
## [15:59:50] WARNING: src/c_api/c_api.cc:935: `ntree_limit` is deprecated, use `iteration_range` instead.
## [15:59:50] WARNING: src/c_api/c_api.cc:935: `ntree_limit` is deprecated, use `iteration_range` instead.
## [15:59:50] WARNING: src/c_api/c_api.cc:935: `ntree_limit` is deprecated, use `iteration_range` instead.
## [15:59:50] WARNING: src/c_api/c_api.cc:935: `ntree_limit` is deprecated, use `iteration_range` instead.
## [15:59:50] WARNING: src/c_api/c_api.cc:935: `ntree_limit` is deprecated, use `iteration_range` instead.
## [15:59:51] WARNING: src/c_api/c_api.cc:935: `ntree_limit` is deprecated, use `iteration_range` instead.
## [15:59:51] WARNING: src/c_api/c_api.cc:935: `ntree_limit` is deprecated, use `iteration_range` instead.
## [15:59:51] WARNING: src/c_api/c_api.cc:935: `ntree_limit` is deprecated, use `iteration_range` instead.
## [15:59:51] WARNING: src/c_api/c_api.cc:935: `ntree_limit` is deprecated, use `iteration_range` instead.
## [15:59:51] WARNING: src/c_api/c_api.cc:935: `ntree_limit` is deprecated, use `iteration_range` instead.
## [15:59:51] WARNING: src/c_api/c_api.cc:935: `ntree_limit` is deprecated, use `iteration_range` instead.
## [15:59:51] WARNING: src/c_api/c_api.cc:935: `ntree_limit` is deprecated, use `iteration_range` instead.
## [15:59:51] WARNING: src/c_api/c_api.cc:935: `ntree_limit` is deprecated, use `iteration_range` instead.
## [15:59:51] WARNING: src/c_api/c_api.cc:935: `ntree_limit` is deprecated, use `iteration_range` instead.
## [15:59:51] WARNING: src/c_api/c_api.cc:935: `ntree_limit` is deprecated, use `iteration_range` instead.
## [15:59:52] WARNING: src/c_api/c_api.cc:935: `ntree_limit` is deprecated, use `iteration_range` instead.
## [15:59:52] WARNING: src/c_api/c_api.cc:935: `ntree_limit` is deprecated, use `iteration_range` instead.
## [15:59:52] WARNING: src/c_api/c_api.cc:935: `ntree_limit` is deprecated, use `iteration_range` instead.
## [15:59:52] WARNING: src/c_api/c_api.cc:935: `ntree_limit` is deprecated, use `iteration_range` instead.
## [15:59:52] WARNING: src/c_api/c_api.cc:935: `ntree_limit` is deprecated, use `iteration_range` instead.
## [15:59:52] WARNING: src/c_api/c_api.cc:935: `ntree_limit` is deprecated, use `iteration_range` instead.
## [15:59:52] WARNING: src/c_api/c_api.cc:935: `ntree_limit` is deprecated, use `iteration_range` instead.
## [15:59:52] WARNING: src/c_api/c_api.cc:935: `ntree_limit` is deprecated, use `iteration_range` instead.
## [15:59:52] WARNING: src/c_api/c_api.cc:935: `ntree_limit` is deprecated, use `iteration_range` instead.
## [15:59:52] WARNING: src/c_api/c_api.cc:935: `ntree_limit` is deprecated, use `iteration_range` instead.
## [15:59:52] WARNING: src/c_api/c_api.cc:935: `ntree_limit` is deprecated, use `iteration_range` instead.
## [15:59:52] WARNING: src/c_api/c_api.cc:935: `ntree_limit` is deprecated, use `iteration_range` instead.
## [15:59:53] WARNING: src/c_api/c_api.cc:935: `ntree_limit` is deprecated, use `iteration_range` instead.
## [15:59:53] WARNING: src/c_api/c_api.cc:935: `ntree_limit` is deprecated, use `iteration_range` instead.
## [15:59:53] WARNING: src/c_api/c_api.cc:935: `ntree_limit` is deprecated, use `iteration_range` instead.
## [15:59:53] WARNING: src/c_api/c_api.cc:935: `ntree_limit` is deprecated, use `iteration_range` instead.
## [15:59:53] WARNING: src/c_api/c_api.cc:935: `ntree_limit` is deprecated, use `iteration_range` instead.
## [15:59:53] WARNING: src/c_api/c_api.cc:935: `ntree_limit` is deprecated, use `iteration_range` instead.
## [15:59:53] WARNING: src/c_api/c_api.cc:935: `ntree_limit` is deprecated, use `iteration_range` instead.
## [15:59:53] WARNING: src/c_api/c_api.cc:935: `ntree_limit` is deprecated, use `iteration_range` instead.
## [15:59:53] WARNING: src/c_api/c_api.cc:935: `ntree_limit` is deprecated, use `iteration_range` instead.
## [15:59:53] WARNING: src/c_api/c_api.cc:935: `ntree_limit` is deprecated, use `iteration_range` instead.
## [15:59:53] WARNING: src/c_api/c_api.cc:935: `ntree_limit` is deprecated, use `iteration_range` instead.
## [15:59:53] WARNING: src/c_api/c_api.cc:935: `ntree_limit` is deprecated, use `iteration_range` instead.
## [15:59:54] WARNING: src/c_api/c_api.cc:935: `ntree_limit` is deprecated, use `iteration_range` instead.
## [15:59:54] WARNING: src/c_api/c_api.cc:935: `ntree_limit` is deprecated, use `iteration_range` instead.
## [15:59:54] WARNING: src/c_api/c_api.cc:935: `ntree_limit` is deprecated, use `iteration_range` instead.
## [15:59:54] WARNING: src/c_api/c_api.cc:935: `ntree_limit` is deprecated, use `iteration_range` instead.
## [15:59:54] WARNING: src/c_api/c_api.cc:935: `ntree_limit` is deprecated, use `iteration_range` instead.
## [15:59:54] WARNING: src/c_api/c_api.cc:935: `ntree_limit` is deprecated, use `iteration_range` instead.
## [15:59:54] WARNING: src/c_api/c_api.cc:935: `ntree_limit` is deprecated, use `iteration_range` instead.
## [15:59:54] WARNING: src/c_api/c_api.cc:935: `ntree_limit` is deprecated, use `iteration_range` instead.
## [15:59:55] WARNING: src/c_api/c_api.cc:935: `ntree_limit` is deprecated, use `iteration_range` instead.
## [15:59:55] WARNING: src/c_api/c_api.cc:935: `ntree_limit` is deprecated, use `iteration_range` instead.
## [15:59:55] WARNING: src/c_api/c_api.cc:935: `ntree_limit` is deprecated, use `iteration_range` instead.
## [15:59:55] WARNING: src/c_api/c_api.cc:935: `ntree_limit` is deprecated, use `iteration_range` instead.
## [15:59:55] WARNING: src/c_api/c_api.cc:935: `ntree_limit` is deprecated, use `iteration_range` instead.
## [15:59:55] WARNING: src/c_api/c_api.cc:935: `ntree_limit` is deprecated, use `iteration_range` instead.
## [15:59:55] WARNING: src/c_api/c_api.cc:935: `ntree_limit` is deprecated, use `iteration_range` instead.
## [15:59:55] WARNING: src/c_api/c_api.cc:935: `ntree_limit` is deprecated, use `iteration_range` instead.
## [15:59:55] WARNING: src/c_api/c_api.cc:935: `ntree_limit` is deprecated, use `iteration_range` instead.
## [15:59:55] WARNING: src/c_api/c_api.cc:935: `ntree_limit` is deprecated, use `iteration_range` instead.
## [15:59:56] WARNING: src/c_api/c_api.cc:935: `ntree_limit` is deprecated, use `iteration_range` instead.
## [15:59:56] WARNING: src/c_api/c_api.cc:935: `ntree_limit` is deprecated, use `iteration_range` instead.
## [15:59:56] WARNING: src/c_api/c_api.cc:935: `ntree_limit` is deprecated, use `iteration_range` instead.
## [15:59:56] WARNING: src/c_api/c_api.cc:935: `ntree_limit` is deprecated, use `iteration_range` instead.
## [15:59:56] WARNING: src/c_api/c_api.cc:935: `ntree_limit` is deprecated, use `iteration_range` instead.
## [15:59:56] WARNING: src/c_api/c_api.cc:935: `ntree_limit` is deprecated, use `iteration_range` instead.
## [15:59:56] WARNING: src/c_api/c_api.cc:935: `ntree_limit` is deprecated, use `iteration_range` instead.
## [15:59:56] WARNING: src/c_api/c_api.cc:935: `ntree_limit` is deprecated, use `iteration_range` instead.
## [15:59:56] WARNING: src/c_api/c_api.cc:935: `ntree_limit` is deprecated, use `iteration_range` instead.
## [15:59:56] WARNING: src/c_api/c_api.cc:935: `ntree_limit` is deprecated, use `iteration_range` instead.
## [15:59:56] WARNING: src/c_api/c_api.cc:935: `ntree_limit` is deprecated, use `iteration_range` instead.
## [15:59:56] WARNING: src/c_api/c_api.cc:935: `ntree_limit` is deprecated, use `iteration_range` instead.
## [15:59:56] WARNING: src/c_api/c_api.cc:935: `ntree_limit` is deprecated, use `iteration_range` instead.
## [15:59:56] WARNING: src/c_api/c_api.cc:935: `ntree_limit` is deprecated, use `iteration_range` instead.
## [15:59:57] WARNING: src/c_api/c_api.cc:935: `ntree_limit` is deprecated, use `iteration_range` instead.
## [15:59:57] WARNING: src/c_api/c_api.cc:935: `ntree_limit` is deprecated, use `iteration_range` instead.
## [15:59:57] WARNING: src/c_api/c_api.cc:935: `ntree_limit` is deprecated, use `iteration_range` instead.
## [15:59:57] WARNING: src/c_api/c_api.cc:935: `ntree_limit` is deprecated, use `iteration_range` instead.
## [15:59:57] WARNING: src/c_api/c_api.cc:935: `ntree_limit` is deprecated, use `iteration_range` instead.
## [15:59:57] WARNING: src/c_api/c_api.cc:935: `ntree_limit` is deprecated, use `iteration_range` instead.
## [15:59:57] WARNING: src/c_api/c_api.cc:935: `ntree_limit` is deprecated, use `iteration_range` instead.
## [15:59:57] WARNING: src/c_api/c_api.cc:935: `ntree_limit` is deprecated, use `iteration_range` instead.
## [15:59:57] WARNING: src/c_api/c_api.cc:935: `ntree_limit` is deprecated, use `iteration_range` instead.
## [15:59:57] WARNING: src/c_api/c_api.cc:935: `ntree_limit` is deprecated, use `iteration_range` instead.
## [15:59:58] WARNING: src/c_api/c_api.cc:935: `ntree_limit` is deprecated, use `iteration_range` instead.
## [15:59:58] WARNING: src/c_api/c_api.cc:935: `ntree_limit` is deprecated, use `iteration_range` instead.
## [15:59:58] WARNING: src/c_api/c_api.cc:935: `ntree_limit` is deprecated, use `iteration_range` instead.
## [15:59:58] WARNING: src/c_api/c_api.cc:935: `ntree_limit` is deprecated, use `iteration_range` instead.
## [15:59:58] WARNING: src/c_api/c_api.cc:935: `ntree_limit` is deprecated, use `iteration_range` instead.
## [15:59:58] WARNING: src/c_api/c_api.cc:935: `ntree_limit` is deprecated, use `iteration_range` instead.
## [15:59:58] WARNING: src/c_api/c_api.cc:935: `ntree_limit` is deprecated, use `iteration_range` instead.
## [15:59:58] WARNING: src/c_api/c_api.cc:935: `ntree_limit` is deprecated, use `iteration_range` instead.
## [15:59:58] WARNING: src/c_api/c_api.cc:935: `ntree_limit` is deprecated, use `iteration_range` instead.
## [15:59:58] WARNING: src/c_api/c_api.cc:935: `ntree_limit` is deprecated, use `iteration_range` instead.
## [15:59:59] WARNING: src/c_api/c_api.cc:935: `ntree_limit` is deprecated, use `iteration_range` instead.
## [15:59:59] WARNING: src/c_api/c_api.cc:935: `ntree_limit` is deprecated, use `iteration_range` instead.
## [15:59:59] WARNING: src/c_api/c_api.cc:935: `ntree_limit` is deprecated, use `iteration_range` instead.
## [15:59:59] WARNING: src/c_api/c_api.cc:935: `ntree_limit` is deprecated, use `iteration_range` instead.
## [15:59:59] WARNING: src/c_api/c_api.cc:935: `ntree_limit` is deprecated, use `iteration_range` instead.
## [15:59:59] WARNING: src/c_api/c_api.cc:935: `ntree_limit` is deprecated, use `iteration_range` instead.
## [15:59:59] WARNING: src/c_api/c_api.cc:935: `ntree_limit` is deprecated, use `iteration_range` instead.
## [15:59:59] WARNING: src/c_api/c_api.cc:935: `ntree_limit` is deprecated, use `iteration_range` instead.
## [15:59:59] WARNING: src/c_api/c_api.cc:935: `ntree_limit` is deprecated, use `iteration_range` instead.
## [15:59:59] WARNING: src/c_api/c_api.cc:935: `ntree_limit` is deprecated, use `iteration_range` instead.
## [15:59:59] WARNING: src/c_api/c_api.cc:935: `ntree_limit` is deprecated, use `iteration_range` instead.
## [15:59:59] WARNING: src/c_api/c_api.cc:935: `ntree_limit` is deprecated, use `iteration_range` instead.
## [15:59:59] WARNING: src/c_api/c_api.cc:935: `ntree_limit` is deprecated, use `iteration_range` instead.
## [15:59:59] WARNING: src/c_api/c_api.cc:935: `ntree_limit` is deprecated, use `iteration_range` instead.
## [16:00:00] WARNING: src/c_api/c_api.cc:935: `ntree_limit` is deprecated, use `iteration_range` instead.
## [16:00:00] WARNING: src/c_api/c_api.cc:935: `ntree_limit` is deprecated, use `iteration_range` instead.
## [16:00:00] WARNING: src/c_api/c_api.cc:935: `ntree_limit` is deprecated, use `iteration_range` instead.
## [16:00:00] WARNING: src/c_api/c_api.cc:935: `ntree_limit` is deprecated, use `iteration_range` instead.
## [16:00:00] WARNING: src/c_api/c_api.cc:935: `ntree_limit` is deprecated, use `iteration_range` instead.
## [16:00:00] WARNING: src/c_api/c_api.cc:935: `ntree_limit` is deprecated, use `iteration_range` instead.
## [16:00:00] WARNING: src/c_api/c_api.cc:935: `ntree_limit` is deprecated, use `iteration_range` instead.
## [16:00:00] WARNING: src/c_api/c_api.cc:935: `ntree_limit` is deprecated, use `iteration_range` instead.
## [16:00:00] WARNING: src/c_api/c_api.cc:935: `ntree_limit` is deprecated, use `iteration_range` instead.
## [16:00:00] WARNING: src/c_api/c_api.cc:935: `ntree_limit` is deprecated, use `iteration_range` instead.
## [16:00:01] WARNING: src/c_api/c_api.cc:935: `ntree_limit` is deprecated, use `iteration_range` instead.
## [16:00:01] WARNING: src/c_api/c_api.cc:935: `ntree_limit` is deprecated, use `iteration_range` instead.
## [16:00:01] WARNING: src/c_api/c_api.cc:935: `ntree_limit` is deprecated, use `iteration_range` instead.
## [16:00:01] WARNING: src/c_api/c_api.cc:935: `ntree_limit` is deprecated, use `iteration_range` instead.
## [16:00:01] WARNING: src/c_api/c_api.cc:935: `ntree_limit` is deprecated, use `iteration_range` instead.
## [16:00:01] WARNING: src/c_api/c_api.cc:935: `ntree_limit` is deprecated, use `iteration_range` instead.
## [16:00:01] WARNING: src/c_api/c_api.cc:935: `ntree_limit` is deprecated, use `iteration_range` instead.
## [16:00:01] WARNING: src/c_api/c_api.cc:935: `ntree_limit` is deprecated, use `iteration_range` instead.
## [16:00:02] WARNING: src/c_api/c_api.cc:935: `ntree_limit` is deprecated, use `iteration_range` instead.
## [16:00:02] WARNING: src/c_api/c_api.cc:935: `ntree_limit` is deprecated, use `iteration_range` instead.
## [16:00:02] WARNING: src/c_api/c_api.cc:935: `ntree_limit` is deprecated, use `iteration_range` instead.
## [16:00:02] WARNING: src/c_api/c_api.cc:935: `ntree_limit` is deprecated, use `iteration_range` instead.
## [16:00:02] WARNING: src/c_api/c_api.cc:935: `ntree_limit` is deprecated, use `iteration_range` instead.
## [16:00:02] WARNING: src/c_api/c_api.cc:935: `ntree_limit` is deprecated, use `iteration_range` instead.
## [16:00:02] WARNING: src/c_api/c_api.cc:935: `ntree_limit` is deprecated, use `iteration_range` instead.
## [16:00:02] WARNING: src/c_api/c_api.cc:935: `ntree_limit` is deprecated, use `iteration_range` instead.
## [16:00:02] WARNING: src/c_api/c_api.cc:935: `ntree_limit` is deprecated, use `iteration_range` instead.
## [16:00:02] WARNING: src/c_api/c_api.cc:935: `ntree_limit` is deprecated, use `iteration_range` instead.
## [16:00:02] WARNING: src/c_api/c_api.cc:935: `ntree_limit` is deprecated, use `iteration_range` instead.
## [16:00:02] WARNING: src/c_api/c_api.cc:935: `ntree_limit` is deprecated, use `iteration_range` instead.
## [16:00:03] WARNING: src/c_api/c_api.cc:935: `ntree_limit` is deprecated, use `iteration_range` instead.
## [16:00:03] WARNING: src/c_api/c_api.cc:935: `ntree_limit` is deprecated, use `iteration_range` instead.
## [16:00:03] WARNING: src/c_api/c_api.cc:935: `ntree_limit` is deprecated, use `iteration_range` instead.
## [16:00:03] WARNING: src/c_api/c_api.cc:935: `ntree_limit` is deprecated, use `iteration_range` instead.
## [16:00:03] WARNING: src/c_api/c_api.cc:935: `ntree_limit` is deprecated, use `iteration_range` instead.
## [16:00:03] WARNING: src/c_api/c_api.cc:935: `ntree_limit` is deprecated, use `iteration_range` instead.
## [16:00:03] WARNING: src/c_api/c_api.cc:935: `ntree_limit` is deprecated, use `iteration_range` instead.
## [16:00:03] WARNING: src/c_api/c_api.cc:935: `ntree_limit` is deprecated, use `iteration_range` instead.
## [16:00:03] WARNING: src/c_api/c_api.cc:935: `ntree_limit` is deprecated, use `iteration_range` instead.
## [16:00:03] WARNING: src/c_api/c_api.cc:935: `ntree_limit` is deprecated, use `iteration_range` instead.
## [16:00:03] WARNING: src/c_api/c_api.cc:935: `ntree_limit` is deprecated, use `iteration_range` instead.
## [16:00:03] WARNING: src/c_api/c_api.cc:935: `ntree_limit` is deprecated, use `iteration_range` instead.
## [16:00:04] WARNING: src/c_api/c_api.cc:935: `ntree_limit` is deprecated, use `iteration_range` instead.
## [16:00:04] WARNING: src/c_api/c_api.cc:935: `ntree_limit` is deprecated, use `iteration_range` instead.
## [16:00:04] WARNING: src/c_api/c_api.cc:935: `ntree_limit` is deprecated, use `iteration_range` instead.
## [16:00:04] WARNING: src/c_api/c_api.cc:935: `ntree_limit` is deprecated, use `iteration_range` instead.
## [16:00:04] WARNING: src/c_api/c_api.cc:935: `ntree_limit` is deprecated, use `iteration_range` instead.
## [16:00:04] WARNING: src/c_api/c_api.cc:935: `ntree_limit` is deprecated, use `iteration_range` instead.
## [16:00:04] WARNING: src/c_api/c_api.cc:935: `ntree_limit` is deprecated, use `iteration_range` instead.
## [16:00:04] WARNING: src/c_api/c_api.cc:935: `ntree_limit` is deprecated, use `iteration_range` instead.
## [16:00:04] WARNING: src/c_api/c_api.cc:935: `ntree_limit` is deprecated, use `iteration_range` instead.
## [16:00:04] WARNING: src/c_api/c_api.cc:935: `ntree_limit` is deprecated, use `iteration_range` instead.
## [16:00:05] WARNING: src/c_api/c_api.cc:935: `ntree_limit` is deprecated, use `iteration_range` instead.
## [16:00:05] WARNING: src/c_api/c_api.cc:935: `ntree_limit` is deprecated, use `iteration_range` instead.
## [16:00:05] WARNING: src/c_api/c_api.cc:935: `ntree_limit` is deprecated, use `iteration_range` instead.
## [16:00:05] WARNING: src/c_api/c_api.cc:935: `ntree_limit` is deprecated, use `iteration_range` instead.
## [16:00:05] WARNING: src/c_api/c_api.cc:935: `ntree_limit` is deprecated, use `iteration_range` instead.
## [16:00:05] WARNING: src/c_api/c_api.cc:935: `ntree_limit` is deprecated, use `iteration_range` instead.
## [16:00:08] WARNING: src/c_api/c_api.cc:935: `ntree_limit` is deprecated, use `iteration_range` instead.
## [16:00:08] WARNING: src/c_api/c_api.cc:935: `ntree_limit` is deprecated, use `iteration_range` instead.
## [16:00:08] WARNING: src/c_api/c_api.cc:935: `ntree_limit` is deprecated, use `iteration_range` instead.
## [16:00:08] WARNING: src/c_api/c_api.cc:935: `ntree_limit` is deprecated, use `iteration_range` instead.
## [16:00:08] WARNING: src/c_api/c_api.cc:935: `ntree_limit` is deprecated, use `iteration_range` instead.
## [16:00:08] WARNING: src/c_api/c_api.cc:935: `ntree_limit` is deprecated, use `iteration_range` instead.
## [16:00:08] WARNING: src/c_api/c_api.cc:935: `ntree_limit` is deprecated, use `iteration_range` instead.
## [16:00:08] WARNING: src/c_api/c_api.cc:935: `ntree_limit` is deprecated, use `iteration_range` instead.
## [16:00:08] WARNING: src/c_api/c_api.cc:935: `ntree_limit` is deprecated, use `iteration_range` instead.
## [16:00:08] WARNING: src/c_api/c_api.cc:935: `ntree_limit` is deprecated, use `iteration_range` instead.
## [16:00:08] WARNING: src/c_api/c_api.cc:935: `ntree_limit` is deprecated, use `iteration_range` instead.
## [16:00:08] WARNING: src/c_api/c_api.cc:935: `ntree_limit` is deprecated, use `iteration_range` instead.
## [16:00:09] WARNING: src/c_api/c_api.cc:935: `ntree_limit` is deprecated, use `iteration_range` instead.
## [16:00:09] WARNING: src/c_api/c_api.cc:935: `ntree_limit` is deprecated, use `iteration_range` instead.
## [16:00:09] WARNING: src/c_api/c_api.cc:935: `ntree_limit` is deprecated, use `iteration_range` instead.
## [16:00:09] WARNING: src/c_api/c_api.cc:935: `ntree_limit` is deprecated, use `iteration_range` instead.
## [16:00:09] WARNING: src/c_api/c_api.cc:935: `ntree_limit` is deprecated, use `iteration_range` instead.
## [16:00:09] WARNING: src/c_api/c_api.cc:935: `ntree_limit` is deprecated, use `iteration_range` instead.
## [16:00:09] WARNING: src/c_api/c_api.cc:935: `ntree_limit` is deprecated, use `iteration_range` instead.
## [16:00:09] WARNING: src/c_api/c_api.cc:935: `ntree_limit` is deprecated, use `iteration_range` instead.
## [16:00:09] WARNING: src/c_api/c_api.cc:935: `ntree_limit` is deprecated, use `iteration_range` instead.
## [16:00:09] WARNING: src/c_api/c_api.cc:935: `ntree_limit` is deprecated, use `iteration_range` instead.
## [16:00:09] WARNING: src/c_api/c_api.cc:935: `ntree_limit` is deprecated, use `iteration_range` instead.
## [16:00:09] WARNING: src/c_api/c_api.cc:935: `ntree_limit` is deprecated, use `iteration_range` instead.
## [16:00:10] WARNING: src/c_api/c_api.cc:935: `ntree_limit` is deprecated, use `iteration_range` instead.
## [16:00:10] WARNING: src/c_api/c_api.cc:935: `ntree_limit` is deprecated, use `iteration_range` instead.
## [16:00:10] WARNING: src/c_api/c_api.cc:935: `ntree_limit` is deprecated, use `iteration_range` instead.
## [16:00:10] WARNING: src/c_api/c_api.cc:935: `ntree_limit` is deprecated, use `iteration_range` instead.
## [16:00:10] WARNING: src/c_api/c_api.cc:935: `ntree_limit` is deprecated, use `iteration_range` instead.
## [16:00:10] WARNING: src/c_api/c_api.cc:935: `ntree_limit` is deprecated, use `iteration_range` instead.
## [16:00:10] WARNING: src/c_api/c_api.cc:935: `ntree_limit` is deprecated, use `iteration_range` instead.
## [16:00:10] WARNING: src/c_api/c_api.cc:935: `ntree_limit` is deprecated, use `iteration_range` instead.
## [16:00:10] WARNING: src/c_api/c_api.cc:935: `ntree_limit` is deprecated, use `iteration_range` instead.
## [16:00:10] WARNING: src/c_api/c_api.cc:935: `ntree_limit` is deprecated, use `iteration_range` instead.
## [16:00:10] WARNING: src/c_api/c_api.cc:935: `ntree_limit` is deprecated, use `iteration_range` instead.
## [16:00:10] WARNING: src/c_api/c_api.cc:935: `ntree_limit` is deprecated, use `iteration_range` instead.
## [16:00:11] WARNING: src/c_api/c_api.cc:935: `ntree_limit` is deprecated, use `iteration_range` instead.
## [16:00:11] WARNING: src/c_api/c_api.cc:935: `ntree_limit` is deprecated, use `iteration_range` instead.
## [16:00:11] WARNING: src/c_api/c_api.cc:935: `ntree_limit` is deprecated, use `iteration_range` instead.
## [16:00:11] WARNING: src/c_api/c_api.cc:935: `ntree_limit` is deprecated, use `iteration_range` instead.
## [16:00:11] WARNING: src/c_api/c_api.cc:935: `ntree_limit` is deprecated, use `iteration_range` instead.
## [16:00:11] WARNING: src/c_api/c_api.cc:935: `ntree_limit` is deprecated, use `iteration_range` instead.
## [16:00:11] WARNING: src/c_api/c_api.cc:935: `ntree_limit` is deprecated, use `iteration_range` instead.
## [16:00:11] WARNING: src/c_api/c_api.cc:935: `ntree_limit` is deprecated, use `iteration_range` instead.
## [16:00:11] WARNING: src/c_api/c_api.cc:935: `ntree_limit` is deprecated, use `iteration_range` instead.
## [16:00:11] WARNING: src/c_api/c_api.cc:935: `ntree_limit` is deprecated, use `iteration_range` instead.
## [16:00:11] WARNING: src/c_api/c_api.cc:935: `ntree_limit` is deprecated, use `iteration_range` instead.
## [16:00:11] WARNING: src/c_api/c_api.cc:935: `ntree_limit` is deprecated, use `iteration_range` instead.
## [16:00:11] WARNING: src/c_api/c_api.cc:935: `ntree_limit` is deprecated, use `iteration_range` instead.
## [16:00:11] WARNING: src/c_api/c_api.cc:935: `ntree_limit` is deprecated, use `iteration_range` instead.
## [16:00:12] WARNING: src/c_api/c_api.cc:935: `ntree_limit` is deprecated, use `iteration_range` instead.
## [16:00:12] WARNING: src/c_api/c_api.cc:935: `ntree_limit` is deprecated, use `iteration_range` instead.
## [16:00:12] WARNING: src/c_api/c_api.cc:935: `ntree_limit` is deprecated, use `iteration_range` instead.
## [16:00:12] WARNING: src/c_api/c_api.cc:935: `ntree_limit` is deprecated, use `iteration_range` instead.
## [16:00:12] WARNING: src/c_api/c_api.cc:935: `ntree_limit` is deprecated, use `iteration_range` instead.
## [16:00:12] WARNING: src/c_api/c_api.cc:935: `ntree_limit` is deprecated, use `iteration_range` instead.
## [16:00:12] WARNING: src/c_api/c_api.cc:935: `ntree_limit` is deprecated, use `iteration_range` instead.
## [16:00:12] WARNING: src/c_api/c_api.cc:935: `ntree_limit` is deprecated, use `iteration_range` instead.
## [16:00:12] WARNING: src/c_api/c_api.cc:935: `ntree_limit` is deprecated, use `iteration_range` instead.
## [16:00:12] WARNING: src/c_api/c_api.cc:935: `ntree_limit` is deprecated, use `iteration_range` instead.
## [16:00:12] WARNING: src/c_api/c_api.cc:935: `ntree_limit` is deprecated, use `iteration_range` instead.
## [16:00:12] WARNING: src/c_api/c_api.cc:935: `ntree_limit` is deprecated, use `iteration_range` instead.
## [16:00:13] WARNING: src/c_api/c_api.cc:935: `ntree_limit` is deprecated, use `iteration_range` instead.
## [16:00:13] WARNING: src/c_api/c_api.cc:935: `ntree_limit` is deprecated, use `iteration_range` instead.
## [16:00:13] WARNING: src/c_api/c_api.cc:935: `ntree_limit` is deprecated, use `iteration_range` instead.
## [16:00:13] WARNING: src/c_api/c_api.cc:935: `ntree_limit` is deprecated, use `iteration_range` instead.
## [16:00:13] WARNING: src/c_api/c_api.cc:935: `ntree_limit` is deprecated, use `iteration_range` instead.
## [16:00:13] WARNING: src/c_api/c_api.cc:935: `ntree_limit` is deprecated, use `iteration_range` instead.
## [16:00:13] WARNING: src/c_api/c_api.cc:935: `ntree_limit` is deprecated, use `iteration_range` instead.
## [16:00:13] WARNING: src/c_api/c_api.cc:935: `ntree_limit` is deprecated, use `iteration_range` instead.
## [16:00:13] WARNING: src/c_api/c_api.cc:935: `ntree_limit` is deprecated, use `iteration_range` instead.
## [16:00:13] WARNING: src/c_api/c_api.cc:935: `ntree_limit` is deprecated, use `iteration_range` instead.
## [16:00:13] WARNING: src/c_api/c_api.cc:935: `ntree_limit` is deprecated, use `iteration_range` instead.
## [16:00:13] WARNING: src/c_api/c_api.cc:935: `ntree_limit` is deprecated, use `iteration_range` instead.
## [16:00:14] WARNING: src/c_api/c_api.cc:935: `ntree_limit` is deprecated, use `iteration_range` instead.
## [16:00:14] WARNING: src/c_api/c_api.cc:935: `ntree_limit` is deprecated, use `iteration_range` instead.
## [16:00:14] WARNING: src/c_api/c_api.cc:935: `ntree_limit` is deprecated, use `iteration_range` instead.
## [16:00:14] WARNING: src/c_api/c_api.cc:935: `ntree_limit` is deprecated, use `iteration_range` instead.
## [16:00:14] WARNING: src/c_api/c_api.cc:935: `ntree_limit` is deprecated, use `iteration_range` instead.
## [16:00:14] WARNING: src/c_api/c_api.cc:935: `ntree_limit` is deprecated, use `iteration_range` instead.
## [16:00:14] WARNING: src/c_api/c_api.cc:935: `ntree_limit` is deprecated, use `iteration_range` instead.
## [16:00:14] WARNING: src/c_api/c_api.cc:935: `ntree_limit` is deprecated, use `iteration_range` instead.
## [16:00:14] WARNING: src/c_api/c_api.cc:935: `ntree_limit` is deprecated, use `iteration_range` instead.
## [16:00:14] WARNING: src/c_api/c_api.cc:935: `ntree_limit` is deprecated, use `iteration_range` instead.
## [16:00:14] WARNING: src/c_api/c_api.cc:935: `ntree_limit` is deprecated, use `iteration_range` instead.
## [16:00:14] WARNING: src/c_api/c_api.cc:935: `ntree_limit` is deprecated, use `iteration_range` instead.
## [16:00:14] WARNING: src/c_api/c_api.cc:935: `ntree_limit` is deprecated, use `iteration_range` instead.
## [16:00:14] WARNING: src/c_api/c_api.cc:935: `ntree_limit` is deprecated, use `iteration_range` instead.
## [16:00:15] WARNING: src/c_api/c_api.cc:935: `ntree_limit` is deprecated, use `iteration_range` instead.
## [16:00:15] WARNING: src/c_api/c_api.cc:935: `ntree_limit` is deprecated, use `iteration_range` instead.
## [16:00:15] WARNING: src/c_api/c_api.cc:935: `ntree_limit` is deprecated, use `iteration_range` instead.
## [16:00:15] WARNING: src/c_api/c_api.cc:935: `ntree_limit` is deprecated, use `iteration_range` instead.
## [16:00:15] WARNING: src/c_api/c_api.cc:935: `ntree_limit` is deprecated, use `iteration_range` instead.
## [16:00:15] WARNING: src/c_api/c_api.cc:935: `ntree_limit` is deprecated, use `iteration_range` instead.
## [16:00:15] WARNING: src/c_api/c_api.cc:935: `ntree_limit` is deprecated, use `iteration_range` instead.
## [16:00:15] WARNING: src/c_api/c_api.cc:935: `ntree_limit` is deprecated, use `iteration_range` instead.
## [16:00:15] WARNING: src/c_api/c_api.cc:935: `ntree_limit` is deprecated, use `iteration_range` instead.
## [16:00:15] WARNING: src/c_api/c_api.cc:935: `ntree_limit` is deprecated, use `iteration_range` instead.
## [16:00:15] WARNING: src/c_api/c_api.cc:935: `ntree_limit` is deprecated, use `iteration_range` instead.
## [16:00:15] WARNING: src/c_api/c_api.cc:935: `ntree_limit` is deprecated, use `iteration_range` instead.
## [16:00:16] WARNING: src/c_api/c_api.cc:935: `ntree_limit` is deprecated, use `iteration_range` instead.
## [16:00:16] WARNING: src/c_api/c_api.cc:935: `ntree_limit` is deprecated, use `iteration_range` instead.
## [16:00:16] WARNING: src/c_api/c_api.cc:935: `ntree_limit` is deprecated, use `iteration_range` instead.
## [16:00:16] WARNING: src/c_api/c_api.cc:935: `ntree_limit` is deprecated, use `iteration_range` instead.
## [16:00:16] WARNING: src/c_api/c_api.cc:935: `ntree_limit` is deprecated, use `iteration_range` instead.
## [16:00:16] WARNING: src/c_api/c_api.cc:935: `ntree_limit` is deprecated, use `iteration_range` instead.
## [16:00:16] WARNING: src/c_api/c_api.cc:935: `ntree_limit` is deprecated, use `iteration_range` instead.
## [16:00:16] WARNING: src/c_api/c_api.cc:935: `ntree_limit` is deprecated, use `iteration_range` instead.
## [16:00:16] WARNING: src/c_api/c_api.cc:935: `ntree_limit` is deprecated, use `iteration_range` instead.
## [16:00:16] WARNING: src/c_api/c_api.cc:935: `ntree_limit` is deprecated, use `iteration_range` instead.
## [16:00:16] WARNING: src/c_api/c_api.cc:935: `ntree_limit` is deprecated, use `iteration_range` instead.
## [16:00:16] WARNING: src/c_api/c_api.cc:935: `ntree_limit` is deprecated, use `iteration_range` instead.
## [16:00:17] WARNING: src/c_api/c_api.cc:935: `ntree_limit` is deprecated, use `iteration_range` instead.
## [16:00:17] WARNING: src/c_api/c_api.cc:935: `ntree_limit` is deprecated, use `iteration_range` instead.
## [16:00:17] WARNING: src/c_api/c_api.cc:935: `ntree_limit` is deprecated, use `iteration_range` instead.
## [16:00:17] WARNING: src/c_api/c_api.cc:935: `ntree_limit` is deprecated, use `iteration_range` instead.
## [16:00:17] WARNING: src/c_api/c_api.cc:935: `ntree_limit` is deprecated, use `iteration_range` instead.
## [16:00:17] WARNING: src/c_api/c_api.cc:935: `ntree_limit` is deprecated, use `iteration_range` instead.
## [16:00:17] WARNING: src/c_api/c_api.cc:935: `ntree_limit` is deprecated, use `iteration_range` instead.
## [16:00:17] WARNING: src/c_api/c_api.cc:935: `ntree_limit` is deprecated, use `iteration_range` instead.
## [16:00:17] WARNING: src/c_api/c_api.cc:935: `ntree_limit` is deprecated, use `iteration_range` instead.
## [16:00:17] WARNING: src/c_api/c_api.cc:935: `ntree_limit` is deprecated, use `iteration_range` instead.
## [16:00:17] WARNING: src/c_api/c_api.cc:935: `ntree_limit` is deprecated, use `iteration_range` instead.
## [16:00:17] WARNING: src/c_api/c_api.cc:935: `ntree_limit` is deprecated, use `iteration_range` instead.
## [16:00:17] WARNING: src/c_api/c_api.cc:935: `ntree_limit` is deprecated, use `iteration_range` instead.
## [16:00:17] WARNING: src/c_api/c_api.cc:935: `ntree_limit` is deprecated, use `iteration_range` instead.
## [16:00:18] WARNING: src/c_api/c_api.cc:935: `ntree_limit` is deprecated, use `iteration_range` instead.
## [16:00:18] WARNING: src/c_api/c_api.cc:935: `ntree_limit` is deprecated, use `iteration_range` instead.
## [16:00:18] WARNING: src/c_api/c_api.cc:935: `ntree_limit` is deprecated, use `iteration_range` instead.
## [16:00:18] WARNING: src/c_api/c_api.cc:935: `ntree_limit` is deprecated, use `iteration_range` instead.
## [16:00:18] WARNING: src/c_api/c_api.cc:935: `ntree_limit` is deprecated, use `iteration_range` instead.
## [16:00:18] WARNING: src/c_api/c_api.cc:935: `ntree_limit` is deprecated, use `iteration_range` instead.
## [16:00:18] WARNING: src/c_api/c_api.cc:935: `ntree_limit` is deprecated, use `iteration_range` instead.
## [16:00:18] WARNING: src/c_api/c_api.cc:935: `ntree_limit` is deprecated, use `iteration_range` instead.
## [16:00:18] WARNING: src/c_api/c_api.cc:935: `ntree_limit` is deprecated, use `iteration_range` instead.
## [16:00:18] WARNING: src/c_api/c_api.cc:935: `ntree_limit` is deprecated, use `iteration_range` instead.
## [16:00:18] WARNING: src/c_api/c_api.cc:935: `ntree_limit` is deprecated, use `iteration_range` instead.
## [16:00:18] WARNING: src/c_api/c_api.cc:935: `ntree_limit` is deprecated, use `iteration_range` instead.
## [16:00:19] WARNING: src/c_api/c_api.cc:935: `ntree_limit` is deprecated, use `iteration_range` instead.
## [16:00:19] WARNING: src/c_api/c_api.cc:935: `ntree_limit` is deprecated, use `iteration_range` instead.
## [16:00:19] WARNING: src/c_api/c_api.cc:935: `ntree_limit` is deprecated, use `iteration_range` instead.
## [16:00:19] WARNING: src/c_api/c_api.cc:935: `ntree_limit` is deprecated, use `iteration_range` instead.
## [16:00:19] WARNING: src/c_api/c_api.cc:935: `ntree_limit` is deprecated, use `iteration_range` instead.
## [16:00:19] WARNING: src/c_api/c_api.cc:935: `ntree_limit` is deprecated, use `iteration_range` instead.
## [16:00:19] WARNING: src/c_api/c_api.cc:935: `ntree_limit` is deprecated, use `iteration_range` instead.
## [16:00:19] WARNING: src/c_api/c_api.cc:935: `ntree_limit` is deprecated, use `iteration_range` instead.
## [16:00:19] WARNING: src/c_api/c_api.cc:935: `ntree_limit` is deprecated, use `iteration_range` instead.
## [16:00:19] WARNING: src/c_api/c_api.cc:935: `ntree_limit` is deprecated, use `iteration_range` instead.
## [16:00:19] WARNING: src/c_api/c_api.cc:935: `ntree_limit` is deprecated, use `iteration_range` instead.
## [16:00:19] WARNING: src/c_api/c_api.cc:935: `ntree_limit` is deprecated, use `iteration_range` instead.
## [16:00:19] WARNING: src/c_api/c_api.cc:935: `ntree_limit` is deprecated, use `iteration_range` instead.
## [16:00:19] WARNING: src/c_api/c_api.cc:935: `ntree_limit` is deprecated, use `iteration_range` instead.
## [16:00:20] WARNING: src/c_api/c_api.cc:935: `ntree_limit` is deprecated, use `iteration_range` instead.
## [16:00:20] WARNING: src/c_api/c_api.cc:935: `ntree_limit` is deprecated, use `iteration_range` instead.
## [16:00:20] WARNING: src/c_api/c_api.cc:935: `ntree_limit` is deprecated, use `iteration_range` instead.
## [16:00:20] WARNING: src/c_api/c_api.cc:935: `ntree_limit` is deprecated, use `iteration_range` instead.
## [16:00:20] WARNING: src/c_api/c_api.cc:935: `ntree_limit` is deprecated, use `iteration_range` instead.
## [16:00:20] WARNING: src/c_api/c_api.cc:935: `ntree_limit` is deprecated, use `iteration_range` instead.
## [16:00:20] WARNING: src/c_api/c_api.cc:935: `ntree_limit` is deprecated, use `iteration_range` instead.
## [16:00:20] WARNING: src/c_api/c_api.cc:935: `ntree_limit` is deprecated, use `iteration_range` instead.
## [16:00:20] WARNING: src/c_api/c_api.cc:935: `ntree_limit` is deprecated, use `iteration_range` instead.
## [16:00:20] WARNING: src/c_api/c_api.cc:935: `ntree_limit` is deprecated, use `iteration_range` instead.
## [16:00:20] WARNING: src/c_api/c_api.cc:935: `ntree_limit` is deprecated, use `iteration_range` instead.
## [16:00:20] WARNING: src/c_api/c_api.cc:935: `ntree_limit` is deprecated, use `iteration_range` instead.
## [16:00:20] WARNING: src/c_api/c_api.cc:935: `ntree_limit` is deprecated, use `iteration_range` instead.
## [16:00:20] WARNING: src/c_api/c_api.cc:935: `ntree_limit` is deprecated, use `iteration_range` instead.
## [16:00:21] WARNING: src/c_api/c_api.cc:935: `ntree_limit` is deprecated, use `iteration_range` instead.
## [16:00:21] WARNING: src/c_api/c_api.cc:935: `ntree_limit` is deprecated, use `iteration_range` instead.
## [16:00:21] WARNING: src/c_api/c_api.cc:935: `ntree_limit` is deprecated, use `iteration_range` instead.
## [16:00:21] WARNING: src/c_api/c_api.cc:935: `ntree_limit` is deprecated, use `iteration_range` instead.
## [16:00:21] WARNING: src/c_api/c_api.cc:935: `ntree_limit` is deprecated, use `iteration_range` instead.
## [16:00:21] WARNING: src/c_api/c_api.cc:935: `ntree_limit` is deprecated, use `iteration_range` instead.
## [16:00:21] WARNING: src/c_api/c_api.cc:935: `ntree_limit` is deprecated, use `iteration_range` instead.
## [16:00:21] WARNING: src/c_api/c_api.cc:935: `ntree_limit` is deprecated, use `iteration_range` instead.
## [16:00:21] WARNING: src/c_api/c_api.cc:935: `ntree_limit` is deprecated, use `iteration_range` instead.
## [16:00:21] WARNING: src/c_api/c_api.cc:935: `ntree_limit` is deprecated, use `iteration_range` instead.
## [16:00:22] WARNING: src/c_api/c_api.cc:935: `ntree_limit` is deprecated, use `iteration_range` instead.
## [16:00:22] WARNING: src/c_api/c_api.cc:935: `ntree_limit` is deprecated, use `iteration_range` instead.
## [16:00:22] WARNING: src/c_api/c_api.cc:935: `ntree_limit` is deprecated, use `iteration_range` instead.
## [16:00:22] WARNING: src/c_api/c_api.cc:935: `ntree_limit` is deprecated, use `iteration_range` instead.
## [16:00:22] WARNING: src/c_api/c_api.cc:935: `ntree_limit` is deprecated, use `iteration_range` instead.
## [16:00:22] WARNING: src/c_api/c_api.cc:935: `ntree_limit` is deprecated, use `iteration_range` instead.
## [16:00:22] WARNING: src/c_api/c_api.cc:935: `ntree_limit` is deprecated, use `iteration_range` instead.
## [16:00:22] WARNING: src/c_api/c_api.cc:935: `ntree_limit` is deprecated, use `iteration_range` instead.
## [16:00:22] WARNING: src/c_api/c_api.cc:935: `ntree_limit` is deprecated, use `iteration_range` instead.
## [16:00:22] WARNING: src/c_api/c_api.cc:935: `ntree_limit` is deprecated, use `iteration_range` instead.
## [16:00:22] WARNING: src/c_api/c_api.cc:935: `ntree_limit` is deprecated, use `iteration_range` instead.
## [16:00:22] WARNING: src/c_api/c_api.cc:935: `ntree_limit` is deprecated, use `iteration_range` instead.
## [16:00:22] WARNING: src/c_api/c_api.cc:935: `ntree_limit` is deprecated, use `iteration_range` instead.
## [16:00:22] WARNING: src/c_api/c_api.cc:935: `ntree_limit` is deprecated, use `iteration_range` instead.
## [16:00:22] WARNING: src/c_api/c_api.cc:935: `ntree_limit` is deprecated, use `iteration_range` instead.
## [16:00:22] WARNING: src/c_api/c_api.cc:935: `ntree_limit` is deprecated, use `iteration_range` instead.
## [16:00:23] WARNING: src/c_api/c_api.cc:935: `ntree_limit` is deprecated, use `iteration_range` instead.
## [16:00:23] WARNING: src/c_api/c_api.cc:935: `ntree_limit` is deprecated, use `iteration_range` instead.
## [16:00:23] WARNING: src/c_api/c_api.cc:935: `ntree_limit` is deprecated, use `iteration_range` instead.
## [16:00:23] WARNING: src/c_api/c_api.cc:935: `ntree_limit` is deprecated, use `iteration_range` instead.
## [16:00:23] WARNING: src/c_api/c_api.cc:935: `ntree_limit` is deprecated, use `iteration_range` instead.
## [16:00:23] WARNING: src/c_api/c_api.cc:935: `ntree_limit` is deprecated, use `iteration_range` instead.
## [16:00:23] WARNING: src/c_api/c_api.cc:935: `ntree_limit` is deprecated, use `iteration_range` instead.
## [16:00:23] WARNING: src/c_api/c_api.cc:935: `ntree_limit` is deprecated, use `iteration_range` instead.
## [16:00:23] WARNING: src/c_api/c_api.cc:935: `ntree_limit` is deprecated, use `iteration_range` instead.
## [16:00:23] WARNING: src/c_api/c_api.cc:935: `ntree_limit` is deprecated, use `iteration_range` instead.
## [16:00:23] WARNING: src/c_api/c_api.cc:935: `ntree_limit` is deprecated, use `iteration_range` instead.
## [16:00:23] WARNING: src/c_api/c_api.cc:935: `ntree_limit` is deprecated, use `iteration_range` instead.
## [16:00:24] WARNING: src/c_api/c_api.cc:935: `ntree_limit` is deprecated, use `iteration_range` instead.
## [16:00:24] WARNING: src/c_api/c_api.cc:935: `ntree_limit` is deprecated, use `iteration_range` instead.
## [16:00:24] WARNING: src/c_api/c_api.cc:935: `ntree_limit` is deprecated, use `iteration_range` instead.
## [16:00:24] WARNING: src/c_api/c_api.cc:935: `ntree_limit` is deprecated, use `iteration_range` instead.
## [16:00:24] WARNING: src/c_api/c_api.cc:935: `ntree_limit` is deprecated, use `iteration_range` instead.
## [16:00:24] WARNING: src/c_api/c_api.cc:935: `ntree_limit` is deprecated, use `iteration_range` instead.
## [16:00:24] WARNING: src/c_api/c_api.cc:935: `ntree_limit` is deprecated, use `iteration_range` instead.
## [16:00:24] WARNING: src/c_api/c_api.cc:935: `ntree_limit` is deprecated, use `iteration_range` instead.
## [16:00:24] WARNING: src/c_api/c_api.cc:935: `ntree_limit` is deprecated, use `iteration_range` instead.
## [16:00:24] WARNING: src/c_api/c_api.cc:935: `ntree_limit` is deprecated, use `iteration_range` instead.
## [16:00:24] WARNING: src/c_api/c_api.cc:935: `ntree_limit` is deprecated, use `iteration_range` instead.
## [16:00:24] WARNING: src/c_api/c_api.cc:935: `ntree_limit` is deprecated, use `iteration_range` instead.
## [16:00:25] WARNING: src/c_api/c_api.cc:935: `ntree_limit` is deprecated, use `iteration_range` instead.
## [16:00:25] WARNING: src/c_api/c_api.cc:935: `ntree_limit` is deprecated, use `iteration_range` instead.
## [16:00:25] WARNING: src/c_api/c_api.cc:935: `ntree_limit` is deprecated, use `iteration_range` instead.
## [16:00:25] WARNING: src/c_api/c_api.cc:935: `ntree_limit` is deprecated, use `iteration_range` instead.
## [16:00:25] WARNING: src/c_api/c_api.cc:935: `ntree_limit` is deprecated, use `iteration_range` instead.
## [16:00:25] WARNING: src/c_api/c_api.cc:935: `ntree_limit` is deprecated, use `iteration_range` instead.
## [16:00:25] WARNING: src/c_api/c_api.cc:935: `ntree_limit` is deprecated, use `iteration_range` instead.
## [16:00:25] WARNING: src/c_api/c_api.cc:935: `ntree_limit` is deprecated, use `iteration_range` instead.
## [16:00:25] WARNING: src/c_api/c_api.cc:935: `ntree_limit` is deprecated, use `iteration_range` instead.
## [16:00:25] WARNING: src/c_api/c_api.cc:935: `ntree_limit` is deprecated, use `iteration_range` instead.
## [16:00:25] WARNING: src/c_api/c_api.cc:935: `ntree_limit` is deprecated, use `iteration_range` instead.
## [16:00:25] WARNING: src/c_api/c_api.cc:935: `ntree_limit` is deprecated, use `iteration_range` instead.
## [16:00:25] WARNING: src/c_api/c_api.cc:935: `ntree_limit` is deprecated, use `iteration_range` instead.
## [16:00:25] WARNING: src/c_api/c_api.cc:935: `ntree_limit` is deprecated, use `iteration_range` instead.
## [16:00:26] WARNING: src/c_api/c_api.cc:935: `ntree_limit` is deprecated, use `iteration_range` instead.
## [16:00:26] WARNING: src/c_api/c_api.cc:935: `ntree_limit` is deprecated, use `iteration_range` instead.
## [16:00:26] WARNING: src/c_api/c_api.cc:935: `ntree_limit` is deprecated, use `iteration_range` instead.
## [16:00:26] WARNING: src/c_api/c_api.cc:935: `ntree_limit` is deprecated, use `iteration_range` instead.
## [16:00:26] WARNING: src/c_api/c_api.cc:935: `ntree_limit` is deprecated, use `iteration_range` instead.
## [16:00:26] WARNING: src/c_api/c_api.cc:935: `ntree_limit` is deprecated, use `iteration_range` instead.
## [16:00:26] WARNING: src/c_api/c_api.cc:935: `ntree_limit` is deprecated, use `iteration_range` instead.
## [16:00:26] WARNING: src/c_api/c_api.cc:935: `ntree_limit` is deprecated, use `iteration_range` instead.
## [16:00:26] WARNING: src/c_api/c_api.cc:935: `ntree_limit` is deprecated, use `iteration_range` instead.
## [16:00:26] WARNING: src/c_api/c_api.cc:935: `ntree_limit` is deprecated, use `iteration_range` instead.
## [16:00:26] WARNING: src/c_api/c_api.cc:935: `ntree_limit` is deprecated, use `iteration_range` instead.
## [16:00:26] WARNING: src/c_api/c_api.cc:935: `ntree_limit` is deprecated, use `iteration_range` instead.
## [16:00:27] WARNING: src/c_api/c_api.cc:935: `ntree_limit` is deprecated, use `iteration_range` instead.
## [16:00:27] WARNING: src/c_api/c_api.cc:935: `ntree_limit` is deprecated, use `iteration_range` instead.
## [16:00:27] WARNING: src/c_api/c_api.cc:935: `ntree_limit` is deprecated, use `iteration_range` instead.
## [16:00:27] WARNING: src/c_api/c_api.cc:935: `ntree_limit` is deprecated, use `iteration_range` instead.
## [16:00:27] WARNING: src/c_api/c_api.cc:935: `ntree_limit` is deprecated, use `iteration_range` instead.
## [16:00:27] WARNING: src/c_api/c_api.cc:935: `ntree_limit` is deprecated, use `iteration_range` instead.
## [16:00:27] WARNING: src/c_api/c_api.cc:935: `ntree_limit` is deprecated, use `iteration_range` instead.
## [16:00:27] WARNING: src/c_api/c_api.cc:935: `ntree_limit` is deprecated, use `iteration_range` instead.
## [16:00:27] WARNING: src/c_api/c_api.cc:935: `ntree_limit` is deprecated, use `iteration_range` instead.
## [16:00:27] WARNING: src/c_api/c_api.cc:935: `ntree_limit` is deprecated, use `iteration_range` instead.
## [16:00:28] WARNING: src/c_api/c_api.cc:935: `ntree_limit` is deprecated, use `iteration_range` instead.
## [16:00:28] WARNING: src/c_api/c_api.cc:935: `ntree_limit` is deprecated, use `iteration_range` instead.
## [16:00:28] WARNING: src/c_api/c_api.cc:935: `ntree_limit` is deprecated, use `iteration_range` instead.
## [16:00:28] WARNING: src/c_api/c_api.cc:935: `ntree_limit` is deprecated, use `iteration_range` instead.
## [16:00:28] WARNING: src/c_api/c_api.cc:935: `ntree_limit` is deprecated, use `iteration_range` instead.
## [16:00:28] WARNING: src/c_api/c_api.cc:935: `ntree_limit` is deprecated, use `iteration_range` instead.
## [16:00:28] WARNING: src/c_api/c_api.cc:935: `ntree_limit` is deprecated, use `iteration_range` instead.
## [16:00:28] WARNING: src/c_api/c_api.cc:935: `ntree_limit` is deprecated, use `iteration_range` instead.
## [16:00:28] WARNING: src/c_api/c_api.cc:935: `ntree_limit` is deprecated, use `iteration_range` instead.
## [16:00:28] WARNING: src/c_api/c_api.cc:935: `ntree_limit` is deprecated, use `iteration_range` instead.
## [16:00:28] WARNING: src/c_api/c_api.cc:935: `ntree_limit` is deprecated, use `iteration_range` instead.
## [16:00:28] WARNING: src/c_api/c_api.cc:935: `ntree_limit` is deprecated, use `iteration_range` instead.
## [16:00:28] WARNING: src/c_api/c_api.cc:935: `ntree_limit` is deprecated, use `iteration_range` instead.
## [16:00:28] WARNING: src/c_api/c_api.cc:935: `ntree_limit` is deprecated, use `iteration_range` instead.
## [16:00:28] WARNING: src/c_api/c_api.cc:935: `ntree_limit` is deprecated, use `iteration_range` instead.
## [16:00:28] WARNING: src/c_api/c_api.cc:935: `ntree_limit` is deprecated, use `iteration_range` instead.
## [16:00:29] WARNING: src/c_api/c_api.cc:935: `ntree_limit` is deprecated, use `iteration_range` instead.
## [16:00:29] WARNING: src/c_api/c_api.cc:935: `ntree_limit` is deprecated, use `iteration_range` instead.
## [16:00:29] WARNING: src/c_api/c_api.cc:935: `ntree_limit` is deprecated, use `iteration_range` instead.
## [16:00:29] WARNING: src/c_api/c_api.cc:935: `ntree_limit` is deprecated, use `iteration_range` instead.
## [16:00:29] WARNING: src/c_api/c_api.cc:935: `ntree_limit` is deprecated, use `iteration_range` instead.
## [16:00:29] WARNING: src/c_api/c_api.cc:935: `ntree_limit` is deprecated, use `iteration_range` instead.
## [16:00:29] WARNING: src/c_api/c_api.cc:935: `ntree_limit` is deprecated, use `iteration_range` instead.
## [16:00:29] WARNING: src/c_api/c_api.cc:935: `ntree_limit` is deprecated, use `iteration_range` instead.
## [16:00:29] WARNING: src/c_api/c_api.cc:935: `ntree_limit` is deprecated, use `iteration_range` instead.
## [16:00:29] WARNING: src/c_api/c_api.cc:935: `ntree_limit` is deprecated, use `iteration_range` instead.
## [16:00:29] WARNING: src/c_api/c_api.cc:935: `ntree_limit` is deprecated, use `iteration_range` instead.
## [16:00:29] WARNING: src/c_api/c_api.cc:935: `ntree_limit` is deprecated, use `iteration_range` instead.
## [16:00:30] WARNING: src/c_api/c_api.cc:935: `ntree_limit` is deprecated, use `iteration_range` instead.
## [16:00:30] WARNING: src/c_api/c_api.cc:935: `ntree_limit` is deprecated, use `iteration_range` instead.
## [16:00:30] WARNING: src/c_api/c_api.cc:935: `ntree_limit` is deprecated, use `iteration_range` instead.
## [16:00:30] WARNING: src/c_api/c_api.cc:935: `ntree_limit` is deprecated, use `iteration_range` instead.
## [16:00:30] WARNING: src/c_api/c_api.cc:935: `ntree_limit` is deprecated, use `iteration_range` instead.
## [16:00:30] WARNING: src/c_api/c_api.cc:935: `ntree_limit` is deprecated, use `iteration_range` instead.
## [16:00:30] WARNING: src/c_api/c_api.cc:935: `ntree_limit` is deprecated, use `iteration_range` instead.
## [16:00:30] WARNING: src/c_api/c_api.cc:935: `ntree_limit` is deprecated, use `iteration_range` instead.
## [16:00:33] WARNING: src/c_api/c_api.cc:935: `ntree_limit` is deprecated, use `iteration_range` instead.
## [16:00:33] WARNING: src/c_api/c_api.cc:935: `ntree_limit` is deprecated, use `iteration_range` instead.
## [16:00:33] WARNING: src/c_api/c_api.cc:935: `ntree_limit` is deprecated, use `iteration_range` instead.
## [16:00:33] WARNING: src/c_api/c_api.cc:935: `ntree_limit` is deprecated, use `iteration_range` instead.
## [16:00:33] WARNING: src/c_api/c_api.cc:935: `ntree_limit` is deprecated, use `iteration_range` instead.
## [16:00:33] WARNING: src/c_api/c_api.cc:935: `ntree_limit` is deprecated, use `iteration_range` instead.
## [16:00:33] WARNING: src/c_api/c_api.cc:935: `ntree_limit` is deprecated, use `iteration_range` instead.
## [16:00:33] WARNING: src/c_api/c_api.cc:935: `ntree_limit` is deprecated, use `iteration_range` instead.
## [16:00:33] WARNING: src/c_api/c_api.cc:935: `ntree_limit` is deprecated, use `iteration_range` instead.
## [16:00:33] WARNING: src/c_api/c_api.cc:935: `ntree_limit` is deprecated, use `iteration_range` instead.
## [16:00:34] WARNING: src/c_api/c_api.cc:935: `ntree_limit` is deprecated, use `iteration_range` instead.
## [16:00:34] WARNING: src/c_api/c_api.cc:935: `ntree_limit` is deprecated, use `iteration_range` instead.
## [16:00:34] WARNING: src/c_api/c_api.cc:935: `ntree_limit` is deprecated, use `iteration_range` instead.
## [16:00:34] WARNING: src/c_api/c_api.cc:935: `ntree_limit` is deprecated, use `iteration_range` instead.
## [16:00:34] WARNING: src/c_api/c_api.cc:935: `ntree_limit` is deprecated, use `iteration_range` instead.
## [16:00:34] WARNING: src/c_api/c_api.cc:935: `ntree_limit` is deprecated, use `iteration_range` instead.
## [16:00:34] WARNING: src/c_api/c_api.cc:935: `ntree_limit` is deprecated, use `iteration_range` instead.
## [16:00:34] WARNING: src/c_api/c_api.cc:935: `ntree_limit` is deprecated, use `iteration_range` instead.
## [16:00:34] WARNING: src/c_api/c_api.cc:935: `ntree_limit` is deprecated, use `iteration_range` instead.
## [16:00:34] WARNING: src/c_api/c_api.cc:935: `ntree_limit` is deprecated, use `iteration_range` instead.
## [16:00:34] WARNING: src/c_api/c_api.cc:935: `ntree_limit` is deprecated, use `iteration_range` instead.
## [16:00:34] WARNING: src/c_api/c_api.cc:935: `ntree_limit` is deprecated, use `iteration_range` instead.
## [16:00:35] WARNING: src/c_api/c_api.cc:935: `ntree_limit` is deprecated, use `iteration_range` instead.
## [16:00:35] WARNING: src/c_api/c_api.cc:935: `ntree_limit` is deprecated, use `iteration_range` instead.
## [16:00:35] WARNING: src/c_api/c_api.cc:935: `ntree_limit` is deprecated, use `iteration_range` instead.
## [16:00:35] WARNING: src/c_api/c_api.cc:935: `ntree_limit` is deprecated, use `iteration_range` instead.
## [16:00:35] WARNING: src/c_api/c_api.cc:935: `ntree_limit` is deprecated, use `iteration_range` instead.
## [16:00:35] WARNING: src/c_api/c_api.cc:935: `ntree_limit` is deprecated, use `iteration_range` instead.
## [16:00:35] WARNING: src/c_api/c_api.cc:935: `ntree_limit` is deprecated, use `iteration_range` instead.
## [16:00:35] WARNING: src/c_api/c_api.cc:935: `ntree_limit` is deprecated, use `iteration_range` instead.
## [16:00:36] WARNING: src/c_api/c_api.cc:935: `ntree_limit` is deprecated, use `iteration_range` instead.
## [16:00:36] WARNING: src/c_api/c_api.cc:935: `ntree_limit` is deprecated, use `iteration_range` instead.
## [16:00:36] WARNING: src/c_api/c_api.cc:935: `ntree_limit` is deprecated, use `iteration_range` instead.
## [16:00:36] WARNING: src/c_api/c_api.cc:935: `ntree_limit` is deprecated, use `iteration_range` instead.
## [16:00:36] WARNING: src/c_api/c_api.cc:935: `ntree_limit` is deprecated, use `iteration_range` instead.
## [16:00:36] WARNING: src/c_api/c_api.cc:935: `ntree_limit` is deprecated, use `iteration_range` instead.
## [16:00:36] WARNING: src/c_api/c_api.cc:935: `ntree_limit` is deprecated, use `iteration_range` instead.
## [16:00:36] WARNING: src/c_api/c_api.cc:935: `ntree_limit` is deprecated, use `iteration_range` instead.
## [16:00:36] WARNING: src/c_api/c_api.cc:935: `ntree_limit` is deprecated, use `iteration_range` instead.
## [16:00:36] WARNING: src/c_api/c_api.cc:935: `ntree_limit` is deprecated, use `iteration_range` instead.
## [16:00:36] WARNING: src/c_api/c_api.cc:935: `ntree_limit` is deprecated, use `iteration_range` instead.
## [16:00:36] WARNING: src/c_api/c_api.cc:935: `ntree_limit` is deprecated, use `iteration_range` instead.
## [16:00:37] WARNING: src/c_api/c_api.cc:935: `ntree_limit` is deprecated, use `iteration_range` instead.
## [16:00:37] WARNING: src/c_api/c_api.cc:935: `ntree_limit` is deprecated, use `iteration_range` instead.
## [16:00:37] WARNING: src/c_api/c_api.cc:935: `ntree_limit` is deprecated, use `iteration_range` instead.
## [16:00:37] WARNING: src/c_api/c_api.cc:935: `ntree_limit` is deprecated, use `iteration_range` instead.
## [16:00:37] WARNING: src/c_api/c_api.cc:935: `ntree_limit` is deprecated, use `iteration_range` instead.
## [16:00:37] WARNING: src/c_api/c_api.cc:935: `ntree_limit` is deprecated, use `iteration_range` instead.
## [16:00:37] WARNING: src/c_api/c_api.cc:935: `ntree_limit` is deprecated, use `iteration_range` instead.
## [16:00:37] WARNING: src/c_api/c_api.cc:935: `ntree_limit` is deprecated, use `iteration_range` instead.
## [16:00:37] WARNING: src/c_api/c_api.cc:935: `ntree_limit` is deprecated, use `iteration_range` instead.
## [16:00:37] WARNING: src/c_api/c_api.cc:935: `ntree_limit` is deprecated, use `iteration_range` instead.
## [16:00:37] WARNING: src/c_api/c_api.cc:935: `ntree_limit` is deprecated, use `iteration_range` instead.
## [16:00:37] WARNING: src/c_api/c_api.cc:935: `ntree_limit` is deprecated, use `iteration_range` instead.
## [16:00:37] WARNING: src/c_api/c_api.cc:935: `ntree_limit` is deprecated, use `iteration_range` instead.
## [16:00:37] WARNING: src/c_api/c_api.cc:935: `ntree_limit` is deprecated, use `iteration_range` instead.
## [16:00:38] WARNING: src/c_api/c_api.cc:935: `ntree_limit` is deprecated, use `iteration_range` instead.
## [16:00:38] WARNING: src/c_api/c_api.cc:935: `ntree_limit` is deprecated, use `iteration_range` instead.
## [16:00:38] WARNING: src/c_api/c_api.cc:935: `ntree_limit` is deprecated, use `iteration_range` instead.
## [16:00:38] WARNING: src/c_api/c_api.cc:935: `ntree_limit` is deprecated, use `iteration_range` instead.
## [16:00:38] WARNING: src/c_api/c_api.cc:935: `ntree_limit` is deprecated, use `iteration_range` instead.
## [16:00:38] WARNING: src/c_api/c_api.cc:935: `ntree_limit` is deprecated, use `iteration_range` instead.
## [16:00:38] WARNING: src/c_api/c_api.cc:935: `ntree_limit` is deprecated, use `iteration_range` instead.
## [16:00:38] WARNING: src/c_api/c_api.cc:935: `ntree_limit` is deprecated, use `iteration_range` instead.
## [16:00:38] WARNING: src/c_api/c_api.cc:935: `ntree_limit` is deprecated, use `iteration_range` instead.
## [16:00:38] WARNING: src/c_api/c_api.cc:935: `ntree_limit` is deprecated, use `iteration_range` instead.
## [16:00:39] WARNING: src/c_api/c_api.cc:935: `ntree_limit` is deprecated, use `iteration_range` instead.
## [16:00:39] WARNING: src/c_api/c_api.cc:935: `ntree_limit` is deprecated, use `iteration_range` instead.
## [16:00:39] WARNING: src/c_api/c_api.cc:935: `ntree_limit` is deprecated, use `iteration_range` instead.
## [16:00:39] WARNING: src/c_api/c_api.cc:935: `ntree_limit` is deprecated, use `iteration_range` instead.
## [16:00:39] WARNING: src/c_api/c_api.cc:935: `ntree_limit` is deprecated, use `iteration_range` instead.
## [16:00:39] WARNING: src/c_api/c_api.cc:935: `ntree_limit` is deprecated, use `iteration_range` instead.
## [16:00:39] WARNING: src/c_api/c_api.cc:935: `ntree_limit` is deprecated, use `iteration_range` instead.
## [16:00:39] WARNING: src/c_api/c_api.cc:935: `ntree_limit` is deprecated, use `iteration_range` instead.
## [16:00:39] WARNING: src/c_api/c_api.cc:935: `ntree_limit` is deprecated, use `iteration_range` instead.
## [16:00:39] WARNING: src/c_api/c_api.cc:935: `ntree_limit` is deprecated, use `iteration_range` instead.
## [16:00:39] WARNING: src/c_api/c_api.cc:935: `ntree_limit` is deprecated, use `iteration_range` instead.
## [16:00:39] WARNING: src/c_api/c_api.cc:935: `ntree_limit` is deprecated, use `iteration_range` instead.
## [16:00:40] WARNING: src/c_api/c_api.cc:935: `ntree_limit` is deprecated, use `iteration_range` instead.
## [16:00:40] WARNING: src/c_api/c_api.cc:935: `ntree_limit` is deprecated, use `iteration_range` instead.
## [16:00:40] WARNING: src/c_api/c_api.cc:935: `ntree_limit` is deprecated, use `iteration_range` instead.
## [16:00:40] WARNING: src/c_api/c_api.cc:935: `ntree_limit` is deprecated, use `iteration_range` instead.
## [16:00:40] WARNING: src/c_api/c_api.cc:935: `ntree_limit` is deprecated, use `iteration_range` instead.
## [16:00:40] WARNING: src/c_api/c_api.cc:935: `ntree_limit` is deprecated, use `iteration_range` instead.
## [16:00:40] WARNING: src/c_api/c_api.cc:935: `ntree_limit` is deprecated, use `iteration_range` instead.
## [16:00:40] WARNING: src/c_api/c_api.cc:935: `ntree_limit` is deprecated, use `iteration_range` instead.
## [16:00:40] WARNING: src/c_api/c_api.cc:935: `ntree_limit` is deprecated, use `iteration_range` instead.
## [16:00:40] WARNING: src/c_api/c_api.cc:935: `ntree_limit` is deprecated, use `iteration_range` instead.
## [16:00:40] WARNING: src/c_api/c_api.cc:935: `ntree_limit` is deprecated, use `iteration_range` instead.
## [16:00:40] WARNING: src/c_api/c_api.cc:935: `ntree_limit` is deprecated, use `iteration_range` instead.
## [16:00:40] WARNING: src/c_api/c_api.cc:935: `ntree_limit` is deprecated, use `iteration_range` instead.
## [16:00:40] WARNING: src/c_api/c_api.cc:935: `ntree_limit` is deprecated, use `iteration_range` instead.
## [16:00:41] WARNING: src/c_api/c_api.cc:935: `ntree_limit` is deprecated, use `iteration_range` instead.
## [16:00:41] WARNING: src/c_api/c_api.cc:935: `ntree_limit` is deprecated, use `iteration_range` instead.
## [16:00:41] WARNING: src/c_api/c_api.cc:935: `ntree_limit` is deprecated, use `iteration_range` instead.
## [16:00:41] WARNING: src/c_api/c_api.cc:935: `ntree_limit` is deprecated, use `iteration_range` instead.
## [16:00:41] WARNING: src/c_api/c_api.cc:935: `ntree_limit` is deprecated, use `iteration_range` instead.
## [16:00:41] WARNING: src/c_api/c_api.cc:935: `ntree_limit` is deprecated, use `iteration_range` instead.
## [16:00:41] WARNING: src/c_api/c_api.cc:935: `ntree_limit` is deprecated, use `iteration_range` instead.
## [16:00:41] WARNING: src/c_api/c_api.cc:935: `ntree_limit` is deprecated, use `iteration_range` instead.
## [16:00:41] WARNING: src/c_api/c_api.cc:935: `ntree_limit` is deprecated, use `iteration_range` instead.
## [16:00:41] WARNING: src/c_api/c_api.cc:935: `ntree_limit` is deprecated, use `iteration_range` instead.
## [16:00:42] WARNING: src/c_api/c_api.cc:935: `ntree_limit` is deprecated, use `iteration_range` instead.
## [16:00:42] WARNING: src/c_api/c_api.cc:935: `ntree_limit` is deprecated, use `iteration_range` instead.
## [16:00:42] WARNING: src/c_api/c_api.cc:935: `ntree_limit` is deprecated, use `iteration_range` instead.
## [16:00:42] WARNING: src/c_api/c_api.cc:935: `ntree_limit` is deprecated, use `iteration_range` instead.
## [16:00:42] WARNING: src/c_api/c_api.cc:935: `ntree_limit` is deprecated, use `iteration_range` instead.
## [16:00:42] WARNING: src/c_api/c_api.cc:935: `ntree_limit` is deprecated, use `iteration_range` instead.
## [16:00:42] WARNING: src/c_api/c_api.cc:935: `ntree_limit` is deprecated, use `iteration_range` instead.
## [16:00:42] WARNING: src/c_api/c_api.cc:935: `ntree_limit` is deprecated, use `iteration_range` instead.
## [16:00:42] WARNING: src/c_api/c_api.cc:935: `ntree_limit` is deprecated, use `iteration_range` instead.
## [16:00:42] WARNING: src/c_api/c_api.cc:935: `ntree_limit` is deprecated, use `iteration_range` instead.
## [16:00:42] WARNING: src/c_api/c_api.cc:935: `ntree_limit` is deprecated, use `iteration_range` instead.
## [16:00:42] WARNING: src/c_api/c_api.cc:935: `ntree_limit` is deprecated, use `iteration_range` instead.
## [16:00:43] WARNING: src/c_api/c_api.cc:935: `ntree_limit` is deprecated, use `iteration_range` instead.
## [16:00:43] WARNING: src/c_api/c_api.cc:935: `ntree_limit` is deprecated, use `iteration_range` instead.
## [16:00:43] WARNING: src/c_api/c_api.cc:935: `ntree_limit` is deprecated, use `iteration_range` instead.
## [16:00:43] WARNING: src/c_api/c_api.cc:935: `ntree_limit` is deprecated, use `iteration_range` instead.
## [16:00:43] WARNING: src/c_api/c_api.cc:935: `ntree_limit` is deprecated, use `iteration_range` instead.
## [16:00:43] WARNING: src/c_api/c_api.cc:935: `ntree_limit` is deprecated, use `iteration_range` instead.
## [16:00:43] WARNING: src/c_api/c_api.cc:935: `ntree_limit` is deprecated, use `iteration_range` instead.
## [16:00:43] WARNING: src/c_api/c_api.cc:935: `ntree_limit` is deprecated, use `iteration_range` instead.
## [16:00:43] WARNING: src/c_api/c_api.cc:935: `ntree_limit` is deprecated, use `iteration_range` instead.
## [16:00:43] WARNING: src/c_api/c_api.cc:935: `ntree_limit` is deprecated, use `iteration_range` instead.
## [16:00:43] WARNING: src/c_api/c_api.cc:935: `ntree_limit` is deprecated, use `iteration_range` instead.
## [16:00:43] WARNING: src/c_api/c_api.cc:935: `ntree_limit` is deprecated, use `iteration_range` instead.
## [16:00:44] WARNING: src/c_api/c_api.cc:935: `ntree_limit` is deprecated, use `iteration_range` instead.
## [16:00:44] WARNING: src/c_api/c_api.cc:935: `ntree_limit` is deprecated, use `iteration_range` instead.
## [16:00:44] WARNING: src/c_api/c_api.cc:935: `ntree_limit` is deprecated, use `iteration_range` instead.
## [16:00:44] WARNING: src/c_api/c_api.cc:935: `ntree_limit` is deprecated, use `iteration_range` instead.
## [16:00:44] WARNING: src/c_api/c_api.cc:935: `ntree_limit` is deprecated, use `iteration_range` instead.
## [16:00:44] WARNING: src/c_api/c_api.cc:935: `ntree_limit` is deprecated, use `iteration_range` instead.
## [16:00:44] WARNING: src/c_api/c_api.cc:935: `ntree_limit` is deprecated, use `iteration_range` instead.
## [16:00:44] WARNING: src/c_api/c_api.cc:935: `ntree_limit` is deprecated, use `iteration_range` instead.
## [16:00:44] WARNING: src/c_api/c_api.cc:935: `ntree_limit` is deprecated, use `iteration_range` instead.
## [16:00:44] WARNING: src/c_api/c_api.cc:935: `ntree_limit` is deprecated, use `iteration_range` instead.
## [16:00:44] WARNING: src/c_api/c_api.cc:935: `ntree_limit` is deprecated, use `iteration_range` instead.
## [16:00:44] WARNING: src/c_api/c_api.cc:935: `ntree_limit` is deprecated, use `iteration_range` instead.
## [16:00:45] WARNING: src/c_api/c_api.cc:935: `ntree_limit` is deprecated, use `iteration_range` instead.
## [16:00:45] WARNING: src/c_api/c_api.cc:935: `ntree_limit` is deprecated, use `iteration_range` instead.
## [16:00:45] WARNING: src/c_api/c_api.cc:935: `ntree_limit` is deprecated, use `iteration_range` instead.
## [16:00:45] WARNING: src/c_api/c_api.cc:935: `ntree_limit` is deprecated, use `iteration_range` instead.
## [16:00:45] WARNING: src/c_api/c_api.cc:935: `ntree_limit` is deprecated, use `iteration_range` instead.
## [16:00:45] WARNING: src/c_api/c_api.cc:935: `ntree_limit` is deprecated, use `iteration_range` instead.
## [16:00:45] WARNING: src/c_api/c_api.cc:935: `ntree_limit` is deprecated, use `iteration_range` instead.
## [16:00:45] WARNING: src/c_api/c_api.cc:935: `ntree_limit` is deprecated, use `iteration_range` instead.
## [16:00:45] WARNING: src/c_api/c_api.cc:935: `ntree_limit` is deprecated, use `iteration_range` instead.
## [16:00:45] WARNING: src/c_api/c_api.cc:935: `ntree_limit` is deprecated, use `iteration_range` instead.
## [16:00:45] WARNING: src/c_api/c_api.cc:935: `ntree_limit` is deprecated, use `iteration_range` instead.
## [16:00:45] WARNING: src/c_api/c_api.cc:935: `ntree_limit` is deprecated, use `iteration_range` instead.
## [16:00:46] WARNING: src/c_api/c_api.cc:935: `ntree_limit` is deprecated, use `iteration_range` instead.
## [16:00:46] WARNING: src/c_api/c_api.cc:935: `ntree_limit` is deprecated, use `iteration_range` instead.
## [16:00:46] WARNING: src/c_api/c_api.cc:935: `ntree_limit` is deprecated, use `iteration_range` instead.
## [16:00:46] WARNING: src/c_api/c_api.cc:935: `ntree_limit` is deprecated, use `iteration_range` instead.
## [16:00:46] WARNING: src/c_api/c_api.cc:935: `ntree_limit` is deprecated, use `iteration_range` instead.
## [16:00:46] WARNING: src/c_api/c_api.cc:935: `ntree_limit` is deprecated, use `iteration_range` instead.
## [16:00:46] WARNING: src/c_api/c_api.cc:935: `ntree_limit` is deprecated, use `iteration_range` instead.
## [16:00:46] WARNING: src/c_api/c_api.cc:935: `ntree_limit` is deprecated, use `iteration_range` instead.
## [16:00:46] WARNING: src/c_api/c_api.cc:935: `ntree_limit` is deprecated, use `iteration_range` instead.
## [16:00:46] WARNING: src/c_api/c_api.cc:935: `ntree_limit` is deprecated, use `iteration_range` instead.
## [16:00:46] WARNING: src/c_api/c_api.cc:935: `ntree_limit` is deprecated, use `iteration_range` instead.
## [16:00:46] WARNING: src/c_api/c_api.cc:935: `ntree_limit` is deprecated, use `iteration_range` instead.
## [16:00:47] WARNING: src/c_api/c_api.cc:935: `ntree_limit` is deprecated, use `iteration_range` instead.
## [16:00:47] WARNING: src/c_api/c_api.cc:935: `ntree_limit` is deprecated, use `iteration_range` instead.
## [16:00:47] WARNING: src/c_api/c_api.cc:935: `ntree_limit` is deprecated, use `iteration_range` instead.
## [16:00:47] WARNING: src/c_api/c_api.cc:935: `ntree_limit` is deprecated, use `iteration_range` instead.
## [16:00:47] WARNING: src/c_api/c_api.cc:935: `ntree_limit` is deprecated, use `iteration_range` instead.
## [16:00:47] WARNING: src/c_api/c_api.cc:935: `ntree_limit` is deprecated, use `iteration_range` instead.
## [16:00:47] WARNING: src/c_api/c_api.cc:935: `ntree_limit` is deprecated, use `iteration_range` instead.
## [16:00:47] WARNING: src/c_api/c_api.cc:935: `ntree_limit` is deprecated, use `iteration_range` instead.
## [16:00:47] WARNING: src/c_api/c_api.cc:935: `ntree_limit` is deprecated, use `iteration_range` instead.
## [16:00:47] WARNING: src/c_api/c_api.cc:935: `ntree_limit` is deprecated, use `iteration_range` instead.
## [16:00:48] WARNING: src/c_api/c_api.cc:935: `ntree_limit` is deprecated, use `iteration_range` instead.
## [16:00:48] WARNING: src/c_api/c_api.cc:935: `ntree_limit` is deprecated, use `iteration_range` instead.
## [16:00:48] WARNING: src/c_api/c_api.cc:935: `ntree_limit` is deprecated, use `iteration_range` instead.
## [16:00:48] WARNING: src/c_api/c_api.cc:935: `ntree_limit` is deprecated, use `iteration_range` instead.
## [16:00:48] WARNING: src/c_api/c_api.cc:935: `ntree_limit` is deprecated, use `iteration_range` instead.
## [16:00:48] WARNING: src/c_api/c_api.cc:935: `ntree_limit` is deprecated, use `iteration_range` instead.
## [16:00:48] WARNING: src/c_api/c_api.cc:935: `ntree_limit` is deprecated, use `iteration_range` instead.
## [16:00:48] WARNING: src/c_api/c_api.cc:935: `ntree_limit` is deprecated, use `iteration_range` instead.
## [16:00:48] WARNING: src/c_api/c_api.cc:935: `ntree_limit` is deprecated, use `iteration_range` instead.
## [16:00:48] WARNING: src/c_api/c_api.cc:935: `ntree_limit` is deprecated, use `iteration_range` instead.
## [16:00:48] WARNING: src/c_api/c_api.cc:935: `ntree_limit` is deprecated, use `iteration_range` instead.
## [16:00:48] WARNING: src/c_api/c_api.cc:935: `ntree_limit` is deprecated, use `iteration_range` instead.
## [16:00:49] WARNING: src/c_api/c_api.cc:935: `ntree_limit` is deprecated, use `iteration_range` instead.
## [16:00:49] WARNING: src/c_api/c_api.cc:935: `ntree_limit` is deprecated, use `iteration_range` instead.
## [16:00:49] WARNING: src/c_api/c_api.cc:935: `ntree_limit` is deprecated, use `iteration_range` instead.
## [16:00:49] WARNING: src/c_api/c_api.cc:935: `ntree_limit` is deprecated, use `iteration_range` instead.
## [16:00:49] WARNING: src/c_api/c_api.cc:935: `ntree_limit` is deprecated, use `iteration_range` instead.
## [16:00:49] WARNING: src/c_api/c_api.cc:935: `ntree_limit` is deprecated, use `iteration_range` instead.
## [16:00:49] WARNING: src/c_api/c_api.cc:935: `ntree_limit` is deprecated, use `iteration_range` instead.
## [16:00:49] WARNING: src/c_api/c_api.cc:935: `ntree_limit` is deprecated, use `iteration_range` instead.
## [16:00:49] WARNING: src/c_api/c_api.cc:935: `ntree_limit` is deprecated, use `iteration_range` instead.
## [16:00:49] WARNING: src/c_api/c_api.cc:935: `ntree_limit` is deprecated, use `iteration_range` instead.
## [16:00:49] WARNING: src/c_api/c_api.cc:935: `ntree_limit` is deprecated, use `iteration_range` instead.
## [16:00:49] WARNING: src/c_api/c_api.cc:935: `ntree_limit` is deprecated, use `iteration_range` instead.
## [16:00:50] WARNING: src/c_api/c_api.cc:935: `ntree_limit` is deprecated, use `iteration_range` instead.
## [16:00:50] WARNING: src/c_api/c_api.cc:935: `ntree_limit` is deprecated, use `iteration_range` instead.
## [16:00:50] WARNING: src/c_api/c_api.cc:935: `ntree_limit` is deprecated, use `iteration_range` instead.
## [16:00:50] WARNING: src/c_api/c_api.cc:935: `ntree_limit` is deprecated, use `iteration_range` instead.
## [16:00:50] WARNING: src/c_api/c_api.cc:935: `ntree_limit` is deprecated, use `iteration_range` instead.
## [16:00:50] WARNING: src/c_api/c_api.cc:935: `ntree_limit` is deprecated, use `iteration_range` instead.
## [16:00:50] WARNING: src/c_api/c_api.cc:935: `ntree_limit` is deprecated, use `iteration_range` instead.
## [16:00:50] WARNING: src/c_api/c_api.cc:935: `ntree_limit` is deprecated, use `iteration_range` instead.
## [16:00:50] WARNING: src/c_api/c_api.cc:935: `ntree_limit` is deprecated, use `iteration_range` instead.
## [16:00:50] WARNING: src/c_api/c_api.cc:935: `ntree_limit` is deprecated, use `iteration_range` instead.
## [16:00:50] WARNING: src/c_api/c_api.cc:935: `ntree_limit` is deprecated, use `iteration_range` instead.
## [16:00:50] WARNING: src/c_api/c_api.cc:935: `ntree_limit` is deprecated, use `iteration_range` instead.
## [16:00:51] WARNING: src/c_api/c_api.cc:935: `ntree_limit` is deprecated, use `iteration_range` instead.
## [16:00:51] WARNING: src/c_api/c_api.cc:935: `ntree_limit` is deprecated, use `iteration_range` instead.
## [16:00:51] WARNING: src/c_api/c_api.cc:935: `ntree_limit` is deprecated, use `iteration_range` instead.
## [16:00:51] WARNING: src/c_api/c_api.cc:935: `ntree_limit` is deprecated, use `iteration_range` instead.
## [16:00:51] WARNING: src/c_api/c_api.cc:935: `ntree_limit` is deprecated, use `iteration_range` instead.
## [16:00:51] WARNING: src/c_api/c_api.cc:935: `ntree_limit` is deprecated, use `iteration_range` instead.
## [16:00:51] WARNING: src/c_api/c_api.cc:935: `ntree_limit` is deprecated, use `iteration_range` instead.
## [16:00:51] WARNING: src/c_api/c_api.cc:935: `ntree_limit` is deprecated, use `iteration_range` instead.
## [16:00:51] WARNING: src/c_api/c_api.cc:935: `ntree_limit` is deprecated, use `iteration_range` instead.
## [16:00:51] WARNING: src/c_api/c_api.cc:935: `ntree_limit` is deprecated, use `iteration_range` instead.
## [16:00:52] WARNING: src/c_api/c_api.cc:935: `ntree_limit` is deprecated, use `iteration_range` instead.
## [16:00:52] WARNING: src/c_api/c_api.cc:935: `ntree_limit` is deprecated, use `iteration_range` instead.
## [16:00:52] WARNING: src/c_api/c_api.cc:935: `ntree_limit` is deprecated, use `iteration_range` instead.
## [16:00:52] WARNING: src/c_api/c_api.cc:935: `ntree_limit` is deprecated, use `iteration_range` instead.
## [16:00:52] WARNING: src/c_api/c_api.cc:935: `ntree_limit` is deprecated, use `iteration_range` instead.
## [16:00:52] WARNING: src/c_api/c_api.cc:935: `ntree_limit` is deprecated, use `iteration_range` instead.
## [16:00:52] WARNING: src/c_api/c_api.cc:935: `ntree_limit` is deprecated, use `iteration_range` instead.
## [16:00:52] WARNING: src/c_api/c_api.cc:935: `ntree_limit` is deprecated, use `iteration_range` instead.
## [16:00:52] WARNING: src/c_api/c_api.cc:935: `ntree_limit` is deprecated, use `iteration_range` instead.
## [16:00:52] WARNING: src/c_api/c_api.cc:935: `ntree_limit` is deprecated, use `iteration_range` instead.
## [16:00:52] WARNING: src/c_api/c_api.cc:935: `ntree_limit` is deprecated, use `iteration_range` instead.
## [16:00:52] WARNING: src/c_api/c_api.cc:935: `ntree_limit` is deprecated, use `iteration_range` instead.
## [16:00:52] WARNING: src/c_api/c_api.cc:935: `ntree_limit` is deprecated, use `iteration_range` instead.
## [16:00:52] WARNING: src/c_api/c_api.cc:935: `ntree_limit` is deprecated, use `iteration_range` instead.
## [16:00:53] WARNING: src/c_api/c_api.cc:935: `ntree_limit` is deprecated, use `iteration_range` instead.
## [16:00:53] WARNING: src/c_api/c_api.cc:935: `ntree_limit` is deprecated, use `iteration_range` instead.
## [16:00:53] WARNING: src/c_api/c_api.cc:935: `ntree_limit` is deprecated, use `iteration_range` instead.
## [16:00:53] WARNING: src/c_api/c_api.cc:935: `ntree_limit` is deprecated, use `iteration_range` instead.
## [16:00:53] WARNING: src/c_api/c_api.cc:935: `ntree_limit` is deprecated, use `iteration_range` instead.
## [16:00:53] WARNING: src/c_api/c_api.cc:935: `ntree_limit` is deprecated, use `iteration_range` instead.
## [16:00:53] WARNING: src/c_api/c_api.cc:935: `ntree_limit` is deprecated, use `iteration_range` instead.
## [16:00:53] WARNING: src/c_api/c_api.cc:935: `ntree_limit` is deprecated, use `iteration_range` instead.
## [16:00:53] WARNING: src/c_api/c_api.cc:935: `ntree_limit` is deprecated, use `iteration_range` instead.
## [16:00:53] WARNING: src/c_api/c_api.cc:935: `ntree_limit` is deprecated, use `iteration_range` instead.
## [16:00:54] WARNING: src/c_api/c_api.cc:935: `ntree_limit` is deprecated, use `iteration_range` instead.
## [16:00:54] WARNING: src/c_api/c_api.cc:935: `ntree_limit` is deprecated, use `iteration_range` instead.
## [16:00:54] WARNING: src/c_api/c_api.cc:935: `ntree_limit` is deprecated, use `iteration_range` instead.
## [16:00:54] WARNING: src/c_api/c_api.cc:935: `ntree_limit` is deprecated, use `iteration_range` instead.
## [16:00:54] WARNING: src/c_api/c_api.cc:935: `ntree_limit` is deprecated, use `iteration_range` instead.
## [16:00:54] WARNING: src/c_api/c_api.cc:935: `ntree_limit` is deprecated, use `iteration_range` instead.
## [16:00:54] WARNING: src/c_api/c_api.cc:935: `ntree_limit` is deprecated, use `iteration_range` instead.
## [16:00:54] WARNING: src/c_api/c_api.cc:935: `ntree_limit` is deprecated, use `iteration_range` instead.
## [16:00:54] WARNING: src/c_api/c_api.cc:935: `ntree_limit` is deprecated, use `iteration_range` instead.
## [16:00:54] WARNING: src/c_api/c_api.cc:935: `ntree_limit` is deprecated, use `iteration_range` instead.
## [16:00:55] WARNING: src/c_api/c_api.cc:935: `ntree_limit` is deprecated, use `iteration_range` instead.
## [16:00:55] WARNING: src/c_api/c_api.cc:935: `ntree_limit` is deprecated, use `iteration_range` instead.
## [16:00:55] WARNING: src/c_api/c_api.cc:935: `ntree_limit` is deprecated, use `iteration_range` instead.
## [16:00:55] WARNING: src/c_api/c_api.cc:935: `ntree_limit` is deprecated, use `iteration_range` instead.
## [16:00:55] WARNING: src/c_api/c_api.cc:935: `ntree_limit` is deprecated, use `iteration_range` instead.
## [16:00:55] WARNING: src/c_api/c_api.cc:935: `ntree_limit` is deprecated, use `iteration_range` instead.
## [16:00:55] WARNING: src/c_api/c_api.cc:935: `ntree_limit` is deprecated, use `iteration_range` instead.
## [16:00:55] WARNING: src/c_api/c_api.cc:935: `ntree_limit` is deprecated, use `iteration_range` instead.
## [16:00:55] WARNING: src/c_api/c_api.cc:935: `ntree_limit` is deprecated, use `iteration_range` instead.
## [16:00:55] WARNING: src/c_api/c_api.cc:935: `ntree_limit` is deprecated, use `iteration_range` instead.
## [16:00:55] WARNING: src/c_api/c_api.cc:935: `ntree_limit` is deprecated, use `iteration_range` instead.
## [16:00:55] WARNING: src/c_api/c_api.cc:935: `ntree_limit` is deprecated, use `iteration_range` instead.
## [16:00:55] WARNING: src/c_api/c_api.cc:935: `ntree_limit` is deprecated, use `iteration_range` instead.
## [16:00:55] WARNING: src/c_api/c_api.cc:935: `ntree_limit` is deprecated, use `iteration_range` instead.
## [16:00:56] WARNING: src/c_api/c_api.cc:935: `ntree_limit` is deprecated, use `iteration_range` instead.
## [16:00:56] WARNING: src/c_api/c_api.cc:935: `ntree_limit` is deprecated, use `iteration_range` instead.
## [16:00:56] WARNING: src/c_api/c_api.cc:935: `ntree_limit` is deprecated, use `iteration_range` instead.
## [16:00:56] WARNING: src/c_api/c_api.cc:935: `ntree_limit` is deprecated, use `iteration_range` instead.
## [16:00:56] WARNING: src/c_api/c_api.cc:935: `ntree_limit` is deprecated, use `iteration_range` instead.
## [16:00:56] WARNING: src/c_api/c_api.cc:935: `ntree_limit` is deprecated, use `iteration_range` instead.
## [16:00:56] WARNING: src/c_api/c_api.cc:935: `ntree_limit` is deprecated, use `iteration_range` instead.
## [16:00:56] WARNING: src/c_api/c_api.cc:935: `ntree_limit` is deprecated, use `iteration_range` instead.
## [16:00:56] WARNING: src/c_api/c_api.cc:935: `ntree_limit` is deprecated, use `iteration_range` instead.
## [16:00:56] WARNING: src/c_api/c_api.cc:935: `ntree_limit` is deprecated, use `iteration_range` instead.
## [16:00:56] WARNING: src/c_api/c_api.cc:935: `ntree_limit` is deprecated, use `iteration_range` instead.
## [16:00:56] WARNING: src/c_api/c_api.cc:935: `ntree_limit` is deprecated, use `iteration_range` instead.
## [16:00:57] WARNING: src/c_api/c_api.cc:935: `ntree_limit` is deprecated, use `iteration_range` instead.
## [16:00:57] WARNING: src/c_api/c_api.cc:935: `ntree_limit` is deprecated, use `iteration_range` instead.
## [16:00:57] WARNING: src/c_api/c_api.cc:935: `ntree_limit` is deprecated, use `iteration_range` instead.
## [16:00:57] WARNING: src/c_api/c_api.cc:935: `ntree_limit` is deprecated, use `iteration_range` instead.
## [16:00:57] WARNING: src/c_api/c_api.cc:935: `ntree_limit` is deprecated, use `iteration_range` instead.
## [16:00:57] WARNING: src/c_api/c_api.cc:935: `ntree_limit` is deprecated, use `iteration_range` instead.
## [16:00:57] WARNING: src/c_api/c_api.cc:935: `ntree_limit` is deprecated, use `iteration_range` instead.
## [16:00:57] WARNING: src/c_api/c_api.cc:935: `ntree_limit` is deprecated, use `iteration_range` instead.
## [16:00:57] WARNING: src/c_api/c_api.cc:935: `ntree_limit` is deprecated, use `iteration_range` instead.
## [16:00:57] WARNING: src/c_api/c_api.cc:935: `ntree_limit` is deprecated, use `iteration_range` instead.
## [16:01:00] WARNING: src/c_api/c_api.cc:935: `ntree_limit` is deprecated, use `iteration_range` instead.
## [16:01:00] WARNING: src/c_api/c_api.cc:935: `ntree_limit` is deprecated, use `iteration_range` instead.
## [16:01:00] WARNING: src/c_api/c_api.cc:935: `ntree_limit` is deprecated, use `iteration_range` instead.
## [16:01:00] WARNING: src/c_api/c_api.cc:935: `ntree_limit` is deprecated, use `iteration_range` instead.
## [16:01:01] WARNING: src/c_api/c_api.cc:935: `ntree_limit` is deprecated, use `iteration_range` instead.
## [16:01:01] WARNING: src/c_api/c_api.cc:935: `ntree_limit` is deprecated, use `iteration_range` instead.
## [16:01:01] WARNING: src/c_api/c_api.cc:935: `ntree_limit` is deprecated, use `iteration_range` instead.
## [16:01:01] WARNING: src/c_api/c_api.cc:935: `ntree_limit` is deprecated, use `iteration_range` instead.
## [16:01:01] WARNING: src/c_api/c_api.cc:935: `ntree_limit` is deprecated, use `iteration_range` instead.
## [16:01:01] WARNING: src/c_api/c_api.cc:935: `ntree_limit` is deprecated, use `iteration_range` instead.
## [16:01:01] WARNING: src/c_api/c_api.cc:935: `ntree_limit` is deprecated, use `iteration_range` instead.
## [16:01:01] WARNING: src/c_api/c_api.cc:935: `ntree_limit` is deprecated, use `iteration_range` instead.
## [16:01:01] WARNING: src/c_api/c_api.cc:935: `ntree_limit` is deprecated, use `iteration_range` instead.
## [16:01:01] WARNING: src/c_api/c_api.cc:935: `ntree_limit` is deprecated, use `iteration_range` instead.
## [16:01:01] WARNING: src/c_api/c_api.cc:935: `ntree_limit` is deprecated, use `iteration_range` instead.
## [16:01:01] WARNING: src/c_api/c_api.cc:935: `ntree_limit` is deprecated, use `iteration_range` instead.
## [16:01:02] WARNING: src/c_api/c_api.cc:935: `ntree_limit` is deprecated, use `iteration_range` instead.
## [16:01:02] WARNING: src/c_api/c_api.cc:935: `ntree_limit` is deprecated, use `iteration_range` instead.
## [16:01:02] WARNING: src/c_api/c_api.cc:935: `ntree_limit` is deprecated, use `iteration_range` instead.
## [16:01:02] WARNING: src/c_api/c_api.cc:935: `ntree_limit` is deprecated, use `iteration_range` instead.
## [16:01:02] WARNING: src/c_api/c_api.cc:935: `ntree_limit` is deprecated, use `iteration_range` instead.
## [16:01:02] WARNING: src/c_api/c_api.cc:935: `ntree_limit` is deprecated, use `iteration_range` instead.
## [16:01:02] WARNING: src/c_api/c_api.cc:935: `ntree_limit` is deprecated, use `iteration_range` instead.
## [16:01:02] WARNING: src/c_api/c_api.cc:935: `ntree_limit` is deprecated, use `iteration_range` instead.
## [16:01:02] WARNING: src/c_api/c_api.cc:935: `ntree_limit` is deprecated, use `iteration_range` instead.
## [16:01:02] WARNING: src/c_api/c_api.cc:935: `ntree_limit` is deprecated, use `iteration_range` instead.
## [16:01:02] WARNING: src/c_api/c_api.cc:935: `ntree_limit` is deprecated, use `iteration_range` instead.
## [16:01:02] WARNING: src/c_api/c_api.cc:935: `ntree_limit` is deprecated, use `iteration_range` instead.
## [16:01:03] WARNING: src/c_api/c_api.cc:935: `ntree_limit` is deprecated, use `iteration_range` instead.
## [16:01:03] WARNING: src/c_api/c_api.cc:935: `ntree_limit` is deprecated, use `iteration_range` instead.
## [16:01:03] WARNING: src/c_api/c_api.cc:935: `ntree_limit` is deprecated, use `iteration_range` instead.
## [16:01:03] WARNING: src/c_api/c_api.cc:935: `ntree_limit` is deprecated, use `iteration_range` instead.
## [16:01:03] WARNING: src/c_api/c_api.cc:935: `ntree_limit` is deprecated, use `iteration_range` instead.
## [16:01:03] WARNING: src/c_api/c_api.cc:935: `ntree_limit` is deprecated, use `iteration_range` instead.
## [16:01:03] WARNING: src/c_api/c_api.cc:935: `ntree_limit` is deprecated, use `iteration_range` instead.
## [16:01:03] WARNING: src/c_api/c_api.cc:935: `ntree_limit` is deprecated, use `iteration_range` instead.
## [16:01:03] WARNING: src/c_api/c_api.cc:935: `ntree_limit` is deprecated, use `iteration_range` instead.
## [16:01:03] WARNING: src/c_api/c_api.cc:935: `ntree_limit` is deprecated, use `iteration_range` instead.
## [16:01:04] WARNING: src/c_api/c_api.cc:935: `ntree_limit` is deprecated, use `iteration_range` instead.
## [16:01:04] WARNING: src/c_api/c_api.cc:935: `ntree_limit` is deprecated, use `iteration_range` instead.
## [16:01:04] WARNING: src/c_api/c_api.cc:935: `ntree_limit` is deprecated, use `iteration_range` instead.
## [16:01:04] WARNING: src/c_api/c_api.cc:935: `ntree_limit` is deprecated, use `iteration_range` instead.
## [16:01:04] WARNING: src/c_api/c_api.cc:935: `ntree_limit` is deprecated, use `iteration_range` instead.
## [16:01:04] WARNING: src/c_api/c_api.cc:935: `ntree_limit` is deprecated, use `iteration_range` instead.
## [16:01:04] WARNING: src/c_api/c_api.cc:935: `ntree_limit` is deprecated, use `iteration_range` instead.
## [16:01:04] WARNING: src/c_api/c_api.cc:935: `ntree_limit` is deprecated, use `iteration_range` instead.
## [16:01:04] WARNING: src/c_api/c_api.cc:935: `ntree_limit` is deprecated, use `iteration_range` instead.
## [16:01:04] WARNING: src/c_api/c_api.cc:935: `ntree_limit` is deprecated, use `iteration_range` instead.
## [16:01:04] WARNING: src/c_api/c_api.cc:935: `ntree_limit` is deprecated, use `iteration_range` instead.
## [16:01:04] WARNING: src/c_api/c_api.cc:935: `ntree_limit` is deprecated, use `iteration_range` instead.
## [16:01:04] WARNING: src/c_api/c_api.cc:935: `ntree_limit` is deprecated, use `iteration_range` instead.
## [16:01:04] WARNING: src/c_api/c_api.cc:935: `ntree_limit` is deprecated, use `iteration_range` instead.
## [16:01:04] WARNING: src/c_api/c_api.cc:935: `ntree_limit` is deprecated, use `iteration_range` instead.
## [16:01:04] WARNING: src/c_api/c_api.cc:935: `ntree_limit` is deprecated, use `iteration_range` instead.
## [16:01:05] WARNING: src/c_api/c_api.cc:935: `ntree_limit` is deprecated, use `iteration_range` instead.
## [16:01:05] WARNING: src/c_api/c_api.cc:935: `ntree_limit` is deprecated, use `iteration_range` instead.
## [16:01:05] WARNING: src/c_api/c_api.cc:935: `ntree_limit` is deprecated, use `iteration_range` instead.
## [16:01:05] WARNING: src/c_api/c_api.cc:935: `ntree_limit` is deprecated, use `iteration_range` instead.
## [16:01:05] WARNING: src/c_api/c_api.cc:935: `ntree_limit` is deprecated, use `iteration_range` instead.
## [16:01:05] WARNING: src/c_api/c_api.cc:935: `ntree_limit` is deprecated, use `iteration_range` instead.
## [16:01:05] WARNING: src/c_api/c_api.cc:935: `ntree_limit` is deprecated, use `iteration_range` instead.
## [16:01:05] WARNING: src/c_api/c_api.cc:935: `ntree_limit` is deprecated, use `iteration_range` instead.
## [16:01:05] WARNING: src/c_api/c_api.cc:935: `ntree_limit` is deprecated, use `iteration_range` instead.
## [16:01:05] WARNING: src/c_api/c_api.cc:935: `ntree_limit` is deprecated, use `iteration_range` instead.
## [16:01:06] WARNING: src/c_api/c_api.cc:935: `ntree_limit` is deprecated, use `iteration_range` instead.
## [16:01:06] WARNING: src/c_api/c_api.cc:935: `ntree_limit` is deprecated, use `iteration_range` instead.
## [16:01:06] WARNING: src/c_api/c_api.cc:935: `ntree_limit` is deprecated, use `iteration_range` instead.
## [16:01:06] WARNING: src/c_api/c_api.cc:935: `ntree_limit` is deprecated, use `iteration_range` instead.
## [16:01:06] WARNING: src/c_api/c_api.cc:935: `ntree_limit` is deprecated, use `iteration_range` instead.
## [16:01:06] WARNING: src/c_api/c_api.cc:935: `ntree_limit` is deprecated, use `iteration_range` instead.
## [16:01:06] WARNING: src/c_api/c_api.cc:935: `ntree_limit` is deprecated, use `iteration_range` instead.
## [16:01:06] WARNING: src/c_api/c_api.cc:935: `ntree_limit` is deprecated, use `iteration_range` instead.
## [16:01:06] WARNING: src/c_api/c_api.cc:935: `ntree_limit` is deprecated, use `iteration_range` instead.
## [16:01:06] WARNING: src/c_api/c_api.cc:935: `ntree_limit` is deprecated, use `iteration_range` instead.
## [16:01:06] WARNING: src/c_api/c_api.cc:935: `ntree_limit` is deprecated, use `iteration_range` instead.
## [16:01:06] WARNING: src/c_api/c_api.cc:935: `ntree_limit` is deprecated, use `iteration_range` instead.
## [16:01:07] WARNING: src/c_api/c_api.cc:935: `ntree_limit` is deprecated, use `iteration_range` instead.
## [16:01:07] WARNING: src/c_api/c_api.cc:935: `ntree_limit` is deprecated, use `iteration_range` instead.
## [16:01:07] WARNING: src/c_api/c_api.cc:935: `ntree_limit` is deprecated, use `iteration_range` instead.
## [16:01:07] WARNING: src/c_api/c_api.cc:935: `ntree_limit` is deprecated, use `iteration_range` instead.
## [16:01:07] WARNING: src/c_api/c_api.cc:935: `ntree_limit` is deprecated, use `iteration_range` instead.
## [16:01:07] WARNING: src/c_api/c_api.cc:935: `ntree_limit` is deprecated, use `iteration_range` instead.
## [16:01:07] WARNING: src/c_api/c_api.cc:935: `ntree_limit` is deprecated, use `iteration_range` instead.
## [16:01:07] WARNING: src/c_api/c_api.cc:935: `ntree_limit` is deprecated, use `iteration_range` instead.
## [16:01:07] WARNING: src/c_api/c_api.cc:935: `ntree_limit` is deprecated, use `iteration_range` instead.
## [16:01:07] WARNING: src/c_api/c_api.cc:935: `ntree_limit` is deprecated, use `iteration_range` instead.
## [16:01:07] WARNING: src/c_api/c_api.cc:935: `ntree_limit` is deprecated, use `iteration_range` instead.
## [16:01:07] WARNING: src/c_api/c_api.cc:935: `ntree_limit` is deprecated, use `iteration_range` instead.
## [16:01:08] WARNING: src/c_api/c_api.cc:935: `ntree_limit` is deprecated, use `iteration_range` instead.
## [16:01:08] WARNING: src/c_api/c_api.cc:935: `ntree_limit` is deprecated, use `iteration_range` instead.
## [16:01:08] WARNING: src/c_api/c_api.cc:935: `ntree_limit` is deprecated, use `iteration_range` instead.
## [16:01:08] WARNING: src/c_api/c_api.cc:935: `ntree_limit` is deprecated, use `iteration_range` instead.
## [16:01:08] WARNING: src/c_api/c_api.cc:935: `ntree_limit` is deprecated, use `iteration_range` instead.
## [16:01:08] WARNING: src/c_api/c_api.cc:935: `ntree_limit` is deprecated, use `iteration_range` instead.
## [16:01:08] WARNING: src/c_api/c_api.cc:935: `ntree_limit` is deprecated, use `iteration_range` instead.
## [16:01:08] WARNING: src/c_api/c_api.cc:935: `ntree_limit` is deprecated, use `iteration_range` instead.
## [16:01:08] WARNING: src/c_api/c_api.cc:935: `ntree_limit` is deprecated, use `iteration_range` instead.
## [16:01:08] WARNING: src/c_api/c_api.cc:935: `ntree_limit` is deprecated, use `iteration_range` instead.
## [16:01:08] WARNING: src/c_api/c_api.cc:935: `ntree_limit` is deprecated, use `iteration_range` instead.
## [16:01:08] WARNING: src/c_api/c_api.cc:935: `ntree_limit` is deprecated, use `iteration_range` instead.
## [16:01:09] WARNING: src/c_api/c_api.cc:935: `ntree_limit` is deprecated, use `iteration_range` instead.
## [16:01:09] WARNING: src/c_api/c_api.cc:935: `ntree_limit` is deprecated, use `iteration_range` instead.
## [16:01:09] WARNING: src/c_api/c_api.cc:935: `ntree_limit` is deprecated, use `iteration_range` instead.
## [16:01:09] WARNING: src/c_api/c_api.cc:935: `ntree_limit` is deprecated, use `iteration_range` instead.
## [16:01:09] WARNING: src/c_api/c_api.cc:935: `ntree_limit` is deprecated, use `iteration_range` instead.
## [16:01:09] WARNING: src/c_api/c_api.cc:935: `ntree_limit` is deprecated, use `iteration_range` instead.
## [16:01:09] WARNING: src/c_api/c_api.cc:935: `ntree_limit` is deprecated, use `iteration_range` instead.
## [16:01:09] WARNING: src/c_api/c_api.cc:935: `ntree_limit` is deprecated, use `iteration_range` instead.
## [16:01:09] WARNING: src/c_api/c_api.cc:935: `ntree_limit` is deprecated, use `iteration_range` instead.
## [16:01:09] WARNING: src/c_api/c_api.cc:935: `ntree_limit` is deprecated, use `iteration_range` instead.
## [16:01:10] WARNING: src/c_api/c_api.cc:935: `ntree_limit` is deprecated, use `iteration_range` instead.
## [16:01:10] WARNING: src/c_api/c_api.cc:935: `ntree_limit` is deprecated, use `iteration_range` instead.
## [16:01:10] WARNING: src/c_api/c_api.cc:935: `ntree_limit` is deprecated, use `iteration_range` instead.
## [16:01:10] WARNING: src/c_api/c_api.cc:935: `ntree_limit` is deprecated, use `iteration_range` instead.
## [16:01:10] WARNING: src/c_api/c_api.cc:935: `ntree_limit` is deprecated, use `iteration_range` instead.
## [16:01:10] WARNING: src/c_api/c_api.cc:935: `ntree_limit` is deprecated, use `iteration_range` instead.
## [16:01:10] WARNING: src/c_api/c_api.cc:935: `ntree_limit` is deprecated, use `iteration_range` instead.
## [16:01:10] WARNING: src/c_api/c_api.cc:935: `ntree_limit` is deprecated, use `iteration_range` instead.
## [16:01:10] WARNING: src/c_api/c_api.cc:935: `ntree_limit` is deprecated, use `iteration_range` instead.
## [16:01:10] WARNING: src/c_api/c_api.cc:935: `ntree_limit` is deprecated, use `iteration_range` instead.
## [16:01:10] WARNING: src/c_api/c_api.cc:935: `ntree_limit` is deprecated, use `iteration_range` instead.
## [16:01:10] WARNING: src/c_api/c_api.cc:935: `ntree_limit` is deprecated, use `iteration_range` instead.
## [16:01:10] WARNING: src/c_api/c_api.cc:935: `ntree_limit` is deprecated, use `iteration_range` instead.
## [16:01:10] WARNING: src/c_api/c_api.cc:935: `ntree_limit` is deprecated, use `iteration_range` instead.
## [16:01:10] WARNING: src/c_api/c_api.cc:935: `ntree_limit` is deprecated, use `iteration_range` instead.
## [16:01:11] WARNING: src/c_api/c_api.cc:935: `ntree_limit` is deprecated, use `iteration_range` instead.
## [16:01:11] WARNING: src/c_api/c_api.cc:935: `ntree_limit` is deprecated, use `iteration_range` instead.
## [16:01:11] WARNING: src/c_api/c_api.cc:935: `ntree_limit` is deprecated, use `iteration_range` instead.
## [16:01:11] WARNING: src/c_api/c_api.cc:935: `ntree_limit` is deprecated, use `iteration_range` instead.
## [16:01:11] WARNING: src/c_api/c_api.cc:935: `ntree_limit` is deprecated, use `iteration_range` instead.
## [16:01:11] WARNING: src/c_api/c_api.cc:935: `ntree_limit` is deprecated, use `iteration_range` instead.
## [16:01:11] WARNING: src/c_api/c_api.cc:935: `ntree_limit` is deprecated, use `iteration_range` instead.
## [16:01:11] WARNING: src/c_api/c_api.cc:935: `ntree_limit` is deprecated, use `iteration_range` instead.
## [16:01:11] WARNING: src/c_api/c_api.cc:935: `ntree_limit` is deprecated, use `iteration_range` instead.
## [16:01:11] WARNING: src/c_api/c_api.cc:935: `ntree_limit` is deprecated, use `iteration_range` instead.
## [16:01:11] WARNING: src/c_api/c_api.cc:935: `ntree_limit` is deprecated, use `iteration_range` instead.
## [16:01:12] WARNING: src/c_api/c_api.cc:935: `ntree_limit` is deprecated, use `iteration_range` instead.
## [16:01:12] WARNING: src/c_api/c_api.cc:935: `ntree_limit` is deprecated, use `iteration_range` instead.
## [16:01:12] WARNING: src/c_api/c_api.cc:935: `ntree_limit` is deprecated, use `iteration_range` instead.
## [16:01:12] WARNING: src/c_api/c_api.cc:935: `ntree_limit` is deprecated, use `iteration_range` instead.
## [16:01:12] WARNING: src/c_api/c_api.cc:935: `ntree_limit` is deprecated, use `iteration_range` instead.
## [16:01:12] WARNING: src/c_api/c_api.cc:935: `ntree_limit` is deprecated, use `iteration_range` instead.
## [16:01:12] WARNING: src/c_api/c_api.cc:935: `ntree_limit` is deprecated, use `iteration_range` instead.
## [16:01:12] WARNING: src/c_api/c_api.cc:935: `ntree_limit` is deprecated, use `iteration_range` instead.
## [16:01:12] WARNING: src/c_api/c_api.cc:935: `ntree_limit` is deprecated, use `iteration_range` instead.
## [16:01:12] WARNING: src/c_api/c_api.cc:935: `ntree_limit` is deprecated, use `iteration_range` instead.
## [16:01:12] WARNING: src/c_api/c_api.cc:935: `ntree_limit` is deprecated, use `iteration_range` instead.
## [16:01:12] WARNING: src/c_api/c_api.cc:935: `ntree_limit` is deprecated, use `iteration_range` instead.
## [16:01:13] WARNING: src/c_api/c_api.cc:935: `ntree_limit` is deprecated, use `iteration_range` instead.
## [16:01:13] WARNING: src/c_api/c_api.cc:935: `ntree_limit` is deprecated, use `iteration_range` instead.
## [16:01:13] WARNING: src/c_api/c_api.cc:935: `ntree_limit` is deprecated, use `iteration_range` instead.
## [16:01:13] WARNING: src/c_api/c_api.cc:935: `ntree_limit` is deprecated, use `iteration_range` instead.
## [16:01:13] WARNING: src/c_api/c_api.cc:935: `ntree_limit` is deprecated, use `iteration_range` instead.
## [16:01:13] WARNING: src/c_api/c_api.cc:935: `ntree_limit` is deprecated, use `iteration_range` instead.
## [16:01:13] WARNING: src/c_api/c_api.cc:935: `ntree_limit` is deprecated, use `iteration_range` instead.
## [16:01:13] WARNING: src/c_api/c_api.cc:935: `ntree_limit` is deprecated, use `iteration_range` instead.
## [16:01:13] WARNING: src/c_api/c_api.cc:935: `ntree_limit` is deprecated, use `iteration_range` instead.
## [16:01:13] WARNING: src/c_api/c_api.cc:935: `ntree_limit` is deprecated, use `iteration_range` instead.
## [16:01:13] WARNING: src/c_api/c_api.cc:935: `ntree_limit` is deprecated, use `iteration_range` instead.
## [16:01:13] WARNING: src/c_api/c_api.cc:935: `ntree_limit` is deprecated, use `iteration_range` instead.
## [16:01:14] WARNING: src/c_api/c_api.cc:935: `ntree_limit` is deprecated, use `iteration_range` instead.
## [16:01:14] WARNING: src/c_api/c_api.cc:935: `ntree_limit` is deprecated, use `iteration_range` instead.
## [16:01:14] WARNING: src/c_api/c_api.cc:935: `ntree_limit` is deprecated, use `iteration_range` instead.
## [16:01:14] WARNING: src/c_api/c_api.cc:935: `ntree_limit` is deprecated, use `iteration_range` instead.
## [16:01:14] WARNING: src/c_api/c_api.cc:935: `ntree_limit` is deprecated, use `iteration_range` instead.
## [16:01:14] WARNING: src/c_api/c_api.cc:935: `ntree_limit` is deprecated, use `iteration_range` instead.
## [16:01:14] WARNING: src/c_api/c_api.cc:935: `ntree_limit` is deprecated, use `iteration_range` instead.
## [16:01:14] WARNING: src/c_api/c_api.cc:935: `ntree_limit` is deprecated, use `iteration_range` instead.
## [16:01:14] WARNING: src/c_api/c_api.cc:935: `ntree_limit` is deprecated, use `iteration_range` instead.
## [16:01:14] WARNING: src/c_api/c_api.cc:935: `ntree_limit` is deprecated, use `iteration_range` instead.
## [16:01:14] WARNING: src/c_api/c_api.cc:935: `ntree_limit` is deprecated, use `iteration_range` instead.
## [16:01:14] WARNING: src/c_api/c_api.cc:935: `ntree_limit` is deprecated, use `iteration_range` instead.
## [16:01:15] WARNING: src/c_api/c_api.cc:935: `ntree_limit` is deprecated, use `iteration_range` instead.
## [16:01:15] WARNING: src/c_api/c_api.cc:935: `ntree_limit` is deprecated, use `iteration_range` instead.
## [16:01:15] WARNING: src/c_api/c_api.cc:935: `ntree_limit` is deprecated, use `iteration_range` instead.
## [16:01:15] WARNING: src/c_api/c_api.cc:935: `ntree_limit` is deprecated, use `iteration_range` instead.
## [16:01:15] WARNING: src/c_api/c_api.cc:935: `ntree_limit` is deprecated, use `iteration_range` instead.
## [16:01:15] WARNING: src/c_api/c_api.cc:935: `ntree_limit` is deprecated, use `iteration_range` instead.
## [16:01:15] WARNING: src/c_api/c_api.cc:935: `ntree_limit` is deprecated, use `iteration_range` instead.
## [16:01:15] WARNING: src/c_api/c_api.cc:935: `ntree_limit` is deprecated, use `iteration_range` instead.
## [16:01:15] WARNING: src/c_api/c_api.cc:935: `ntree_limit` is deprecated, use `iteration_range` instead.
## [16:01:15] WARNING: src/c_api/c_api.cc:935: `ntree_limit` is deprecated, use `iteration_range` instead.
## [16:01:16] WARNING: src/c_api/c_api.cc:935: `ntree_limit` is deprecated, use `iteration_range` instead.
## [16:01:16] WARNING: src/c_api/c_api.cc:935: `ntree_limit` is deprecated, use `iteration_range` instead.
## [16:01:16] WARNING: src/c_api/c_api.cc:935: `ntree_limit` is deprecated, use `iteration_range` instead.
## [16:01:16] WARNING: src/c_api/c_api.cc:935: `ntree_limit` is deprecated, use `iteration_range` instead.
## [16:01:16] WARNING: src/c_api/c_api.cc:935: `ntree_limit` is deprecated, use `iteration_range` instead.
## [16:01:16] WARNING: src/c_api/c_api.cc:935: `ntree_limit` is deprecated, use `iteration_range` instead.
## [16:01:16] WARNING: src/c_api/c_api.cc:935: `ntree_limit` is deprecated, use `iteration_range` instead.
## [16:01:16] WARNING: src/c_api/c_api.cc:935: `ntree_limit` is deprecated, use `iteration_range` instead.
## [16:01:16] WARNING: src/c_api/c_api.cc:935: `ntree_limit` is deprecated, use `iteration_range` instead.
## [16:01:16] WARNING: src/c_api/c_api.cc:935: `ntree_limit` is deprecated, use `iteration_range` instead.
## [16:01:16] WARNING: src/c_api/c_api.cc:935: `ntree_limit` is deprecated, use `iteration_range` instead.
## [16:01:16] WARNING: src/c_api/c_api.cc:935: `ntree_limit` is deprecated, use `iteration_range` instead.
## [16:01:16] WARNING: src/c_api/c_api.cc:935: `ntree_limit` is deprecated, use `iteration_range` instead.
## [16:01:16] WARNING: src/c_api/c_api.cc:935: `ntree_limit` is deprecated, use `iteration_range` instead.
## [16:01:17] WARNING: src/c_api/c_api.cc:935: `ntree_limit` is deprecated, use `iteration_range` instead.
## [16:01:17] WARNING: src/c_api/c_api.cc:935: `ntree_limit` is deprecated, use `iteration_range` instead.
## [16:01:17] WARNING: src/c_api/c_api.cc:935: `ntree_limit` is deprecated, use `iteration_range` instead.
## [16:01:17] WARNING: src/c_api/c_api.cc:935: `ntree_limit` is deprecated, use `iteration_range` instead.
## [16:01:17] WARNING: src/c_api/c_api.cc:935: `ntree_limit` is deprecated, use `iteration_range` instead.
## [16:01:17] WARNING: src/c_api/c_api.cc:935: `ntree_limit` is deprecated, use `iteration_range` instead.
## [16:01:17] WARNING: src/c_api/c_api.cc:935: `ntree_limit` is deprecated, use `iteration_range` instead.
## [16:01:17] WARNING: src/c_api/c_api.cc:935: `ntree_limit` is deprecated, use `iteration_range` instead.
## [16:01:17] WARNING: src/c_api/c_api.cc:935: `ntree_limit` is deprecated, use `iteration_range` instead.
## [16:01:17] WARNING: src/c_api/c_api.cc:935: `ntree_limit` is deprecated, use `iteration_range` instead.
## [16:01:18] WARNING: src/c_api/c_api.cc:935: `ntree_limit` is deprecated, use `iteration_range` instead.
## [16:01:18] WARNING: src/c_api/c_api.cc:935: `ntree_limit` is deprecated, use `iteration_range` instead.
## [16:01:18] WARNING: src/c_api/c_api.cc:935: `ntree_limit` is deprecated, use `iteration_range` instead.
## [16:01:18] WARNING: src/c_api/c_api.cc:935: `ntree_limit` is deprecated, use `iteration_range` instead.
## [16:01:18] WARNING: src/c_api/c_api.cc:935: `ntree_limit` is deprecated, use `iteration_range` instead.
## [16:01:18] WARNING: src/c_api/c_api.cc:935: `ntree_limit` is deprecated, use `iteration_range` instead.
## [16:01:18] WARNING: src/c_api/c_api.cc:935: `ntree_limit` is deprecated, use `iteration_range` instead.
## [16:01:18] WARNING: src/c_api/c_api.cc:935: `ntree_limit` is deprecated, use `iteration_range` instead.
## [16:01:18] WARNING: src/c_api/c_api.cc:935: `ntree_limit` is deprecated, use `iteration_range` instead.
## [16:01:18] WARNING: src/c_api/c_api.cc:935: `ntree_limit` is deprecated, use `iteration_range` instead.
## [16:01:18] WARNING: src/c_api/c_api.cc:935: `ntree_limit` is deprecated, use `iteration_range` instead.
## [16:01:18] WARNING: src/c_api/c_api.cc:935: `ntree_limit` is deprecated, use `iteration_range` instead.
## [16:01:19] WARNING: src/c_api/c_api.cc:935: `ntree_limit` is deprecated, use `iteration_range` instead.
## [16:01:19] WARNING: src/c_api/c_api.cc:935: `ntree_limit` is deprecated, use `iteration_range` instead.
## [16:01:19] WARNING: src/c_api/c_api.cc:935: `ntree_limit` is deprecated, use `iteration_range` instead.
## [16:01:19] WARNING: src/c_api/c_api.cc:935: `ntree_limit` is deprecated, use `iteration_range` instead.
## [16:01:19] WARNING: src/c_api/c_api.cc:935: `ntree_limit` is deprecated, use `iteration_range` instead.
## [16:01:19] WARNING: src/c_api/c_api.cc:935: `ntree_limit` is deprecated, use `iteration_range` instead.
## [16:01:19] WARNING: src/c_api/c_api.cc:935: `ntree_limit` is deprecated, use `iteration_range` instead.
## [16:01:19] WARNING: src/c_api/c_api.cc:935: `ntree_limit` is deprecated, use `iteration_range` instead.
## [16:01:19] WARNING: src/c_api/c_api.cc:935: `ntree_limit` is deprecated, use `iteration_range` instead.
## [16:01:19] WARNING: src/c_api/c_api.cc:935: `ntree_limit` is deprecated, use `iteration_range` instead.
## [16:01:19] WARNING: src/c_api/c_api.cc:935: `ntree_limit` is deprecated, use `iteration_range` instead.
## [16:01:19] WARNING: src/c_api/c_api.cc:935: `ntree_limit` is deprecated, use `iteration_range` instead.
## [16:01:19] WARNING: src/c_api/c_api.cc:935: `ntree_limit` is deprecated, use `iteration_range` instead.
## [16:01:19] WARNING: src/c_api/c_api.cc:935: `ntree_limit` is deprecated, use `iteration_range` instead.
## [16:01:20] WARNING: src/c_api/c_api.cc:935: `ntree_limit` is deprecated, use `iteration_range` instead.
## [16:01:20] WARNING: src/c_api/c_api.cc:935: `ntree_limit` is deprecated, use `iteration_range` instead.
## [16:01:20] WARNING: src/c_api/c_api.cc:935: `ntree_limit` is deprecated, use `iteration_range` instead.
## [16:01:20] WARNING: src/c_api/c_api.cc:935: `ntree_limit` is deprecated, use `iteration_range` instead.
## [16:01:20] WARNING: src/c_api/c_api.cc:935: `ntree_limit` is deprecated, use `iteration_range` instead.
## [16:01:20] WARNING: src/c_api/c_api.cc:935: `ntree_limit` is deprecated, use `iteration_range` instead.
## [16:01:20] WARNING: src/c_api/c_api.cc:935: `ntree_limit` is deprecated, use `iteration_range` instead.
## [16:01:20] WARNING: src/c_api/c_api.cc:935: `ntree_limit` is deprecated, use `iteration_range` instead.
## [16:01:20] WARNING: src/c_api/c_api.cc:935: `ntree_limit` is deprecated, use `iteration_range` instead.
## [16:01:20] WARNING: src/c_api/c_api.cc:935: `ntree_limit` is deprecated, use `iteration_range` instead.
## [16:01:21] WARNING: src/c_api/c_api.cc:935: `ntree_limit` is deprecated, use `iteration_range` instead.
## [16:01:21] WARNING: src/c_api/c_api.cc:935: `ntree_limit` is deprecated, use `iteration_range` instead.
## [16:01:21] WARNING: src/c_api/c_api.cc:935: `ntree_limit` is deprecated, use `iteration_range` instead.
## [16:01:21] WARNING: src/c_api/c_api.cc:935: `ntree_limit` is deprecated, use `iteration_range` instead.
## [16:01:21] WARNING: src/c_api/c_api.cc:935: `ntree_limit` is deprecated, use `iteration_range` instead.
## [16:01:21] WARNING: src/c_api/c_api.cc:935: `ntree_limit` is deprecated, use `iteration_range` instead.
## [16:01:21] WARNING: src/c_api/c_api.cc:935: `ntree_limit` is deprecated, use `iteration_range` instead.
## [16:01:21] WARNING: src/c_api/c_api.cc:935: `ntree_limit` is deprecated, use `iteration_range` instead.
## [16:01:21] WARNING: src/c_api/c_api.cc:935: `ntree_limit` is deprecated, use `iteration_range` instead.
## [16:01:21] WARNING: src/c_api/c_api.cc:935: `ntree_limit` is deprecated, use `iteration_range` instead.
## [16:01:22] WARNING: src/c_api/c_api.cc:935: `ntree_limit` is deprecated, use `iteration_range` instead.
## [16:01:22] WARNING: src/c_api/c_api.cc:935: `ntree_limit` is deprecated, use `iteration_range` instead.
## [16:01:22] WARNING: src/c_api/c_api.cc:935: `ntree_limit` is deprecated, use `iteration_range` instead.
## [16:01:22] WARNING: src/c_api/c_api.cc:935: `ntree_limit` is deprecated, use `iteration_range` instead.
## [16:01:22] WARNING: src/c_api/c_api.cc:935: `ntree_limit` is deprecated, use `iteration_range` instead.
## [16:01:22] WARNING: src/c_api/c_api.cc:935: `ntree_limit` is deprecated, use `iteration_range` instead.
## [16:01:22] WARNING: src/c_api/c_api.cc:935: `ntree_limit` is deprecated, use `iteration_range` instead.
## [16:01:22] WARNING: src/c_api/c_api.cc:935: `ntree_limit` is deprecated, use `iteration_range` instead.
## [16:01:22] WARNING: src/c_api/c_api.cc:935: `ntree_limit` is deprecated, use `iteration_range` instead.
## [16:01:22] WARNING: src/c_api/c_api.cc:935: `ntree_limit` is deprecated, use `iteration_range` instead.
## [16:01:22] WARNING: src/c_api/c_api.cc:935: `ntree_limit` is deprecated, use `iteration_range` instead.
## [16:01:22] WARNING: src/c_api/c_api.cc:935: `ntree_limit` is deprecated, use `iteration_range` instead.
## [16:01:22] WARNING: src/c_api/c_api.cc:935: `ntree_limit` is deprecated, use `iteration_range` instead.
## [16:01:22] WARNING: src/c_api/c_api.cc:935: `ntree_limit` is deprecated, use `iteration_range` instead.
## [16:01:22] WARNING: src/c_api/c_api.cc:935: `ntree_limit` is deprecated, use `iteration_range` instead.
## [16:01:22] WARNING: src/c_api/c_api.cc:935: `ntree_limit` is deprecated, use `iteration_range` instead.
## [16:01:23] WARNING: src/c_api/c_api.cc:935: `ntree_limit` is deprecated, use `iteration_range` instead.
## [16:01:23] WARNING: src/c_api/c_api.cc:935: `ntree_limit` is deprecated, use `iteration_range` instead.
## [16:01:23] WARNING: src/c_api/c_api.cc:935: `ntree_limit` is deprecated, use `iteration_range` instead.
## [16:01:23] WARNING: src/c_api/c_api.cc:935: `ntree_limit` is deprecated, use `iteration_range` instead.
## [16:01:23] WARNING: src/c_api/c_api.cc:935: `ntree_limit` is deprecated, use `iteration_range` instead.
## [16:01:23] WARNING: src/c_api/c_api.cc:935: `ntree_limit` is deprecated, use `iteration_range` instead.
## [16:01:23] WARNING: src/c_api/c_api.cc:935: `ntree_limit` is deprecated, use `iteration_range` instead.
## [16:01:23] WARNING: src/c_api/c_api.cc:935: `ntree_limit` is deprecated, use `iteration_range` instead.
## [16:01:23] WARNING: src/c_api/c_api.cc:935: `ntree_limit` is deprecated, use `iteration_range` instead.
## [16:01:23] WARNING: src/c_api/c_api.cc:935: `ntree_limit` is deprecated, use `iteration_range` instead.
## [16:01:24] WARNING: src/c_api/c_api.cc:935: `ntree_limit` is deprecated, use `iteration_range` instead.
## [16:01:24] WARNING: src/c_api/c_api.cc:935: `ntree_limit` is deprecated, use `iteration_range` instead.
## [16:01:24] WARNING: src/c_api/c_api.cc:935: `ntree_limit` is deprecated, use `iteration_range` instead.
## [16:01:24] WARNING: src/c_api/c_api.cc:935: `ntree_limit` is deprecated, use `iteration_range` instead.
## [16:01:24] WARNING: src/c_api/c_api.cc:935: `ntree_limit` is deprecated, use `iteration_range` instead.
## [16:01:24] WARNING: src/c_api/c_api.cc:935: `ntree_limit` is deprecated, use `iteration_range` instead.
## [16:01:24] WARNING: src/c_api/c_api.cc:935: `ntree_limit` is deprecated, use `iteration_range` instead.
## [16:01:24] WARNING: src/c_api/c_api.cc:935: `ntree_limit` is deprecated, use `iteration_range` instead.
## [16:01:24] WARNING: src/c_api/c_api.cc:935: `ntree_limit` is deprecated, use `iteration_range` instead.
## [16:01:24] WARNING: src/c_api/c_api.cc:935: `ntree_limit` is deprecated, use `iteration_range` instead.
## [16:01:27] WARNING: src/c_api/c_api.cc:935: `ntree_limit` is deprecated, use `iteration_range` instead.
## [16:01:27] WARNING: src/c_api/c_api.cc:935: `ntree_limit` is deprecated, use `iteration_range` instead.
## [16:01:27] WARNING: src/c_api/c_api.cc:935: `ntree_limit` is deprecated, use `iteration_range` instead.
## [16:01:27] WARNING: src/c_api/c_api.cc:935: `ntree_limit` is deprecated, use `iteration_range` instead.
## [16:01:27] WARNING: src/c_api/c_api.cc:935: `ntree_limit` is deprecated, use `iteration_range` instead.
## [16:01:27] WARNING: src/c_api/c_api.cc:935: `ntree_limit` is deprecated, use `iteration_range` instead.
## [16:01:28] WARNING: src/c_api/c_api.cc:935: `ntree_limit` is deprecated, use `iteration_range` instead.
## [16:01:28] WARNING: src/c_api/c_api.cc:935: `ntree_limit` is deprecated, use `iteration_range` instead.
## [16:01:28] WARNING: src/c_api/c_api.cc:935: `ntree_limit` is deprecated, use `iteration_range` instead.
## [16:01:28] WARNING: src/c_api/c_api.cc:935: `ntree_limit` is deprecated, use `iteration_range` instead.
## [16:01:28] WARNING: src/c_api/c_api.cc:935: `ntree_limit` is deprecated, use `iteration_range` instead.
## [16:01:28] WARNING: src/c_api/c_api.cc:935: `ntree_limit` is deprecated, use `iteration_range` instead.
## [16:01:28] WARNING: src/c_api/c_api.cc:935: `ntree_limit` is deprecated, use `iteration_range` instead.
## [16:01:28] WARNING: src/c_api/c_api.cc:935: `ntree_limit` is deprecated, use `iteration_range` instead.
## [16:01:28] WARNING: src/c_api/c_api.cc:935: `ntree_limit` is deprecated, use `iteration_range` instead.
## [16:01:28] WARNING: src/c_api/c_api.cc:935: `ntree_limit` is deprecated, use `iteration_range` instead.
## [16:01:28] WARNING: src/c_api/c_api.cc:935: `ntree_limit` is deprecated, use `iteration_range` instead.
## [16:01:28] WARNING: src/c_api/c_api.cc:935: `ntree_limit` is deprecated, use `iteration_range` instead.
## [16:01:29] WARNING: src/c_api/c_api.cc:935: `ntree_limit` is deprecated, use `iteration_range` instead.
## [16:01:29] WARNING: src/c_api/c_api.cc:935: `ntree_limit` is deprecated, use `iteration_range` instead.
## [16:01:29] WARNING: src/c_api/c_api.cc:935: `ntree_limit` is deprecated, use `iteration_range` instead.
## [16:01:29] WARNING: src/c_api/c_api.cc:935: `ntree_limit` is deprecated, use `iteration_range` instead.
## [16:01:29] WARNING: src/c_api/c_api.cc:935: `ntree_limit` is deprecated, use `iteration_range` instead.
## [16:01:29] WARNING: src/c_api/c_api.cc:935: `ntree_limit` is deprecated, use `iteration_range` instead.
## [16:01:29] WARNING: src/c_api/c_api.cc:935: `ntree_limit` is deprecated, use `iteration_range` instead.
## [16:01:29] WARNING: src/c_api/c_api.cc:935: `ntree_limit` is deprecated, use `iteration_range` instead.
## [16:01:29] WARNING: src/c_api/c_api.cc:935: `ntree_limit` is deprecated, use `iteration_range` instead.
## [16:01:29] WARNING: src/c_api/c_api.cc:935: `ntree_limit` is deprecated, use `iteration_range` instead.
## [16:01:30] WARNING: src/c_api/c_api.cc:935: `ntree_limit` is deprecated, use `iteration_range` instead.
## [16:01:30] WARNING: src/c_api/c_api.cc:935: `ntree_limit` is deprecated, use `iteration_range` instead.
## [16:01:30] WARNING: src/c_api/c_api.cc:935: `ntree_limit` is deprecated, use `iteration_range` instead.
## [16:01:30] WARNING: src/c_api/c_api.cc:935: `ntree_limit` is deprecated, use `iteration_range` instead.
## [16:01:30] WARNING: src/c_api/c_api.cc:935: `ntree_limit` is deprecated, use `iteration_range` instead.
## [16:01:30] WARNING: src/c_api/c_api.cc:935: `ntree_limit` is deprecated, use `iteration_range` instead.
## [16:01:30] WARNING: src/c_api/c_api.cc:935: `ntree_limit` is deprecated, use `iteration_range` instead.
## [16:01:30] WARNING: src/c_api/c_api.cc:935: `ntree_limit` is deprecated, use `iteration_range` instead.
## [16:01:30] WARNING: src/c_api/c_api.cc:935: `ntree_limit` is deprecated, use `iteration_range` instead.
## [16:01:30] WARNING: src/c_api/c_api.cc:935: `ntree_limit` is deprecated, use `iteration_range` instead.
## [16:01:30] WARNING: src/c_api/c_api.cc:935: `ntree_limit` is deprecated, use `iteration_range` instead.
## [16:01:30] WARNING: src/c_api/c_api.cc:935: `ntree_limit` is deprecated, use `iteration_range` instead.
## [16:01:31] WARNING: src/c_api/c_api.cc:935: `ntree_limit` is deprecated, use `iteration_range` instead.
## [16:01:31] WARNING: src/c_api/c_api.cc:935: `ntree_limit` is deprecated, use `iteration_range` instead.
## [16:01:31] WARNING: src/c_api/c_api.cc:935: `ntree_limit` is deprecated, use `iteration_range` instead.
## [16:01:31] WARNING: src/c_api/c_api.cc:935: `ntree_limit` is deprecated, use `iteration_range` instead.
## [16:01:31] WARNING: src/c_api/c_api.cc:935: `ntree_limit` is deprecated, use `iteration_range` instead.
## [16:01:31] WARNING: src/c_api/c_api.cc:935: `ntree_limit` is deprecated, use `iteration_range` instead.
## [16:01:31] WARNING: src/c_api/c_api.cc:935: `ntree_limit` is deprecated, use `iteration_range` instead.
## [16:01:31] WARNING: src/c_api/c_api.cc:935: `ntree_limit` is deprecated, use `iteration_range` instead.
## [16:01:31] WARNING: src/c_api/c_api.cc:935: `ntree_limit` is deprecated, use `iteration_range` instead.
## [16:01:31] WARNING: src/c_api/c_api.cc:935: `ntree_limit` is deprecated, use `iteration_range` instead.
## [16:01:31] WARNING: src/c_api/c_api.cc:935: `ntree_limit` is deprecated, use `iteration_range` instead.
## [16:01:31] WARNING: src/c_api/c_api.cc:935: `ntree_limit` is deprecated, use `iteration_range` instead.
## [16:01:32] WARNING: src/c_api/c_api.cc:935: `ntree_limit` is deprecated, use `iteration_range` instead.
## [16:01:32] WARNING: src/c_api/c_api.cc:935: `ntree_limit` is deprecated, use `iteration_range` instead.
## [16:01:32] WARNING: src/c_api/c_api.cc:935: `ntree_limit` is deprecated, use `iteration_range` instead.
## [16:01:32] WARNING: src/c_api/c_api.cc:935: `ntree_limit` is deprecated, use `iteration_range` instead.
## [16:01:32] WARNING: src/c_api/c_api.cc:935: `ntree_limit` is deprecated, use `iteration_range` instead.
## [16:01:32] WARNING: src/c_api/c_api.cc:935: `ntree_limit` is deprecated, use `iteration_range` instead.
## [16:01:32] WARNING: src/c_api/c_api.cc:935: `ntree_limit` is deprecated, use `iteration_range` instead.
## [16:01:32] WARNING: src/c_api/c_api.cc:935: `ntree_limit` is deprecated, use `iteration_range` instead.
## [16:01:32] WARNING: src/c_api/c_api.cc:935: `ntree_limit` is deprecated, use `iteration_range` instead.
## [16:01:32] WARNING: src/c_api/c_api.cc:935: `ntree_limit` is deprecated, use `iteration_range` instead.
## [16:01:33] WARNING: src/c_api/c_api.cc:935: `ntree_limit` is deprecated, use `iteration_range` instead.
## [16:01:33] WARNING: src/c_api/c_api.cc:935: `ntree_limit` is deprecated, use `iteration_range` instead.
## [16:01:33] WARNING: src/c_api/c_api.cc:935: `ntree_limit` is deprecated, use `iteration_range` instead.
## [16:01:33] WARNING: src/c_api/c_api.cc:935: `ntree_limit` is deprecated, use `iteration_range` instead.
## [16:01:33] WARNING: src/c_api/c_api.cc:935: `ntree_limit` is deprecated, use `iteration_range` instead.
## [16:01:33] WARNING: src/c_api/c_api.cc:935: `ntree_limit` is deprecated, use `iteration_range` instead.
## [16:01:33] WARNING: src/c_api/c_api.cc:935: `ntree_limit` is deprecated, use `iteration_range` instead.
## [16:01:33] WARNING: src/c_api/c_api.cc:935: `ntree_limit` is deprecated, use `iteration_range` instead.
## [16:01:33] WARNING: src/c_api/c_api.cc:935: `ntree_limit` is deprecated, use `iteration_range` instead.
## [16:01:33] WARNING: src/c_api/c_api.cc:935: `ntree_limit` is deprecated, use `iteration_range` instead.
## [16:01:34] WARNING: src/c_api/c_api.cc:935: `ntree_limit` is deprecated, use `iteration_range` instead.
## [16:01:34] WARNING: src/c_api/c_api.cc:935: `ntree_limit` is deprecated, use `iteration_range` instead.
## [16:01:34] WARNING: src/c_api/c_api.cc:935: `ntree_limit` is deprecated, use `iteration_range` instead.
## [16:01:34] WARNING: src/c_api/c_api.cc:935: `ntree_limit` is deprecated, use `iteration_range` instead.
## [16:01:34] WARNING: src/c_api/c_api.cc:935: `ntree_limit` is deprecated, use `iteration_range` instead.
## [16:01:34] WARNING: src/c_api/c_api.cc:935: `ntree_limit` is deprecated, use `iteration_range` instead.
## [16:01:34] WARNING: src/c_api/c_api.cc:935: `ntree_limit` is deprecated, use `iteration_range` instead.
## [16:01:34] WARNING: src/c_api/c_api.cc:935: `ntree_limit` is deprecated, use `iteration_range` instead.
## [16:01:34] WARNING: src/c_api/c_api.cc:935: `ntree_limit` is deprecated, use `iteration_range` instead.
## [16:01:34] WARNING: src/c_api/c_api.cc:935: `ntree_limit` is deprecated, use `iteration_range` instead.
## [16:01:34] WARNING: src/c_api/c_api.cc:935: `ntree_limit` is deprecated, use `iteration_range` instead.
## [16:01:34] WARNING: src/c_api/c_api.cc:935: `ntree_limit` is deprecated, use `iteration_range` instead.
## [16:01:34] WARNING: src/c_api/c_api.cc:935: `ntree_limit` is deprecated, use `iteration_range` instead.
## [16:01:34] WARNING: src/c_api/c_api.cc:935: `ntree_limit` is deprecated, use `iteration_range` instead.
## [16:01:35] WARNING: src/c_api/c_api.cc:935: `ntree_limit` is deprecated, use `iteration_range` instead.
## [16:01:35] WARNING: src/c_api/c_api.cc:935: `ntree_limit` is deprecated, use `iteration_range` instead.
## [16:01:35] WARNING: src/c_api/c_api.cc:935: `ntree_limit` is deprecated, use `iteration_range` instead.
## [16:01:35] WARNING: src/c_api/c_api.cc:935: `ntree_limit` is deprecated, use `iteration_range` instead.
## [16:01:35] WARNING: src/c_api/c_api.cc:935: `ntree_limit` is deprecated, use `iteration_range` instead.
## [16:01:35] WARNING: src/c_api/c_api.cc:935: `ntree_limit` is deprecated, use `iteration_range` instead.
## [16:01:35] WARNING: src/c_api/c_api.cc:935: `ntree_limit` is deprecated, use `iteration_range` instead.
## [16:01:35] WARNING: src/c_api/c_api.cc:935: `ntree_limit` is deprecated, use `iteration_range` instead.
## [16:01:35] WARNING: src/c_api/c_api.cc:935: `ntree_limit` is deprecated, use `iteration_range` instead.
## [16:01:35] WARNING: src/c_api/c_api.cc:935: `ntree_limit` is deprecated, use `iteration_range` instead.
## [16:01:36] WARNING: src/c_api/c_api.cc:935: `ntree_limit` is deprecated, use `iteration_range` instead.
## [16:01:36] WARNING: src/c_api/c_api.cc:935: `ntree_limit` is deprecated, use `iteration_range` instead.
## [16:01:36] WARNING: src/c_api/c_api.cc:935: `ntree_limit` is deprecated, use `iteration_range` instead.
## [16:01:36] WARNING: src/c_api/c_api.cc:935: `ntree_limit` is deprecated, use `iteration_range` instead.
## [16:01:36] WARNING: src/c_api/c_api.cc:935: `ntree_limit` is deprecated, use `iteration_range` instead.
## [16:01:36] WARNING: src/c_api/c_api.cc:935: `ntree_limit` is deprecated, use `iteration_range` instead.
## [16:01:36] WARNING: src/c_api/c_api.cc:935: `ntree_limit` is deprecated, use `iteration_range` instead.
## [16:01:36] WARNING: src/c_api/c_api.cc:935: `ntree_limit` is deprecated, use `iteration_range` instead.
## [16:01:36] WARNING: src/c_api/c_api.cc:935: `ntree_limit` is deprecated, use `iteration_range` instead.
## [16:01:36] WARNING: src/c_api/c_api.cc:935: `ntree_limit` is deprecated, use `iteration_range` instead.
## [16:01:37] WARNING: src/c_api/c_api.cc:935: `ntree_limit` is deprecated, use `iteration_range` instead.
## [16:01:37] WARNING: src/c_api/c_api.cc:935: `ntree_limit` is deprecated, use `iteration_range` instead.
## [16:01:37] WARNING: src/c_api/c_api.cc:935: `ntree_limit` is deprecated, use `iteration_range` instead.
## [16:01:37] WARNING: src/c_api/c_api.cc:935: `ntree_limit` is deprecated, use `iteration_range` instead.
## [16:01:37] WARNING: src/c_api/c_api.cc:935: `ntree_limit` is deprecated, use `iteration_range` instead.
## [16:01:37] WARNING: src/c_api/c_api.cc:935: `ntree_limit` is deprecated, use `iteration_range` instead.
## [16:01:37] WARNING: src/c_api/c_api.cc:935: `ntree_limit` is deprecated, use `iteration_range` instead.
## [16:01:37] WARNING: src/c_api/c_api.cc:935: `ntree_limit` is deprecated, use `iteration_range` instead.
## [16:01:37] WARNING: src/c_api/c_api.cc:935: `ntree_limit` is deprecated, use `iteration_range` instead.
## [16:01:37] WARNING: src/c_api/c_api.cc:935: `ntree_limit` is deprecated, use `iteration_range` instead.
## [16:01:37] WARNING: src/c_api/c_api.cc:935: `ntree_limit` is deprecated, use `iteration_range` instead.
## [16:01:37] WARNING: src/c_api/c_api.cc:935: `ntree_limit` is deprecated, use `iteration_range` instead.
## [16:01:37] WARNING: src/c_api/c_api.cc:935: `ntree_limit` is deprecated, use `iteration_range` instead.
## [16:01:37] WARNING: src/c_api/c_api.cc:935: `ntree_limit` is deprecated, use `iteration_range` instead.
## [16:01:38] WARNING: src/c_api/c_api.cc:935: `ntree_limit` is deprecated, use `iteration_range` instead.
## [16:01:38] WARNING: src/c_api/c_api.cc:935: `ntree_limit` is deprecated, use `iteration_range` instead.
## [16:01:38] WARNING: src/c_api/c_api.cc:935: `ntree_limit` is deprecated, use `iteration_range` instead.
## [16:01:38] WARNING: src/c_api/c_api.cc:935: `ntree_limit` is deprecated, use `iteration_range` instead.
## [16:01:38] WARNING: src/c_api/c_api.cc:935: `ntree_limit` is deprecated, use `iteration_range` instead.
## [16:01:38] WARNING: src/c_api/c_api.cc:935: `ntree_limit` is deprecated, use `iteration_range` instead.
## [16:01:38] WARNING: src/c_api/c_api.cc:935: `ntree_limit` is deprecated, use `iteration_range` instead.
## [16:01:38] WARNING: src/c_api/c_api.cc:935: `ntree_limit` is deprecated, use `iteration_range` instead.
## [16:01:38] WARNING: src/c_api/c_api.cc:935: `ntree_limit` is deprecated, use `iteration_range` instead.
## [16:01:38] WARNING: src/c_api/c_api.cc:935: `ntree_limit` is deprecated, use `iteration_range` instead.
## [16:01:38] WARNING: src/c_api/c_api.cc:935: `ntree_limit` is deprecated, use `iteration_range` instead.
## [16:01:38] WARNING: src/c_api/c_api.cc:935: `ntree_limit` is deprecated, use `iteration_range` instead.
## [16:01:39] WARNING: src/c_api/c_api.cc:935: `ntree_limit` is deprecated, use `iteration_range` instead.
## [16:01:39] WARNING: src/c_api/c_api.cc:935: `ntree_limit` is deprecated, use `iteration_range` instead.
## [16:01:39] WARNING: src/c_api/c_api.cc:935: `ntree_limit` is deprecated, use `iteration_range` instead.
## [16:01:39] WARNING: src/c_api/c_api.cc:935: `ntree_limit` is deprecated, use `iteration_range` instead.
## [16:01:39] WARNING: src/c_api/c_api.cc:935: `ntree_limit` is deprecated, use `iteration_range` instead.
## [16:01:39] WARNING: src/c_api/c_api.cc:935: `ntree_limit` is deprecated, use `iteration_range` instead.
## [16:01:39] WARNING: src/c_api/c_api.cc:935: `ntree_limit` is deprecated, use `iteration_range` instead.
## [16:01:39] WARNING: src/c_api/c_api.cc:935: `ntree_limit` is deprecated, use `iteration_range` instead.
## [16:01:40] WARNING: src/c_api/c_api.cc:935: `ntree_limit` is deprecated, use `iteration_range` instead.
## [16:01:40] WARNING: src/c_api/c_api.cc:935: `ntree_limit` is deprecated, use `iteration_range` instead.
## [16:01:40] WARNING: src/c_api/c_api.cc:935: `ntree_limit` is deprecated, use `iteration_range` instead.
## [16:01:40] WARNING: src/c_api/c_api.cc:935: `ntree_limit` is deprecated, use `iteration_range` instead.
## [16:01:40] WARNING: src/c_api/c_api.cc:935: `ntree_limit` is deprecated, use `iteration_range` instead.
## [16:01:40] WARNING: src/c_api/c_api.cc:935: `ntree_limit` is deprecated, use `iteration_range` instead.
## [16:01:40] WARNING: src/c_api/c_api.cc:935: `ntree_limit` is deprecated, use `iteration_range` instead.
## [16:01:40] WARNING: src/c_api/c_api.cc:935: `ntree_limit` is deprecated, use `iteration_range` instead.
## [16:01:40] WARNING: src/c_api/c_api.cc:935: `ntree_limit` is deprecated, use `iteration_range` instead.
## [16:01:40] WARNING: src/c_api/c_api.cc:935: `ntree_limit` is deprecated, use `iteration_range` instead.
## [16:01:40] WARNING: src/c_api/c_api.cc:935: `ntree_limit` is deprecated, use `iteration_range` instead.
## [16:01:40] WARNING: src/c_api/c_api.cc:935: `ntree_limit` is deprecated, use `iteration_range` instead.
## [16:01:40] WARNING: src/c_api/c_api.cc:935: `ntree_limit` is deprecated, use `iteration_range` instead.
## [16:01:40] WARNING: src/c_api/c_api.cc:935: `ntree_limit` is deprecated, use `iteration_range` instead.
## [16:01:40] WARNING: src/c_api/c_api.cc:935: `ntree_limit` is deprecated, use `iteration_range` instead.
## [16:01:40] WARNING: src/c_api/c_api.cc:935: `ntree_limit` is deprecated, use `iteration_range` instead.
## [16:01:41] WARNING: src/c_api/c_api.cc:935: `ntree_limit` is deprecated, use `iteration_range` instead.
## [16:01:41] WARNING: src/c_api/c_api.cc:935: `ntree_limit` is deprecated, use `iteration_range` instead.
## [16:01:41] WARNING: src/c_api/c_api.cc:935: `ntree_limit` is deprecated, use `iteration_range` instead.
## [16:01:41] WARNING: src/c_api/c_api.cc:935: `ntree_limit` is deprecated, use `iteration_range` instead.
## [16:01:41] WARNING: src/c_api/c_api.cc:935: `ntree_limit` is deprecated, use `iteration_range` instead.
## [16:01:41] WARNING: src/c_api/c_api.cc:935: `ntree_limit` is deprecated, use `iteration_range` instead.
## [16:01:41] WARNING: src/c_api/c_api.cc:935: `ntree_limit` is deprecated, use `iteration_range` instead.
## [16:01:41] WARNING: src/c_api/c_api.cc:935: `ntree_limit` is deprecated, use `iteration_range` instead.
## [16:01:41] WARNING: src/c_api/c_api.cc:935: `ntree_limit` is deprecated, use `iteration_range` instead.
## [16:01:41] WARNING: src/c_api/c_api.cc:935: `ntree_limit` is deprecated, use `iteration_range` instead.
## [16:01:42] WARNING: src/c_api/c_api.cc:935: `ntree_limit` is deprecated, use `iteration_range` instead.
## [16:01:42] WARNING: src/c_api/c_api.cc:935: `ntree_limit` is deprecated, use `iteration_range` instead.
## [16:01:42] WARNING: src/c_api/c_api.cc:935: `ntree_limit` is deprecated, use `iteration_range` instead.
## [16:01:42] WARNING: src/c_api/c_api.cc:935: `ntree_limit` is deprecated, use `iteration_range` instead.
## [16:01:42] WARNING: src/c_api/c_api.cc:935: `ntree_limit` is deprecated, use `iteration_range` instead.
## [16:01:42] WARNING: src/c_api/c_api.cc:935: `ntree_limit` is deprecated, use `iteration_range` instead.
## [16:01:42] WARNING: src/c_api/c_api.cc:935: `ntree_limit` is deprecated, use `iteration_range` instead.
## [16:01:42] WARNING: src/c_api/c_api.cc:935: `ntree_limit` is deprecated, use `iteration_range` instead.
## [16:01:43] WARNING: src/c_api/c_api.cc:935: `ntree_limit` is deprecated, use `iteration_range` instead.
## [16:01:43] WARNING: src/c_api/c_api.cc:935: `ntree_limit` is deprecated, use `iteration_range` instead.
## [16:01:43] WARNING: src/c_api/c_api.cc:935: `ntree_limit` is deprecated, use `iteration_range` instead.
## [16:01:43] WARNING: src/c_api/c_api.cc:935: `ntree_limit` is deprecated, use `iteration_range` instead.
## [16:01:43] WARNING: src/c_api/c_api.cc:935: `ntree_limit` is deprecated, use `iteration_range` instead.
## [16:01:43] WARNING: src/c_api/c_api.cc:935: `ntree_limit` is deprecated, use `iteration_range` instead.
## [16:01:43] WARNING: src/c_api/c_api.cc:935: `ntree_limit` is deprecated, use `iteration_range` instead.
## [16:01:43] WARNING: src/c_api/c_api.cc:935: `ntree_limit` is deprecated, use `iteration_range` instead.
## [16:01:43] WARNING: src/c_api/c_api.cc:935: `ntree_limit` is deprecated, use `iteration_range` instead.
## [16:01:43] WARNING: src/c_api/c_api.cc:935: `ntree_limit` is deprecated, use `iteration_range` instead.
## [16:01:43] WARNING: src/c_api/c_api.cc:935: `ntree_limit` is deprecated, use `iteration_range` instead.
## [16:01:43] WARNING: src/c_api/c_api.cc:935: `ntree_limit` is deprecated, use `iteration_range` instead.
## [16:01:44] WARNING: src/c_api/c_api.cc:935: `ntree_limit` is deprecated, use `iteration_range` instead.
## [16:01:44] WARNING: src/c_api/c_api.cc:935: `ntree_limit` is deprecated, use `iteration_range` instead.
## [16:01:44] WARNING: src/c_api/c_api.cc:935: `ntree_limit` is deprecated, use `iteration_range` instead.
## [16:01:44] WARNING: src/c_api/c_api.cc:935: `ntree_limit` is deprecated, use `iteration_range` instead.
## [16:01:44] WARNING: src/c_api/c_api.cc:935: `ntree_limit` is deprecated, use `iteration_range` instead.
## [16:01:44] WARNING: src/c_api/c_api.cc:935: `ntree_limit` is deprecated, use `iteration_range` instead.
## [16:01:44] WARNING: src/c_api/c_api.cc:935: `ntree_limit` is deprecated, use `iteration_range` instead.
## [16:01:44] WARNING: src/c_api/c_api.cc:935: `ntree_limit` is deprecated, use `iteration_range` instead.
## [16:01:44] WARNING: src/c_api/c_api.cc:935: `ntree_limit` is deprecated, use `iteration_range` instead.
## [16:01:44] WARNING: src/c_api/c_api.cc:935: `ntree_limit` is deprecated, use `iteration_range` instead.
## [16:01:44] WARNING: src/c_api/c_api.cc:935: `ntree_limit` is deprecated, use `iteration_range` instead.
## [16:01:44] WARNING: src/c_api/c_api.cc:935: `ntree_limit` is deprecated, use `iteration_range` instead.
## [16:01:44] WARNING: src/c_api/c_api.cc:935: `ntree_limit` is deprecated, use `iteration_range` instead.
## [16:01:44] WARNING: src/c_api/c_api.cc:935: `ntree_limit` is deprecated, use `iteration_range` instead.
## [16:01:45] WARNING: src/c_api/c_api.cc:935: `ntree_limit` is deprecated, use `iteration_range` instead.
## [16:01:45] WARNING: src/c_api/c_api.cc:935: `ntree_limit` is deprecated, use `iteration_range` instead.
## [16:01:45] WARNING: src/c_api/c_api.cc:935: `ntree_limit` is deprecated, use `iteration_range` instead.
## [16:01:45] WARNING: src/c_api/c_api.cc:935: `ntree_limit` is deprecated, use `iteration_range` instead.
## [16:01:45] WARNING: src/c_api/c_api.cc:935: `ntree_limit` is deprecated, use `iteration_range` instead.
## [16:01:45] WARNING: src/c_api/c_api.cc:935: `ntree_limit` is deprecated, use `iteration_range` instead.
## [16:01:45] WARNING: src/c_api/c_api.cc:935: `ntree_limit` is deprecated, use `iteration_range` instead.
## [16:01:45] WARNING: src/c_api/c_api.cc:935: `ntree_limit` is deprecated, use `iteration_range` instead.
## [16:01:45] WARNING: src/c_api/c_api.cc:935: `ntree_limit` is deprecated, use `iteration_range` instead.
## [16:01:45] WARNING: src/c_api/c_api.cc:935: `ntree_limit` is deprecated, use `iteration_range` instead.
## [16:01:46] WARNING: src/c_api/c_api.cc:935: `ntree_limit` is deprecated, use `iteration_range` instead.
## [16:01:46] WARNING: src/c_api/c_api.cc:935: `ntree_limit` is deprecated, use `iteration_range` instead.
## [16:01:46] WARNING: src/c_api/c_api.cc:935: `ntree_limit` is deprecated, use `iteration_range` instead.
## [16:01:46] WARNING: src/c_api/c_api.cc:935: `ntree_limit` is deprecated, use `iteration_range` instead.
## [16:01:46] WARNING: src/c_api/c_api.cc:935: `ntree_limit` is deprecated, use `iteration_range` instead.
## [16:01:46] WARNING: src/c_api/c_api.cc:935: `ntree_limit` is deprecated, use `iteration_range` instead.
## [16:01:46] WARNING: src/c_api/c_api.cc:935: `ntree_limit` is deprecated, use `iteration_range` instead.
## [16:01:46] WARNING: src/c_api/c_api.cc:935: `ntree_limit` is deprecated, use `iteration_range` instead.
## [16:01:46] WARNING: src/c_api/c_api.cc:935: `ntree_limit` is deprecated, use `iteration_range` instead.
## [16:01:46] WARNING: src/c_api/c_api.cc:935: `ntree_limit` is deprecated, use `iteration_range` instead.
## [16:01:47] WARNING: src/c_api/c_api.cc:935: `ntree_limit` is deprecated, use `iteration_range` instead.
## [16:01:47] WARNING: src/c_api/c_api.cc:935: `ntree_limit` is deprecated, use `iteration_range` instead.
## [16:01:47] WARNING: src/c_api/c_api.cc:935: `ntree_limit` is deprecated, use `iteration_range` instead.
## [16:01:47] WARNING: src/c_api/c_api.cc:935: `ntree_limit` is deprecated, use `iteration_range` instead.
## [16:01:47] WARNING: src/c_api/c_api.cc:935: `ntree_limit` is deprecated, use `iteration_range` instead.
## [16:01:47] WARNING: src/c_api/c_api.cc:935: `ntree_limit` is deprecated, use `iteration_range` instead.
## [16:01:47] WARNING: src/c_api/c_api.cc:935: `ntree_limit` is deprecated, use `iteration_range` instead.
## [16:01:47] WARNING: src/c_api/c_api.cc:935: `ntree_limit` is deprecated, use `iteration_range` instead.
## [16:01:47] WARNING: src/c_api/c_api.cc:935: `ntree_limit` is deprecated, use `iteration_range` instead.
## [16:01:47] WARNING: src/c_api/c_api.cc:935: `ntree_limit` is deprecated, use `iteration_range` instead.
## [16:01:47] WARNING: src/c_api/c_api.cc:935: `ntree_limit` is deprecated, use `iteration_range` instead.
## [16:01:47] WARNING: src/c_api/c_api.cc:935: `ntree_limit` is deprecated, use `iteration_range` instead.
## [16:01:47] WARNING: src/c_api/c_api.cc:935: `ntree_limit` is deprecated, use `iteration_range` instead.
## [16:01:47] WARNING: src/c_api/c_api.cc:935: `ntree_limit` is deprecated, use `iteration_range` instead.
## [16:01:48] WARNING: src/c_api/c_api.cc:935: `ntree_limit` is deprecated, use `iteration_range` instead.
## [16:01:48] WARNING: src/c_api/c_api.cc:935: `ntree_limit` is deprecated, use `iteration_range` instead.
## [16:01:48] WARNING: src/c_api/c_api.cc:935: `ntree_limit` is deprecated, use `iteration_range` instead.
## [16:01:48] WARNING: src/c_api/c_api.cc:935: `ntree_limit` is deprecated, use `iteration_range` instead.
## [16:01:48] WARNING: src/c_api/c_api.cc:935: `ntree_limit` is deprecated, use `iteration_range` instead.
## [16:01:48] WARNING: src/c_api/c_api.cc:935: `ntree_limit` is deprecated, use `iteration_range` instead.
## [16:01:48] WARNING: src/c_api/c_api.cc:935: `ntree_limit` is deprecated, use `iteration_range` instead.
## [16:01:48] WARNING: src/c_api/c_api.cc:935: `ntree_limit` is deprecated, use `iteration_range` instead.
## [16:01:48] WARNING: src/c_api/c_api.cc:935: `ntree_limit` is deprecated, use `iteration_range` instead.
## [16:01:48] WARNING: src/c_api/c_api.cc:935: `ntree_limit` is deprecated, use `iteration_range` instead.
## [16:01:49] WARNING: src/c_api/c_api.cc:935: `ntree_limit` is deprecated, use `iteration_range` instead.
## [16:01:49] WARNING: src/c_api/c_api.cc:935: `ntree_limit` is deprecated, use `iteration_range` instead.
## [16:01:49] WARNING: src/c_api/c_api.cc:935: `ntree_limit` is deprecated, use `iteration_range` instead.
## [16:01:49] WARNING: src/c_api/c_api.cc:935: `ntree_limit` is deprecated, use `iteration_range` instead.
## [16:01:49] WARNING: src/c_api/c_api.cc:935: `ntree_limit` is deprecated, use `iteration_range` instead.
## [16:01:49] WARNING: src/c_api/c_api.cc:935: `ntree_limit` is deprecated, use `iteration_range` instead.
## [16:01:49] WARNING: src/c_api/c_api.cc:935: `ntree_limit` is deprecated, use `iteration_range` instead.
## [16:01:49] WARNING: src/c_api/c_api.cc:935: `ntree_limit` is deprecated, use `iteration_range` instead.
## [16:01:50] WARNING: src/c_api/c_api.cc:935: `ntree_limit` is deprecated, use `iteration_range` instead.
## [16:01:50] WARNING: src/c_api/c_api.cc:935: `ntree_limit` is deprecated, use `iteration_range` instead.
## [16:01:50] WARNING: src/c_api/c_api.cc:935: `ntree_limit` is deprecated, use `iteration_range` instead.
## [16:01:50] WARNING: src/c_api/c_api.cc:935: `ntree_limit` is deprecated, use `iteration_range` instead.
## [16:01:50] WARNING: src/c_api/c_api.cc:935: `ntree_limit` is deprecated, use `iteration_range` instead.
## [16:01:50] WARNING: src/c_api/c_api.cc:935: `ntree_limit` is deprecated, use `iteration_range` instead.
## [16:01:50] WARNING: src/c_api/c_api.cc:935: `ntree_limit` is deprecated, use `iteration_range` instead.
## [16:01:50] WARNING: src/c_api/c_api.cc:935: `ntree_limit` is deprecated, use `iteration_range` instead.
## [16:01:50] WARNING: src/c_api/c_api.cc:935: `ntree_limit` is deprecated, use `iteration_range` instead.
## [16:01:50] WARNING: src/c_api/c_api.cc:935: `ntree_limit` is deprecated, use `iteration_range` instead.
## [16:01:50] WARNING: src/c_api/c_api.cc:935: `ntree_limit` is deprecated, use `iteration_range` instead.
## [16:01:50] WARNING: src/c_api/c_api.cc:935: `ntree_limit` is deprecated, use `iteration_range` instead.
## [16:01:50] WARNING: src/c_api/c_api.cc:935: `ntree_limit` is deprecated, use `iteration_range` instead.
## [16:01:50] WARNING: src/c_api/c_api.cc:935: `ntree_limit` is deprecated, use `iteration_range` instead.
## [16:01:51] WARNING: src/c_api/c_api.cc:935: `ntree_limit` is deprecated, use `iteration_range` instead.
## [16:01:51] WARNING: src/c_api/c_api.cc:935: `ntree_limit` is deprecated, use `iteration_range` instead.
## [16:01:51] WARNING: src/c_api/c_api.cc:935: `ntree_limit` is deprecated, use `iteration_range` instead.
## [16:01:51] WARNING: src/c_api/c_api.cc:935: `ntree_limit` is deprecated, use `iteration_range` instead.
## [16:01:51] WARNING: src/c_api/c_api.cc:935: `ntree_limit` is deprecated, use `iteration_range` instead.
## [16:01:51] WARNING: src/c_api/c_api.cc:935: `ntree_limit` is deprecated, use `iteration_range` instead.
## [16:01:51] WARNING: src/c_api/c_api.cc:935: `ntree_limit` is deprecated, use `iteration_range` instead.
## [16:01:51] WARNING: src/c_api/c_api.cc:935: `ntree_limit` is deprecated, use `iteration_range` instead.
## [16:01:51] WARNING: src/c_api/c_api.cc:935: `ntree_limit` is deprecated, use `iteration_range` instead.
## [16:01:51] WARNING: src/c_api/c_api.cc:935: `ntree_limit` is deprecated, use `iteration_range` instead.
## [16:01:51] WARNING: src/c_api/c_api.cc:935: `ntree_limit` is deprecated, use `iteration_range` instead.
## [16:01:51] WARNING: src/c_api/c_api.cc:935: `ntree_limit` is deprecated, use `iteration_range` instead.
## [16:01:52] WARNING: src/c_api/c_api.cc:935: `ntree_limit` is deprecated, use `iteration_range` instead.
## [16:01:52] WARNING: src/c_api/c_api.cc:935: `ntree_limit` is deprecated, use `iteration_range` instead.
## [16:01:52] WARNING: src/c_api/c_api.cc:935: `ntree_limit` is deprecated, use `iteration_range` instead.
## [16:01:52] WARNING: src/c_api/c_api.cc:935: `ntree_limit` is deprecated, use `iteration_range` instead.
## [16:01:52] WARNING: src/c_api/c_api.cc:935: `ntree_limit` is deprecated, use `iteration_range` instead.
## [16:01:52] WARNING: src/c_api/c_api.cc:935: `ntree_limit` is deprecated, use `iteration_range` instead.
## [16:01:52] WARNING: src/c_api/c_api.cc:935: `ntree_limit` is deprecated, use `iteration_range` instead.
## [16:01:52] WARNING: src/c_api/c_api.cc:935: `ntree_limit` is deprecated, use `iteration_range` instead.
## [16:01:53] WARNING: src/c_api/c_api.cc:935: `ntree_limit` is deprecated, use `iteration_range` instead.
## [16:01:53] WARNING: src/c_api/c_api.cc:935: `ntree_limit` is deprecated, use `iteration_range` instead.
## [16:01:55] WARNING: src/c_api/c_api.cc:935: `ntree_limit` is deprecated, use `iteration_range` instead.
## [16:01:55] WARNING: src/c_api/c_api.cc:935: `ntree_limit` is deprecated, use `iteration_range` instead.
## [16:01:56] WARNING: src/c_api/c_api.cc:935: `ntree_limit` is deprecated, use `iteration_range` instead.
## [16:01:56] WARNING: src/c_api/c_api.cc:935: `ntree_limit` is deprecated, use `iteration_range` instead.
## [16:01:56] WARNING: src/c_api/c_api.cc:935: `ntree_limit` is deprecated, use `iteration_range` instead.
## [16:01:56] WARNING: src/c_api/c_api.cc:935: `ntree_limit` is deprecated, use `iteration_range` instead.
## [16:01:56] WARNING: src/c_api/c_api.cc:935: `ntree_limit` is deprecated, use `iteration_range` instead.
## [16:01:56] WARNING: src/c_api/c_api.cc:935: `ntree_limit` is deprecated, use `iteration_range` instead.
## [16:01:56] WARNING: src/c_api/c_api.cc:935: `ntree_limit` is deprecated, use `iteration_range` instead.
## [16:01:56] WARNING: src/c_api/c_api.cc:935: `ntree_limit` is deprecated, use `iteration_range` instead.
## [16:01:56] WARNING: src/c_api/c_api.cc:935: `ntree_limit` is deprecated, use `iteration_range` instead.
## [16:01:56] WARNING: src/c_api/c_api.cc:935: `ntree_limit` is deprecated, use `iteration_range` instead.
## [16:01:56] WARNING: src/c_api/c_api.cc:935: `ntree_limit` is deprecated, use `iteration_range` instead.
## [16:01:56] WARNING: src/c_api/c_api.cc:935: `ntree_limit` is deprecated, use `iteration_range` instead.
## [16:01:57] WARNING: src/c_api/c_api.cc:935: `ntree_limit` is deprecated, use `iteration_range` instead.
## [16:01:57] WARNING: src/c_api/c_api.cc:935: `ntree_limit` is deprecated, use `iteration_range` instead.
## [16:01:57] WARNING: src/c_api/c_api.cc:935: `ntree_limit` is deprecated, use `iteration_range` instead.
## [16:01:57] WARNING: src/c_api/c_api.cc:935: `ntree_limit` is deprecated, use `iteration_range` instead.
## [16:01:57] WARNING: src/c_api/c_api.cc:935: `ntree_limit` is deprecated, use `iteration_range` instead.
## [16:01:57] WARNING: src/c_api/c_api.cc:935: `ntree_limit` is deprecated, use `iteration_range` instead.
## [16:01:57] WARNING: src/c_api/c_api.cc:935: `ntree_limit` is deprecated, use `iteration_range` instead.
## [16:01:57] WARNING: src/c_api/c_api.cc:935: `ntree_limit` is deprecated, use `iteration_range` instead.
## [16:01:57] WARNING: src/c_api/c_api.cc:935: `ntree_limit` is deprecated, use `iteration_range` instead.
## [16:01:57] WARNING: src/c_api/c_api.cc:935: `ntree_limit` is deprecated, use `iteration_range` instead.
## [16:01:58] WARNING: src/c_api/c_api.cc:935: `ntree_limit` is deprecated, use `iteration_range` instead.
## [16:01:58] WARNING: src/c_api/c_api.cc:935: `ntree_limit` is deprecated, use `iteration_range` instead.
## [16:01:58] WARNING: src/c_api/c_api.cc:935: `ntree_limit` is deprecated, use `iteration_range` instead.
## [16:01:58] WARNING: src/c_api/c_api.cc:935: `ntree_limit` is deprecated, use `iteration_range` instead.
## [16:01:58] WARNING: src/c_api/c_api.cc:935: `ntree_limit` is deprecated, use `iteration_range` instead.
## [16:01:58] WARNING: src/c_api/c_api.cc:935: `ntree_limit` is deprecated, use `iteration_range` instead.
## [16:01:58] WARNING: src/c_api/c_api.cc:935: `ntree_limit` is deprecated, use `iteration_range` instead.
## [16:01:58] WARNING: src/c_api/c_api.cc:935: `ntree_limit` is deprecated, use `iteration_range` instead.
## [16:01:58] WARNING: src/c_api/c_api.cc:935: `ntree_limit` is deprecated, use `iteration_range` instead.
## [16:01:58] WARNING: src/c_api/c_api.cc:935: `ntree_limit` is deprecated, use `iteration_range` instead.
## [16:01:59] WARNING: src/c_api/c_api.cc:935: `ntree_limit` is deprecated, use `iteration_range` instead.
## [16:01:59] WARNING: src/c_api/c_api.cc:935: `ntree_limit` is deprecated, use `iteration_range` instead.
## [16:01:59] WARNING: src/c_api/c_api.cc:935: `ntree_limit` is deprecated, use `iteration_range` instead.
## [16:01:59] WARNING: src/c_api/c_api.cc:935: `ntree_limit` is deprecated, use `iteration_range` instead.
## [16:01:59] WARNING: src/c_api/c_api.cc:935: `ntree_limit` is deprecated, use `iteration_range` instead.
## [16:01:59] WARNING: src/c_api/c_api.cc:935: `ntree_limit` is deprecated, use `iteration_range` instead.
## [16:01:59] WARNING: src/c_api/c_api.cc:935: `ntree_limit` is deprecated, use `iteration_range` instead.
## [16:01:59] WARNING: src/c_api/c_api.cc:935: `ntree_limit` is deprecated, use `iteration_range` instead.
## [16:01:59] WARNING: src/c_api/c_api.cc:935: `ntree_limit` is deprecated, use `iteration_range` instead.
## [16:01:59] WARNING: src/c_api/c_api.cc:935: `ntree_limit` is deprecated, use `iteration_range` instead.
## [16:01:59] WARNING: src/c_api/c_api.cc:935: `ntree_limit` is deprecated, use `iteration_range` instead.
## [16:01:59] WARNING: src/c_api/c_api.cc:935: `ntree_limit` is deprecated, use `iteration_range` instead.
## [16:02:00] WARNING: src/c_api/c_api.cc:935: `ntree_limit` is deprecated, use `iteration_range` instead.
## [16:02:00] WARNING: src/c_api/c_api.cc:935: `ntree_limit` is deprecated, use `iteration_range` instead.
## [16:02:00] WARNING: src/c_api/c_api.cc:935: `ntree_limit` is deprecated, use `iteration_range` instead.
## [16:02:00] WARNING: src/c_api/c_api.cc:935: `ntree_limit` is deprecated, use `iteration_range` instead.
## [16:02:00] WARNING: src/c_api/c_api.cc:935: `ntree_limit` is deprecated, use `iteration_range` instead.
## [16:02:00] WARNING: src/c_api/c_api.cc:935: `ntree_limit` is deprecated, use `iteration_range` instead.
## [16:02:00] WARNING: src/c_api/c_api.cc:935: `ntree_limit` is deprecated, use `iteration_range` instead.
## [16:02:00] WARNING: src/c_api/c_api.cc:935: `ntree_limit` is deprecated, use `iteration_range` instead.
## [16:02:00] WARNING: src/c_api/c_api.cc:935: `ntree_limit` is deprecated, use `iteration_range` instead.
## [16:02:00] WARNING: src/c_api/c_api.cc:935: `ntree_limit` is deprecated, use `iteration_range` instead.
## [16:02:01] WARNING: src/c_api/c_api.cc:935: `ntree_limit` is deprecated, use `iteration_range` instead.
## [16:02:01] WARNING: src/c_api/c_api.cc:935: `ntree_limit` is deprecated, use `iteration_range` instead.
## [16:02:01] WARNING: src/c_api/c_api.cc:935: `ntree_limit` is deprecated, use `iteration_range` instead.
## [16:02:01] WARNING: src/c_api/c_api.cc:935: `ntree_limit` is deprecated, use `iteration_range` instead.
## [16:02:01] WARNING: src/c_api/c_api.cc:935: `ntree_limit` is deprecated, use `iteration_range` instead.
## [16:02:01] WARNING: src/c_api/c_api.cc:935: `ntree_limit` is deprecated, use `iteration_range` instead.
## [16:02:01] WARNING: src/c_api/c_api.cc:935: `ntree_limit` is deprecated, use `iteration_range` instead.
## [16:02:01] WARNING: src/c_api/c_api.cc:935: `ntree_limit` is deprecated, use `iteration_range` instead.
## [16:02:02] WARNING: src/c_api/c_api.cc:935: `ntree_limit` is deprecated, use `iteration_range` instead.
## [16:02:02] WARNING: src/c_api/c_api.cc:935: `ntree_limit` is deprecated, use `iteration_range` instead.
## [16:02:02] WARNING: src/c_api/c_api.cc:935: `ntree_limit` is deprecated, use `iteration_range` instead.
## [16:02:02] WARNING: src/c_api/c_api.cc:935: `ntree_limit` is deprecated, use `iteration_range` instead.
## [16:02:02] WARNING: src/c_api/c_api.cc:935: `ntree_limit` is deprecated, use `iteration_range` instead.
## [16:02:02] WARNING: src/c_api/c_api.cc:935: `ntree_limit` is deprecated, use `iteration_range` instead.
## [16:02:02] WARNING: src/c_api/c_api.cc:935: `ntree_limit` is deprecated, use `iteration_range` instead.
## [16:02:02] WARNING: src/c_api/c_api.cc:935: `ntree_limit` is deprecated, use `iteration_range` instead.
## [16:02:02] WARNING: src/c_api/c_api.cc:935: `ntree_limit` is deprecated, use `iteration_range` instead.
## [16:02:02] WARNING: src/c_api/c_api.cc:935: `ntree_limit` is deprecated, use `iteration_range` instead.
## [16:02:02] WARNING: src/c_api/c_api.cc:935: `ntree_limit` is deprecated, use `iteration_range` instead.
## [16:02:02] WARNING: src/c_api/c_api.cc:935: `ntree_limit` is deprecated, use `iteration_range` instead.
## [16:02:03] WARNING: src/c_api/c_api.cc:935: `ntree_limit` is deprecated, use `iteration_range` instead.
## [16:02:03] WARNING: src/c_api/c_api.cc:935: `ntree_limit` is deprecated, use `iteration_range` instead.
## [16:02:03] WARNING: src/c_api/c_api.cc:935: `ntree_limit` is deprecated, use `iteration_range` instead.
## [16:02:03] WARNING: src/c_api/c_api.cc:935: `ntree_limit` is deprecated, use `iteration_range` instead.
## [16:02:03] WARNING: src/c_api/c_api.cc:935: `ntree_limit` is deprecated, use `iteration_range` instead.
## [16:02:03] WARNING: src/c_api/c_api.cc:935: `ntree_limit` is deprecated, use `iteration_range` instead.
## [16:02:03] WARNING: src/c_api/c_api.cc:935: `ntree_limit` is deprecated, use `iteration_range` instead.
## [16:02:03] WARNING: src/c_api/c_api.cc:935: `ntree_limit` is deprecated, use `iteration_range` instead.
## [16:02:03] WARNING: src/c_api/c_api.cc:935: `ntree_limit` is deprecated, use `iteration_range` instead.
## [16:02:03] WARNING: src/c_api/c_api.cc:935: `ntree_limit` is deprecated, use `iteration_range` instead.
## [16:02:03] WARNING: src/c_api/c_api.cc:935: `ntree_limit` is deprecated, use `iteration_range` instead.
## [16:02:03] WARNING: src/c_api/c_api.cc:935: `ntree_limit` is deprecated, use `iteration_range` instead.
## [16:02:04] WARNING: src/c_api/c_api.cc:935: `ntree_limit` is deprecated, use `iteration_range` instead.
## [16:02:04] WARNING: src/c_api/c_api.cc:935: `ntree_limit` is deprecated, use `iteration_range` instead.
## [16:02:04] WARNING: src/c_api/c_api.cc:935: `ntree_limit` is deprecated, use `iteration_range` instead.
## [16:02:04] WARNING: src/c_api/c_api.cc:935: `ntree_limit` is deprecated, use `iteration_range` instead.
## [16:02:04] WARNING: src/c_api/c_api.cc:935: `ntree_limit` is deprecated, use `iteration_range` instead.
## [16:02:04] WARNING: src/c_api/c_api.cc:935: `ntree_limit` is deprecated, use `iteration_range` instead.
## [16:02:04] WARNING: src/c_api/c_api.cc:935: `ntree_limit` is deprecated, use `iteration_range` instead.
## [16:02:04] WARNING: src/c_api/c_api.cc:935: `ntree_limit` is deprecated, use `iteration_range` instead.
## [16:02:04] WARNING: src/c_api/c_api.cc:935: `ntree_limit` is deprecated, use `iteration_range` instead.
## [16:02:04] WARNING: src/c_api/c_api.cc:935: `ntree_limit` is deprecated, use `iteration_range` instead.
## [16:02:05] WARNING: src/c_api/c_api.cc:935: `ntree_limit` is deprecated, use `iteration_range` instead.
## [16:02:05] WARNING: src/c_api/c_api.cc:935: `ntree_limit` is deprecated, use `iteration_range` instead.
## [16:02:05] WARNING: src/c_api/c_api.cc:935: `ntree_limit` is deprecated, use `iteration_range` instead.
## [16:02:05] WARNING: src/c_api/c_api.cc:935: `ntree_limit` is deprecated, use `iteration_range` instead.
## [16:02:05] WARNING: src/c_api/c_api.cc:935: `ntree_limit` is deprecated, use `iteration_range` instead.
## [16:02:05] WARNING: src/c_api/c_api.cc:935: `ntree_limit` is deprecated, use `iteration_range` instead.
## [16:02:05] WARNING: src/c_api/c_api.cc:935: `ntree_limit` is deprecated, use `iteration_range` instead.
## [16:02:05] WARNING: src/c_api/c_api.cc:935: `ntree_limit` is deprecated, use `iteration_range` instead.
## [16:02:06] WARNING: src/c_api/c_api.cc:935: `ntree_limit` is deprecated, use `iteration_range` instead.
## [16:02:06] WARNING: src/c_api/c_api.cc:935: `ntree_limit` is deprecated, use `iteration_range` instead.
## [16:02:06] WARNING: src/c_api/c_api.cc:935: `ntree_limit` is deprecated, use `iteration_range` instead.
## [16:02:06] WARNING: src/c_api/c_api.cc:935: `ntree_limit` is deprecated, use `iteration_range` instead.
## [16:02:06] WARNING: src/c_api/c_api.cc:935: `ntree_limit` is deprecated, use `iteration_range` instead.
## [16:02:06] WARNING: src/c_api/c_api.cc:935: `ntree_limit` is deprecated, use `iteration_range` instead.
## [16:02:06] WARNING: src/c_api/c_api.cc:935: `ntree_limit` is deprecated, use `iteration_range` instead.
## [16:02:06] WARNING: src/c_api/c_api.cc:935: `ntree_limit` is deprecated, use `iteration_range` instead.
## [16:02:06] WARNING: src/c_api/c_api.cc:935: `ntree_limit` is deprecated, use `iteration_range` instead.
## [16:02:06] WARNING: src/c_api/c_api.cc:935: `ntree_limit` is deprecated, use `iteration_range` instead.
## [16:02:06] WARNING: src/c_api/c_api.cc:935: `ntree_limit` is deprecated, use `iteration_range` instead.
## [16:02:06] WARNING: src/c_api/c_api.cc:935: `ntree_limit` is deprecated, use `iteration_range` instead.
## [16:02:06] WARNING: src/c_api/c_api.cc:935: `ntree_limit` is deprecated, use `iteration_range` instead.
## [16:02:06] WARNING: src/c_api/c_api.cc:935: `ntree_limit` is deprecated, use `iteration_range` instead.
## [16:02:06] WARNING: src/c_api/c_api.cc:935: `ntree_limit` is deprecated, use `iteration_range` instead.
## [16:02:06] WARNING: src/c_api/c_api.cc:935: `ntree_limit` is deprecated, use `iteration_range` instead.
## [16:02:07] WARNING: src/c_api/c_api.cc:935: `ntree_limit` is deprecated, use `iteration_range` instead.
## [16:02:07] WARNING: src/c_api/c_api.cc:935: `ntree_limit` is deprecated, use `iteration_range` instead.
## [16:02:07] WARNING: src/c_api/c_api.cc:935: `ntree_limit` is deprecated, use `iteration_range` instead.
## [16:02:07] WARNING: src/c_api/c_api.cc:935: `ntree_limit` is deprecated, use `iteration_range` instead.
## [16:02:07] WARNING: src/c_api/c_api.cc:935: `ntree_limit` is deprecated, use `iteration_range` instead.
## [16:02:07] WARNING: src/c_api/c_api.cc:935: `ntree_limit` is deprecated, use `iteration_range` instead.
## [16:02:07] WARNING: src/c_api/c_api.cc:935: `ntree_limit` is deprecated, use `iteration_range` instead.
## [16:02:07] WARNING: src/c_api/c_api.cc:935: `ntree_limit` is deprecated, use `iteration_range` instead.
## [16:02:07] WARNING: src/c_api/c_api.cc:935: `ntree_limit` is deprecated, use `iteration_range` instead.
## [16:02:07] WARNING: src/c_api/c_api.cc:935: `ntree_limit` is deprecated, use `iteration_range` instead.
## [16:02:08] WARNING: src/c_api/c_api.cc:935: `ntree_limit` is deprecated, use `iteration_range` instead.
## [16:02:08] WARNING: src/c_api/c_api.cc:935: `ntree_limit` is deprecated, use `iteration_range` instead.
## [16:02:08] WARNING: src/c_api/c_api.cc:935: `ntree_limit` is deprecated, use `iteration_range` instead.
## [16:02:08] WARNING: src/c_api/c_api.cc:935: `ntree_limit` is deprecated, use `iteration_range` instead.
## [16:02:08] WARNING: src/c_api/c_api.cc:935: `ntree_limit` is deprecated, use `iteration_range` instead.
## [16:02:08] WARNING: src/c_api/c_api.cc:935: `ntree_limit` is deprecated, use `iteration_range` instead.
## [16:02:08] WARNING: src/c_api/c_api.cc:935: `ntree_limit` is deprecated, use `iteration_range` instead.
## [16:02:08] WARNING: src/c_api/c_api.cc:935: `ntree_limit` is deprecated, use `iteration_range` instead.
## [16:02:08] WARNING: src/c_api/c_api.cc:935: `ntree_limit` is deprecated, use `iteration_range` instead.
## [16:02:08] WARNING: src/c_api/c_api.cc:935: `ntree_limit` is deprecated, use `iteration_range` instead.
## [16:02:09] WARNING: src/c_api/c_api.cc:935: `ntree_limit` is deprecated, use `iteration_range` instead.
## [16:02:09] WARNING: src/c_api/c_api.cc:935: `ntree_limit` is deprecated, use `iteration_range` instead.
## [16:02:09] WARNING: src/c_api/c_api.cc:935: `ntree_limit` is deprecated, use `iteration_range` instead.
## [16:02:09] WARNING: src/c_api/c_api.cc:935: `ntree_limit` is deprecated, use `iteration_range` instead.
## [16:02:09] WARNING: src/c_api/c_api.cc:935: `ntree_limit` is deprecated, use `iteration_range` instead.
## [16:02:09] WARNING: src/c_api/c_api.cc:935: `ntree_limit` is deprecated, use `iteration_range` instead.
## [16:02:09] WARNING: src/c_api/c_api.cc:935: `ntree_limit` is deprecated, use `iteration_range` instead.
## [16:02:09] WARNING: src/c_api/c_api.cc:935: `ntree_limit` is deprecated, use `iteration_range` instead.
## [16:02:09] WARNING: src/c_api/c_api.cc:935: `ntree_limit` is deprecated, use `iteration_range` instead.
## [16:02:09] WARNING: src/c_api/c_api.cc:935: `ntree_limit` is deprecated, use `iteration_range` instead.
## [16:02:09] WARNING: src/c_api/c_api.cc:935: `ntree_limit` is deprecated, use `iteration_range` instead.
## [16:02:09] WARNING: src/c_api/c_api.cc:935: `ntree_limit` is deprecated, use `iteration_range` instead.
## [16:02:09] WARNING: src/c_api/c_api.cc:935: `ntree_limit` is deprecated, use `iteration_range` instead.
## [16:02:09] WARNING: src/c_api/c_api.cc:935: `ntree_limit` is deprecated, use `iteration_range` instead.
## [16:02:10] WARNING: src/c_api/c_api.cc:935: `ntree_limit` is deprecated, use `iteration_range` instead.
## [16:02:10] WARNING: src/c_api/c_api.cc:935: `ntree_limit` is deprecated, use `iteration_range` instead.
## [16:02:10] WARNING: src/c_api/c_api.cc:935: `ntree_limit` is deprecated, use `iteration_range` instead.
## [16:02:10] WARNING: src/c_api/c_api.cc:935: `ntree_limit` is deprecated, use `iteration_range` instead.
## [16:02:10] WARNING: src/c_api/c_api.cc:935: `ntree_limit` is deprecated, use `iteration_range` instead.
## [16:02:10] WARNING: src/c_api/c_api.cc:935: `ntree_limit` is deprecated, use `iteration_range` instead.
## [16:02:10] WARNING: src/c_api/c_api.cc:935: `ntree_limit` is deprecated, use `iteration_range` instead.
## [16:02:10] WARNING: src/c_api/c_api.cc:935: `ntree_limit` is deprecated, use `iteration_range` instead.
## [16:02:10] WARNING: src/c_api/c_api.cc:935: `ntree_limit` is deprecated, use `iteration_range` instead.
## [16:02:10] WARNING: src/c_api/c_api.cc:935: `ntree_limit` is deprecated, use `iteration_range` instead.
## [16:02:11] WARNING: src/c_api/c_api.cc:935: `ntree_limit` is deprecated, use `iteration_range` instead.
## [16:02:11] WARNING: src/c_api/c_api.cc:935: `ntree_limit` is deprecated, use `iteration_range` instead.
## [16:02:11] WARNING: src/c_api/c_api.cc:935: `ntree_limit` is deprecated, use `iteration_range` instead.
## [16:02:11] WARNING: src/c_api/c_api.cc:935: `ntree_limit` is deprecated, use `iteration_range` instead.
## [16:02:11] WARNING: src/c_api/c_api.cc:935: `ntree_limit` is deprecated, use `iteration_range` instead.
## [16:02:11] WARNING: src/c_api/c_api.cc:935: `ntree_limit` is deprecated, use `iteration_range` instead.
## [16:02:11] WARNING: src/c_api/c_api.cc:935: `ntree_limit` is deprecated, use `iteration_range` instead.
## [16:02:11] WARNING: src/c_api/c_api.cc:935: `ntree_limit` is deprecated, use `iteration_range` instead.
## [16:02:12] WARNING: src/c_api/c_api.cc:935: `ntree_limit` is deprecated, use `iteration_range` instead.
## [16:02:12] WARNING: src/c_api/c_api.cc:935: `ntree_limit` is deprecated, use `iteration_range` instead.
## [16:02:12] WARNING: src/c_api/c_api.cc:935: `ntree_limit` is deprecated, use `iteration_range` instead.
## [16:02:12] WARNING: src/c_api/c_api.cc:935: `ntree_limit` is deprecated, use `iteration_range` instead.
## [16:02:12] WARNING: src/c_api/c_api.cc:935: `ntree_limit` is deprecated, use `iteration_range` instead.
## [16:02:12] WARNING: src/c_api/c_api.cc:935: `ntree_limit` is deprecated, use `iteration_range` instead.
## [16:02:12] WARNING: src/c_api/c_api.cc:935: `ntree_limit` is deprecated, use `iteration_range` instead.
## [16:02:12] WARNING: src/c_api/c_api.cc:935: `ntree_limit` is deprecated, use `iteration_range` instead.
## [16:02:12] WARNING: src/c_api/c_api.cc:935: `ntree_limit` is deprecated, use `iteration_range` instead.
## [16:02:12] WARNING: src/c_api/c_api.cc:935: `ntree_limit` is deprecated, use `iteration_range` instead.
## [16:02:12] WARNING: src/c_api/c_api.cc:935: `ntree_limit` is deprecated, use `iteration_range` instead.
## [16:02:12] WARNING: src/c_api/c_api.cc:935: `ntree_limit` is deprecated, use `iteration_range` instead.
## [16:02:12] WARNING: src/c_api/c_api.cc:935: `ntree_limit` is deprecated, use `iteration_range` instead.
## [16:02:12] WARNING: src/c_api/c_api.cc:935: `ntree_limit` is deprecated, use `iteration_range` instead.
## [16:02:13] WARNING: src/c_api/c_api.cc:935: `ntree_limit` is deprecated, use `iteration_range` instead.
## [16:02:13] WARNING: src/c_api/c_api.cc:935: `ntree_limit` is deprecated, use `iteration_range` instead.
## [16:02:13] WARNING: src/c_api/c_api.cc:935: `ntree_limit` is deprecated, use `iteration_range` instead.
## [16:02:13] WARNING: src/c_api/c_api.cc:935: `ntree_limit` is deprecated, use `iteration_range` instead.
## [16:02:13] WARNING: src/c_api/c_api.cc:935: `ntree_limit` is deprecated, use `iteration_range` instead.
## [16:02:13] WARNING: src/c_api/c_api.cc:935: `ntree_limit` is deprecated, use `iteration_range` instead.
## [16:02:13] WARNING: src/c_api/c_api.cc:935: `ntree_limit` is deprecated, use `iteration_range` instead.
## [16:02:13] WARNING: src/c_api/c_api.cc:935: `ntree_limit` is deprecated, use `iteration_range` instead.
## [16:02:13] WARNING: src/c_api/c_api.cc:935: `ntree_limit` is deprecated, use `iteration_range` instead.
## [16:02:13] WARNING: src/c_api/c_api.cc:935: `ntree_limit` is deprecated, use `iteration_range` instead.
## [16:02:13] WARNING: src/c_api/c_api.cc:935: `ntree_limit` is deprecated, use `iteration_range` instead.
## [16:02:13] WARNING: src/c_api/c_api.cc:935: `ntree_limit` is deprecated, use `iteration_range` instead.
## [16:02:14] WARNING: src/c_api/c_api.cc:935: `ntree_limit` is deprecated, use `iteration_range` instead.
## [16:02:14] WARNING: src/c_api/c_api.cc:935: `ntree_limit` is deprecated, use `iteration_range` instead.
## [16:02:14] WARNING: src/c_api/c_api.cc:935: `ntree_limit` is deprecated, use `iteration_range` instead.
## [16:02:14] WARNING: src/c_api/c_api.cc:935: `ntree_limit` is deprecated, use `iteration_range` instead.
## [16:02:14] WARNING: src/c_api/c_api.cc:935: `ntree_limit` is deprecated, use `iteration_range` instead.
## [16:02:14] WARNING: src/c_api/c_api.cc:935: `ntree_limit` is deprecated, use `iteration_range` instead.
## [16:02:14] WARNING: src/c_api/c_api.cc:935: `ntree_limit` is deprecated, use `iteration_range` instead.
## [16:02:14] WARNING: src/c_api/c_api.cc:935: `ntree_limit` is deprecated, use `iteration_range` instead.
## [16:02:14] WARNING: src/c_api/c_api.cc:935: `ntree_limit` is deprecated, use `iteration_range` instead.
## [16:02:14] WARNING: src/c_api/c_api.cc:935: `ntree_limit` is deprecated, use `iteration_range` instead.
## [16:02:15] WARNING: src/c_api/c_api.cc:935: `ntree_limit` is deprecated, use `iteration_range` instead.
## [16:02:15] WARNING: src/c_api/c_api.cc:935: `ntree_limit` is deprecated, use `iteration_range` instead.
## [16:02:15] WARNING: src/c_api/c_api.cc:935: `ntree_limit` is deprecated, use `iteration_range` instead.
## [16:02:15] WARNING: src/c_api/c_api.cc:935: `ntree_limit` is deprecated, use `iteration_range` instead.
## [16:02:15] WARNING: src/c_api/c_api.cc:935: `ntree_limit` is deprecated, use `iteration_range` instead.
## [16:02:15] WARNING: src/c_api/c_api.cc:935: `ntree_limit` is deprecated, use `iteration_range` instead.
## [16:02:15] WARNING: src/c_api/c_api.cc:935: `ntree_limit` is deprecated, use `iteration_range` instead.
## [16:02:15] WARNING: src/c_api/c_api.cc:935: `ntree_limit` is deprecated, use `iteration_range` instead.
## [16:02:15] WARNING: src/c_api/c_api.cc:935: `ntree_limit` is deprecated, use `iteration_range` instead.
## [16:02:15] WARNING: src/c_api/c_api.cc:935: `ntree_limit` is deprecated, use `iteration_range` instead.
## [16:02:15] WARNING: src/c_api/c_api.cc:935: `ntree_limit` is deprecated, use `iteration_range` instead.
## [16:02:15] WARNING: src/c_api/c_api.cc:935: `ntree_limit` is deprecated, use `iteration_range` instead.
## [16:02:16] WARNING: src/c_api/c_api.cc:935: `ntree_limit` is deprecated, use `iteration_range` instead.
## [16:02:16] WARNING: src/c_api/c_api.cc:935: `ntree_limit` is deprecated, use `iteration_range` instead.
## [16:02:16] WARNING: src/c_api/c_api.cc:935: `ntree_limit` is deprecated, use `iteration_range` instead.
## [16:02:16] WARNING: src/c_api/c_api.cc:935: `ntree_limit` is deprecated, use `iteration_range` instead.
## [16:02:16] WARNING: src/c_api/c_api.cc:935: `ntree_limit` is deprecated, use `iteration_range` instead.
## [16:02:16] WARNING: src/c_api/c_api.cc:935: `ntree_limit` is deprecated, use `iteration_range` instead.
## [16:02:16] WARNING: src/c_api/c_api.cc:935: `ntree_limit` is deprecated, use `iteration_range` instead.
## [16:02:16] WARNING: src/c_api/c_api.cc:935: `ntree_limit` is deprecated, use `iteration_range` instead.
## [16:02:16] WARNING: src/c_api/c_api.cc:935: `ntree_limit` is deprecated, use `iteration_range` instead.
## [16:02:16] WARNING: src/c_api/c_api.cc:935: `ntree_limit` is deprecated, use `iteration_range` instead.
## [16:02:16] WARNING: src/c_api/c_api.cc:935: `ntree_limit` is deprecated, use `iteration_range` instead.
## [16:02:16] WARNING: src/c_api/c_api.cc:935: `ntree_limit` is deprecated, use `iteration_range` instead.
## [16:02:17] WARNING: src/c_api/c_api.cc:935: `ntree_limit` is deprecated, use `iteration_range` instead.
## [16:02:17] WARNING: src/c_api/c_api.cc:935: `ntree_limit` is deprecated, use `iteration_range` instead.
## [16:02:17] WARNING: src/c_api/c_api.cc:935: `ntree_limit` is deprecated, use `iteration_range` instead.
## [16:02:17] WARNING: src/c_api/c_api.cc:935: `ntree_limit` is deprecated, use `iteration_range` instead.
## [16:02:17] WARNING: src/c_api/c_api.cc:935: `ntree_limit` is deprecated, use `iteration_range` instead.
## [16:02:17] WARNING: src/c_api/c_api.cc:935: `ntree_limit` is deprecated, use `iteration_range` instead.
## [16:02:17] WARNING: src/c_api/c_api.cc:935: `ntree_limit` is deprecated, use `iteration_range` instead.
## [16:02:17] WARNING: src/c_api/c_api.cc:935: `ntree_limit` is deprecated, use `iteration_range` instead.
## [16:02:17] WARNING: src/c_api/c_api.cc:935: `ntree_limit` is deprecated, use `iteration_range` instead.
## [16:02:17] WARNING: src/c_api/c_api.cc:935: `ntree_limit` is deprecated, use `iteration_range` instead.
## [16:02:18] WARNING: src/c_api/c_api.cc:935: `ntree_limit` is deprecated, use `iteration_range` instead.
## [16:02:18] WARNING: src/c_api/c_api.cc:935: `ntree_limit` is deprecated, use `iteration_range` instead.
## [16:02:18] WARNING: src/c_api/c_api.cc:935: `ntree_limit` is deprecated, use `iteration_range` instead.
## [16:02:18] WARNING: src/c_api/c_api.cc:935: `ntree_limit` is deprecated, use `iteration_range` instead.
## [16:02:18] WARNING: src/c_api/c_api.cc:935: `ntree_limit` is deprecated, use `iteration_range` instead.
## [16:02:18] WARNING: src/c_api/c_api.cc:935: `ntree_limit` is deprecated, use `iteration_range` instead.
## [16:02:18] WARNING: src/c_api/c_api.cc:935: `ntree_limit` is deprecated, use `iteration_range` instead.
## [16:02:18] WARNING: src/c_api/c_api.cc:935: `ntree_limit` is deprecated, use `iteration_range` instead.
## [16:02:19] WARNING: src/c_api/c_api.cc:935: `ntree_limit` is deprecated, use `iteration_range` instead.
## [16:02:19] WARNING: src/c_api/c_api.cc:935: `ntree_limit` is deprecated, use `iteration_range` instead.
## [16:02:19] WARNING: src/c_api/c_api.cc:935: `ntree_limit` is deprecated, use `iteration_range` instead.
## [16:02:19] WARNING: src/c_api/c_api.cc:935: `ntree_limit` is deprecated, use `iteration_range` instead.
## [16:02:19] WARNING: src/c_api/c_api.cc:935: `ntree_limit` is deprecated, use `iteration_range` instead.
## [16:02:19] WARNING: src/c_api/c_api.cc:935: `ntree_limit` is deprecated, use `iteration_range` instead.
## [16:02:19] WARNING: src/c_api/c_api.cc:935: `ntree_limit` is deprecated, use `iteration_range` instead.
## [16:02:19] WARNING: src/c_api/c_api.cc:935: `ntree_limit` is deprecated, use `iteration_range` instead.
## [16:02:19] WARNING: src/c_api/c_api.cc:935: `ntree_limit` is deprecated, use `iteration_range` instead.
## [16:02:19] WARNING: src/c_api/c_api.cc:935: `ntree_limit` is deprecated, use `iteration_range` instead.
## [16:02:19] WARNING: src/c_api/c_api.cc:935: `ntree_limit` is deprecated, use `iteration_range` instead.
## [16:02:19] WARNING: src/c_api/c_api.cc:935: `ntree_limit` is deprecated, use `iteration_range` instead.
## [16:02:19] WARNING: src/c_api/c_api.cc:935: `ntree_limit` is deprecated, use `iteration_range` instead.
## [16:02:19] WARNING: src/c_api/c_api.cc:935: `ntree_limit` is deprecated, use `iteration_range` instead.
## [16:02:20] WARNING: src/c_api/c_api.cc:935: `ntree_limit` is deprecated, use `iteration_range` instead.
## [16:02:20] WARNING: src/c_api/c_api.cc:935: `ntree_limit` is deprecated, use `iteration_range` instead.
## [16:02:20] WARNING: src/c_api/c_api.cc:935: `ntree_limit` is deprecated, use `iteration_range` instead.
## [16:02:20] WARNING: src/c_api/c_api.cc:935: `ntree_limit` is deprecated, use `iteration_range` instead.
## [16:02:20] WARNING: src/c_api/c_api.cc:935: `ntree_limit` is deprecated, use `iteration_range` instead.
## [16:02:20] WARNING: src/c_api/c_api.cc:935: `ntree_limit` is deprecated, use `iteration_range` instead.
## [16:02:20] WARNING: src/c_api/c_api.cc:935: `ntree_limit` is deprecated, use `iteration_range` instead.
## [16:02:20] WARNING: src/c_api/c_api.cc:935: `ntree_limit` is deprecated, use `iteration_range` instead.
## [16:02:20] WARNING: src/c_api/c_api.cc:935: `ntree_limit` is deprecated, use `iteration_range` instead.
## [16:02:20] WARNING: src/c_api/c_api.cc:935: `ntree_limit` is deprecated, use `iteration_range` instead.
## [16:02:20] WARNING: src/c_api/c_api.cc:935: `ntree_limit` is deprecated, use `iteration_range` instead.
## [16:02:20] WARNING: src/c_api/c_api.cc:935: `ntree_limit` is deprecated, use `iteration_range` instead.
## [16:02:21] WARNING: src/c_api/c_api.cc:935: `ntree_limit` is deprecated, use `iteration_range` instead.
## [16:02:21] WARNING: src/c_api/c_api.cc:935: `ntree_limit` is deprecated, use `iteration_range` instead.
## [16:02:21] WARNING: src/c_api/c_api.cc:935: `ntree_limit` is deprecated, use `iteration_range` instead.
## [16:02:21] WARNING: src/c_api/c_api.cc:935: `ntree_limit` is deprecated, use `iteration_range` instead.
## [16:02:21] WARNING: src/c_api/c_api.cc:935: `ntree_limit` is deprecated, use `iteration_range` instead.
## [16:02:21] WARNING: src/c_api/c_api.cc:935: `ntree_limit` is deprecated, use `iteration_range` instead.
## [16:02:21] WARNING: src/c_api/c_api.cc:935: `ntree_limit` is deprecated, use `iteration_range` instead.
## [16:02:21] WARNING: src/c_api/c_api.cc:935: `ntree_limit` is deprecated, use `iteration_range` instead.
## [16:02:22] WARNING: src/c_api/c_api.cc:935: `ntree_limit` is deprecated, use `iteration_range` instead.
## [16:02:22] WARNING: src/c_api/c_api.cc:935: `ntree_limit` is deprecated, use `iteration_range` instead.
## [16:02:24] WARNING: src/c_api/c_api.cc:935: `ntree_limit` is deprecated, use `iteration_range` instead.
## [16:02:24] WARNING: src/c_api/c_api.cc:935: `ntree_limit` is deprecated, use `iteration_range` instead.
## [16:02:24] WARNING: src/c_api/c_api.cc:935: `ntree_limit` is deprecated, use `iteration_range` instead.
## [16:02:24] WARNING: src/c_api/c_api.cc:935: `ntree_limit` is deprecated, use `iteration_range` instead.
## [16:02:25] WARNING: src/c_api/c_api.cc:935: `ntree_limit` is deprecated, use `iteration_range` instead.
## [16:02:25] WARNING: src/c_api/c_api.cc:935: `ntree_limit` is deprecated, use `iteration_range` instead.
## [16:02:25] WARNING: src/c_api/c_api.cc:935: `ntree_limit` is deprecated, use `iteration_range` instead.
## [16:02:25] WARNING: src/c_api/c_api.cc:935: `ntree_limit` is deprecated, use `iteration_range` instead.
## [16:02:25] WARNING: src/c_api/c_api.cc:935: `ntree_limit` is deprecated, use `iteration_range` instead.
## [16:02:25] WARNING: src/c_api/c_api.cc:935: `ntree_limit` is deprecated, use `iteration_range` instead.
## [16:02:25] WARNING: src/c_api/c_api.cc:935: `ntree_limit` is deprecated, use `iteration_range` instead.
## [16:02:25] WARNING: src/c_api/c_api.cc:935: `ntree_limit` is deprecated, use `iteration_range` instead.
## [16:02:25] WARNING: src/c_api/c_api.cc:935: `ntree_limit` is deprecated, use `iteration_range` instead.
## [16:02:25] WARNING: src/c_api/c_api.cc:935: `ntree_limit` is deprecated, use `iteration_range` instead.
## [16:02:25] WARNING: src/c_api/c_api.cc:935: `ntree_limit` is deprecated, use `iteration_range` instead.
## [16:02:25] WARNING: src/c_api/c_api.cc:935: `ntree_limit` is deprecated, use `iteration_range` instead.
## [16:02:26] WARNING: src/c_api/c_api.cc:935: `ntree_limit` is deprecated, use `iteration_range` instead.
## [16:02:26] WARNING: src/c_api/c_api.cc:935: `ntree_limit` is deprecated, use `iteration_range` instead.
## [16:02:26] WARNING: src/c_api/c_api.cc:935: `ntree_limit` is deprecated, use `iteration_range` instead.
## [16:02:26] WARNING: src/c_api/c_api.cc:935: `ntree_limit` is deprecated, use `iteration_range` instead.
## [16:02:26] WARNING: src/c_api/c_api.cc:935: `ntree_limit` is deprecated, use `iteration_range` instead.
## [16:02:26] WARNING: src/c_api/c_api.cc:935: `ntree_limit` is deprecated, use `iteration_range` instead.
## [16:02:26] WARNING: src/c_api/c_api.cc:935: `ntree_limit` is deprecated, use `iteration_range` instead.
## [16:02:26] WARNING: src/c_api/c_api.cc:935: `ntree_limit` is deprecated, use `iteration_range` instead.
## [16:02:26] WARNING: src/c_api/c_api.cc:935: `ntree_limit` is deprecated, use `iteration_range` instead.
## [16:02:26] WARNING: src/c_api/c_api.cc:935: `ntree_limit` is deprecated, use `iteration_range` instead.
## [16:02:27] WARNING: src/c_api/c_api.cc:935: `ntree_limit` is deprecated, use `iteration_range` instead.
## [16:02:27] WARNING: src/c_api/c_api.cc:935: `ntree_limit` is deprecated, use `iteration_range` instead.
## [16:02:27] WARNING: src/c_api/c_api.cc:935: `ntree_limit` is deprecated, use `iteration_range` instead.
## [16:02:27] WARNING: src/c_api/c_api.cc:935: `ntree_limit` is deprecated, use `iteration_range` instead.
## [16:02:27] WARNING: src/c_api/c_api.cc:935: `ntree_limit` is deprecated, use `iteration_range` instead.
## [16:02:27] WARNING: src/c_api/c_api.cc:935: `ntree_limit` is deprecated, use `iteration_range` instead.
## [16:02:27] WARNING: src/c_api/c_api.cc:935: `ntree_limit` is deprecated, use `iteration_range` instead.
## [16:02:27] WARNING: src/c_api/c_api.cc:935: `ntree_limit` is deprecated, use `iteration_range` instead.
## [16:02:28] WARNING: src/c_api/c_api.cc:935: `ntree_limit` is deprecated, use `iteration_range` instead.
## [16:02:28] WARNING: src/c_api/c_api.cc:935: `ntree_limit` is deprecated, use `iteration_range` instead.
## [16:02:28] WARNING: src/c_api/c_api.cc:935: `ntree_limit` is deprecated, use `iteration_range` instead.
## [16:02:28] WARNING: src/c_api/c_api.cc:935: `ntree_limit` is deprecated, use `iteration_range` instead.
## [16:02:28] WARNING: src/c_api/c_api.cc:935: `ntree_limit` is deprecated, use `iteration_range` instead.
## [16:02:28] WARNING: src/c_api/c_api.cc:935: `ntree_limit` is deprecated, use `iteration_range` instead.
## [16:02:28] WARNING: src/c_api/c_api.cc:935: `ntree_limit` is deprecated, use `iteration_range` instead.
## [16:02:28] WARNING: src/c_api/c_api.cc:935: `ntree_limit` is deprecated, use `iteration_range` instead.
## [16:02:28] WARNING: src/c_api/c_api.cc:935: `ntree_limit` is deprecated, use `iteration_range` instead.
## [16:02:28] WARNING: src/c_api/c_api.cc:935: `ntree_limit` is deprecated, use `iteration_range` instead.
## [16:02:28] WARNING: src/c_api/c_api.cc:935: `ntree_limit` is deprecated, use `iteration_range` instead.
## [16:02:28] WARNING: src/c_api/c_api.cc:935: `ntree_limit` is deprecated, use `iteration_range` instead.
## [16:02:28] WARNING: src/c_api/c_api.cc:935: `ntree_limit` is deprecated, use `iteration_range` instead.
## [16:02:28] WARNING: src/c_api/c_api.cc:935: `ntree_limit` is deprecated, use `iteration_range` instead.
## [16:02:29] WARNING: src/c_api/c_api.cc:935: `ntree_limit` is deprecated, use `iteration_range` instead.
## [16:02:29] WARNING: src/c_api/c_api.cc:935: `ntree_limit` is deprecated, use `iteration_range` instead.
## [16:02:29] WARNING: src/c_api/c_api.cc:935: `ntree_limit` is deprecated, use `iteration_range` instead.
## [16:02:29] WARNING: src/c_api/c_api.cc:935: `ntree_limit` is deprecated, use `iteration_range` instead.
## [16:02:29] WARNING: src/c_api/c_api.cc:935: `ntree_limit` is deprecated, use `iteration_range` instead.
## [16:02:29] WARNING: src/c_api/c_api.cc:935: `ntree_limit` is deprecated, use `iteration_range` instead.
## [16:02:29] WARNING: src/c_api/c_api.cc:935: `ntree_limit` is deprecated, use `iteration_range` instead.
## [16:02:29] WARNING: src/c_api/c_api.cc:935: `ntree_limit` is deprecated, use `iteration_range` instead.
## [16:02:29] WARNING: src/c_api/c_api.cc:935: `ntree_limit` is deprecated, use `iteration_range` instead.
## [16:02:29] WARNING: src/c_api/c_api.cc:935: `ntree_limit` is deprecated, use `iteration_range` instead.
## [16:02:29] WARNING: src/c_api/c_api.cc:935: `ntree_limit` is deprecated, use `iteration_range` instead.
## [16:02:29] WARNING: src/c_api/c_api.cc:935: `ntree_limit` is deprecated, use `iteration_range` instead.
## [16:02:30] WARNING: src/c_api/c_api.cc:935: `ntree_limit` is deprecated, use `iteration_range` instead.
## [16:02:30] WARNING: src/c_api/c_api.cc:935: `ntree_limit` is deprecated, use `iteration_range` instead.
## [16:02:30] WARNING: src/c_api/c_api.cc:935: `ntree_limit` is deprecated, use `iteration_range` instead.
## [16:02:30] WARNING: src/c_api/c_api.cc:935: `ntree_limit` is deprecated, use `iteration_range` instead.
## [16:02:30] WARNING: src/c_api/c_api.cc:935: `ntree_limit` is deprecated, use `iteration_range` instead.
## [16:02:30] WARNING: src/c_api/c_api.cc:935: `ntree_limit` is deprecated, use `iteration_range` instead.
## [16:02:30] WARNING: src/c_api/c_api.cc:935: `ntree_limit` is deprecated, use `iteration_range` instead.
## [16:02:30] WARNING: src/c_api/c_api.cc:935: `ntree_limit` is deprecated, use `iteration_range` instead.
## [16:02:31] WARNING: src/c_api/c_api.cc:935: `ntree_limit` is deprecated, use `iteration_range` instead.
## [16:02:31] WARNING: src/c_api/c_api.cc:935: `ntree_limit` is deprecated, use `iteration_range` instead.
## [16:02:31] WARNING: src/c_api/c_api.cc:935: `ntree_limit` is deprecated, use `iteration_range` instead.
## [16:02:31] WARNING: src/c_api/c_api.cc:935: `ntree_limit` is deprecated, use `iteration_range` instead.
## [16:02:31] WARNING: src/c_api/c_api.cc:935: `ntree_limit` is deprecated, use `iteration_range` instead.
## [16:02:31] WARNING: src/c_api/c_api.cc:935: `ntree_limit` is deprecated, use `iteration_range` instead.
## [16:02:31] WARNING: src/c_api/c_api.cc:935: `ntree_limit` is deprecated, use `iteration_range` instead.
## [16:02:31] WARNING: src/c_api/c_api.cc:935: `ntree_limit` is deprecated, use `iteration_range` instead.
## [16:02:31] WARNING: src/c_api/c_api.cc:935: `ntree_limit` is deprecated, use `iteration_range` instead.
## [16:02:31] WARNING: src/c_api/c_api.cc:935: `ntree_limit` is deprecated, use `iteration_range` instead.
## [16:02:31] WARNING: src/c_api/c_api.cc:935: `ntree_limit` is deprecated, use `iteration_range` instead.
## [16:02:31] WARNING: src/c_api/c_api.cc:935: `ntree_limit` is deprecated, use `iteration_range` instead.
## [16:02:31] WARNING: src/c_api/c_api.cc:935: `ntree_limit` is deprecated, use `iteration_range` instead.
## [16:02:31] WARNING: src/c_api/c_api.cc:935: `ntree_limit` is deprecated, use `iteration_range` instead.
## [16:02:32] WARNING: src/c_api/c_api.cc:935: `ntree_limit` is deprecated, use `iteration_range` instead.
## [16:02:32] WARNING: src/c_api/c_api.cc:935: `ntree_limit` is deprecated, use `iteration_range` instead.
## [16:02:32] WARNING: src/c_api/c_api.cc:935: `ntree_limit` is deprecated, use `iteration_range` instead.
## [16:02:32] WARNING: src/c_api/c_api.cc:935: `ntree_limit` is deprecated, use `iteration_range` instead.
## [16:02:32] WARNING: src/c_api/c_api.cc:935: `ntree_limit` is deprecated, use `iteration_range` instead.
## [16:02:32] WARNING: src/c_api/c_api.cc:935: `ntree_limit` is deprecated, use `iteration_range` instead.
## [16:02:32] WARNING: src/c_api/c_api.cc:935: `ntree_limit` is deprecated, use `iteration_range` instead.
## [16:02:32] WARNING: src/c_api/c_api.cc:935: `ntree_limit` is deprecated, use `iteration_range` instead.
## [16:02:32] WARNING: src/c_api/c_api.cc:935: `ntree_limit` is deprecated, use `iteration_range` instead.
## [16:02:32] WARNING: src/c_api/c_api.cc:935: `ntree_limit` is deprecated, use `iteration_range` instead.
## [16:02:32] WARNING: src/c_api/c_api.cc:935: `ntree_limit` is deprecated, use `iteration_range` instead.
## [16:02:32] WARNING: src/c_api/c_api.cc:935: `ntree_limit` is deprecated, use `iteration_range` instead.
## [16:02:33] WARNING: src/c_api/c_api.cc:935: `ntree_limit` is deprecated, use `iteration_range` instead.
## [16:02:33] WARNING: src/c_api/c_api.cc:935: `ntree_limit` is deprecated, use `iteration_range` instead.
## [16:02:33] WARNING: src/c_api/c_api.cc:935: `ntree_limit` is deprecated, use `iteration_range` instead.
## [16:02:33] WARNING: src/c_api/c_api.cc:935: `ntree_limit` is deprecated, use `iteration_range` instead.
## [16:02:33] WARNING: src/c_api/c_api.cc:935: `ntree_limit` is deprecated, use `iteration_range` instead.
## [16:02:33] WARNING: src/c_api/c_api.cc:935: `ntree_limit` is deprecated, use `iteration_range` instead.
## [16:02:33] WARNING: src/c_api/c_api.cc:935: `ntree_limit` is deprecated, use `iteration_range` instead.
## [16:02:33] WARNING: src/c_api/c_api.cc:935: `ntree_limit` is deprecated, use `iteration_range` instead.
## [16:02:34] WARNING: src/c_api/c_api.cc:935: `ntree_limit` is deprecated, use `iteration_range` instead.
## [16:02:34] WARNING: src/c_api/c_api.cc:935: `ntree_limit` is deprecated, use `iteration_range` instead.
## [16:02:34] WARNING: src/c_api/c_api.cc:935: `ntree_limit` is deprecated, use `iteration_range` instead.
## [16:02:34] WARNING: src/c_api/c_api.cc:935: `ntree_limit` is deprecated, use `iteration_range` instead.
## [16:02:34] WARNING: src/c_api/c_api.cc:935: `ntree_limit` is deprecated, use `iteration_range` instead.
## [16:02:34] WARNING: src/c_api/c_api.cc:935: `ntree_limit` is deprecated, use `iteration_range` instead.
## [16:02:34] WARNING: src/c_api/c_api.cc:935: `ntree_limit` is deprecated, use `iteration_range` instead.
## [16:02:34] WARNING: src/c_api/c_api.cc:935: `ntree_limit` is deprecated, use `iteration_range` instead.
## [16:02:34] WARNING: src/c_api/c_api.cc:935: `ntree_limit` is deprecated, use `iteration_range` instead.
## [16:02:34] WARNING: src/c_api/c_api.cc:935: `ntree_limit` is deprecated, use `iteration_range` instead.
## [16:02:34] WARNING: src/c_api/c_api.cc:935: `ntree_limit` is deprecated, use `iteration_range` instead.
## [16:02:34] WARNING: src/c_api/c_api.cc:935: `ntree_limit` is deprecated, use `iteration_range` instead.
## [16:02:35] WARNING: src/c_api/c_api.cc:935: `ntree_limit` is deprecated, use `iteration_range` instead.
## [16:02:35] WARNING: src/c_api/c_api.cc:935: `ntree_limit` is deprecated, use `iteration_range` instead.
## [16:02:35] WARNING: src/c_api/c_api.cc:935: `ntree_limit` is deprecated, use `iteration_range` instead.
## [16:02:35] WARNING: src/c_api/c_api.cc:935: `ntree_limit` is deprecated, use `iteration_range` instead.
## [16:02:35] WARNING: src/c_api/c_api.cc:935: `ntree_limit` is deprecated, use `iteration_range` instead.
## [16:02:35] WARNING: src/c_api/c_api.cc:935: `ntree_limit` is deprecated, use `iteration_range` instead.
## [16:02:35] WARNING: src/c_api/c_api.cc:935: `ntree_limit` is deprecated, use `iteration_range` instead.
## [16:02:35] WARNING: src/c_api/c_api.cc:935: `ntree_limit` is deprecated, use `iteration_range` instead.
## [16:02:35] WARNING: src/c_api/c_api.cc:935: `ntree_limit` is deprecated, use `iteration_range` instead.
## [16:02:35] WARNING: src/c_api/c_api.cc:935: `ntree_limit` is deprecated, use `iteration_range` instead.
## [16:02:35] WARNING: src/c_api/c_api.cc:935: `ntree_limit` is deprecated, use `iteration_range` instead.
## [16:02:35] WARNING: src/c_api/c_api.cc:935: `ntree_limit` is deprecated, use `iteration_range` instead.
## [16:02:36] WARNING: src/c_api/c_api.cc:935: `ntree_limit` is deprecated, use `iteration_range` instead.
## [16:02:36] WARNING: src/c_api/c_api.cc:935: `ntree_limit` is deprecated, use `iteration_range` instead.
## [16:02:36] WARNING: src/c_api/c_api.cc:935: `ntree_limit` is deprecated, use `iteration_range` instead.
## [16:02:36] WARNING: src/c_api/c_api.cc:935: `ntree_limit` is deprecated, use `iteration_range` instead.
## [16:02:36] WARNING: src/c_api/c_api.cc:935: `ntree_limit` is deprecated, use `iteration_range` instead.
## [16:02:36] WARNING: src/c_api/c_api.cc:935: `ntree_limit` is deprecated, use `iteration_range` instead.
## [16:02:36] WARNING: src/c_api/c_api.cc:935: `ntree_limit` is deprecated, use `iteration_range` instead.
## [16:02:36] WARNING: src/c_api/c_api.cc:935: `ntree_limit` is deprecated, use `iteration_range` instead.
## [16:02:36] WARNING: src/c_api/c_api.cc:935: `ntree_limit` is deprecated, use `iteration_range` instead.
## [16:02:36] WARNING: src/c_api/c_api.cc:935: `ntree_limit` is deprecated, use `iteration_range` instead.
## [16:02:37] WARNING: src/c_api/c_api.cc:935: `ntree_limit` is deprecated, use `iteration_range` instead.
## [16:02:37] WARNING: src/c_api/c_api.cc:935: `ntree_limit` is deprecated, use `iteration_range` instead.
## [16:02:37] WARNING: src/c_api/c_api.cc:935: `ntree_limit` is deprecated, use `iteration_range` instead.
## [16:02:37] WARNING: src/c_api/c_api.cc:935: `ntree_limit` is deprecated, use `iteration_range` instead.
## [16:02:37] WARNING: src/c_api/c_api.cc:935: `ntree_limit` is deprecated, use `iteration_range` instead.
## [16:02:37] WARNING: src/c_api/c_api.cc:935: `ntree_limit` is deprecated, use `iteration_range` instead.
## [16:02:37] WARNING: src/c_api/c_api.cc:935: `ntree_limit` is deprecated, use `iteration_range` instead.
## [16:02:37] WARNING: src/c_api/c_api.cc:935: `ntree_limit` is deprecated, use `iteration_range` instead.
## [16:02:37] WARNING: src/c_api/c_api.cc:935: `ntree_limit` is deprecated, use `iteration_range` instead.
## [16:02:37] WARNING: src/c_api/c_api.cc:935: `ntree_limit` is deprecated, use `iteration_range` instead.
## [16:02:38] WARNING: src/c_api/c_api.cc:935: `ntree_limit` is deprecated, use `iteration_range` instead.
## [16:02:38] WARNING: src/c_api/c_api.cc:935: `ntree_limit` is deprecated, use `iteration_range` instead.
## [16:02:38] WARNING: src/c_api/c_api.cc:935: `ntree_limit` is deprecated, use `iteration_range` instead.
## [16:02:38] WARNING: src/c_api/c_api.cc:935: `ntree_limit` is deprecated, use `iteration_range` instead.
## [16:02:38] WARNING: src/c_api/c_api.cc:935: `ntree_limit` is deprecated, use `iteration_range` instead.
## [16:02:38] WARNING: src/c_api/c_api.cc:935: `ntree_limit` is deprecated, use `iteration_range` instead.
## [16:02:38] WARNING: src/c_api/c_api.cc:935: `ntree_limit` is deprecated, use `iteration_range` instead.
## [16:02:38] WARNING: src/c_api/c_api.cc:935: `ntree_limit` is deprecated, use `iteration_range` instead.
## [16:02:38] WARNING: src/c_api/c_api.cc:935: `ntree_limit` is deprecated, use `iteration_range` instead.
## [16:02:38] WARNING: src/c_api/c_api.cc:935: `ntree_limit` is deprecated, use `iteration_range` instead.
## [16:02:38] WARNING: src/c_api/c_api.cc:935: `ntree_limit` is deprecated, use `iteration_range` instead.
## [16:02:38] WARNING: src/c_api/c_api.cc:935: `ntree_limit` is deprecated, use `iteration_range` instead.
## [16:02:38] WARNING: src/c_api/c_api.cc:935: `ntree_limit` is deprecated, use `iteration_range` instead.
## [16:02:38] WARNING: src/c_api/c_api.cc:935: `ntree_limit` is deprecated, use `iteration_range` instead.
## [16:02:39] WARNING: src/c_api/c_api.cc:935: `ntree_limit` is deprecated, use `iteration_range` instead.
## [16:02:39] WARNING: src/c_api/c_api.cc:935: `ntree_limit` is deprecated, use `iteration_range` instead.
## [16:02:39] WARNING: src/c_api/c_api.cc:935: `ntree_limit` is deprecated, use `iteration_range` instead.
## [16:02:39] WARNING: src/c_api/c_api.cc:935: `ntree_limit` is deprecated, use `iteration_range` instead.
## [16:02:39] WARNING: src/c_api/c_api.cc:935: `ntree_limit` is deprecated, use `iteration_range` instead.
## [16:02:39] WARNING: src/c_api/c_api.cc:935: `ntree_limit` is deprecated, use `iteration_range` instead.
## [16:02:39] WARNING: src/c_api/c_api.cc:935: `ntree_limit` is deprecated, use `iteration_range` instead.
## [16:02:39] WARNING: src/c_api/c_api.cc:935: `ntree_limit` is deprecated, use `iteration_range` instead.
## [16:02:40] WARNING: src/c_api/c_api.cc:935: `ntree_limit` is deprecated, use `iteration_range` instead.
## [16:02:40] WARNING: src/c_api/c_api.cc:935: `ntree_limit` is deprecated, use `iteration_range` instead.
## [16:02:40] WARNING: src/c_api/c_api.cc:935: `ntree_limit` is deprecated, use `iteration_range` instead.
## [16:02:40] WARNING: src/c_api/c_api.cc:935: `ntree_limit` is deprecated, use `iteration_range` instead.
## [16:02:40] WARNING: src/c_api/c_api.cc:935: `ntree_limit` is deprecated, use `iteration_range` instead.
## [16:02:40] WARNING: src/c_api/c_api.cc:935: `ntree_limit` is deprecated, use `iteration_range` instead.
## [16:02:40] WARNING: src/c_api/c_api.cc:935: `ntree_limit` is deprecated, use `iteration_range` instead.
## [16:02:40] WARNING: src/c_api/c_api.cc:935: `ntree_limit` is deprecated, use `iteration_range` instead.
## [16:02:40] WARNING: src/c_api/c_api.cc:935: `ntree_limit` is deprecated, use `iteration_range` instead.
## [16:02:40] WARNING: src/c_api/c_api.cc:935: `ntree_limit` is deprecated, use `iteration_range` instead.
## [16:02:41] WARNING: src/c_api/c_api.cc:935: `ntree_limit` is deprecated, use `iteration_range` instead.
## [16:02:41] WARNING: src/c_api/c_api.cc:935: `ntree_limit` is deprecated, use `iteration_range` instead.
## [16:02:41] WARNING: src/c_api/c_api.cc:935: `ntree_limit` is deprecated, use `iteration_range` instead.
## [16:02:41] WARNING: src/c_api/c_api.cc:935: `ntree_limit` is deprecated, use `iteration_range` instead.
## [16:02:41] WARNING: src/c_api/c_api.cc:935: `ntree_limit` is deprecated, use `iteration_range` instead.
## [16:02:41] WARNING: src/c_api/c_api.cc:935: `ntree_limit` is deprecated, use `iteration_range` instead.
## [16:02:41] WARNING: src/c_api/c_api.cc:935: `ntree_limit` is deprecated, use `iteration_range` instead.
## [16:02:41] WARNING: src/c_api/c_api.cc:935: `ntree_limit` is deprecated, use `iteration_range` instead.
## [16:02:41] WARNING: src/c_api/c_api.cc:935: `ntree_limit` is deprecated, use `iteration_range` instead.
## [16:02:41] WARNING: src/c_api/c_api.cc:935: `ntree_limit` is deprecated, use `iteration_range` instead.
## [16:02:41] WARNING: src/c_api/c_api.cc:935: `ntree_limit` is deprecated, use `iteration_range` instead.
## [16:02:41] WARNING: src/c_api/c_api.cc:935: `ntree_limit` is deprecated, use `iteration_range` instead.
## [16:02:41] WARNING: src/c_api/c_api.cc:935: `ntree_limit` is deprecated, use `iteration_range` instead.
## [16:02:41] WARNING: src/c_api/c_api.cc:935: `ntree_limit` is deprecated, use `iteration_range` instead.
## [16:02:42] WARNING: src/c_api/c_api.cc:935: `ntree_limit` is deprecated, use `iteration_range` instead.
## [16:02:42] WARNING: src/c_api/c_api.cc:935: `ntree_limit` is deprecated, use `iteration_range` instead.
## [16:02:42] WARNING: src/c_api/c_api.cc:935: `ntree_limit` is deprecated, use `iteration_range` instead.
## [16:02:42] WARNING: src/c_api/c_api.cc:935: `ntree_limit` is deprecated, use `iteration_range` instead.
## [16:02:42] WARNING: src/c_api/c_api.cc:935: `ntree_limit` is deprecated, use `iteration_range` instead.
## [16:02:42] WARNING: src/c_api/c_api.cc:935: `ntree_limit` is deprecated, use `iteration_range` instead.
## [16:02:42] WARNING: src/c_api/c_api.cc:935: `ntree_limit` is deprecated, use `iteration_range` instead.
## [16:02:42] WARNING: src/c_api/c_api.cc:935: `ntree_limit` is deprecated, use `iteration_range` instead.
## [16:02:42] WARNING: src/c_api/c_api.cc:935: `ntree_limit` is deprecated, use `iteration_range` instead.
## [16:02:42] WARNING: src/c_api/c_api.cc:935: `ntree_limit` is deprecated, use `iteration_range` instead.
## [16:02:42] WARNING: src/c_api/c_api.cc:935: `ntree_limit` is deprecated, use `iteration_range` instead.
## [16:02:42] WARNING: src/c_api/c_api.cc:935: `ntree_limit` is deprecated, use `iteration_range` instead.
## [16:02:43] WARNING: src/c_api/c_api.cc:935: `ntree_limit` is deprecated, use `iteration_range` instead.
## [16:02:43] WARNING: src/c_api/c_api.cc:935: `ntree_limit` is deprecated, use `iteration_range` instead.
## [16:02:43] WARNING: src/c_api/c_api.cc:935: `ntree_limit` is deprecated, use `iteration_range` instead.
## [16:02:43] WARNING: src/c_api/c_api.cc:935: `ntree_limit` is deprecated, use `iteration_range` instead.
## [16:02:43] WARNING: src/c_api/c_api.cc:935: `ntree_limit` is deprecated, use `iteration_range` instead.
## [16:02:43] WARNING: src/c_api/c_api.cc:935: `ntree_limit` is deprecated, use `iteration_range` instead.
## [16:02:43] WARNING: src/c_api/c_api.cc:935: `ntree_limit` is deprecated, use `iteration_range` instead.
## [16:02:43] WARNING: src/c_api/c_api.cc:935: `ntree_limit` is deprecated, use `iteration_range` instead.
## [16:02:44] WARNING: src/c_api/c_api.cc:935: `ntree_limit` is deprecated, use `iteration_range` instead.
## [16:02:44] WARNING: src/c_api/c_api.cc:935: `ntree_limit` is deprecated, use `iteration_range` instead.
## [16:02:44] WARNING: src/c_api/c_api.cc:935: `ntree_limit` is deprecated, use `iteration_range` instead.
## [16:02:44] WARNING: src/c_api/c_api.cc:935: `ntree_limit` is deprecated, use `iteration_range` instead.
## [16:02:44] WARNING: src/c_api/c_api.cc:935: `ntree_limit` is deprecated, use `iteration_range` instead.
## [16:02:44] WARNING: src/c_api/c_api.cc:935: `ntree_limit` is deprecated, use `iteration_range` instead.
## [16:02:44] WARNING: src/c_api/c_api.cc:935: `ntree_limit` is deprecated, use `iteration_range` instead.
## [16:02:44] WARNING: src/c_api/c_api.cc:935: `ntree_limit` is deprecated, use `iteration_range` instead.
## [16:02:44] WARNING: src/c_api/c_api.cc:935: `ntree_limit` is deprecated, use `iteration_range` instead.
## [16:02:44] WARNING: src/c_api/c_api.cc:935: `ntree_limit` is deprecated, use `iteration_range` instead.
## [16:02:44] WARNING: src/c_api/c_api.cc:935: `ntree_limit` is deprecated, use `iteration_range` instead.
## [16:02:44] WARNING: src/c_api/c_api.cc:935: `ntree_limit` is deprecated, use `iteration_range` instead.
## [16:02:44] WARNING: src/c_api/c_api.cc:935: `ntree_limit` is deprecated, use `iteration_range` instead.
## [16:02:44] WARNING: src/c_api/c_api.cc:935: `ntree_limit` is deprecated, use `iteration_range` instead.
## [16:02:45] WARNING: src/c_api/c_api.cc:935: `ntree_limit` is deprecated, use `iteration_range` instead.
## [16:02:45] WARNING: src/c_api/c_api.cc:935: `ntree_limit` is deprecated, use `iteration_range` instead.
## [16:02:45] WARNING: src/c_api/c_api.cc:935: `ntree_limit` is deprecated, use `iteration_range` instead.
## [16:02:45] WARNING: src/c_api/c_api.cc:935: `ntree_limit` is deprecated, use `iteration_range` instead.
## [16:02:45] WARNING: src/c_api/c_api.cc:935: `ntree_limit` is deprecated, use `iteration_range` instead.
## [16:02:45] WARNING: src/c_api/c_api.cc:935: `ntree_limit` is deprecated, use `iteration_range` instead.
## [16:02:45] WARNING: src/c_api/c_api.cc:935: `ntree_limit` is deprecated, use `iteration_range` instead.
## [16:02:45] WARNING: src/c_api/c_api.cc:935: `ntree_limit` is deprecated, use `iteration_range` instead.
## [16:02:45] WARNING: src/c_api/c_api.cc:935: `ntree_limit` is deprecated, use `iteration_range` instead.
## [16:02:45] WARNING: src/c_api/c_api.cc:935: `ntree_limit` is deprecated, use `iteration_range` instead.
## [16:02:46] WARNING: src/c_api/c_api.cc:935: `ntree_limit` is deprecated, use `iteration_range` instead.
## [16:02:46] WARNING: src/c_api/c_api.cc:935: `ntree_limit` is deprecated, use `iteration_range` instead.
## [16:02:46] WARNING: src/c_api/c_api.cc:935: `ntree_limit` is deprecated, use `iteration_range` instead.
## [16:02:46] WARNING: src/c_api/c_api.cc:935: `ntree_limit` is deprecated, use `iteration_range` instead.
## [16:02:46] WARNING: src/c_api/c_api.cc:935: `ntree_limit` is deprecated, use `iteration_range` instead.
## [16:02:46] WARNING: src/c_api/c_api.cc:935: `ntree_limit` is deprecated, use `iteration_range` instead.
## [16:02:46] WARNING: src/c_api/c_api.cc:935: `ntree_limit` is deprecated, use `iteration_range` instead.
## [16:02:46] WARNING: src/c_api/c_api.cc:935: `ntree_limit` is deprecated, use `iteration_range` instead.
## [16:02:46] WARNING: src/c_api/c_api.cc:935: `ntree_limit` is deprecated, use `iteration_range` instead.
## [16:02:46] WARNING: src/c_api/c_api.cc:935: `ntree_limit` is deprecated, use `iteration_range` instead.
## [16:02:47] WARNING: src/c_api/c_api.cc:935: `ntree_limit` is deprecated, use `iteration_range` instead.
## [16:02:47] WARNING: src/c_api/c_api.cc:935: `ntree_limit` is deprecated, use `iteration_range` instead.
## [16:02:47] WARNING: src/c_api/c_api.cc:935: `ntree_limit` is deprecated, use `iteration_range` instead.
## [16:02:47] WARNING: src/c_api/c_api.cc:935: `ntree_limit` is deprecated, use `iteration_range` instead.
## [16:02:47] WARNING: src/c_api/c_api.cc:935: `ntree_limit` is deprecated, use `iteration_range` instead.
## [16:02:47] WARNING: src/c_api/c_api.cc:935: `ntree_limit` is deprecated, use `iteration_range` instead.
## [16:02:47] WARNING: src/c_api/c_api.cc:935: `ntree_limit` is deprecated, use `iteration_range` instead.
## [16:02:47] WARNING: src/c_api/c_api.cc:935: `ntree_limit` is deprecated, use `iteration_range` instead.
## [16:02:47] WARNING: src/c_api/c_api.cc:935: `ntree_limit` is deprecated, use `iteration_range` instead.
## [16:02:47] WARNING: src/c_api/c_api.cc:935: `ntree_limit` is deprecated, use `iteration_range` instead.
## [16:02:48] WARNING: src/c_api/c_api.cc:935: `ntree_limit` is deprecated, use `iteration_range` instead.
## [16:02:48] WARNING: src/c_api/c_api.cc:935: `ntree_limit` is deprecated, use `iteration_range` instead.
## [16:02:48] WARNING: src/c_api/c_api.cc:935: `ntree_limit` is deprecated, use `iteration_range` instead.
## [16:02:48] WARNING: src/c_api/c_api.cc:935: `ntree_limit` is deprecated, use `iteration_range` instead.
## [16:02:48] WARNING: src/c_api/c_api.cc:935: `ntree_limit` is deprecated, use `iteration_range` instead.
## [16:02:48] WARNING: src/c_api/c_api.cc:935: `ntree_limit` is deprecated, use `iteration_range` instead.
## [16:02:48] WARNING: src/c_api/c_api.cc:935: `ntree_limit` is deprecated, use `iteration_range` instead.
## [16:02:48] WARNING: src/c_api/c_api.cc:935: `ntree_limit` is deprecated, use `iteration_range` instead.
## [16:02:48] WARNING: src/c_api/c_api.cc:935: `ntree_limit` is deprecated, use `iteration_range` instead.
## [16:02:48] WARNING: src/c_api/c_api.cc:935: `ntree_limit` is deprecated, use `iteration_range` instead.
## [16:02:48] WARNING: src/c_api/c_api.cc:935: `ntree_limit` is deprecated, use `iteration_range` instead.
## [16:02:48] WARNING: src/c_api/c_api.cc:935: `ntree_limit` is deprecated, use `iteration_range` instead.
## [16:02:48] WARNING: src/c_api/c_api.cc:935: `ntree_limit` is deprecated, use `iteration_range` instead.
## [16:02:48] WARNING: src/c_api/c_api.cc:935: `ntree_limit` is deprecated, use `iteration_range` instead.
## [16:02:49] WARNING: src/c_api/c_api.cc:935: `ntree_limit` is deprecated, use `iteration_range` instead.
## [16:02:49] WARNING: src/c_api/c_api.cc:935: `ntree_limit` is deprecated, use `iteration_range` instead.
## [16:02:49] WARNING: src/c_api/c_api.cc:935: `ntree_limit` is deprecated, use `iteration_range` instead.
## [16:02:49] WARNING: src/c_api/c_api.cc:935: `ntree_limit` is deprecated, use `iteration_range` instead.
## [16:02:49] WARNING: src/c_api/c_api.cc:935: `ntree_limit` is deprecated, use `iteration_range` instead.
## [16:02:49] WARNING: src/c_api/c_api.cc:935: `ntree_limit` is deprecated, use `iteration_range` instead.
## [16:02:49] WARNING: src/c_api/c_api.cc:935: `ntree_limit` is deprecated, use `iteration_range` instead.
## [16:02:49] WARNING: src/c_api/c_api.cc:935: `ntree_limit` is deprecated, use `iteration_range` instead.
## [16:02:49] WARNING: src/c_api/c_api.cc:935: `ntree_limit` is deprecated, use `iteration_range` instead.
## [16:02:49] WARNING: src/c_api/c_api.cc:935: `ntree_limit` is deprecated, use `iteration_range` instead.
## [16:02:50] WARNING: src/c_api/c_api.cc:935: `ntree_limit` is deprecated, use `iteration_range` instead.
## [16:02:50] WARNING: src/c_api/c_api.cc:935: `ntree_limit` is deprecated, use `iteration_range` instead.
## [16:02:50] WARNING: src/c_api/c_api.cc:935: `ntree_limit` is deprecated, use `iteration_range` instead.
## [16:02:50] WARNING: src/c_api/c_api.cc:935: `ntree_limit` is deprecated, use `iteration_range` instead.
## [16:02:50] WARNING: src/c_api/c_api.cc:935: `ntree_limit` is deprecated, use `iteration_range` instead.
## [16:02:50] WARNING: src/c_api/c_api.cc:935: `ntree_limit` is deprecated, use `iteration_range` instead.
## [16:02:50] WARNING: src/c_api/c_api.cc:935: `ntree_limit` is deprecated, use `iteration_range` instead.
## [16:02:50] WARNING: src/c_api/c_api.cc:935: `ntree_limit` is deprecated, use `iteration_range` instead.
## [16:02:53] WARNING: src/c_api/c_api.cc:935: `ntree_limit` is deprecated, use `iteration_range` instead.
## [16:02:53] WARNING: src/c_api/c_api.cc:935: `ntree_limit` is deprecated, use `iteration_range` instead.
## [16:02:53] WARNING: src/c_api/c_api.cc:935: `ntree_limit` is deprecated, use `iteration_range` instead.
## [16:02:53] WARNING: src/c_api/c_api.cc:935: `ntree_limit` is deprecated, use `iteration_range` instead.
## [16:02:54] WARNING: src/c_api/c_api.cc:935: `ntree_limit` is deprecated, use `iteration_range` instead.
## [16:02:54] WARNING: src/c_api/c_api.cc:935: `ntree_limit` is deprecated, use `iteration_range` instead.
## [16:02:54] WARNING: src/c_api/c_api.cc:935: `ntree_limit` is deprecated, use `iteration_range` instead.
## [16:02:54] WARNING: src/c_api/c_api.cc:935: `ntree_limit` is deprecated, use `iteration_range` instead.
## [16:02:54] WARNING: src/c_api/c_api.cc:935: `ntree_limit` is deprecated, use `iteration_range` instead.
## [16:02:54] WARNING: src/c_api/c_api.cc:935: `ntree_limit` is deprecated, use `iteration_range` instead.
## [16:02:54] WARNING: src/c_api/c_api.cc:935: `ntree_limit` is deprecated, use `iteration_range` instead.
## [16:02:54] WARNING: src/c_api/c_api.cc:935: `ntree_limit` is deprecated, use `iteration_range` instead.
## [16:02:54] WARNING: src/c_api/c_api.cc:935: `ntree_limit` is deprecated, use `iteration_range` instead.
## [16:02:54] WARNING: src/c_api/c_api.cc:935: `ntree_limit` is deprecated, use `iteration_range` instead.
## [16:02:54] WARNING: src/c_api/c_api.cc:935: `ntree_limit` is deprecated, use `iteration_range` instead.
## [16:02:54] WARNING: src/c_api/c_api.cc:935: `ntree_limit` is deprecated, use `iteration_range` instead.
## [16:02:55] WARNING: src/c_api/c_api.cc:935: `ntree_limit` is deprecated, use `iteration_range` instead.
## [16:02:55] WARNING: src/c_api/c_api.cc:935: `ntree_limit` is deprecated, use `iteration_range` instead.
## [16:02:55] WARNING: src/c_api/c_api.cc:935: `ntree_limit` is deprecated, use `iteration_range` instead.
## [16:02:55] WARNING: src/c_api/c_api.cc:935: `ntree_limit` is deprecated, use `iteration_range` instead.
## [16:02:55] WARNING: src/c_api/c_api.cc:935: `ntree_limit` is deprecated, use `iteration_range` instead.
## [16:02:55] WARNING: src/c_api/c_api.cc:935: `ntree_limit` is deprecated, use `iteration_range` instead.
## [16:02:55] WARNING: src/c_api/c_api.cc:935: `ntree_limit` is deprecated, use `iteration_range` instead.
## [16:02:55] WARNING: src/c_api/c_api.cc:935: `ntree_limit` is deprecated, use `iteration_range` instead.
## [16:02:55] WARNING: src/c_api/c_api.cc:935: `ntree_limit` is deprecated, use `iteration_range` instead.
## [16:02:55] WARNING: src/c_api/c_api.cc:935: `ntree_limit` is deprecated, use `iteration_range` instead.
## [16:02:56] WARNING: src/c_api/c_api.cc:935: `ntree_limit` is deprecated, use `iteration_range` instead.
## [16:02:56] WARNING: src/c_api/c_api.cc:935: `ntree_limit` is deprecated, use `iteration_range` instead.
## [16:02:56] WARNING: src/c_api/c_api.cc:935: `ntree_limit` is deprecated, use `iteration_range` instead.
## [16:02:56] WARNING: src/c_api/c_api.cc:935: `ntree_limit` is deprecated, use `iteration_range` instead.
## [16:02:56] WARNING: src/c_api/c_api.cc:935: `ntree_limit` is deprecated, use `iteration_range` instead.
## [16:02:56] WARNING: src/c_api/c_api.cc:935: `ntree_limit` is deprecated, use `iteration_range` instead.
## [16:02:56] WARNING: src/c_api/c_api.cc:935: `ntree_limit` is deprecated, use `iteration_range` instead.
## [16:02:56] WARNING: src/c_api/c_api.cc:935: `ntree_limit` is deprecated, use `iteration_range` instead.
## [16:02:57] WARNING: src/c_api/c_api.cc:935: `ntree_limit` is deprecated, use `iteration_range` instead.
## [16:02:57] WARNING: src/c_api/c_api.cc:935: `ntree_limit` is deprecated, use `iteration_range` instead.
## [16:02:57] WARNING: src/c_api/c_api.cc:935: `ntree_limit` is deprecated, use `iteration_range` instead.
## [16:02:57] WARNING: src/c_api/c_api.cc:935: `ntree_limit` is deprecated, use `iteration_range` instead.
## [16:02:57] WARNING: src/c_api/c_api.cc:935: `ntree_limit` is deprecated, use `iteration_range` instead.
## [16:02:57] WARNING: src/c_api/c_api.cc:935: `ntree_limit` is deprecated, use `iteration_range` instead.
## [16:02:57] WARNING: src/c_api/c_api.cc:935: `ntree_limit` is deprecated, use `iteration_range` instead.
## [16:02:57] WARNING: src/c_api/c_api.cc:935: `ntree_limit` is deprecated, use `iteration_range` instead.
## [16:02:57] WARNING: src/c_api/c_api.cc:935: `ntree_limit` is deprecated, use `iteration_range` instead.
## [16:02:57] WARNING: src/c_api/c_api.cc:935: `ntree_limit` is deprecated, use `iteration_range` instead.
## [16:02:57] WARNING: src/c_api/c_api.cc:935: `ntree_limit` is deprecated, use `iteration_range` instead.
## [16:02:57] WARNING: src/c_api/c_api.cc:935: `ntree_limit` is deprecated, use `iteration_range` instead.
## [16:02:57] WARNING: src/c_api/c_api.cc:935: `ntree_limit` is deprecated, use `iteration_range` instead.
## [16:02:57] WARNING: src/c_api/c_api.cc:935: `ntree_limit` is deprecated, use `iteration_range` instead.
## [16:02:58] WARNING: src/c_api/c_api.cc:935: `ntree_limit` is deprecated, use `iteration_range` instead.
## [16:02:58] WARNING: src/c_api/c_api.cc:935: `ntree_limit` is deprecated, use `iteration_range` instead.
## [16:02:58] WARNING: src/c_api/c_api.cc:935: `ntree_limit` is deprecated, use `iteration_range` instead.
## [16:02:58] WARNING: src/c_api/c_api.cc:935: `ntree_limit` is deprecated, use `iteration_range` instead.
## [16:02:58] WARNING: src/c_api/c_api.cc:935: `ntree_limit` is deprecated, use `iteration_range` instead.
## [16:02:58] WARNING: src/c_api/c_api.cc:935: `ntree_limit` is deprecated, use `iteration_range` instead.
## [16:02:58] WARNING: src/c_api/c_api.cc:935: `ntree_limit` is deprecated, use `iteration_range` instead.
## [16:02:58] WARNING: src/c_api/c_api.cc:935: `ntree_limit` is deprecated, use `iteration_range` instead.
## [16:02:58] WARNING: src/c_api/c_api.cc:935: `ntree_limit` is deprecated, use `iteration_range` instead.
## [16:02:58] WARNING: src/c_api/c_api.cc:935: `ntree_limit` is deprecated, use `iteration_range` instead.
## [16:02:58] WARNING: src/c_api/c_api.cc:935: `ntree_limit` is deprecated, use `iteration_range` instead.
## [16:02:58] WARNING: src/c_api/c_api.cc:935: `ntree_limit` is deprecated, use `iteration_range` instead.
## [16:02:59] WARNING: src/c_api/c_api.cc:935: `ntree_limit` is deprecated, use `iteration_range` instead.
## [16:02:59] WARNING: src/c_api/c_api.cc:935: `ntree_limit` is deprecated, use `iteration_range` instead.
## [16:02:59] WARNING: src/c_api/c_api.cc:935: `ntree_limit` is deprecated, use `iteration_range` instead.
## [16:02:59] WARNING: src/c_api/c_api.cc:935: `ntree_limit` is deprecated, use `iteration_range` instead.
## [16:02:59] WARNING: src/c_api/c_api.cc:935: `ntree_limit` is deprecated, use `iteration_range` instead.
## [16:02:59] WARNING: src/c_api/c_api.cc:935: `ntree_limit` is deprecated, use `iteration_range` instead.
## [16:02:59] WARNING: src/c_api/c_api.cc:935: `ntree_limit` is deprecated, use `iteration_range` instead.
## [16:02:59] WARNING: src/c_api/c_api.cc:935: `ntree_limit` is deprecated, use `iteration_range` instead.
## [16:03:00] WARNING: src/c_api/c_api.cc:935: `ntree_limit` is deprecated, use `iteration_range` instead.
## [16:03:00] WARNING: src/c_api/c_api.cc:935: `ntree_limit` is deprecated, use `iteration_range` instead.
## [16:03:00] WARNING: src/c_api/c_api.cc:935: `ntree_limit` is deprecated, use `iteration_range` instead.
## [16:03:00] WARNING: src/c_api/c_api.cc:935: `ntree_limit` is deprecated, use `iteration_range` instead.
## [16:03:00] WARNING: src/c_api/c_api.cc:935: `ntree_limit` is deprecated, use `iteration_range` instead.
## [16:03:00] WARNING: src/c_api/c_api.cc:935: `ntree_limit` is deprecated, use `iteration_range` instead.
## [16:03:00] WARNING: src/c_api/c_api.cc:935: `ntree_limit` is deprecated, use `iteration_range` instead.
## [16:03:00] WARNING: src/c_api/c_api.cc:935: `ntree_limit` is deprecated, use `iteration_range` instead.
## [16:03:00] WARNING: src/c_api/c_api.cc:935: `ntree_limit` is deprecated, use `iteration_range` instead.
## [16:03:00] WARNING: src/c_api/c_api.cc:935: `ntree_limit` is deprecated, use `iteration_range` instead.
## [16:03:00] WARNING: src/c_api/c_api.cc:935: `ntree_limit` is deprecated, use `iteration_range` instead.
## [16:03:00] WARNING: src/c_api/c_api.cc:935: `ntree_limit` is deprecated, use `iteration_range` instead.
## [16:03:00] WARNING: src/c_api/c_api.cc:935: `ntree_limit` is deprecated, use `iteration_range` instead.
## [16:03:00] WARNING: src/c_api/c_api.cc:935: `ntree_limit` is deprecated, use `iteration_range` instead.
## [16:03:01] WARNING: src/c_api/c_api.cc:935: `ntree_limit` is deprecated, use `iteration_range` instead.
## [16:03:01] WARNING: src/c_api/c_api.cc:935: `ntree_limit` is deprecated, use `iteration_range` instead.
## [16:03:01] WARNING: src/c_api/c_api.cc:935: `ntree_limit` is deprecated, use `iteration_range` instead.
## [16:03:01] WARNING: src/c_api/c_api.cc:935: `ntree_limit` is deprecated, use `iteration_range` instead.
## [16:03:01] WARNING: src/c_api/c_api.cc:935: `ntree_limit` is deprecated, use `iteration_range` instead.
## [16:03:01] WARNING: src/c_api/c_api.cc:935: `ntree_limit` is deprecated, use `iteration_range` instead.
## [16:03:01] WARNING: src/c_api/c_api.cc:935: `ntree_limit` is deprecated, use `iteration_range` instead.
## [16:03:01] WARNING: src/c_api/c_api.cc:935: `ntree_limit` is deprecated, use `iteration_range` instead.
## [16:03:01] WARNING: src/c_api/c_api.cc:935: `ntree_limit` is deprecated, use `iteration_range` instead.
## [16:03:01] WARNING: src/c_api/c_api.cc:935: `ntree_limit` is deprecated, use `iteration_range` instead.
## [16:03:02] WARNING: src/c_api/c_api.cc:935: `ntree_limit` is deprecated, use `iteration_range` instead.
## [16:03:02] WARNING: src/c_api/c_api.cc:935: `ntree_limit` is deprecated, use `iteration_range` instead.
## [16:03:02] WARNING: src/c_api/c_api.cc:935: `ntree_limit` is deprecated, use `iteration_range` instead.
## [16:03:02] WARNING: src/c_api/c_api.cc:935: `ntree_limit` is deprecated, use `iteration_range` instead.
## [16:03:02] WARNING: src/c_api/c_api.cc:935: `ntree_limit` is deprecated, use `iteration_range` instead.
## [16:03:02] WARNING: src/c_api/c_api.cc:935: `ntree_limit` is deprecated, use `iteration_range` instead.
## [16:03:02] WARNING: src/c_api/c_api.cc:935: `ntree_limit` is deprecated, use `iteration_range` instead.
## [16:03:02] WARNING: src/c_api/c_api.cc:935: `ntree_limit` is deprecated, use `iteration_range` instead.
## [16:03:02] WARNING: src/c_api/c_api.cc:935: `ntree_limit` is deprecated, use `iteration_range` instead.
## [16:03:02] WARNING: src/c_api/c_api.cc:935: `ntree_limit` is deprecated, use `iteration_range` instead.
## [16:03:03] WARNING: src/c_api/c_api.cc:935: `ntree_limit` is deprecated, use `iteration_range` instead.
## [16:03:03] WARNING: src/c_api/c_api.cc:935: `ntree_limit` is deprecated, use `iteration_range` instead.
## [16:03:03] WARNING: src/c_api/c_api.cc:935: `ntree_limit` is deprecated, use `iteration_range` instead.
## [16:03:03] WARNING: src/c_api/c_api.cc:935: `ntree_limit` is deprecated, use `iteration_range` instead.
## [16:03:03] WARNING: src/c_api/c_api.cc:935: `ntree_limit` is deprecated, use `iteration_range` instead.
## [16:03:03] WARNING: src/c_api/c_api.cc:935: `ntree_limit` is deprecated, use `iteration_range` instead.
## [16:03:03] WARNING: src/c_api/c_api.cc:935: `ntree_limit` is deprecated, use `iteration_range` instead.
## [16:03:03] WARNING: src/c_api/c_api.cc:935: `ntree_limit` is deprecated, use `iteration_range` instead.
## [16:03:03] WARNING: src/c_api/c_api.cc:935: `ntree_limit` is deprecated, use `iteration_range` instead.
## [16:03:03] WARNING: src/c_api/c_api.cc:935: `ntree_limit` is deprecated, use `iteration_range` instead.
## [16:03:04] WARNING: src/c_api/c_api.cc:935: `ntree_limit` is deprecated, use `iteration_range` instead.
## [16:03:04] WARNING: src/c_api/c_api.cc:935: `ntree_limit` is deprecated, use `iteration_range` instead.
## [16:03:04] WARNING: src/c_api/c_api.cc:935: `ntree_limit` is deprecated, use `iteration_range` instead.
## [16:03:04] WARNING: src/c_api/c_api.cc:935: `ntree_limit` is deprecated, use `iteration_range` instead.
## [16:03:04] WARNING: src/c_api/c_api.cc:935: `ntree_limit` is deprecated, use `iteration_range` instead.
## [16:03:04] WARNING: src/c_api/c_api.cc:935: `ntree_limit` is deprecated, use `iteration_range` instead.
## [16:03:04] WARNING: src/c_api/c_api.cc:935: `ntree_limit` is deprecated, use `iteration_range` instead.
## [16:03:04] WARNING: src/c_api/c_api.cc:935: `ntree_limit` is deprecated, use `iteration_range` instead.
## [16:03:04] WARNING: src/c_api/c_api.cc:935: `ntree_limit` is deprecated, use `iteration_range` instead.
## [16:03:04] WARNING: src/c_api/c_api.cc:935: `ntree_limit` is deprecated, use `iteration_range` instead.
## [16:03:04] WARNING: src/c_api/c_api.cc:935: `ntree_limit` is deprecated, use `iteration_range` instead.
## [16:03:04] WARNING: src/c_api/c_api.cc:935: `ntree_limit` is deprecated, use `iteration_range` instead.
## [16:03:05] WARNING: src/c_api/c_api.cc:935: `ntree_limit` is deprecated, use `iteration_range` instead.
## [16:03:05] WARNING: src/c_api/c_api.cc:935: `ntree_limit` is deprecated, use `iteration_range` instead.
## [16:03:05] WARNING: src/c_api/c_api.cc:935: `ntree_limit` is deprecated, use `iteration_range` instead.
## [16:03:05] WARNING: src/c_api/c_api.cc:935: `ntree_limit` is deprecated, use `iteration_range` instead.
## [16:03:05] WARNING: src/c_api/c_api.cc:935: `ntree_limit` is deprecated, use `iteration_range` instead.
## [16:03:05] WARNING: src/c_api/c_api.cc:935: `ntree_limit` is deprecated, use `iteration_range` instead.
## [16:03:05] WARNING: src/c_api/c_api.cc:935: `ntree_limit` is deprecated, use `iteration_range` instead.
## [16:03:05] WARNING: src/c_api/c_api.cc:935: `ntree_limit` is deprecated, use `iteration_range` instead.
## [16:03:05] WARNING: src/c_api/c_api.cc:935: `ntree_limit` is deprecated, use `iteration_range` instead.
## [16:03:05] WARNING: src/c_api/c_api.cc:935: `ntree_limit` is deprecated, use `iteration_range` instead.
## [16:03:06] WARNING: src/c_api/c_api.cc:935: `ntree_limit` is deprecated, use `iteration_range` instead.
## [16:03:06] WARNING: src/c_api/c_api.cc:935: `ntree_limit` is deprecated, use `iteration_range` instead.
## [16:03:06] WARNING: src/c_api/c_api.cc:935: `ntree_limit` is deprecated, use `iteration_range` instead.
## [16:03:06] WARNING: src/c_api/c_api.cc:935: `ntree_limit` is deprecated, use `iteration_range` instead.
## [16:03:06] WARNING: src/c_api/c_api.cc:935: `ntree_limit` is deprecated, use `iteration_range` instead.
## [16:03:06] WARNING: src/c_api/c_api.cc:935: `ntree_limit` is deprecated, use `iteration_range` instead.
## [16:03:06] WARNING: src/c_api/c_api.cc:935: `ntree_limit` is deprecated, use `iteration_range` instead.
## [16:03:06] WARNING: src/c_api/c_api.cc:935: `ntree_limit` is deprecated, use `iteration_range` instead.
## [16:03:06] WARNING: src/c_api/c_api.cc:935: `ntree_limit` is deprecated, use `iteration_range` instead.
## [16:03:06] WARNING: src/c_api/c_api.cc:935: `ntree_limit` is deprecated, use `iteration_range` instead.
## [16:03:06] WARNING: src/c_api/c_api.cc:935: `ntree_limit` is deprecated, use `iteration_range` instead.
## [16:03:06] WARNING: src/c_api/c_api.cc:935: `ntree_limit` is deprecated, use `iteration_range` instead.
## [16:03:07] WARNING: src/c_api/c_api.cc:935: `ntree_limit` is deprecated, use `iteration_range` instead.
## [16:03:07] WARNING: src/c_api/c_api.cc:935: `ntree_limit` is deprecated, use `iteration_range` instead.
## [16:03:07] WARNING: src/c_api/c_api.cc:935: `ntree_limit` is deprecated, use `iteration_range` instead.
## [16:03:07] WARNING: src/c_api/c_api.cc:935: `ntree_limit` is deprecated, use `iteration_range` instead.
## [16:03:07] WARNING: src/c_api/c_api.cc:935: `ntree_limit` is deprecated, use `iteration_range` instead.
## [16:03:07] WARNING: src/c_api/c_api.cc:935: `ntree_limit` is deprecated, use `iteration_range` instead.
## [16:03:07] WARNING: src/c_api/c_api.cc:935: `ntree_limit` is deprecated, use `iteration_range` instead.
## [16:03:07] WARNING: src/c_api/c_api.cc:935: `ntree_limit` is deprecated, use `iteration_range` instead.
## [16:03:07] WARNING: src/c_api/c_api.cc:935: `ntree_limit` is deprecated, use `iteration_range` instead.
## [16:03:07] WARNING: src/c_api/c_api.cc:935: `ntree_limit` is deprecated, use `iteration_range` instead.
## [16:03:07] WARNING: src/c_api/c_api.cc:935: `ntree_limit` is deprecated, use `iteration_range` instead.
## [16:03:07] WARNING: src/c_api/c_api.cc:935: `ntree_limit` is deprecated, use `iteration_range` instead.
## [16:03:08] WARNING: src/c_api/c_api.cc:935: `ntree_limit` is deprecated, use `iteration_range` instead.
## [16:03:08] WARNING: src/c_api/c_api.cc:935: `ntree_limit` is deprecated, use `iteration_range` instead.
## [16:03:08] WARNING: src/c_api/c_api.cc:935: `ntree_limit` is deprecated, use `iteration_range` instead.
## [16:03:08] WARNING: src/c_api/c_api.cc:935: `ntree_limit` is deprecated, use `iteration_range` instead.
## [16:03:08] WARNING: src/c_api/c_api.cc:935: `ntree_limit` is deprecated, use `iteration_range` instead.
## [16:03:08] WARNING: src/c_api/c_api.cc:935: `ntree_limit` is deprecated, use `iteration_range` instead.
## [16:03:08] WARNING: src/c_api/c_api.cc:935: `ntree_limit` is deprecated, use `iteration_range` instead.
## [16:03:08] WARNING: src/c_api/c_api.cc:935: `ntree_limit` is deprecated, use `iteration_range` instead.
## [16:03:08] WARNING: src/c_api/c_api.cc:935: `ntree_limit` is deprecated, use `iteration_range` instead.
## [16:03:08] WARNING: src/c_api/c_api.cc:935: `ntree_limit` is deprecated, use `iteration_range` instead.
## [16:03:09] WARNING: src/c_api/c_api.cc:935: `ntree_limit` is deprecated, use `iteration_range` instead.
## [16:03:09] WARNING: src/c_api/c_api.cc:935: `ntree_limit` is deprecated, use `iteration_range` instead.
## [16:03:09] WARNING: src/c_api/c_api.cc:935: `ntree_limit` is deprecated, use `iteration_range` instead.
## [16:03:09] WARNING: src/c_api/c_api.cc:935: `ntree_limit` is deprecated, use `iteration_range` instead.
## [16:03:09] WARNING: src/c_api/c_api.cc:935: `ntree_limit` is deprecated, use `iteration_range` instead.
## [16:03:09] WARNING: src/c_api/c_api.cc:935: `ntree_limit` is deprecated, use `iteration_range` instead.
## [16:03:09] WARNING: src/c_api/c_api.cc:935: `ntree_limit` is deprecated, use `iteration_range` instead.
## [16:03:09] WARNING: src/c_api/c_api.cc:935: `ntree_limit` is deprecated, use `iteration_range` instead.
## [16:03:10] WARNING: src/c_api/c_api.cc:935: `ntree_limit` is deprecated, use `iteration_range` instead.
## [16:03:10] WARNING: src/c_api/c_api.cc:935: `ntree_limit` is deprecated, use `iteration_range` instead.
## [16:03:10] WARNING: src/c_api/c_api.cc:935: `ntree_limit` is deprecated, use `iteration_range` instead.
## [16:03:10] WARNING: src/c_api/c_api.cc:935: `ntree_limit` is deprecated, use `iteration_range` instead.
## [16:03:10] WARNING: src/c_api/c_api.cc:935: `ntree_limit` is deprecated, use `iteration_range` instead.
## [16:03:10] WARNING: src/c_api/c_api.cc:935: `ntree_limit` is deprecated, use `iteration_range` instead.
## [16:03:10] WARNING: src/c_api/c_api.cc:935: `ntree_limit` is deprecated, use `iteration_range` instead.
## [16:03:10] WARNING: src/c_api/c_api.cc:935: `ntree_limit` is deprecated, use `iteration_range` instead.
## [16:03:10] WARNING: src/c_api/c_api.cc:935: `ntree_limit` is deprecated, use `iteration_range` instead.
## [16:03:10] WARNING: src/c_api/c_api.cc:935: `ntree_limit` is deprecated, use `iteration_range` instead.
## [16:03:10] WARNING: src/c_api/c_api.cc:935: `ntree_limit` is deprecated, use `iteration_range` instead.
## [16:03:10] WARNING: src/c_api/c_api.cc:935: `ntree_limit` is deprecated, use `iteration_range` instead.
## [16:03:10] WARNING: src/c_api/c_api.cc:935: `ntree_limit` is deprecated, use `iteration_range` instead.
## [16:03:10] WARNING: src/c_api/c_api.cc:935: `ntree_limit` is deprecated, use `iteration_range` instead.
## [16:03:11] WARNING: src/c_api/c_api.cc:935: `ntree_limit` is deprecated, use `iteration_range` instead.
## [16:03:11] WARNING: src/c_api/c_api.cc:935: `ntree_limit` is deprecated, use `iteration_range` instead.
## [16:03:11] WARNING: src/c_api/c_api.cc:935: `ntree_limit` is deprecated, use `iteration_range` instead.
## [16:03:11] WARNING: src/c_api/c_api.cc:935: `ntree_limit` is deprecated, use `iteration_range` instead.
## [16:03:11] WARNING: src/c_api/c_api.cc:935: `ntree_limit` is deprecated, use `iteration_range` instead.
## [16:03:11] WARNING: src/c_api/c_api.cc:935: `ntree_limit` is deprecated, use `iteration_range` instead.
## [16:03:11] WARNING: src/c_api/c_api.cc:935: `ntree_limit` is deprecated, use `iteration_range` instead.
## [16:03:11] WARNING: src/c_api/c_api.cc:935: `ntree_limit` is deprecated, use `iteration_range` instead.
## [16:03:11] WARNING: src/c_api/c_api.cc:935: `ntree_limit` is deprecated, use `iteration_range` instead.
## [16:03:11] WARNING: src/c_api/c_api.cc:935: `ntree_limit` is deprecated, use `iteration_range` instead.
## [16:03:12] WARNING: src/c_api/c_api.cc:935: `ntree_limit` is deprecated, use `iteration_range` instead.
## [16:03:12] WARNING: src/c_api/c_api.cc:935: `ntree_limit` is deprecated, use `iteration_range` instead.
## [16:03:12] WARNING: src/c_api/c_api.cc:935: `ntree_limit` is deprecated, use `iteration_range` instead.
## [16:03:12] WARNING: src/c_api/c_api.cc:935: `ntree_limit` is deprecated, use `iteration_range` instead.
## [16:03:12] WARNING: src/c_api/c_api.cc:935: `ntree_limit` is deprecated, use `iteration_range` instead.
## [16:03:12] WARNING: src/c_api/c_api.cc:935: `ntree_limit` is deprecated, use `iteration_range` instead.
## [16:03:12] WARNING: src/c_api/c_api.cc:935: `ntree_limit` is deprecated, use `iteration_range` instead.
## [16:03:12] WARNING: src/c_api/c_api.cc:935: `ntree_limit` is deprecated, use `iteration_range` instead.
## [16:03:12] WARNING: src/c_api/c_api.cc:935: `ntree_limit` is deprecated, use `iteration_range` instead.
## [16:03:12] WARNING: src/c_api/c_api.cc:935: `ntree_limit` is deprecated, use `iteration_range` instead.
## [16:03:13] WARNING: src/c_api/c_api.cc:935: `ntree_limit` is deprecated, use `iteration_range` instead.
## [16:03:13] WARNING: src/c_api/c_api.cc:935: `ntree_limit` is deprecated, use `iteration_range` instead.
## [16:03:13] WARNING: src/c_api/c_api.cc:935: `ntree_limit` is deprecated, use `iteration_range` instead.
## [16:03:13] WARNING: src/c_api/c_api.cc:935: `ntree_limit` is deprecated, use `iteration_range` instead.
## [16:03:13] WARNING: src/c_api/c_api.cc:935: `ntree_limit` is deprecated, use `iteration_range` instead.
## [16:03:13] WARNING: src/c_api/c_api.cc:935: `ntree_limit` is deprecated, use `iteration_range` instead.
## [16:03:13] WARNING: src/c_api/c_api.cc:935: `ntree_limit` is deprecated, use `iteration_range` instead.
## [16:03:13] WARNING: src/c_api/c_api.cc:935: `ntree_limit` is deprecated, use `iteration_range` instead.
## [16:03:13] WARNING: src/c_api/c_api.cc:935: `ntree_limit` is deprecated, use `iteration_range` instead.
## [16:03:13] WARNING: src/c_api/c_api.cc:935: `ntree_limit` is deprecated, use `iteration_range` instead.
## [16:03:13] WARNING: src/c_api/c_api.cc:935: `ntree_limit` is deprecated, use `iteration_range` instead.
## [16:03:13] WARNING: src/c_api/c_api.cc:935: `ntree_limit` is deprecated, use `iteration_range` instead.
## [16:03:14] WARNING: src/c_api/c_api.cc:935: `ntree_limit` is deprecated, use `iteration_range` instead.
## [16:03:14] WARNING: src/c_api/c_api.cc:935: `ntree_limit` is deprecated, use `iteration_range` instead.
## [16:03:14] WARNING: src/c_api/c_api.cc:935: `ntree_limit` is deprecated, use `iteration_range` instead.
## [16:03:14] WARNING: src/c_api/c_api.cc:935: `ntree_limit` is deprecated, use `iteration_range` instead.
## [16:03:14] WARNING: src/c_api/c_api.cc:935: `ntree_limit` is deprecated, use `iteration_range` instead.
## [16:03:14] WARNING: src/c_api/c_api.cc:935: `ntree_limit` is deprecated, use `iteration_range` instead.
## [16:03:14] WARNING: src/c_api/c_api.cc:935: `ntree_limit` is deprecated, use `iteration_range` instead.
## [16:03:14] WARNING: src/c_api/c_api.cc:935: `ntree_limit` is deprecated, use `iteration_range` instead.
## [16:03:14] WARNING: src/c_api/c_api.cc:935: `ntree_limit` is deprecated, use `iteration_range` instead.
## [16:03:14] WARNING: src/c_api/c_api.cc:935: `ntree_limit` is deprecated, use `iteration_range` instead.
## [16:03:14] WARNING: src/c_api/c_api.cc:935: `ntree_limit` is deprecated, use `iteration_range` instead.
## [16:03:14] WARNING: src/c_api/c_api.cc:935: `ntree_limit` is deprecated, use `iteration_range` instead.
## [16:03:15] WARNING: src/c_api/c_api.cc:935: `ntree_limit` is deprecated, use `iteration_range` instead.
## [16:03:15] WARNING: src/c_api/c_api.cc:935: `ntree_limit` is deprecated, use `iteration_range` instead.
## [16:03:15] WARNING: src/c_api/c_api.cc:935: `ntree_limit` is deprecated, use `iteration_range` instead.
## [16:03:15] WARNING: src/c_api/c_api.cc:935: `ntree_limit` is deprecated, use `iteration_range` instead.
## [16:03:15] WARNING: src/c_api/c_api.cc:935: `ntree_limit` is deprecated, use `iteration_range` instead.
## [16:03:15] WARNING: src/c_api/c_api.cc:935: `ntree_limit` is deprecated, use `iteration_range` instead.
## [16:03:15] WARNING: src/c_api/c_api.cc:935: `ntree_limit` is deprecated, use `iteration_range` instead.
## [16:03:15] WARNING: src/c_api/c_api.cc:935: `ntree_limit` is deprecated, use `iteration_range` instead.
## [16:03:15] WARNING: src/c_api/c_api.cc:935: `ntree_limit` is deprecated, use `iteration_range` instead.
## [16:03:15] WARNING: src/c_api/c_api.cc:935: `ntree_limit` is deprecated, use `iteration_range` instead.
## [16:03:16] WARNING: src/c_api/c_api.cc:935: `ntree_limit` is deprecated, use `iteration_range` instead.
## [16:03:16] WARNING: src/c_api/c_api.cc:935: `ntree_limit` is deprecated, use `iteration_range` instead.
## [16:03:16] WARNING: src/c_api/c_api.cc:935: `ntree_limit` is deprecated, use `iteration_range` instead.
## [16:03:16] WARNING: src/c_api/c_api.cc:935: `ntree_limit` is deprecated, use `iteration_range` instead.
## [16:03:16] WARNING: src/c_api/c_api.cc:935: `ntree_limit` is deprecated, use `iteration_range` instead.
## [16:03:16] WARNING: src/c_api/c_api.cc:935: `ntree_limit` is deprecated, use `iteration_range` instead.
## [16:03:16] WARNING: src/c_api/c_api.cc:935: `ntree_limit` is deprecated, use `iteration_range` instead.
## [16:03:16] WARNING: src/c_api/c_api.cc:935: `ntree_limit` is deprecated, use `iteration_range` instead.
## [16:03:17] WARNING: src/c_api/c_api.cc:935: `ntree_limit` is deprecated, use `iteration_range` instead.
## [16:03:17] WARNING: src/c_api/c_api.cc:935: `ntree_limit` is deprecated, use `iteration_range` instead.
## [16:03:17] WARNING: src/c_api/c_api.cc:935: `ntree_limit` is deprecated, use `iteration_range` instead.
## [16:03:17] WARNING: src/c_api/c_api.cc:935: `ntree_limit` is deprecated, use `iteration_range` instead.
## [16:03:17] WARNING: src/c_api/c_api.cc:935: `ntree_limit` is deprecated, use `iteration_range` instead.
## [16:03:17] WARNING: src/c_api/c_api.cc:935: `ntree_limit` is deprecated, use `iteration_range` instead.
## [16:03:17] WARNING: src/c_api/c_api.cc:935: `ntree_limit` is deprecated, use `iteration_range` instead.
## [16:03:17] WARNING: src/c_api/c_api.cc:935: `ntree_limit` is deprecated, use `iteration_range` instead.
## [16:03:17] WARNING: src/c_api/c_api.cc:935: `ntree_limit` is deprecated, use `iteration_range` instead.
## [16:03:17] WARNING: src/c_api/c_api.cc:935: `ntree_limit` is deprecated, use `iteration_range` instead.
## [16:03:17] WARNING: src/c_api/c_api.cc:935: `ntree_limit` is deprecated, use `iteration_range` instead.
## [16:03:17] WARNING: src/c_api/c_api.cc:935: `ntree_limit` is deprecated, use `iteration_range` instead.
## [16:03:18] WARNING: src/c_api/c_api.cc:935: `ntree_limit` is deprecated, use `iteration_range` instead.
## [16:03:18] WARNING: src/c_api/c_api.cc:935: `ntree_limit` is deprecated, use `iteration_range` instead.
## [16:03:18] WARNING: src/c_api/c_api.cc:935: `ntree_limit` is deprecated, use `iteration_range` instead.
## [16:03:18] WARNING: src/c_api/c_api.cc:935: `ntree_limit` is deprecated, use `iteration_range` instead.
## [16:03:18] WARNING: src/c_api/c_api.cc:935: `ntree_limit` is deprecated, use `iteration_range` instead.
## [16:03:18] WARNING: src/c_api/c_api.cc:935: `ntree_limit` is deprecated, use `iteration_range` instead.
## [16:03:18] WARNING: src/c_api/c_api.cc:935: `ntree_limit` is deprecated, use `iteration_range` instead.
## [16:03:18] WARNING: src/c_api/c_api.cc:935: `ntree_limit` is deprecated, use `iteration_range` instead.
## [16:03:18] WARNING: src/c_api/c_api.cc:935: `ntree_limit` is deprecated, use `iteration_range` instead.
## [16:03:18] WARNING: src/c_api/c_api.cc:935: `ntree_limit` is deprecated, use `iteration_range` instead.
## [16:03:18] WARNING: src/c_api/c_api.cc:935: `ntree_limit` is deprecated, use `iteration_range` instead.
## [16:03:18] WARNING: src/c_api/c_api.cc:935: `ntree_limit` is deprecated, use `iteration_range` instead.
## [16:03:19] WARNING: src/c_api/c_api.cc:935: `ntree_limit` is deprecated, use `iteration_range` instead.
## [16:03:19] WARNING: src/c_api/c_api.cc:935: `ntree_limit` is deprecated, use `iteration_range` instead.
## [16:03:19] WARNING: src/c_api/c_api.cc:935: `ntree_limit` is deprecated, use `iteration_range` instead.
## [16:03:19] WARNING: src/c_api/c_api.cc:935: `ntree_limit` is deprecated, use `iteration_range` instead.
## [16:03:19] WARNING: src/c_api/c_api.cc:935: `ntree_limit` is deprecated, use `iteration_range` instead.
## [16:03:19] WARNING: src/c_api/c_api.cc:935: `ntree_limit` is deprecated, use `iteration_range` instead.
## [16:03:19] WARNING: src/c_api/c_api.cc:935: `ntree_limit` is deprecated, use `iteration_range` instead.
## [16:03:19] WARNING: src/c_api/c_api.cc:935: `ntree_limit` is deprecated, use `iteration_range` instead.
## [16:03:20] WARNING: src/c_api/c_api.cc:935: `ntree_limit` is deprecated, use `iteration_range` instead.
## [16:03:20] WARNING: src/c_api/c_api.cc:935: `ntree_limit` is deprecated, use `iteration_range` instead.
## [16:03:20] WARNING: src/c_api/c_api.cc:935: `ntree_limit` is deprecated, use `iteration_range` instead.
## [16:03:20] WARNING: src/c_api/c_api.cc:935: `ntree_limit` is deprecated, use `iteration_range` instead.
## [16:03:23] WARNING: src/c_api/c_api.cc:935: `ntree_limit` is deprecated, use `iteration_range` instead.
## [16:03:23] WARNING: src/c_api/c_api.cc:935: `ntree_limit` is deprecated, use `iteration_range` instead.
## [16:03:23] WARNING: src/c_api/c_api.cc:935: `ntree_limit` is deprecated, use `iteration_range` instead.
## [16:03:23] WARNING: src/c_api/c_api.cc:935: `ntree_limit` is deprecated, use `iteration_range` instead.
## [16:03:23] WARNING: src/c_api/c_api.cc:935: `ntree_limit` is deprecated, use `iteration_range` instead.
## [16:03:23] WARNING: src/c_api/c_api.cc:935: `ntree_limit` is deprecated, use `iteration_range` instead.
## [16:03:23] WARNING: src/c_api/c_api.cc:935: `ntree_limit` is deprecated, use `iteration_range` instead.
## [16:03:23] WARNING: src/c_api/c_api.cc:935: `ntree_limit` is deprecated, use `iteration_range` instead.
## [16:03:23] WARNING: src/c_api/c_api.cc:935: `ntree_limit` is deprecated, use `iteration_range` instead.
## [16:03:23] WARNING: src/c_api/c_api.cc:935: `ntree_limit` is deprecated, use `iteration_range` instead.
## [16:03:23] WARNING: src/c_api/c_api.cc:935: `ntree_limit` is deprecated, use `iteration_range` instead.
## [16:03:23] WARNING: src/c_api/c_api.cc:935: `ntree_limit` is deprecated, use `iteration_range` instead.
## [16:03:23] WARNING: src/c_api/c_api.cc:935: `ntree_limit` is deprecated, use `iteration_range` instead.
## [16:03:23] WARNING: src/c_api/c_api.cc:935: `ntree_limit` is deprecated, use `iteration_range` instead.
## [16:03:24] WARNING: src/c_api/c_api.cc:935: `ntree_limit` is deprecated, use `iteration_range` instead.
## [16:03:24] WARNING: src/c_api/c_api.cc:935: `ntree_limit` is deprecated, use `iteration_range` instead.
## [16:03:24] WARNING: src/c_api/c_api.cc:935: `ntree_limit` is deprecated, use `iteration_range` instead.
## [16:03:24] WARNING: src/c_api/c_api.cc:935: `ntree_limit` is deprecated, use `iteration_range` instead.
## [16:03:24] WARNING: src/c_api/c_api.cc:935: `ntree_limit` is deprecated, use `iteration_range` instead.
## [16:03:24] WARNING: src/c_api/c_api.cc:935: `ntree_limit` is deprecated, use `iteration_range` instead.
## [16:03:24] WARNING: src/c_api/c_api.cc:935: `ntree_limit` is deprecated, use `iteration_range` instead.
## [16:03:24] WARNING: src/c_api/c_api.cc:935: `ntree_limit` is deprecated, use `iteration_range` instead.
## [16:03:24] WARNING: src/c_api/c_api.cc:935: `ntree_limit` is deprecated, use `iteration_range` instead.
## [16:03:24] WARNING: src/c_api/c_api.cc:935: `ntree_limit` is deprecated, use `iteration_range` instead.
## [16:03:25] WARNING: src/c_api/c_api.cc:935: `ntree_limit` is deprecated, use `iteration_range` instead.
## [16:03:25] WARNING: src/c_api/c_api.cc:935: `ntree_limit` is deprecated, use `iteration_range` instead.
## [16:03:25] WARNING: src/c_api/c_api.cc:935: `ntree_limit` is deprecated, use `iteration_range` instead.
## [16:03:25] WARNING: src/c_api/c_api.cc:935: `ntree_limit` is deprecated, use `iteration_range` instead.
## [16:03:25] WARNING: src/c_api/c_api.cc:935: `ntree_limit` is deprecated, use `iteration_range` instead.
## [16:03:25] WARNING: src/c_api/c_api.cc:935: `ntree_limit` is deprecated, use `iteration_range` instead.
## [16:03:25] WARNING: src/c_api/c_api.cc:935: `ntree_limit` is deprecated, use `iteration_range` instead.
## [16:03:25] WARNING: src/c_api/c_api.cc:935: `ntree_limit` is deprecated, use `iteration_range` instead.
## [16:03:26] WARNING: src/c_api/c_api.cc:935: `ntree_limit` is deprecated, use `iteration_range` instead.
## [16:03:26] WARNING: src/c_api/c_api.cc:935: `ntree_limit` is deprecated, use `iteration_range` instead.
## [16:03:26] WARNING: src/c_api/c_api.cc:935: `ntree_limit` is deprecated, use `iteration_range` instead.
## [16:03:26] WARNING: src/c_api/c_api.cc:935: `ntree_limit` is deprecated, use `iteration_range` instead.
## [16:03:26] WARNING: src/c_api/c_api.cc:935: `ntree_limit` is deprecated, use `iteration_range` instead.
## [16:03:26] WARNING: src/c_api/c_api.cc:935: `ntree_limit` is deprecated, use `iteration_range` instead.
## [16:03:26] WARNING: src/c_api/c_api.cc:935: `ntree_limit` is deprecated, use `iteration_range` instead.
## [16:03:26] WARNING: src/c_api/c_api.cc:935: `ntree_limit` is deprecated, use `iteration_range` instead.
## [16:03:26] WARNING: src/c_api/c_api.cc:935: `ntree_limit` is deprecated, use `iteration_range` instead.
## [16:03:26] WARNING: src/c_api/c_api.cc:935: `ntree_limit` is deprecated, use `iteration_range` instead.
## [16:03:26] WARNING: src/c_api/c_api.cc:935: `ntree_limit` is deprecated, use `iteration_range` instead.
## [16:03:26] WARNING: src/c_api/c_api.cc:935: `ntree_limit` is deprecated, use `iteration_range` instead.
## [16:03:26] WARNING: src/c_api/c_api.cc:935: `ntree_limit` is deprecated, use `iteration_range` instead.
## [16:03:26] WARNING: src/c_api/c_api.cc:935: `ntree_limit` is deprecated, use `iteration_range` instead.
## [16:03:27] WARNING: src/c_api/c_api.cc:935: `ntree_limit` is deprecated, use `iteration_range` instead.
## [16:03:27] WARNING: src/c_api/c_api.cc:935: `ntree_limit` is deprecated, use `iteration_range` instead.
## [16:03:27] WARNING: src/c_api/c_api.cc:935: `ntree_limit` is deprecated, use `iteration_range` instead.
## [16:03:27] WARNING: src/c_api/c_api.cc:935: `ntree_limit` is deprecated, use `iteration_range` instead.
## [16:03:27] WARNING: src/c_api/c_api.cc:935: `ntree_limit` is deprecated, use `iteration_range` instead.
## [16:03:27] WARNING: src/c_api/c_api.cc:935: `ntree_limit` is deprecated, use `iteration_range` instead.
## [16:03:27] WARNING: src/c_api/c_api.cc:935: `ntree_limit` is deprecated, use `iteration_range` instead.
## [16:03:27] WARNING: src/c_api/c_api.cc:935: `ntree_limit` is deprecated, use `iteration_range` instead.
## [16:03:27] WARNING: src/c_api/c_api.cc:935: `ntree_limit` is deprecated, use `iteration_range` instead.
## [16:03:27] WARNING: src/c_api/c_api.cc:935: `ntree_limit` is deprecated, use `iteration_range` instead.
## [16:03:28] WARNING: src/c_api/c_api.cc:935: `ntree_limit` is deprecated, use `iteration_range` instead.
## [16:03:28] WARNING: src/c_api/c_api.cc:935: `ntree_limit` is deprecated, use `iteration_range` instead.
## [16:03:28] WARNING: src/c_api/c_api.cc:935: `ntree_limit` is deprecated, use `iteration_range` instead.
## [16:03:28] WARNING: src/c_api/c_api.cc:935: `ntree_limit` is deprecated, use `iteration_range` instead.
## [16:03:28] WARNING: src/c_api/c_api.cc:935: `ntree_limit` is deprecated, use `iteration_range` instead.
## [16:03:28] WARNING: src/c_api/c_api.cc:935: `ntree_limit` is deprecated, use `iteration_range` instead.
## [16:03:28] WARNING: src/c_api/c_api.cc:935: `ntree_limit` is deprecated, use `iteration_range` instead.
## [16:03:28] WARNING: src/c_api/c_api.cc:935: `ntree_limit` is deprecated, use `iteration_range` instead.
## [16:03:28] WARNING: src/c_api/c_api.cc:935: `ntree_limit` is deprecated, use `iteration_range` instead.
## [16:03:28] WARNING: src/c_api/c_api.cc:935: `ntree_limit` is deprecated, use `iteration_range` instead.
## [16:03:29] WARNING: src/c_api/c_api.cc:935: `ntree_limit` is deprecated, use `iteration_range` instead.
## [16:03:29] WARNING: src/c_api/c_api.cc:935: `ntree_limit` is deprecated, use `iteration_range` instead.
## [16:03:29] WARNING: src/c_api/c_api.cc:935: `ntree_limit` is deprecated, use `iteration_range` instead.
## [16:03:29] WARNING: src/c_api/c_api.cc:935: `ntree_limit` is deprecated, use `iteration_range` instead.
## [16:03:29] WARNING: src/c_api/c_api.cc:935: `ntree_limit` is deprecated, use `iteration_range` instead.
## [16:03:29] WARNING: src/c_api/c_api.cc:935: `ntree_limit` is deprecated, use `iteration_range` instead.
## [16:03:29] WARNING: src/c_api/c_api.cc:935: `ntree_limit` is deprecated, use `iteration_range` instead.
## [16:03:29] WARNING: src/c_api/c_api.cc:935: `ntree_limit` is deprecated, use `iteration_range` instead.
## [16:03:29] WARNING: src/c_api/c_api.cc:935: `ntree_limit` is deprecated, use `iteration_range` instead.
## [16:03:29] WARNING: src/c_api/c_api.cc:935: `ntree_limit` is deprecated, use `iteration_range` instead.
## [16:03:29] WARNING: src/c_api/c_api.cc:935: `ntree_limit` is deprecated, use `iteration_range` instead.
## [16:03:29] WARNING: src/c_api/c_api.cc:935: `ntree_limit` is deprecated, use `iteration_range` instead.
## [16:03:29] WARNING: src/c_api/c_api.cc:935: `ntree_limit` is deprecated, use `iteration_range` instead.
## [16:03:29] WARNING: src/c_api/c_api.cc:935: `ntree_limit` is deprecated, use `iteration_range` instead.
## [16:03:30] WARNING: src/c_api/c_api.cc:935: `ntree_limit` is deprecated, use `iteration_range` instead.
## [16:03:30] WARNING: src/c_api/c_api.cc:935: `ntree_limit` is deprecated, use `iteration_range` instead.
## [16:03:30] WARNING: src/c_api/c_api.cc:935: `ntree_limit` is deprecated, use `iteration_range` instead.
## [16:03:30] WARNING: src/c_api/c_api.cc:935: `ntree_limit` is deprecated, use `iteration_range` instead.
## [16:03:30] WARNING: src/c_api/c_api.cc:935: `ntree_limit` is deprecated, use `iteration_range` instead.
## [16:03:30] WARNING: src/c_api/c_api.cc:935: `ntree_limit` is deprecated, use `iteration_range` instead.
## [16:03:30] WARNING: src/c_api/c_api.cc:935: `ntree_limit` is deprecated, use `iteration_range` instead.
## [16:03:30] WARNING: src/c_api/c_api.cc:935: `ntree_limit` is deprecated, use `iteration_range` instead.
## [16:03:30] WARNING: src/c_api/c_api.cc:935: `ntree_limit` is deprecated, use `iteration_range` instead.
## [16:03:30] WARNING: src/c_api/c_api.cc:935: `ntree_limit` is deprecated, use `iteration_range` instead.
## [16:03:31] WARNING: src/c_api/c_api.cc:935: `ntree_limit` is deprecated, use `iteration_range` instead.
## [16:03:31] WARNING: src/c_api/c_api.cc:935: `ntree_limit` is deprecated, use `iteration_range` instead.
## [16:03:31] WARNING: src/c_api/c_api.cc:935: `ntree_limit` is deprecated, use `iteration_range` instead.
## [16:03:31] WARNING: src/c_api/c_api.cc:935: `ntree_limit` is deprecated, use `iteration_range` instead.
## [16:03:31] WARNING: src/c_api/c_api.cc:935: `ntree_limit` is deprecated, use `iteration_range` instead.
## [16:03:31] WARNING: src/c_api/c_api.cc:935: `ntree_limit` is deprecated, use `iteration_range` instead.
## [16:03:31] WARNING: src/c_api/c_api.cc:935: `ntree_limit` is deprecated, use `iteration_range` instead.
## [16:03:31] WARNING: src/c_api/c_api.cc:935: `ntree_limit` is deprecated, use `iteration_range` instead.
## [16:03:31] WARNING: src/c_api/c_api.cc:935: `ntree_limit` is deprecated, use `iteration_range` instead.
## [16:03:31] WARNING: src/c_api/c_api.cc:935: `ntree_limit` is deprecated, use `iteration_range` instead.
## [16:03:32] WARNING: src/c_api/c_api.cc:935: `ntree_limit` is deprecated, use `iteration_range` instead.
## [16:03:32] WARNING: src/c_api/c_api.cc:935: `ntree_limit` is deprecated, use `iteration_range` instead.
## [16:03:32] WARNING: src/c_api/c_api.cc:935: `ntree_limit` is deprecated, use `iteration_range` instead.
## [16:03:32] WARNING: src/c_api/c_api.cc:935: `ntree_limit` is deprecated, use `iteration_range` instead.
## [16:03:32] WARNING: src/c_api/c_api.cc:935: `ntree_limit` is deprecated, use `iteration_range` instead.
## [16:03:32] WARNING: src/c_api/c_api.cc:935: `ntree_limit` is deprecated, use `iteration_range` instead.
## [16:03:32] WARNING: src/c_api/c_api.cc:935: `ntree_limit` is deprecated, use `iteration_range` instead.
## [16:03:32] WARNING: src/c_api/c_api.cc:935: `ntree_limit` is deprecated, use `iteration_range` instead.
## [16:03:32] WARNING: src/c_api/c_api.cc:935: `ntree_limit` is deprecated, use `iteration_range` instead.
## [16:03:32] WARNING: src/c_api/c_api.cc:935: `ntree_limit` is deprecated, use `iteration_range` instead.
## [16:03:33] WARNING: src/c_api/c_api.cc:935: `ntree_limit` is deprecated, use `iteration_range` instead.
## [16:03:33] WARNING: src/c_api/c_api.cc:935: `ntree_limit` is deprecated, use `iteration_range` instead.
## [16:03:33] WARNING: src/c_api/c_api.cc:935: `ntree_limit` is deprecated, use `iteration_range` instead.
## [16:03:33] WARNING: src/c_api/c_api.cc:935: `ntree_limit` is deprecated, use `iteration_range` instead.
## [16:03:33] WARNING: src/c_api/c_api.cc:935: `ntree_limit` is deprecated, use `iteration_range` instead.
## [16:03:33] WARNING: src/c_api/c_api.cc:935: `ntree_limit` is deprecated, use `iteration_range` instead.
## [16:03:33] WARNING: src/c_api/c_api.cc:935: `ntree_limit` is deprecated, use `iteration_range` instead.
## [16:03:33] WARNING: src/c_api/c_api.cc:935: `ntree_limit` is deprecated, use `iteration_range` instead.
## [16:03:33] WARNING: src/c_api/c_api.cc:935: `ntree_limit` is deprecated, use `iteration_range` instead.
## [16:03:33] WARNING: src/c_api/c_api.cc:935: `ntree_limit` is deprecated, use `iteration_range` instead.
## [16:03:33] WARNING: src/c_api/c_api.cc:935: `ntree_limit` is deprecated, use `iteration_range` instead.
## [16:03:33] WARNING: src/c_api/c_api.cc:935: `ntree_limit` is deprecated, use `iteration_range` instead.
## [16:03:33] WARNING: src/c_api/c_api.cc:935: `ntree_limit` is deprecated, use `iteration_range` instead.
## [16:03:33] WARNING: src/c_api/c_api.cc:935: `ntree_limit` is deprecated, use `iteration_range` instead.
## [16:03:34] WARNING: src/c_api/c_api.cc:935: `ntree_limit` is deprecated, use `iteration_range` instead.
## [16:03:34] WARNING: src/c_api/c_api.cc:935: `ntree_limit` is deprecated, use `iteration_range` instead.
## [16:03:34] WARNING: src/c_api/c_api.cc:935: `ntree_limit` is deprecated, use `iteration_range` instead.
## [16:03:34] WARNING: src/c_api/c_api.cc:935: `ntree_limit` is deprecated, use `iteration_range` instead.
## [16:03:34] WARNING: src/c_api/c_api.cc:935: `ntree_limit` is deprecated, use `iteration_range` instead.
## [16:03:34] WARNING: src/c_api/c_api.cc:935: `ntree_limit` is deprecated, use `iteration_range` instead.
## [16:03:34] WARNING: src/c_api/c_api.cc:935: `ntree_limit` is deprecated, use `iteration_range` instead.
## [16:03:34] WARNING: src/c_api/c_api.cc:935: `ntree_limit` is deprecated, use `iteration_range` instead.
## [16:03:34] WARNING: src/c_api/c_api.cc:935: `ntree_limit` is deprecated, use `iteration_range` instead.
## [16:03:34] WARNING: src/c_api/c_api.cc:935: `ntree_limit` is deprecated, use `iteration_range` instead.
## [16:03:35] WARNING: src/c_api/c_api.cc:935: `ntree_limit` is deprecated, use `iteration_range` instead.
## [16:03:35] WARNING: src/c_api/c_api.cc:935: `ntree_limit` is deprecated, use `iteration_range` instead.
## [16:03:35] WARNING: src/c_api/c_api.cc:935: `ntree_limit` is deprecated, use `iteration_range` instead.
## [16:03:35] WARNING: src/c_api/c_api.cc:935: `ntree_limit` is deprecated, use `iteration_range` instead.
## [16:03:35] WARNING: src/c_api/c_api.cc:935: `ntree_limit` is deprecated, use `iteration_range` instead.
## [16:03:35] WARNING: src/c_api/c_api.cc:935: `ntree_limit` is deprecated, use `iteration_range` instead.
## [16:03:35] WARNING: src/c_api/c_api.cc:935: `ntree_limit` is deprecated, use `iteration_range` instead.
## [16:03:35] WARNING: src/c_api/c_api.cc:935: `ntree_limit` is deprecated, use `iteration_range` instead.
## [16:03:35] WARNING: src/c_api/c_api.cc:935: `ntree_limit` is deprecated, use `iteration_range` instead.
## [16:03:35] WARNING: src/c_api/c_api.cc:935: `ntree_limit` is deprecated, use `iteration_range` instead.
## [16:03:36] WARNING: src/c_api/c_api.cc:935: `ntree_limit` is deprecated, use `iteration_range` instead.
## [16:03:36] WARNING: src/c_api/c_api.cc:935: `ntree_limit` is deprecated, use `iteration_range` instead.
## [16:03:36] WARNING: src/c_api/c_api.cc:935: `ntree_limit` is deprecated, use `iteration_range` instead.
## [16:03:36] WARNING: src/c_api/c_api.cc:935: `ntree_limit` is deprecated, use `iteration_range` instead.
## [16:03:36] WARNING: src/c_api/c_api.cc:935: `ntree_limit` is deprecated, use `iteration_range` instead.
## [16:03:36] WARNING: src/c_api/c_api.cc:935: `ntree_limit` is deprecated, use `iteration_range` instead.
## [16:03:36] WARNING: src/c_api/c_api.cc:935: `ntree_limit` is deprecated, use `iteration_range` instead.
## [16:03:36] WARNING: src/c_api/c_api.cc:935: `ntree_limit` is deprecated, use `iteration_range` instead.
## [16:03:36] WARNING: src/c_api/c_api.cc:935: `ntree_limit` is deprecated, use `iteration_range` instead.
## [16:03:36] WARNING: src/c_api/c_api.cc:935: `ntree_limit` is deprecated, use `iteration_range` instead.
## [16:03:36] WARNING: src/c_api/c_api.cc:935: `ntree_limit` is deprecated, use `iteration_range` instead.
## [16:03:36] WARNING: src/c_api/c_api.cc:935: `ntree_limit` is deprecated, use `iteration_range` instead.
## [16:03:36] WARNING: src/c_api/c_api.cc:935: `ntree_limit` is deprecated, use `iteration_range` instead.
## [16:03:36] WARNING: src/c_api/c_api.cc:935: `ntree_limit` is deprecated, use `iteration_range` instead.
## [16:03:37] WARNING: src/c_api/c_api.cc:935: `ntree_limit` is deprecated, use `iteration_range` instead.
## [16:03:37] WARNING: src/c_api/c_api.cc:935: `ntree_limit` is deprecated, use `iteration_range` instead.
## [16:03:37] WARNING: src/c_api/c_api.cc:935: `ntree_limit` is deprecated, use `iteration_range` instead.
## [16:03:37] WARNING: src/c_api/c_api.cc:935: `ntree_limit` is deprecated, use `iteration_range` instead.
## [16:03:37] WARNING: src/c_api/c_api.cc:935: `ntree_limit` is deprecated, use `iteration_range` instead.
## [16:03:37] WARNING: src/c_api/c_api.cc:935: `ntree_limit` is deprecated, use `iteration_range` instead.
## [16:03:37] WARNING: src/c_api/c_api.cc:935: `ntree_limit` is deprecated, use `iteration_range` instead.
## [16:03:37] WARNING: src/c_api/c_api.cc:935: `ntree_limit` is deprecated, use `iteration_range` instead.
## [16:03:37] WARNING: src/c_api/c_api.cc:935: `ntree_limit` is deprecated, use `iteration_range` instead.
## [16:03:37] WARNING: src/c_api/c_api.cc:935: `ntree_limit` is deprecated, use `iteration_range` instead.
## [16:03:38] WARNING: src/c_api/c_api.cc:935: `ntree_limit` is deprecated, use `iteration_range` instead.
## [16:03:38] WARNING: src/c_api/c_api.cc:935: `ntree_limit` is deprecated, use `iteration_range` instead.
## [16:03:38] WARNING: src/c_api/c_api.cc:935: `ntree_limit` is deprecated, use `iteration_range` instead.
## [16:03:38] WARNING: src/c_api/c_api.cc:935: `ntree_limit` is deprecated, use `iteration_range` instead.
## [16:03:38] WARNING: src/c_api/c_api.cc:935: `ntree_limit` is deprecated, use `iteration_range` instead.
## [16:03:38] WARNING: src/c_api/c_api.cc:935: `ntree_limit` is deprecated, use `iteration_range` instead.
## [16:03:38] WARNING: src/c_api/c_api.cc:935: `ntree_limit` is deprecated, use `iteration_range` instead.
## [16:03:38] WARNING: src/c_api/c_api.cc:935: `ntree_limit` is deprecated, use `iteration_range` instead.
## [16:03:38] WARNING: src/c_api/c_api.cc:935: `ntree_limit` is deprecated, use `iteration_range` instead.
## [16:03:38] WARNING: src/c_api/c_api.cc:935: `ntree_limit` is deprecated, use `iteration_range` instead.
## [16:03:39] WARNING: src/c_api/c_api.cc:935: `ntree_limit` is deprecated, use `iteration_range` instead.
## [16:03:39] WARNING: src/c_api/c_api.cc:935: `ntree_limit` is deprecated, use `iteration_range` instead.
## [16:03:39] WARNING: src/c_api/c_api.cc:935: `ntree_limit` is deprecated, use `iteration_range` instead.
## [16:03:39] WARNING: src/c_api/c_api.cc:935: `ntree_limit` is deprecated, use `iteration_range` instead.
## [16:03:39] WARNING: src/c_api/c_api.cc:935: `ntree_limit` is deprecated, use `iteration_range` instead.
## [16:03:39] WARNING: src/c_api/c_api.cc:935: `ntree_limit` is deprecated, use `iteration_range` instead.
## [16:03:39] WARNING: src/c_api/c_api.cc:935: `ntree_limit` is deprecated, use `iteration_range` instead.
## [16:03:39] WARNING: src/c_api/c_api.cc:935: `ntree_limit` is deprecated, use `iteration_range` instead.
## [16:03:39] WARNING: src/c_api/c_api.cc:935: `ntree_limit` is deprecated, use `iteration_range` instead.
## [16:03:39] WARNING: src/c_api/c_api.cc:935: `ntree_limit` is deprecated, use `iteration_range` instead.
## [16:03:39] WARNING: src/c_api/c_api.cc:935: `ntree_limit` is deprecated, use `iteration_range` instead.
## [16:03:39] WARNING: src/c_api/c_api.cc:935: `ntree_limit` is deprecated, use `iteration_range` instead.
## [16:03:39] WARNING: src/c_api/c_api.cc:935: `ntree_limit` is deprecated, use `iteration_range` instead.
## [16:03:39] WARNING: src/c_api/c_api.cc:935: `ntree_limit` is deprecated, use `iteration_range` instead.
## [16:03:40] WARNING: src/c_api/c_api.cc:935: `ntree_limit` is deprecated, use `iteration_range` instead.
## [16:03:40] WARNING: src/c_api/c_api.cc:935: `ntree_limit` is deprecated, use `iteration_range` instead.
## [16:03:40] WARNING: src/c_api/c_api.cc:935: `ntree_limit` is deprecated, use `iteration_range` instead.
## [16:03:40] WARNING: src/c_api/c_api.cc:935: `ntree_limit` is deprecated, use `iteration_range` instead.
## [16:03:40] WARNING: src/c_api/c_api.cc:935: `ntree_limit` is deprecated, use `iteration_range` instead.
## [16:03:40] WARNING: src/c_api/c_api.cc:935: `ntree_limit` is deprecated, use `iteration_range` instead.
## [16:03:40] WARNING: src/c_api/c_api.cc:935: `ntree_limit` is deprecated, use `iteration_range` instead.
## [16:03:40] WARNING: src/c_api/c_api.cc:935: `ntree_limit` is deprecated, use `iteration_range` instead.
## [16:03:40] WARNING: src/c_api/c_api.cc:935: `ntree_limit` is deprecated, use `iteration_range` instead.
## [16:03:40] WARNING: src/c_api/c_api.cc:935: `ntree_limit` is deprecated, use `iteration_range` instead.
## [16:03:41] WARNING: src/c_api/c_api.cc:935: `ntree_limit` is deprecated, use `iteration_range` instead.
## [16:03:41] WARNING: src/c_api/c_api.cc:935: `ntree_limit` is deprecated, use `iteration_range` instead.
## [16:03:41] WARNING: src/c_api/c_api.cc:935: `ntree_limit` is deprecated, use `iteration_range` instead.
## [16:03:41] WARNING: src/c_api/c_api.cc:935: `ntree_limit` is deprecated, use `iteration_range` instead.
## [16:03:41] WARNING: src/c_api/c_api.cc:935: `ntree_limit` is deprecated, use `iteration_range` instead.
## [16:03:41] WARNING: src/c_api/c_api.cc:935: `ntree_limit` is deprecated, use `iteration_range` instead.
## [16:03:41] WARNING: src/c_api/c_api.cc:935: `ntree_limit` is deprecated, use `iteration_range` instead.
## [16:03:41] WARNING: src/c_api/c_api.cc:935: `ntree_limit` is deprecated, use `iteration_range` instead.
## [16:03:41] WARNING: src/c_api/c_api.cc:935: `ntree_limit` is deprecated, use `iteration_range` instead.
## [16:03:41] WARNING: src/c_api/c_api.cc:935: `ntree_limit` is deprecated, use `iteration_range` instead.
## [16:03:42] WARNING: src/c_api/c_api.cc:935: `ntree_limit` is deprecated, use `iteration_range` instead.
## [16:03:42] WARNING: src/c_api/c_api.cc:935: `ntree_limit` is deprecated, use `iteration_range` instead.
## [16:03:42] WARNING: src/c_api/c_api.cc:935: `ntree_limit` is deprecated, use `iteration_range` instead.
## [16:03:42] WARNING: src/c_api/c_api.cc:935: `ntree_limit` is deprecated, use `iteration_range` instead.
## [16:03:42] WARNING: src/c_api/c_api.cc:935: `ntree_limit` is deprecated, use `iteration_range` instead.
## [16:03:42] WARNING: src/c_api/c_api.cc:935: `ntree_limit` is deprecated, use `iteration_range` instead.
## [16:03:42] WARNING: src/c_api/c_api.cc:935: `ntree_limit` is deprecated, use `iteration_range` instead.
## [16:03:42] WARNING: src/c_api/c_api.cc:935: `ntree_limit` is deprecated, use `iteration_range` instead.
## [16:03:42] WARNING: src/c_api/c_api.cc:935: `ntree_limit` is deprecated, use `iteration_range` instead.
## [16:03:42] WARNING: src/c_api/c_api.cc:935: `ntree_limit` is deprecated, use `iteration_range` instead.
## [16:03:42] WARNING: src/c_api/c_api.cc:935: `ntree_limit` is deprecated, use `iteration_range` instead.
## [16:03:42] WARNING: src/c_api/c_api.cc:935: `ntree_limit` is deprecated, use `iteration_range` instead.
## [16:03:42] WARNING: src/c_api/c_api.cc:935: `ntree_limit` is deprecated, use `iteration_range` instead.
## [16:03:42] WARNING: src/c_api/c_api.cc:935: `ntree_limit` is deprecated, use `iteration_range` instead.
## [16:03:43] WARNING: src/c_api/c_api.cc:935: `ntree_limit` is deprecated, use `iteration_range` instead.
## [16:03:43] WARNING: src/c_api/c_api.cc:935: `ntree_limit` is deprecated, use `iteration_range` instead.
## [16:03:43] WARNING: src/c_api/c_api.cc:935: `ntree_limit` is deprecated, use `iteration_range` instead.
## [16:03:43] WARNING: src/c_api/c_api.cc:935: `ntree_limit` is deprecated, use `iteration_range` instead.
## [16:03:43] WARNING: src/c_api/c_api.cc:935: `ntree_limit` is deprecated, use `iteration_range` instead.
## [16:03:43] WARNING: src/c_api/c_api.cc:935: `ntree_limit` is deprecated, use `iteration_range` instead.
## [16:03:43] WARNING: src/c_api/c_api.cc:935: `ntree_limit` is deprecated, use `iteration_range` instead.
## [16:03:43] WARNING: src/c_api/c_api.cc:935: `ntree_limit` is deprecated, use `iteration_range` instead.
## [16:03:43] WARNING: src/c_api/c_api.cc:935: `ntree_limit` is deprecated, use `iteration_range` instead.
## [16:03:43] WARNING: src/c_api/c_api.cc:935: `ntree_limit` is deprecated, use `iteration_range` instead.
## [16:03:44] WARNING: src/c_api/c_api.cc:935: `ntree_limit` is deprecated, use `iteration_range` instead.
## [16:03:44] WARNING: src/c_api/c_api.cc:935: `ntree_limit` is deprecated, use `iteration_range` instead.
## [16:03:44] WARNING: src/c_api/c_api.cc:935: `ntree_limit` is deprecated, use `iteration_range` instead.
## [16:03:44] WARNING: src/c_api/c_api.cc:935: `ntree_limit` is deprecated, use `iteration_range` instead.
## [16:03:44] WARNING: src/c_api/c_api.cc:935: `ntree_limit` is deprecated, use `iteration_range` instead.
## [16:03:44] WARNING: src/c_api/c_api.cc:935: `ntree_limit` is deprecated, use `iteration_range` instead.
## [16:03:44] WARNING: src/c_api/c_api.cc:935: `ntree_limit` is deprecated, use `iteration_range` instead.
## [16:03:44] WARNING: src/c_api/c_api.cc:935: `ntree_limit` is deprecated, use `iteration_range` instead.
## [16:03:44] WARNING: src/c_api/c_api.cc:935: `ntree_limit` is deprecated, use `iteration_range` instead.
## [16:03:44] WARNING: src/c_api/c_api.cc:935: `ntree_limit` is deprecated, use `iteration_range` instead.
## [16:03:45] WARNING: src/c_api/c_api.cc:935: `ntree_limit` is deprecated, use `iteration_range` instead.
## [16:03:45] WARNING: src/c_api/c_api.cc:935: `ntree_limit` is deprecated, use `iteration_range` instead.
## [16:03:45] WARNING: src/c_api/c_api.cc:935: `ntree_limit` is deprecated, use `iteration_range` instead.
## [16:03:45] WARNING: src/c_api/c_api.cc:935: `ntree_limit` is deprecated, use `iteration_range` instead.
## [16:03:45] WARNING: src/c_api/c_api.cc:935: `ntree_limit` is deprecated, use `iteration_range` instead.
## [16:03:45] WARNING: src/c_api/c_api.cc:935: `ntree_limit` is deprecated, use `iteration_range` instead.
## [16:03:45] WARNING: src/c_api/c_api.cc:935: `ntree_limit` is deprecated, use `iteration_range` instead.
## [16:03:45] WARNING: src/c_api/c_api.cc:935: `ntree_limit` is deprecated, use `iteration_range` instead.
## [16:03:45] WARNING: src/c_api/c_api.cc:935: `ntree_limit` is deprecated, use `iteration_range` instead.
## [16:03:45] WARNING: src/c_api/c_api.cc:935: `ntree_limit` is deprecated, use `iteration_range` instead.
## [16:03:46] WARNING: src/c_api/c_api.cc:935: `ntree_limit` is deprecated, use `iteration_range` instead.
## [16:03:46] WARNING: src/c_api/c_api.cc:935: `ntree_limit` is deprecated, use `iteration_range` instead.
## [16:03:46] WARNING: src/c_api/c_api.cc:935: `ntree_limit` is deprecated, use `iteration_range` instead.
## [16:03:46] WARNING: src/c_api/c_api.cc:935: `ntree_limit` is deprecated, use `iteration_range` instead.
## [16:03:46] WARNING: src/c_api/c_api.cc:935: `ntree_limit` is deprecated, use `iteration_range` instead.
## [16:03:46] WARNING: src/c_api/c_api.cc:935: `ntree_limit` is deprecated, use `iteration_range` instead.
## [16:03:46] WARNING: src/c_api/c_api.cc:935: `ntree_limit` is deprecated, use `iteration_range` instead.
## [16:03:46] WARNING: src/c_api/c_api.cc:935: `ntree_limit` is deprecated, use `iteration_range` instead.
## [16:03:46] WARNING: src/c_api/c_api.cc:935: `ntree_limit` is deprecated, use `iteration_range` instead.
## [16:03:46] WARNING: src/c_api/c_api.cc:935: `ntree_limit` is deprecated, use `iteration_range` instead.
## [16:03:46] WARNING: src/c_api/c_api.cc:935: `ntree_limit` is deprecated, use `iteration_range` instead.
## [16:03:46] WARNING: src/c_api/c_api.cc:935: `ntree_limit` is deprecated, use `iteration_range` instead.
## [16:03:46] WARNING: src/c_api/c_api.cc:935: `ntree_limit` is deprecated, use `iteration_range` instead.
## [16:03:46] WARNING: src/c_api/c_api.cc:935: `ntree_limit` is deprecated, use `iteration_range` instead.
## [16:03:47] WARNING: src/c_api/c_api.cc:935: `ntree_limit` is deprecated, use `iteration_range` instead.
## [16:03:47] WARNING: src/c_api/c_api.cc:935: `ntree_limit` is deprecated, use `iteration_range` instead.
## [16:03:47] WARNING: src/c_api/c_api.cc:935: `ntree_limit` is deprecated, use `iteration_range` instead.
## [16:03:47] WARNING: src/c_api/c_api.cc:935: `ntree_limit` is deprecated, use `iteration_range` instead.
## [16:03:47] WARNING: src/c_api/c_api.cc:935: `ntree_limit` is deprecated, use `iteration_range` instead.
## [16:03:47] WARNING: src/c_api/c_api.cc:935: `ntree_limit` is deprecated, use `iteration_range` instead.
## [16:03:47] WARNING: src/c_api/c_api.cc:935: `ntree_limit` is deprecated, use `iteration_range` instead.
## [16:03:47] WARNING: src/c_api/c_api.cc:935: `ntree_limit` is deprecated, use `iteration_range` instead.
## [16:03:47] WARNING: src/c_api/c_api.cc:935: `ntree_limit` is deprecated, use `iteration_range` instead.
## [16:03:47] WARNING: src/c_api/c_api.cc:935: `ntree_limit` is deprecated, use `iteration_range` instead.
## [16:03:48] WARNING: src/c_api/c_api.cc:935: `ntree_limit` is deprecated, use `iteration_range` instead.
## [16:03:48] WARNING: src/c_api/c_api.cc:935: `ntree_limit` is deprecated, use `iteration_range` instead.
## [16:03:48] WARNING: src/c_api/c_api.cc:935: `ntree_limit` is deprecated, use `iteration_range` instead.
## [16:03:48] WARNING: src/c_api/c_api.cc:935: `ntree_limit` is deprecated, use `iteration_range` instead.
## [16:03:48] WARNING: src/c_api/c_api.cc:935: `ntree_limit` is deprecated, use `iteration_range` instead.
## [16:03:48] WARNING: src/c_api/c_api.cc:935: `ntree_limit` is deprecated, use `iteration_range` instead.
## [16:03:48] WARNING: src/c_api/c_api.cc:935: `ntree_limit` is deprecated, use `iteration_range` instead.
## [16:03:48] WARNING: src/c_api/c_api.cc:935: `ntree_limit` is deprecated, use `iteration_range` instead.
## [16:03:51] WARNING: src/c_api/c_api.cc:935: `ntree_limit` is deprecated, use `iteration_range` instead.
## [16:03:51] WARNING: src/c_api/c_api.cc:935: `ntree_limit` is deprecated, use `iteration_range` instead.
## [16:03:51] WARNING: src/c_api/c_api.cc:935: `ntree_limit` is deprecated, use `iteration_range` instead.
## [16:03:51] WARNING: src/c_api/c_api.cc:935: `ntree_limit` is deprecated, use `iteration_range` instead.
## [16:03:51] WARNING: src/c_api/c_api.cc:935: `ntree_limit` is deprecated, use `iteration_range` instead.
## [16:03:51] WARNING: src/c_api/c_api.cc:935: `ntree_limit` is deprecated, use `iteration_range` instead.
## [16:03:51] WARNING: src/c_api/c_api.cc:935: `ntree_limit` is deprecated, use `iteration_range` instead.
## [16:03:51] WARNING: src/c_api/c_api.cc:935: `ntree_limit` is deprecated, use `iteration_range` instead.
## [16:03:51] WARNING: src/c_api/c_api.cc:935: `ntree_limit` is deprecated, use `iteration_range` instead.
## [16:03:51] WARNING: src/c_api/c_api.cc:935: `ntree_limit` is deprecated, use `iteration_range` instead.
## [16:03:52] WARNING: src/c_api/c_api.cc:935: `ntree_limit` is deprecated, use `iteration_range` instead.
## [16:03:52] WARNING: src/c_api/c_api.cc:935: `ntree_limit` is deprecated, use `iteration_range` instead.
## [16:03:52] WARNING: src/c_api/c_api.cc:935: `ntree_limit` is deprecated, use `iteration_range` instead.
## [16:03:52] WARNING: src/c_api/c_api.cc:935: `ntree_limit` is deprecated, use `iteration_range` instead.
## [16:03:52] WARNING: src/c_api/c_api.cc:935: `ntree_limit` is deprecated, use `iteration_range` instead.
## [16:03:52] WARNING: src/c_api/c_api.cc:935: `ntree_limit` is deprecated, use `iteration_range` instead.
## [16:03:52] WARNING: src/c_api/c_api.cc:935: `ntree_limit` is deprecated, use `iteration_range` instead.
## [16:03:52] WARNING: src/c_api/c_api.cc:935: `ntree_limit` is deprecated, use `iteration_range` instead.
## [16:03:52] WARNING: src/c_api/c_api.cc:935: `ntree_limit` is deprecated, use `iteration_range` instead.
## [16:03:52] WARNING: src/c_api/c_api.cc:935: `ntree_limit` is deprecated, use `iteration_range` instead.
## [16:03:52] WARNING: src/c_api/c_api.cc:935: `ntree_limit` is deprecated, use `iteration_range` instead.
## [16:03:52] WARNING: src/c_api/c_api.cc:935: `ntree_limit` is deprecated, use `iteration_range` instead.
## [16:03:53] WARNING: src/c_api/c_api.cc:935: `ntree_limit` is deprecated, use `iteration_range` instead.
## [16:03:53] WARNING: src/c_api/c_api.cc:935: `ntree_limit` is deprecated, use `iteration_range` instead.
## [16:03:53] WARNING: src/c_api/c_api.cc:935: `ntree_limit` is deprecated, use `iteration_range` instead.
## [16:03:53] WARNING: src/c_api/c_api.cc:935: `ntree_limit` is deprecated, use `iteration_range` instead.
## [16:03:53] WARNING: src/c_api/c_api.cc:935: `ntree_limit` is deprecated, use `iteration_range` instead.
## [16:03:53] WARNING: src/c_api/c_api.cc:935: `ntree_limit` is deprecated, use `iteration_range` instead.
## [16:03:53] WARNING: src/c_api/c_api.cc:935: `ntree_limit` is deprecated, use `iteration_range` instead.
## [16:03:53] WARNING: src/c_api/c_api.cc:935: `ntree_limit` is deprecated, use `iteration_range` instead.
## [16:03:54] WARNING: src/c_api/c_api.cc:935: `ntree_limit` is deprecated, use `iteration_range` instead.
## [16:03:54] WARNING: src/c_api/c_api.cc:935: `ntree_limit` is deprecated, use `iteration_range` instead.
## [16:03:54] WARNING: src/c_api/c_api.cc:935: `ntree_limit` is deprecated, use `iteration_range` instead.
## [16:03:54] WARNING: src/c_api/c_api.cc:935: `ntree_limit` is deprecated, use `iteration_range` instead.
## [16:03:54] WARNING: src/c_api/c_api.cc:935: `ntree_limit` is deprecated, use `iteration_range` instead.
## [16:03:54] WARNING: src/c_api/c_api.cc:935: `ntree_limit` is deprecated, use `iteration_range` instead.
## [16:03:54] WARNING: src/c_api/c_api.cc:935: `ntree_limit` is deprecated, use `iteration_range` instead.
## [16:03:54] WARNING: src/c_api/c_api.cc:935: `ntree_limit` is deprecated, use `iteration_range` instead.
## [16:03:54] WARNING: src/c_api/c_api.cc:935: `ntree_limit` is deprecated, use `iteration_range` instead.
## [16:03:54] WARNING: src/c_api/c_api.cc:935: `ntree_limit` is deprecated, use `iteration_range` instead.
## [16:03:55] WARNING: src/c_api/c_api.cc:935: `ntree_limit` is deprecated, use `iteration_range` instead.
## [16:03:55] WARNING: src/c_api/c_api.cc:935: `ntree_limit` is deprecated, use `iteration_range` instead.
## [16:03:55] WARNING: src/c_api/c_api.cc:935: `ntree_limit` is deprecated, use `iteration_range` instead.
## [16:03:55] WARNING: src/c_api/c_api.cc:935: `ntree_limit` is deprecated, use `iteration_range` instead.
## [16:03:55] WARNING: src/c_api/c_api.cc:935: `ntree_limit` is deprecated, use `iteration_range` instead.
## [16:03:55] WARNING: src/c_api/c_api.cc:935: `ntree_limit` is deprecated, use `iteration_range` instead.
## [16:03:55] WARNING: src/c_api/c_api.cc:935: `ntree_limit` is deprecated, use `iteration_range` instead.
## [16:03:55] WARNING: src/c_api/c_api.cc:935: `ntree_limit` is deprecated, use `iteration_range` instead.
## [16:03:55] WARNING: src/c_api/c_api.cc:935: `ntree_limit` is deprecated, use `iteration_range` instead.
## [16:03:55] WARNING: src/c_api/c_api.cc:935: `ntree_limit` is deprecated, use `iteration_range` instead.
## [16:03:55] WARNING: src/c_api/c_api.cc:935: `ntree_limit` is deprecated, use `iteration_range` instead.
## [16:03:55] WARNING: src/c_api/c_api.cc:935: `ntree_limit` is deprecated, use `iteration_range` instead.
## [16:03:55] WARNING: src/c_api/c_api.cc:935: `ntree_limit` is deprecated, use `iteration_range` instead.
## [16:03:55] WARNING: src/c_api/c_api.cc:935: `ntree_limit` is deprecated, use `iteration_range` instead.
## [16:03:56] WARNING: src/c_api/c_api.cc:935: `ntree_limit` is deprecated, use `iteration_range` instead.
## [16:03:56] WARNING: src/c_api/c_api.cc:935: `ntree_limit` is deprecated, use `iteration_range` instead.
## [16:03:56] WARNING: src/c_api/c_api.cc:935: `ntree_limit` is deprecated, use `iteration_range` instead.
## [16:03:56] WARNING: src/c_api/c_api.cc:935: `ntree_limit` is deprecated, use `iteration_range` instead.
## [16:03:56] WARNING: src/c_api/c_api.cc:935: `ntree_limit` is deprecated, use `iteration_range` instead.
## [16:03:56] WARNING: src/c_api/c_api.cc:935: `ntree_limit` is deprecated, use `iteration_range` instead.
## [16:03:56] WARNING: src/c_api/c_api.cc:935: `ntree_limit` is deprecated, use `iteration_range` instead.
## [16:03:56] WARNING: src/c_api/c_api.cc:935: `ntree_limit` is deprecated, use `iteration_range` instead.
## [16:03:56] WARNING: src/c_api/c_api.cc:935: `ntree_limit` is deprecated, use `iteration_range` instead.
## [16:03:56] WARNING: src/c_api/c_api.cc:935: `ntree_limit` is deprecated, use `iteration_range` instead.
## [16:03:57] WARNING: src/c_api/c_api.cc:935: `ntree_limit` is deprecated, use `iteration_range` instead.
## [16:03:57] WARNING: src/c_api/c_api.cc:935: `ntree_limit` is deprecated, use `iteration_range` instead.
## [16:03:57] WARNING: src/c_api/c_api.cc:935: `ntree_limit` is deprecated, use `iteration_range` instead.
## [16:03:57] WARNING: src/c_api/c_api.cc:935: `ntree_limit` is deprecated, use `iteration_range` instead.
## [16:03:57] WARNING: src/c_api/c_api.cc:935: `ntree_limit` is deprecated, use `iteration_range` instead.
## [16:03:57] WARNING: src/c_api/c_api.cc:935: `ntree_limit` is deprecated, use `iteration_range` instead.
## [16:03:57] WARNING: src/c_api/c_api.cc:935: `ntree_limit` is deprecated, use `iteration_range` instead.
## [16:03:57] WARNING: src/c_api/c_api.cc:935: `ntree_limit` is deprecated, use `iteration_range` instead.
## [16:03:57] WARNING: src/c_api/c_api.cc:935: `ntree_limit` is deprecated, use `iteration_range` instead.
## [16:03:57] WARNING: src/c_api/c_api.cc:935: `ntree_limit` is deprecated, use `iteration_range` instead.
## [16:03:58] WARNING: src/c_api/c_api.cc:935: `ntree_limit` is deprecated, use `iteration_range` instead.
## [16:03:58] WARNING: src/c_api/c_api.cc:935: `ntree_limit` is deprecated, use `iteration_range` instead.
## [16:03:58] WARNING: src/c_api/c_api.cc:935: `ntree_limit` is deprecated, use `iteration_range` instead.
## [16:03:58] WARNING: src/c_api/c_api.cc:935: `ntree_limit` is deprecated, use `iteration_range` instead.
## [16:03:58] WARNING: src/c_api/c_api.cc:935: `ntree_limit` is deprecated, use `iteration_range` instead.
## [16:03:58] WARNING: src/c_api/c_api.cc:935: `ntree_limit` is deprecated, use `iteration_range` instead.
## [16:03:58] WARNING: src/c_api/c_api.cc:935: `ntree_limit` is deprecated, use `iteration_range` instead.
## [16:03:58] WARNING: src/c_api/c_api.cc:935: `ntree_limit` is deprecated, use `iteration_range` instead.
## [16:03:58] WARNING: src/c_api/c_api.cc:935: `ntree_limit` is deprecated, use `iteration_range` instead.
## [16:03:58] WARNING: src/c_api/c_api.cc:935: `ntree_limit` is deprecated, use `iteration_range` instead.
## [16:03:58] WARNING: src/c_api/c_api.cc:935: `ntree_limit` is deprecated, use `iteration_range` instead.
## [16:03:58] WARNING: src/c_api/c_api.cc:935: `ntree_limit` is deprecated, use `iteration_range` instead.
## [16:03:58] WARNING: src/c_api/c_api.cc:935: `ntree_limit` is deprecated, use `iteration_range` instead.
## [16:03:58] WARNING: src/c_api/c_api.cc:935: `ntree_limit` is deprecated, use `iteration_range` instead.
## [16:03:59] WARNING: src/c_api/c_api.cc:935: `ntree_limit` is deprecated, use `iteration_range` instead.
## [16:03:59] WARNING: src/c_api/c_api.cc:935: `ntree_limit` is deprecated, use `iteration_range` instead.
## [16:03:59] WARNING: src/c_api/c_api.cc:935: `ntree_limit` is deprecated, use `iteration_range` instead.
## [16:03:59] WARNING: src/c_api/c_api.cc:935: `ntree_limit` is deprecated, use `iteration_range` instead.
## [16:03:59] WARNING: src/c_api/c_api.cc:935: `ntree_limit` is deprecated, use `iteration_range` instead.
## [16:03:59] WARNING: src/c_api/c_api.cc:935: `ntree_limit` is deprecated, use `iteration_range` instead.
## [16:03:59] WARNING: src/c_api/c_api.cc:935: `ntree_limit` is deprecated, use `iteration_range` instead.
## [16:03:59] WARNING: src/c_api/c_api.cc:935: `ntree_limit` is deprecated, use `iteration_range` instead.
## [16:03:59] WARNING: src/c_api/c_api.cc:935: `ntree_limit` is deprecated, use `iteration_range` instead.
## [16:03:59] WARNING: src/c_api/c_api.cc:935: `ntree_limit` is deprecated, use `iteration_range` instead.
## [16:04:00] WARNING: src/c_api/c_api.cc:935: `ntree_limit` is deprecated, use `iteration_range` instead.
## [16:04:00] WARNING: src/c_api/c_api.cc:935: `ntree_limit` is deprecated, use `iteration_range` instead.
## [16:04:00] WARNING: src/c_api/c_api.cc:935: `ntree_limit` is deprecated, use `iteration_range` instead.
## [16:04:00] WARNING: src/c_api/c_api.cc:935: `ntree_limit` is deprecated, use `iteration_range` instead.
## [16:04:00] WARNING: src/c_api/c_api.cc:935: `ntree_limit` is deprecated, use `iteration_range` instead.
## [16:04:00] WARNING: src/c_api/c_api.cc:935: `ntree_limit` is deprecated, use `iteration_range` instead.
## [16:04:00] WARNING: src/c_api/c_api.cc:935: `ntree_limit` is deprecated, use `iteration_range` instead.
## [16:04:00] WARNING: src/c_api/c_api.cc:935: `ntree_limit` is deprecated, use `iteration_range` instead.
## [16:04:00] WARNING: src/c_api/c_api.cc:935: `ntree_limit` is deprecated, use `iteration_range` instead.
## [16:04:00] WARNING: src/c_api/c_api.cc:935: `ntree_limit` is deprecated, use `iteration_range` instead.
## [16:04:01] WARNING: src/c_api/c_api.cc:935: `ntree_limit` is deprecated, use `iteration_range` instead.
## [16:04:01] WARNING: src/c_api/c_api.cc:935: `ntree_limit` is deprecated, use `iteration_range` instead.
## [16:04:01] WARNING: src/c_api/c_api.cc:935: `ntree_limit` is deprecated, use `iteration_range` instead.
## [16:04:01] WARNING: src/c_api/c_api.cc:935: `ntree_limit` is deprecated, use `iteration_range` instead.
## [16:04:01] WARNING: src/c_api/c_api.cc:935: `ntree_limit` is deprecated, use `iteration_range` instead.
## [16:04:01] WARNING: src/c_api/c_api.cc:935: `ntree_limit` is deprecated, use `iteration_range` instead.
## [16:04:01] WARNING: src/c_api/c_api.cc:935: `ntree_limit` is deprecated, use `iteration_range` instead.
## [16:04:01] WARNING: src/c_api/c_api.cc:935: `ntree_limit` is deprecated, use `iteration_range` instead.
## [16:04:01] WARNING: src/c_api/c_api.cc:935: `ntree_limit` is deprecated, use `iteration_range` instead.
## [16:04:01] WARNING: src/c_api/c_api.cc:935: `ntree_limit` is deprecated, use `iteration_range` instead.
## [16:04:01] WARNING: src/c_api/c_api.cc:935: `ntree_limit` is deprecated, use `iteration_range` instead.
## [16:04:01] WARNING: src/c_api/c_api.cc:935: `ntree_limit` is deprecated, use `iteration_range` instead.
## [16:04:02] WARNING: src/c_api/c_api.cc:935: `ntree_limit` is deprecated, use `iteration_range` instead.
## [16:04:02] WARNING: src/c_api/c_api.cc:935: `ntree_limit` is deprecated, use `iteration_range` instead.
## [16:04:02] WARNING: src/c_api/c_api.cc:935: `ntree_limit` is deprecated, use `iteration_range` instead.
## [16:04:02] WARNING: src/c_api/c_api.cc:935: `ntree_limit` is deprecated, use `iteration_range` instead.
## [16:04:02] WARNING: src/c_api/c_api.cc:935: `ntree_limit` is deprecated, use `iteration_range` instead.
## [16:04:02] WARNING: src/c_api/c_api.cc:935: `ntree_limit` is deprecated, use `iteration_range` instead.
## [16:04:02] WARNING: src/c_api/c_api.cc:935: `ntree_limit` is deprecated, use `iteration_range` instead.
## [16:04:02] WARNING: src/c_api/c_api.cc:935: `ntree_limit` is deprecated, use `iteration_range` instead.
## [16:04:02] WARNING: src/c_api/c_api.cc:935: `ntree_limit` is deprecated, use `iteration_range` instead.
## [16:04:02] WARNING: src/c_api/c_api.cc:935: `ntree_limit` is deprecated, use `iteration_range` instead.
## [16:04:02] WARNING: src/c_api/c_api.cc:935: `ntree_limit` is deprecated, use `iteration_range` instead.
## [16:04:02] WARNING: src/c_api/c_api.cc:935: `ntree_limit` is deprecated, use `iteration_range` instead.
## [16:04:03] WARNING: src/c_api/c_api.cc:935: `ntree_limit` is deprecated, use `iteration_range` instead.
## [16:04:03] WARNING: src/c_api/c_api.cc:935: `ntree_limit` is deprecated, use `iteration_range` instead.
## [16:04:03] WARNING: src/c_api/c_api.cc:935: `ntree_limit` is deprecated, use `iteration_range` instead.
## [16:04:03] WARNING: src/c_api/c_api.cc:935: `ntree_limit` is deprecated, use `iteration_range` instead.
## [16:04:03] WARNING: src/c_api/c_api.cc:935: `ntree_limit` is deprecated, use `iteration_range` instead.
## [16:04:03] WARNING: src/c_api/c_api.cc:935: `ntree_limit` is deprecated, use `iteration_range` instead.
## [16:04:03] WARNING: src/c_api/c_api.cc:935: `ntree_limit` is deprecated, use `iteration_range` instead.
## [16:04:03] WARNING: src/c_api/c_api.cc:935: `ntree_limit` is deprecated, use `iteration_range` instead.
## [16:04:03] WARNING: src/c_api/c_api.cc:935: `ntree_limit` is deprecated, use `iteration_range` instead.
## [16:04:03] WARNING: src/c_api/c_api.cc:935: `ntree_limit` is deprecated, use `iteration_range` instead.
## [16:04:04] WARNING: src/c_api/c_api.cc:935: `ntree_limit` is deprecated, use `iteration_range` instead.
## [16:04:04] WARNING: src/c_api/c_api.cc:935: `ntree_limit` is deprecated, use `iteration_range` instead.
## [16:04:04] WARNING: src/c_api/c_api.cc:935: `ntree_limit` is deprecated, use `iteration_range` instead.
## [16:04:04] WARNING: src/c_api/c_api.cc:935: `ntree_limit` is deprecated, use `iteration_range` instead.
## [16:04:04] WARNING: src/c_api/c_api.cc:935: `ntree_limit` is deprecated, use `iteration_range` instead.
## [16:04:04] WARNING: src/c_api/c_api.cc:935: `ntree_limit` is deprecated, use `iteration_range` instead.
## [16:04:04] WARNING: src/c_api/c_api.cc:935: `ntree_limit` is deprecated, use `iteration_range` instead.
## [16:04:04] WARNING: src/c_api/c_api.cc:935: `ntree_limit` is deprecated, use `iteration_range` instead.
## [16:04:04] WARNING: src/c_api/c_api.cc:935: `ntree_limit` is deprecated, use `iteration_range` instead.
## [16:04:04] WARNING: src/c_api/c_api.cc:935: `ntree_limit` is deprecated, use `iteration_range` instead.
## [16:04:04] WARNING: src/c_api/c_api.cc:935: `ntree_limit` is deprecated, use `iteration_range` instead.
## [16:04:04] WARNING: src/c_api/c_api.cc:935: `ntree_limit` is deprecated, use `iteration_range` instead.
## [16:04:04] WARNING: src/c_api/c_api.cc:935: `ntree_limit` is deprecated, use `iteration_range` instead.
## [16:04:04] WARNING: src/c_api/c_api.cc:935: `ntree_limit` is deprecated, use `iteration_range` instead.
## [16:04:05] WARNING: src/c_api/c_api.cc:935: `ntree_limit` is deprecated, use `iteration_range` instead.
## [16:04:05] WARNING: src/c_api/c_api.cc:935: `ntree_limit` is deprecated, use `iteration_range` instead.
## [16:04:05] WARNING: src/c_api/c_api.cc:935: `ntree_limit` is deprecated, use `iteration_range` instead.
## [16:04:05] WARNING: src/c_api/c_api.cc:935: `ntree_limit` is deprecated, use `iteration_range` instead.
## [16:04:05] WARNING: src/c_api/c_api.cc:935: `ntree_limit` is deprecated, use `iteration_range` instead.
## [16:04:05] WARNING: src/c_api/c_api.cc:935: `ntree_limit` is deprecated, use `iteration_range` instead.
## [16:04:05] WARNING: src/c_api/c_api.cc:935: `ntree_limit` is deprecated, use `iteration_range` instead.
## [16:04:05] WARNING: src/c_api/c_api.cc:935: `ntree_limit` is deprecated, use `iteration_range` instead.
## [16:04:05] WARNING: src/c_api/c_api.cc:935: `ntree_limit` is deprecated, use `iteration_range` instead.
## [16:04:05] WARNING: src/c_api/c_api.cc:935: `ntree_limit` is deprecated, use `iteration_range` instead.
## [16:04:06] WARNING: src/c_api/c_api.cc:935: `ntree_limit` is deprecated, use `iteration_range` instead.
## [16:04:06] WARNING: src/c_api/c_api.cc:935: `ntree_limit` is deprecated, use `iteration_range` instead.
## [16:04:06] WARNING: src/c_api/c_api.cc:935: `ntree_limit` is deprecated, use `iteration_range` instead.
## [16:04:06] WARNING: src/c_api/c_api.cc:935: `ntree_limit` is deprecated, use `iteration_range` instead.
## [16:04:06] WARNING: src/c_api/c_api.cc:935: `ntree_limit` is deprecated, use `iteration_range` instead.
## [16:04:06] WARNING: src/c_api/c_api.cc:935: `ntree_limit` is deprecated, use `iteration_range` instead.
## [16:04:06] WARNING: src/c_api/c_api.cc:935: `ntree_limit` is deprecated, use `iteration_range` instead.
## [16:04:06] WARNING: src/c_api/c_api.cc:935: `ntree_limit` is deprecated, use `iteration_range` instead.
## [16:04:06] WARNING: src/c_api/c_api.cc:935: `ntree_limit` is deprecated, use `iteration_range` instead.
## [16:04:06] WARNING: src/c_api/c_api.cc:935: `ntree_limit` is deprecated, use `iteration_range` instead.
## [16:04:07] WARNING: src/c_api/c_api.cc:935: `ntree_limit` is deprecated, use `iteration_range` instead.
## [16:04:07] WARNING: src/c_api/c_api.cc:935: `ntree_limit` is deprecated, use `iteration_range` instead.
## [16:04:07] WARNING: src/c_api/c_api.cc:935: `ntree_limit` is deprecated, use `iteration_range` instead.
## [16:04:07] WARNING: src/c_api/c_api.cc:935: `ntree_limit` is deprecated, use `iteration_range` instead.
## [16:04:07] WARNING: src/c_api/c_api.cc:935: `ntree_limit` is deprecated, use `iteration_range` instead.
## [16:04:07] WARNING: src/c_api/c_api.cc:935: `ntree_limit` is deprecated, use `iteration_range` instead.
## [16:04:07] WARNING: src/c_api/c_api.cc:935: `ntree_limit` is deprecated, use `iteration_range` instead.
## [16:04:07] WARNING: src/c_api/c_api.cc:935: `ntree_limit` is deprecated, use `iteration_range` instead.
## [16:04:07] WARNING: src/c_api/c_api.cc:935: `ntree_limit` is deprecated, use `iteration_range` instead.
## [16:04:07] WARNING: src/c_api/c_api.cc:935: `ntree_limit` is deprecated, use `iteration_range` instead.
## [16:04:07] WARNING: src/c_api/c_api.cc:935: `ntree_limit` is deprecated, use `iteration_range` instead.
## [16:04:07] WARNING: src/c_api/c_api.cc:935: `ntree_limit` is deprecated, use `iteration_range` instead.
## [16:04:08] WARNING: src/c_api/c_api.cc:935: `ntree_limit` is deprecated, use `iteration_range` instead.
## [16:04:08] WARNING: src/c_api/c_api.cc:935: `ntree_limit` is deprecated, use `iteration_range` instead.
## [16:04:08] WARNING: src/c_api/c_api.cc:935: `ntree_limit` is deprecated, use `iteration_range` instead.
## [16:04:08] WARNING: src/c_api/c_api.cc:935: `ntree_limit` is deprecated, use `iteration_range` instead.
## [16:04:08] WARNING: src/c_api/c_api.cc:935: `ntree_limit` is deprecated, use `iteration_range` instead.
## [16:04:08] WARNING: src/c_api/c_api.cc:935: `ntree_limit` is deprecated, use `iteration_range` instead.
## [16:04:08] WARNING: src/c_api/c_api.cc:935: `ntree_limit` is deprecated, use `iteration_range` instead.
## [16:04:08] WARNING: src/c_api/c_api.cc:935: `ntree_limit` is deprecated, use `iteration_range` instead.
## [16:04:08] WARNING: src/c_api/c_api.cc:935: `ntree_limit` is deprecated, use `iteration_range` instead.
## [16:04:08] WARNING: src/c_api/c_api.cc:935: `ntree_limit` is deprecated, use `iteration_range` instead.
## [16:04:08] WARNING: src/c_api/c_api.cc:935: `ntree_limit` is deprecated, use `iteration_range` instead.
## [16:04:08] WARNING: src/c_api/c_api.cc:935: `ntree_limit` is deprecated, use `iteration_range` instead.
## [16:04:09] WARNING: src/c_api/c_api.cc:935: `ntree_limit` is deprecated, use `iteration_range` instead.
## [16:04:09] WARNING: src/c_api/c_api.cc:935: `ntree_limit` is deprecated, use `iteration_range` instead.
## [16:04:09] WARNING: src/c_api/c_api.cc:935: `ntree_limit` is deprecated, use `iteration_range` instead.
## [16:04:09] WARNING: src/c_api/c_api.cc:935: `ntree_limit` is deprecated, use `iteration_range` instead.
## [16:04:09] WARNING: src/c_api/c_api.cc:935: `ntree_limit` is deprecated, use `iteration_range` instead.
## [16:04:09] WARNING: src/c_api/c_api.cc:935: `ntree_limit` is deprecated, use `iteration_range` instead.
## [16:04:09] WARNING: src/c_api/c_api.cc:935: `ntree_limit` is deprecated, use `iteration_range` instead.
## [16:04:09] WARNING: src/c_api/c_api.cc:935: `ntree_limit` is deprecated, use `iteration_range` instead.
## [16:04:09] WARNING: src/c_api/c_api.cc:935: `ntree_limit` is deprecated, use `iteration_range` instead.
## [16:04:09] WARNING: src/c_api/c_api.cc:935: `ntree_limit` is deprecated, use `iteration_range` instead.
## [16:04:10] WARNING: src/c_api/c_api.cc:935: `ntree_limit` is deprecated, use `iteration_range` instead.
## [16:04:10] WARNING: src/c_api/c_api.cc:935: `ntree_limit` is deprecated, use `iteration_range` instead.
## [16:04:10] WARNING: src/c_api/c_api.cc:935: `ntree_limit` is deprecated, use `iteration_range` instead.
## [16:04:10] WARNING: src/c_api/c_api.cc:935: `ntree_limit` is deprecated, use `iteration_range` instead.
## [16:04:10] WARNING: src/c_api/c_api.cc:935: `ntree_limit` is deprecated, use `iteration_range` instead.
## [16:04:10] WARNING: src/c_api/c_api.cc:935: `ntree_limit` is deprecated, use `iteration_range` instead.
## [16:04:10] WARNING: src/c_api/c_api.cc:935: `ntree_limit` is deprecated, use `iteration_range` instead.
## [16:04:10] WARNING: src/c_api/c_api.cc:935: `ntree_limit` is deprecated, use `iteration_range` instead.
## [16:04:10] WARNING: src/c_api/c_api.cc:935: `ntree_limit` is deprecated, use `iteration_range` instead.
## [16:04:10] WARNING: src/c_api/c_api.cc:935: `ntree_limit` is deprecated, use `iteration_range` instead.
## [16:04:10] WARNING: src/c_api/c_api.cc:935: `ntree_limit` is deprecated, use `iteration_range` instead.
## [16:04:10] WARNING: src/c_api/c_api.cc:935: `ntree_limit` is deprecated, use `iteration_range` instead.
## [16:04:10] WARNING: src/c_api/c_api.cc:935: `ntree_limit` is deprecated, use `iteration_range` instead.
## [16:04:10] WARNING: src/c_api/c_api.cc:935: `ntree_limit` is deprecated, use `iteration_range` instead.
## [16:04:11] WARNING: src/c_api/c_api.cc:935: `ntree_limit` is deprecated, use `iteration_range` instead.
## [16:04:11] WARNING: src/c_api/c_api.cc:935: `ntree_limit` is deprecated, use `iteration_range` instead.
## [16:04:11] WARNING: src/c_api/c_api.cc:935: `ntree_limit` is deprecated, use `iteration_range` instead.
## [16:04:11] WARNING: src/c_api/c_api.cc:935: `ntree_limit` is deprecated, use `iteration_range` instead.
## [16:04:11] WARNING: src/c_api/c_api.cc:935: `ntree_limit` is deprecated, use `iteration_range` instead.
## [16:04:11] WARNING: src/c_api/c_api.cc:935: `ntree_limit` is deprecated, use `iteration_range` instead.
## [16:04:11] WARNING: src/c_api/c_api.cc:935: `ntree_limit` is deprecated, use `iteration_range` instead.
## [16:04:11] WARNING: src/c_api/c_api.cc:935: `ntree_limit` is deprecated, use `iteration_range` instead.
## [16:04:11] WARNING: src/c_api/c_api.cc:935: `ntree_limit` is deprecated, use `iteration_range` instead.
## [16:04:11] WARNING: src/c_api/c_api.cc:935: `ntree_limit` is deprecated, use `iteration_range` instead.
## [16:04:11] WARNING: src/c_api/c_api.cc:935: `ntree_limit` is deprecated, use `iteration_range` instead.
## [16:04:11] WARNING: src/c_api/c_api.cc:935: `ntree_limit` is deprecated, use `iteration_range` instead.
## [16:04:12] WARNING: src/c_api/c_api.cc:935: `ntree_limit` is deprecated, use `iteration_range` instead.
## [16:04:12] WARNING: src/c_api/c_api.cc:935: `ntree_limit` is deprecated, use `iteration_range` instead.
## [16:04:12] WARNING: src/c_api/c_api.cc:935: `ntree_limit` is deprecated, use `iteration_range` instead.
## [16:04:12] WARNING: src/c_api/c_api.cc:935: `ntree_limit` is deprecated, use `iteration_range` instead.
## [16:04:12] WARNING: src/c_api/c_api.cc:935: `ntree_limit` is deprecated, use `iteration_range` instead.
## [16:04:12] WARNING: src/c_api/c_api.cc:935: `ntree_limit` is deprecated, use `iteration_range` instead.
## [16:04:12] WARNING: src/c_api/c_api.cc:935: `ntree_limit` is deprecated, use `iteration_range` instead.
## [16:04:12] WARNING: src/c_api/c_api.cc:935: `ntree_limit` is deprecated, use `iteration_range` instead.
## [16:04:13] WARNING: src/c_api/c_api.cc:935: `ntree_limit` is deprecated, use `iteration_range` instead.
## [16:04:13] WARNING: src/c_api/c_api.cc:935: `ntree_limit` is deprecated, use `iteration_range` instead.
## [16:04:13] WARNING: src/c_api/c_api.cc:935: `ntree_limit` is deprecated, use `iteration_range` instead.
## [16:04:13] WARNING: src/c_api/c_api.cc:935: `ntree_limit` is deprecated, use `iteration_range` instead.
## [16:04:13] WARNING: src/c_api/c_api.cc:935: `ntree_limit` is deprecated, use `iteration_range` instead.
## [16:04:13] WARNING: src/c_api/c_api.cc:935: `ntree_limit` is deprecated, use `iteration_range` instead.
## [16:04:13] WARNING: src/c_api/c_api.cc:935: `ntree_limit` is deprecated, use `iteration_range` instead.
## [16:04:13] WARNING: src/c_api/c_api.cc:935: `ntree_limit` is deprecated, use `iteration_range` instead.
## [16:04:13] WARNING: src/c_api/c_api.cc:935: `ntree_limit` is deprecated, use `iteration_range` instead.
## [16:04:13] WARNING: src/c_api/c_api.cc:935: `ntree_limit` is deprecated, use `iteration_range` instead.
## [16:04:14] WARNING: src/c_api/c_api.cc:935: `ntree_limit` is deprecated, use `iteration_range` instead.
## [16:04:14] WARNING: src/c_api/c_api.cc:935: `ntree_limit` is deprecated, use `iteration_range` instead.
## [16:04:14] WARNING: src/c_api/c_api.cc:935: `ntree_limit` is deprecated, use `iteration_range` instead.
## [16:04:14] WARNING: src/c_api/c_api.cc:935: `ntree_limit` is deprecated, use `iteration_range` instead.
## [16:04:14] WARNING: src/c_api/c_api.cc:935: `ntree_limit` is deprecated, use `iteration_range` instead.
## [16:04:14] WARNING: src/c_api/c_api.cc:935: `ntree_limit` is deprecated, use `iteration_range` instead.
## [16:04:14] WARNING: src/c_api/c_api.cc:935: `ntree_limit` is deprecated, use `iteration_range` instead.
## [16:04:14] WARNING: src/c_api/c_api.cc:935: `ntree_limit` is deprecated, use `iteration_range` instead.
## [16:04:14] WARNING: src/c_api/c_api.cc:935: `ntree_limit` is deprecated, use `iteration_range` instead.
## [16:04:14] WARNING: src/c_api/c_api.cc:935: `ntree_limit` is deprecated, use `iteration_range` instead.
## [16:04:14] WARNING: src/c_api/c_api.cc:935: `ntree_limit` is deprecated, use `iteration_range` instead.
## [16:04:14] WARNING: src/c_api/c_api.cc:935: `ntree_limit` is deprecated, use `iteration_range` instead.
## [16:04:14] WARNING: src/c_api/c_api.cc:935: `ntree_limit` is deprecated, use `iteration_range` instead.
## [16:04:14] WARNING: src/c_api/c_api.cc:935: `ntree_limit` is deprecated, use `iteration_range` instead.
## [16:04:15] WARNING: src/c_api/c_api.cc:935: `ntree_limit` is deprecated, use `iteration_range` instead.
## [16:04:15] WARNING: src/c_api/c_api.cc:935: `ntree_limit` is deprecated, use `iteration_range` instead.
## [16:04:15] WARNING: src/c_api/c_api.cc:935: `ntree_limit` is deprecated, use `iteration_range` instead.
## [16:04:15] WARNING: src/c_api/c_api.cc:935: `ntree_limit` is deprecated, use `iteration_range` instead.
## [16:04:15] WARNING: src/c_api/c_api.cc:935: `ntree_limit` is deprecated, use `iteration_range` instead.
## [16:04:15] WARNING: src/c_api/c_api.cc:935: `ntree_limit` is deprecated, use `iteration_range` instead.
## [16:04:15] WARNING: src/c_api/c_api.cc:935: `ntree_limit` is deprecated, use `iteration_range` instead.
## [16:04:15] WARNING: src/c_api/c_api.cc:935: `ntree_limit` is deprecated, use `iteration_range` instead.
## [16:04:15] WARNING: src/c_api/c_api.cc:935: `ntree_limit` is deprecated, use `iteration_range` instead.
## [16:04:15] WARNING: src/c_api/c_api.cc:935: `ntree_limit` is deprecated, use `iteration_range` instead.
## [16:04:16] WARNING: src/c_api/c_api.cc:935: `ntree_limit` is deprecated, use `iteration_range` instead.
## [16:04:16] WARNING: src/c_api/c_api.cc:935: `ntree_limit` is deprecated, use `iteration_range` instead.
## [16:04:16] WARNING: src/c_api/c_api.cc:935: `ntree_limit` is deprecated, use `iteration_range` instead.
## [16:04:16] WARNING: src/c_api/c_api.cc:935: `ntree_limit` is deprecated, use `iteration_range` instead.
## [16:04:16] WARNING: src/c_api/c_api.cc:935: `ntree_limit` is deprecated, use `iteration_range` instead.
## [16:04:16] WARNING: src/c_api/c_api.cc:935: `ntree_limit` is deprecated, use `iteration_range` instead.
## [16:04:16] WARNING: src/c_api/c_api.cc:935: `ntree_limit` is deprecated, use `iteration_range` instead.
## [16:04:16] WARNING: src/c_api/c_api.cc:935: `ntree_limit` is deprecated, use `iteration_range` instead.
## [16:04:16] WARNING: src/c_api/c_api.cc:935: `ntree_limit` is deprecated, use `iteration_range` instead.
## [16:04:16] WARNING: src/c_api/c_api.cc:935: `ntree_limit` is deprecated, use `iteration_range` instead.
## [16:04:19] WARNING: src/c_api/c_api.cc:935: `ntree_limit` is deprecated, use `iteration_range` instead.
## [16:04:19] WARNING: src/c_api/c_api.cc:935: `ntree_limit` is deprecated, use `iteration_range` instead.
## [16:04:20] WARNING: src/c_api/c_api.cc:935: `ntree_limit` is deprecated, use `iteration_range` instead.
## [16:04:20] WARNING: src/c_api/c_api.cc:935: `ntree_limit` is deprecated, use `iteration_range` instead.
## [16:04:20] WARNING: src/c_api/c_api.cc:935: `ntree_limit` is deprecated, use `iteration_range` instead.
## [16:04:20] WARNING: src/c_api/c_api.cc:935: `ntree_limit` is deprecated, use `iteration_range` instead.
## [16:04:20] WARNING: src/c_api/c_api.cc:935: `ntree_limit` is deprecated, use `iteration_range` instead.
## [16:04:20] WARNING: src/c_api/c_api.cc:935: `ntree_limit` is deprecated, use `iteration_range` instead.
## [16:04:20] WARNING: src/c_api/c_api.cc:935: `ntree_limit` is deprecated, use `iteration_range` instead.
## [16:04:20] WARNING: src/c_api/c_api.cc:935: `ntree_limit` is deprecated, use `iteration_range` instead.
## [16:04:20] WARNING: src/c_api/c_api.cc:935: `ntree_limit` is deprecated, use `iteration_range` instead.
## [16:04:20] WARNING: src/c_api/c_api.cc:935: `ntree_limit` is deprecated, use `iteration_range` instead.
## [16:04:20] WARNING: src/c_api/c_api.cc:935: `ntree_limit` is deprecated, use `iteration_range` instead.
## [16:04:20] WARNING: src/c_api/c_api.cc:935: `ntree_limit` is deprecated, use `iteration_range` instead.
## [16:04:20] WARNING: src/c_api/c_api.cc:935: `ntree_limit` is deprecated, use `iteration_range` instead.
## [16:04:20] WARNING: src/c_api/c_api.cc:935: `ntree_limit` is deprecated, use `iteration_range` instead.
## [16:04:21] WARNING: src/c_api/c_api.cc:935: `ntree_limit` is deprecated, use `iteration_range` instead.
## [16:04:21] WARNING: src/c_api/c_api.cc:935: `ntree_limit` is deprecated, use `iteration_range` instead.
## [16:04:21] WARNING: src/c_api/c_api.cc:935: `ntree_limit` is deprecated, use `iteration_range` instead.
## [16:04:21] WARNING: src/c_api/c_api.cc:935: `ntree_limit` is deprecated, use `iteration_range` instead.
## [16:04:21] WARNING: src/c_api/c_api.cc:935: `ntree_limit` is deprecated, use `iteration_range` instead.
## [16:04:21] WARNING: src/c_api/c_api.cc:935: `ntree_limit` is deprecated, use `iteration_range` instead.
## [16:04:21] WARNING: src/c_api/c_api.cc:935: `ntree_limit` is deprecated, use `iteration_range` instead.
## [16:04:21] WARNING: src/c_api/c_api.cc:935: `ntree_limit` is deprecated, use `iteration_range` instead.
## [16:04:21] WARNING: src/c_api/c_api.cc:935: `ntree_limit` is deprecated, use `iteration_range` instead.
## [16:04:21] WARNING: src/c_api/c_api.cc:935: `ntree_limit` is deprecated, use `iteration_range` instead.
## [16:04:22] WARNING: src/c_api/c_api.cc:935: `ntree_limit` is deprecated, use `iteration_range` instead.
## [16:04:22] WARNING: src/c_api/c_api.cc:935: `ntree_limit` is deprecated, use `iteration_range` instead.
## [16:04:22] WARNING: src/c_api/c_api.cc:935: `ntree_limit` is deprecated, use `iteration_range` instead.
## [16:04:22] WARNING: src/c_api/c_api.cc:935: `ntree_limit` is deprecated, use `iteration_range` instead.
## [16:04:22] WARNING: src/c_api/c_api.cc:935: `ntree_limit` is deprecated, use `iteration_range` instead.
## [16:04:22] WARNING: src/c_api/c_api.cc:935: `ntree_limit` is deprecated, use `iteration_range` instead.
## [16:04:22] WARNING: src/c_api/c_api.cc:935: `ntree_limit` is deprecated, use `iteration_range` instead.
## [16:04:22] WARNING: src/c_api/c_api.cc:935: `ntree_limit` is deprecated, use `iteration_range` instead.
## [16:04:23] WARNING: src/c_api/c_api.cc:935: `ntree_limit` is deprecated, use `iteration_range` instead.
## [16:04:23] WARNING: src/c_api/c_api.cc:935: `ntree_limit` is deprecated, use `iteration_range` instead.
## [16:04:23] WARNING: src/c_api/c_api.cc:935: `ntree_limit` is deprecated, use `iteration_range` instead.
## [16:04:23] WARNING: src/c_api/c_api.cc:935: `ntree_limit` is deprecated, use `iteration_range` instead.
## [16:04:23] WARNING: src/c_api/c_api.cc:935: `ntree_limit` is deprecated, use `iteration_range` instead.
## [16:04:23] WARNING: src/c_api/c_api.cc:935: `ntree_limit` is deprecated, use `iteration_range` instead.
## [16:04:23] WARNING: src/c_api/c_api.cc:935: `ntree_limit` is deprecated, use `iteration_range` instead.
## [16:04:23] WARNING: src/c_api/c_api.cc:935: `ntree_limit` is deprecated, use `iteration_range` instead.
## [16:04:23] WARNING: src/c_api/c_api.cc:935: `ntree_limit` is deprecated, use `iteration_range` instead.
## [16:04:23] WARNING: src/c_api/c_api.cc:935: `ntree_limit` is deprecated, use `iteration_range` instead.
## [16:04:23] WARNING: src/c_api/c_api.cc:935: `ntree_limit` is deprecated, use `iteration_range` instead.
## [16:04:23] WARNING: src/c_api/c_api.cc:935: `ntree_limit` is deprecated, use `iteration_range` instead.
## [16:04:23] WARNING: src/c_api/c_api.cc:935: `ntree_limit` is deprecated, use `iteration_range` instead.
## [16:04:23] WARNING: src/c_api/c_api.cc:935: `ntree_limit` is deprecated, use `iteration_range` instead.
## [16:04:24] WARNING: src/c_api/c_api.cc:935: `ntree_limit` is deprecated, use `iteration_range` instead.
## [16:04:24] WARNING: src/c_api/c_api.cc:935: `ntree_limit` is deprecated, use `iteration_range` instead.
## [16:04:24] WARNING: src/c_api/c_api.cc:935: `ntree_limit` is deprecated, use `iteration_range` instead.
## [16:04:24] WARNING: src/c_api/c_api.cc:935: `ntree_limit` is deprecated, use `iteration_range` instead.
## [16:04:24] WARNING: src/c_api/c_api.cc:935: `ntree_limit` is deprecated, use `iteration_range` instead.
## [16:04:24] WARNING: src/c_api/c_api.cc:935: `ntree_limit` is deprecated, use `iteration_range` instead.
## [16:04:24] WARNING: src/c_api/c_api.cc:935: `ntree_limit` is deprecated, use `iteration_range` instead.
## [16:04:24] WARNING: src/c_api/c_api.cc:935: `ntree_limit` is deprecated, use `iteration_range` instead.
## [16:04:24] WARNING: src/c_api/c_api.cc:935: `ntree_limit` is deprecated, use `iteration_range` instead.
## [16:04:24] WARNING: src/c_api/c_api.cc:935: `ntree_limit` is deprecated, use `iteration_range` instead.
## [16:04:24] WARNING: src/c_api/c_api.cc:935: `ntree_limit` is deprecated, use `iteration_range` instead.
## [16:04:24] WARNING: src/c_api/c_api.cc:935: `ntree_limit` is deprecated, use `iteration_range` instead.
## [16:04:25] WARNING: src/c_api/c_api.cc:935: `ntree_limit` is deprecated, use `iteration_range` instead.
## [16:04:25] WARNING: src/c_api/c_api.cc:935: `ntree_limit` is deprecated, use `iteration_range` instead.
## [16:04:25] WARNING: src/c_api/c_api.cc:935: `ntree_limit` is deprecated, use `iteration_range` instead.
## [16:04:25] WARNING: src/c_api/c_api.cc:935: `ntree_limit` is deprecated, use `iteration_range` instead.
## [16:04:25] WARNING: src/c_api/c_api.cc:935: `ntree_limit` is deprecated, use `iteration_range` instead.
## [16:04:25] WARNING: src/c_api/c_api.cc:935: `ntree_limit` is deprecated, use `iteration_range` instead.
## [16:04:25] WARNING: src/c_api/c_api.cc:935: `ntree_limit` is deprecated, use `iteration_range` instead.
## [16:04:25] WARNING: src/c_api/c_api.cc:935: `ntree_limit` is deprecated, use `iteration_range` instead.
## [16:04:25] WARNING: src/c_api/c_api.cc:935: `ntree_limit` is deprecated, use `iteration_range` instead.
## [16:04:25] WARNING: src/c_api/c_api.cc:935: `ntree_limit` is deprecated, use `iteration_range` instead.
## [16:04:26] WARNING: src/c_api/c_api.cc:935: `ntree_limit` is deprecated, use `iteration_range` instead.
## [16:04:26] WARNING: src/c_api/c_api.cc:935: `ntree_limit` is deprecated, use `iteration_range` instead.
## [16:04:26] WARNING: src/c_api/c_api.cc:935: `ntree_limit` is deprecated, use `iteration_range` instead.
## [16:04:26] WARNING: src/c_api/c_api.cc:935: `ntree_limit` is deprecated, use `iteration_range` instead.
## [16:04:26] WARNING: src/c_api/c_api.cc:935: `ntree_limit` is deprecated, use `iteration_range` instead.
## [16:04:26] WARNING: src/c_api/c_api.cc:935: `ntree_limit` is deprecated, use `iteration_range` instead.
## [16:04:26] WARNING: src/c_api/c_api.cc:935: `ntree_limit` is deprecated, use `iteration_range` instead.
## [16:04:26] WARNING: src/c_api/c_api.cc:935: `ntree_limit` is deprecated, use `iteration_range` instead.
## [16:04:26] WARNING: src/c_api/c_api.cc:935: `ntree_limit` is deprecated, use `iteration_range` instead.
## [16:04:26] WARNING: src/c_api/c_api.cc:935: `ntree_limit` is deprecated, use `iteration_range` instead.
## [16:04:26] WARNING: src/c_api/c_api.cc:935: `ntree_limit` is deprecated, use `iteration_range` instead.
## [16:04:26] WARNING: src/c_api/c_api.cc:935: `ntree_limit` is deprecated, use `iteration_range` instead.
## [16:04:26] WARNING: src/c_api/c_api.cc:935: `ntree_limit` is deprecated, use `iteration_range` instead.
## [16:04:26] WARNING: src/c_api/c_api.cc:935: `ntree_limit` is deprecated, use `iteration_range` instead.
## [16:04:27] WARNING: src/c_api/c_api.cc:935: `ntree_limit` is deprecated, use `iteration_range` instead.
## [16:04:27] WARNING: src/c_api/c_api.cc:935: `ntree_limit` is deprecated, use `iteration_range` instead.
## [16:04:27] WARNING: src/c_api/c_api.cc:935: `ntree_limit` is deprecated, use `iteration_range` instead.
## [16:04:27] WARNING: src/c_api/c_api.cc:935: `ntree_limit` is deprecated, use `iteration_range` instead.
## [16:04:27] WARNING: src/c_api/c_api.cc:935: `ntree_limit` is deprecated, use `iteration_range` instead.
## [16:04:27] WARNING: src/c_api/c_api.cc:935: `ntree_limit` is deprecated, use `iteration_range` instead.
## [16:04:27] WARNING: src/c_api/c_api.cc:935: `ntree_limit` is deprecated, use `iteration_range` instead.
## [16:04:27] WARNING: src/c_api/c_api.cc:935: `ntree_limit` is deprecated, use `iteration_range` instead.
## [16:04:27] WARNING: src/c_api/c_api.cc:935: `ntree_limit` is deprecated, use `iteration_range` instead.
## [16:04:27] WARNING: src/c_api/c_api.cc:935: `ntree_limit` is deprecated, use `iteration_range` instead.
## [16:04:27] WARNING: src/c_api/c_api.cc:935: `ntree_limit` is deprecated, use `iteration_range` instead.
## [16:04:27] WARNING: src/c_api/c_api.cc:935: `ntree_limit` is deprecated, use `iteration_range` instead.
## [16:04:28] WARNING: src/c_api/c_api.cc:935: `ntree_limit` is deprecated, use `iteration_range` instead.
## [16:04:28] WARNING: src/c_api/c_api.cc:935: `ntree_limit` is deprecated, use `iteration_range` instead.
## [16:04:28] WARNING: src/c_api/c_api.cc:935: `ntree_limit` is deprecated, use `iteration_range` instead.
## [16:04:28] WARNING: src/c_api/c_api.cc:935: `ntree_limit` is deprecated, use `iteration_range` instead.
## [16:04:28] WARNING: src/c_api/c_api.cc:935: `ntree_limit` is deprecated, use `iteration_range` instead.
## [16:04:28] WARNING: src/c_api/c_api.cc:935: `ntree_limit` is deprecated, use `iteration_range` instead.
## [16:04:28] WARNING: src/c_api/c_api.cc:935: `ntree_limit` is deprecated, use `iteration_range` instead.
## [16:04:28] WARNING: src/c_api/c_api.cc:935: `ntree_limit` is deprecated, use `iteration_range` instead.
## [16:04:29] WARNING: src/c_api/c_api.cc:935: `ntree_limit` is deprecated, use `iteration_range` instead.
## [16:04:29] WARNING: src/c_api/c_api.cc:935: `ntree_limit` is deprecated, use `iteration_range` instead.
## [16:04:29] WARNING: src/c_api/c_api.cc:935: `ntree_limit` is deprecated, use `iteration_range` instead.
## [16:04:29] WARNING: src/c_api/c_api.cc:935: `ntree_limit` is deprecated, use `iteration_range` instead.
## [16:04:29] WARNING: src/c_api/c_api.cc:935: `ntree_limit` is deprecated, use `iteration_range` instead.
## [16:04:29] WARNING: src/c_api/c_api.cc:935: `ntree_limit` is deprecated, use `iteration_range` instead.
## [16:04:29] WARNING: src/c_api/c_api.cc:935: `ntree_limit` is deprecated, use `iteration_range` instead.
## [16:04:29] WARNING: src/c_api/c_api.cc:935: `ntree_limit` is deprecated, use `iteration_range` instead.
## [16:04:29] WARNING: src/c_api/c_api.cc:935: `ntree_limit` is deprecated, use `iteration_range` instead.
## [16:04:29] WARNING: src/c_api/c_api.cc:935: `ntree_limit` is deprecated, use `iteration_range` instead.
## [16:04:29] WARNING: src/c_api/c_api.cc:935: `ntree_limit` is deprecated, use `iteration_range` instead.
## [16:04:29] WARNING: src/c_api/c_api.cc:935: `ntree_limit` is deprecated, use `iteration_range` instead.
## [16:04:29] WARNING: src/c_api/c_api.cc:935: `ntree_limit` is deprecated, use `iteration_range` instead.
## [16:04:29] WARNING: src/c_api/c_api.cc:935: `ntree_limit` is deprecated, use `iteration_range` instead.
## [16:04:30] WARNING: src/c_api/c_api.cc:935: `ntree_limit` is deprecated, use `iteration_range` instead.
## [16:04:30] WARNING: src/c_api/c_api.cc:935: `ntree_limit` is deprecated, use `iteration_range` instead.
## [16:04:30] WARNING: src/c_api/c_api.cc:935: `ntree_limit` is deprecated, use `iteration_range` instead.
## [16:04:30] WARNING: src/c_api/c_api.cc:935: `ntree_limit` is deprecated, use `iteration_range` instead.
## [16:04:30] WARNING: src/c_api/c_api.cc:935: `ntree_limit` is deprecated, use `iteration_range` instead.
## [16:04:30] WARNING: src/c_api/c_api.cc:935: `ntree_limit` is deprecated, use `iteration_range` instead.
## [16:04:30] WARNING: src/c_api/c_api.cc:935: `ntree_limit` is deprecated, use `iteration_range` instead.
## [16:04:30] WARNING: src/c_api/c_api.cc:935: `ntree_limit` is deprecated, use `iteration_range` instead.
## [16:04:30] WARNING: src/c_api/c_api.cc:935: `ntree_limit` is deprecated, use `iteration_range` instead.
## [16:04:30] WARNING: src/c_api/c_api.cc:935: `ntree_limit` is deprecated, use `iteration_range` instead.
## [16:04:30] WARNING: src/c_api/c_api.cc:935: `ntree_limit` is deprecated, use `iteration_range` instead.
## [16:04:30] WARNING: src/c_api/c_api.cc:935: `ntree_limit` is deprecated, use `iteration_range` instead.
## [16:04:31] WARNING: src/c_api/c_api.cc:935: `ntree_limit` is deprecated, use `iteration_range` instead.
## [16:04:31] WARNING: src/c_api/c_api.cc:935: `ntree_limit` is deprecated, use `iteration_range` instead.
## [16:04:31] WARNING: src/c_api/c_api.cc:935: `ntree_limit` is deprecated, use `iteration_range` instead.
## [16:04:31] WARNING: src/c_api/c_api.cc:935: `ntree_limit` is deprecated, use `iteration_range` instead.
## [16:04:31] WARNING: src/c_api/c_api.cc:935: `ntree_limit` is deprecated, use `iteration_range` instead.
## [16:04:31] WARNING: src/c_api/c_api.cc:935: `ntree_limit` is deprecated, use `iteration_range` instead.
## [16:04:31] WARNING: src/c_api/c_api.cc:935: `ntree_limit` is deprecated, use `iteration_range` instead.
## [16:04:31] WARNING: src/c_api/c_api.cc:935: `ntree_limit` is deprecated, use `iteration_range` instead.
## [16:04:32] WARNING: src/c_api/c_api.cc:935: `ntree_limit` is deprecated, use `iteration_range` instead.
## [16:04:32] WARNING: src/c_api/c_api.cc:935: `ntree_limit` is deprecated, use `iteration_range` instead.
## [16:04:32] WARNING: src/c_api/c_api.cc:935: `ntree_limit` is deprecated, use `iteration_range` instead.
## [16:04:32] WARNING: src/c_api/c_api.cc:935: `ntree_limit` is deprecated, use `iteration_range` instead.
## [16:04:32] WARNING: src/c_api/c_api.cc:935: `ntree_limit` is deprecated, use `iteration_range` instead.
## [16:04:32] WARNING: src/c_api/c_api.cc:935: `ntree_limit` is deprecated, use `iteration_range` instead.
## [16:04:32] WARNING: src/c_api/c_api.cc:935: `ntree_limit` is deprecated, use `iteration_range` instead.
## [16:04:32] WARNING: src/c_api/c_api.cc:935: `ntree_limit` is deprecated, use `iteration_range` instead.
## [16:04:32] WARNING: src/c_api/c_api.cc:935: `ntree_limit` is deprecated, use `iteration_range` instead.
## [16:04:32] WARNING: src/c_api/c_api.cc:935: `ntree_limit` is deprecated, use `iteration_range` instead.
## [16:04:32] WARNING: src/c_api/c_api.cc:935: `ntree_limit` is deprecated, use `iteration_range` instead.
## [16:04:32] WARNING: src/c_api/c_api.cc:935: `ntree_limit` is deprecated, use `iteration_range` instead.
## [16:04:32] WARNING: src/c_api/c_api.cc:935: `ntree_limit` is deprecated, use `iteration_range` instead.
## [16:04:32] WARNING: src/c_api/c_api.cc:935: `ntree_limit` is deprecated, use `iteration_range` instead.
## [16:04:33] WARNING: src/c_api/c_api.cc:935: `ntree_limit` is deprecated, use `iteration_range` instead.
## [16:04:33] WARNING: src/c_api/c_api.cc:935: `ntree_limit` is deprecated, use `iteration_range` instead.
## [16:04:33] WARNING: src/c_api/c_api.cc:935: `ntree_limit` is deprecated, use `iteration_range` instead.
## [16:04:33] WARNING: src/c_api/c_api.cc:935: `ntree_limit` is deprecated, use `iteration_range` instead.
## [16:04:33] WARNING: src/c_api/c_api.cc:935: `ntree_limit` is deprecated, use `iteration_range` instead.
## [16:04:33] WARNING: src/c_api/c_api.cc:935: `ntree_limit` is deprecated, use `iteration_range` instead.
## [16:04:33] WARNING: src/c_api/c_api.cc:935: `ntree_limit` is deprecated, use `iteration_range` instead.
## [16:04:33] WARNING: src/c_api/c_api.cc:935: `ntree_limit` is deprecated, use `iteration_range` instead.
## [16:04:33] WARNING: src/c_api/c_api.cc:935: `ntree_limit` is deprecated, use `iteration_range` instead.
## [16:04:33] WARNING: src/c_api/c_api.cc:935: `ntree_limit` is deprecated, use `iteration_range` instead.
## [16:04:33] WARNING: src/c_api/c_api.cc:935: `ntree_limit` is deprecated, use `iteration_range` instead.
## [16:04:33] WARNING: src/c_api/c_api.cc:935: `ntree_limit` is deprecated, use `iteration_range` instead.
## [16:04:34] WARNING: src/c_api/c_api.cc:935: `ntree_limit` is deprecated, use `iteration_range` instead.
## [16:04:34] WARNING: src/c_api/c_api.cc:935: `ntree_limit` is deprecated, use `iteration_range` instead.
## [16:04:34] WARNING: src/c_api/c_api.cc:935: `ntree_limit` is deprecated, use `iteration_range` instead.
## [16:04:34] WARNING: src/c_api/c_api.cc:935: `ntree_limit` is deprecated, use `iteration_range` instead.
## [16:04:34] WARNING: src/c_api/c_api.cc:935: `ntree_limit` is deprecated, use `iteration_range` instead.
## [16:04:34] WARNING: src/c_api/c_api.cc:935: `ntree_limit` is deprecated, use `iteration_range` instead.
## [16:04:34] WARNING: src/c_api/c_api.cc:935: `ntree_limit` is deprecated, use `iteration_range` instead.
## [16:04:34] WARNING: src/c_api/c_api.cc:935: `ntree_limit` is deprecated, use `iteration_range` instead.
## [16:04:35] WARNING: src/c_api/c_api.cc:935: `ntree_limit` is deprecated, use `iteration_range` instead.
## [16:04:35] WARNING: src/c_api/c_api.cc:935: `ntree_limit` is deprecated, use `iteration_range` instead.
## [16:04:35] WARNING: src/c_api/c_api.cc:935: `ntree_limit` is deprecated, use `iteration_range` instead.
## [16:04:35] WARNING: src/c_api/c_api.cc:935: `ntree_limit` is deprecated, use `iteration_range` instead.
## [16:04:35] WARNING: src/c_api/c_api.cc:935: `ntree_limit` is deprecated, use `iteration_range` instead.
## [16:04:35] WARNING: src/c_api/c_api.cc:935: `ntree_limit` is deprecated, use `iteration_range` instead.
## [16:04:35] WARNING: src/c_api/c_api.cc:935: `ntree_limit` is deprecated, use `iteration_range` instead.
## [16:04:35] WARNING: src/c_api/c_api.cc:935: `ntree_limit` is deprecated, use `iteration_range` instead.
## [16:04:35] WARNING: src/c_api/c_api.cc:935: `ntree_limit` is deprecated, use `iteration_range` instead.
## [16:04:35] WARNING: src/c_api/c_api.cc:935: `ntree_limit` is deprecated, use `iteration_range` instead.
## [16:04:36] WARNING: src/c_api/c_api.cc:935: `ntree_limit` is deprecated, use `iteration_range` instead.
## [16:04:36] WARNING: src/c_api/c_api.cc:935: `ntree_limit` is deprecated, use `iteration_range` instead.
## [16:04:36] WARNING: src/c_api/c_api.cc:935: `ntree_limit` is deprecated, use `iteration_range` instead.
## [16:04:36] WARNING: src/c_api/c_api.cc:935: `ntree_limit` is deprecated, use `iteration_range` instead.
## [16:04:36] WARNING: src/c_api/c_api.cc:935: `ntree_limit` is deprecated, use `iteration_range` instead.
## [16:04:36] WARNING: src/c_api/c_api.cc:935: `ntree_limit` is deprecated, use `iteration_range` instead.
## [16:04:36] WARNING: src/c_api/c_api.cc:935: `ntree_limit` is deprecated, use `iteration_range` instead.
## [16:04:36] WARNING: src/c_api/c_api.cc:935: `ntree_limit` is deprecated, use `iteration_range` instead.
## [16:04:36] WARNING: src/c_api/c_api.cc:935: `ntree_limit` is deprecated, use `iteration_range` instead.
## [16:04:36] WARNING: src/c_api/c_api.cc:935: `ntree_limit` is deprecated, use `iteration_range` instead.
## [16:04:36] WARNING: src/c_api/c_api.cc:935: `ntree_limit` is deprecated, use `iteration_range` instead.
## [16:04:36] WARNING: src/c_api/c_api.cc:935: `ntree_limit` is deprecated, use `iteration_range` instead.
## [16:04:36] WARNING: src/c_api/c_api.cc:935: `ntree_limit` is deprecated, use `iteration_range` instead.
## [16:04:36] WARNING: src/c_api/c_api.cc:935: `ntree_limit` is deprecated, use `iteration_range` instead.
## [16:04:37] WARNING: src/c_api/c_api.cc:935: `ntree_limit` is deprecated, use `iteration_range` instead.
## [16:04:37] WARNING: src/c_api/c_api.cc:935: `ntree_limit` is deprecated, use `iteration_range` instead.
## [16:04:37] WARNING: src/c_api/c_api.cc:935: `ntree_limit` is deprecated, use `iteration_range` instead.
## [16:04:37] WARNING: src/c_api/c_api.cc:935: `ntree_limit` is deprecated, use `iteration_range` instead.
## [16:04:37] WARNING: src/c_api/c_api.cc:935: `ntree_limit` is deprecated, use `iteration_range` instead.
## [16:04:37] WARNING: src/c_api/c_api.cc:935: `ntree_limit` is deprecated, use `iteration_range` instead.
## [16:04:37] WARNING: src/c_api/c_api.cc:935: `ntree_limit` is deprecated, use `iteration_range` instead.
## [16:04:37] WARNING: src/c_api/c_api.cc:935: `ntree_limit` is deprecated, use `iteration_range` instead.
## [16:04:37] WARNING: src/c_api/c_api.cc:935: `ntree_limit` is deprecated, use `iteration_range` instead.
## [16:04:37] WARNING: src/c_api/c_api.cc:935: `ntree_limit` is deprecated, use `iteration_range` instead.
## [16:04:38] WARNING: src/c_api/c_api.cc:935: `ntree_limit` is deprecated, use `iteration_range` instead.
## [16:04:38] WARNING: src/c_api/c_api.cc:935: `ntree_limit` is deprecated, use `iteration_range` instead.
## [16:04:38] WARNING: src/c_api/c_api.cc:935: `ntree_limit` is deprecated, use `iteration_range` instead.
## [16:04:38] WARNING: src/c_api/c_api.cc:935: `ntree_limit` is deprecated, use `iteration_range` instead.
## [16:04:38] WARNING: src/c_api/c_api.cc:935: `ntree_limit` is deprecated, use `iteration_range` instead.
## [16:04:38] WARNING: src/c_api/c_api.cc:935: `ntree_limit` is deprecated, use `iteration_range` instead.
## [16:04:38] WARNING: src/c_api/c_api.cc:935: `ntree_limit` is deprecated, use `iteration_range` instead.
## [16:04:38] WARNING: src/c_api/c_api.cc:935: `ntree_limit` is deprecated, use `iteration_range` instead.
## [16:04:38] WARNING: src/c_api/c_api.cc:935: `ntree_limit` is deprecated, use `iteration_range` instead.
## [16:04:38] WARNING: src/c_api/c_api.cc:935: `ntree_limit` is deprecated, use `iteration_range` instead.
## [16:04:38] WARNING: src/c_api/c_api.cc:935: `ntree_limit` is deprecated, use `iteration_range` instead.
## [16:04:38] WARNING: src/c_api/c_api.cc:935: `ntree_limit` is deprecated, use `iteration_range` instead.
## [16:04:39] WARNING: src/c_api/c_api.cc:935: `ntree_limit` is deprecated, use `iteration_range` instead.
## [16:04:39] WARNING: src/c_api/c_api.cc:935: `ntree_limit` is deprecated, use `iteration_range` instead.
## [16:04:39] WARNING: src/c_api/c_api.cc:935: `ntree_limit` is deprecated, use `iteration_range` instead.
## [16:04:39] WARNING: src/c_api/c_api.cc:935: `ntree_limit` is deprecated, use `iteration_range` instead.
## [16:04:39] WARNING: src/c_api/c_api.cc:935: `ntree_limit` is deprecated, use `iteration_range` instead.
## [16:04:39] WARNING: src/c_api/c_api.cc:935: `ntree_limit` is deprecated, use `iteration_range` instead.
## [16:04:39] WARNING: src/c_api/c_api.cc:935: `ntree_limit` is deprecated, use `iteration_range` instead.
## [16:04:39] WARNING: src/c_api/c_api.cc:935: `ntree_limit` is deprecated, use `iteration_range` instead.
## [16:04:39] WARNING: src/c_api/c_api.cc:935: `ntree_limit` is deprecated, use `iteration_range` instead.
## [16:04:39] WARNING: src/c_api/c_api.cc:935: `ntree_limit` is deprecated, use `iteration_range` instead.
## [16:04:39] WARNING: src/c_api/c_api.cc:935: `ntree_limit` is deprecated, use `iteration_range` instead.
## [16:04:39] WARNING: src/c_api/c_api.cc:935: `ntree_limit` is deprecated, use `iteration_range` instead.
## [16:04:40] WARNING: src/c_api/c_api.cc:935: `ntree_limit` is deprecated, use `iteration_range` instead.
## [16:04:40] WARNING: src/c_api/c_api.cc:935: `ntree_limit` is deprecated, use `iteration_range` instead.
## [16:04:40] WARNING: src/c_api/c_api.cc:935: `ntree_limit` is deprecated, use `iteration_range` instead.
## [16:04:40] WARNING: src/c_api/c_api.cc:935: `ntree_limit` is deprecated, use `iteration_range` instead.
## [16:04:40] WARNING: src/c_api/c_api.cc:935: `ntree_limit` is deprecated, use `iteration_range` instead.
## [16:04:40] WARNING: src/c_api/c_api.cc:935: `ntree_limit` is deprecated, use `iteration_range` instead.
## [16:04:40] WARNING: src/c_api/c_api.cc:935: `ntree_limit` is deprecated, use `iteration_range` instead.
## [16:04:40] WARNING: src/c_api/c_api.cc:935: `ntree_limit` is deprecated, use `iteration_range` instead.
## [16:04:40] WARNING: src/c_api/c_api.cc:935: `ntree_limit` is deprecated, use `iteration_range` instead.
## [16:04:40] WARNING: src/c_api/c_api.cc:935: `ntree_limit` is deprecated, use `iteration_range` instead.
## [16:04:41] WARNING: src/c_api/c_api.cc:935: `ntree_limit` is deprecated, use `iteration_range` instead.
## [16:04:41] WARNING: src/c_api/c_api.cc:935: `ntree_limit` is deprecated, use `iteration_range` instead.
## [16:04:41] WARNING: src/c_api/c_api.cc:935: `ntree_limit` is deprecated, use `iteration_range` instead.
## [16:04:41] WARNING: src/c_api/c_api.cc:935: `ntree_limit` is deprecated, use `iteration_range` instead.
## [16:04:41] WARNING: src/c_api/c_api.cc:935: `ntree_limit` is deprecated, use `iteration_range` instead.
## [16:04:41] WARNING: src/c_api/c_api.cc:935: `ntree_limit` is deprecated, use `iteration_range` instead.
## [16:04:41] WARNING: src/c_api/c_api.cc:935: `ntree_limit` is deprecated, use `iteration_range` instead.
## [16:04:41] WARNING: src/c_api/c_api.cc:935: `ntree_limit` is deprecated, use `iteration_range` instead.
## [16:04:41] WARNING: src/c_api/c_api.cc:935: `ntree_limit` is deprecated, use `iteration_range` instead.
## [16:04:41] WARNING: src/c_api/c_api.cc:935: `ntree_limit` is deprecated, use `iteration_range` instead.
## [16:04:42] WARNING: src/c_api/c_api.cc:935: `ntree_limit` is deprecated, use `iteration_range` instead.
## [16:04:42] WARNING: src/c_api/c_api.cc:935: `ntree_limit` is deprecated, use `iteration_range` instead.
## [16:04:42] WARNING: src/c_api/c_api.cc:935: `ntree_limit` is deprecated, use `iteration_range` instead.
## [16:04:42] WARNING: src/c_api/c_api.cc:935: `ntree_limit` is deprecated, use `iteration_range` instead.
## [16:04:42] WARNING: src/c_api/c_api.cc:935: `ntree_limit` is deprecated, use `iteration_range` instead.
## [16:04:42] WARNING: src/c_api/c_api.cc:935: `ntree_limit` is deprecated, use `iteration_range` instead.
## [16:04:42] WARNING: src/c_api/c_api.cc:935: `ntree_limit` is deprecated, use `iteration_range` instead.
## [16:04:42] WARNING: src/c_api/c_api.cc:935: `ntree_limit` is deprecated, use `iteration_range` instead.
## [16:04:42] WARNING: src/c_api/c_api.cc:935: `ntree_limit` is deprecated, use `iteration_range` instead.
## [16:04:42] WARNING: src/c_api/c_api.cc:935: `ntree_limit` is deprecated, use `iteration_range` instead.
## [16:04:42] WARNING: src/c_api/c_api.cc:935: `ntree_limit` is deprecated, use `iteration_range` instead.
## [16:04:42] WARNING: src/c_api/c_api.cc:935: `ntree_limit` is deprecated, use `iteration_range` instead.
## [16:04:42] WARNING: src/c_api/c_api.cc:935: `ntree_limit` is deprecated, use `iteration_range` instead.
## [16:04:42] WARNING: src/c_api/c_api.cc:935: `ntree_limit` is deprecated, use `iteration_range` instead.
## [16:04:43] WARNING: src/c_api/c_api.cc:935: `ntree_limit` is deprecated, use `iteration_range` instead.
## [16:04:43] WARNING: src/c_api/c_api.cc:935: `ntree_limit` is deprecated, use `iteration_range` instead.
## [16:04:43] WARNING: src/c_api/c_api.cc:935: `ntree_limit` is deprecated, use `iteration_range` instead.
## [16:04:43] WARNING: src/c_api/c_api.cc:935: `ntree_limit` is deprecated, use `iteration_range` instead.
## [16:04:43] WARNING: src/c_api/c_api.cc:935: `ntree_limit` is deprecated, use `iteration_range` instead.
## [16:04:43] WARNING: src/c_api/c_api.cc:935: `ntree_limit` is deprecated, use `iteration_range` instead.
## [16:04:43] WARNING: src/c_api/c_api.cc:935: `ntree_limit` is deprecated, use `iteration_range` instead.
## [16:04:43] WARNING: src/c_api/c_api.cc:935: `ntree_limit` is deprecated, use `iteration_range` instead.
## [16:04:43] WARNING: src/c_api/c_api.cc:935: `ntree_limit` is deprecated, use `iteration_range` instead.
## [16:04:43] WARNING: src/c_api/c_api.cc:935: `ntree_limit` is deprecated, use `iteration_range` instead.
## [16:04:43] WARNING: src/c_api/c_api.cc:935: `ntree_limit` is deprecated, use `iteration_range` instead.
## [16:04:43] WARNING: src/c_api/c_api.cc:935: `ntree_limit` is deprecated, use `iteration_range` instead.
## [16:04:44] WARNING: src/c_api/c_api.cc:935: `ntree_limit` is deprecated, use `iteration_range` instead.
## [16:04:44] WARNING: src/c_api/c_api.cc:935: `ntree_limit` is deprecated, use `iteration_range` instead.
## [16:04:44] WARNING: src/c_api/c_api.cc:935: `ntree_limit` is deprecated, use `iteration_range` instead.
## [16:04:44] WARNING: src/c_api/c_api.cc:935: `ntree_limit` is deprecated, use `iteration_range` instead.
## [16:04:44] WARNING: src/c_api/c_api.cc:935: `ntree_limit` is deprecated, use `iteration_range` instead.
## [16:04:44] WARNING: src/c_api/c_api.cc:935: `ntree_limit` is deprecated, use `iteration_range` instead.
## [16:04:44] WARNING: src/c_api/c_api.cc:935: `ntree_limit` is deprecated, use `iteration_range` instead.
## [16:04:44] WARNING: src/c_api/c_api.cc:935: `ntree_limit` is deprecated, use `iteration_range` instead.
## [16:04:44] WARNING: src/c_api/c_api.cc:935: `ntree_limit` is deprecated, use `iteration_range` instead.
## [16:04:44] WARNING: src/c_api/c_api.cc:935: `ntree_limit` is deprecated, use `iteration_range` instead.
## [16:04:45] WARNING: src/c_api/c_api.cc:935: `ntree_limit` is deprecated, use `iteration_range` instead.
## [16:04:45] WARNING: src/c_api/c_api.cc:935: `ntree_limit` is deprecated, use `iteration_range` instead.
## [16:04:47] WARNING: src/c_api/c_api.cc:935: `ntree_limit` is deprecated, use `iteration_range` instead.
## [16:04:47] WARNING: src/c_api/c_api.cc:935: `ntree_limit` is deprecated, use `iteration_range` instead.
## [16:04:48] WARNING: src/c_api/c_api.cc:935: `ntree_limit` is deprecated, use `iteration_range` instead.
## [16:04:48] WARNING: src/c_api/c_api.cc:935: `ntree_limit` is deprecated, use `iteration_range` instead.
## [16:04:48] WARNING: src/c_api/c_api.cc:935: `ntree_limit` is deprecated, use `iteration_range` instead.
## [16:04:48] WARNING: src/c_api/c_api.cc:935: `ntree_limit` is deprecated, use `iteration_range` instead.
## [16:04:48] WARNING: src/c_api/c_api.cc:935: `ntree_limit` is deprecated, use `iteration_range` instead.
## [16:04:48] WARNING: src/c_api/c_api.cc:935: `ntree_limit` is deprecated, use `iteration_range` instead.
## [16:04:48] WARNING: src/c_api/c_api.cc:935: `ntree_limit` is deprecated, use `iteration_range` instead.
## [16:04:48] WARNING: src/c_api/c_api.cc:935: `ntree_limit` is deprecated, use `iteration_range` instead.
## [16:04:48] WARNING: src/c_api/c_api.cc:935: `ntree_limit` is deprecated, use `iteration_range` instead.
## [16:04:48] WARNING: src/c_api/c_api.cc:935: `ntree_limit` is deprecated, use `iteration_range` instead.
## [16:04:48] WARNING: src/c_api/c_api.cc:935: `ntree_limit` is deprecated, use `iteration_range` instead.
## [16:04:48] WARNING: src/c_api/c_api.cc:935: `ntree_limit` is deprecated, use `iteration_range` instead.
## [16:04:48] WARNING: src/c_api/c_api.cc:935: `ntree_limit` is deprecated, use `iteration_range` instead.
## [16:04:48] WARNING: src/c_api/c_api.cc:935: `ntree_limit` is deprecated, use `iteration_range` instead.
## [16:04:49] WARNING: src/c_api/c_api.cc:935: `ntree_limit` is deprecated, use `iteration_range` instead.
## [16:04:49] WARNING: src/c_api/c_api.cc:935: `ntree_limit` is deprecated, use `iteration_range` instead.
## [16:04:49] WARNING: src/c_api/c_api.cc:935: `ntree_limit` is deprecated, use `iteration_range` instead.
## [16:04:49] WARNING: src/c_api/c_api.cc:935: `ntree_limit` is deprecated, use `iteration_range` instead.
## [16:04:49] WARNING: src/c_api/c_api.cc:935: `ntree_limit` is deprecated, use `iteration_range` instead.
## [16:04:49] WARNING: src/c_api/c_api.cc:935: `ntree_limit` is deprecated, use `iteration_range` instead.
## [16:04:49] WARNING: src/c_api/c_api.cc:935: `ntree_limit` is deprecated, use `iteration_range` instead.
## [16:04:49] WARNING: src/c_api/c_api.cc:935: `ntree_limit` is deprecated, use `iteration_range` instead.
## [16:04:49] WARNING: src/c_api/c_api.cc:935: `ntree_limit` is deprecated, use `iteration_range` instead.
## [16:04:49] WARNING: src/c_api/c_api.cc:935: `ntree_limit` is deprecated, use `iteration_range` instead.
## [16:04:50] WARNING: src/c_api/c_api.cc:935: `ntree_limit` is deprecated, use `iteration_range` instead.
## [16:04:50] WARNING: src/c_api/c_api.cc:935: `ntree_limit` is deprecated, use `iteration_range` instead.
## [16:04:50] WARNING: src/c_api/c_api.cc:935: `ntree_limit` is deprecated, use `iteration_range` instead.
## [16:04:50] WARNING: src/c_api/c_api.cc:935: `ntree_limit` is deprecated, use `iteration_range` instead.
## [16:04:50] WARNING: src/c_api/c_api.cc:935: `ntree_limit` is deprecated, use `iteration_range` instead.
## [16:04:50] WARNING: src/c_api/c_api.cc:935: `ntree_limit` is deprecated, use `iteration_range` instead.
## [16:04:50] WARNING: src/c_api/c_api.cc:935: `ntree_limit` is deprecated, use `iteration_range` instead.
## [16:04:50] WARNING: src/c_api/c_api.cc:935: `ntree_limit` is deprecated, use `iteration_range` instead.
## [16:04:51] WARNING: src/c_api/c_api.cc:935: `ntree_limit` is deprecated, use `iteration_range` instead.
## [16:04:51] WARNING: src/c_api/c_api.cc:935: `ntree_limit` is deprecated, use `iteration_range` instead.
## [16:04:51] WARNING: src/c_api/c_api.cc:935: `ntree_limit` is deprecated, use `iteration_range` instead.
## [16:04:51] WARNING: src/c_api/c_api.cc:935: `ntree_limit` is deprecated, use `iteration_range` instead.
## [16:04:51] WARNING: src/c_api/c_api.cc:935: `ntree_limit` is deprecated, use `iteration_range` instead.
## [16:04:51] WARNING: src/c_api/c_api.cc:935: `ntree_limit` is deprecated, use `iteration_range` instead.
## [16:04:51] WARNING: src/c_api/c_api.cc:935: `ntree_limit` is deprecated, use `iteration_range` instead.
## [16:04:51] WARNING: src/c_api/c_api.cc:935: `ntree_limit` is deprecated, use `iteration_range` instead.
## [16:04:51] WARNING: src/c_api/c_api.cc:935: `ntree_limit` is deprecated, use `iteration_range` instead.
## [16:04:51] WARNING: src/c_api/c_api.cc:935: `ntree_limit` is deprecated, use `iteration_range` instead.
## [16:04:51] WARNING: src/c_api/c_api.cc:935: `ntree_limit` is deprecated, use `iteration_range` instead.
## [16:04:51] WARNING: src/c_api/c_api.cc:935: `ntree_limit` is deprecated, use `iteration_range` instead.
## [16:04:52] WARNING: src/c_api/c_api.cc:935: `ntree_limit` is deprecated, use `iteration_range` instead.
## [16:04:52] WARNING: src/c_api/c_api.cc:935: `ntree_limit` is deprecated, use `iteration_range` instead.
## [16:04:52] WARNING: src/c_api/c_api.cc:935: `ntree_limit` is deprecated, use `iteration_range` instead.
## [16:04:52] WARNING: src/c_api/c_api.cc:935: `ntree_limit` is deprecated, use `iteration_range` instead.
## [16:04:52] WARNING: src/c_api/c_api.cc:935: `ntree_limit` is deprecated, use `iteration_range` instead.
## [16:04:52] WARNING: src/c_api/c_api.cc:935: `ntree_limit` is deprecated, use `iteration_range` instead.
## [16:04:52] WARNING: src/c_api/c_api.cc:935: `ntree_limit` is deprecated, use `iteration_range` instead.
## [16:04:52] WARNING: src/c_api/c_api.cc:935: `ntree_limit` is deprecated, use `iteration_range` instead.
## [16:04:52] WARNING: src/c_api/c_api.cc:935: `ntree_limit` is deprecated, use `iteration_range` instead.
## [16:04:52] WARNING: src/c_api/c_api.cc:935: `ntree_limit` is deprecated, use `iteration_range` instead.
## [16:04:52] WARNING: src/c_api/c_api.cc:935: `ntree_limit` is deprecated, use `iteration_range` instead.
## [16:04:52] WARNING: src/c_api/c_api.cc:935: `ntree_limit` is deprecated, use `iteration_range` instead.
## [16:04:53] WARNING: src/c_api/c_api.cc:935: `ntree_limit` is deprecated, use `iteration_range` instead.
## [16:04:53] WARNING: src/c_api/c_api.cc:935: `ntree_limit` is deprecated, use `iteration_range` instead.
## [16:04:53] WARNING: src/c_api/c_api.cc:935: `ntree_limit` is deprecated, use `iteration_range` instead.
## [16:04:53] WARNING: src/c_api/c_api.cc:935: `ntree_limit` is deprecated, use `iteration_range` instead.
## [16:04:53] WARNING: src/c_api/c_api.cc:935: `ntree_limit` is deprecated, use `iteration_range` instead.
## [16:04:53] WARNING: src/c_api/c_api.cc:935: `ntree_limit` is deprecated, use `iteration_range` instead.
## [16:04:53] WARNING: src/c_api/c_api.cc:935: `ntree_limit` is deprecated, use `iteration_range` instead.
## [16:04:53] WARNING: src/c_api/c_api.cc:935: `ntree_limit` is deprecated, use `iteration_range` instead.
## [16:04:53] WARNING: src/c_api/c_api.cc:935: `ntree_limit` is deprecated, use `iteration_range` instead.
## [16:04:53] WARNING: src/c_api/c_api.cc:935: `ntree_limit` is deprecated, use `iteration_range` instead.
## [16:04:54] WARNING: src/c_api/c_api.cc:935: `ntree_limit` is deprecated, use `iteration_range` instead.
## [16:04:54] WARNING: src/c_api/c_api.cc:935: `ntree_limit` is deprecated, use `iteration_range` instead.
## [16:04:54] WARNING: src/c_api/c_api.cc:935: `ntree_limit` is deprecated, use `iteration_range` instead.
## [16:04:54] WARNING: src/c_api/c_api.cc:935: `ntree_limit` is deprecated, use `iteration_range` instead.
## [16:04:54] WARNING: src/c_api/c_api.cc:935: `ntree_limit` is deprecated, use `iteration_range` instead.
## [16:04:54] WARNING: src/c_api/c_api.cc:935: `ntree_limit` is deprecated, use `iteration_range` instead.
## [16:04:54] WARNING: src/c_api/c_api.cc:935: `ntree_limit` is deprecated, use `iteration_range` instead.
## [16:04:54] WARNING: src/c_api/c_api.cc:935: `ntree_limit` is deprecated, use `iteration_range` instead.
## [16:04:54] WARNING: src/c_api/c_api.cc:935: `ntree_limit` is deprecated, use `iteration_range` instead.
## [16:04:54] WARNING: src/c_api/c_api.cc:935: `ntree_limit` is deprecated, use `iteration_range` instead.
## [16:04:54] WARNING: src/c_api/c_api.cc:935: `ntree_limit` is deprecated, use `iteration_range` instead.
## [16:04:54] WARNING: src/c_api/c_api.cc:935: `ntree_limit` is deprecated, use `iteration_range` instead.
## [16:04:55] WARNING: src/c_api/c_api.cc:935: `ntree_limit` is deprecated, use `iteration_range` instead.
## [16:04:55] WARNING: src/c_api/c_api.cc:935: `ntree_limit` is deprecated, use `iteration_range` instead.
## [16:04:55] WARNING: src/c_api/c_api.cc:935: `ntree_limit` is deprecated, use `iteration_range` instead.
## [16:04:55] WARNING: src/c_api/c_api.cc:935: `ntree_limit` is deprecated, use `iteration_range` instead.
## [16:04:55] WARNING: src/c_api/c_api.cc:935: `ntree_limit` is deprecated, use `iteration_range` instead.
## [16:04:55] WARNING: src/c_api/c_api.cc:935: `ntree_limit` is deprecated, use `iteration_range` instead.
## [16:04:55] WARNING: src/c_api/c_api.cc:935: `ntree_limit` is deprecated, use `iteration_range` instead.
## [16:04:55] WARNING: src/c_api/c_api.cc:935: `ntree_limit` is deprecated, use `iteration_range` instead.
## [16:04:55] WARNING: src/c_api/c_api.cc:935: `ntree_limit` is deprecated, use `iteration_range` instead.
## [16:04:55] WARNING: src/c_api/c_api.cc:935: `ntree_limit` is deprecated, use `iteration_range` instead.
## [16:04:55] WARNING: src/c_api/c_api.cc:935: `ntree_limit` is deprecated, use `iteration_range` instead.
## [16:04:55] WARNING: src/c_api/c_api.cc:935: `ntree_limit` is deprecated, use `iteration_range` instead.
## [16:04:56] WARNING: src/c_api/c_api.cc:935: `ntree_limit` is deprecated, use `iteration_range` instead.
## [16:04:56] WARNING: src/c_api/c_api.cc:935: `ntree_limit` is deprecated, use `iteration_range` instead.
## [16:04:56] WARNING: src/c_api/c_api.cc:935: `ntree_limit` is deprecated, use `iteration_range` instead.
## [16:04:56] WARNING: src/c_api/c_api.cc:935: `ntree_limit` is deprecated, use `iteration_range` instead.
## [16:04:56] WARNING: src/c_api/c_api.cc:935: `ntree_limit` is deprecated, use `iteration_range` instead.
## [16:04:56] WARNING: src/c_api/c_api.cc:935: `ntree_limit` is deprecated, use `iteration_range` instead.
## [16:04:56] WARNING: src/c_api/c_api.cc:935: `ntree_limit` is deprecated, use `iteration_range` instead.
## [16:04:56] WARNING: src/c_api/c_api.cc:935: `ntree_limit` is deprecated, use `iteration_range` instead.
## [16:04:57] WARNING: src/c_api/c_api.cc:935: `ntree_limit` is deprecated, use `iteration_range` instead.
## [16:04:57] WARNING: src/c_api/c_api.cc:935: `ntree_limit` is deprecated, use `iteration_range` instead.
## [16:04:57] WARNING: src/c_api/c_api.cc:935: `ntree_limit` is deprecated, use `iteration_range` instead.
## [16:04:57] WARNING: src/c_api/c_api.cc:935: `ntree_limit` is deprecated, use `iteration_range` instead.
## [16:04:57] WARNING: src/c_api/c_api.cc:935: `ntree_limit` is deprecated, use `iteration_range` instead.
## [16:04:57] WARNING: src/c_api/c_api.cc:935: `ntree_limit` is deprecated, use `iteration_range` instead.
## [16:04:57] WARNING: src/c_api/c_api.cc:935: `ntree_limit` is deprecated, use `iteration_range` instead.
## [16:04:57] WARNING: src/c_api/c_api.cc:935: `ntree_limit` is deprecated, use `iteration_range` instead.
## [16:04:57] WARNING: src/c_api/c_api.cc:935: `ntree_limit` is deprecated, use `iteration_range` instead.
## [16:04:57] WARNING: src/c_api/c_api.cc:935: `ntree_limit` is deprecated, use `iteration_range` instead.
## [16:04:58] WARNING: src/c_api/c_api.cc:935: `ntree_limit` is deprecated, use `iteration_range` instead.
## [16:04:58] WARNING: src/c_api/c_api.cc:935: `ntree_limit` is deprecated, use `iteration_range` instead.
## [16:04:58] WARNING: src/c_api/c_api.cc:935: `ntree_limit` is deprecated, use `iteration_range` instead.
## [16:04:58] WARNING: src/c_api/c_api.cc:935: `ntree_limit` is deprecated, use `iteration_range` instead.
## [16:04:58] WARNING: src/c_api/c_api.cc:935: `ntree_limit` is deprecated, use `iteration_range` instead.
## [16:04:58] WARNING: src/c_api/c_api.cc:935: `ntree_limit` is deprecated, use `iteration_range` instead.
## [16:04:58] WARNING: src/c_api/c_api.cc:935: `ntree_limit` is deprecated, use `iteration_range` instead.
## [16:04:58] WARNING: src/c_api/c_api.cc:935: `ntree_limit` is deprecated, use `iteration_range` instead.
## [16:04:58] WARNING: src/c_api/c_api.cc:935: `ntree_limit` is deprecated, use `iteration_range` instead.
## [16:04:58] WARNING: src/c_api/c_api.cc:935: `ntree_limit` is deprecated, use `iteration_range` instead.
## [16:04:58] WARNING: src/c_api/c_api.cc:935: `ntree_limit` is deprecated, use `iteration_range` instead.
## [16:04:58] WARNING: src/c_api/c_api.cc:935: `ntree_limit` is deprecated, use `iteration_range` instead.
## [16:04:58] WARNING: src/c_api/c_api.cc:935: `ntree_limit` is deprecated, use `iteration_range` instead.
## [16:04:58] WARNING: src/c_api/c_api.cc:935: `ntree_limit` is deprecated, use `iteration_range` instead.
## [16:04:59] WARNING: src/c_api/c_api.cc:935: `ntree_limit` is deprecated, use `iteration_range` instead.
## [16:04:59] WARNING: src/c_api/c_api.cc:935: `ntree_limit` is deprecated, use `iteration_range` instead.
## [16:04:59] WARNING: src/c_api/c_api.cc:935: `ntree_limit` is deprecated, use `iteration_range` instead.
## [16:04:59] WARNING: src/c_api/c_api.cc:935: `ntree_limit` is deprecated, use `iteration_range` instead.
## [16:04:59] WARNING: src/c_api/c_api.cc:935: `ntree_limit` is deprecated, use `iteration_range` instead.
## [16:04:59] WARNING: src/c_api/c_api.cc:935: `ntree_limit` is deprecated, use `iteration_range` instead.
## [16:04:59] WARNING: src/c_api/c_api.cc:935: `ntree_limit` is deprecated, use `iteration_range` instead.
## [16:04:59] WARNING: src/c_api/c_api.cc:935: `ntree_limit` is deprecated, use `iteration_range` instead.
## [16:05:00] WARNING: src/c_api/c_api.cc:935: `ntree_limit` is deprecated, use `iteration_range` instead.
## [16:05:00] WARNING: src/c_api/c_api.cc:935: `ntree_limit` is deprecated, use `iteration_range` instead.
## [16:05:00] WARNING: src/c_api/c_api.cc:935: `ntree_limit` is deprecated, use `iteration_range` instead.
## [16:05:00] WARNING: src/c_api/c_api.cc:935: `ntree_limit` is deprecated, use `iteration_range` instead.
## [16:05:00] WARNING: src/c_api/c_api.cc:935: `ntree_limit` is deprecated, use `iteration_range` instead.
## [16:05:00] WARNING: src/c_api/c_api.cc:935: `ntree_limit` is deprecated, use `iteration_range` instead.
## [16:05:00] WARNING: src/c_api/c_api.cc:935: `ntree_limit` is deprecated, use `iteration_range` instead.
## [16:05:00] WARNING: src/c_api/c_api.cc:935: `ntree_limit` is deprecated, use `iteration_range` instead.
## [16:05:00] WARNING: src/c_api/c_api.cc:935: `ntree_limit` is deprecated, use `iteration_range` instead.
## [16:05:00] WARNING: src/c_api/c_api.cc:935: `ntree_limit` is deprecated, use `iteration_range` instead.
## [16:05:01] WARNING: src/c_api/c_api.cc:935: `ntree_limit` is deprecated, use `iteration_range` instead.
## [16:05:01] WARNING: src/c_api/c_api.cc:935: `ntree_limit` is deprecated, use `iteration_range` instead.
## [16:05:01] WARNING: src/c_api/c_api.cc:935: `ntree_limit` is deprecated, use `iteration_range` instead.
## [16:05:01] WARNING: src/c_api/c_api.cc:935: `ntree_limit` is deprecated, use `iteration_range` instead.
## [16:05:01] WARNING: src/c_api/c_api.cc:935: `ntree_limit` is deprecated, use `iteration_range` instead.
## [16:05:01] WARNING: src/c_api/c_api.cc:935: `ntree_limit` is deprecated, use `iteration_range` instead.
## [16:05:01] WARNING: src/c_api/c_api.cc:935: `ntree_limit` is deprecated, use `iteration_range` instead.
## [16:05:01] WARNING: src/c_api/c_api.cc:935: `ntree_limit` is deprecated, use `iteration_range` instead.
## [16:05:01] WARNING: src/c_api/c_api.cc:935: `ntree_limit` is deprecated, use `iteration_range` instead.
## [16:05:01] WARNING: src/c_api/c_api.cc:935: `ntree_limit` is deprecated, use `iteration_range` instead.
## [16:05:01] WARNING: src/c_api/c_api.cc:935: `ntree_limit` is deprecated, use `iteration_range` instead.
## [16:05:01] WARNING: src/c_api/c_api.cc:935: `ntree_limit` is deprecated, use `iteration_range` instead.
## [16:05:01] WARNING: src/c_api/c_api.cc:935: `ntree_limit` is deprecated, use `iteration_range` instead.
## [16:05:01] WARNING: src/c_api/c_api.cc:935: `ntree_limit` is deprecated, use `iteration_range` instead.
## [16:05:02] WARNING: src/c_api/c_api.cc:935: `ntree_limit` is deprecated, use `iteration_range` instead.
## [16:05:02] WARNING: src/c_api/c_api.cc:935: `ntree_limit` is deprecated, use `iteration_range` instead.
## [16:05:02] WARNING: src/c_api/c_api.cc:935: `ntree_limit` is deprecated, use `iteration_range` instead.
## [16:05:02] WARNING: src/c_api/c_api.cc:935: `ntree_limit` is deprecated, use `iteration_range` instead.
## [16:05:02] WARNING: src/c_api/c_api.cc:935: `ntree_limit` is deprecated, use `iteration_range` instead.
## [16:05:02] WARNING: src/c_api/c_api.cc:935: `ntree_limit` is deprecated, use `iteration_range` instead.
## [16:05:02] WARNING: src/c_api/c_api.cc:935: `ntree_limit` is deprecated, use `iteration_range` instead.
## [16:05:02] WARNING: src/c_api/c_api.cc:935: `ntree_limit` is deprecated, use `iteration_range` instead.
## [16:05:02] WARNING: src/c_api/c_api.cc:935: `ntree_limit` is deprecated, use `iteration_range` instead.
## [16:05:02] WARNING: src/c_api/c_api.cc:935: `ntree_limit` is deprecated, use `iteration_range` instead.
## [16:05:03] WARNING: src/c_api/c_api.cc:935: `ntree_limit` is deprecated, use `iteration_range` instead.
## [16:05:03] WARNING: src/c_api/c_api.cc:935: `ntree_limit` is deprecated, use `iteration_range` instead.
## [16:05:03] WARNING: src/c_api/c_api.cc:935: `ntree_limit` is deprecated, use `iteration_range` instead.
## [16:05:03] WARNING: src/c_api/c_api.cc:935: `ntree_limit` is deprecated, use `iteration_range` instead.
## [16:05:03] WARNING: src/c_api/c_api.cc:935: `ntree_limit` is deprecated, use `iteration_range` instead.
## [16:05:03] WARNING: src/c_api/c_api.cc:935: `ntree_limit` is deprecated, use `iteration_range` instead.
## [16:05:03] WARNING: src/c_api/c_api.cc:935: `ntree_limit` is deprecated, use `iteration_range` instead.
## [16:05:03] WARNING: src/c_api/c_api.cc:935: `ntree_limit` is deprecated, use `iteration_range` instead.
## [16:05:04] WARNING: src/c_api/c_api.cc:935: `ntree_limit` is deprecated, use `iteration_range` instead.
## [16:05:04] WARNING: src/c_api/c_api.cc:935: `ntree_limit` is deprecated, use `iteration_range` instead.
## [16:05:04] WARNING: src/c_api/c_api.cc:935: `ntree_limit` is deprecated, use `iteration_range` instead.
## [16:05:04] WARNING: src/c_api/c_api.cc:935: `ntree_limit` is deprecated, use `iteration_range` instead.
## [16:05:04] WARNING: src/c_api/c_api.cc:935: `ntree_limit` is deprecated, use `iteration_range` instead.
## [16:05:04] WARNING: src/c_api/c_api.cc:935: `ntree_limit` is deprecated, use `iteration_range` instead.
## [16:05:04] WARNING: src/c_api/c_api.cc:935: `ntree_limit` is deprecated, use `iteration_range` instead.
## [16:05:04] WARNING: src/c_api/c_api.cc:935: `ntree_limit` is deprecated, use `iteration_range` instead.
## [16:05:04] WARNING: src/c_api/c_api.cc:935: `ntree_limit` is deprecated, use `iteration_range` instead.
## [16:05:04] WARNING: src/c_api/c_api.cc:935: `ntree_limit` is deprecated, use `iteration_range` instead.
## [16:05:04] WARNING: src/c_api/c_api.cc:935: `ntree_limit` is deprecated, use `iteration_range` instead.
## [16:05:04] WARNING: src/c_api/c_api.cc:935: `ntree_limit` is deprecated, use `iteration_range` instead.
## [16:05:05] WARNING: src/c_api/c_api.cc:935: `ntree_limit` is deprecated, use `iteration_range` instead.
## [16:05:05] WARNING: src/c_api/c_api.cc:935: `ntree_limit` is deprecated, use `iteration_range` instead.
## [16:05:05] WARNING: src/c_api/c_api.cc:935: `ntree_limit` is deprecated, use `iteration_range` instead.
## [16:05:05] WARNING: src/c_api/c_api.cc:935: `ntree_limit` is deprecated, use `iteration_range` instead.
## [16:05:05] WARNING: src/c_api/c_api.cc:935: `ntree_limit` is deprecated, use `iteration_range` instead.
## [16:05:05] WARNING: src/c_api/c_api.cc:935: `ntree_limit` is deprecated, use `iteration_range` instead.
## [16:05:05] WARNING: src/c_api/c_api.cc:935: `ntree_limit` is deprecated, use `iteration_range` instead.
## [16:05:05] WARNING: src/c_api/c_api.cc:935: `ntree_limit` is deprecated, use `iteration_range` instead.
## [16:05:05] WARNING: src/c_api/c_api.cc:935: `ntree_limit` is deprecated, use `iteration_range` instead.
## [16:05:05] WARNING: src/c_api/c_api.cc:935: `ntree_limit` is deprecated, use `iteration_range` instead.
## [16:05:05] WARNING: src/c_api/c_api.cc:935: `ntree_limit` is deprecated, use `iteration_range` instead.
## [16:05:05] WARNING: src/c_api/c_api.cc:935: `ntree_limit` is deprecated, use `iteration_range` instead.
## [16:05:06] WARNING: src/c_api/c_api.cc:935: `ntree_limit` is deprecated, use `iteration_range` instead.
## [16:05:06] WARNING: src/c_api/c_api.cc:935: `ntree_limit` is deprecated, use `iteration_range` instead.
## [16:05:06] WARNING: src/c_api/c_api.cc:935: `ntree_limit` is deprecated, use `iteration_range` instead.
## [16:05:06] WARNING: src/c_api/c_api.cc:935: `ntree_limit` is deprecated, use `iteration_range` instead.
## [16:05:06] WARNING: src/c_api/c_api.cc:935: `ntree_limit` is deprecated, use `iteration_range` instead.
## [16:05:06] WARNING: src/c_api/c_api.cc:935: `ntree_limit` is deprecated, use `iteration_range` instead.
## [16:05:06] WARNING: src/c_api/c_api.cc:935: `ntree_limit` is deprecated, use `iteration_range` instead.
## [16:05:06] WARNING: src/c_api/c_api.cc:935: `ntree_limit` is deprecated, use `iteration_range` instead.
## [16:05:06] WARNING: src/c_api/c_api.cc:935: `ntree_limit` is deprecated, use `iteration_range` instead.
## [16:05:06] WARNING: src/c_api/c_api.cc:935: `ntree_limit` is deprecated, use `iteration_range` instead.
## [16:05:07] WARNING: src/c_api/c_api.cc:935: `ntree_limit` is deprecated, use `iteration_range` instead.
## [16:05:07] WARNING: src/c_api/c_api.cc:935: `ntree_limit` is deprecated, use `iteration_range` instead.
## [16:05:07] WARNING: src/c_api/c_api.cc:935: `ntree_limit` is deprecated, use `iteration_range` instead.
## [16:05:07] WARNING: src/c_api/c_api.cc:935: `ntree_limit` is deprecated, use `iteration_range` instead.
## [16:05:07] WARNING: src/c_api/c_api.cc:935: `ntree_limit` is deprecated, use `iteration_range` instead.
## [16:05:07] WARNING: src/c_api/c_api.cc:935: `ntree_limit` is deprecated, use `iteration_range` instead.
## [16:05:07] WARNING: src/c_api/c_api.cc:935: `ntree_limit` is deprecated, use `iteration_range` instead.
## [16:05:07] WARNING: src/c_api/c_api.cc:935: `ntree_limit` is deprecated, use `iteration_range` instead.
## [16:05:07] WARNING: src/c_api/c_api.cc:935: `ntree_limit` is deprecated, use `iteration_range` instead.
## [16:05:07] WARNING: src/c_api/c_api.cc:935: `ntree_limit` is deprecated, use `iteration_range` instead.
## [16:05:08] WARNING: src/c_api/c_api.cc:935: `ntree_limit` is deprecated, use `iteration_range` instead.
## [16:05:08] WARNING: src/c_api/c_api.cc:935: `ntree_limit` is deprecated, use `iteration_range` instead.
## [16:05:08] WARNING: src/c_api/c_api.cc:935: `ntree_limit` is deprecated, use `iteration_range` instead.
## [16:05:08] WARNING: src/c_api/c_api.cc:935: `ntree_limit` is deprecated, use `iteration_range` instead.
## [16:05:08] WARNING: src/c_api/c_api.cc:935: `ntree_limit` is deprecated, use `iteration_range` instead.
## [16:05:08] WARNING: src/c_api/c_api.cc:935: `ntree_limit` is deprecated, use `iteration_range` instead.
## [16:05:08] WARNING: src/c_api/c_api.cc:935: `ntree_limit` is deprecated, use `iteration_range` instead.
## [16:05:08] WARNING: src/c_api/c_api.cc:935: `ntree_limit` is deprecated, use `iteration_range` instead.
## [16:05:08] WARNING: src/c_api/c_api.cc:935: `ntree_limit` is deprecated, use `iteration_range` instead.
## [16:05:08] WARNING: src/c_api/c_api.cc:935: `ntree_limit` is deprecated, use `iteration_range` instead.
## [16:05:08] WARNING: src/c_api/c_api.cc:935: `ntree_limit` is deprecated, use `iteration_range` instead.
## [16:05:08] WARNING: src/c_api/c_api.cc:935: `ntree_limit` is deprecated, use `iteration_range` instead.
## [16:05:08] WARNING: src/c_api/c_api.cc:935: `ntree_limit` is deprecated, use `iteration_range` instead.
## [16:05:08] WARNING: src/c_api/c_api.cc:935: `ntree_limit` is deprecated, use `iteration_range` instead.
## [16:05:09] WARNING: src/c_api/c_api.cc:935: `ntree_limit` is deprecated, use `iteration_range` instead.
## [16:05:09] WARNING: src/c_api/c_api.cc:935: `ntree_limit` is deprecated, use `iteration_range` instead.
## [16:05:09] WARNING: src/c_api/c_api.cc:935: `ntree_limit` is deprecated, use `iteration_range` instead.
## [16:05:09] WARNING: src/c_api/c_api.cc:935: `ntree_limit` is deprecated, use `iteration_range` instead.
## [16:05:09] WARNING: src/c_api/c_api.cc:935: `ntree_limit` is deprecated, use `iteration_range` instead.
## [16:05:09] WARNING: src/c_api/c_api.cc:935: `ntree_limit` is deprecated, use `iteration_range` instead.
## [16:05:09] WARNING: src/c_api/c_api.cc:935: `ntree_limit` is deprecated, use `iteration_range` instead.
## [16:05:09] WARNING: src/c_api/c_api.cc:935: `ntree_limit` is deprecated, use `iteration_range` instead.
## [16:05:09] WARNING: src/c_api/c_api.cc:935: `ntree_limit` is deprecated, use `iteration_range` instead.
## [16:05:09] WARNING: src/c_api/c_api.cc:935: `ntree_limit` is deprecated, use `iteration_range` instead.
## [16:05:10] WARNING: src/c_api/c_api.cc:935: `ntree_limit` is deprecated, use `iteration_range` instead.
## [16:05:10] WARNING: src/c_api/c_api.cc:935: `ntree_limit` is deprecated, use `iteration_range` instead.
## [16:05:10] WARNING: src/c_api/c_api.cc:935: `ntree_limit` is deprecated, use `iteration_range` instead.
## [16:05:10] WARNING: src/c_api/c_api.cc:935: `ntree_limit` is deprecated, use `iteration_range` instead.
## [16:05:10] WARNING: src/c_api/c_api.cc:935: `ntree_limit` is deprecated, use `iteration_range` instead.
## [16:05:10] WARNING: src/c_api/c_api.cc:935: `ntree_limit` is deprecated, use `iteration_range` instead.
## [16:05:10] WARNING: src/c_api/c_api.cc:935: `ntree_limit` is deprecated, use `iteration_range` instead.
## [16:05:10] WARNING: src/c_api/c_api.cc:935: `ntree_limit` is deprecated, use `iteration_range` instead.
## [16:05:11] WARNING: src/c_api/c_api.cc:935: `ntree_limit` is deprecated, use `iteration_range` instead.
## [16:05:11] WARNING: src/c_api/c_api.cc:935: `ntree_limit` is deprecated, use `iteration_range` instead.
## [16:05:11] WARNING: src/c_api/c_api.cc:935: `ntree_limit` is deprecated, use `iteration_range` instead.
## [16:05:11] WARNING: src/c_api/c_api.cc:935: `ntree_limit` is deprecated, use `iteration_range` instead.
## [16:05:11] WARNING: src/c_api/c_api.cc:935: `ntree_limit` is deprecated, use `iteration_range` instead.
## [16:05:11] WARNING: src/c_api/c_api.cc:935: `ntree_limit` is deprecated, use `iteration_range` instead.
## [16:05:11] WARNING: src/c_api/c_api.cc:935: `ntree_limit` is deprecated, use `iteration_range` instead.
## [16:05:11] WARNING: src/c_api/c_api.cc:935: `ntree_limit` is deprecated, use `iteration_range` instead.
## [16:05:11] WARNING: src/c_api/c_api.cc:935: `ntree_limit` is deprecated, use `iteration_range` instead.
## [16:05:11] WARNING: src/c_api/c_api.cc:935: `ntree_limit` is deprecated, use `iteration_range` instead.
## [16:05:11] WARNING: src/c_api/c_api.cc:935: `ntree_limit` is deprecated, use `iteration_range` instead.
## [16:05:11] WARNING: src/c_api/c_api.cc:935: `ntree_limit` is deprecated, use `iteration_range` instead.
## [16:05:11] WARNING: src/c_api/c_api.cc:935: `ntree_limit` is deprecated, use `iteration_range` instead.
## [16:05:11] WARNING: src/c_api/c_api.cc:935: `ntree_limit` is deprecated, use `iteration_range` instead.
## [16:05:12] WARNING: src/c_api/c_api.cc:935: `ntree_limit` is deprecated, use `iteration_range` instead.
## [16:05:12] WARNING: src/c_api/c_api.cc:935: `ntree_limit` is deprecated, use `iteration_range` instead.
## [16:05:12] WARNING: src/c_api/c_api.cc:935: `ntree_limit` is deprecated, use `iteration_range` instead.
## [16:05:12] WARNING: src/c_api/c_api.cc:935: `ntree_limit` is deprecated, use `iteration_range` instead.
## [16:05:12] WARNING: src/c_api/c_api.cc:935: `ntree_limit` is deprecated, use `iteration_range` instead.
## [16:05:12] WARNING: src/c_api/c_api.cc:935: `ntree_limit` is deprecated, use `iteration_range` instead.
## [16:05:12] WARNING: src/c_api/c_api.cc:935: `ntree_limit` is deprecated, use `iteration_range` instead.
## [16:05:12] WARNING: src/c_api/c_api.cc:935: `ntree_limit` is deprecated, use `iteration_range` instead.
## [16:05:12] WARNING: src/c_api/c_api.cc:935: `ntree_limit` is deprecated, use `iteration_range` instead.
## [16:05:12] WARNING: src/c_api/c_api.cc:935: `ntree_limit` is deprecated, use `iteration_range` instead.
## [16:05:13] WARNING: src/c_api/c_api.cc:935: `ntree_limit` is deprecated, use `iteration_range` instead.
## [16:05:13] WARNING: src/c_api/c_api.cc:935: `ntree_limit` is deprecated, use `iteration_range` instead.
## [16:05:13] WARNING: src/c_api/c_api.cc:935: `ntree_limit` is deprecated, use `iteration_range` instead.
## [16:05:13] WARNING: src/c_api/c_api.cc:935: `ntree_limit` is deprecated, use `iteration_range` instead.
## [16:05:13] WARNING: src/c_api/c_api.cc:935: `ntree_limit` is deprecated, use `iteration_range` instead.
## [16:05:13] WARNING: src/c_api/c_api.cc:935: `ntree_limit` is deprecated, use `iteration_range` instead.
## [16:05:13] WARNING: src/c_api/c_api.cc:935: `ntree_limit` is deprecated, use `iteration_range` instead.
## [16:05:13] WARNING: src/c_api/c_api.cc:935: `ntree_limit` is deprecated, use `iteration_range` instead.
## [16:05:13] WARNING: src/c_api/c_api.cc:935: `ntree_limit` is deprecated, use `iteration_range` instead.
## [16:05:13] WARNING: src/c_api/c_api.cc:935: `ntree_limit` is deprecated, use `iteration_range` instead.
## [16:05:14] WARNING: src/c_api/c_api.cc:935: `ntree_limit` is deprecated, use `iteration_range` instead.
## [16:05:14] WARNING: src/c_api/c_api.cc:935: `ntree_limit` is deprecated, use `iteration_range` instead.
## [16:05:14] WARNING: src/c_api/c_api.cc:935: `ntree_limit` is deprecated, use `iteration_range` instead.
## [16:05:14] WARNING: src/c_api/c_api.cc:935: `ntree_limit` is deprecated, use `iteration_range` instead.
## [16:05:17] WARNING: src/c_api/c_api.cc:935: `ntree_limit` is deprecated, use `iteration_range` instead.
## [16:05:17] WARNING: src/c_api/c_api.cc:935: `ntree_limit` is deprecated, use `iteration_range` instead.
## [16:05:17] WARNING: src/c_api/c_api.cc:935: `ntree_limit` is deprecated, use `iteration_range` instead.
## [16:05:17] WARNING: src/c_api/c_api.cc:935: `ntree_limit` is deprecated, use `iteration_range` instead.
## [16:05:17] WARNING: src/c_api/c_api.cc:935: `ntree_limit` is deprecated, use `iteration_range` instead.
## [16:05:17] WARNING: src/c_api/c_api.cc:935: `ntree_limit` is deprecated, use `iteration_range` instead.
## [16:05:17] WARNING: src/c_api/c_api.cc:935: `ntree_limit` is deprecated, use `iteration_range` instead.
## [16:05:17] WARNING: src/c_api/c_api.cc:935: `ntree_limit` is deprecated, use `iteration_range` instead.
## [16:05:17] WARNING: src/c_api/c_api.cc:935: `ntree_limit` is deprecated, use `iteration_range` instead.
## [16:05:17] WARNING: src/c_api/c_api.cc:935: `ntree_limit` is deprecated, use `iteration_range` instead.
## [16:05:17] WARNING: src/c_api/c_api.cc:935: `ntree_limit` is deprecated, use `iteration_range` instead.
## [16:05:17] WARNING: src/c_api/c_api.cc:935: `ntree_limit` is deprecated, use `iteration_range` instead.
## [16:05:18] WARNING: src/c_api/c_api.cc:935: `ntree_limit` is deprecated, use `iteration_range` instead.
## [16:05:18] WARNING: src/c_api/c_api.cc:935: `ntree_limit` is deprecated, use `iteration_range` instead.
## [16:05:18] WARNING: src/c_api/c_api.cc:935: `ntree_limit` is deprecated, use `iteration_range` instead.
## [16:05:18] WARNING: src/c_api/c_api.cc:935: `ntree_limit` is deprecated, use `iteration_range` instead.
## [16:05:18] WARNING: src/c_api/c_api.cc:935: `ntree_limit` is deprecated, use `iteration_range` instead.
## [16:05:18] WARNING: src/c_api/c_api.cc:935: `ntree_limit` is deprecated, use `iteration_range` instead.
## [16:05:18] WARNING: src/c_api/c_api.cc:935: `ntree_limit` is deprecated, use `iteration_range` instead.
## [16:05:18] WARNING: src/c_api/c_api.cc:935: `ntree_limit` is deprecated, use `iteration_range` instead.
## [16:05:18] WARNING: src/c_api/c_api.cc:935: `ntree_limit` is deprecated, use `iteration_range` instead.
## [16:05:18] WARNING: src/c_api/c_api.cc:935: `ntree_limit` is deprecated, use `iteration_range` instead.
## [16:05:19] WARNING: src/c_api/c_api.cc:935: `ntree_limit` is deprecated, use `iteration_range` instead.
## [16:05:19] WARNING: src/c_api/c_api.cc:935: `ntree_limit` is deprecated, use `iteration_range` instead.
## [16:05:19] WARNING: src/c_api/c_api.cc:935: `ntree_limit` is deprecated, use `iteration_range` instead.
## [16:05:19] WARNING: src/c_api/c_api.cc:935: `ntree_limit` is deprecated, use `iteration_range` instead.
## [16:05:19] WARNING: src/c_api/c_api.cc:935: `ntree_limit` is deprecated, use `iteration_range` instead.
## [16:05:19] WARNING: src/c_api/c_api.cc:935: `ntree_limit` is deprecated, use `iteration_range` instead.
## [16:05:19] WARNING: src/c_api/c_api.cc:935: `ntree_limit` is deprecated, use `iteration_range` instead.
## [16:05:19] WARNING: src/c_api/c_api.cc:935: `ntree_limit` is deprecated, use `iteration_range` instead.
## [16:05:20] WARNING: src/c_api/c_api.cc:935: `ntree_limit` is deprecated, use `iteration_range` instead.
## [16:05:20] WARNING: src/c_api/c_api.cc:935: `ntree_limit` is deprecated, use `iteration_range` instead.
## [16:05:20] WARNING: src/c_api/c_api.cc:935: `ntree_limit` is deprecated, use `iteration_range` instead.
## [16:05:20] WARNING: src/c_api/c_api.cc:935: `ntree_limit` is deprecated, use `iteration_range` instead.
## [16:05:20] WARNING: src/c_api/c_api.cc:935: `ntree_limit` is deprecated, use `iteration_range` instead.
## [16:05:20] WARNING: src/c_api/c_api.cc:935: `ntree_limit` is deprecated, use `iteration_range` instead.
## [16:05:20] WARNING: src/c_api/c_api.cc:935: `ntree_limit` is deprecated, use `iteration_range` instead.
## [16:05:20] WARNING: src/c_api/c_api.cc:935: `ntree_limit` is deprecated, use `iteration_range` instead.
## [16:05:20] WARNING: src/c_api/c_api.cc:935: `ntree_limit` is deprecated, use `iteration_range` instead.
## [16:05:20] WARNING: src/c_api/c_api.cc:935: `ntree_limit` is deprecated, use `iteration_range` instead.
## [16:05:20] WARNING: src/c_api/c_api.cc:935: `ntree_limit` is deprecated, use `iteration_range` instead.
## [16:05:20] WARNING: src/c_api/c_api.cc:935: `ntree_limit` is deprecated, use `iteration_range` instead.
## [16:05:20] WARNING: src/c_api/c_api.cc:935: `ntree_limit` is deprecated, use `iteration_range` instead.
## [16:05:20] WARNING: src/c_api/c_api.cc:935: `ntree_limit` is deprecated, use `iteration_range` instead.
## [16:05:21] WARNING: src/c_api/c_api.cc:935: `ntree_limit` is deprecated, use `iteration_range` instead.
## [16:05:21] WARNING: src/c_api/c_api.cc:935: `ntree_limit` is deprecated, use `iteration_range` instead.
## [16:05:21] WARNING: src/c_api/c_api.cc:935: `ntree_limit` is deprecated, use `iteration_range` instead.
## [16:05:21] WARNING: src/c_api/c_api.cc:935: `ntree_limit` is deprecated, use `iteration_range` instead.
## [16:05:21] WARNING: src/c_api/c_api.cc:935: `ntree_limit` is deprecated, use `iteration_range` instead.
## [16:05:21] WARNING: src/c_api/c_api.cc:935: `ntree_limit` is deprecated, use `iteration_range` instead.
## [16:05:21] WARNING: src/c_api/c_api.cc:935: `ntree_limit` is deprecated, use `iteration_range` instead.
## [16:05:21] WARNING: src/c_api/c_api.cc:935: `ntree_limit` is deprecated, use `iteration_range` instead.
## [16:05:21] WARNING: src/c_api/c_api.cc:935: `ntree_limit` is deprecated, use `iteration_range` instead.
## [16:05:21] WARNING: src/c_api/c_api.cc:935: `ntree_limit` is deprecated, use `iteration_range` instead.
## [16:05:22] WARNING: src/c_api/c_api.cc:935: `ntree_limit` is deprecated, use `iteration_range` instead.
## [16:05:22] WARNING: src/c_api/c_api.cc:935: `ntree_limit` is deprecated, use `iteration_range` instead.
## [16:05:22] WARNING: src/c_api/c_api.cc:935: `ntree_limit` is deprecated, use `iteration_range` instead.
## [16:05:22] WARNING: src/c_api/c_api.cc:935: `ntree_limit` is deprecated, use `iteration_range` instead.
## [16:05:22] WARNING: src/c_api/c_api.cc:935: `ntree_limit` is deprecated, use `iteration_range` instead.
## [16:05:22] WARNING: src/c_api/c_api.cc:935: `ntree_limit` is deprecated, use `iteration_range` instead.
## [16:05:22] WARNING: src/c_api/c_api.cc:935: `ntree_limit` is deprecated, use `iteration_range` instead.
## [16:05:22] WARNING: src/c_api/c_api.cc:935: `ntree_limit` is deprecated, use `iteration_range` instead.
## [16:05:22] WARNING: src/c_api/c_api.cc:935: `ntree_limit` is deprecated, use `iteration_range` instead.
## [16:05:22] WARNING: src/c_api/c_api.cc:935: `ntree_limit` is deprecated, use `iteration_range` instead.
## [16:05:23] WARNING: src/c_api/c_api.cc:935: `ntree_limit` is deprecated, use `iteration_range` instead.
## [16:05:23] WARNING: src/c_api/c_api.cc:935: `ntree_limit` is deprecated, use `iteration_range` instead.
## [16:05:23] WARNING: src/c_api/c_api.cc:935: `ntree_limit` is deprecated, use `iteration_range` instead.
## [16:05:23] WARNING: src/c_api/c_api.cc:935: `ntree_limit` is deprecated, use `iteration_range` instead.
## [16:05:23] WARNING: src/c_api/c_api.cc:935: `ntree_limit` is deprecated, use `iteration_range` instead.
## [16:05:23] WARNING: src/c_api/c_api.cc:935: `ntree_limit` is deprecated, use `iteration_range` instead.
## [16:05:23] WARNING: src/c_api/c_api.cc:935: `ntree_limit` is deprecated, use `iteration_range` instead.
## [16:05:23] WARNING: src/c_api/c_api.cc:935: `ntree_limit` is deprecated, use `iteration_range` instead.
## [16:05:23] WARNING: src/c_api/c_api.cc:935: `ntree_limit` is deprecated, use `iteration_range` instead.
## [16:05:23] WARNING: src/c_api/c_api.cc:935: `ntree_limit` is deprecated, use `iteration_range` instead.
## [16:05:24] WARNING: src/c_api/c_api.cc:935: `ntree_limit` is deprecated, use `iteration_range` instead.
## [16:05:24] WARNING: src/c_api/c_api.cc:935: `ntree_limit` is deprecated, use `iteration_range` instead.
## [16:05:24] WARNING: src/c_api/c_api.cc:935: `ntree_limit` is deprecated, use `iteration_range` instead.
## [16:05:24] WARNING: src/c_api/c_api.cc:935: `ntree_limit` is deprecated, use `iteration_range` instead.
## [16:05:24] WARNING: src/c_api/c_api.cc:935: `ntree_limit` is deprecated, use `iteration_range` instead.
## [16:05:24] WARNING: src/c_api/c_api.cc:935: `ntree_limit` is deprecated, use `iteration_range` instead.
## [16:05:24] WARNING: src/c_api/c_api.cc:935: `ntree_limit` is deprecated, use `iteration_range` instead.
## [16:05:24] WARNING: src/c_api/c_api.cc:935: `ntree_limit` is deprecated, use `iteration_range` instead.
## [16:05:24] WARNING: src/c_api/c_api.cc:935: `ntree_limit` is deprecated, use `iteration_range` instead.
## [16:05:24] WARNING: src/c_api/c_api.cc:935: `ntree_limit` is deprecated, use `iteration_range` instead.
## [16:05:24] WARNING: src/c_api/c_api.cc:935: `ntree_limit` is deprecated, use `iteration_range` instead.
## [16:05:24] WARNING: src/c_api/c_api.cc:935: `ntree_limit` is deprecated, use `iteration_range` instead.
## [16:05:24] WARNING: src/c_api/c_api.cc:935: `ntree_limit` is deprecated, use `iteration_range` instead.
## [16:05:24] WARNING: src/c_api/c_api.cc:935: `ntree_limit` is deprecated, use `iteration_range` instead.
## [16:05:25] WARNING: src/c_api/c_api.cc:935: `ntree_limit` is deprecated, use `iteration_range` instead.
## [16:05:25] WARNING: src/c_api/c_api.cc:935: `ntree_limit` is deprecated, use `iteration_range` instead.
## [16:05:25] WARNING: src/c_api/c_api.cc:935: `ntree_limit` is deprecated, use `iteration_range` instead.
## [16:05:25] WARNING: src/c_api/c_api.cc:935: `ntree_limit` is deprecated, use `iteration_range` instead.
## [16:05:25] WARNING: src/c_api/c_api.cc:935: `ntree_limit` is deprecated, use `iteration_range` instead.
## [16:05:25] WARNING: src/c_api/c_api.cc:935: `ntree_limit` is deprecated, use `iteration_range` instead.
## [16:05:25] WARNING: src/c_api/c_api.cc:935: `ntree_limit` is deprecated, use `iteration_range` instead.
## [16:05:25] WARNING: src/c_api/c_api.cc:935: `ntree_limit` is deprecated, use `iteration_range` instead.
## [16:05:26] WARNING: src/c_api/c_api.cc:935: `ntree_limit` is deprecated, use `iteration_range` instead.
## [16:05:26] WARNING: src/c_api/c_api.cc:935: `ntree_limit` is deprecated, use `iteration_range` instead.
## [16:05:26] WARNING: src/c_api/c_api.cc:935: `ntree_limit` is deprecated, use `iteration_range` instead.
## [16:05:26] WARNING: src/c_api/c_api.cc:935: `ntree_limit` is deprecated, use `iteration_range` instead.
## [16:05:26] WARNING: src/c_api/c_api.cc:935: `ntree_limit` is deprecated, use `iteration_range` instead.
## [16:05:26] WARNING: src/c_api/c_api.cc:935: `ntree_limit` is deprecated, use `iteration_range` instead.
## [16:05:26] WARNING: src/c_api/c_api.cc:935: `ntree_limit` is deprecated, use `iteration_range` instead.
## [16:05:26] WARNING: src/c_api/c_api.cc:935: `ntree_limit` is deprecated, use `iteration_range` instead.
## [16:05:26] WARNING: src/c_api/c_api.cc:935: `ntree_limit` is deprecated, use `iteration_range` instead.
## [16:05:26] WARNING: src/c_api/c_api.cc:935: `ntree_limit` is deprecated, use `iteration_range` instead.
## [16:05:27] WARNING: src/c_api/c_api.cc:935: `ntree_limit` is deprecated, use `iteration_range` instead.
## [16:05:27] WARNING: src/c_api/c_api.cc:935: `ntree_limit` is deprecated, use `iteration_range` instead.
## [16:05:27] WARNING: src/c_api/c_api.cc:935: `ntree_limit` is deprecated, use `iteration_range` instead.
## [16:05:27] WARNING: src/c_api/c_api.cc:935: `ntree_limit` is deprecated, use `iteration_range` instead.
## [16:05:27] WARNING: src/c_api/c_api.cc:935: `ntree_limit` is deprecated, use `iteration_range` instead.
## [16:05:27] WARNING: src/c_api/c_api.cc:935: `ntree_limit` is deprecated, use `iteration_range` instead.
## [16:05:27] WARNING: src/c_api/c_api.cc:935: `ntree_limit` is deprecated, use `iteration_range` instead.
## [16:05:27] WARNING: src/c_api/c_api.cc:935: `ntree_limit` is deprecated, use `iteration_range` instead.
## [16:05:27] WARNING: src/c_api/c_api.cc:935: `ntree_limit` is deprecated, use `iteration_range` instead.
## [16:05:27] WARNING: src/c_api/c_api.cc:935: `ntree_limit` is deprecated, use `iteration_range` instead.
## [16:05:27] WARNING: src/c_api/c_api.cc:935: `ntree_limit` is deprecated, use `iteration_range` instead.
## [16:05:27] WARNING: src/c_api/c_api.cc:935: `ntree_limit` is deprecated, use `iteration_range` instead.
## [16:05:28] WARNING: src/c_api/c_api.cc:935: `ntree_limit` is deprecated, use `iteration_range` instead.
## [16:05:28] WARNING: src/c_api/c_api.cc:935: `ntree_limit` is deprecated, use `iteration_range` instead.
## [16:05:28] WARNING: src/c_api/c_api.cc:935: `ntree_limit` is deprecated, use `iteration_range` instead.
## [16:05:28] WARNING: src/c_api/c_api.cc:935: `ntree_limit` is deprecated, use `iteration_range` instead.
## [16:05:28] WARNING: src/c_api/c_api.cc:935: `ntree_limit` is deprecated, use `iteration_range` instead.
## [16:05:28] WARNING: src/c_api/c_api.cc:935: `ntree_limit` is deprecated, use `iteration_range` instead.
## [16:05:28] WARNING: src/c_api/c_api.cc:935: `ntree_limit` is deprecated, use `iteration_range` instead.
## [16:05:28] WARNING: src/c_api/c_api.cc:935: `ntree_limit` is deprecated, use `iteration_range` instead.
## [16:05:28] WARNING: src/c_api/c_api.cc:935: `ntree_limit` is deprecated, use `iteration_range` instead.
## [16:05:28] WARNING: src/c_api/c_api.cc:935: `ntree_limit` is deprecated, use `iteration_range` instead.
## [16:05:28] WARNING: src/c_api/c_api.cc:935: `ntree_limit` is deprecated, use `iteration_range` instead.
## [16:05:28] WARNING: src/c_api/c_api.cc:935: `ntree_limit` is deprecated, use `iteration_range` instead.
## [16:05:29] WARNING: src/c_api/c_api.cc:935: `ntree_limit` is deprecated, use `iteration_range` instead.
## [16:05:29] WARNING: src/c_api/c_api.cc:935: `ntree_limit` is deprecated, use `iteration_range` instead.
## [16:05:29] WARNING: src/c_api/c_api.cc:935: `ntree_limit` is deprecated, use `iteration_range` instead.
## [16:05:29] WARNING: src/c_api/c_api.cc:935: `ntree_limit` is deprecated, use `iteration_range` instead.
## [16:05:29] WARNING: src/c_api/c_api.cc:935: `ntree_limit` is deprecated, use `iteration_range` instead.
## [16:05:29] WARNING: src/c_api/c_api.cc:935: `ntree_limit` is deprecated, use `iteration_range` instead.
## [16:05:29] WARNING: src/c_api/c_api.cc:935: `ntree_limit` is deprecated, use `iteration_range` instead.
## [16:05:29] WARNING: src/c_api/c_api.cc:935: `ntree_limit` is deprecated, use `iteration_range` instead.
## [16:05:30] WARNING: src/c_api/c_api.cc:935: `ntree_limit` is deprecated, use `iteration_range` instead.
## [16:05:30] WARNING: src/c_api/c_api.cc:935: `ntree_limit` is deprecated, use `iteration_range` instead.
## [16:05:30] WARNING: src/c_api/c_api.cc:935: `ntree_limit` is deprecated, use `iteration_range` instead.
## [16:05:30] WARNING: src/c_api/c_api.cc:935: `ntree_limit` is deprecated, use `iteration_range` instead.
## [16:05:30] WARNING: src/c_api/c_api.cc:935: `ntree_limit` is deprecated, use `iteration_range` instead.
## [16:05:30] WARNING: src/c_api/c_api.cc:935: `ntree_limit` is deprecated, use `iteration_range` instead.
## [16:05:30] WARNING: src/c_api/c_api.cc:935: `ntree_limit` is deprecated, use `iteration_range` instead.
## [16:05:30] WARNING: src/c_api/c_api.cc:935: `ntree_limit` is deprecated, use `iteration_range` instead.
## [16:05:30] WARNING: src/c_api/c_api.cc:935: `ntree_limit` is deprecated, use `iteration_range` instead.
## [16:05:30] WARNING: src/c_api/c_api.cc:935: `ntree_limit` is deprecated, use `iteration_range` instead.
## [16:05:30] WARNING: src/c_api/c_api.cc:935: `ntree_limit` is deprecated, use `iteration_range` instead.
## [16:05:30] WARNING: src/c_api/c_api.cc:935: `ntree_limit` is deprecated, use `iteration_range` instead.
## [16:05:31] WARNING: src/c_api/c_api.cc:935: `ntree_limit` is deprecated, use `iteration_range` instead.
## [16:05:31] WARNING: src/c_api/c_api.cc:935: `ntree_limit` is deprecated, use `iteration_range` instead.
## [16:05:31] WARNING: src/c_api/c_api.cc:935: `ntree_limit` is deprecated, use `iteration_range` instead.
## [16:05:31] WARNING: src/c_api/c_api.cc:935: `ntree_limit` is deprecated, use `iteration_range` instead.
## [16:05:31] WARNING: src/c_api/c_api.cc:935: `ntree_limit` is deprecated, use `iteration_range` instead.
## [16:05:31] WARNING: src/c_api/c_api.cc:935: `ntree_limit` is deprecated, use `iteration_range` instead.
## [16:05:31] WARNING: src/c_api/c_api.cc:935: `ntree_limit` is deprecated, use `iteration_range` instead.
## [16:05:31] WARNING: src/c_api/c_api.cc:935: `ntree_limit` is deprecated, use `iteration_range` instead.
## [16:05:31] WARNING: src/c_api/c_api.cc:935: `ntree_limit` is deprecated, use `iteration_range` instead.
## [16:05:31] WARNING: src/c_api/c_api.cc:935: `ntree_limit` is deprecated, use `iteration_range` instead.
## [16:05:31] WARNING: src/c_api/c_api.cc:935: `ntree_limit` is deprecated, use `iteration_range` instead.
## [16:05:31] WARNING: src/c_api/c_api.cc:935: `ntree_limit` is deprecated, use `iteration_range` instead.
## [16:05:32] WARNING: src/c_api/c_api.cc:935: `ntree_limit` is deprecated, use `iteration_range` instead.
## [16:05:32] WARNING: src/c_api/c_api.cc:935: `ntree_limit` is deprecated, use `iteration_range` instead.
## [16:05:32] WARNING: src/c_api/c_api.cc:935: `ntree_limit` is deprecated, use `iteration_range` instead.
## [16:05:32] WARNING: src/c_api/c_api.cc:935: `ntree_limit` is deprecated, use `iteration_range` instead.
## [16:05:32] WARNING: src/c_api/c_api.cc:935: `ntree_limit` is deprecated, use `iteration_range` instead.
## [16:05:32] WARNING: src/c_api/c_api.cc:935: `ntree_limit` is deprecated, use `iteration_range` instead.
## [16:05:32] WARNING: src/c_api/c_api.cc:935: `ntree_limit` is deprecated, use `iteration_range` instead.
## [16:05:32] WARNING: src/c_api/c_api.cc:935: `ntree_limit` is deprecated, use `iteration_range` instead.
## [16:05:32] WARNING: src/c_api/c_api.cc:935: `ntree_limit` is deprecated, use `iteration_range` instead.
## [16:05:32] WARNING: src/c_api/c_api.cc:935: `ntree_limit` is deprecated, use `iteration_range` instead.
## [16:05:33] WARNING: src/c_api/c_api.cc:935: `ntree_limit` is deprecated, use `iteration_range` instead.
## [16:05:33] WARNING: src/c_api/c_api.cc:935: `ntree_limit` is deprecated, use `iteration_range` instead.
## [16:05:33] WARNING: src/c_api/c_api.cc:935: `ntree_limit` is deprecated, use `iteration_range` instead.
## [16:05:33] WARNING: src/c_api/c_api.cc:935: `ntree_limit` is deprecated, use `iteration_range` instead.
## [16:05:33] WARNING: src/c_api/c_api.cc:935: `ntree_limit` is deprecated, use `iteration_range` instead.
## [16:05:33] WARNING: src/c_api/c_api.cc:935: `ntree_limit` is deprecated, use `iteration_range` instead.
## [16:05:33] WARNING: src/c_api/c_api.cc:935: `ntree_limit` is deprecated, use `iteration_range` instead.
## [16:05:33] WARNING: src/c_api/c_api.cc:935: `ntree_limit` is deprecated, use `iteration_range` instead.
## [16:05:34] WARNING: src/c_api/c_api.cc:935: `ntree_limit` is deprecated, use `iteration_range` instead.
## [16:05:34] WARNING: src/c_api/c_api.cc:935: `ntree_limit` is deprecated, use `iteration_range` instead.
## [16:05:34] WARNING: src/c_api/c_api.cc:935: `ntree_limit` is deprecated, use `iteration_range` instead.
## [16:05:34] WARNING: src/c_api/c_api.cc:935: `ntree_limit` is deprecated, use `iteration_range` instead.
## [16:05:34] WARNING: src/c_api/c_api.cc:935: `ntree_limit` is deprecated, use `iteration_range` instead.
## [16:05:34] WARNING: src/c_api/c_api.cc:935: `ntree_limit` is deprecated, use `iteration_range` instead.
## [16:05:34] WARNING: src/c_api/c_api.cc:935: `ntree_limit` is deprecated, use `iteration_range` instead.
## [16:05:34] WARNING: src/c_api/c_api.cc:935: `ntree_limit` is deprecated, use `iteration_range` instead.
## [16:05:34] WARNING: src/c_api/c_api.cc:935: `ntree_limit` is deprecated, use `iteration_range` instead.
## [16:05:34] WARNING: src/c_api/c_api.cc:935: `ntree_limit` is deprecated, use `iteration_range` instead.
## [16:05:34] WARNING: src/c_api/c_api.cc:935: `ntree_limit` is deprecated, use `iteration_range` instead.
## [16:05:34] WARNING: src/c_api/c_api.cc:935: `ntree_limit` is deprecated, use `iteration_range` instead.
## [16:05:34] WARNING: src/c_api/c_api.cc:935: `ntree_limit` is deprecated, use `iteration_range` instead.
## [16:05:34] WARNING: src/c_api/c_api.cc:935: `ntree_limit` is deprecated, use `iteration_range` instead.
## [16:05:35] WARNING: src/c_api/c_api.cc:935: `ntree_limit` is deprecated, use `iteration_range` instead.
## [16:05:35] WARNING: src/c_api/c_api.cc:935: `ntree_limit` is deprecated, use `iteration_range` instead.
## [16:05:35] WARNING: src/c_api/c_api.cc:935: `ntree_limit` is deprecated, use `iteration_range` instead.
## [16:05:35] WARNING: src/c_api/c_api.cc:935: `ntree_limit` is deprecated, use `iteration_range` instead.
## [16:05:35] WARNING: src/c_api/c_api.cc:935: `ntree_limit` is deprecated, use `iteration_range` instead.
## [16:05:35] WARNING: src/c_api/c_api.cc:935: `ntree_limit` is deprecated, use `iteration_range` instead.
## [16:05:35] WARNING: src/c_api/c_api.cc:935: `ntree_limit` is deprecated, use `iteration_range` instead.
## [16:05:35] WARNING: src/c_api/c_api.cc:935: `ntree_limit` is deprecated, use `iteration_range` instead.
## [16:05:35] WARNING: src/c_api/c_api.cc:935: `ntree_limit` is deprecated, use `iteration_range` instead.
## [16:05:35] WARNING: src/c_api/c_api.cc:935: `ntree_limit` is deprecated, use `iteration_range` instead.
## [16:05:36] WARNING: src/c_api/c_api.cc:935: `ntree_limit` is deprecated, use `iteration_range` instead.
## [16:05:36] WARNING: src/c_api/c_api.cc:935: `ntree_limit` is deprecated, use `iteration_range` instead.
## [16:05:36] WARNING: src/c_api/c_api.cc:935: `ntree_limit` is deprecated, use `iteration_range` instead.
## [16:05:36] WARNING: src/c_api/c_api.cc:935: `ntree_limit` is deprecated, use `iteration_range` instead.
## [16:05:36] WARNING: src/c_api/c_api.cc:935: `ntree_limit` is deprecated, use `iteration_range` instead.
## [16:05:36] WARNING: src/c_api/c_api.cc:935: `ntree_limit` is deprecated, use `iteration_range` instead.
## [16:05:36] WARNING: src/c_api/c_api.cc:935: `ntree_limit` is deprecated, use `iteration_range` instead.
## [16:05:36] WARNING: src/c_api/c_api.cc:935: `ntree_limit` is deprecated, use `iteration_range` instead.
## [16:05:36] WARNING: src/c_api/c_api.cc:935: `ntree_limit` is deprecated, use `iteration_range` instead.
## [16:05:36] WARNING: src/c_api/c_api.cc:935: `ntree_limit` is deprecated, use `iteration_range` instead.
## [16:05:37] WARNING: src/c_api/c_api.cc:935: `ntree_limit` is deprecated, use `iteration_range` instead.
## [16:05:37] WARNING: src/c_api/c_api.cc:935: `ntree_limit` is deprecated, use `iteration_range` instead.
## [16:05:37] WARNING: src/c_api/c_api.cc:935: `ntree_limit` is deprecated, use `iteration_range` instead.
## [16:05:37] WARNING: src/c_api/c_api.cc:935: `ntree_limit` is deprecated, use `iteration_range` instead.
## [16:05:37] WARNING: src/c_api/c_api.cc:935: `ntree_limit` is deprecated, use `iteration_range` instead.
## [16:05:37] WARNING: src/c_api/c_api.cc:935: `ntree_limit` is deprecated, use `iteration_range` instead.
## [16:05:37] WARNING: src/c_api/c_api.cc:935: `ntree_limit` is deprecated, use `iteration_range` instead.
## [16:05:37] WARNING: src/c_api/c_api.cc:935: `ntree_limit` is deprecated, use `iteration_range` instead.
## [16:05:37] WARNING: src/c_api/c_api.cc:935: `ntree_limit` is deprecated, use `iteration_range` instead.
## [16:05:37] WARNING: src/c_api/c_api.cc:935: `ntree_limit` is deprecated, use `iteration_range` instead.
## [16:05:37] WARNING: src/c_api/c_api.cc:935: `ntree_limit` is deprecated, use `iteration_range` instead.
## [16:05:37] WARNING: src/c_api/c_api.cc:935: `ntree_limit` is deprecated, use `iteration_range` instead.
## [16:05:38] WARNING: src/c_api/c_api.cc:935: `ntree_limit` is deprecated, use `iteration_range` instead.
## [16:05:38] WARNING: src/c_api/c_api.cc:935: `ntree_limit` is deprecated, use `iteration_range` instead.
## [16:05:38] WARNING: src/c_api/c_api.cc:935: `ntree_limit` is deprecated, use `iteration_range` instead.
## [16:05:38] WARNING: src/c_api/c_api.cc:935: `ntree_limit` is deprecated, use `iteration_range` instead.
## [16:05:38] WARNING: src/c_api/c_api.cc:935: `ntree_limit` is deprecated, use `iteration_range` instead.
## [16:05:38] WARNING: src/c_api/c_api.cc:935: `ntree_limit` is deprecated, use `iteration_range` instead.
## [16:05:38] WARNING: src/c_api/c_api.cc:935: `ntree_limit` is deprecated, use `iteration_range` instead.
## [16:05:38] WARNING: src/c_api/c_api.cc:935: `ntree_limit` is deprecated, use `iteration_range` instead.
## [16:05:38] WARNING: src/c_api/c_api.cc:935: `ntree_limit` is deprecated, use `iteration_range` instead.
## [16:05:38] WARNING: src/c_api/c_api.cc:935: `ntree_limit` is deprecated, use `iteration_range` instead.
## [16:05:39] WARNING: src/c_api/c_api.cc:935: `ntree_limit` is deprecated, use `iteration_range` instead.
## [16:05:39] WARNING: src/c_api/c_api.cc:935: `ntree_limit` is deprecated, use `iteration_range` instead.
## [16:05:39] WARNING: src/c_api/c_api.cc:935: `ntree_limit` is deprecated, use `iteration_range` instead.
## [16:05:39] WARNING: src/c_api/c_api.cc:935: `ntree_limit` is deprecated, use `iteration_range` instead.
## [16:05:39] WARNING: src/c_api/c_api.cc:935: `ntree_limit` is deprecated, use `iteration_range` instead.
## [16:05:39] WARNING: src/c_api/c_api.cc:935: `ntree_limit` is deprecated, use `iteration_range` instead.
## [16:05:39] WARNING: src/c_api/c_api.cc:935: `ntree_limit` is deprecated, use `iteration_range` instead.
## [16:05:39] WARNING: src/c_api/c_api.cc:935: `ntree_limit` is deprecated, use `iteration_range` instead.
## [16:05:39] WARNING: src/c_api/c_api.cc:935: `ntree_limit` is deprecated, use `iteration_range` instead.
## [16:05:39] WARNING: src/c_api/c_api.cc:935: `ntree_limit` is deprecated, use `iteration_range` instead.
## [16:05:40] WARNING: src/c_api/c_api.cc:935: `ntree_limit` is deprecated, use `iteration_range` instead.
## [16:05:40] WARNING: src/c_api/c_api.cc:935: `ntree_limit` is deprecated, use `iteration_range` instead.
## [16:05:40] WARNING: src/c_api/c_api.cc:935: `ntree_limit` is deprecated, use `iteration_range` instead.
## [16:05:40] WARNING: src/c_api/c_api.cc:935: `ntree_limit` is deprecated, use `iteration_range` instead.
## [16:05:40] WARNING: src/c_api/c_api.cc:935: `ntree_limit` is deprecated, use `iteration_range` instead.
## [16:05:40] WARNING: src/c_api/c_api.cc:935: `ntree_limit` is deprecated, use `iteration_range` instead.
## [16:05:40] WARNING: src/c_api/c_api.cc:935: `ntree_limit` is deprecated, use `iteration_range` instead.
## [16:05:40] WARNING: src/c_api/c_api.cc:935: `ntree_limit` is deprecated, use `iteration_range` instead.
## [16:05:40] WARNING: src/c_api/c_api.cc:935: `ntree_limit` is deprecated, use `iteration_range` instead.
## [16:05:40] WARNING: src/c_api/c_api.cc:935: `ntree_limit` is deprecated, use `iteration_range` instead.
## [16:05:41] WARNING: src/c_api/c_api.cc:935: `ntree_limit` is deprecated, use `iteration_range` instead.
## [16:05:41] WARNING: src/c_api/c_api.cc:935: `ntree_limit` is deprecated, use `iteration_range` instead.
## [16:05:41] WARNING: src/c_api/c_api.cc:935: `ntree_limit` is deprecated, use `iteration_range` instead.
## [16:05:41] WARNING: src/c_api/c_api.cc:935: `ntree_limit` is deprecated, use `iteration_range` instead.
## [16:05:41] WARNING: src/c_api/c_api.cc:935: `ntree_limit` is deprecated, use `iteration_range` instead.
## [16:05:41] WARNING: src/c_api/c_api.cc:935: `ntree_limit` is deprecated, use `iteration_range` instead.
## [16:05:41] WARNING: src/c_api/c_api.cc:935: `ntree_limit` is deprecated, use `iteration_range` instead.
## [16:05:41] WARNING: src/c_api/c_api.cc:935: `ntree_limit` is deprecated, use `iteration_range` instead.
## [16:05:41] WARNING: src/c_api/c_api.cc:935: `ntree_limit` is deprecated, use `iteration_range` instead.
## [16:05:41] WARNING: src/c_api/c_api.cc:935: `ntree_limit` is deprecated, use `iteration_range` instead.
## [16:05:41] WARNING: src/c_api/c_api.cc:935: `ntree_limit` is deprecated, use `iteration_range` instead.
## [16:05:41] WARNING: src/c_api/c_api.cc:935: `ntree_limit` is deprecated, use `iteration_range` instead.
## [16:05:42] WARNING: src/c_api/c_api.cc:935: `ntree_limit` is deprecated, use `iteration_range` instead.
## [16:05:42] WARNING: src/c_api/c_api.cc:935: `ntree_limit` is deprecated, use `iteration_range` instead.
## [16:05:42] WARNING: src/c_api/c_api.cc:935: `ntree_limit` is deprecated, use `iteration_range` instead.
## [16:05:42] WARNING: src/c_api/c_api.cc:935: `ntree_limit` is deprecated, use `iteration_range` instead.
## [16:05:42] WARNING: src/c_api/c_api.cc:935: `ntree_limit` is deprecated, use `iteration_range` instead.
## [16:05:42] WARNING: src/c_api/c_api.cc:935: `ntree_limit` is deprecated, use `iteration_range` instead.
## [16:05:42] WARNING: src/c_api/c_api.cc:935: `ntree_limit` is deprecated, use `iteration_range` instead.
## [16:05:42] WARNING: src/c_api/c_api.cc:935: `ntree_limit` is deprecated, use `iteration_range` instead.
## [16:05:42] WARNING: src/c_api/c_api.cc:935: `ntree_limit` is deprecated, use `iteration_range` instead.
## [16:05:42] WARNING: src/c_api/c_api.cc:935: `ntree_limit` is deprecated, use `iteration_range` instead.
## [16:05:43] WARNING: src/c_api/c_api.cc:935: `ntree_limit` is deprecated, use `iteration_range` instead.
## [16:05:43] WARNING: src/c_api/c_api.cc:935: `ntree_limit` is deprecated, use `iteration_range` instead.
## [16:05:43] WARNING: src/c_api/c_api.cc:935: `ntree_limit` is deprecated, use `iteration_range` instead.
## [16:05:43] WARNING: src/c_api/c_api.cc:935: `ntree_limit` is deprecated, use `iteration_range` instead.
## [16:05:43] WARNING: src/c_api/c_api.cc:935: `ntree_limit` is deprecated, use `iteration_range` instead.
## [16:05:43] WARNING: src/c_api/c_api.cc:935: `ntree_limit` is deprecated, use `iteration_range` instead.
## [16:05:43] WARNING: src/c_api/c_api.cc:935: `ntree_limit` is deprecated, use `iteration_range` instead.
## [16:05:43] WARNING: src/c_api/c_api.cc:935: `ntree_limit` is deprecated, use `iteration_range` instead.
## [16:05:43] WARNING: src/c_api/c_api.cc:935: `ntree_limit` is deprecated, use `iteration_range` instead.
## [16:05:43] WARNING: src/c_api/c_api.cc:935: `ntree_limit` is deprecated, use `iteration_range` instead.
## [16:05:46] WARNING: src/c_api/c_api.cc:935: `ntree_limit` is deprecated, use `iteration_range` instead.
## [16:05:46] WARNING: src/c_api/c_api.cc:935: `ntree_limit` is deprecated, use `iteration_range` instead.
## [16:05:47] WARNING: src/c_api/c_api.cc:935: `ntree_limit` is deprecated, use `iteration_range` instead.
## [16:05:47] WARNING: src/c_api/c_api.cc:935: `ntree_limit` is deprecated, use `iteration_range` instead.
## [16:05:47] WARNING: src/c_api/c_api.cc:935: `ntree_limit` is deprecated, use `iteration_range` instead.
## [16:05:47] WARNING: src/c_api/c_api.cc:935: `ntree_limit` is deprecated, use `iteration_range` instead.
## [16:05:47] WARNING: src/c_api/c_api.cc:935: `ntree_limit` is deprecated, use `iteration_range` instead.
## [16:05:47] WARNING: src/c_api/c_api.cc:935: `ntree_limit` is deprecated, use `iteration_range` instead.
## [16:05:47] WARNING: src/c_api/c_api.cc:935: `ntree_limit` is deprecated, use `iteration_range` instead.
## [16:05:47] WARNING: src/c_api/c_api.cc:935: `ntree_limit` is deprecated, use `iteration_range` instead.
## [16:05:47] WARNING: src/c_api/c_api.cc:935: `ntree_limit` is deprecated, use `iteration_range` instead.
## [16:05:47] WARNING: src/c_api/c_api.cc:935: `ntree_limit` is deprecated, use `iteration_range` instead.
## [16:05:47] WARNING: src/c_api/c_api.cc:935: `ntree_limit` is deprecated, use `iteration_range` instead.
## [16:05:47] WARNING: src/c_api/c_api.cc:935: `ntree_limit` is deprecated, use `iteration_range` instead.
## [16:05:47] WARNING: src/c_api/c_api.cc:935: `ntree_limit` is deprecated, use `iteration_range` instead.
## [16:05:47] WARNING: src/c_api/c_api.cc:935: `ntree_limit` is deprecated, use `iteration_range` instead.
## [16:05:48] WARNING: src/c_api/c_api.cc:935: `ntree_limit` is deprecated, use `iteration_range` instead.
## [16:05:48] WARNING: src/c_api/c_api.cc:935: `ntree_limit` is deprecated, use `iteration_range` instead.
## [16:05:48] WARNING: src/c_api/c_api.cc:935: `ntree_limit` is deprecated, use `iteration_range` instead.
## [16:05:48] WARNING: src/c_api/c_api.cc:935: `ntree_limit` is deprecated, use `iteration_range` instead.
## [16:05:48] WARNING: src/c_api/c_api.cc:935: `ntree_limit` is deprecated, use `iteration_range` instead.
## [16:05:48] WARNING: src/c_api/c_api.cc:935: `ntree_limit` is deprecated, use `iteration_range` instead.
## [16:05:48] WARNING: src/c_api/c_api.cc:935: `ntree_limit` is deprecated, use `iteration_range` instead.
## [16:05:48] WARNING: src/c_api/c_api.cc:935: `ntree_limit` is deprecated, use `iteration_range` instead.
## [16:05:48] WARNING: src/c_api/c_api.cc:935: `ntree_limit` is deprecated, use `iteration_range` instead.
## [16:05:48] WARNING: src/c_api/c_api.cc:935: `ntree_limit` is deprecated, use `iteration_range` instead.
## [16:05:49] WARNING: src/c_api/c_api.cc:935: `ntree_limit` is deprecated, use `iteration_range` instead.
## [16:05:49] WARNING: src/c_api/c_api.cc:935: `ntree_limit` is deprecated, use `iteration_range` instead.
## [16:05:49] WARNING: src/c_api/c_api.cc:935: `ntree_limit` is deprecated, use `iteration_range` instead.
## [16:05:49] WARNING: src/c_api/c_api.cc:935: `ntree_limit` is deprecated, use `iteration_range` instead.
## [16:05:49] WARNING: src/c_api/c_api.cc:935: `ntree_limit` is deprecated, use `iteration_range` instead.
## [16:05:49] WARNING: src/c_api/c_api.cc:935: `ntree_limit` is deprecated, use `iteration_range` instead.
## [16:05:49] WARNING: src/c_api/c_api.cc:935: `ntree_limit` is deprecated, use `iteration_range` instead.
## [16:05:49] WARNING: src/c_api/c_api.cc:935: `ntree_limit` is deprecated, use `iteration_range` instead.
## [16:05:50] WARNING: src/c_api/c_api.cc:935: `ntree_limit` is deprecated, use `iteration_range` instead.
## [16:05:50] WARNING: src/c_api/c_api.cc:935: `ntree_limit` is deprecated, use `iteration_range` instead.
## [16:05:50] WARNING: src/c_api/c_api.cc:935: `ntree_limit` is deprecated, use `iteration_range` instead.
## [16:05:50] WARNING: src/c_api/c_api.cc:935: `ntree_limit` is deprecated, use `iteration_range` instead.
## [16:05:50] WARNING: src/c_api/c_api.cc:935: `ntree_limit` is deprecated, use `iteration_range` instead.
## [16:05:50] WARNING: src/c_api/c_api.cc:935: `ntree_limit` is deprecated, use `iteration_range` instead.
## [16:05:50] WARNING: src/c_api/c_api.cc:935: `ntree_limit` is deprecated, use `iteration_range` instead.
## [16:05:50] WARNING: src/c_api/c_api.cc:935: `ntree_limit` is deprecated, use `iteration_range` instead.
## [16:05:50] WARNING: src/c_api/c_api.cc:935: `ntree_limit` is deprecated, use `iteration_range` instead.
## [16:05:50] WARNING: src/c_api/c_api.cc:935: `ntree_limit` is deprecated, use `iteration_range` instead.
## [16:05:50] WARNING: src/c_api/c_api.cc:935: `ntree_limit` is deprecated, use `iteration_range` instead.
## [16:05:50] WARNING: src/c_api/c_api.cc:935: `ntree_limit` is deprecated, use `iteration_range` instead.
## [16:05:50] WARNING: src/c_api/c_api.cc:935: `ntree_limit` is deprecated, use `iteration_range` instead.
## [16:05:50] WARNING: src/c_api/c_api.cc:935: `ntree_limit` is deprecated, use `iteration_range` instead.
## [16:05:51] WARNING: src/c_api/c_api.cc:935: `ntree_limit` is deprecated, use `iteration_range` instead.
## [16:05:51] WARNING: src/c_api/c_api.cc:935: `ntree_limit` is deprecated, use `iteration_range` instead.
## [16:05:51] WARNING: src/c_api/c_api.cc:935: `ntree_limit` is deprecated, use `iteration_range` instead.
## [16:05:51] WARNING: src/c_api/c_api.cc:935: `ntree_limit` is deprecated, use `iteration_range` instead.
## [16:05:51] WARNING: src/c_api/c_api.cc:935: `ntree_limit` is deprecated, use `iteration_range` instead.
## [16:05:51] WARNING: src/c_api/c_api.cc:935: `ntree_limit` is deprecated, use `iteration_range` instead.
## [16:05:51] WARNING: src/c_api/c_api.cc:935: `ntree_limit` is deprecated, use `iteration_range` instead.
## [16:05:51] WARNING: src/c_api/c_api.cc:935: `ntree_limit` is deprecated, use `iteration_range` instead.
## [16:05:51] WARNING: src/c_api/c_api.cc:935: `ntree_limit` is deprecated, use `iteration_range` instead.
## [16:05:51] WARNING: src/c_api/c_api.cc:935: `ntree_limit` is deprecated, use `iteration_range` instead.
## [16:05:52] WARNING: src/c_api/c_api.cc:935: `ntree_limit` is deprecated, use `iteration_range` instead.
## [16:05:52] WARNING: src/c_api/c_api.cc:935: `ntree_limit` is deprecated, use `iteration_range` instead.
## [16:05:52] WARNING: src/c_api/c_api.cc:935: `ntree_limit` is deprecated, use `iteration_range` instead.
## [16:05:52] WARNING: src/c_api/c_api.cc:935: `ntree_limit` is deprecated, use `iteration_range` instead.
## [16:05:52] WARNING: src/c_api/c_api.cc:935: `ntree_limit` is deprecated, use `iteration_range` instead.
## [16:05:52] WARNING: src/c_api/c_api.cc:935: `ntree_limit` is deprecated, use `iteration_range` instead.
## [16:05:52] WARNING: src/c_api/c_api.cc:935: `ntree_limit` is deprecated, use `iteration_range` instead.
## [16:05:52] WARNING: src/c_api/c_api.cc:935: `ntree_limit` is deprecated, use `iteration_range` instead.
## [16:05:52] WARNING: src/c_api/c_api.cc:935: `ntree_limit` is deprecated, use `iteration_range` instead.
## [16:05:52] WARNING: src/c_api/c_api.cc:935: `ntree_limit` is deprecated, use `iteration_range` instead.
## [16:05:53] WARNING: src/c_api/c_api.cc:935: `ntree_limit` is deprecated, use `iteration_range` instead.
## [16:05:53] WARNING: src/c_api/c_api.cc:935: `ntree_limit` is deprecated, use `iteration_range` instead.
## [16:05:53] WARNING: src/c_api/c_api.cc:935: `ntree_limit` is deprecated, use `iteration_range` instead.
## [16:05:53] WARNING: src/c_api/c_api.cc:935: `ntree_limit` is deprecated, use `iteration_range` instead.
## [16:05:53] WARNING: src/c_api/c_api.cc:935: `ntree_limit` is deprecated, use `iteration_range` instead.
## [16:05:53] WARNING: src/c_api/c_api.cc:935: `ntree_limit` is deprecated, use `iteration_range` instead.
## [16:05:53] WARNING: src/c_api/c_api.cc:935: `ntree_limit` is deprecated, use `iteration_range` instead.
## [16:05:53] WARNING: src/c_api/c_api.cc:935: `ntree_limit` is deprecated, use `iteration_range` instead.
## [16:05:53] WARNING: src/c_api/c_api.cc:935: `ntree_limit` is deprecated, use `iteration_range` instead.
## [16:05:53] WARNING: src/c_api/c_api.cc:935: `ntree_limit` is deprecated, use `iteration_range` instead.
## [16:05:53] WARNING: src/c_api/c_api.cc:935: `ntree_limit` is deprecated, use `iteration_range` instead.
## [16:05:53] WARNING: src/c_api/c_api.cc:935: `ntree_limit` is deprecated, use `iteration_range` instead.
## [16:05:53] WARNING: src/c_api/c_api.cc:935: `ntree_limit` is deprecated, use `iteration_range` instead.
## [16:05:53] WARNING: src/c_api/c_api.cc:935: `ntree_limit` is deprecated, use `iteration_range` instead.
## [16:05:54] WARNING: src/c_api/c_api.cc:935: `ntree_limit` is deprecated, use `iteration_range` instead.
## [16:05:54] WARNING: src/c_api/c_api.cc:935: `ntree_limit` is deprecated, use `iteration_range` instead.
## [16:05:54] WARNING: src/c_api/c_api.cc:935: `ntree_limit` is deprecated, use `iteration_range` instead.
## [16:05:54] WARNING: src/c_api/c_api.cc:935: `ntree_limit` is deprecated, use `iteration_range` instead.
## [16:05:54] WARNING: src/c_api/c_api.cc:935: `ntree_limit` is deprecated, use `iteration_range` instead.
## [16:05:54] WARNING: src/c_api/c_api.cc:935: `ntree_limit` is deprecated, use `iteration_range` instead.
## [16:05:54] WARNING: src/c_api/c_api.cc:935: `ntree_limit` is deprecated, use `iteration_range` instead.
## [16:05:54] WARNING: src/c_api/c_api.cc:935: `ntree_limit` is deprecated, use `iteration_range` instead.
## [16:05:54] WARNING: src/c_api/c_api.cc:935: `ntree_limit` is deprecated, use `iteration_range` instead.
## [16:05:54] WARNING: src/c_api/c_api.cc:935: `ntree_limit` is deprecated, use `iteration_range` instead.
## [16:05:55] WARNING: src/c_api/c_api.cc:935: `ntree_limit` is deprecated, use `iteration_range` instead.
## [16:05:55] WARNING: src/c_api/c_api.cc:935: `ntree_limit` is deprecated, use `iteration_range` instead.
## [16:05:55] WARNING: src/c_api/c_api.cc:935: `ntree_limit` is deprecated, use `iteration_range` instead.
## [16:05:55] WARNING: src/c_api/c_api.cc:935: `ntree_limit` is deprecated, use `iteration_range` instead.
## [16:05:55] WARNING: src/c_api/c_api.cc:935: `ntree_limit` is deprecated, use `iteration_range` instead.
## [16:05:55] WARNING: src/c_api/c_api.cc:935: `ntree_limit` is deprecated, use `iteration_range` instead.
## [16:05:55] WARNING: src/c_api/c_api.cc:935: `ntree_limit` is deprecated, use `iteration_range` instead.
## [16:05:55] WARNING: src/c_api/c_api.cc:935: `ntree_limit` is deprecated, use `iteration_range` instead.
## [16:05:56] WARNING: src/c_api/c_api.cc:935: `ntree_limit` is deprecated, use `iteration_range` instead.
## [16:05:56] WARNING: src/c_api/c_api.cc:935: `ntree_limit` is deprecated, use `iteration_range` instead.
## [16:05:56] WARNING: src/c_api/c_api.cc:935: `ntree_limit` is deprecated, use `iteration_range` instead.
## [16:05:56] WARNING: src/c_api/c_api.cc:935: `ntree_limit` is deprecated, use `iteration_range` instead.
## [16:05:56] WARNING: src/c_api/c_api.cc:935: `ntree_limit` is deprecated, use `iteration_range` instead.
## [16:05:56] WARNING: src/c_api/c_api.cc:935: `ntree_limit` is deprecated, use `iteration_range` instead.
## [16:05:56] WARNING: src/c_api/c_api.cc:935: `ntree_limit` is deprecated, use `iteration_range` instead.
## [16:05:56] WARNING: src/c_api/c_api.cc:935: `ntree_limit` is deprecated, use `iteration_range` instead.
## [16:05:56] WARNING: src/c_api/c_api.cc:935: `ntree_limit` is deprecated, use `iteration_range` instead.
## [16:05:56] WARNING: src/c_api/c_api.cc:935: `ntree_limit` is deprecated, use `iteration_range` instead.
## [16:05:56] WARNING: src/c_api/c_api.cc:935: `ntree_limit` is deprecated, use `iteration_range` instead.
## [16:05:56] WARNING: src/c_api/c_api.cc:935: `ntree_limit` is deprecated, use `iteration_range` instead.
## [16:05:57] WARNING: src/c_api/c_api.cc:935: `ntree_limit` is deprecated, use `iteration_range` instead.
## [16:05:57] WARNING: src/c_api/c_api.cc:935: `ntree_limit` is deprecated, use `iteration_range` instead.
## [16:05:57] WARNING: src/c_api/c_api.cc:935: `ntree_limit` is deprecated, use `iteration_range` instead.
## [16:05:57] WARNING: src/c_api/c_api.cc:935: `ntree_limit` is deprecated, use `iteration_range` instead.
## [16:05:57] WARNING: src/c_api/c_api.cc:935: `ntree_limit` is deprecated, use `iteration_range` instead.
## [16:05:57] WARNING: src/c_api/c_api.cc:935: `ntree_limit` is deprecated, use `iteration_range` instead.
## [16:05:57] WARNING: src/c_api/c_api.cc:935: `ntree_limit` is deprecated, use `iteration_range` instead.
## [16:05:57] WARNING: src/c_api/c_api.cc:935: `ntree_limit` is deprecated, use `iteration_range` instead.
## [16:05:57] WARNING: src/c_api/c_api.cc:935: `ntree_limit` is deprecated, use `iteration_range` instead.
## [16:05:57] WARNING: src/c_api/c_api.cc:935: `ntree_limit` is deprecated, use `iteration_range` instead.
## [16:05:57] WARNING: src/c_api/c_api.cc:935: `ntree_limit` is deprecated, use `iteration_range` instead.
## [16:05:57] WARNING: src/c_api/c_api.cc:935: `ntree_limit` is deprecated, use `iteration_range` instead.
## [16:05:58] WARNING: src/c_api/c_api.cc:935: `ntree_limit` is deprecated, use `iteration_range` instead.
## [16:05:58] WARNING: src/c_api/c_api.cc:935: `ntree_limit` is deprecated, use `iteration_range` instead.
## [16:05:58] WARNING: src/c_api/c_api.cc:935: `ntree_limit` is deprecated, use `iteration_range` instead.
## [16:05:58] WARNING: src/c_api/c_api.cc:935: `ntree_limit` is deprecated, use `iteration_range` instead.
## [16:05:58] WARNING: src/c_api/c_api.cc:935: `ntree_limit` is deprecated, use `iteration_range` instead.
## [16:05:58] WARNING: src/c_api/c_api.cc:935: `ntree_limit` is deprecated, use `iteration_range` instead.
## [16:05:58] WARNING: src/c_api/c_api.cc:935: `ntree_limit` is deprecated, use `iteration_range` instead.
## [16:05:58] WARNING: src/c_api/c_api.cc:935: `ntree_limit` is deprecated, use `iteration_range` instead.
## [16:05:58] WARNING: src/c_api/c_api.cc:935: `ntree_limit` is deprecated, use `iteration_range` instead.
## [16:05:58] WARNING: src/c_api/c_api.cc:935: `ntree_limit` is deprecated, use `iteration_range` instead.
## [16:05:59] WARNING: src/c_api/c_api.cc:935: `ntree_limit` is deprecated, use `iteration_range` instead.
## [16:05:59] WARNING: src/c_api/c_api.cc:935: `ntree_limit` is deprecated, use `iteration_range` instead.
## [16:05:59] WARNING: src/c_api/c_api.cc:935: `ntree_limit` is deprecated, use `iteration_range` instead.
## [16:05:59] WARNING: src/c_api/c_api.cc:935: `ntree_limit` is deprecated, use `iteration_range` instead.
## [16:05:59] WARNING: src/c_api/c_api.cc:935: `ntree_limit` is deprecated, use `iteration_range` instead.
## [16:05:59] WARNING: src/c_api/c_api.cc:935: `ntree_limit` is deprecated, use `iteration_range` instead.
## [16:05:59] WARNING: src/c_api/c_api.cc:935: `ntree_limit` is deprecated, use `iteration_range` instead.
## [16:05:59] WARNING: src/c_api/c_api.cc:935: `ntree_limit` is deprecated, use `iteration_range` instead.
## [16:05:59] WARNING: src/c_api/c_api.cc:935: `ntree_limit` is deprecated, use `iteration_range` instead.
## [16:05:59] WARNING: src/c_api/c_api.cc:935: `ntree_limit` is deprecated, use `iteration_range` instead.
## [16:06:00] WARNING: src/c_api/c_api.cc:935: `ntree_limit` is deprecated, use `iteration_range` instead.
## [16:06:00] WARNING: src/c_api/c_api.cc:935: `ntree_limit` is deprecated, use `iteration_range` instead.
## [16:06:00] WARNING: src/c_api/c_api.cc:935: `ntree_limit` is deprecated, use `iteration_range` instead.
## [16:06:00] WARNING: src/c_api/c_api.cc:935: `ntree_limit` is deprecated, use `iteration_range` instead.
## [16:06:00] WARNING: src/c_api/c_api.cc:935: `ntree_limit` is deprecated, use `iteration_range` instead.
## [16:06:00] WARNING: src/c_api/c_api.cc:935: `ntree_limit` is deprecated, use `iteration_range` instead.
## [16:06:00] WARNING: src/c_api/c_api.cc:935: `ntree_limit` is deprecated, use `iteration_range` instead.
## [16:06:00] WARNING: src/c_api/c_api.cc:935: `ntree_limit` is deprecated, use `iteration_range` instead.
## [16:06:00] WARNING: src/c_api/c_api.cc:935: `ntree_limit` is deprecated, use `iteration_range` instead.
## [16:06:00] WARNING: src/c_api/c_api.cc:935: `ntree_limit` is deprecated, use `iteration_range` instead.
## [16:06:00] WARNING: src/c_api/c_api.cc:935: `ntree_limit` is deprecated, use `iteration_range` instead.
## [16:06:00] WARNING: src/c_api/c_api.cc:935: `ntree_limit` is deprecated, use `iteration_range` instead.
## [16:06:00] WARNING: src/c_api/c_api.cc:935: `ntree_limit` is deprecated, use `iteration_range` instead.
## [16:06:00] WARNING: src/c_api/c_api.cc:935: `ntree_limit` is deprecated, use `iteration_range` instead.
## [16:06:01] WARNING: src/c_api/c_api.cc:935: `ntree_limit` is deprecated, use `iteration_range` instead.
## [16:06:01] WARNING: src/c_api/c_api.cc:935: `ntree_limit` is deprecated, use `iteration_range` instead.
## [16:06:01] WARNING: src/c_api/c_api.cc:935: `ntree_limit` is deprecated, use `iteration_range` instead.
## [16:06:01] WARNING: src/c_api/c_api.cc:935: `ntree_limit` is deprecated, use `iteration_range` instead.
## [16:06:01] WARNING: src/c_api/c_api.cc:935: `ntree_limit` is deprecated, use `iteration_range` instead.
## [16:06:01] WARNING: src/c_api/c_api.cc:935: `ntree_limit` is deprecated, use `iteration_range` instead.
## [16:06:01] WARNING: src/c_api/c_api.cc:935: `ntree_limit` is deprecated, use `iteration_range` instead.
## [16:06:01] WARNING: src/c_api/c_api.cc:935: `ntree_limit` is deprecated, use `iteration_range` instead.
## [16:06:01] WARNING: src/c_api/c_api.cc:935: `ntree_limit` is deprecated, use `iteration_range` instead.
## [16:06:01] WARNING: src/c_api/c_api.cc:935: `ntree_limit` is deprecated, use `iteration_range` instead.
## [16:06:02] WARNING: src/c_api/c_api.cc:935: `ntree_limit` is deprecated, use `iteration_range` instead.
## [16:06:02] WARNING: src/c_api/c_api.cc:935: `ntree_limit` is deprecated, use `iteration_range` instead.
## [16:06:02] WARNING: src/c_api/c_api.cc:935: `ntree_limit` is deprecated, use `iteration_range` instead.
## [16:06:02] WARNING: src/c_api/c_api.cc:935: `ntree_limit` is deprecated, use `iteration_range` instead.
## [16:06:02] WARNING: src/c_api/c_api.cc:935: `ntree_limit` is deprecated, use `iteration_range` instead.
## [16:06:02] WARNING: src/c_api/c_api.cc:935: `ntree_limit` is deprecated, use `iteration_range` instead.
## [16:06:02] WARNING: src/c_api/c_api.cc:935: `ntree_limit` is deprecated, use `iteration_range` instead.
## [16:06:02] WARNING: src/c_api/c_api.cc:935: `ntree_limit` is deprecated, use `iteration_range` instead.
## [16:06:03] WARNING: src/c_api/c_api.cc:935: `ntree_limit` is deprecated, use `iteration_range` instead.
## [16:06:03] WARNING: src/c_api/c_api.cc:935: `ntree_limit` is deprecated, use `iteration_range` instead.
## [16:06:03] WARNING: src/c_api/c_api.cc:935: `ntree_limit` is deprecated, use `iteration_range` instead.
## [16:06:03] WARNING: src/c_api/c_api.cc:935: `ntree_limit` is deprecated, use `iteration_range` instead.
## [16:06:03] WARNING: src/c_api/c_api.cc:935: `ntree_limit` is deprecated, use `iteration_range` instead.
## [16:06:03] WARNING: src/c_api/c_api.cc:935: `ntree_limit` is deprecated, use `iteration_range` instead.
## [16:06:03] WARNING: src/c_api/c_api.cc:935: `ntree_limit` is deprecated, use `iteration_range` instead.
## [16:06:03] WARNING: src/c_api/c_api.cc:935: `ntree_limit` is deprecated, use `iteration_range` instead.
## [16:06:03] WARNING: src/c_api/c_api.cc:935: `ntree_limit` is deprecated, use `iteration_range` instead.
## [16:06:03] WARNING: src/c_api/c_api.cc:935: `ntree_limit` is deprecated, use `iteration_range` instead.
## [16:06:03] WARNING: src/c_api/c_api.cc:935: `ntree_limit` is deprecated, use `iteration_range` instead.
## [16:06:03] WARNING: src/c_api/c_api.cc:935: `ntree_limit` is deprecated, use `iteration_range` instead.
## [16:06:04] WARNING: src/c_api/c_api.cc:935: `ntree_limit` is deprecated, use `iteration_range` instead.
## [16:06:04] WARNING: src/c_api/c_api.cc:935: `ntree_limit` is deprecated, use `iteration_range` instead.
## [16:06:04] WARNING: src/c_api/c_api.cc:935: `ntree_limit` is deprecated, use `iteration_range` instead.
## [16:06:04] WARNING: src/c_api/c_api.cc:935: `ntree_limit` is deprecated, use `iteration_range` instead.
## [16:06:04] WARNING: src/c_api/c_api.cc:935: `ntree_limit` is deprecated, use `iteration_range` instead.
## [16:06:04] WARNING: src/c_api/c_api.cc:935: `ntree_limit` is deprecated, use `iteration_range` instead.
## [16:06:04] WARNING: src/c_api/c_api.cc:935: `ntree_limit` is deprecated, use `iteration_range` instead.
## [16:06:04] WARNING: src/c_api/c_api.cc:935: `ntree_limit` is deprecated, use `iteration_range` instead.
## [16:06:04] WARNING: src/c_api/c_api.cc:935: `ntree_limit` is deprecated, use `iteration_range` instead.
## [16:06:04] WARNING: src/c_api/c_api.cc:935: `ntree_limit` is deprecated, use `iteration_range` instead.
## [16:06:04] WARNING: src/c_api/c_api.cc:935: `ntree_limit` is deprecated, use `iteration_range` instead.
## [16:06:04] WARNING: src/c_api/c_api.cc:935: `ntree_limit` is deprecated, use `iteration_range` instead.
## [16:06:05] WARNING: src/c_api/c_api.cc:935: `ntree_limit` is deprecated, use `iteration_range` instead.
## [16:06:05] WARNING: src/c_api/c_api.cc:935: `ntree_limit` is deprecated, use `iteration_range` instead.
## [16:06:05] WARNING: src/c_api/c_api.cc:935: `ntree_limit` is deprecated, use `iteration_range` instead.
## [16:06:05] WARNING: src/c_api/c_api.cc:935: `ntree_limit` is deprecated, use `iteration_range` instead.
## [16:06:05] WARNING: src/c_api/c_api.cc:935: `ntree_limit` is deprecated, use `iteration_range` instead.
## [16:06:05] WARNING: src/c_api/c_api.cc:935: `ntree_limit` is deprecated, use `iteration_range` instead.
## [16:06:05] WARNING: src/c_api/c_api.cc:935: `ntree_limit` is deprecated, use `iteration_range` instead.
## [16:06:05] WARNING: src/c_api/c_api.cc:935: `ntree_limit` is deprecated, use `iteration_range` instead.
## [16:06:05] WARNING: src/c_api/c_api.cc:935: `ntree_limit` is deprecated, use `iteration_range` instead.
## [16:06:05] WARNING: src/c_api/c_api.cc:935: `ntree_limit` is deprecated, use `iteration_range` instead.
## [16:06:06] WARNING: src/c_api/c_api.cc:935: `ntree_limit` is deprecated, use `iteration_range` instead.
## [16:06:06] WARNING: src/c_api/c_api.cc:935: `ntree_limit` is deprecated, use `iteration_range` instead.
## [16:06:06] WARNING: src/c_api/c_api.cc:935: `ntree_limit` is deprecated, use `iteration_range` instead.
## [16:06:06] WARNING: src/c_api/c_api.cc:935: `ntree_limit` is deprecated, use `iteration_range` instead.
## [16:06:06] WARNING: src/c_api/c_api.cc:935: `ntree_limit` is deprecated, use `iteration_range` instead.
## [16:06:06] WARNING: src/c_api/c_api.cc:935: `ntree_limit` is deprecated, use `iteration_range` instead.
## [16:06:06] WARNING: src/c_api/c_api.cc:935: `ntree_limit` is deprecated, use `iteration_range` instead.
## [16:06:06] WARNING: src/c_api/c_api.cc:935: `ntree_limit` is deprecated, use `iteration_range` instead.
## [16:06:06] WARNING: src/c_api/c_api.cc:935: `ntree_limit` is deprecated, use `iteration_range` instead.
## [16:06:06] WARNING: src/c_api/c_api.cc:935: `ntree_limit` is deprecated, use `iteration_range` instead.
## [16:06:06] WARNING: src/c_api/c_api.cc:935: `ntree_limit` is deprecated, use `iteration_range` instead.
## [16:06:06] WARNING: src/c_api/c_api.cc:935: `ntree_limit` is deprecated, use `iteration_range` instead.
## [16:06:06] WARNING: src/c_api/c_api.cc:935: `ntree_limit` is deprecated, use `iteration_range` instead.
## [16:06:06] WARNING: src/c_api/c_api.cc:935: `ntree_limit` is deprecated, use `iteration_range` instead.
## [16:06:07] WARNING: src/c_api/c_api.cc:935: `ntree_limit` is deprecated, use `iteration_range` instead.
## [16:06:07] WARNING: src/c_api/c_api.cc:935: `ntree_limit` is deprecated, use `iteration_range` instead.
## [16:06:07] WARNING: src/c_api/c_api.cc:935: `ntree_limit` is deprecated, use `iteration_range` instead.
## [16:06:07] WARNING: src/c_api/c_api.cc:935: `ntree_limit` is deprecated, use `iteration_range` instead.
## [16:06:07] WARNING: src/c_api/c_api.cc:935: `ntree_limit` is deprecated, use `iteration_range` instead.
## [16:06:07] WARNING: src/c_api/c_api.cc:935: `ntree_limit` is deprecated, use `iteration_range` instead.
## [16:06:07] WARNING: src/c_api/c_api.cc:935: `ntree_limit` is deprecated, use `iteration_range` instead.
## [16:06:07] WARNING: src/c_api/c_api.cc:935: `ntree_limit` is deprecated, use `iteration_range` instead.
## [16:06:07] WARNING: src/c_api/c_api.cc:935: `ntree_limit` is deprecated, use `iteration_range` instead.
## [16:06:07] WARNING: src/c_api/c_api.cc:935: `ntree_limit` is deprecated, use `iteration_range` instead.
## [16:06:08] WARNING: src/c_api/c_api.cc:935: `ntree_limit` is deprecated, use `iteration_range` instead.
## [16:06:08] WARNING: src/c_api/c_api.cc:935: `ntree_limit` is deprecated, use `iteration_range` instead.
## [16:06:08] WARNING: src/c_api/c_api.cc:935: `ntree_limit` is deprecated, use `iteration_range` instead.
## [16:06:08] WARNING: src/c_api/c_api.cc:935: `ntree_limit` is deprecated, use `iteration_range` instead.
## [16:06:08] WARNING: src/c_api/c_api.cc:935: `ntree_limit` is deprecated, use `iteration_range` instead.
## [16:06:08] WARNING: src/c_api/c_api.cc:935: `ntree_limit` is deprecated, use `iteration_range` instead.
## [16:06:08] WARNING: src/c_api/c_api.cc:935: `ntree_limit` is deprecated, use `iteration_range` instead.
## [16:06:08] WARNING: src/c_api/c_api.cc:935: `ntree_limit` is deprecated, use `iteration_range` instead.
## [16:06:08] WARNING: src/c_api/c_api.cc:935: `ntree_limit` is deprecated, use `iteration_range` instead.
## [16:06:08] WARNING: src/c_api/c_api.cc:935: `ntree_limit` is deprecated, use `iteration_range` instead.
## [16:06:09] WARNING: src/c_api/c_api.cc:935: `ntree_limit` is deprecated, use `iteration_range` instead.
## [16:06:09] WARNING: src/c_api/c_api.cc:935: `ntree_limit` is deprecated, use `iteration_range` instead.
## [16:06:09] WARNING: src/c_api/c_api.cc:935: `ntree_limit` is deprecated, use `iteration_range` instead.
## [16:06:09] WARNING: src/c_api/c_api.cc:935: `ntree_limit` is deprecated, use `iteration_range` instead.
## [16:06:09] WARNING: src/c_api/c_api.cc:935: `ntree_limit` is deprecated, use `iteration_range` instead.
## [16:06:09] WARNING: src/c_api/c_api.cc:935: `ntree_limit` is deprecated, use `iteration_range` instead.
## [16:06:09] WARNING: src/c_api/c_api.cc:935: `ntree_limit` is deprecated, use `iteration_range` instead.
## [16:06:09] WARNING: src/c_api/c_api.cc:935: `ntree_limit` is deprecated, use `iteration_range` instead.
## [16:06:09] WARNING: src/c_api/c_api.cc:935: `ntree_limit` is deprecated, use `iteration_range` instead.
## [16:06:09] WARNING: src/c_api/c_api.cc:935: `ntree_limit` is deprecated, use `iteration_range` instead.
## [16:06:10] WARNING: src/c_api/c_api.cc:935: `ntree_limit` is deprecated, use `iteration_range` instead.
## [16:06:10] WARNING: src/c_api/c_api.cc:935: `ntree_limit` is deprecated, use `iteration_range` instead.
## [16:06:10] WARNING: src/c_api/c_api.cc:935: `ntree_limit` is deprecated, use `iteration_range` instead.
## [16:06:10] WARNING: src/c_api/c_api.cc:935: `ntree_limit` is deprecated, use `iteration_range` instead.
## [16:06:10] WARNING: src/c_api/c_api.cc:935: `ntree_limit` is deprecated, use `iteration_range` instead.
## [16:06:10] WARNING: src/c_api/c_api.cc:935: `ntree_limit` is deprecated, use `iteration_range` instead.
## [16:06:10] WARNING: src/c_api/c_api.cc:935: `ntree_limit` is deprecated, use `iteration_range` instead.
## [16:06:10] WARNING: src/c_api/c_api.cc:935: `ntree_limit` is deprecated, use `iteration_range` instead.
## [16:06:10] WARNING: src/c_api/c_api.cc:935: `ntree_limit` is deprecated, use `iteration_range` instead.
## [16:06:10] WARNING: src/c_api/c_api.cc:935: `ntree_limit` is deprecated, use `iteration_range` instead.
## [16:06:10] WARNING: src/c_api/c_api.cc:935: `ntree_limit` is deprecated, use `iteration_range` instead.
## [16:06:10] WARNING: src/c_api/c_api.cc:935: `ntree_limit` is deprecated, use `iteration_range` instead.
## [16:06:11] WARNING: src/c_api/c_api.cc:935: `ntree_limit` is deprecated, use `iteration_range` instead.
## [16:06:11] WARNING: src/c_api/c_api.cc:935: `ntree_limit` is deprecated, use `iteration_range` instead.
## [16:06:11] WARNING: src/c_api/c_api.cc:935: `ntree_limit` is deprecated, use `iteration_range` instead.
## [16:06:11] WARNING: src/c_api/c_api.cc:935: `ntree_limit` is deprecated, use `iteration_range` instead.
## [16:06:11] WARNING: src/c_api/c_api.cc:935: `ntree_limit` is deprecated, use `iteration_range` instead.
## [16:06:11] WARNING: src/c_api/c_api.cc:935: `ntree_limit` is deprecated, use `iteration_range` instead.
## [16:06:11] WARNING: src/c_api/c_api.cc:935: `ntree_limit` is deprecated, use `iteration_range` instead.
## [16:06:11] WARNING: src/c_api/c_api.cc:935: `ntree_limit` is deprecated, use `iteration_range` instead.
## [16:06:11] WARNING: src/c_api/c_api.cc:935: `ntree_limit` is deprecated, use `iteration_range` instead.
## [16:06:11] WARNING: src/c_api/c_api.cc:935: `ntree_limit` is deprecated, use `iteration_range` instead.
## [16:06:12] WARNING: src/c_api/c_api.cc:935: `ntree_limit` is deprecated, use `iteration_range` instead.
## [16:06:12] WARNING: src/c_api/c_api.cc:935: `ntree_limit` is deprecated, use `iteration_range` instead.
## [16:06:12] WARNING: src/c_api/c_api.cc:935: `ntree_limit` is deprecated, use `iteration_range` instead.
## [16:06:12] WARNING: src/c_api/c_api.cc:935: `ntree_limit` is deprecated, use `iteration_range` instead.
## [16:06:12] WARNING: src/c_api/c_api.cc:935: `ntree_limit` is deprecated, use `iteration_range` instead.
## [16:06:12] WARNING: src/c_api/c_api.cc:935: `ntree_limit` is deprecated, use `iteration_range` instead.
## [16:06:12] WARNING: src/c_api/c_api.cc:935: `ntree_limit` is deprecated, use `iteration_range` instead.
## [16:06:12] WARNING: src/c_api/c_api.cc:935: `ntree_limit` is deprecated, use `iteration_range` instead.
## [16:06:12] WARNING: src/c_api/c_api.cc:935: `ntree_limit` is deprecated, use `iteration_range` instead.
## [16:06:12] WARNING: src/c_api/c_api.cc:935: `ntree_limit` is deprecated, use `iteration_range` instead.
## [16:06:13] WARNING: src/c_api/c_api.cc:935: `ntree_limit` is deprecated, use `iteration_range` instead.
## [16:06:13] WARNING: src/c_api/c_api.cc:935: `ntree_limit` is deprecated, use `iteration_range` instead.
## [16:06:16] WARNING: src/c_api/c_api.cc:935: `ntree_limit` is deprecated, use `iteration_range` instead.
## [16:06:16] WARNING: src/c_api/c_api.cc:935: `ntree_limit` is deprecated, use `iteration_range` instead.
## [16:06:16] WARNING: src/c_api/c_api.cc:935: `ntree_limit` is deprecated, use `iteration_range` instead.
## [16:06:16] WARNING: src/c_api/c_api.cc:935: `ntree_limit` is deprecated, use `iteration_range` instead.
## [16:06:16] WARNING: src/c_api/c_api.cc:935: `ntree_limit` is deprecated, use `iteration_range` instead.
## [16:06:16] WARNING: src/c_api/c_api.cc:935: `ntree_limit` is deprecated, use `iteration_range` instead.
## [16:06:16] WARNING: src/c_api/c_api.cc:935: `ntree_limit` is deprecated, use `iteration_range` instead.
## [16:06:16] WARNING: src/c_api/c_api.cc:935: `ntree_limit` is deprecated, use `iteration_range` instead.
## [16:06:16] WARNING: src/c_api/c_api.cc:935: `ntree_limit` is deprecated, use `iteration_range` instead.
## [16:06:16] WARNING: src/c_api/c_api.cc:935: `ntree_limit` is deprecated, use `iteration_range` instead.
## [16:06:17] WARNING: src/c_api/c_api.cc:935: `ntree_limit` is deprecated, use `iteration_range` instead.
## [16:06:17] WARNING: src/c_api/c_api.cc:935: `ntree_limit` is deprecated, use `iteration_range` instead.
## [16:06:17] WARNING: src/c_api/c_api.cc:935: `ntree_limit` is deprecated, use `iteration_range` instead.
## [16:06:17] WARNING: src/c_api/c_api.cc:935: `ntree_limit` is deprecated, use `iteration_range` instead.
## [16:06:17] WARNING: src/c_api/c_api.cc:935: `ntree_limit` is deprecated, use `iteration_range` instead.
## [16:06:17] WARNING: src/c_api/c_api.cc:935: `ntree_limit` is deprecated, use `iteration_range` instead.
## [16:06:17] WARNING: src/c_api/c_api.cc:935: `ntree_limit` is deprecated, use `iteration_range` instead.
## [16:06:17] WARNING: src/c_api/c_api.cc:935: `ntree_limit` is deprecated, use `iteration_range` instead.
## [16:06:17] WARNING: src/c_api/c_api.cc:935: `ntree_limit` is deprecated, use `iteration_range` instead.
## [16:06:17] WARNING: src/c_api/c_api.cc:935: `ntree_limit` is deprecated, use `iteration_range` instead.
## [16:06:18] WARNING: src/c_api/c_api.cc:935: `ntree_limit` is deprecated, use `iteration_range` instead.
## [16:06:18] WARNING: src/c_api/c_api.cc:935: `ntree_limit` is deprecated, use `iteration_range` instead.
## [16:06:18] WARNING: src/c_api/c_api.cc:935: `ntree_limit` is deprecated, use `iteration_range` instead.
## [16:06:18] WARNING: src/c_api/c_api.cc:935: `ntree_limit` is deprecated, use `iteration_range` instead.
## [16:06:18] WARNING: src/c_api/c_api.cc:935: `ntree_limit` is deprecated, use `iteration_range` instead.
## [16:06:18] WARNING: src/c_api/c_api.cc:935: `ntree_limit` is deprecated, use `iteration_range` instead.
## [16:06:18] WARNING: src/c_api/c_api.cc:935: `ntree_limit` is deprecated, use `iteration_range` instead.
## [16:06:18] WARNING: src/c_api/c_api.cc:935: `ntree_limit` is deprecated, use `iteration_range` instead.
## [16:06:18] WARNING: src/c_api/c_api.cc:935: `ntree_limit` is deprecated, use `iteration_range` instead.
## [16:06:18] WARNING: src/c_api/c_api.cc:935: `ntree_limit` is deprecated, use `iteration_range` instead.
## [16:06:19] WARNING: src/c_api/c_api.cc:935: `ntree_limit` is deprecated, use `iteration_range` instead.
## [16:06:19] WARNING: src/c_api/c_api.cc:935: `ntree_limit` is deprecated, use `iteration_range` instead.
## [16:06:19] WARNING: src/c_api/c_api.cc:935: `ntree_limit` is deprecated, use `iteration_range` instead.
## [16:06:19] WARNING: src/c_api/c_api.cc:935: `ntree_limit` is deprecated, use `iteration_range` instead.
## [16:06:19] WARNING: src/c_api/c_api.cc:935: `ntree_limit` is deprecated, use `iteration_range` instead.
## [16:06:19] WARNING: src/c_api/c_api.cc:935: `ntree_limit` is deprecated, use `iteration_range` instead.
## [16:06:19] WARNING: src/c_api/c_api.cc:935: `ntree_limit` is deprecated, use `iteration_range` instead.
## [16:06:19] WARNING: src/c_api/c_api.cc:935: `ntree_limit` is deprecated, use `iteration_range` instead.
## [16:06:19] WARNING: src/c_api/c_api.cc:935: `ntree_limit` is deprecated, use `iteration_range` instead.
## [16:06:19] WARNING: src/c_api/c_api.cc:935: `ntree_limit` is deprecated, use `iteration_range` instead.
## [16:06:20] WARNING: src/c_api/c_api.cc:935: `ntree_limit` is deprecated, use `iteration_range` instead.
## [16:06:20] WARNING: src/c_api/c_api.cc:935: `ntree_limit` is deprecated, use `iteration_range` instead.
## [16:06:20] WARNING: src/c_api/c_api.cc:935: `ntree_limit` is deprecated, use `iteration_range` instead.
## [16:06:20] WARNING: src/c_api/c_api.cc:935: `ntree_limit` is deprecated, use `iteration_range` instead.
## [16:06:20] WARNING: src/c_api/c_api.cc:935: `ntree_limit` is deprecated, use `iteration_range` instead.
## [16:06:20] WARNING: src/c_api/c_api.cc:935: `ntree_limit` is deprecated, use `iteration_range` instead.
## [16:06:20] WARNING: src/c_api/c_api.cc:935: `ntree_limit` is deprecated, use `iteration_range` instead.
## [16:06:20] WARNING: src/c_api/c_api.cc:935: `ntree_limit` is deprecated, use `iteration_range` instead.
## [16:06:20] WARNING: src/c_api/c_api.cc:935: `ntree_limit` is deprecated, use `iteration_range` instead.
## [16:06:20] WARNING: src/c_api/c_api.cc:935: `ntree_limit` is deprecated, use `iteration_range` instead.
## [16:06:20] WARNING: src/c_api/c_api.cc:935: `ntree_limit` is deprecated, use `iteration_range` instead.
## [16:06:20] WARNING: src/c_api/c_api.cc:935: `ntree_limit` is deprecated, use `iteration_range` instead.
## [16:06:20] WARNING: src/c_api/c_api.cc:935: `ntree_limit` is deprecated, use `iteration_range` instead.
## [16:06:20] WARNING: src/c_api/c_api.cc:935: `ntree_limit` is deprecated, use `iteration_range` instead.
## [16:06:21] WARNING: src/c_api/c_api.cc:935: `ntree_limit` is deprecated, use `iteration_range` instead.
## [16:06:21] WARNING: src/c_api/c_api.cc:935: `ntree_limit` is deprecated, use `iteration_range` instead.
## [16:06:21] WARNING: src/c_api/c_api.cc:935: `ntree_limit` is deprecated, use `iteration_range` instead.
## [16:06:21] WARNING: src/c_api/c_api.cc:935: `ntree_limit` is deprecated, use `iteration_range` instead.
## [16:06:21] WARNING: src/c_api/c_api.cc:935: `ntree_limit` is deprecated, use `iteration_range` instead.
## [16:06:21] WARNING: src/c_api/c_api.cc:935: `ntree_limit` is deprecated, use `iteration_range` instead.
## [16:06:21] WARNING: src/c_api/c_api.cc:935: `ntree_limit` is deprecated, use `iteration_range` instead.
## [16:06:21] WARNING: src/c_api/c_api.cc:935: `ntree_limit` is deprecated, use `iteration_range` instead.
## [16:06:21] WARNING: src/c_api/c_api.cc:935: `ntree_limit` is deprecated, use `iteration_range` instead.
## [16:06:21] WARNING: src/c_api/c_api.cc:935: `ntree_limit` is deprecated, use `iteration_range` instead.
## [16:06:22] WARNING: src/c_api/c_api.cc:935: `ntree_limit` is deprecated, use `iteration_range` instead.
## [16:06:22] WARNING: src/c_api/c_api.cc:935: `ntree_limit` is deprecated, use `iteration_range` instead.
## [16:06:22] WARNING: src/c_api/c_api.cc:935: `ntree_limit` is deprecated, use `iteration_range` instead.
## [16:06:22] WARNING: src/c_api/c_api.cc:935: `ntree_limit` is deprecated, use `iteration_range` instead.
## [16:06:22] WARNING: src/c_api/c_api.cc:935: `ntree_limit` is deprecated, use `iteration_range` instead.
## [16:06:22] WARNING: src/c_api/c_api.cc:935: `ntree_limit` is deprecated, use `iteration_range` instead.
## [16:06:22] WARNING: src/c_api/c_api.cc:935: `ntree_limit` is deprecated, use `iteration_range` instead.
## [16:06:22] WARNING: src/c_api/c_api.cc:935: `ntree_limit` is deprecated, use `iteration_range` instead.
## [16:06:22] WARNING: src/c_api/c_api.cc:935: `ntree_limit` is deprecated, use `iteration_range` instead.
## [16:06:22] WARNING: src/c_api/c_api.cc:935: `ntree_limit` is deprecated, use `iteration_range` instead.
## [16:06:23] WARNING: src/c_api/c_api.cc:935: `ntree_limit` is deprecated, use `iteration_range` instead.
## [16:06:23] WARNING: src/c_api/c_api.cc:935: `ntree_limit` is deprecated, use `iteration_range` instead.
## [16:06:23] WARNING: src/c_api/c_api.cc:935: `ntree_limit` is deprecated, use `iteration_range` instead.
## [16:06:23] WARNING: src/c_api/c_api.cc:935: `ntree_limit` is deprecated, use `iteration_range` instead.
## [16:06:23] WARNING: src/c_api/c_api.cc:935: `ntree_limit` is deprecated, use `iteration_range` instead.
## [16:06:23] WARNING: src/c_api/c_api.cc:935: `ntree_limit` is deprecated, use `iteration_range` instead.
## [16:06:23] WARNING: src/c_api/c_api.cc:935: `ntree_limit` is deprecated, use `iteration_range` instead.
## [16:06:23] WARNING: src/c_api/c_api.cc:935: `ntree_limit` is deprecated, use `iteration_range` instead.
## [16:06:23] WARNING: src/c_api/c_api.cc:935: `ntree_limit` is deprecated, use `iteration_range` instead.
## [16:06:23] WARNING: src/c_api/c_api.cc:935: `ntree_limit` is deprecated, use `iteration_range` instead.
## [16:06:23] WARNING: src/c_api/c_api.cc:935: `ntree_limit` is deprecated, use `iteration_range` instead.
## [16:06:23] WARNING: src/c_api/c_api.cc:935: `ntree_limit` is deprecated, use `iteration_range` instead.
## [16:06:24] WARNING: src/c_api/c_api.cc:935: `ntree_limit` is deprecated, use `iteration_range` instead.
## [16:06:24] WARNING: src/c_api/c_api.cc:935: `ntree_limit` is deprecated, use `iteration_range` instead.
## [16:06:24] WARNING: src/c_api/c_api.cc:935: `ntree_limit` is deprecated, use `iteration_range` instead.
## [16:06:24] WARNING: src/c_api/c_api.cc:935: `ntree_limit` is deprecated, use `iteration_range` instead.
## [16:06:24] WARNING: src/c_api/c_api.cc:935: `ntree_limit` is deprecated, use `iteration_range` instead.
## [16:06:24] WARNING: src/c_api/c_api.cc:935: `ntree_limit` is deprecated, use `iteration_range` instead.
## [16:06:24] WARNING: src/c_api/c_api.cc:935: `ntree_limit` is deprecated, use `iteration_range` instead.
## [16:06:24] WARNING: src/c_api/c_api.cc:935: `ntree_limit` is deprecated, use `iteration_range` instead.
## [16:06:24] WARNING: src/c_api/c_api.cc:935: `ntree_limit` is deprecated, use `iteration_range` instead.
## [16:06:24] WARNING: src/c_api/c_api.cc:935: `ntree_limit` is deprecated, use `iteration_range` instead.
## [16:06:25] WARNING: src/c_api/c_api.cc:935: `ntree_limit` is deprecated, use `iteration_range` instead.
## [16:06:25] WARNING: src/c_api/c_api.cc:935: `ntree_limit` is deprecated, use `iteration_range` instead.
## [16:06:25] WARNING: src/c_api/c_api.cc:935: `ntree_limit` is deprecated, use `iteration_range` instead.
## [16:06:25] WARNING: src/c_api/c_api.cc:935: `ntree_limit` is deprecated, use `iteration_range` instead.
## [16:06:25] WARNING: src/c_api/c_api.cc:935: `ntree_limit` is deprecated, use `iteration_range` instead.
## [16:06:25] WARNING: src/c_api/c_api.cc:935: `ntree_limit` is deprecated, use `iteration_range` instead.
## [16:06:25] WARNING: src/c_api/c_api.cc:935: `ntree_limit` is deprecated, use `iteration_range` instead.
## [16:06:25] WARNING: src/c_api/c_api.cc:935: `ntree_limit` is deprecated, use `iteration_range` instead.
## [16:06:26] WARNING: src/c_api/c_api.cc:935: `ntree_limit` is deprecated, use `iteration_range` instead.
## [16:06:26] WARNING: src/c_api/c_api.cc:935: `ntree_limit` is deprecated, use `iteration_range` instead.
## [16:06:26] WARNING: src/c_api/c_api.cc:935: `ntree_limit` is deprecated, use `iteration_range` instead.
## [16:06:26] WARNING: src/c_api/c_api.cc:935: `ntree_limit` is deprecated, use `iteration_range` instead.
## [16:06:26] WARNING: src/c_api/c_api.cc:935: `ntree_limit` is deprecated, use `iteration_range` instead.
## [16:06:26] WARNING: src/c_api/c_api.cc:935: `ntree_limit` is deprecated, use `iteration_range` instead.
## [16:06:26] WARNING: src/c_api/c_api.cc:935: `ntree_limit` is deprecated, use `iteration_range` instead.
## [16:06:26] WARNING: src/c_api/c_api.cc:935: `ntree_limit` is deprecated, use `iteration_range` instead.
## [16:06:26] WARNING: src/c_api/c_api.cc:935: `ntree_limit` is deprecated, use `iteration_range` instead.
## [16:06:26] WARNING: src/c_api/c_api.cc:935: `ntree_limit` is deprecated, use `iteration_range` instead.
## [16:06:26] WARNING: src/c_api/c_api.cc:935: `ntree_limit` is deprecated, use `iteration_range` instead.
## [16:06:26] WARNING: src/c_api/c_api.cc:935: `ntree_limit` is deprecated, use `iteration_range` instead.
## [16:06:26] WARNING: src/c_api/c_api.cc:935: `ntree_limit` is deprecated, use `iteration_range` instead.
## [16:06:26] WARNING: src/c_api/c_api.cc:935: `ntree_limit` is deprecated, use `iteration_range` instead.
## [16:06:26] WARNING: src/c_api/c_api.cc:935: `ntree_limit` is deprecated, use `iteration_range` instead.
## [16:06:27] WARNING: src/c_api/c_api.cc:935: `ntree_limit` is deprecated, use `iteration_range` instead.
## [16:06:27] WARNING: src/c_api/c_api.cc:935: `ntree_limit` is deprecated, use `iteration_range` instead.
## [16:06:27] WARNING: src/c_api/c_api.cc:935: `ntree_limit` is deprecated, use `iteration_range` instead.
## [16:06:27] WARNING: src/c_api/c_api.cc:935: `ntree_limit` is deprecated, use `iteration_range` instead.
## [16:06:27] WARNING: src/c_api/c_api.cc:935: `ntree_limit` is deprecated, use `iteration_range` instead.
## [16:06:27] WARNING: src/c_api/c_api.cc:935: `ntree_limit` is deprecated, use `iteration_range` instead.
## [16:06:27] WARNING: src/c_api/c_api.cc:935: `ntree_limit` is deprecated, use `iteration_range` instead.
## [16:06:27] WARNING: src/c_api/c_api.cc:935: `ntree_limit` is deprecated, use `iteration_range` instead.
## [16:06:27] WARNING: src/c_api/c_api.cc:935: `ntree_limit` is deprecated, use `iteration_range` instead.
## [16:06:27] WARNING: src/c_api/c_api.cc:935: `ntree_limit` is deprecated, use `iteration_range` instead.
## [16:06:27] WARNING: src/c_api/c_api.cc:935: `ntree_limit` is deprecated, use `iteration_range` instead.
## [16:06:28] WARNING: src/c_api/c_api.cc:935: `ntree_limit` is deprecated, use `iteration_range` instead.
## [16:06:28] WARNING: src/c_api/c_api.cc:935: `ntree_limit` is deprecated, use `iteration_range` instead.
## [16:06:28] WARNING: src/c_api/c_api.cc:935: `ntree_limit` is deprecated, use `iteration_range` instead.
## [16:06:28] WARNING: src/c_api/c_api.cc:935: `ntree_limit` is deprecated, use `iteration_range` instead.
## [16:06:28] WARNING: src/c_api/c_api.cc:935: `ntree_limit` is deprecated, use `iteration_range` instead.
## [16:06:28] WARNING: src/c_api/c_api.cc:935: `ntree_limit` is deprecated, use `iteration_range` instead.
## [16:06:28] WARNING: src/c_api/c_api.cc:935: `ntree_limit` is deprecated, use `iteration_range` instead.
## [16:06:28] WARNING: src/c_api/c_api.cc:935: `ntree_limit` is deprecated, use `iteration_range` instead.
## [16:06:29] WARNING: src/c_api/c_api.cc:935: `ntree_limit` is deprecated, use `iteration_range` instead.
## [16:06:29] WARNING: src/c_api/c_api.cc:935: `ntree_limit` is deprecated, use `iteration_range` instead.
## [16:06:29] WARNING: src/c_api/c_api.cc:935: `ntree_limit` is deprecated, use `iteration_range` instead.
## [16:06:29] WARNING: src/c_api/c_api.cc:935: `ntree_limit` is deprecated, use `iteration_range` instead.
## [16:06:29] WARNING: src/c_api/c_api.cc:935: `ntree_limit` is deprecated, use `iteration_range` instead.
## [16:06:29] WARNING: src/c_api/c_api.cc:935: `ntree_limit` is deprecated, use `iteration_range` instead.
## [16:06:29] WARNING: src/c_api/c_api.cc:935: `ntree_limit` is deprecated, use `iteration_range` instead.
## [16:06:29] WARNING: src/c_api/c_api.cc:935: `ntree_limit` is deprecated, use `iteration_range` instead.
## [16:06:29] WARNING: src/c_api/c_api.cc:935: `ntree_limit` is deprecated, use `iteration_range` instead.
## [16:06:29] WARNING: src/c_api/c_api.cc:935: `ntree_limit` is deprecated, use `iteration_range` instead.
## [16:06:29] WARNING: src/c_api/c_api.cc:935: `ntree_limit` is deprecated, use `iteration_range` instead.
## [16:06:29] WARNING: src/c_api/c_api.cc:935: `ntree_limit` is deprecated, use `iteration_range` instead.
## [16:06:29] WARNING: src/c_api/c_api.cc:935: `ntree_limit` is deprecated, use `iteration_range` instead.
## [16:06:29] WARNING: src/c_api/c_api.cc:935: `ntree_limit` is deprecated, use `iteration_range` instead.
## [16:06:30] WARNING: src/c_api/c_api.cc:935: `ntree_limit` is deprecated, use `iteration_range` instead.
## [16:06:30] WARNING: src/c_api/c_api.cc:935: `ntree_limit` is deprecated, use `iteration_range` instead.
## [16:06:30] WARNING: src/c_api/c_api.cc:935: `ntree_limit` is deprecated, use `iteration_range` instead.
## [16:06:30] WARNING: src/c_api/c_api.cc:935: `ntree_limit` is deprecated, use `iteration_range` instead.
## [16:06:30] WARNING: src/c_api/c_api.cc:935: `ntree_limit` is deprecated, use `iteration_range` instead.
## [16:06:30] WARNING: src/c_api/c_api.cc:935: `ntree_limit` is deprecated, use `iteration_range` instead.
## [16:06:30] WARNING: src/c_api/c_api.cc:935: `ntree_limit` is deprecated, use `iteration_range` instead.
## [16:06:30] WARNING: src/c_api/c_api.cc:935: `ntree_limit` is deprecated, use `iteration_range` instead.
## [16:06:30] WARNING: src/c_api/c_api.cc:935: `ntree_limit` is deprecated, use `iteration_range` instead.
## [16:06:30] WARNING: src/c_api/c_api.cc:935: `ntree_limit` is deprecated, use `iteration_range` instead.
## [16:06:31] WARNING: src/c_api/c_api.cc:935: `ntree_limit` is deprecated, use `iteration_range` instead.
## [16:06:31] WARNING: src/c_api/c_api.cc:935: `ntree_limit` is deprecated, use `iteration_range` instead.
## [16:06:31] WARNING: src/c_api/c_api.cc:935: `ntree_limit` is deprecated, use `iteration_range` instead.
## [16:06:31] WARNING: src/c_api/c_api.cc:935: `ntree_limit` is deprecated, use `iteration_range` instead.
## [16:06:31] WARNING: src/c_api/c_api.cc:935: `ntree_limit` is deprecated, use `iteration_range` instead.
## [16:06:31] WARNING: src/c_api/c_api.cc:935: `ntree_limit` is deprecated, use `iteration_range` instead.
## [16:06:31] WARNING: src/c_api/c_api.cc:935: `ntree_limit` is deprecated, use `iteration_range` instead.
## [16:06:31] WARNING: src/c_api/c_api.cc:935: `ntree_limit` is deprecated, use `iteration_range` instead.
## [16:06:31] WARNING: src/c_api/c_api.cc:935: `ntree_limit` is deprecated, use `iteration_range` instead.
## [16:06:31] WARNING: src/c_api/c_api.cc:935: `ntree_limit` is deprecated, use `iteration_range` instead.
## [16:06:32] WARNING: src/c_api/c_api.cc:935: `ntree_limit` is deprecated, use `iteration_range` instead.
## [16:06:32] WARNING: src/c_api/c_api.cc:935: `ntree_limit` is deprecated, use `iteration_range` instead.
## [16:06:32] WARNING: src/c_api/c_api.cc:935: `ntree_limit` is deprecated, use `iteration_range` instead.
## [16:06:32] WARNING: src/c_api/c_api.cc:935: `ntree_limit` is deprecated, use `iteration_range` instead.
## [16:06:32] WARNING: src/c_api/c_api.cc:935: `ntree_limit` is deprecated, use `iteration_range` instead.
## [16:06:32] WARNING: src/c_api/c_api.cc:935: `ntree_limit` is deprecated, use `iteration_range` instead.
## [16:06:32] WARNING: src/c_api/c_api.cc:935: `ntree_limit` is deprecated, use `iteration_range` instead.
## [16:06:32] WARNING: src/c_api/c_api.cc:935: `ntree_limit` is deprecated, use `iteration_range` instead.
## [16:06:32] WARNING: src/c_api/c_api.cc:935: `ntree_limit` is deprecated, use `iteration_range` instead.
## [16:06:32] WARNING: src/c_api/c_api.cc:935: `ntree_limit` is deprecated, use `iteration_range` instead.
## [16:06:33] WARNING: src/c_api/c_api.cc:935: `ntree_limit` is deprecated, use `iteration_range` instead.
## [16:06:33] WARNING: src/c_api/c_api.cc:935: `ntree_limit` is deprecated, use `iteration_range` instead.
## [16:06:33] WARNING: src/c_api/c_api.cc:935: `ntree_limit` is deprecated, use `iteration_range` instead.
## [16:06:33] WARNING: src/c_api/c_api.cc:935: `ntree_limit` is deprecated, use `iteration_range` instead.
## [16:06:33] WARNING: src/c_api/c_api.cc:935: `ntree_limit` is deprecated, use `iteration_range` instead.
## [16:06:33] WARNING: src/c_api/c_api.cc:935: `ntree_limit` is deprecated, use `iteration_range` instead.
## [16:06:33] WARNING: src/c_api/c_api.cc:935: `ntree_limit` is deprecated, use `iteration_range` instead.
## [16:06:33] WARNING: src/c_api/c_api.cc:935: `ntree_limit` is deprecated, use `iteration_range` instead.
## [16:06:33] WARNING: src/c_api/c_api.cc:935: `ntree_limit` is deprecated, use `iteration_range` instead.
## [16:06:33] WARNING: src/c_api/c_api.cc:935: `ntree_limit` is deprecated, use `iteration_range` instead.
## [16:06:33] WARNING: src/c_api/c_api.cc:935: `ntree_limit` is deprecated, use `iteration_range` instead.
## [16:06:33] WARNING: src/c_api/c_api.cc:935: `ntree_limit` is deprecated, use `iteration_range` instead.
## [16:06:33] WARNING: src/c_api/c_api.cc:935: `ntree_limit` is deprecated, use `iteration_range` instead.
## [16:06:33] WARNING: src/c_api/c_api.cc:935: `ntree_limit` is deprecated, use `iteration_range` instead.
## [16:06:34] WARNING: src/c_api/c_api.cc:935: `ntree_limit` is deprecated, use `iteration_range` instead.
## [16:06:34] WARNING: src/c_api/c_api.cc:935: `ntree_limit` is deprecated, use `iteration_range` instead.
## [16:06:34] WARNING: src/c_api/c_api.cc:935: `ntree_limit` is deprecated, use `iteration_range` instead.
## [16:06:34] WARNING: src/c_api/c_api.cc:935: `ntree_limit` is deprecated, use `iteration_range` instead.
## [16:06:34] WARNING: src/c_api/c_api.cc:935: `ntree_limit` is deprecated, use `iteration_range` instead.
## [16:06:34] WARNING: src/c_api/c_api.cc:935: `ntree_limit` is deprecated, use `iteration_range` instead.
## [16:06:34] WARNING: src/c_api/c_api.cc:935: `ntree_limit` is deprecated, use `iteration_range` instead.
## [16:06:34] WARNING: src/c_api/c_api.cc:935: `ntree_limit` is deprecated, use `iteration_range` instead.
## [16:06:34] WARNING: src/c_api/c_api.cc:935: `ntree_limit` is deprecated, use `iteration_range` instead.
## [16:06:34] WARNING: src/c_api/c_api.cc:935: `ntree_limit` is deprecated, use `iteration_range` instead.
## [16:06:35] WARNING: src/c_api/c_api.cc:935: `ntree_limit` is deprecated, use `iteration_range` instead.
## [16:06:35] WARNING: src/c_api/c_api.cc:935: `ntree_limit` is deprecated, use `iteration_range` instead.
## [16:06:35] WARNING: src/c_api/c_api.cc:935: `ntree_limit` is deprecated, use `iteration_range` instead.
## [16:06:35] WARNING: src/c_api/c_api.cc:935: `ntree_limit` is deprecated, use `iteration_range` instead.
## [16:06:35] WARNING: src/c_api/c_api.cc:935: `ntree_limit` is deprecated, use `iteration_range` instead.
## [16:06:35] WARNING: src/c_api/c_api.cc:935: `ntree_limit` is deprecated, use `iteration_range` instead.
## [16:06:35] WARNING: src/c_api/c_api.cc:935: `ntree_limit` is deprecated, use `iteration_range` instead.
## [16:06:35] WARNING: src/c_api/c_api.cc:935: `ntree_limit` is deprecated, use `iteration_range` instead.
## [16:06:35] WARNING: src/c_api/c_api.cc:935: `ntree_limit` is deprecated, use `iteration_range` instead.
## [16:06:35] WARNING: src/c_api/c_api.cc:935: `ntree_limit` is deprecated, use `iteration_range` instead.
## [16:06:36] WARNING: src/c_api/c_api.cc:935: `ntree_limit` is deprecated, use `iteration_range` instead.
## [16:06:36] WARNING: src/c_api/c_api.cc:935: `ntree_limit` is deprecated, use `iteration_range` instead.
## [16:06:36] WARNING: src/c_api/c_api.cc:935: `ntree_limit` is deprecated, use `iteration_range` instead.
## [16:06:36] WARNING: src/c_api/c_api.cc:935: `ntree_limit` is deprecated, use `iteration_range` instead.
## [16:06:36] WARNING: src/c_api/c_api.cc:935: `ntree_limit` is deprecated, use `iteration_range` instead.
## [16:06:36] WARNING: src/c_api/c_api.cc:935: `ntree_limit` is deprecated, use `iteration_range` instead.
## [16:06:36] WARNING: src/c_api/c_api.cc:935: `ntree_limit` is deprecated, use `iteration_range` instead.
## [16:06:36] WARNING: src/c_api/c_api.cc:935: `ntree_limit` is deprecated, use `iteration_range` instead.
## [16:06:36] WARNING: src/c_api/c_api.cc:935: `ntree_limit` is deprecated, use `iteration_range` instead.
## [16:06:36] WARNING: src/c_api/c_api.cc:935: `ntree_limit` is deprecated, use `iteration_range` instead.
## [16:06:36] WARNING: src/c_api/c_api.cc:935: `ntree_limit` is deprecated, use `iteration_range` instead.
## [16:06:36] WARNING: src/c_api/c_api.cc:935: `ntree_limit` is deprecated, use `iteration_range` instead.
## [16:06:36] WARNING: src/c_api/c_api.cc:935: `ntree_limit` is deprecated, use `iteration_range` instead.
## [16:06:36] WARNING: src/c_api/c_api.cc:935: `ntree_limit` is deprecated, use `iteration_range` instead.
## [16:06:37] WARNING: src/c_api/c_api.cc:935: `ntree_limit` is deprecated, use `iteration_range` instead.
## [16:06:37] WARNING: src/c_api/c_api.cc:935: `ntree_limit` is deprecated, use `iteration_range` instead.
## [16:06:37] WARNING: src/c_api/c_api.cc:935: `ntree_limit` is deprecated, use `iteration_range` instead.
## [16:06:37] WARNING: src/c_api/c_api.cc:935: `ntree_limit` is deprecated, use `iteration_range` instead.
## [16:06:37] WARNING: src/c_api/c_api.cc:935: `ntree_limit` is deprecated, use `iteration_range` instead.
## [16:06:37] WARNING: src/c_api/c_api.cc:935: `ntree_limit` is deprecated, use `iteration_range` instead.
## [16:06:37] WARNING: src/c_api/c_api.cc:935: `ntree_limit` is deprecated, use `iteration_range` instead.
## [16:06:37] WARNING: src/c_api/c_api.cc:935: `ntree_limit` is deprecated, use `iteration_range` instead.
## [16:06:37] WARNING: src/c_api/c_api.cc:935: `ntree_limit` is deprecated, use `iteration_range` instead.
## [16:06:37] WARNING: src/c_api/c_api.cc:935: `ntree_limit` is deprecated, use `iteration_range` instead.
## [16:06:38] WARNING: src/c_api/c_api.cc:935: `ntree_limit` is deprecated, use `iteration_range` instead.
## [16:06:38] WARNING: src/c_api/c_api.cc:935: `ntree_limit` is deprecated, use `iteration_range` instead.
## [16:06:38] WARNING: src/c_api/c_api.cc:935: `ntree_limit` is deprecated, use `iteration_range` instead.
## [16:06:38] WARNING: src/c_api/c_api.cc:935: `ntree_limit` is deprecated, use `iteration_range` instead.
## [16:06:38] WARNING: src/c_api/c_api.cc:935: `ntree_limit` is deprecated, use `iteration_range` instead.
## [16:06:38] WARNING: src/c_api/c_api.cc:935: `ntree_limit` is deprecated, use `iteration_range` instead.
## [16:06:38] WARNING: src/c_api/c_api.cc:935: `ntree_limit` is deprecated, use `iteration_range` instead.
## [16:06:38] WARNING: src/c_api/c_api.cc:935: `ntree_limit` is deprecated, use `iteration_range` instead.
## [16:06:39] WARNING: src/c_api/c_api.cc:935: `ntree_limit` is deprecated, use `iteration_range` instead.
## [16:06:39] WARNING: src/c_api/c_api.cc:935: `ntree_limit` is deprecated, use `iteration_range` instead.
## [16:06:39] WARNING: src/c_api/c_api.cc:935: `ntree_limit` is deprecated, use `iteration_range` instead.
## [16:06:39] WARNING: src/c_api/c_api.cc:935: `ntree_limit` is deprecated, use `iteration_range` instead.
## [16:06:39] WARNING: src/c_api/c_api.cc:935: `ntree_limit` is deprecated, use `iteration_range` instead.
## [16:06:39] WARNING: src/c_api/c_api.cc:935: `ntree_limit` is deprecated, use `iteration_range` instead.
## [16:06:39] WARNING: src/c_api/c_api.cc:935: `ntree_limit` is deprecated, use `iteration_range` instead.
## [16:06:39] WARNING: src/c_api/c_api.cc:935: `ntree_limit` is deprecated, use `iteration_range` instead.
## [16:06:39] WARNING: src/c_api/c_api.cc:935: `ntree_limit` is deprecated, use `iteration_range` instead.
## [16:06:39] WARNING: src/c_api/c_api.cc:935: `ntree_limit` is deprecated, use `iteration_range` instead.
## [16:06:39] WARNING: src/c_api/c_api.cc:935: `ntree_limit` is deprecated, use `iteration_range` instead.
## [16:06:39] WARNING: src/c_api/c_api.cc:935: `ntree_limit` is deprecated, use `iteration_range` instead.
## [16:06:40] WARNING: src/c_api/c_api.cc:935: `ntree_limit` is deprecated, use `iteration_range` instead.
## [16:06:40] WARNING: src/c_api/c_api.cc:935: `ntree_limit` is deprecated, use `iteration_range` instead.
## [16:06:40] WARNING: src/c_api/c_api.cc:935: `ntree_limit` is deprecated, use `iteration_range` instead.
## [16:06:40] WARNING: src/c_api/c_api.cc:935: `ntree_limit` is deprecated, use `iteration_range` instead.
## [16:06:40] WARNING: src/c_api/c_api.cc:935: `ntree_limit` is deprecated, use `iteration_range` instead.
## [16:06:40] WARNING: src/c_api/c_api.cc:935: `ntree_limit` is deprecated, use `iteration_range` instead.
## [16:06:40] WARNING: src/c_api/c_api.cc:935: `ntree_limit` is deprecated, use `iteration_range` instead.
## [16:06:40] WARNING: src/c_api/c_api.cc:935: `ntree_limit` is deprecated, use `iteration_range` instead.
## [16:06:40] WARNING: src/c_api/c_api.cc:935: `ntree_limit` is deprecated, use `iteration_range` instead.
## [16:06:40] WARNING: src/c_api/c_api.cc:935: `ntree_limit` is deprecated, use `iteration_range` instead.
## [16:06:40] WARNING: src/c_api/c_api.cc:935: `ntree_limit` is deprecated, use `iteration_range` instead.
## [16:06:40] WARNING: src/c_api/c_api.cc:935: `ntree_limit` is deprecated, use `iteration_range` instead.
## [16:06:41] WARNING: src/c_api/c_api.cc:935: `ntree_limit` is deprecated, use `iteration_range` instead.
## [16:06:41] WARNING: src/c_api/c_api.cc:935: `ntree_limit` is deprecated, use `iteration_range` instead.
## [16:06:41] WARNING: src/c_api/c_api.cc:935: `ntree_limit` is deprecated, use `iteration_range` instead.
## [16:06:41] WARNING: src/c_api/c_api.cc:935: `ntree_limit` is deprecated, use `iteration_range` instead.
## [16:06:41] WARNING: src/c_api/c_api.cc:935: `ntree_limit` is deprecated, use `iteration_range` instead.
## [16:06:41] WARNING: src/c_api/c_api.cc:935: `ntree_limit` is deprecated, use `iteration_range` instead.
## [16:06:41] WARNING: src/c_api/c_api.cc:935: `ntree_limit` is deprecated, use `iteration_range` instead.
## [16:06:41] WARNING: src/c_api/c_api.cc:935: `ntree_limit` is deprecated, use `iteration_range` instead.
## [16:06:41] WARNING: src/c_api/c_api.cc:935: `ntree_limit` is deprecated, use `iteration_range` instead.
## [16:06:41] WARNING: src/c_api/c_api.cc:935: `ntree_limit` is deprecated, use `iteration_range` instead.
## [16:06:42] WARNING: src/c_api/c_api.cc:935: `ntree_limit` is deprecated, use `iteration_range` instead.
## [16:06:42] WARNING: src/c_api/c_api.cc:935: `ntree_limit` is deprecated, use `iteration_range` instead.
## [16:06:42] WARNING: src/c_api/c_api.cc:935: `ntree_limit` is deprecated, use `iteration_range` instead.
## [16:06:42] WARNING: src/c_api/c_api.cc:935: `ntree_limit` is deprecated, use `iteration_range` instead.
## [16:06:42] WARNING: src/c_api/c_api.cc:935: `ntree_limit` is deprecated, use `iteration_range` instead.
## [16:06:42] WARNING: src/c_api/c_api.cc:935: `ntree_limit` is deprecated, use `iteration_range` instead.
```

## Comparing the predicted RTs with the experimental retention times

```
ggplot(data = data_prediction) +
  geom_point(mapping = aes(x = RTI,
                           y = RTI_xgbTREE_pred),
                  color = highlightercolor6) +
        facet_wrap(~CS_code) +
        ylim(0, 1000) +
        xlim(0, 1000) +
        labs(x = "RTI measured", y = "RTI pred") +
        my_theme +
  theme(axis.text.x = element_text(angle = 90))
```

Statistical overview of the results

```
data_summary = data_prediction %>%
  na.omit() %>%
  group_by(CS_code, nr_points) %>%
  summarize(RMSE = rmse(RTI, RTI_xgbTREE_pred),
            MAD = mad(RTI, RTI_xgbTREE_pred),
            Q95 = quantile(abs(RTI - RTI_xgbTREE_pred), probs = c(0.95)),
            R2 = cor(RTI, RTI_xgbTREE_pred)^2) %>%
  ungroup() %>%
  arrange(RMSE)

data_summary
```

```
## # A tibble: 37 × 6
##    CS_code nr_points  RMSE   MAD   Q95    R2
##    <chr>       <int> <dbl> <dbl> <dbl> <dbl>
##  1 DS_JBQ         33  131.  114.  263. 0.534
##  2 DS_JSG         33  138.  148.  253. 0.617
##  3 DS_QBD         29  139.  163.  261. 0.558
##  4 DS_BIJ         28  140.  142.  273. 0.606
##  5 DS_Q           28  146.  190.  259. 0.480
##  6 DS_TJQ         26  150.  172.  240. 0.753
##  7 DS_QJS         20  152.  147.  333. 0.501
##  8 DS_QQT         26  155.  168.  250. 0.594
##  9 DS_QDF         33  156.  188.  287. 0.404
## 10 DS_AWW         29  163.  166.  281. 0.450
## # ℹ 27 more rows
```

```
cor.test(data_summary$nr_points, data_summary$RMSE)
```

```
## 
##  Pearson's product-moment correlation
## 
## data:  data_summary$nr_points and data_summary$RMSE
## t = -4.1666, df = 35, p-value = 0.0001923
## alternative hypothesis: true correlation is not equal to 0
## 95 percent confidence interval:
##  -0.7583440 -0.3095469
## sample estimates:
##        cor 
## -0.5758124
```

# Prediction+Projection vs Training on Small dataset

Reading in both data

```
data_large = read_delim("results/NORMAN_RTI_prediction_results.csv") 
data_large = data_large %>%
  left_join(data %>%
              select(compound, SMILES) %>%
              unique()) %>%
  rename(RTI_pred_large = RTI_pred) %>%
  filter(sample_type == "sus")
```

```
data_small = data_prediction %>%
  rename(RTI_pred_small = RTI_xgbTREE_pred)
```

```
data_comb = data_large %>% 
  left_join(data_small) %>%
  na.omit()
```

Statistical comparison of the models.

```
data_summary = data_comb %>%
  na.omit() %>%
  group_by(CS_code, nr_points) %>%
  summarize(RMSE_small = rmse(RTI, RTI_pred_small),
            RMSE_large = rmse(RTI, RTI_pred_large),
            better = case_when(
              RMSE_small < RMSE_large ~ "small_model",
              TRUE ~ "large_model"
            )) %>%
  ungroup() %>%
  arrange(better, RMSE_small)
```

```
results_var_test = tibble()
for(CS_this in levels(factor(data_comb$CS_code))) {
    data_this = data_comb %>%
      filter(CS_code == CS_this) %>%
      na.omit()
    
    result = var.test(lm(RTI ~ 0 + offset(1*RTI_pred_small), 
                         data = data_this), 
                      lm(RTI ~ 0 + offset(1*RTI_pred_large), 
                         data = data_this))

    results_var_test = results_var_test %>%
      bind_rows(tibble(CS_code = CS_this,
                       p_value = result$p.value))
}
```

```
data_summary = data_summary %>%
  left_join(results_var_test) %>%
  arrange(better, p_value)
```

```
## Joining with `by = join_by(CS_code)`
```

```
data_summary %>%
  group_by(better) %>%
  summarize(count_sign = sum(ifelse(p_value < 0.05, 1, 0)),
            count_insign = sum(ifelse(p_value >= 0.05, 1, 0))) %>%
  ungroup()
```

```
## # A tibble: 2 × 3
##   better      count_sign count_insign
##   <chr>            <dbl>        <dbl>
## 1 large_model         11           22
## 2 small_model          0            4
```
